# Photocatalytic decarboxylative amidosulfonation enables direct transformation of carboxylic acids to sulfonamides†

**DOI:** 10.1039/d1sc01389k

**Published:** 2021-04-13

**Authors:** Vu T. Nguyen, Graham C. Haug, Viet D. Nguyen, Ngan T. H. Vuong, Hadi D. Arman, Oleg V. Larionov

**Affiliations:** Department of Chemistry, The University of Texas at San Antonio One UTSA Circle San Antonio TX 78249 USA oleg.larionov@utsa.edu

## Abstract

Sulfonamides feature prominently in organic synthesis, materials science and medicinal chemistry, where they play important roles as bioisosteric replacements of carboxylic acids and other carbonyls. Yet, a general synthetic platform for the direct conversion of carboxylic acids to a range of functionalized sulfonamides has remained elusive. Herein, we present a visible light-induced, dual catalytic platform that for the first time allows for a one-step access to sulfonamides and sulfonyl azides directly from carboxylic acids. The broad scope of the direct decarboxylative amidosulfonation (DDAS) platform is enabled by the efficient direct conversion of carboxylic acids to sulfinic acids that is catalyzed by acridine photocatalysts and interfaced with copper-catalyzed sulfur–nitrogen bond-forming cross-couplings with both electrophilic and nucleophilic reagents.

## Abstract 2

Sulfonamides are now accessible directly from carboxylic acids by a one-step, tricomponent decarboxylative amidosulfonation that provides the missing link between the two key functionalities.

## Graphical abstract

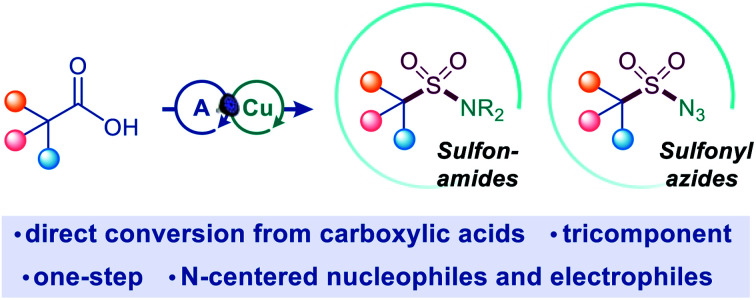

## Introduction

**Fig. 1:**
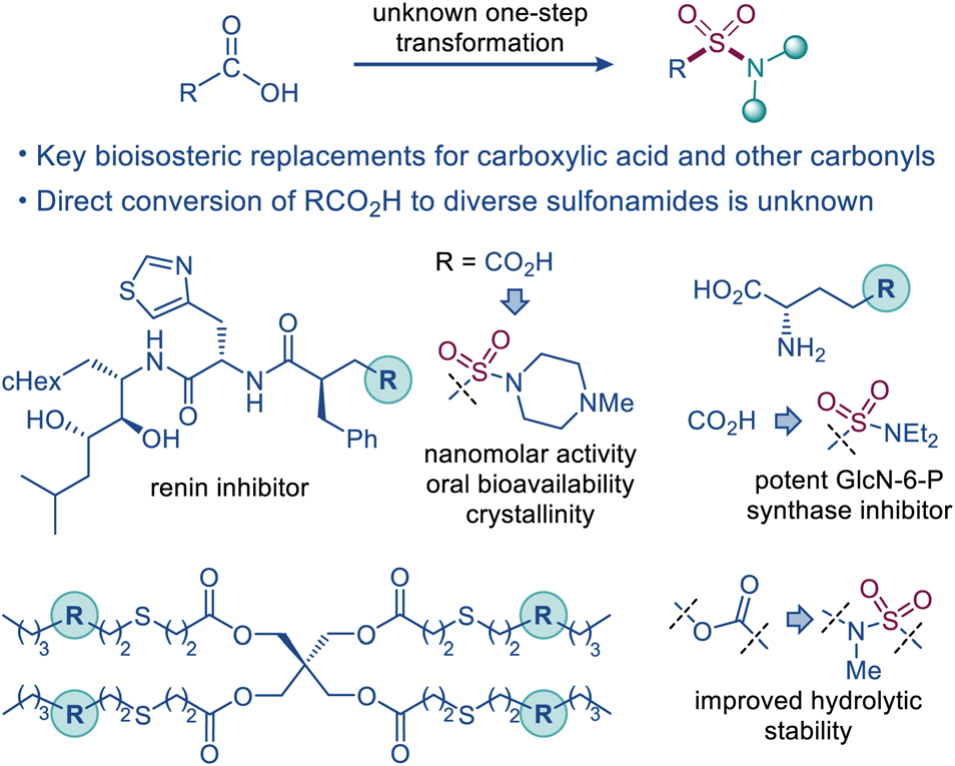
Decarboxylative amidosulfonation as an enabling synthetic tool for the direct conversion of carboxylic acids to sulfonamides.

The sulfonamide group is one the most centrally important functionalities.^1^ In drug discovery, the sulfonamide group occupies a preeminent position, due to the favorable properties that include a pronounced electron withdrawing character, hydrolytic stability, polarity, hydrogen bonding ability, and resistance to reduction and oxidation. The combination of the favorable properties has made sulfonamide-containing groups some of the most widely used bioisosteric replacements for a broad range of more reactive and labile functionalities, *e.g.*, ketone, ether, and especially carboxylic acid moieties, as, for example, the distance between the two oxygen atoms in carboxylate and sulfonamide is virtually identical (2.1–2.3 Å).^2^ Remarkably, although the sulfonamide group has been used as a bioisosteric replacement for the carboxylic acid moiety since 1930s, predating Friedman's definition of bioisosterism,^2*a*,3^ the direct decarboxylative conversion of carboxylic acids to sulfonamides has remained elusive (Fig. 1).

The one-step decarboxylative amidosulfonation will be particularly important for the construction of C(sp^3^)–sulfonamide bonds, since they cannot be accessed by one-step methods developed for the C(sp^2^)–sulfonamide bond formation, *e.g.*, Sandmeyer-type reactions and transition metal-catalyzed cross-couplings.^4^ New reactions that produce aliphatic sulfonamides from a previously inaccessible precursor chemical space in an operationally uninterrupted manner will increase the structural diversity and facilitate synthetic access to C(sp^3^)–sulfonamide bonds.

In addition, aliphatic carboxylic acids are abundant feedstock materials that are readily available from natural sources and industrial precursors, while their central position in drug discovery, agroscience, and materials chemistry^5^ warrants the development of a reaction that enables direct conversion of carboxylic acids to diverse bioisosteric and structurally analogous sulfonamides to streamline library synthesis, lead optimization, and process chemistry, and improve access to new functionalized materials.^6^ A broad-scope decarboxylative amidosulfonation will also facilitate direct access to other SO_2_–N functionalities, *e.g.*, sulfonyl azides that are versatile reagents with applications in materials synthesis and chemical biology.^7^

**Fig. 2:**
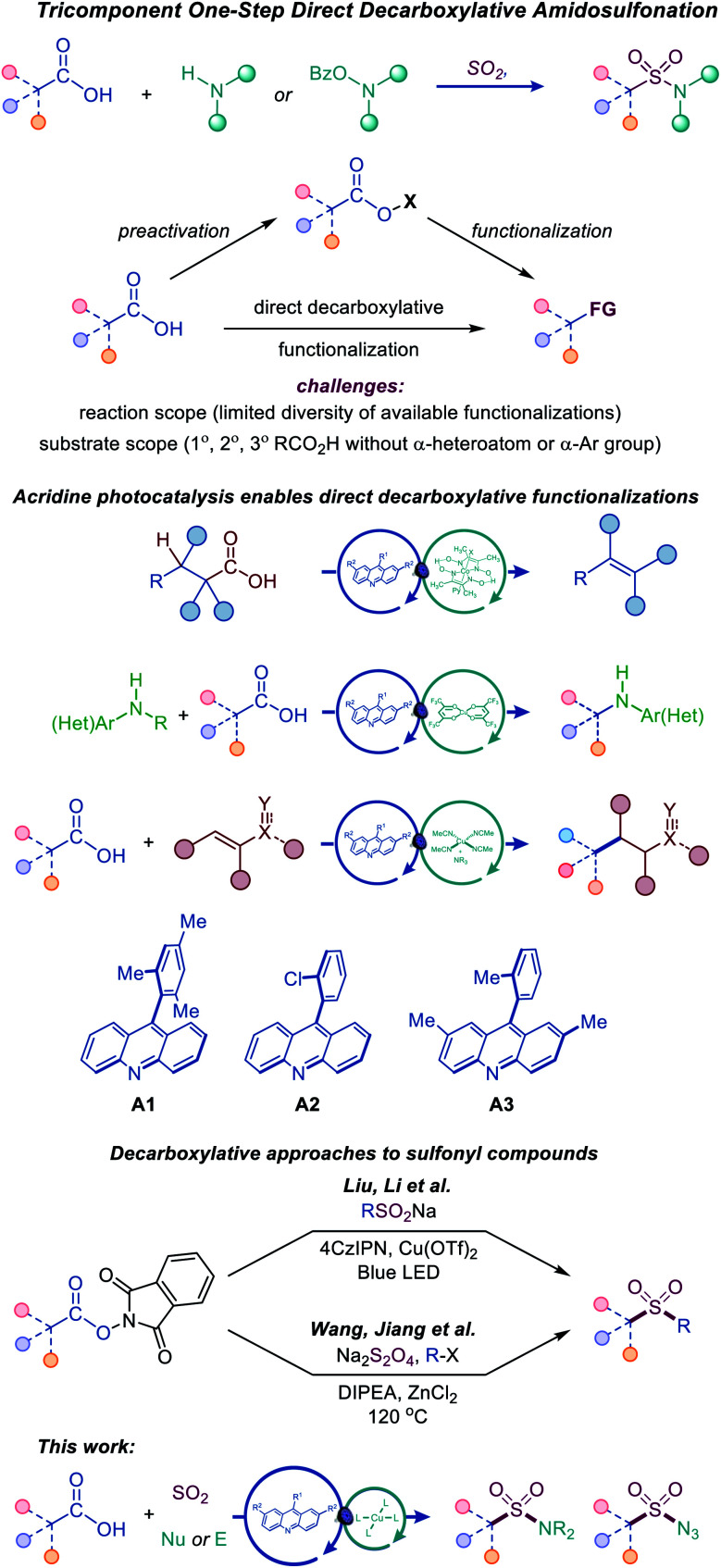
Tricomponent synthesis of sulfonamides and sulfonyl azides by the dual catalytic direct decarboxylative amidosulfonation.

Photocatalytic decarboxylative functionalizations are typically accomplished in a stepwise manner by first converting carboxylic acids to redox-active derivatives, *e.g.*, *N*-hydroxyphthalimide or hypervalent iodine esters, because they provide a workaround for the challenging direct oxidation of carboxylic acids that occurs at prohibitively high oxidation potentials (Fig. 2).^8^ Direct decarboxylation of carboxylic acids can enable a decarboxylative radical generation in one step and without prior derivatization. However, direct decarboxylation of unactivated carboxylic acids that do not bear stabilizing α-heteroatoms or aromatic groups remains challenging, because of the high oxidation potentials of carboxylic acids and carboxylates, requiring strong photooxidants that are unsuitable for easily oxidizable functionalities, *e.g.*, sulfinates, anilines, alcohols, and electron-rich heterocycles. Additionally, photocatalytic direct decarboxylation may be more difficult to interface with transition metal-catalyzed processes, due to the reactivity of the carboxylic group. Consequently, the scope of transformations that can be accomplished by photocatalytic direct decarboxylation of diverse carboxylic acids has remained narrow.^8^ Photocatalytic systems that selectively induce direct decarboxylation of carboxylic acids without affecting other oxidizable functionalities and are compatible with a wide range of uncatalyzed and catalytic functionalizations of the nascent radical intermediate have the potential to provide broadly applicable synthetic platforms for construction of carbon–carbon and carbon–heteroatom bonds. Accordingly, further work is needed to develop such photocatalytic systems and expand the scope, both with respect to substrates and the diversity of transformations. In particular, mechanistically novel photocatalytic systems that are based on hydrogen bonding can provide the directional character that is necessary for improving chemoselectivity and facilitating new transformations. We recently described a photocatalytic system based on the 9-arylacridine structure (**A1–A3**), enabling visible light-induced direct decarboxylative dehydrodecarboxylation, amination and conjugate addition reactions of unactivated primary, secondary and tertiary alkylcarboxylic acids (Fig. 2).^9^

The role of the photocatalytic system is particularly critical for the development of the direct decarboxylative amidosulfonation, as it involves sulfinate intermediates. Since sulfinates are more readily oxidizable than carboxylates (*e.g.*, *E*_ox_ = 0.3 V *vs.* SCE for CH_3_SO_2_NBu_4_ and 1.28 V for CH_3_CO_2_NBu_4_ in MeCN), photocatalytic systems operating by a single electron oxidation of carboxylate anions are expected to be unsuitable for the development of the DDAS platform.

Recent examples of construction of sulfones from redox-active *N*-hydroxyphthalimide esters by Wang and Jiang,^10^ and Liu and Li^11^ have highlighted the synthetic potential of the decarboxylative approach to installation of sulfonyl-containing functionalities (Fig. 2).

We report herein the development of a simple dual catalytic system for the first direct decarboxylative amidosulfonation (DDAS) of carboxylic acids that can be adapted to access *N*-alkyl and *N*-aryl sulfonamides, as well as sulfonyl azides in one step and in a multicomponent fashion, *i.e.*, with concomitant formation of the C–SO_2_ and O_2_S–N bonds by directly combining carboxylic acids with a sulfur dioxide source and corresponding nucleophilic and electrophilic N-centered coupling partners in the presence of acridine photocatalysts **A1–A3** that proved to be uniquely active in enabling this new reaction. This study provides the first example of an adaptive decarboxylative amidosulfonation platform that can be readily adjusted to produce a divergent array of SO_2_–N functionalities from a common carboxylic precursor and offers a mechanistic rationale for the observed dual catalytic activity.

## Results and discussion

We first focused on the direct conversion of carboxylic acids to *N*-alkyl sulfonamides, hypothesizing that *O*-benzoylhydroxylamines could serve as readily available N-centered coupling partners. After initial optimization studies, we found that palmitic acid can be efficiently converted to the corresponding sulfonamide **1a** in a tricomponent reaction with *N*-BzO-morpholine and DABSO (DABCO–bis(sulfur dioxide) adduct)^12^ in the presence of acridine catalyst **A1** with 400 or 420 nm LED irradiation (Table 1, entries 1 and 2), providing the first example of direct decarboxylative amidosulfonation and catalytic S–N bond formation with *O*-benzoylhydroxylamines. The reaction did not proceed without light and the acridine catalyst (entries 3 and 4). Other acridine catalysts **A2** and **A3** provided lower catalytic performance (entries 5 and 6). Importantly, other classes of photocatalysts (*e.g.*, *N*-methyl 9-mesitylacridinium catalyst [Acr-Mes]^+^(ClO_4_)^−^, Ir- and Ru-based photocatalysts, and 4CzIPN) failed to deliver the sulfonamide product (entry 7 and Table S1†). While copper(ii) fluoride emerged as the most efficient pre-catalyst, other Cu^I^ and Cu^II^ salts (entries 8 and 9) also promoted amidosulfonation, pointing to a common in situ-formed catalytically active Cu species.

**Table 1:** Reaction conditions for the photocatalytic direct decarboxylative amidosulfonation^a^

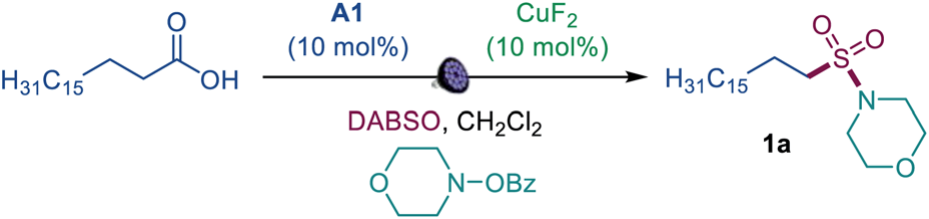		
Entry	Change from optimal conditions	Yield, %
1	None	92 (89^b^)
2	420 nm instead of 400 nm LED	95
3	No light	0
4	No **A1**	0
5	**A2** instead of **A1**	81
6	**A3** instead of **A2**	59
7	[Acr-Mes]^+^(ClO_4_)^−^ instead of **A1**	0
8	Cu(OAc)_2_ instead of CuF_2_	88
9	CuOAc instead of CuF_2_	87

a Reaction conditions: carboxylic acid (0.3 mmol), DABSO (0.33 mmol), **A1** (10 mol%), CuF_2_ (10 mol%) *O*-benzoylhydroxylamine (0.6 mmol), CH_2_Cl_2_ (6 mL), LED light (400 nm), 12 h. Yield was determined by ^1^H NMR spectroscopy with 1,4-dimethoxybenzene as an internal standard.

b Isolated yield. DABSO = O_2_S–N(CH_2_CH_2_)_3_N–SO_2_. [Acr-Mes]^+^(ClO_4_)^−^ = *N*-methyl 9-mesitylacridinium perchlorate.

**Scheme 1:**
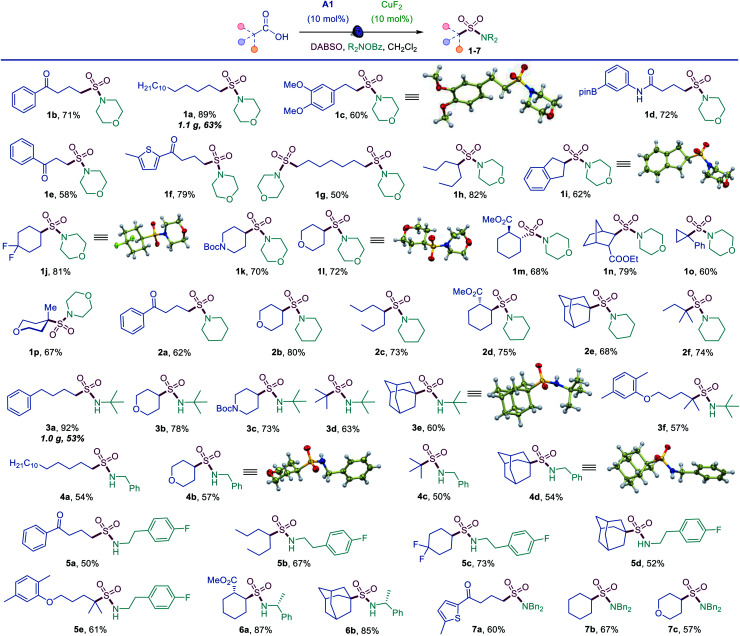
Scope of the direct decarboxylative amidosulfonation with *O*-benzylhydoxylamines. Reaction conditions: carboxylic acid (0.15–0.3 mmol), DABSO (0.33 mmol), **A1** (10 mol%), CuF_2_ (10 mol%) *O*-benzoylhydroxylamine (0.6 mmol), CH_2_Cl_2_ (6 mL), LED light (400 nm).

The simplicity of the direct decarboxylative amidosulfonation provided an opportunity to carry out an initial evaluation of the substrate scope with morpholine as a common amine unit that revealed broad compatibility and efficiency with a wide array of functionalized carboxylic acids (sulfonamides **1b–1p**, Scheme 1). An assortment of diverse functional groups were well tolerated, including electron rich aryl and boryl groups (**1c**, **1i** and **1d**). The reaction can be used for a simultaneous installation of two sulfonamide groups (**1g**) and performs well with a variety of cyclic acids (**1i–1p**), proceeding highly stereoselectively in the norbornane and cyclohexane series (**1m**, **1n**). Sulfonamides derived from other secondary and primary amines can also be readily accessed, including piperidine (**2a–2f**), *tert*-butyl (3a–3f), benzyl (4a–4d), 2-arylethyl (5a–5e), 1-phenylethyl (**6a**, **6b**), and dibenzyl (7a–7c) amines.

Tertiary acids can be efficiently converted to corresponding sulfonamides, (*e.g.*, **1o**, **1p**, **2e**, **2f**) including sterically encumbered sulfonamides **3d** and **3e** that feature tertiary alkyl groups both on the S and N atoms.

**Scheme 2:**
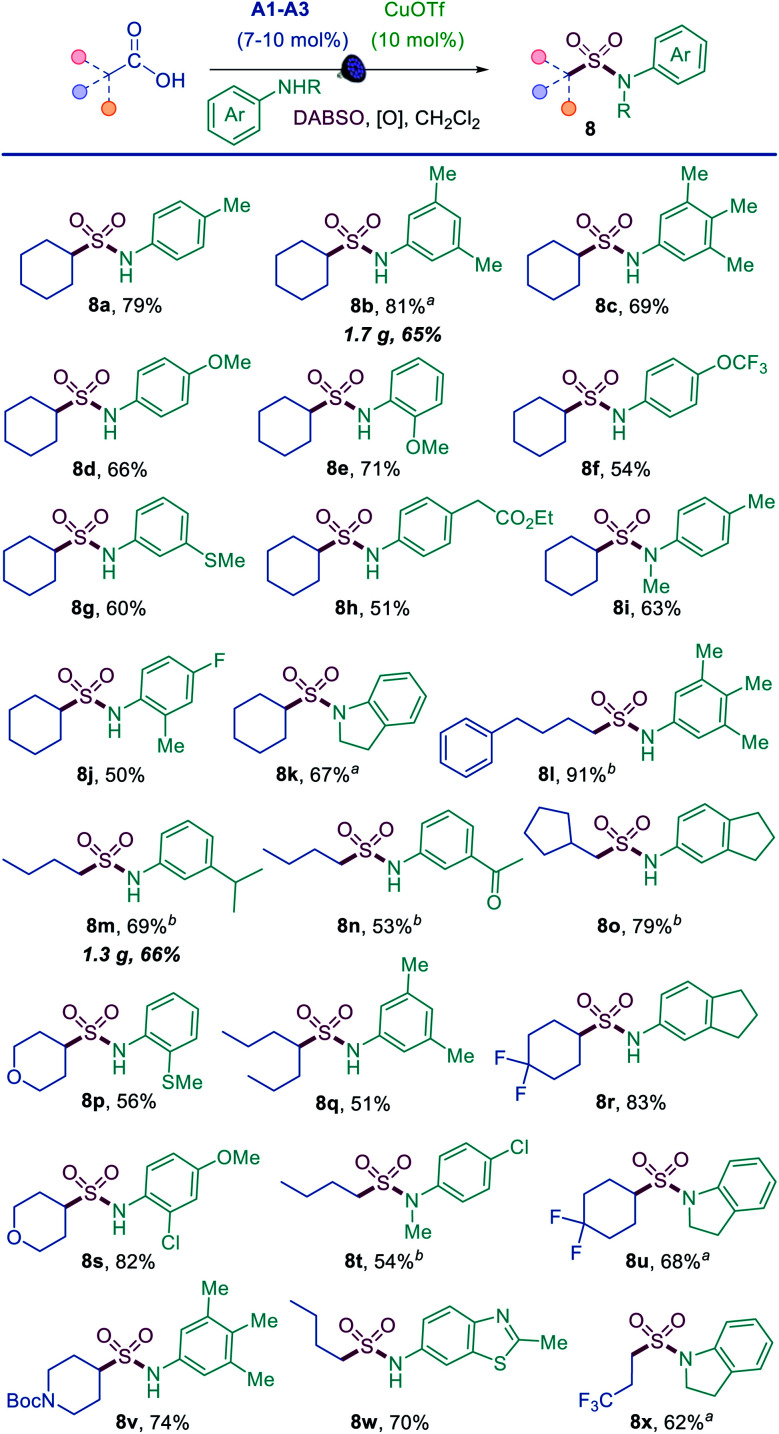
Scope of the direct decarboxylative amidosulfonation of anilines. Reaction conditions: aniline (0.3 mmol), carboxylic acid (0.6–0.75 mmol), CuOTf·½PhMe (10 mol%), **A2** (7 mol%), DABSO (0.45 mmol), ^*t*^BuO_2_Bz (0.36–0.45 mmol), CH_2_Cl_2_ (3 mL), LED light (400 nm). ^a^**A1** (7 mol%). ^b^**A3** (10 mol%), Cu(acac)_2_ (10 mol%).

The results demonstrate that the direct decarboxylative amidosulfonation with *O*-benzoylhydroxylamines allows for a facile construction of *N*-alkylsulfonamides. However, it would not be as practical for the synthesis of aromatic sulfonamides because the corresponding *N*-aryl-substituted *O*-benzoylhydroxylamines are not readily available. We therefore questioned if aromatic sulfonamides could instead be accessed by a direct decarboxylative amidosulfonation with anilines. Indeed, simple modification of the reaction conditions (*i.e.*, with copper(i) triflate as a catalyst and *tert*-butyl perbenzoate as an oxidant) resulted in an efficient amidosulfonation of anilines (Scheme 2). The scope of anilines was investigated with cyclohexanecarboxylic acid, and a range of substituted aromatic sulfonamides were readily accessed in one step (**8a–8k**). The reaction performs well with *para*-, *meta*-, and *ortho*-substituted anilines (**8a**, **8b**, **8e**, **8j**). Acyclic and cyclic *N*-substituted anilines were also suitable substrates (**8i**, **8k**). Other combinations of carboxylic acids and anilines were further tested (**8l–8y**), and comparable performance was observed in all cases with a diverse set of anilines bearing electron-withdrawing, electron-donating, and heterocyclic substituents (**8n**, **8r**, **8w**). These results indicate that the acridine/copper dual catalytic, decarboxylative amidosulfonation can be readily accomplished directly with carboxylic acids and anilines, providing a straightforward access to *N*-aryl sulfonamides.

**Scheme 3:**
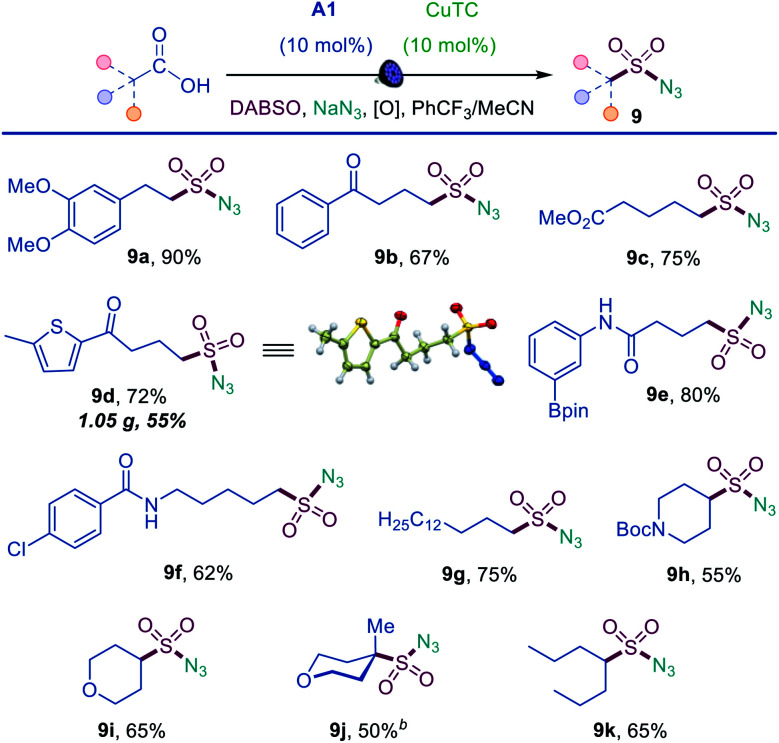
Scope of the direct decarboxylative azidosulfonation. Reaction conditions: carboxylic acid (0.3 mmol), copper 2-thiophenecarboxylate (CuTC) (10 mol%), **A1** (10 mol%), DABSO (0.45 mmol), ^*t*^BuO_2_Bz (0.75 mmol), NaN_3_ (0.9 mmol), PhCF_3_/MeCN (3 : 1, 3 mL), LED light (400 nm), 12 h.

**Fig. 3:**
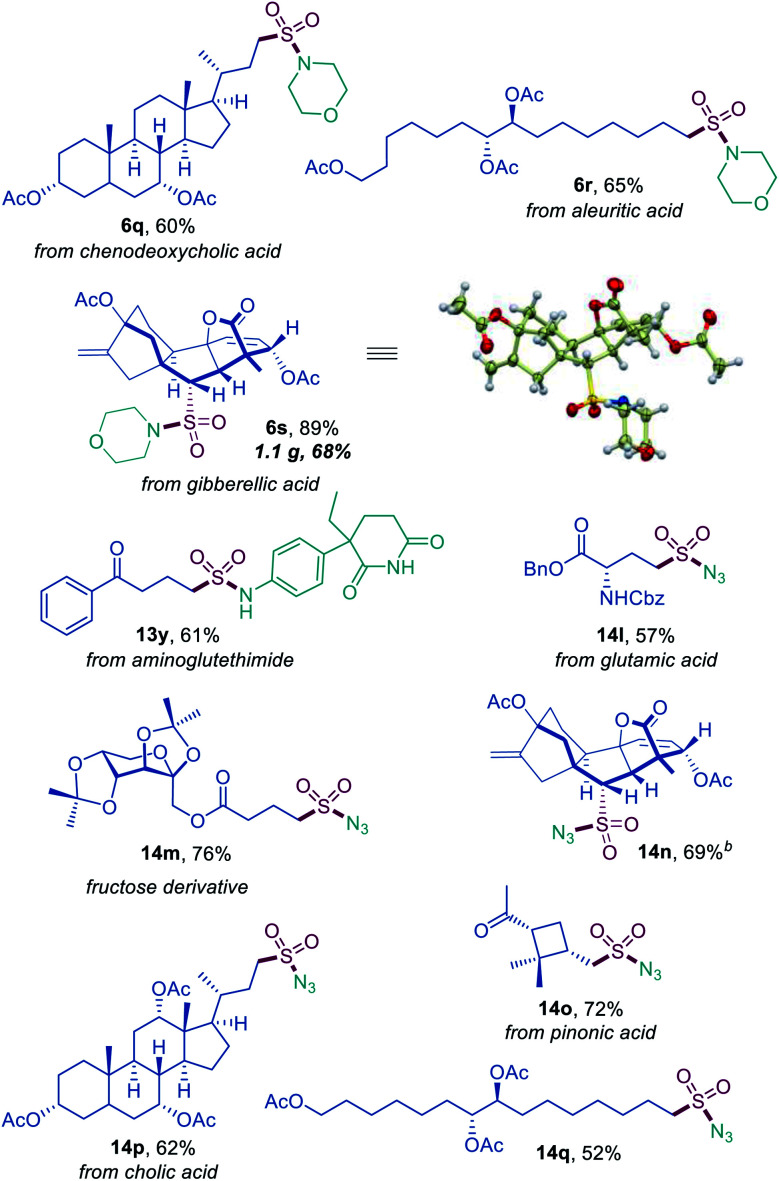
Direct decarboxylative amidosulfonation and azidosulfonation of natural products and active pharmaceutical ingredients.

Sulfonyl azides have emerged as versatile synthetic intermediates,^7^ yet their accessibility is limited and subject to the availability of their typical precursors – sulfonyl chlorides and sulfonamides.^13^ We, therefore, tested if the dual catalytic direct decarboxylative procedure could be extended to a coupling reaction with azides, resulting in a one-step conversion of carboxylic acids to sulfonyl azides. Gratifyingly, the direct decarboxylative azidosulfonation could indeed be readily accomplished with sodium azide as the nitrogen nucleophile with minor adjustments of the reaction conditions (Table S4† and Scheme 3). The method can be used to prepare an assortment of functionalized sulfonyl azides (**9a–9k**), bearing ketone (**9b**, **9d**), ester (**9c**), amide (**9e**, **9f**), boronate (**9e**), and other functional groups. Importantly, the facile adaptability of the DDAS reaction to an inorganic nucleophile demonstrates the synthetic potential of the method. Taken together, the developed dual catalytic methods provide the first examples of direct tricomponent conversion of carboxylic acids to sulfonamides and sulfonyl azides. The synthetic utility of the developed DDAS approach to the diverse classes of sulfonamides was further tested in several gram scale syntheses, and the corresponding products **1a**, **3a**, **8b**, **8m**, **9d** were readily obtained in good yields. The synthetic potential of the acridine-photocatalyzed direct decarboxylative amidosulfonation in the drug discovery context is also evident from the facility of the generation of libraries of sulfonamide bioisosteres and synthetic sulfa-analogues of the anticonvulsant drug valproic acid (**1h**, **2c**, **5b**, **8q**, **9k**, Schemes 1–3) and the lipid regulator gemfibrozil (**3f**, **5e**, Scheme 1). Sulfonamide and sulfonyl azide analogues of other drugs and natural products, *e.g.*, bile acids (**10a**, **10i**), aleuritic acid (**10b**, **10j**), plant growth regulator gibberellic acid (**10c**, **10g**), breast and prostate drug aminoglutethimide (**10d**), amino acid and carbohydrate derivatives (**10e**, **10f**), as well as pinonic acid (**10h**) are also readily produced (Fig. 3).

**Fig. 4:**
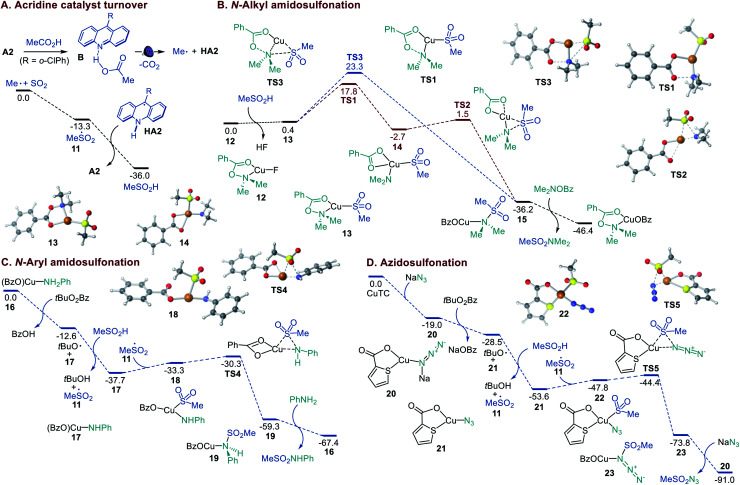
Computed energy profiles of the acridine catalyst turnover in the sulfinic acid formation and the Cu-catalyzed *N*-alkyl and *N*-aryl amidosulfonation and azidosulfonation, Δ*G*, kcal mol^−1^.

Previous experimental and computational studies of the acridine photocatalysis established that the photoinduced decarboxylation takes place after a proton-coupled electron transfer in the singlet excited acridine–carboxylic acid complex **B** (Fig. 4A). Acridinium carboxylates are not formed due to the low basicity of acridine (*e.g.*, p*K*_BH_^+^ = 12.7 in the ground state and 15.5 in the lowest singlet excited state for acridine *versus* p*K*_a_ = 21.6 for AcOH in MeCN).^9*a*^ Consistent with this conclusion, *N*-methylacridinium carboxylates do not undergo photoinduced decarboxylation, acridines do not catalyze photoinduced decarboxylation of carboxylate salts, and an addition of excess organic bases leads to suppression of the acridine-catalyzed decarboxylation.^9^ In agreement with the prior observations, no decarboxylative amidosulfonation reaction was observed with the *N*-methyl 9-mesitylacridinium catalyst (Table S1†), and the involvement of alkyl and alkylsulfonyl radicals was supported by radical trapping and EPR spectroscopic experiments (Figures S1 and S2†). The experimental studies show that acridines **A1–A3** are capable of catalyzing the direct amidosulfonation of carboxylic acids, while no reaction is observed with other common types of photocatalysts (Table S1†).

A DFT computational study was therefore carried out to clarify the mechanistic details that underpin the efficiency and facility of the acridine turnover and the dual catalytic processes (Fig. 4). The studies showed that alkylsulfonyl radical **11** that is formed in an exergonic reaction of the alkyl radical with SO_2_ can abstract a hydrogen atom from acridinyl radical **HA2** in a highly thermodynamically favorable process, enabling a facile turnover of the acridine catalyst. The Cu-catalyzed amidosulfonation with *O*-benzoylhydroxylamines was studied next. CuF_2_ can be readily reduced by acridinyl radical **HA2** (0.7 V *vs.* SCE and −0.6 V *vs.* SCE, respectively), producing Cu^I^ intermediate **12** upon complexation with the *O*-benzoylhydroxylamine reagent. The subsequent displacement of the fluoride with sulfinate in hydroxylamine-ligated complex **12** affords S-bound sulfinate complex **13** that can further undergo the exergonic and readily kinetically accessible (*via***TS1**) N–O bond cleavage-induced formation of Cu amido intermediate **14** (Fig. 4B). The subsequent S–N bond-forming step proceeds highly exergonically and over a small barrier (**TS2**) en route to sulfonamide-ligated species **15**. The ensuing ligand exchange with the hydroxylamine reagent liberates the sulfonamide product. An alternative pathway that involves concerted displacement of the benzoate by the sulfinate on the nitrogen atom in Cu^I^ complex **13** proceeds over a substantially higher barrier (*cf.*, **TS1***vs.***TS3**), pointing to the stepwise pathway as the major amidosulfonation route.

In the amidosulfonation with anilines (Fig. 4C), complex **16** can be readily formed in an exergonic reaction of copper(i) triflate with a carboxylic acid (*e.g.*, benzoic acid formed from the oxidant) and aniline (Figure S3†). Aniline complex **16** can undergo an exergonic proton-coupled electron transfer,^9*b*^ producing amido complex **17**, along with benzoic acid and the *tert*-butoxy radical that generates sulfonyl radical **11** in a reaction with the sulfinic acid. Subsequent reaction of amido complex **17** with sulfonyl radical **11** produces Cu sulfinate intermediate **18**. The ensuing S–N bond-forming coupling proceeds exergonically over a small barrier of 3 kcal mol^−1^ (**TS4**, 7.4 kcal mol^−1^ from **17**), affording Cu-ligated sulfonamide **19** that subsequently releases the sulfonamide product in a reaction with aniline.

Finally, we investigated the mechanism of the azidosulfonation reaction (Fig. 4D). Azide complex **20** that is readily formed from CuTC can reduce the oxidant, affording azide complex **21** and the *tert*-butoxy radical in an exergonic step. The latter engages the sulfinic acid in a HAT, producing sulfonyl radical **11**. The addition of sulfonyl radical **11** to azide complex **21** results in intermediate **22** that undergoes a facile S–N bond-forming reaction with an accessible overall barrier of 9.2 kcal mol^−1^ (**TS5**) from **21**.^14,15^ Collectively, the efficiency of the dual catalytic decarboxylative amidosulfonation and azidosulfonation reactions is enabled by the thermodynamically favorable acridine catalyst turnover in the reaction of the acridinyl radical with the sulfonyl radical and the kinetic and thermodynamic facility of the Cu-catalyzed S–N bond-forming processes.

## Conclusions

In conclusion, we have developed a visible light-induced photocatalytic amidosulfonation platform that enables the first direct conversion of a wide range of carboxylic acids to aliphatic and aromatic sulfonamides, as well as sulfonyl azides in an adaptive tricomponent process with electrophilic and nucleophilic N-centered coupling partners. The scope and functional group tolerance of the method were further demonstrated on representative functionalized substrates, as well as natural products and medicinally relevant compounds. The development of the dual catalytic process for concomitant formation of C–S and C–N bonds with electrophilic and nucleophilic nitrogen coupling partners points to a potential adaptability of the photocatalytic platform to a wide range of sulfonyl products.

## Author contributions

OVL conceived the project. VTN, VDN, and NTHV carried out the experiments and GCH performed the computational studies, in consultation with OVL. HDA performed the X-ray crystallography studies. OVL, wrote the manuscript, and VTN, VDN, NTHV, and GCH contributed to writing the manuscript.

## Conflicts of interest

There are no conflicts to declare.

## Supplementary Material

SC-012-D1SC01389K-s001

SC-012-D1SC01389K-s002

## References

[cit1] (a) The Chemistry of Sulphinic Acids, Esters and their Derivatives, ed. S. Patai, John Wiley & Sons, Ltd, New Jersey, 1990

[cit2] (b) Metabolism, Pharmacokinetics and Toxicity of Functional Groups, ed. D. A. Smith, Royal Society of Chemistry: London, United Kingdom, 2010, pp. 99–167, 210–274

[cit3] Friedman H. L., Influence of isosteric replacements upon biological activity, NAS-NRS, Washington, DC, 1951, vol. 206, pp. 295–358, NAS-NRS Publication No. 206

[cit4] Nguyen B., Emmett E. J., Willis M. C. (2010). J. Am. Chem. Soc..

[cit5] (a) Bioactive Carboxylic Compound Classes: Pharmaceuticals and Agrochemicals, ed. C. Lamberth and J. Dinges, Wiley, 2016

[cit6] Langdon S. R., Ertl P., Brown N. (2010). Mol. Inf..

[cit7] Hein J. E., Fokin V. V. (2010). Chem. Soc. Rev..

[cit8] McMurray L., McGuire T. M., Howells R. L. (2020). Synthesis.

[cit9] Nguyen V. D., Nguyen V. T., Haug G. C., Dang H. T., Jin S., Li Z., Flores-Hansen C., Benavides B., Arman H. D., Larionov O. V. (2019). ACS Catal..

[cit10] Li Y., Chen S., Wang M., Jiang X. (2020). Angew. Chem., Int. Ed..

[cit11] He J., Chen G., Zhang B., Li Y., Chen J. R., Xiao W. J., Liu F., Li C. (2020). Chem.

[cit12] Woolven H., González-Rodríguez C., Marco I., Thompson A. L., Willis M. C. (2011). Org. Lett..

[cit13] El-Sayed R. A. (2004). Phosphorus, Sulfur Silicon Relat. Elem..

[cit14] In addition to the described pathways that proceed *via* S-bound Cu sulfinate complexes, the pathways involving O-bound Cu sulfinate intermediates were also studied, and a similar mechanistic behavior was observed (Fig. S4–S6†). The *O*-benzoylhydroxylamine-mediated amidosulfonation proceeds over a 1.9 kcal/mol lower barrier with the O-bound sulfinate intermediates than with the S-bound Cu sulfinate intermediates, retaining the kinetic preference of the stepwise mechanism. In contrast, the pathways proceeding *via* sulfur-bound Cu sulfinate intermediates are substantially more favored for the aniline amidosulfonation and the azidosulfonation reactions.

[cit15] Alternative pathways for the amidosulfonation with N-nucleophiles that proceed *via* an addition of a N-nucleophile to the sulfonyl radical were explored with aniline as a typical nucleophile (Fig. S7†). The reactions of both aniline and the deprotonated aniline with sulfonyl radical **11**, were highly endergonic, ruling out their involvement in the amidosulfonation.

