# Supplementary material for: Photocatalytic decarboxylative amidosulfonation enables direct transformation of carboxylic acids to sulfonamides
Source: Chem Sci. 2021 Apr 13;12(18):6429–36. doi: 10.1039/d1sc01389k (PMC8115300; doi:10.1039/d1sc01389k)
Supplement: SC-012-D1SC01389K-s001 [file SC-012-D1SC01389K-s001.pdf]

Electronic Supplementary Material (ESI) for Chemical Science.
This journal is © The Royal Society of Chemistry 2021
Evaluation Only. Created with Aspose.Pdf. Copyright 2002-2014 Aspose Pty Ltd.

Supporting Information

Photocatalytic Decarboxylative Amidosulfonation Enables Direct Transformation of Carboxylic Acids to Sulfonamides

Vu T. Nguyen,[+] Graham C. Haug,[+] Viet D. Nguyen, Ngan H. T. Vuong, Hadi D. Arman, and Oleg V. Larionov*

Department of Chemistry, The University of Texas at San Antonio, San Antonio, Texas 78249, United States

oleg.larionov@utsa.edu

Contents

Materials and experimental details S2
General procedures S3
Additional experiemental and computational studies S5
Figure S1 .... S8
Figures S2-S3 .... S9
Figures S4-S7 .... S9
Acridine synthesis S13
Sulfonamide products S14
Sulfonyl azides S80
Computational data S102
X-ray crystallographic data S172
NMR spectroscopic data S190
References S368

Materials and experimental details

Materials: Acridines **A1**, **A2**,[1] as well as DABSO,[2] compounds 5-oxo-5-((3-(4,4,5,5-tetramethyl-1,3,2-dioxaborolan-2-yl)phenyl)amino)pentanoic acid (**S8**),[3] 5-(5-

[+] V.T.N. and G.C.H. contributed equally

Evaluation Only. Created with Aspose.Pdf. Copyright 2002-2014 Aspose Pty Ltd.

methylthiophen-2-yl)-5-oxopentanoic acid (**S9**),[4] 9,10,16-triacetoxyhexadecanoic acid (**S10**),[5] 3,13-di-*O*-acetylgibberellic acid (**S11**),[6] 3α,7α-diacetoxy-5β-cholanic acid (**S12**),[7] 5-oxo-5-(((3a*S*,5a*R*,8a*R*,8b*S*)-2,2,7,7-tetramethyltetrahydro-3a*H*-bis([1,3]dioxolo)[4,5-b:4',5'-d]pyran-3a-yl)methoxy)pentanoic acid (**S13**),[8] and cholic acid 3,7,12-triacetate (**S14**)[7] were prepared as previously described. All other chemicals were used as commercially available.

Experimental equipment: Reactions were set up by purging vigorously stirred reaction mixtures with argon for 3 min prior to irradiation, or in a glovebox. Borosilicate glass test-tubes (9- and 10-mL capacity) fitted with GL14 and GL18 screw-caps were used, and the sealed reaction test-tubes were placed in a test-tube rack on a magnetic stirplate that was flanked by two 400 nm 36W LED lights. The temperature in the test-tube rack was 35 °C. Eight parallel reactions arranged in two rows of four tubes were typically carried out in one test-tube rack.

Glovebox work was carried out in a nitrogen-filled LC Technology Solutions LCPW-220 glovebox.

Purification: Column chromatography was performed using CombiFlash Rf-200 (Teledyne-Isco) automated flash chromatography system, as well as manually. Thin layer chromatography was carried out on silica gel-coated glass plates (Merck Kieselgel 60 F254). Plates were visualized under ultraviolet light (254 nm) and using a potassium permanganate stain.

Characterization: ^{1}H, ^{13}C, ^{11}B, and ^{19}F NMR spectra were recorded at 500 MHz (^{1}H), 125 MHz (^{13}C), 202 MHz (^{31}P), 470 MHz (^{19}F), and 160 MHz (^{11}B) on Bruker AVANCE III 500 instruments in $CDCl_3$ or other specified deuterated solvents with and without tetramethylsilane (TMS) as an internal standard at 25 °C, unless specified otherwise. Chemical shifts (δ) are reported in parts per million (ppm) from tetramethylsilane (^{1}H and ^{13}C), $BF_3{\cdot}OEt_2$ (^{11}B), and $CFCl_3$ (^{19}F). Coupling constants (*J*) are in Hz. Proton

Evaluation Only. Created with Aspose.Pdf. Copyright 2002-2014 Aspose Pty Ltd.

multiplicity is assigned using the following abbreviations: singlet (s), doublet (d), triplet (t), quartet (q), quintet (quint.), septet (sept.), multiplet (m), broad (br).
Infrared measurements were carried out neat on a Bruker Vector 22 FT-IR spectrometer fitted with a Specac diamond attenuated total reflectance (ATR) module. EPR Spectra were collected on a Bruker EMX X-band EPR spectrometer.

General Procedures

General procedure for the visible light-induced, dual catalytic decarboxylative amidosulfonation with *O*-benzoylhydroxylamines (GP1)

To a 10 mL test-tube, carboxylic acid (0.3 mmol), DABSO (79 mg, 0.33 mmol, 1.1 equiv.), *O*-benzoylhydroxylamine (0.6 mmol, 2 equiv.), acridine catalyst **A1** (9 mg, 0.03 mmol, 10 mol%), copper difluoride (3 mg, 0.03 mmol, 10 mol%), and degassed dichloromethane (6 mL) were added. The test-tube was capped and the reaction mixture was irradiated with LED light (λ = 400 nm) while stirring for 12 h. The reaction mixture was then quenched with saturated solution of sodium hydrogen carbonate (3 mL) and extracted with ethyl acetate (3 × 10 mL). The organic layer was collected, dried over anhydrous sodium sulfate, concentrated under reduced pressure, and the remaining material was purified by flash chromatography on silica gel to give the sulfonamide product.

General procedure for the visible light-induced, dual catalytic decarboxylative amidosulfonation with anilines (GP2)

To a 10 mL test-tube, aniline (0.3 mmol), carboxylic acid (0.75 mmol, 2.5 equiv.), DABSO (108 mg, 0.45 mmol, 1.5 equiv.), acridine catalyst (0.02–0.03 mmol, 7–10 mol%), *tert*-butyl peroxybenzoate (87 mg, 0.45 mmol, 1.5 equiv.), copper salt (0.03 mmol, 10 mol%), and degassed dichloromethane (3 mL) were added. The test-tube was capped and the reaction mixture was irradiated with LED light (λ = 400 nm) while stirring for 12 h. The reaction

Evaluation Only. Created with Aspose.Pdf. Copyright 2002-2014 Aspose Pty Ltd.

mixture was then quenched with saturated solution of sodium hydrogen carbonate (3 mL), extracted with ethyl acetate (3 × 10 mL). The organic layer was collected, dried over anhydrous sodium sulfate, concentrated under reduced pressure, and the remaining material was purified by flash chromatography on silica gel to give the sulfonamide product.

General procedure for the visible light-induced, dual catalytic decarboxylative azinosulfonation (GP3)

To a 10 mL test-tube, carboxylic acid (0.3 mmol), DABSO (108 mg, 0.45 mmol, 1.5 equiv.), acridine catalyst **A1** (9 mg, 0.03 mmol, 10 mol%), sodium or potassium azide (0.9 mmol, 3.0 equiv.), *tert*-butyl peroxybenzoate (145 mg, 0.75 mmol, 2.5 equiv.), copper salt (0.03 mmol, 10 mol%) and degassed $PhCF_3$/MeCN (3mL, 3 : 1 v/v) were added. The test-tube was capped and the reaction mixture was irradiated with LED light (λ = 400 nm) while stirring at room temperature for 12 h. The reaction mixture was treated with saturated solution of sodium carbonate (3 mL) and extracted with DCM (3 × 10 mL). The organic layer was collected, dried over anhydrous sodium sulfate, concentrated under reduced pressure, and the remaining material was purified by flash chromatography on silica gel to give the sulfonyl azide product.

Evaluation Only. Created with Aspose.Pdf. Copyright 2002-2014 Aspose Pty Ltd.

Additional experiemental and computational studies

Table S1. Catalyst Performance in the Photocatalytic Direct Decarboxylative *N*-Alkyl Aminosulfonation. [a]

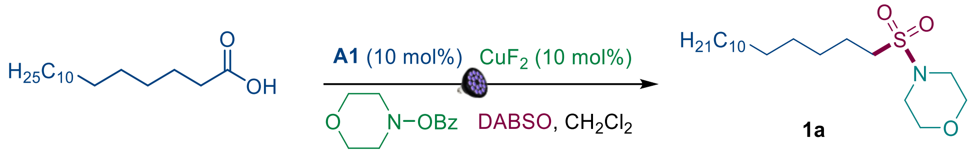

Entry	Photocatalyst	Yield, %
1	Eosin Y at 450 nm	0
2	Eosin Y at 420 nm	0
3	Eosin Y at 400 nm	0
4	Eosin Y disodium salt at 450 nm	0
5	4CzIPN at 450 nm	0
6	4CzIPN at 420 nm	0
7	4CzIPN at 400 nm	0
8	$[Acr\text{-}Mes]^+(ClO_4)^-$ at 400 nm	0
9	$[Acr\text{-}Mes]^+(ClO_4)^-$ at 450 nm	0[b]
10	$Ir(ppy)_3$ at 450 nm	0[b]
11	$Ir(ppy)_2(pq)$ at 450 nm	0[b]
12	$(Ir[dF(CF_3)ppy]_2(dtbpy))PF_6$ at 450 nm	0[b]
13	$Ru(bpm)_2Cl_2$ at 450 nm	0[b]
14	Ru(*p*-CF_3-bpy)$_3$(BF_4)$_2$ at 450 nm	0[b]
15	TiO_2, anatase	0[c]

[a] Reaction conditions: carboxylic acid (0.3 mmol), DABSO (0.33 mmol), DABSO (0.33 mmol), acridine **A1**(10 mol%), CuF_2 (10 mol%) *O*-benzoylhydroxylamine (0.6 mmol), CH_2Cl cx$_2$ (6 mL), LED light (400 nm), 12 h. Yield was determined by ^1H NMR spectroscopy with 1,4-dimethoxybenzene as an internal standard. [b] 2 mol% photocatalyst was used. [c] nanopowder, <25 nm particle size, 30 mg. 4CzIPN: 1,2,3,5-Tetrakis(carbazol-9-yl)-4,6-dicyanobenzene, $[Acr\text{-}Mes]^+(ClO_4)^-$: 10-Methyl-9-(2,4,6-trimethylphenyl)acridinium perchlorate, $Ir(ppy)_3$: Tris(2-phenylpyridine)iridium(III), $Ir(ppy)_2(pq)$: bis(2-phenylpyridine)(2-phenyl-quinoline)iridium(III), $(Ir[dF(CF_3)ppy]_2(dtbpy))PF_6$: [4,4′-Bis(1,1-dimethylethyl)-2,2′-bipyridine-*N1,N1′*]bis[3,5-difluoro-2-[5-(trifluoromethyl)-2-pyridinyl-*N*]phenyl-C]Iridium(III) hexafluoro-phosphate, $Ru(bpm)_2Cl_2$: Tris(2,2′-bipyrimide)ruthenium(II) dichloride, Ru(*p*-CF_3-bpy)$_3$(BF_4)$_2$: Tris(2,2′-(*p*CF_3)bipyridine)-ruthenium(II) tetrafluoroborate.

Evaluation Only. Created with Aspose.Pdf. Copyright 2002-2014 Aspose Pty Ltd.

Table S2. Reaction Conditions for the Photocatalytic Direct Decarboxylative *N*-Alkyl Aminosulfonation [a]

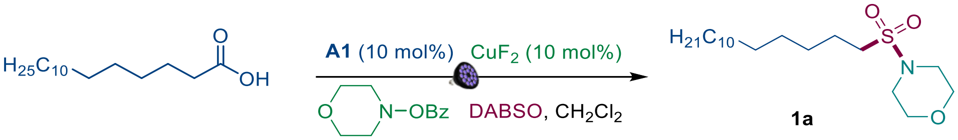

Entry	Variations from standard conditions	Yield, %
1	none	92 (89[b])
2	$Cu(OAc)_2$ instead of CuF_2	88
3	CuOAc instead of CuF_2	87
4	CuCl instead of CuF_2	82
5	MeCN instead of CH_2Cl_2	46
6	$PhCF_3$ instead of CH_2Cl_2	64
7	EtOAc instead of CH_2Cl_2	58

[a] Reaction conditions: carboxylic acid (0.3 mmol), DABSO (0.33 mmol), DABSO (0.33 mmol), acridine **A1**(10 mol%), CuF_2 (10 mol%) *O*-benzoylhydroxylamine (0.6 mmol), CH_2Cl cx$_2$ (6 mL), LED light (400 nm), 12 h. Yield was determined by ^{1}H NMR spectroscopy with 1,4-dimethoxybenzene as an internal standard. [b] Isolated yield.

Table S3. Reaction Conditions for the Photocatalytic Direct Decarboxylative Aminosulfonation with Anilines [a]

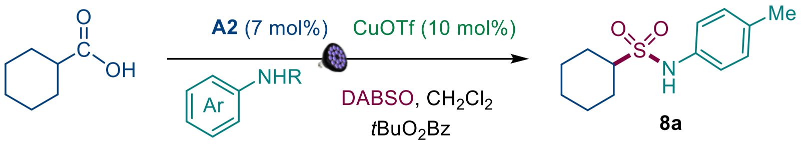

Entry	Variations from standard conditions	Yield, %
1	none	81(79[b])
2	$CuOTf_2$·½PhH instead of CuOTf·½$PhCH_3$	76
3	$Cu(acac)_2$ instead of CuOTf·½$PhCH_3$	51
4	$PhCF_3$ instead of CH_2Cl_2	48
5	EtOAc instead of CH_2Cl_2	25
6	DTBP instead of *t*BuO_2Bz	0
7	TBHP (70% w/w in water) instead of *t*BuO_2Bz	0
8	Bz_2O_2 instead of *t*BuO_2Bz	18
9	DCP instead of *t*BuO_2Bz	0

[a] Reaction conditions: aniline (0.3 mmol), carboxylic acid (0.75 mmol), DABSO (0.45 mmol), acridine **A2** (7 mol%), CuOTf·½$PhCH_3$ (10 mol%), *t*BuO_2Bz (0.45 mmol), CH_2Cl_2 (3 mL), LED light

Go back to table of contents

Evaluation Only. Created with Aspose.Pdf. Copyright 2002-2014 Aspose Pty Ltd.

(400 nm), 12 h. Yield was determined by ^{1}H NMR spectroscopy with 1,4-dimethoxybenzene as an internal standard. [b] Isolated yield. DTBP = Di-*tert*-butyl peroxide, TBHP = *tert*-Butyl hydroperoxide, DCP = dicumyl peroxide.

Table S4. Reaction Conditions for the Photocatalytic Direct Decarboxylative Azinosulfonation[a]

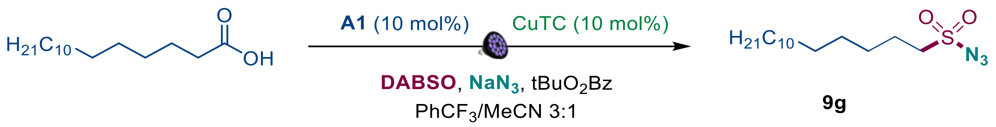

Entry	Variations from standard conditions	Yield, %
1	none	78 (75[b])
2	Cu(hfac)$_2$ instead of CuTC	52
3	CuOAc instead of CuTC	70
4	DTBP instead of *t*BuO$_2$Bz	0
5	$Na_2S_2O_8$ instead of *t*BuO$_2$Bz	40
6	TMSN$_3$ instead of NaN$_3$	44
7	PhCF$_3$ instead of PhCF$_3$/MeCN	54
7	EtOAc instead of PhCF$_3$/MeCN	5

[a] Reaction conditions: carboxylic acid (0.3 mmol), CuTC (10 mol%), DABSO (0.45 mmol), acridine **A1** (10 mol%), NaN$_3$ (0.9 mmol), *t*BuO$_2$Bz (0.75 mmol), PhCF$_3$/MeCN (3:1 v/v 3 mL), LED light (400 nm), 12 h. Yield was determined by ^{1}H NMR spectroscopy with 1,4-dimethoxybenzene as an internal standard. [b] Isolated yield. CuTC = copper 2-thiophenecarboxylate; hfac = hexafluoroacetylacetonate. DTBP = di-*tert*-butyl peroxide.

Cyclic voltammetry studies

Cyclic voltammetry (CV) measurements were performed on a CHI 650D potentiostat using a three-electrode cell with a glassy-carbon working electrode, a Ag|AgCl (1M KCl) reference electrode and a platinum counter electrode. CV was conducted at a scan rate of 100 mV s^{-1} for tetrabutalammonium methanesulfinate and tetrabutalammonium methanesulfinate (0.4 mM) and 50 mV s^{-1} for copper difluoride (0.2 mM) in anhydrous degassed acetonitrile with tetrabutylammonium hexafluorophosphate (0.2M) as an electrolyte. Inflection-point potentials (E_{red}) were used to characterize irreversible redox

Evaluation Only. Created with Aspose.Pdf. Copyright 2002-2014 Aspose Pty Ltd.

processes, since they were shown to provide the best approximation of standard electrochemical potentials for irreversible redox systems.[9] The measured oxidation potentials vs SCE were 0.30 V for tetrabutylammonium methanesulfinate and 1.28 V for tetrabutylammonium acetate. The Cu^{II}/Cu^{I} reduction potential of copper difluoride was 0.70 V vs SCE.

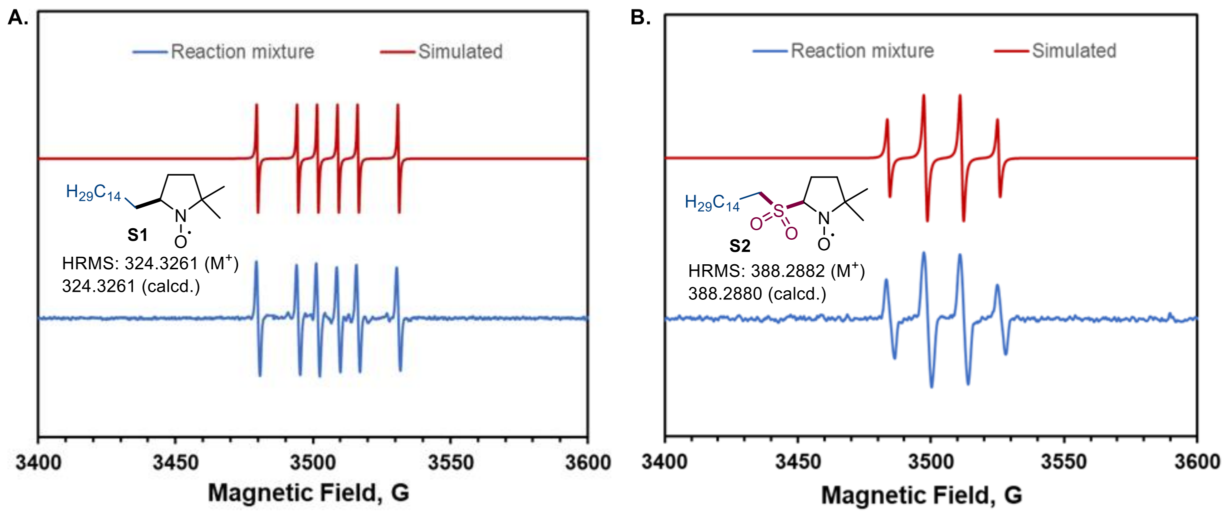

Figure S1. Room temperature X-band EPR spectroscopic spin-trapping studies of the direct decarboxylative reaction with palmitic acid as a substrate and DMPO as a spin trap. **A.** Experimental and simulated EPR spectra as well as high resolution mass spectrometry data of the alkyl-DMPO spin adduct **S1** (g = 2.0058, a_N = 14.7 G, a_H = 22.0 G) observed in the reaction with DABSO as a sulfur dioxide source. **B.** Experimental and simulated EPR spectra as well as high resolution mass spectrometry data of the alkylsulfonyl-DMPO spin adduct **S2** (g = 2.0059, a_N = 13.6 G, a_H = 14.2 G) observed in the reaction with sulfur dioxide. The use of DABSO allowed for successful spin trapping of the alkyl spin adduct due to the lower solubility of DABSO and lower concentration of sulfur dioxide, while the use of sulfur dioxide allowed for the detection of the alkylsulfonyl spin adduct.

Evaluation Only. Created with Aspose.Pdf. Copyright 2002-2014 Aspose Pty Ltd.

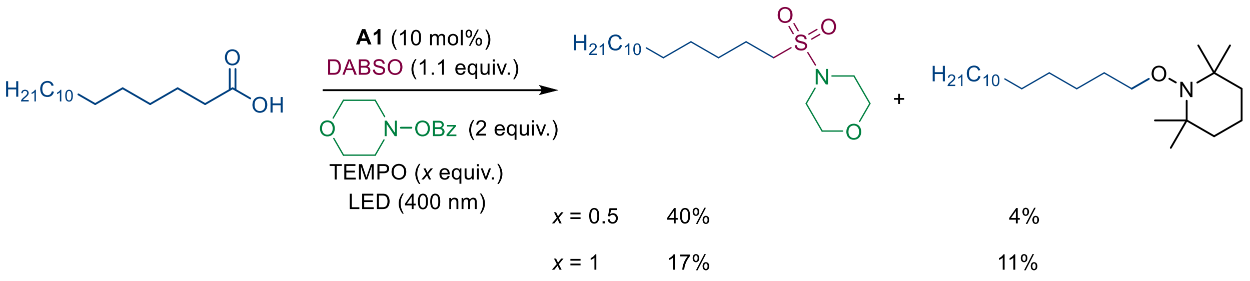

Figure S2. Alkyl radical trapping studies with TEMPO.

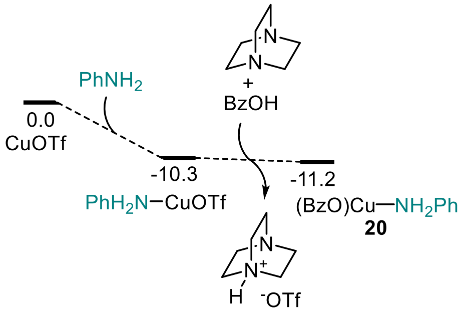

Figure S3. A computed energy profile for the formation of copper-ligated aniline intermediate **20**, Δ*G*, kcal/mol. Benzoate was selected as a spectator ligand because it is formed as a by-product of the reduction of *t*BuO_2Bz and a similar reactivity is expected with aliphatic carboxylates.

Investigation of the reaction pathways proceeding via O-bound Cu sulfinate intermediates

In addition to the described pathways that proceed via S-bound Cu sulfinate complexes, the energy profiles of the pathways with the O-bound Cu sulfinate intermediates were studied (Figures S4-S6). The *O*-benzoylhydroxylamine-mediated aminosulfonation proceeds over a 1.9 kcal/mol lower barrier with the O-bound sulfinate intermediates than with the S-bound Cu sulfinate intermediates, retaining the kinetic preference of the stepwise mechanism. In contrast, the pathways proceeding via S-bound Cu sulfinate intermediates are substantially more favored for the aniline aminosulfonation and the azinosulfonation reactions.

Evaluation Only. Created with Aspose.Pdf. Copyright 2002-2014 Aspose Pty Ltd.

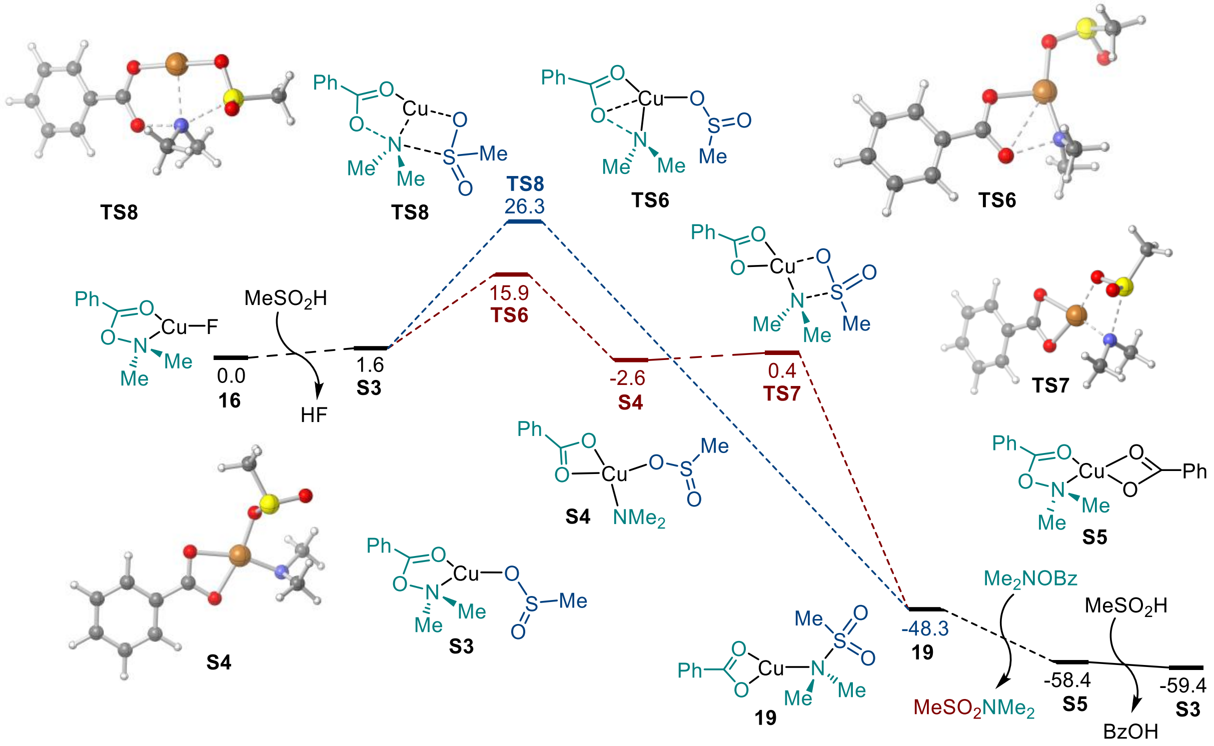

Figure S4. A computed energy profile for the radical copper-catalyzed *N*-alkyl aminosulfonation proceeding via oxygen-bound copper sulfinate intermediates **S3** and **S4**, ΔG, kcal/mol.

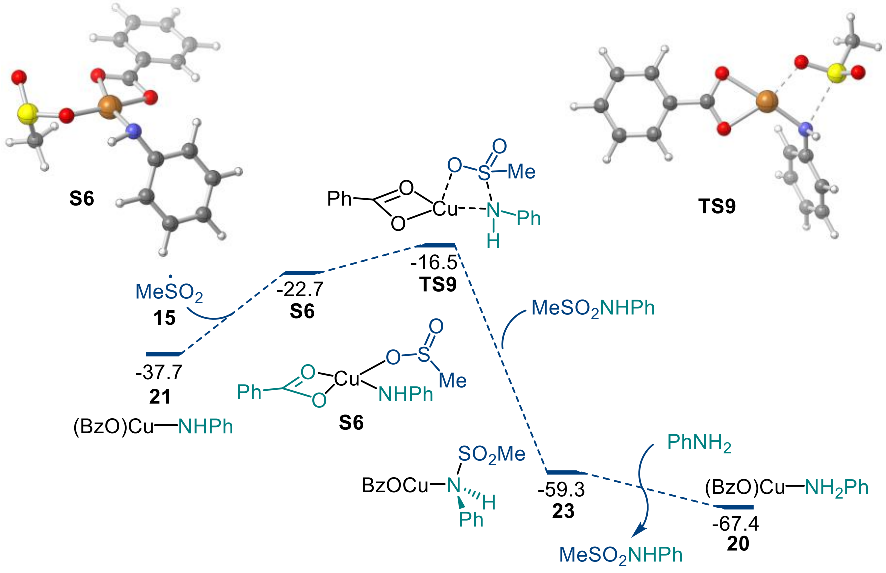

Figure S5. A computed energy profile for the radical copper-catalyzed *N*-aryl aminosulfonation proceeding via oxygen-bound copper sulfinate intermediate **S6**, ΔG, kcal/mol.

Go back to table of contents

Evaluation Only. Created with Aspose.Pdf. Copyright 2002-2014 Aspose Pty Ltd.

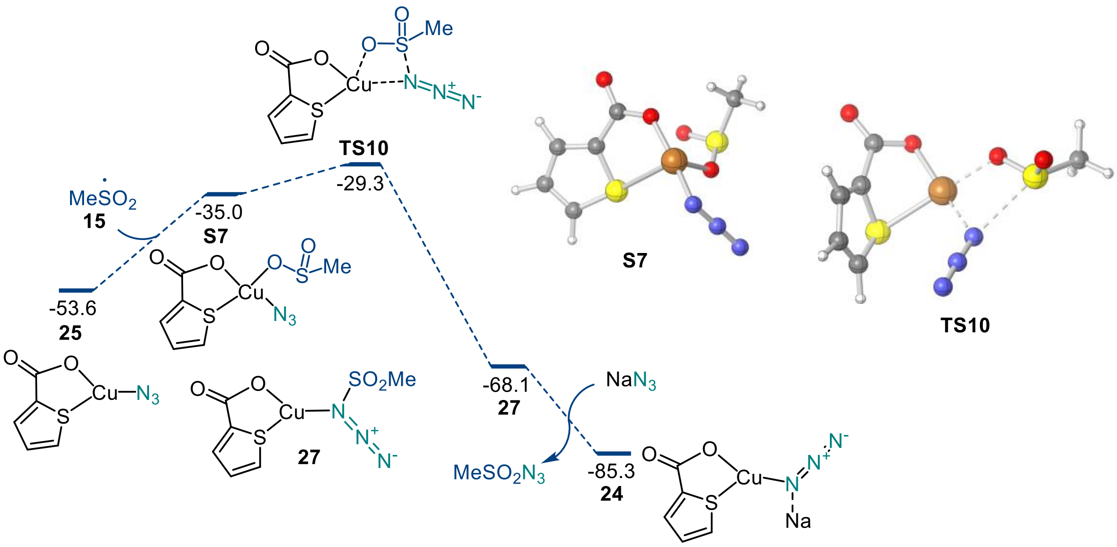

Figure S6. A computed energy profile for the radical copper-catalyzed azinosulfonation proceeding via oxygen-bound copper sulfinate intermediate **S7**, Δ*G*, kcal/mol.

Investigation of alternative mechanistic pathways for the amidosulfonation with N-nucleophiles

An alternative mechanism that involves the initial N-nucleophile addition to sulfonyl radical **11** was also investigated computationally with aniline as a typical nucleophile (Figure S7). The addition of aniline to sulfonyl radical **11** was found to be substantially exergonic by 8.6 kcal/mol. We also studied the pathway that involves the addition of the deprotonated aniline to sulfonyl radical **11**. Because of the absence of a strong base under the reaction conditions, the deprotonation was modeled with aniline, and was found to be prohibitively endergonic ($\Delta G = 55.6$ kcal/mol). Furthermore, the addition of the aniline anion to sulfonyl radical **11** was also significantly thermodynamically unfavorable by 14.3 kcal/mol. Given that the pathway involves subsequent biomolecular oxidation of the transient addition intermediate, in contrast to the low-barrier unimolecular reductive elimination steps in the pathway presented in Figure 4, and the high endergonicity of the addition steps, the calculations suggest that the pathway involving the N-nucleophile addition to sulfonyl radicals is not operative under the present reaction conditions.

Evaluation Only. Created with Aspose.Pdf. Copyright 2002-2014 Aspose Pty Ltd.

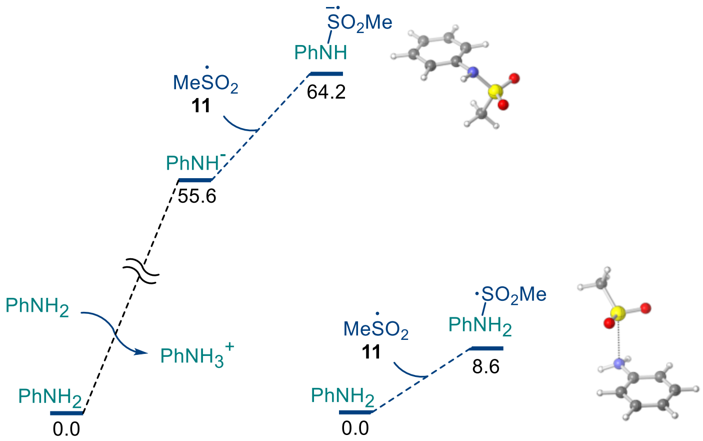

Figure S7. Computed energy profiles for the addition of aniline and the deprotonated aniline to sulfonyl radical **11**, Δ*G*, kcal/mol.

Evaluation Only. Created with Aspose.Pdf. Copyright 2002-2014 Aspose Pty Ltd.

Acridine synthesis

2,7-Dimethyl-9-(*o*-tolyl)acridine (A3)

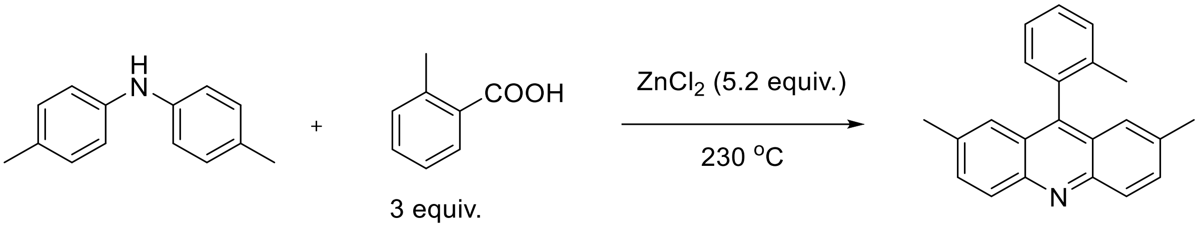

A3

According to the known procedure for synthesis of **A1**,[1] the reaction was carried out with di-*p*-tolylamine (1.42 g, 7.2 mmol), 2-methylbenzoic acid (2.94 g, 21.6 mmol, 3 equiv.), zinc chloride (5.10 g, 37.4 mmol, 5.2 equiv.) in a sand bath at 230 °C for 14 h. After completion, the reaction was quenched with a saturated solution of ammonium hydroxide (50 mL) and then extracted with ethyl acetate (3 x 75 mL). The organic layer was washed with brine, separated, and dried over Na_2SO_4. Removal of the solvent and purification by silica gel chromatography (hexane/ ethyl acetate 9 : 1 v/v) afforded acridine **A3** as a yellow solid (1.39 g, 65%).

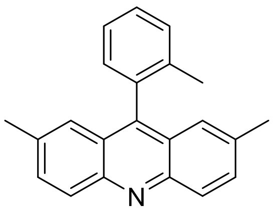

m.p.: 158–160 °C. – ^{1}H NMR (500 MHz, $CDCl_3$): 8.16 (2 H, d, *J* = 8.8 Hz), 7.57 (2 H, dd, *J* = 8.9, 1.9 Hz), 7.53–7.43 (2 H, m), 7.41 (1 H, td, *J* = 7.3, 1.5 Hz), 7.24–7.19 (3 H, m), 2.43 (6 H, s), 1.88 (3 H, s) ppm. – ^{13}C NMR (125 MHz, $CDCl_3$): 147.4, 144.7, 137.1, 136.1, 135.6, 132.6, 130.4, 130.3, 129.5, 128.5, 126.0, 125.4, 124.5, 22.1, 19.9 ppm. – IR: 3025, 2956, 1610, 1560, 1523, 1436, 1370, 1008, 856 cm^{-1}. – HRMS: calcd for $C_{22}H_{20}N$: 298.1590, found 298.1590 [$M+H^+$].

Go back to table of contents

Evaluation Only. Created with Aspose.Pdf. Copyright 2002-2014 Aspose Pty Ltd.

Sulfonamides

4-(Pentadecylsulfonyl)morpholine (1a)

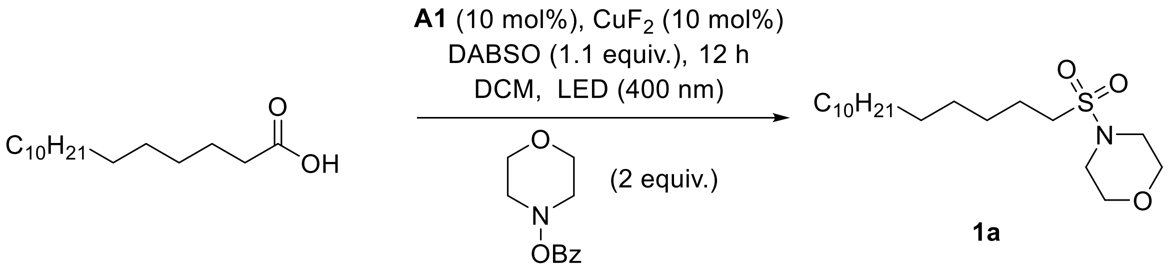

According to **GP1**, the reaction was carried out with palmitic acid (77 mg, 0.3 mmol), DABSO (79 mg, 0.33 mmol, 1.1 equiv.), *N*-(benzoyloxy)morpholine (124 mg, 0.6 mmol, 2 equiv.), acridine catalyst **A1** (9 mg, 0.03 mmol, 10 mol%), copper difluoride (3 mg, 0.03 mmol, 10 mol%), and degassed dichloromethane (6 mL). The test-tube was capped and the reaction mixture was irradiated with LED light (λ = 400 nm) while stirring for 12 h. The reaction mixture was then quenched with saturated solution of sodium hydrogen carbonate (3 mL) and extracted with ethyl acetate (3 × 10 mL). The organic layer was collected, dried over anhydrous sodium sulfate, concentrated under reduced pressure, and the remaining material was purified by flash chromatography on silica gel (hexane/ ethyl acetate 9 : 1 v/v) to give the sulfone product **1a** (96 mg, 89%) as a colorless solid.

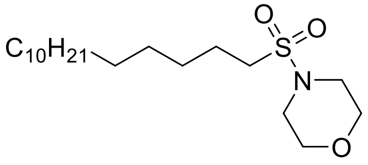

m.p.: 40–42°C. – ^{1}H NMR (500 MHz, $CDCl_3$): 3.79–3.66 (4 H, m), 3.33–3.19 (4 H, m), 2.95–2.82 (2 H, m), 1.88–1.69 (2 H, m), 1.41 (2 H, p, *J* = 7.2 Hz), 1.25 (22 H, s), 0.87 (3 H, t, *J* = 6.9 Hz) ppm. – ^{13}C NMR (125 MHz, $CDCl_3$): 66.8, 49.0, 46.0, 32.0, 29.8, 29.8, 29.7, 29.6, 29.5, 29.4, 29.4, 29.2, 28.6, 23.1, 22.8, 14.2 ppm. – IR: 3053, 2985, 2310, 1416, 1264, 895 cm^{-1}. – HRMS: calcd for $C_{19}H_{40}NO_3S$: 362.2729, found 362.2731 [M+H^{+}].

Evaluation Only. Created with Aspose.Pdf. Copyright 2002-2014 Aspose Pty Ltd.

4-(Morpholinosulfonyl)-1-phenylbutan-1-one (1b)

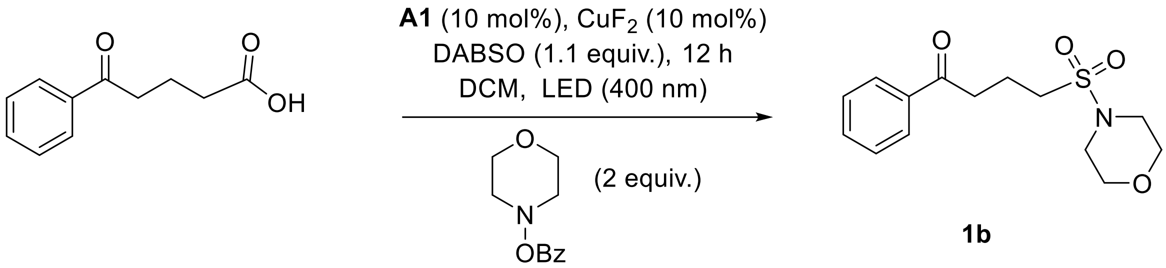

1b

According to **GP1**, the reaction was carried out with 5-oxo-5-phenylpentanoic acid (58 mg, 0.3 mmol), DABSO (79 mg, 0.33 mmol, 1.1 equiv.), *N*-(benzoyloxy)morpholine (124 mg, 0.6 mmol, 2 equiv.), acridine catalyst **A1** (9 mg, 0.03 mmol, 10 mol%), copper difluoride (3 mg, 0.03 mmol, 10 mol%), and degassed dichloromethane (6 mL). The test-tube was capped and the reaction mixture was irradiated with LED light (λ = 400 nm) while stirring for 12 h. The reaction mixture was then quenched with saturated solution of sodium hydrogen carbonate (3 mL) and extracted with ethyl acetate (3 × 10 mL). The organic layer was collected, dried over anhydrous sodium sulfate, concentrated under reduced pressure, and the remaining material was purified by flash chromatography on silica gel (hexane/ ethyl acetate 4 : 1 v/v) to give the sulfone product **1b** (63 mg, 71%) as a colorless solid.

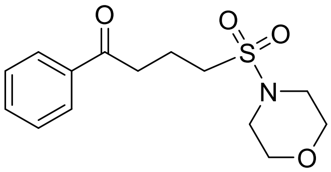

m.p.: 51–53 °C. – ^{1}H NMR (300 MHz, $CDCl_3$): 7.97–7.89 (2 H, m), 7.60–7.52 (1 H, m), 7.49–7.36 (2 H, m), 3.76–3.66 (4 H, m), 3.30–3.14 (6 H, m), 3.11–2.98 (2 H, m), 2.25 (2 H, dt, *J* = 13.7, 6.7 Hz) ppm. – ^{13}C NMR (75 MHz, $CDCl_3$): 198.6, 136.5, 133.4, 128.7, 128.0, 66.6, 47.9, 45.8, 36.3, 17.7 ppm. – IR: 2974, 2918, 2904, 2857, 1681, 1597, 1448, 1339, 1259, 1149, 1111, 1073, 947, 734, 691 cm^{-1}. – HRMS: calcd for $C_{14}H_{19}NNaO_4S$: 320.0927, found 320.0928 [M+Na^{+}].

Evaluation Only. Created with Aspose.Pdf. Copyright 2002-2014 Aspose Pty Ltd.

4-((3,4-Dimethoxyphenethyl)sulfonyl)morpholine (1c)

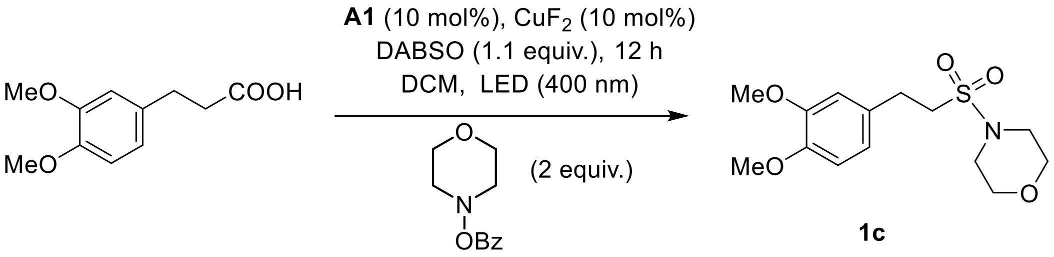

According to **GP1**, the reaction was carried out with 3-(3,4-dimethoxyphenyl)propanoic acid (63 mg, 0.3 mmol), DABSO (79 mg, 0.33 mmol, 1.1 equiv.), *N*-(benzoyloxy)morpholine (124 mg, 0.6 mmol, 2 equiv.), acridine catalyst **A1** (9 mg, 0.03 mmol, 10 mol%), copper difluoride (3 mg, 0.03 mmol, 10 mol%) in degassed dichloromethane (6 mL). The test-tube was capped and the reaction mixture was irradiated with LED light (λ = 400 nm) while stirring for 12 h. The reaction mixture was then quenched with saturated solution of sodium hydrogen carbonate (3 mL) and extracted with ethyl acetate (3 × 10 mL). The organic layer was collected, dried over anhydrous sodium sulfate, concentrated under reduced pressure, and the remaining material was purified by flash chromatography on silica gel (hexane/ethyl acetate 1 : 1 v/v) to give the sulfonamide product **1c** (57 mg, 60%) as a white solid.

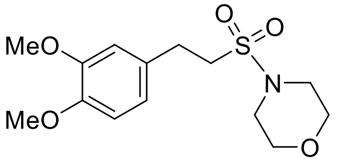

m.p.: 71–73°C. – ^{1}H NMR (500 MHz, $CDCl_3$): 6.86–6.68 (3 H, m), 3.86 (6 H, dd, *J* = 9.0, 3.0 Hz), 3.74 (4 H, q, *J* = 6.3, 4.2 Hz), 3.27 (4 H, q, *J* = 7.0, 4.2 Hz), 3.20–3.01 (4 H, m) ppm. – ^{13}C NMR (125 MHz, $CDCl_3$): 149.3, 148.2, 130.5, 120.4, 111.7, 111.6, 66.7, 56.1, 50.6, 45.9, 29.0 ppm. – IR: 3002, 2966, 2922, 2856, 1603, 1589, 1515, 1462, 1443, 1342, 1292, 1135, 1107, 1068, 1024, 938, 850, 791, 740, 704 cm^{-1}. – HRMS: calcd for $C_{14}H_{21}NO_5S$: 315.1140, found 315.1138 [M^+].

Evaluation Only. Created with Aspose.Pdf. Copyright 2002-2014 Aspose Pty Ltd.

N-(3-(Morpholinosulfonyl)propyl)-3-(4,4,5,5-tetramethyl-1,3,2-dioxaborolan-2-yl)benzamide (1d)

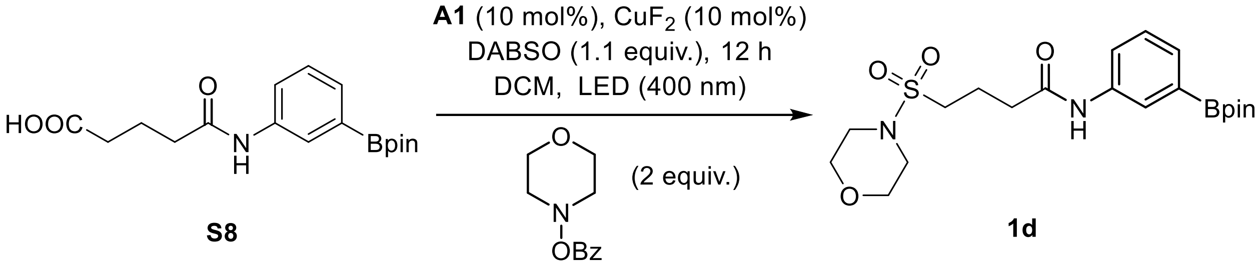

According to **GP1**, the reaction was carried out with acid **S8** (100 mg, 0.3 mmol), DABSO (79 mg, 0.33 mmol, 1.1 equiv.), *N*-(benzoyloxy)morpholine (124 mg, 0.6 mmol, 2 equiv.), acridine catalyst **A1** (9 mg, 0.03 mmol, 10 mol%), copper difluoride (3 mg, 0.03 mmol, 10 mol%) in degassed dichloromethane (6 mL). The test-tube was capped and the reaction mixture was irradiated with LED light (λ = 400 nm) while stirring for 12 h. The reaction mixture was then quenched with saturated solution of sodium hydrogen carbonate (3 mL) and extracted with ethyl acetate (3 × 10 mL). The organic layer was collected, dried over anhydrous sodium sulfate, concentrated under reduced pressure, and the remaining material was purified by flash chromatography on silica gel (hexane/ethyl acetate 7 : 3 v/v) to give the sulfonamide product **1d** (95 mg, 72%) as a white solid.

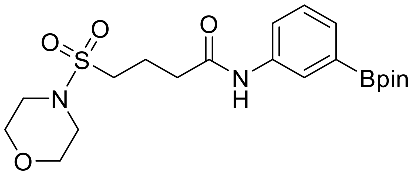

m.p.: 110–112°C. – ^{1}H NMR (500 MHz, $CDCl_3$): 7.92 (1 H, s), 7.79–7.71 (2 H, m), 7.52 (1 H, d, *J* = 7.3 Hz), 7.30 (1 H, t, *J* = 7.3 Hz), 3.75–3.66 (4 H, m), 3.25–3.17 (4 H, m), 3.02 (2 H, t, *J* = 7.1 Hz), 2.56 (2 H, t, *J* = 6.8 Hz), 2.21 (2 H, p, *J* = 7.0 Hz), 1.30 (12 H, s) ppm. – ^{13}C NMR (125 MHz, $CDCl_3$): 170.0, 137.4, 130.8, 128.6, 125.9, 123.1, 84.0, 66.5, 47.4, 45.8, 34.8, 24.9, 19.2 ppm. ^{11}B NMR (160 MHz, $CDCl_3$): 35.44 ppm. – IR: 3055, 2985, 2320, 1422, 1357, 1264, 1145, 895 cm^{-1}. – HRMS: calcd for $C_{20}H_{31}BN_2O_6SNa$: 461.1892, found 461.1888 [M+Na^{+}]

Go back to table of contents

Evaluation Only. Created with Aspose.Pdf. Copyright 2002-2014 Aspose Pty Ltd.

3-(Morpholinosulfonyl)-1-phenylpropan-1-one (1e)

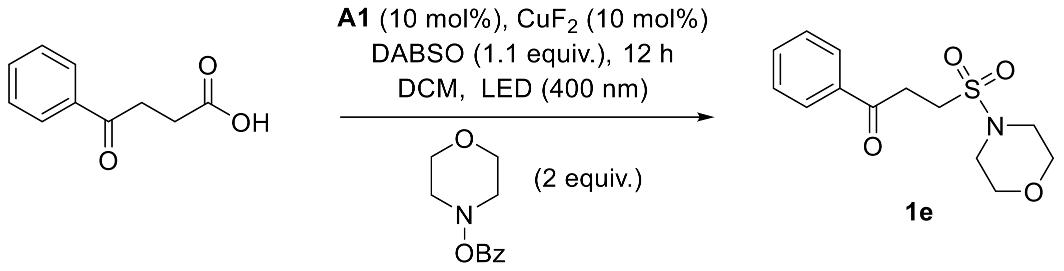

According to **GP1**, the reaction was carried out with 4-oxo-4-phenylbutanoic acid (53 mg, 0.3 mmol), DABSO (79 mg, 0.33 mmol, 1.1 equiv.), *N*-(benzoyloxy)morpholine (124 mg, 0.6 mmol, 2 equiv.), acridine catalyst **A1** (9 mg, 0.03 mmol, 10 mol%), copper difluoride (3 mg, 0.03 mmol, 10 mol%), and degassed dichloromethane (6 mL). The test-tube was capped and the reaction mixture was irradiated with LED light (λ = 400 nm) while stirring for 12 h. The reaction mixture was then quenched with saturated solution of sodium hydrogen carbonate (3 mL) and extracted with ethyl acetate (3 × 10 mL). The organic layer was collected, dried over anhydrous sodium sulfate, concentrated under reduced pressure, and the remaining material was purified by flash chromatography on silica gel (hexane/ ethyl acetate 4 : 1 v/v) to give the sulfone product **1e** (49 mg, 58%) as a colorless solid.

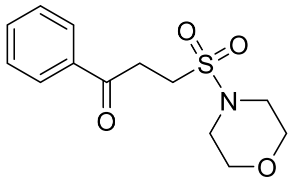

m.p.: 128–130 °C. – ^{1}H NMR (500 MHz, $CDCl_3$): 8.00–7.94 (2 H, m), 7.64–7.56 (1 H, m), 7.49 (2 H, t, *J* = 7.8 Hz), 3.75–3.69 (4 H, m), 3.51 (2 H, dd, *J* = 8.0, 6.6 Hz), 3.38 (2 H, dd, *J* = 7.9, 6.5 Hz), 3.29–3.24 (4 H, m) ppm. – ^{13}C NMR (125 MHz, $CDCl_3$): 196.0, 136.0, 134.0, 129.0, 128.2, 66.5, 45.8, 43.4, 32.1 ppm. – IR: 3062, 2956, 2914, 2893, 2856, 1682, 1596, 1580, 1447, 1417, 1359, 1341, 1259, 1147, 1111, 1072, 973, 951, 920, 846, 797, 746, 706, 693 cm^{-1}. – HRMS: calcd for $C_{13}H_{18}NO_4S$: 284.0951 found 284.0963 [M+H^{+}].

Evaluation Only. Created with Aspose.Pdf. Copyright 2002-2014 Aspose Pty Ltd.

1-(5-Methylthiophen-2-yl)-4-(morpholinosulfonyl)butan-1-one (1f)

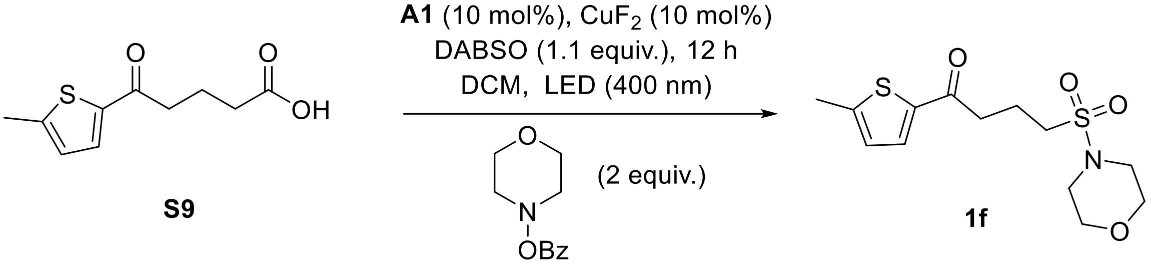

According to **GP1**, the reaction was carried out with acid **S9** (64 mg, 0.3 mmol), DABSO (79 mg, 0.33 mmol, 1.1 equiv.), *N*-(benzoyloxy)morpholine (124 mg, 0.6 mmol, 2 equiv.), acridine catalyst **A1** (9 mg, 0.03 mmol, 10 mol%), copper difluoride (3 mg, 0.03 mmol, 10 mol%), and degassed dichloromethane (6 mL). The test-tube was capped and the reaction mixture was irradiated with LED light (λ = 400 nm) while stirring for 12 h. The reaction mixture was then quenched with saturated solution of sodium hydrogen carbonate (3 mL) and extracted with ethyl acetate (3 × 10 mL). The organic layer was collected, dried over anhydrous sodium sulfate, concentrated under reduced pressure, and the remaining material was purified by flash chromatography on silica gel (hexane/ ethyl acetate 3 : 2 v/v) to give the sulfone product **1f** (75 mg, 79%) as a yellow solid.

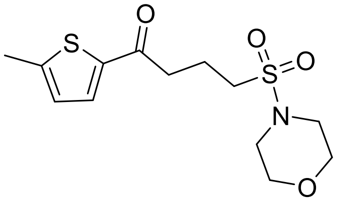

m.p.: 80–83 °C. – ^{1}H NMR (500 MHz, $CDCl_3$): 7.52 (1 H, d, *J* = 3.7 Hz), 6.77 (1 H, d, *J* = 3.7 Hz), 3.71 (4 H, t, *J* = 4.7 Hz), 3.27–3.18 (4 H, m), 3.04 (4 H, dt, *J* = 14.7, 7.1 Hz), 2.50 (3 H, s), 2.21 (2 H, quint., *J* = 7.0, 6.5 Hz) ppm. – ^{13}C NMR (125 MHz, $CDCl_3$): 191.2, 150.1, 141.5, 132.8, 127.0, 66.6, 47.9, 45.8, 36.3, 18.0, 16.1 ppm. – IR: 2974, 2925, 2901, 2857, 2253, 1651, 1452, 1339, 1324, 1259, 1151, 1112, 1072, 949, 915, 807, 729 cm^{-1}. – HRMS: calcd for $C_{13}H_{19}NNaO_4S_2$: 340.0648, found 340.0650 [M+Na$^+$].

Go back to table of contents

Evaluation Only. Created with Aspose.Pdf. Copyright 2002-2014 Aspose Pty Ltd.

1,7-Bis(morpholinosulfonyl)heptane (1g)

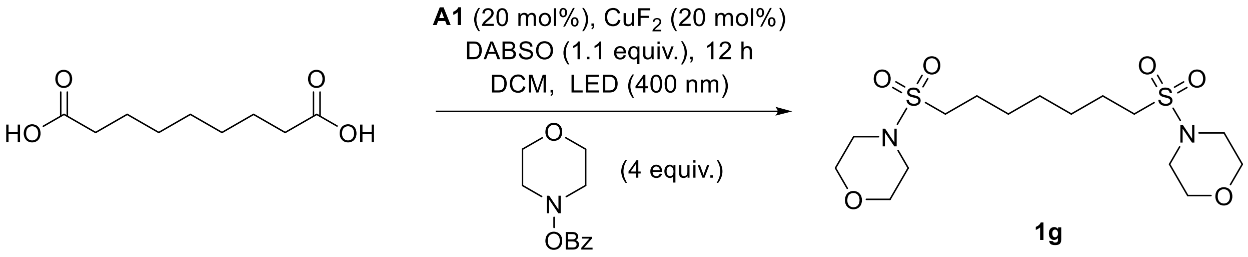

According to **GP1**, the reaction was carried out with azelaic acid (28 mg, 0.15 mmol), DABSO (79 mg, 0.33 mmol, 1.1 equiv.), *N*-(benzoyloxy)morpholine (124 mg, 0.6 mmol, 4 equiv.), acridine catalyst **A1** (9 mg, 0.03 mmol, 20 mol%), copper difluoride (3 mg, 0.03 mmol, 20 mol%), and degassed dichloromethane (6 mL). The test-tube was capped and the reaction mixture was irradiated with LED light (λ = 400 nm) while stirring for 12 h. The reaction mixture was then quenched with saturated solution of sodium hydrogen carbonate (3 mL) and extracted with ethyl acetate (3 × 10 mL). The organic layer was collected, dried over anhydrous sodium sulfate, concentrated under reduced pressure, and the remaining material was purified by flash chromatography on silica gel (hexane/ ethyl acetate 1 : 4 v/v) to give the sulfone product **1g** (30 mg, 50%) as a yellow solid.

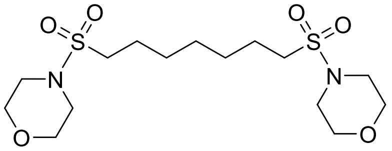

m.p.: 70–72°C. – ^{1}H NMR (500 MHz, $CDCl_3$): 3.79–3.73 (8 H, m), 3.29–3.23 (8 H, m), 2.93–2.84 (4 H, m), 1.88–1.76 (4 H, m), 1.51–1.34 (6 H, m) ppm. – ^{13}C NMR (125 MHz, $CDCl_3$): 66.7, 48.7, 46.0, 28.7, 28.2, 23.0 ppm. – IR: 2985, 2926, 2906, 2858, 1734, 1727, 1452, 1403, 1337, 1264, 1222, 1154, 1120, 1068, 1068, 953, 856, 736 cm^{-1}. – HRMS: calcd for $C_{15}H_{30}N_2NaO_6S_2$: 421.1427, found 421.1443 [M+H$^+$].

Go back to table of contents

Evaluation Only. Created with Aspose.Pdf. Copyright 2002-2014 Aspose Pty Ltd.

4-(Heptan-4-ylsulfonyl)morpholine (1h)

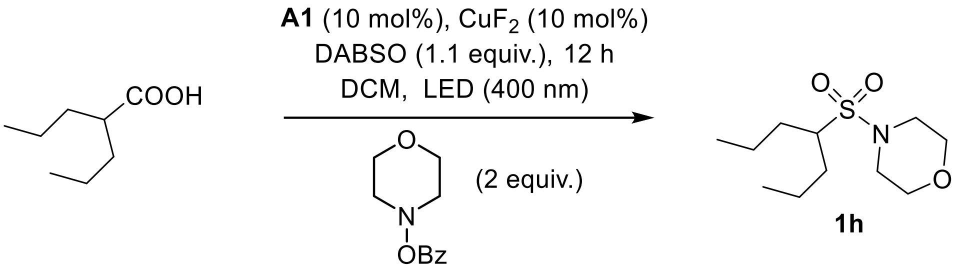

According to **GP1**, the reaction was carried out with 2-propylpentanoic acid (43 mg, 0.3 mmol), DABSO (79 mg, 0.33 mmol, 1.1 equiv.), *N*-(benzoyloxy)morpholine (124 mg, 0.6 mmol, 2 equiv.), acridine catalyst **A1** (9 mg, 0.03 mmol, 10 mol%), copper difluoride (3 mg, 0.03 mmol, 10 mol%) in degassed dichloromethane (6 mL). The test-tube was capped and the reaction mixture was irradiated with LED light (λ = 400 nm) while stirring for 12 h. The reaction mixture was then quenched with saturated solution of sodium hydrogen carbonate (3 mL) and extracted with ethyl acetate (3 × 10 mL). The organic layer was collected, dried over anhydrous sodium sulfate, concentrated under reduced pressure, and the remaining material was purified by flash chromatography on silica gel (hexane/ethyl acetate 9 : 1 v/v) to give the sulfonamide product **1h** (61 mg, 82%) as a yellow liquid.

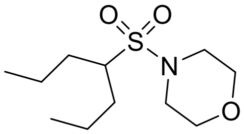

^{1}H NMR (500 MHz, $CDCl_3$): 3.77–3.68 (4 H, m), 3.39–3.26 (4 H, m), 2.90 (1 H, tt, *J* = 7.2, 4.7 Hz), 1.80 (2 H, dddd, *J* = 14.1, 10.6, 5.9, 4.8 Hz), 1.62 (2 H, dddd, *J* = 14.1, 10.4, 7.2, 5.1 Hz), 1.58–1.36 (4 H, m), 0.94 (6 H, t, *J* = 7.3 Hz) ppm. – ^{13}C NMR (125 MHz, $CDCl_3$): 67.1, 61.8, 46.4, 30.9, 20.1, 14.2 ppm. – IR: 3062, 2935, 2873, 2864, 1462, 1263, 1138, 1114, 1066 cm^{-1}. – HRMS: calcd for $C_{11}H_{23}NO_3SNa$: 272.1291, found 272.1291 [M+Na^{+}]

Evaluation Only. Created with Aspose.Pdf. Copyright 2002-2014 Aspose Pty Ltd.

4-((2,3-Dihydro-1*H*-inden-2-yl)sulfonyl)morpholine (1i)

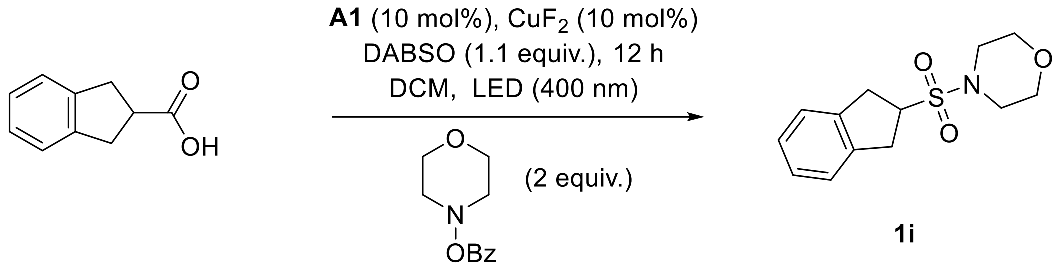

According to **GP1**, the reaction was carried out with 2,3-dihydro-1*H*-indene-2-carboxylic acid (49 mg, 0.3 mmol), DABSO (79 mg, 0.33 mmol, 1.1 equiv.), *N*-(benzoyloxy)morpholine (124 mg, 0.6 mmol, 2 equiv.), acridine catalyst **A1** (9 mg, 0.03 mmol, 10 mol%), copper difluoride (3 mg, 0.03 mmol, 10 mol%), and degassed dichloromethane (6 mL). The test-tube was capped and the reaction mixture was irradiated with LED light (λ = 400 nm) while stirring for 12 h. The reaction mixture was then quenched with saturated solution of sodium hydrogen carbonate (3 mL) and extracted with ethyl acetate (3 × 10 mL). The organic layer was collected, dried over anhydrous sodium sulfate, concentrated under reduced pressure, and the remaining material was purified by flash chromatography on silica gel (hexane/ ethyl acetate 4 : 1 v/v) to give the sulfone product **1i** (50 mg, 62%) as a colorless solid.

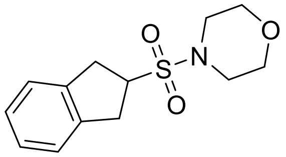

m.p.: 92–95 °C. – ^{1}H NMR (500 MHz, $CDCl_3$): 7.30–7.11 (5 H, m), 3.99 (1 H, quint., *J* = 7.9 Hz), 3.64 (4 H, t, *J* = 4.6 Hz), 3.43 (2 H, dd, *J* = 16.4, 7.1 Hz), 3.37–3.20 (6 H, m) ppm. – ^{13}C NMR (125 MHz, $CDCl_3$): 139.8, 127.4, 124.5, 66.9, 60.5, 46.4, 34.7 ppm. – IR: 3077, 3036, 2959, 2906, 2859, 1721, 1482, 1442, 1364, 1319, 1296, 1246, 1141, 1107, 1076, 1017, 952, 917, 842, 760, 739, 711 cm^{-1}. – HRMS: calcd for $C_{13}H_{17}NNaO_3S$: 290.0821, found 290.0813 [M+Na^{+}].

Go back to table of contents

Evaluation Only. Created with Aspose.Pdf. Copyright 2002-2014 Aspose Pty Ltd.

4-((4,4-Difluorocyclohexyl)sulfonyl)morpholine (1j)

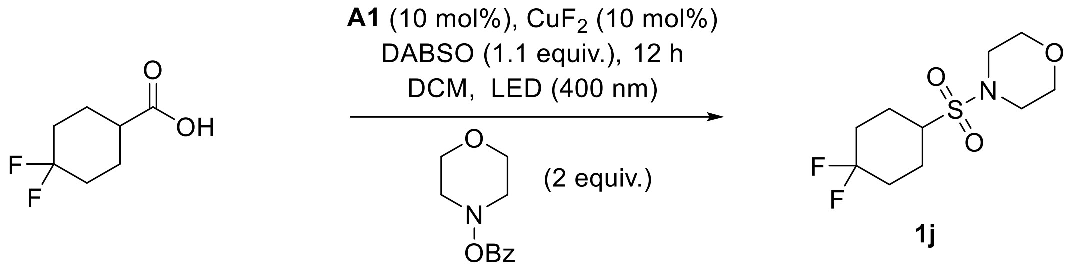

According to **GP1**, the reaction was carried out with 4,4-difluorocyclohexane-1-carboxylic acid (49 mg, 0.3 mmol), DABSO (79 mg, 0.33 mmol, 1.1 equiv.), *N*-(benzoyloxy)morpholine (124 mg, 0.6 mmol, 2 equiv.), acridine catalyst **A1** (9 mg, 0.03 mmol, 10 mol%), copper difluoride (3 mg, 0.03 mmol, 10 mol%), and degassed dichloromethane (6 mL). The test-tube was capped and the reaction mixture was irradiated with LED light (λ = 400 nm) while stirring for 12 h. The reaction mixture was then quenched with saturated solution of sodium hydrogen carbonate (3 mL) and extracted with ethyl acetate (3 × 10 mL). The organic layer was collected, dried over anhydrous sodium sulfate, concentrated under reduced pressure, and the remaining material was purified by flash chromatography on silica gel (hexane/ ethyl acetate 4 : 1 v/v) to give the sulfone product **1j** (65 mg, 81%) as a colorless solid.

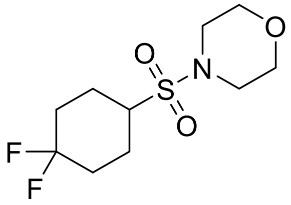

m.p.: 73–75 °C. – ^{1}H NMR (500 MHz, $CDCl_3$): 3.74–3.66 (4 H, m), 3.36–3.30 (4 H, m), 2.97 (1 H, ttd, *J* = 11.5, 3.7, 1.3 Hz), 2.32–2.10 (4H, m), 1.89 (2 H, qd, *J* = 12.8, 3.6 Hz), 1.82–1.69 (2 H, m) ppm. – ^{13}C NMR (125 MHz, $CDCl_3$): 122.9 (d, $^{1}J_{C-F}$ = 241.0 Hz), 67.1, 58.7, 46.5, 32.3 (t, $^{2}J_{C-F}$ = 25.0 Hz), 23.3 (d, $^{3}J_{C-F}$ = 9.3 Hz) ppm. – ^{19}F NMR (470 MHz, $CDCl_3$): –94.38 (d, *J* = 240.2 Hz), -102.01 (d, *J* = 240.1 Hz) ppm. – IR: 2969, 2930, 2860, 1455, 1322, 1261, 1148, 1108, 1074, 953, 743, 714 cm^{-1}. – HRMS: calcd for $C_{10}H_{17}F_2NO_3S$: 269.0897, found 269.0903 [M^{+}].

Evaluation Only. Created with Aspose.Pdf. Copyright 2002-2014 Aspose Pty Ltd.

tert-Butyl 4-(morpholinosulfonyl)piperidine-1-carboxylate (1k)

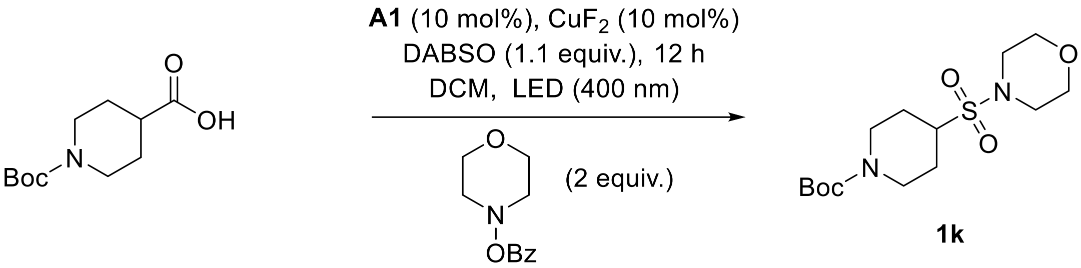

According to **GP1**, the reaction was carried out with 1-(*tert*-butoxycarbonyl)piperidine-4-carboxylic acid (69 mg, 0.3 mmol), DABSO (79 mg, 0.33 mmol, 1.1 equiv.), *N*-(benzoyloxy)morpholine (124 mg, 0.6 mmol, 2 equiv.), acridine catalyst **A1** (9 mg, 0.03 mmol, 10 mol%), copper difluoride (3 mg, 0.03 mmol, 10 mol%), and degassed dichloromethane (6 mL). The test-tube was capped and the reaction mixture was irradiated with LED light (λ = 400 nm) while stirring for 12 h. The reaction mixture was then quenched with saturated solution of sodium hydrogen carbonate (3 mL) and extracted with ethyl acetate (3 × 10 mL). The organic layer was collected, dried over anhydrous sodium sulfate, concentrated under reduced pressure, and the remaining material was purified by flash chromatography on silica gel (hexane/ ethyl acetate 1 : 1 v/v) to give the sulfone product **1k** (70 mg, 70%) as a colorless solid.

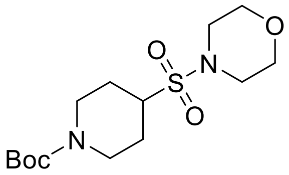

m.p.: 105–107 °C. – ^{1}H NMR (500 MHz, $CDCl_3$): 4.29–4.11 (2 H, m), 3.72–3.62 (4 H, m), 3.30 (4 H, dd, *J* = 5.6, 3.8 Hz), 3.01 (1 H, tt, *J* = 12.0, 3.7 Hz), 2.75–2.55 (2 H, m), 2.03–1.91 (2 H, m), 1.65 (2 H, qd, *J* = 12.5, 4.6 Hz), 1.41 (9 H, s) ppm. – ^{13}C NMR (125 MHz, $CDCl_3$): 154.4, 80.1, 67.0, 59.4, 46.4, 42.6, 28.4, 26.0 ppm. – IR: 2980, 2959, 2925, 2865, 1676, 1460, 1406, 1364, 1314, 1284, 1142, 1111, 1076, 1006, 982, 948, 874, 779, 720 cm^{-1}. – HRMS: calcd for $C_{14}H_{26}N_2NaO_5S$: 357.1455, found 357.1462 [M+Na^{+}].

Go back to table of contents

Evaluation Only. Created with Aspose.Pdf. Copyright 2002-2014 Aspose Pty Ltd.

4-((Tetrahydro-2*H*-pyran-4-yl)sulfonyl)morpholine (1l)

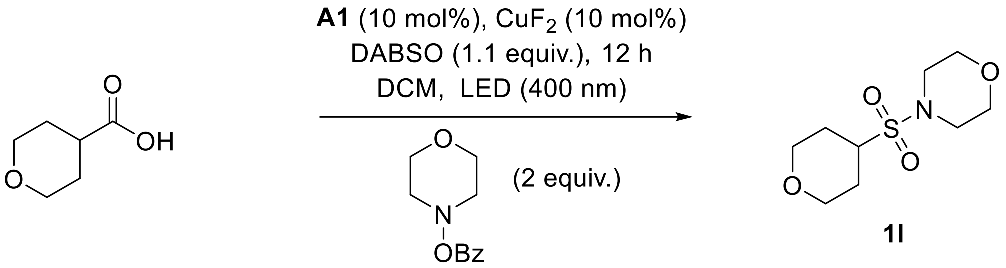

1l

According to **GP1**, the reaction was carried out with tetrahydro-2H-pyran-4-carboxylic acid (39 mg, 0.3 mmol), DABSO (79 mg, 0.33 mmol, 1.1 equiv.), *N*-(benzoyloxy)morpholine (124 mg, 0.6 mmol, 2 equiv.), acridine catalyst **A1** (9 mg, 0.03 mmol, 10 mol%), copper difluoride (3 mg, 0.03 mmol, 10 mol%), and degassed dichloromethane (6 mL). The test-tube was capped and the reaction mixture was irradiated with LED light (λ = 400 nm) while stirring for 12 h. The reaction mixture was then quenched with saturated solution of sodium hydrogen carbonate (3 mL) and extracted with ethyl acetate (3 × 10 mL). The organic layer was collected, dried over anhydrous sodium sulfate, concentrated under reduced pressure, and the remaining material was purified by flash chromatography on silica gel (hexane/ ethyl acetate 1 : 4 v/v) to give the sulfone product **1l** (51 mg, 72%) as a colorless solid.

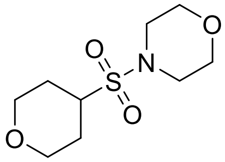

m.p.: 80–82 °C. – ^{1}H NMR (500 MHz, $CDCl_3$): 4.05 (2 H, ddd, *J* = 11.6, 4.7, 1.8 Hz), 3.73–3.66 (4 H, m), 3.41–3.29 (6 H, m), 3.13 (1 H, tt, *J* = 11.8, 4.0 Hz), 1.99–1.75 (4 H, m) ppm. – ^{13}C NMR (125 MHz, $CDCl_3$): 67.1, 66.6, 58.4, 46.5, 26.7 ppm. – IR: 2957, 2920, 2851, 1717, 1444, 1366, 1317, 1298, 1261, 1233, 1137, 1108, 1077, 1019, 984, 888, 788, 717 cm^{-1}. – HRMS: calcd for $C_9H_{17}NNaO_4S$: 258.0770, found 258.0771 [M+H$^+$].

Go back to table of contents

Evaluation Only. Created with Aspose.Pdf. Copyright 2002-2014 Aspose Pty Ltd.

Methyl (1*S*,2*R*)-2-(morpholinosulfonyl)cyclohexane-1-carboxylate (1m)

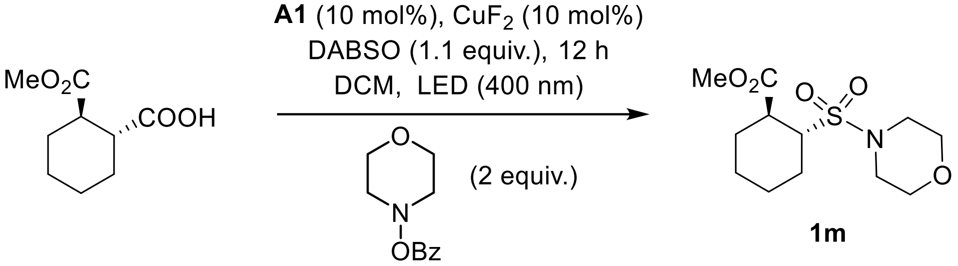

1m

According to **GP1**, the reaction was carried out with (1*R*,2*R*)-2-(methoxycarbonyl)cyclohexane-1-carboxylic acid (56 mg, 0.3 mmol), DABSO (79 mg, 0.33 mmol, 1.1 equiv.), *N*-(benzoyloxy)morpholine (124 mg, 0.6 mmol, 2 equiv.), acridine catalyst **A1** (9 mg, 0.03 mmol, 10 mol%), copper difluoride (3 mg, 0.03 mmol, 10 mol%) in degassed dichloromethane (6 mL). The test-tube was capped and the reaction mixture was irradiated with LED light (λ = 400 nm) while stirring for 12 h. The reaction mixture was then quenched with saturated solution of sodium hydrogen carbonate (3 mL) and extracted with ethyl acetate (3 × 10 mL). The organic layer was collected, dried over anhydrous sodium sulfate, concentrated under reduced pressure, and the remaining material was purified by flash chromatography on silica gel (hexane/ethyl acetate 7 : 3 v/v) to give the sulfonamide product **1m** (59 mg, 68%) as a yellow liquid.

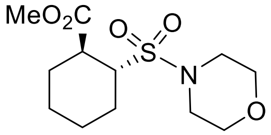

^{1}H NMR (500 MHz, $CDCl_3$): 3.74 (7 H, tq, *J* = 9.2, 4.5 Hz), 3.44 (1 H, td, *J* = 11.7, 5.4 Hz), 3.31 (4 H, q, *J* = 5.4 Hz), 2.67 (1 H, ddd, *J* = 15.8, 9.6, 3.9 Hz), 2.12 (2 H, ddt, *J* = 47.0, 13.7, 4.1 Hz), 1.97–1.86 (1 H, m), 1.82–1.72 (1 H, m), 1.71–1.45 (2 H, m), 1.41–1.21 (2 H, m) ppm. – ^{13}C NMR (125 MHz, $CDCl_3$): 174.8, 68.1, 67.0, 61.0, 52.3, 46.3, 43.7, 30.5, 25.7, 25.7, 24.4, 24.1 ppm. – IR: 2985, 2922, 2860, 1732, 1453, 1322, 1261, 1221, 1149, 1114, 1074, 952, 737 cm^{-1}. – HRMS: calcd for $C_{12}H_{21}NO_5SNa$: 314.1033, found 314.1031 [M+Na^{+}].

Evaluation Only. Created with Aspose.Pdf. Copyright 2002-2014 Aspose Pty Ltd.

Ethyl (1*R*,2*S*,4*S*)-3-(morpholinosulfonyl)bicyclo[2.2.1]heptane-2-carboxylate (1n)

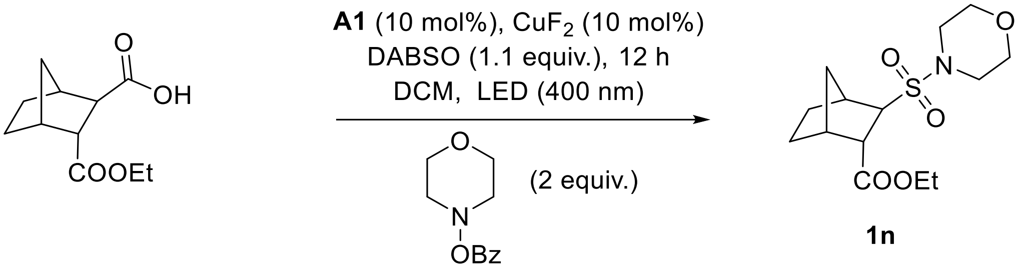

1n

According to **GP1**, the reaction was carried out with (1*S*,3*R*,4*R*)-3-(ethoxycarbonyl)bicyclo[2.2.1]heptane-2-carboxylic acid (64 mg, 0.3 mmol), DABSO (79 mg, 0.33 mmol, 1.1 equiv.), *N*-(benzoyloxy)morpholine (124 mg, 0.6 mmol, 2 equiv.), acridine catalyst **A1** (9 mg, 0.03 mmol, 10 mol%), copper difluoride (3 mg, 0.03 mmol, 10 mol%), and degassed dichloromethane (6 mL). The test-tube was capped and the reaction mixture was irradiated with LED light (λ = 400 nm) while stirring for 12 h. The reaction mixture was then quenched with saturated solution of sodium hydrogen carbonate (3 mL) and extracted with ethyl acetate (3 × 10 mL). The organic layer was collected, dried over anhydrous sodium sulfate, concentrated under reduced pressure, and the remaining material was purified by flash chromatography on silica gel (hexane/ ethyl acetate 1 : 1 v/v) to give the sulfone product **1n** (75 mg, 79%) as a colorless oil.

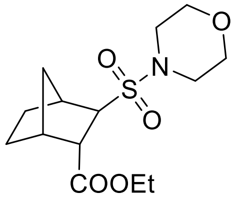

^{1}H NMR (500 MHz, $CDCl_3$): 4.21 (2 H, q, *J* = 7.1 Hz), 3.67 (4 H, ddd, *J* = 5.3, 3.9, 1.3 Hz), 3.45 (1 H, dd, *J* = 5.8, 1.4 Hz), 3.32–3.25 (2 H, m), 3.25–3.19 (2 H, m), 3.11 (1 H, ddd, *J* = 6.0, 4.3, 1.9 Hz), 2.79 (1 H, dd, *J* = 4.4, 1.5 Hz), 2.69 (1 H, td, *J* = 4.2, 1.6 Hz), 2.02 (1 H, dt, *J* = 10.4, 1.9 Hz, 1.68 (1 H, tt, *J* = 12.5, 4.7 Hz), 1.49 (1 H, ttd, *J* = 12.6, 4.2, 1.9 Hz), 1.39–1.31 (2 H, m), 1.29 (3 H, t, *J* = 7.1 Hz), 1.22 (1 H, dddd, *J* = 1.8, 8.9, 4.5, 2.2 Hz) ppm. – ^{13}C NMR (125 MHz, $CDCl_3$): 172.3, 66.9, 63.5, 61.4, 50.1, 45.9, 40.6, 40.1, 38.4, 29.3, 24.0, 14.5 ppm. – IR: 2975, 2930, 2860, 1727, 1454, 1325, 1296, 1260, 1220, 1187, 1151, 1114, 1074, 951, 733 cm^{-1}. – HRMS: calcd for $C_{14}H_{24}NO_5S$: 318.1370, found 318.1360 [M+H$^+$].

Go back to table of contents

Evaluation Only. Created with Aspose.Pdf. Copyright 2002-2014 Aspose Pty Ltd.

4-((1-Phenylcyclopropyl)sulfonyl)morpholine (1o)

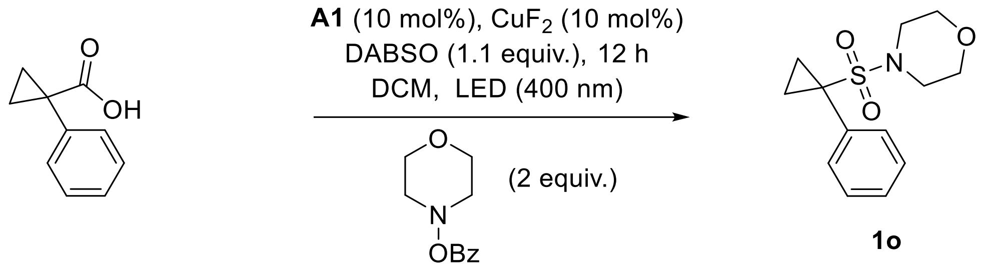

1o

According to **GP1**, the reaction was carried out with 1-phenylcyclopropane-1-carboxylic acid (49 mg, 0.3 mmol), DABSO (79 mg, 0.33 mmol, 1.1 equiv.), *N*-(benzoyloxy)morpholine (124 mg, 0.6 mmol, 2 equiv.), acridine catalyst **A1** (9 mg, 0.03 mmol, 10 mol%), copper difluoride (3 mg, 0.03 mmol, 10 mol%), and degassed dichloromethane (6 mL). The test-tube was capped and the reaction mixture was irradiated with LED light (λ = 400 nm) while stirring for 12 h. The reaction mixture was then quenched with saturated solution of sodium hydrogen carbonate (3 mL) and extracted with ethyl acetate (3 × 10 mL). The organic layer was collected, dried over anhydrous sodium sulfate, concentrated under reduced pressure, and the remaining material was purified by flash chromatography on silica gel (hexane/ ethyl acetate 4 : 1 v/v) to give the sulfone product **1o** (48 mg, 60%) as a colorless solid.

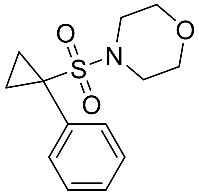

m.p.: 78–80 °C. – ^{1}H NMR (500 MHz, $CDCl_3$): 7.59–7.53 (2 H, m), 7.40–7.32 (3 H, m), 3.56–3.50 (2 H, m), 3.03–2.95 (2 H, m), 1.75–1.70 (2 H, m), 1.23 (2 H, m) ppm. – ^{13}C NMR (125 MHz, $CDCl_3$): 134.6, 132.0, 129.2, 128.6, 66.9, 46.5, 44.3, 12.4 ppm. – IR: 2984, 2914, 2857, 1496, 1447, 1334, 1313, 1260, 1215, 1138, 1115, 1069, 1043, 952, 932, 903, 804, 770, 720, 702 cm^{-1}. – HRMS: calcd for $C_{13}H_{18}NO_3S$: 268.1002, found 268.1009 [M+H$^+$].

Evaluation Only. Created with Aspose.Pdf. Copyright 2002-2014 Aspose Pty Ltd.

4-((4-Methyltetrahydro-2*H*-pyran-4-yl)sulfonyl)morpholine (1p)

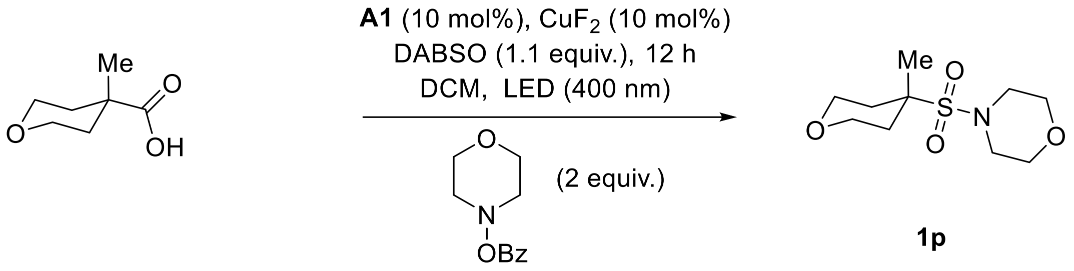

According to **GP1**, the reaction was carried out with 4-methyltetrahydro-2*H*-pyran-4-carboxylic acid (43 mg, 0.3 mmol), DABSO (79 mg, 0.33 mmol, 1.1 equiv.), *N*-(benzoyloxy)morpholine (124 mg, 0.6 mmol, 2 equiv.), acridine catalyst **A1** (9 mg, 0.03 mmol, 10 mol%), copper difluoride (3 mg, 0.03 mmol, 10 mol%), and degassed dichloromethane (6 mL). The test-tube was capped and the reaction mixture was irradiated with LED light (λ = 400 nm) while stirring for 12 h. The reaction mixture was then quenched with saturated solution of sodium hydrogen carbonate (3 mL) and extracted with ethyl acetate (3 × 10 mL). The organic layer was collected, dried over anhydrous sodium sulfate, concentrated under reduced pressure, and the remaining material was purified by flash chromatography on silica gel (hexane/ ethyl acetate 1 : 4 v/v) to give the sulfone product **1p** (50 mg, 67%) as a slightly yellow solid.

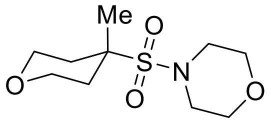

m.p.: 70–72 °C – ^{1}H NMR (500 MHz, $CDCl_3$ 3.90 (2 H, ddd, *J* = 12.1, 5.1, 2.1 Hz), 3.68 (4H, t, *J* = 4.7 Hz), 3.48 (2 H, td, *J* = 12.0, 2.2 Hz), 3.37 (4 H, t, *J* = 4.7 Hz), 2.14 (2 H, td, *J* = 12.7, 5.1 Hz), 1.54 (2 H, dt, *J* = 13.0, 2.4 Hz), 1.46 (3H, s) ppm. – ^{13}C NMR (125 MHz, $CDCl_3$): 67.3, 63.2, 62.2, 47.9, 31.2, 17.4 ppm. – IR: 2962, 2943, 2909, 2866, 1629, 1474, 1450, 1328, 1286, 1260, 1137, 1104, 1067, 1039, 1010, 938, 851, 809, 718 cm^{-1}. – HRMS: calcd for $C_{10}H_{19}NNaO_4S$: 272.0927, found 272.0925 [M+Na$^+$].

Go back to table of contents

Evaluation Only. Created with Aspose.Pdf. Copyright 2002-2014 Aspose Pty Ltd.

1-Phenyl-4-(piperidin-1-ylsulfonyl)butan-1-one (2a)

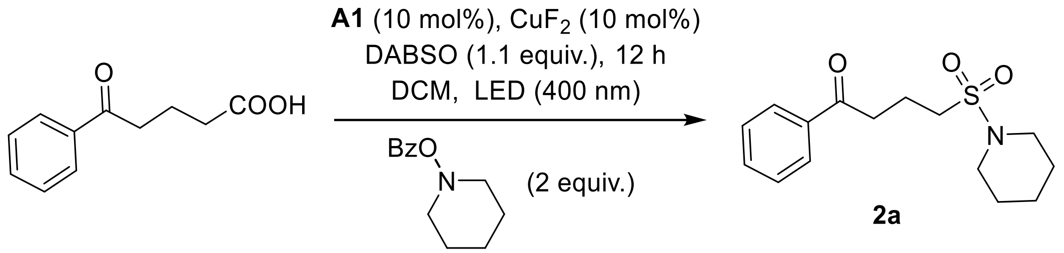

According to **GP1**, the reaction was carried out with 5-oxo-5-phenylpentanoic acid (58 mg, 0.3 mmol), DABSO (79 mg, 0.33 mmol, 1.1 equiv.), *N*-(benzoyloxy)piperidine (123 mg, 0.6 mmol, 2 equiv.), acridine catalyst **A1** (9 mg, 0.03 mmol, 10 mol%), copper difluoride (3 mg, 0.03 mmol, 10 mol%) in degassed dichloromethane (6 mL). The test-tube was capped and the reaction mixture was irradiated with LED light (λ = 400 nm) while stirring for 12 h. The reaction mixture was then quenched with saturated solution of sodium hydrogen carbonate (3 mL) and extracted with ethyl acetate (3 × 10 mL). The organic layer was collected, dried over anhydrous sodium sulfate, concentrated under reduced pressure, and the remaining material was purified by flash chromatography on silica gel (hexane/ethyl acetate 7 : 3 v/v) to give the sulfonamide product **2a** (55 mg, 62%) as a yellow liquid.

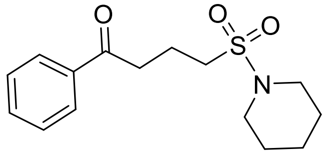

^{1}H NMR (500 MHz, $CDCl_3$): 7.98–7.93 (2 H, m), 7.61–7.52 (1 H, m), 7.46 (2 H, dd, *J* = 8.4, 7.1 Hz), 3.28–3.16 (6 H, m), 3.03 (2 H, dd, *J* = 8.0, 6.7 Hz), 2.30–2.18 (2 H, m), 1.70–1.51 (6 H, m) ppm. – ^{13}C NMR (125 MHz, $CDCl_3$): 198.9, 136.7, 133.5, 128.8, 128.1, 48.3, 46.8, 36.5, 25.8, 23.9, 17.9 ppm. – IR: 2986, 2945, 2850, 1739, 1685, 1558, 1332, 1265, 1161, 1143 cm^{-1}. – HRMS: calcd for $C_{15}H_{21}NO_3SNa$: 318.1134, found 318.1133 [M+Na^{+}].

Evaluation Only. Created with Aspose.Pdf. Copyright 2002-2014 Aspose Pty Ltd.

1-((Tetrahydro-2*H*-pyran-4-yl)sulfonyl)piperidine (2b)

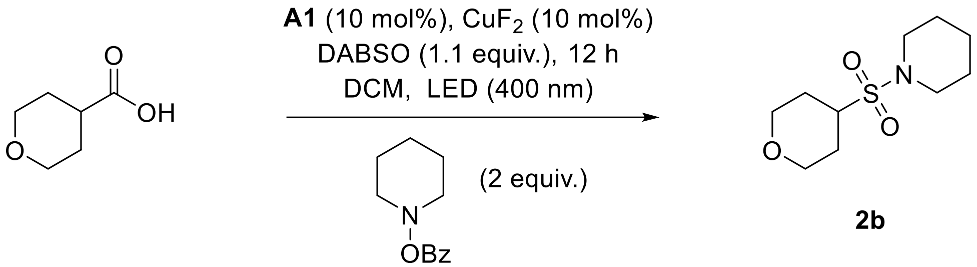

2b

According to **GP1**, the reaction was carried out with tetrahydro-2*H*-pyran-4-carboxylic acid (39 mg, 0.3 mmol), DABSO (79 mg, 0.33 mmol, 1.1 equiv.), *N*-(benzoyloxy)piperidine (123 mg, 0.6 mmol, 2 equiv.), acridine catalyst **A1** (9 mg, 0.03 mmol, 10 mol%), copper difluoride (3 mg, 0.03 mmol, 10 mol%), and degassed dichloromethane (6 mL). The test-tube was capped and the reaction mixture was irradiated with LED light (λ = 400 nm) while stirring for 12 h. The reaction mixture was then quenched with saturated solution of sodium hydrogen carbonate (3 mL) and extracted with ethyl acetate (3 × 10 mL). The organic layer was collected, dried over anhydrous sodium sulfate, concentrated under reduced pressure, and the remaining material was purified by flash chromatography on silica gel (hexane/ ethyl acetate 3 : 2 v/v) to give the sulfone product **2b** (56 mg, 80%) as a colorless solid.

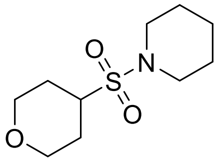

m.p.: 65–67 °C. – ^{1}H NMR (500 MHz, $CDCl_3$): ^{1}H NMR (499 MHz, Chloroform-*d*) δ 4.09–3.99 (2 H, m), 3.38–3.24 (4 H, m), 3.09 (1 H, tt, *J* = 11.8, 4.0 Hz), 1.95–1.90 (2 H, m), 1.85 (2 H, qd, *J* = 12.3, 4.6 Hz), 1.62-1.54(6 H, m) ppm. – ^{13}C NMR (125 MHz, $CDCl_3$): ^{13}C NMR (75 MHz, $CDCl_3$) δ 66.79, 58.44, 47.38, 26.90, 26.30, 24.05 ppm. – IR: 2970, 2938, 2875, 2853, 1745, 1468, 1379, 1329, 1240, 1138, 1089, 1055, 953, 895, 738, 712 cm^{-1}. – HRMS: calcd for $C_{10}H_{20}NO_3S$: 234.1158, found 234.1160 [M+H$^+$].

Evaluation Only. Created with Aspose.Pdf. Copyright 2002-2014 Aspose Pty Ltd.

1-(Heptan-4-ylsulfonyl)piperidine (2c)

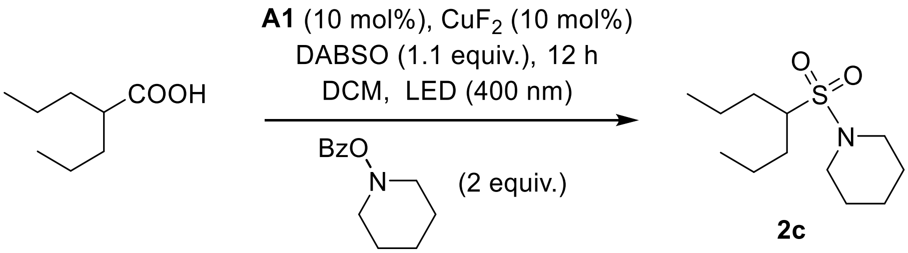

According to **GP1**, the reaction was carried out with 2-propylpentanoic acid (43 mg, 0.3 mmol), DABSO (79 mg, 0.33 mmol, 1.1 equiv.), N-(benzoyloxy)piperidine (123 mg, 0.6 mmol, 2 equiv.), acridine catalyst **A1** (9 mg, 0.03 mmol, 10 mol%), copper difluoride (3 mg, 0.03 mmol, 10 mol%) in degassed dichloromethane (6 mL). The test-tube was capped and the reaction mixture was irradiated with LED light (λ = 400 nm) while stirring for 12 h. The reaction mixture was then quenched with saturated solution of sodium hydrogen carbonate (3 mL) and extracted with ethyl acetate (3 × 10 mL). The organic layer was collected, dried over anhydrous sodium sulfate, concentrated under reduced pressure, and the remaining material was purified by flash chromatography on silica gel (hexane/ethyl acetate 7 : 3 v/v) to give the sulfonamide product **2c** (54 mg, 73%) as a yellow liquid.

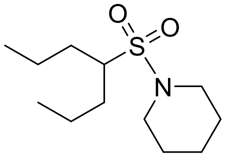

^{1}H NMR (500 MHz, $CDCl_3$): 3.34–3.27 (4 H, m), 2.88 (1 H, tt, *J* = 7.1, 4.8 Hz), 1.82 (2 H, dddd, *J* = 13.9, 10.4, 5.9, 4.7 Hz), 1.67–1.40 (12 H, m), 0.95 (6 H, t, *J* = 7.3 Hz) ppm. – ^{13}C NMR (125 MHz, $CDCl_3$): 61.6, 47.1, 31.0, 26.3, 24.1, 20.2, 14.2 ppm. – IR: 2958, 2936, 2872, 2855, 1764, 1465, 1330, 1319, 1161, 1134 cm^{-1}. – HRMS: calcd for $C_{12}H_{26}NO_2S$: 248.1679, found 248.1679 [M+H$^+$].

Evaluation Only. Created with Aspose.Pdf. Copyright 2002-2014 Aspose Pty Ltd.

Methyl (1*S,2*R**)-2-(piperidin-1-ylsulfonyl)cyclohexane-1-carboxylate (2d)**

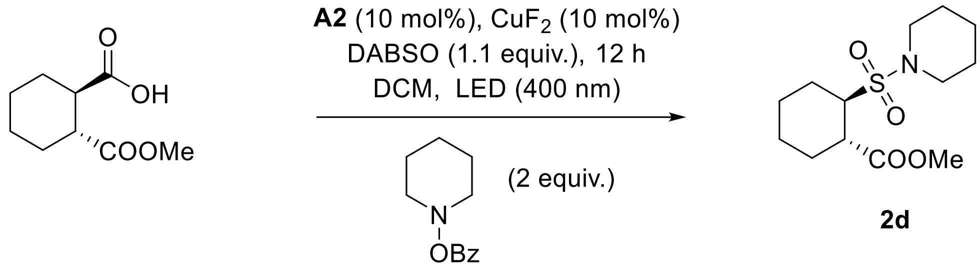

According to **GP1**, the reaction was carried out with (1*R**,2*R**)-2-(methoxycarbonyl)cyclohexane-1-carboxylic acid (56 mg, 0.3 mmol), DABSO (79 mg, 0.33 mmol, 1.1 equiv.), *N*-(benzoyloxy)piperidine (123 mg, 0.6 mmol, 2 equiv.), acridine catalyst **A1** (9 mg, 0.03 mmol, 10 mol%), copper difluoride (3 mg, 0.03 mmol, 10 mol%), and degassed dichloromethane (6 mL). The test-tube was capped and the reaction mixture was irradiated with LED light (λ = 400 nm) while stirring for 12 h. The reaction mixture was then quenched with saturated solution of sodium hydrogen carbonate (3 mL) and extracted with ethyl acetate (3 × 10 mL). The organic layer was collected, dried over anhydrous sodium sulfate, concentrated under reduced pressure, and the remaining material was purified by flash chromatography on silica gel (hexane/ ethyl acetate 3 : 2 v/v) to give the sulfone product **2d** (65 mg, 80%) as a colorless liquid.

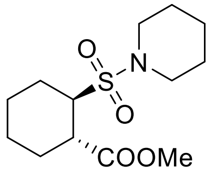

^{1}H NMR (500 MHz, $CDCl_3$): 3.69 (3 H, s), 3.34 (1 H, td, *J* = 11.5, 4.1 Hz), 3.27–3.13 (4 H, m), 2.62 (1 H, td, *J* = 11.4, 4.2 Hz), 2.10 (1 H, dd, *J* = 13.6, 4.0 Hz), 2.06–1.99 (1 H, m), 1.86–1.79 (1 H, m), 1.77–1.66 (1 H, m), 1.61–1.51 (7 H, m), 1.50–1.40 (1 H, m), 1.35–1.16 (2 H, m) ppm. – ^{13}C NMR (125 MHz, $CDCl_3$): 174.9, 60.9, 52.2, 47.0, 43.7, 30.1, 26.1, 25.7, 24.4, 24.2, 24.0 ppm. – IR: 3014, 2959, 2862, 1737, 1445, 1365, 1322, 1278, 1217, 1163, 1138, 1053, 1023, 938 cm^{-1}. – HRMS: calcd for $C_{13}H_{23}NNaO_4S$: 312.1240, found 312.1241 [M+H$^+$].

Evaluation Only. Created with Aspose.Pdf. Copyright 2002-2014 Aspose Pty Ltd.

1-Adamantan-1-yl)sulfonyl)piperidine (2e)

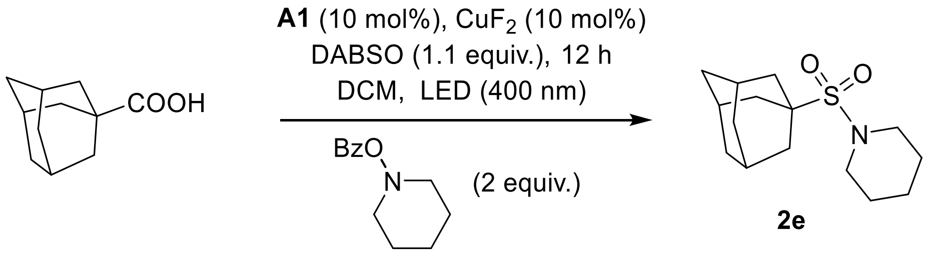

According to **GP1**, the reaction was carried out with adamantane-1-carboxylic acid (54 mg, 0.3 mmol), DABSO (79 mg, 0.33 mmol, 1.1 equiv.), N-(benzoyloxy)piperidine (123 mg, 0.6 mmol, 2 equiv.), acridine catalyst **A1** (9 mg, 0.03 mmol, 10 mol%), copper difluoride (3 mg, 0.03 mmol, 10 mol%) in degassed dichloromethane (6 mL). The test-tube was capped and the reaction mixture was irradiated with LED light (λ = 400 nm) while stirring for 12 h. The reaction mixture was then quenched with saturated solution of sodium hydrogen carbonate (3 mL) and extracted with ethyl acetate (3 × 10 mL). The organic layer was collected, dried over anhydrous sodium sulfate, concentrated under reduced pressure, and the remaining material was purified by flash chromatography on silica gel (hexane/ethyl acetate 7 : 3 v/v) to give the sulfonamide product **2e** (58 mg, 68%) as a yellow solid.

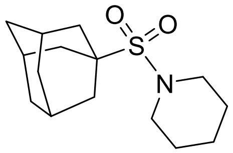

m.p.: 45–47°C. – ^{1}H NMR (500 MHz, $CDCl_3$): 3.42–3.23 (4 H, m), 2.14 (3 H, p, J = 3.1 Hz), 2.04–1.96 (6 H, m), 1.77–1.67 (6 H, m), 1.66–1.60 (6 H, m) ppm. – ^{13}C NMR (125 MHz, $CDCl_3$): 62.4, 48.3, 36.1, 36.0, 28.2, 26.5, 24.0 ppm. – IR: 2950, 2914, 2855, 1738, 1455, 1365, 1306, 1264, 1216, 1142, 1062, 943 cm^{-1}. – HRMS: calcd for $C_{15}H_{25}NO_2SNa$: 306.1498, found 306.1497 [M+Na$^+$].

Go back to table of contents

Evaluation Only. Created with Aspose.Pdf. Copyright 2002-2014 Aspose Pty Ltd.

1-(*tert*-Pentylsulfonyl)piperidine (2f)

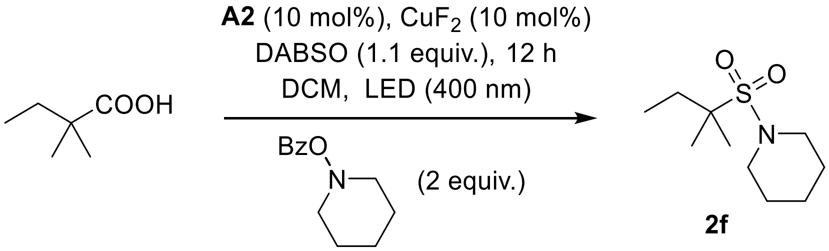

According to **GP1**, the reaction was carried out with 2,2-dimethylbutanoic acid (35 mg, 0.3 mmol), DABSO (79 mg, 0.33 mmol, 1.1 equiv.), N-(benzoyloxy)piperidine (123 mg, 0.6 mmol, 2 equiv.), acridine catalyst **A1** (9 mg, 0.03 mmol, 10 mol%), copper difluoride (3 mg, 0.03 mmol, 10 mol%) in degassed dichloromethane (6 mL). The test-tube was capped and the reaction mixture was irradiated with LED light (λ = 400 nm) while stirring for 12 h. The reaction mixture was then quenched with saturated solution of sodium hydrogen carbonate (3 mL) and extracted with ethyl acetate (3 × 10 mL). The organic layer was collected, dried over anhydrous sodium sulfate, concentrated under reduced pressure, and the remaining material was purified by flash chromatography on silica gel (hexane/ethyl acetate 7 : 3 v/v) to give the sulfonamide product **2f** (49 mg, 74%) as a yellow liquid.

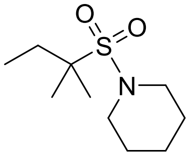

^{1}H NMR (500 MHz, $CDCl_3$): 3.43–3.25 (4 H, m), 1.78 (2 H, q, *J* = 7.6 Hz), 1.67–1.56 (6 H, m), 1.29 (6 H, s), 0.97 (3 H, t, *J* = 7.6 Hz) ppm. – ^{13}C NMR (125 MHz, $CDCl_3$): 64.8, 48.6, 28.9, 26.61, 24.1, 21.0, 8.5 ppm. – IR: 3014, 2975, 2953, 2901, 2862, 1712, 1478, 1315, 1261, 1180, 1116, 1067, 952, 715 cm^{-1}. – HRMS: calcd for $C_{10}H_{21}NO_2SNa$: 242.1185, found 242.1185 [M+Na$^+$].

Evaluation Only. Created with Aspose.Pdf. Copyright 2002-2014 Aspose Pty Ltd.

N-(*tert*-Butyl)-4-phenylbutane-1-sulfonamide (3a)

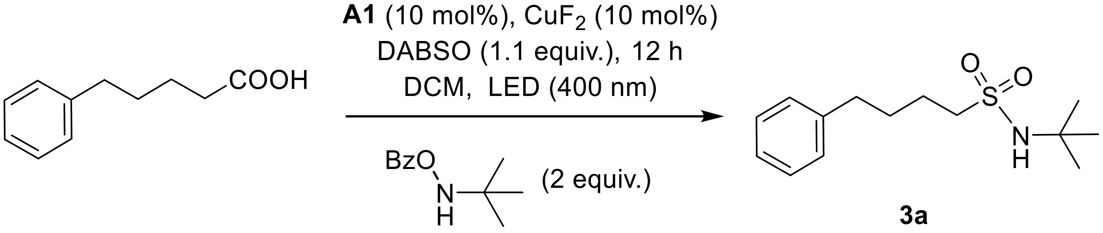

3a

According to **GP1**, the reaction was carried out with 5-phenylpentanoic acid (53 mg, 0.3 mmol), DABSO (79 mg, 0.33 mmol, 1.1 equiv.), *O*-benzoyl-*N*-(*tert*-butyl)hydroxylamine (116 mg, 0.6 mmol, 2 equiv.), acridine catalyst **A1** (9 mg, 0.03 mmol, 10 mol%), copper difluoride (3 mg, 0.03 mmol, 10 mol%) in degassed dichloromethane (6 mL). The test-tube was capped and the reaction mixture was irradiated with LED light (λ = 400 nm) while stirring for 12 h. The reaction mixture was then quenched with saturated solution of sodium hydrogen carbonate (3 mL) and extracted with ethyl acetate (3 × 10 mL). The organic layer was collected, dried over anhydrous sodium sulfate, concentrated under reduced pressure, and the remaining material was purified by flash chromatography on silica gel (hexane/ethyl acetate 7 : 3 v/v) to give the sulfonamide product **3a** (74 mg, 92%) as a yellow liquid.

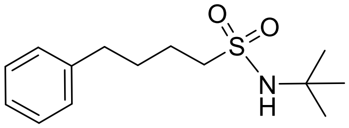

^{1}H NMR (500 MHz, $CDCl_3$): 7.30–7.26 (2 H, m), 7.22–7.15 (3 H, m), 4.30 (1 H, s), 3.10–2.97 (2 H, m), 2.65 (2 H, t, *J* = 7.5 Hz), 1.90–1.81 (2 H, m), 1.80–1.71 (2 H, m), 1.34 (9 H, s) ppm. – ^{13}C NMR (125 MHz, $CDCl_3$): 141.6, 128.5, 128.5, 126.1, 56.1, 54.6, 35.5, 30.4, 30.1, 23.8 ppm. – IR: 3460, 3277, 3071, 3016, 2970, 2944, 2873, 1739, 1434, 1365, 1228, 1217, 1138, 1092, 900 cm^{-1}. – HRMS: calcd for $C_{14}H_{23}NO_2SNa$: 292.1342, found 292.1339 [M+Na$^+$].

Go back to table of contents

Evaluation Only. Created with Aspose.Pdf. Copyright 2002-2014 Aspose Pty Ltd.

N-(tert-Butyl)tetrahydro-2H-pyran-4-sulfonamide (3b)

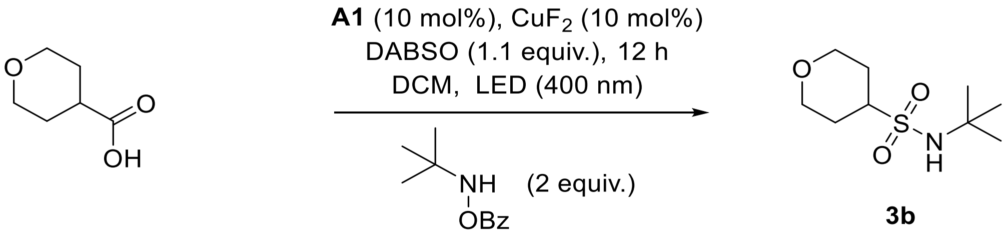

According to **GP1**, the reaction was carried out with tetrahydro-2*H*-pyran-4-carboxylic acid (39 mg, 0.3 mmol), DABSO (79 mg, 0.33 mmol, 1.1 equiv.), *O*-benzoyl-*N*-(*tert*-butyl)hydroxylamine (116 mg, 0.6 mmol, 2 equiv.), acridine catalyst **A1** (9 mg, 0.03 mmol, 10 mol%), copper difluoride (3 mg, 0.03 mmol, 10 mol%), and degassed dichloromethane (6 mL). The test-tube was capped and the reaction mixture was irradiated with LED light (λ = 400 nm) while stirring for 12 h. The reaction mixture was then quenched with saturated solution of sodium hydrogen carbonate (3 mL) and extracted with ethyl acetate (3 × 10 mL). The organic layer was collected, dried over anhydrous sodium sulfate, concentrated under reduced pressure, and the remaining material was purified by flash chromatography on silica gel (hexane/ ethyl acetate 7 : 3 v/v) to give the sulfone product **3b** (52 mg, 78%) as a slightly yellow solid.

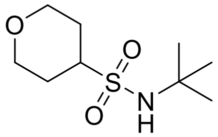

m.p.: 117–119 °C. – ^{1}H NMR (500 MHz, CDCl$_3$): 4.49 (1 H, s), 4.10–4.03 (2 H, m), 3.34 (2 H, td, *J* = 12.0, 2.1 Hz), 2.99 (1 H, tt, *J* = 11.9, 3.8 Hz), 2.00 (2 H, ddt, *J* = 12.8, 4.1, 2.0 Hz), 1.84 (2 H, dtd, *J* = 13.4, 12.0, 4.7 Hz), 1.35 (9 H, s) ppm. – ^{13}C NMR (125 MHz, CDCl$_3$): 66.7, 60.5, 54.9, 30.5, 26.8 ppm. – IR: 3193, 2968, 2942, 2857, 1472, 1446, 1385, 1311, 1300, 1263, 1234, 1127, 1005, 921, 889, 787, 733 cm^{-1}. – HRMS: calcd for $C_9H_{19}NNaO_3S$: 244.0978, found 244.0976 [M+Na$^+$].

Evaluation Only. Created with Aspose.Pdf. Copyright 2002-2014 Aspose Pty Ltd.

tert-Butyl 4-(*N*-(*tert*-butyl)sulfamoyl)piperidine-1-carboxylate (3c)

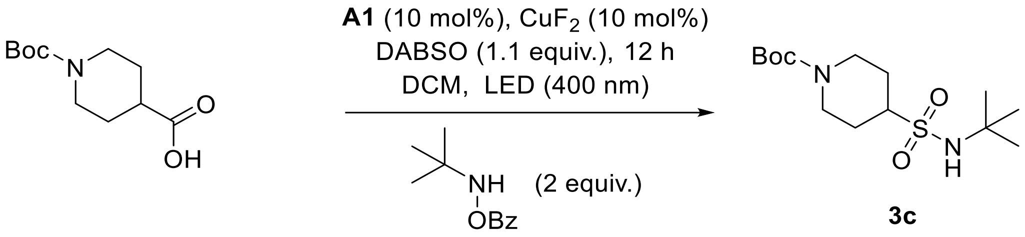

According to **GP1**, the reaction was carried out with 1-(*tert*-butoxycarbonyl)piperidine-4-carboxylic acid (69 mg, 0.3 mmol), DABSO (79 mg, 0.33 mmol, 1.1 equiv.), *O*-benzoyl-*N*-(*tert*-butyl)hydroxylamine (116 mg, 0.6 mmol, 2 equiv.), acridine catalyst **A1** (9 mg, 0.03 mmol, 10 mol%), copper difluoride (3 mg, 0.03 mmol, 10 mol%), and degassed dichloromethane (6 mL). The test-tube was capped and the reaction mixture was irradiated with LED light (λ = 400 nm) while stirring for 12 h. The reaction mixture was then quenched with saturated solution of sodium hydrogen carbonate (3 mL) and extracted with ethyl acetate (3 × 10 mL). The organic layer was collected, dried over anhydrous sodium sulfate, concentrated under reduced pressure, and the remaining material was purified by flash chromatography on silica gel (hexane/ ethyl acetate 3 : 2 v/v) to give the sulfone product **3c** (70 mg, 73%) as a colorless solid.

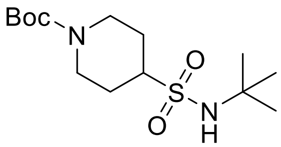

m.p.: 128–130 °C. – ^{1}H NMR (500 MHz, $CDCl_3$): 4.31 (1 H, s), 4.23 (1 H, s), 2.90 (1 H, tt, *J* = 11.9, 3.7 Hz), 2.75–2.55 (2 H, m), 2.08 (2 H, ddd, *J* = 12.6, 4.0, 2.1 Hz), 1.67 (2 H, qd, *J* = 12.5, 4.6 Hz), 1.43 (9 H, s), 1.35 (9H, s) ppm. – ^{13}C NMR (125 MHz, $CDCl_3$): 154.5, 80.0, 61.7, 55.0, 42.8, 30.5, 28.5, 26.2 ppm. – IR: 3295, 2972, 2961, 2928, 2872, 1709, 1670, 1421, 1363, 1316, 1222, 1133, 1002, 880, 731 cm^{-1}. – HRMS: calcd for $C_{14}H_{28}N_2NaO_4S$: 343.1662, found 343.1656 [M+Na$^+$].

Evaluation Only. Created with Aspose.Pdf. Copyright 2002-2014 Aspose Pty Ltd.

N-(*tert*-Butyl)-2-methylpropane-2-sulfonamide (3d)

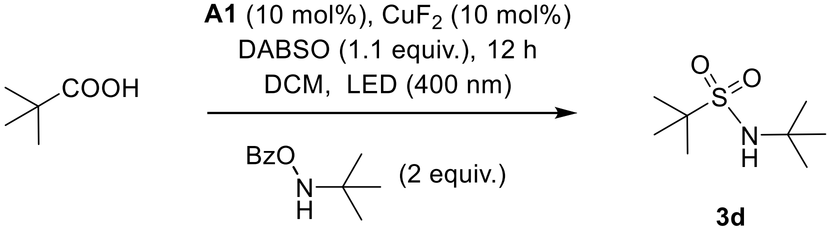

3d

According to **GP1**, the reaction was carried out with pivalic acid (31 mg, 0.3 mmol), DABSO (79 mg, 0.33 mmol, 1.1 equiv.), *O*-benzoyl-*N*-(*tert*-butyl)hydroxylamine (116 mg, 0.6 mmol, 2 equiv.), acridine catalyst **A1** (9 mg, 0.03 mmol, 10 mol%), copper difluoride (3 mg, 0.03 mmol, 10 mol%) in degassed dichloromethane (6 mL). The test-tube was capped and the reaction mixture was irradiated with LED light ($\lambda = 400$ nm) while stirring for 12 h. The reaction mixture was then quenched with saturated solution of sodium hydrogen carbonate (3 mL) and extracted with ethyl acetate (3 × 10 mL). The organic layer was collected, dried over anhydrous sodium sulfate, concentrated under reduced pressure, and the remaining material was purified by flash chromatography on silica gel (hexane/ethyl acetate 7 : 3 v/v) to give the sulfonamide product **3d** (37 mg, 63%) as a yellow liquid.

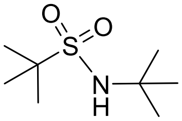

^{1}H NMR (500 MHz, $CDCl_3$): 3.50 (1 H, s), 1.43–1.40 (18 H, m) ppm. – ^{13}C NMR (125 MHz, $CDCl_3$): 59.6, 56.0, 30.9, 24.5 ppm. – IR: 3055, 2985, 2326, 2260, 1738, 1367, 1304, 1264, 1122, 907 cm^{-1}. – HRMS: calcd for $C_8H_{19}NO_2SNa$: 216.1029, found 216.1028 [M+Na$^+$].

Evaluation Only. Created with Aspose.Pdf. Copyright 2002-2014 Aspose Pty Ltd.

N-(*tert*-Butyl)adamantane-1-sulfonamide (3e)

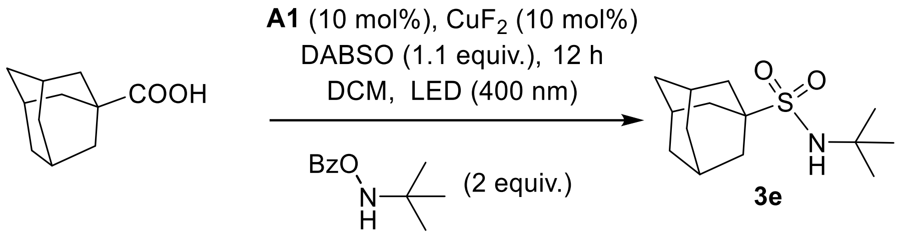

According to **GP1**, the reaction was carried out with adamantane-1-carboxylic acid (54 mg, 0.3 mmol), DABSO (79 mg, 0.33 mmol, 1.1 equiv.), *O*-benzoyl-*N*-(*tert*-butyl)hydroxylamine (116 mg, 0.6 mmol, 2 equiv.), acridine catalyst **A1** (9 mg, 0.03 mmol, 10 mol%), copper difluoride (3 mg, 0.03 mmol, 10 mol%) in degassed dichloromethane (6 mL). The test-tube was capped and the reaction mixture was irradiated with LED light (λ = 400 nm) while stirring for 12 h. The reaction mixture was then quenched with saturated solution of sodium hydrogen carbonate (3 mL) and extracted with ethyl acetate (3 × 10 mL). The organic layer was collected, dried over anhydrous sodium sulfate, concentrated under reduced pressure, and the remaining material was purified by flash chromatography on silica gel (hexane/ethyl acetate 7 : 3 v/v) to give the sulfonamide product **3e** (49 mg, 60%) as a white solid.

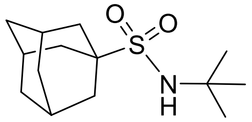

m.p.: 118–120°C. – ^{1}H NMR (500 MHz, $CDCl_3$): 3.43 (1 H, s), 2.13 (3 H, q, *J* = 3.1 Hz), 2.00 (6 H, d, *J* = 3.0 Hz), 1.75–1.64 (6 H, m), 1.38 (9 H, s) ppm. – ^{13}C NMR (125 MHz, $CDCl_3$): 60.5, 55.6, 35.9, 35.7, 30.8, 28.2 ppm. – IR: 3288, 2994, 2960, 2897, 2852, 1737, 1455, 1426, 1288, 1228, 1133, 982 cm^{-1}. – HRMS: calcd for $C_{14}H_{25}NO_2SNa$: 294.1498, found 294.1496 [M+Na$^+$].

Go back to table of contents

Evaluation Only. Created with Aspose.Pdf. Copyright 2002-2014 Aspose Pty Ltd.

N-(*tert*-Butyl)-5-(2,5-dimethylphenoxy)-2-methylpentane-2-sulfonamide (3f)

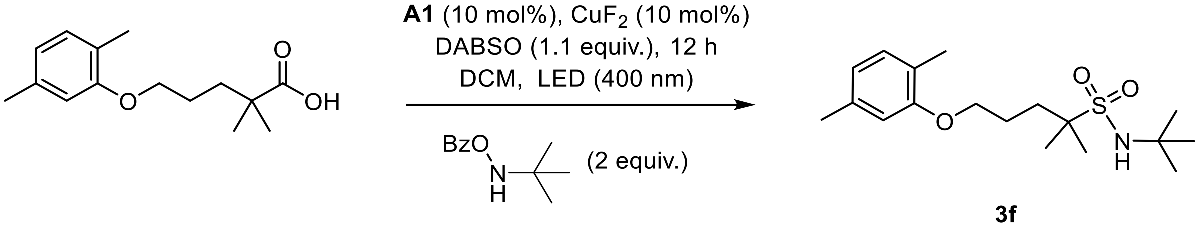

3f

According to **GP1**, the reaction was carried out with gemfibrozil (75 mg, 0.3 mmol), DABSO (79 mg, 0.33 mmol, 1.1 equiv.), *O*-benzoyl-*N*-(*tert*-butyl)hydroxylamine (116 mg, 0.6 mmol, 2 equiv.), acridine catalyst **A1** (9 mg, 0.03 mmol, 10 mol%), copper difluoride (3 mg, 0.03 mmol, 10 mol%) in degassed dichloromethane (6 mL). The test-tube was capped and the reaction mixture was irradiated with LED light (λ = 400 nm) while stirring for 12 h. The reaction mixture was then quenched with saturated solution of sodium hydrogen carbonate (3 mL) and extracted with ethyl acetate (3 × 10 mL). The organic layer was collected, dried over anhydrous sodium sulfate, concentrated under reduced pressure, and the remaining material was purified by flash chromatography on silica gel (hexane/ethyl acetate 7 : 3 v/v) to give the sulfonamide product **3f** (58 mg, 57%) as a yellow solid.

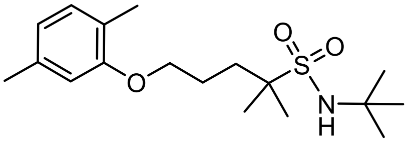

m.p.: 50–52°C.–^{1}H NMR (500 MHz, $CDCl_3$): 7.01 (1 H, d, *J* = 7.5 Hz), 6.67 (1 H, dd, *J* = 7.6, 1.6 Hz), 6.62 (1 H, d, *J* = 1.6 Hz), 3.97 (2 H, t, *J* = 5.9 Hz), 3.58 (1 H, s), 2.31 (3 H, s), 2.18 (3 H, s), 1.97 (2 H, qd, *J* = 6.3, 2.6 Hz), 1.93–1.84 (2 H, m), 1.40 (9 H, s), 1.39 (6 H, s)ppm. – ^{13}C NMR (125 MHz, $CDCl_3$): 156.9, 136.6, 130.5, 123.7, 121.0, 112.1, 67.8, 62.5, 56.1, 32.8, 30.9, 24.6, 21.6, 21.5, 15.9 ppm. – IR: 3295, 2977, 2929, 2862, 1732, 1670, 1422, 1392, 1317, 1285, 1229, 1166, 1134, 1070, 1002, 880, 787 cm^{-1}. – HRMS: calcd for $C_{18}H_{31}NNaO_3S$: 364.1917, found 364.1915 [M+Na^{+}].

Go back to table of contents

Evaluation Only. Created with Aspose.Pdf. Copyright 2002-2014 Aspose Pty Ltd.

N-Benzylpentadecane-1-sulfonamide (4a)

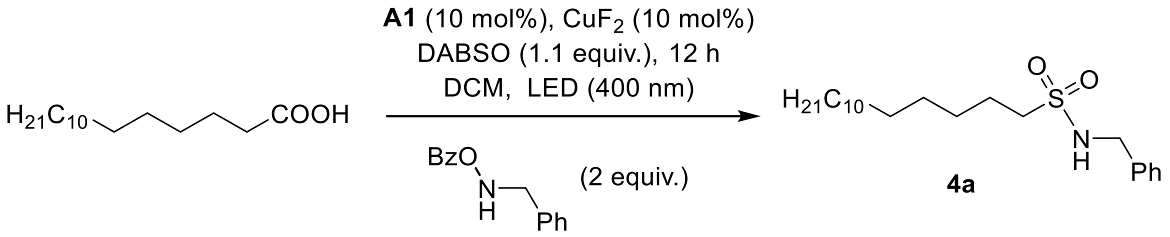

According to **GP1**, the reaction was carried out with palmitic acid (77 mg, 0.3 mmol), DABSO (79 mg, 0.33 mmol, 1.1 equiv.), *O*-benzoyl-*N*-benzylhydroxylamine (136 mg, 0.6 mmol, 2 equiv.), acridine catalyst **A1** (9 mg, 0.03 mmol, 10 mol%), copper difluoride (3 mg, 0.03 mmol, 10 mol%) in degassed dichloromethane (6 mL). The test-tube was capped and the reaction mixture was irradiated with LED light (λ = 400 nm) while stirring for 12 h. The reaction mixture was then quenched with saturated solution of sodium hydrogen carbonate (3 mL) and extracted with ethyl acetate (3 × 10 mL). The organic layer was collected, dried over anhydrous sodium sulfate, concentrated under reduced pressure, and the remaining material was purified by flash chromatography on silica gel (hexane/ethyl acetate 3 : 2 v/v) to give the sulfonamide product **4a** (62 mg, 54%) as a white solid.

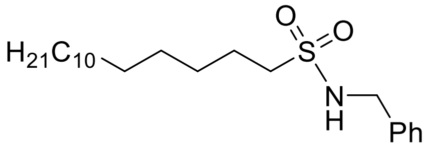

m.p.: 50–52°C. – ^{1}H NMR (500 MHz, $CDCl_3$): 7.43–7.27 (5 H, m), 4.62 (1 H, p, *J* = 6.1 Hz), 4.29 (2 H, d, *J* = 6.1 Hz), 2.98–2.84 (2 H, m), 1.84–1.66 (2 H, m), 1.38–1.14 (24 H, m), 0.88 (3 H, t, *J* = 6.9 Hz) ppm. – ^{13}C NMR (125 MHz, $CDCl_3$): 137.1, 129.0, 128.2, 128.2, 53.5, 47.4, 32.1, 29.8, 29.8, 29.7, 29.7, 29.5, 29.4, 29.2, 28.4, 23.7, 22.8, 14.3 ppm. – IR: 2985, 2836, 2154, 2015, 1265, 1189, 895 cm^{-1}. – HRMS: calcd for $C_{22}H_{40}NO_2S$: 382.2780, found 382.2755 [M+H^{+}].

Go back to table of contents

Evaluation Only. Created with Aspose.Pdf. Copyright 2002-2014 Aspose Pty Ltd.

N-Benzyltetrahydro-2*H*-pyran-4-sulfonamide (4b)

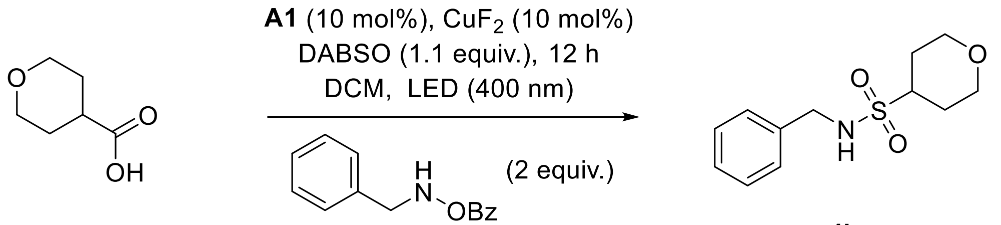

4b

According to **GP1**, the reaction was carried out with tetrahydro-2*H*-pyran-4-carboxylic acid (39 mg, 0.3 mmol), DABSO (79 mg, 0.33 mmol, 1.1 equiv.), *O*-benzoyl-*N*-benzylhydroxylamine (136 mg, 0.6 mmol, 2 equiv.), acridine catalyst **A1** (9 mg, 0.03 mmol, 10 mol%), copper difluoride (3 mg, 0.03 mmol, 10 mol%), and degassed dichloromethane (6 mL) were added. The test-tube was capped and the reaction mixture was irradiated with LED light (λ = 400 nm) while stirring for 12 h. The reaction mixture was then quenched with saturated solution of sodium hydrogen carbonate (3 mL) and extracted with ethyl acetate (3 × 10 mL). The organic layer was collected, dried over anhydrous sodium sulfate, concentrated under reduced pressure, and the remaining material was purified by flash chromatography on silica gel (hexane/ ethyl acetate 3 : 2 v/v) to give the sulfone product **4b** (44 mg, 57%) as a colorless solid.

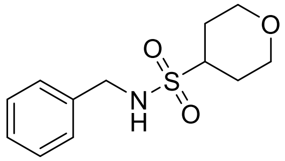

m.p.: 100–103 °C. – ^{1}H NMR (500 MHz, $CDCl_3$): 7.39–7.23 (5 H, m), 4.92 (1 H, brs), 4.28 (2 H, d, *J* = 6.0 Hz), 4.00 (2 H, dt, *J* = 11.9, 3.0 Hz), 3.24 (2 H, td, *J* = 11.9, 2.2 Hz), 2.92 (1 H, tt, *J* = 11.9, 3.9 Hz), 1.91 (2 H, ddd, *J* = 12.9, 4.3, 2.1 Hz), 1.81 (2 H, qd, *J* = 12.3, 4.6 Hz) ppm. – ^{13}C NMR (125 MHz, $CDCl_3$): 137.3, 128.9, 128.1, 128.0, 66.6, 58.9, 47.6, 26.6 ppm. – IR: 3282, 3250, 3063, 2976, 2948, 2921, 2850, 1605, 1494, 1451, 1423, 1380, 1299, 1238, 1133, 1063, 1009, 859, 730 cm^{-1}. – HRMS: calcd for $C_{12}H_{17}NNaO_3S$: 278.0821, found 278.0817 [$M+Na^+$].

Evaluation Only. Created with Aspose.Pdf. Copyright 2002-2014 Aspose Pty Ltd.

N-Benzyl-2-methylpropane-2-sulfonamide (4c)

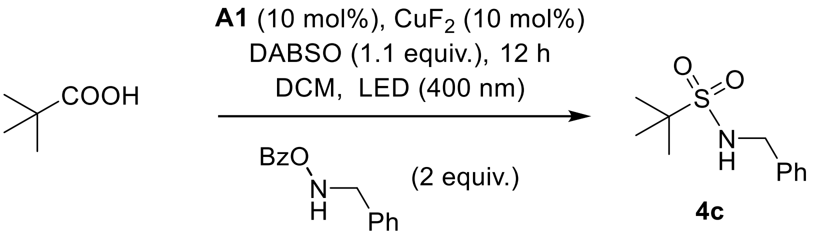

According to **GP1**, the reaction was carried out with pivalic acid (31 mg, 0.3 mmol), DABSO (79 mg, 0.33 mmol, 1.1 equiv.), *O*-benzoyl-*N*-benzylhydroxylamine (136 mg, 0.6 mmol, 2 equiv.), acridine catalyst **A1** (9 mg, 0.03 mmol, 10 mol%), copper difluoride (3 mg, 0.03 mmol, 10 mol%) in degassed dichloromethane (6 mL). The test-tube was capped and the reaction mixture was irradiated with LED light (λ = 400 nm) while stirring for 12 h. The reaction mixture was then quenched with saturated solution of sodium hydrogen carbonate (3 mL) and extracted with ethyl acetate (3 × 10 mL). The organic layer was collected, dried over anhydrous sodium sulfate, concentrated under reduced pressure, and the remaining material was purified by flash chromatography on silica gel (hexane/ethyl acetate 7 : 3 v/v) to give the sulfonamide product **4c** (34 mg, 50%) as a white solid.

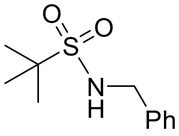

m.p.: 83–85°C. – ^{1}H NMR (500 MHz, $CDCl_3$): 7.38–7.28 (5 H, m), 4.36 (2 H, d, *J* = 6.0 Hz), 4.20 (1 H, d, *J* = 6.9 Hz), 1.42 (9 H, s) ppm. – ^{13}C NMR (125 MHz, $CDCl_3$): 137.9, 128.9, 128.0, 127.9, 60.2, 48.7, 24.5 ppm. – IR: 3284, 3025, 2971, 1875, 1606, 1495, 1474, 1453, 1395, 1364, 1296, 1202, 1118, 1094 cm^{-1}. – HRMS: calcd for $C_{11}H_{17}NO_2SNa$: 250.0872, found 250.0872 [M+Na$^+$].

Evaluation Only. Created with Aspose.Pdf. Copyright 2002-2014 Aspose Pty Ltd.

N-Benzyladamantane-1-sulfonamide (4d)

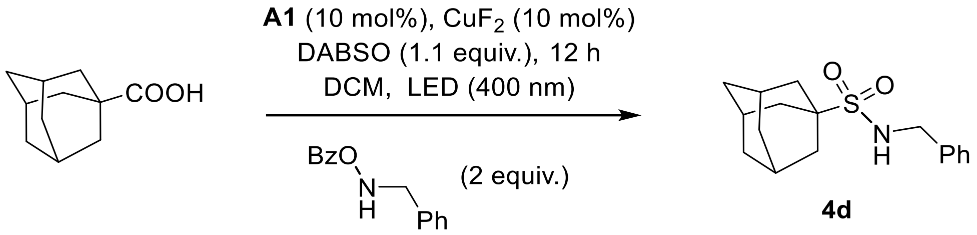

According to **GP1**, the reaction was carried out with adamantane-1-carboxylic acid (54 mg, 0.3 mmol), DABSO (79 mg, 0.33 mmol, 1.1 equiv.), *O*-benzoyl-*N*-benzylhydroxylamine (136 mg, 0.6 mmol, 2 equiv.), acridine catalyst **A1** (9 mg, 0.03 mmol, 10 mol%), copper difluoride (3 mg, 0.03 mmol, 10 mol%) in degassed dichloromethane (6 mL). The test-tube was capped and the reaction mixture was irradiated with LED light (λ = 400 nm) while stirring for 12 h. The reaction mixture was then quenched with saturated solution of sodium hydrogen carbonate (3 mL) and extracted with ethyl acetate (3 × 10 mL). The organic layer was collected, dried over anhydrous sodium sulfate, concentrated under reduced pressure, and the remaining material was purified by flash chromatography on silica gel (hexane/ethyl acetate 7 : 3 v/v) to give the sulfonamide product **4d** (38 mg, 41%) as a white solid.

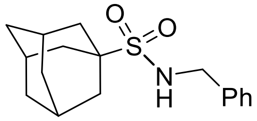

m.p.: 148–150°C. – ^{1}H NMR (500 MHz, $CDCl_3$): 7.42–7.23 (5 H, m), 4.47–4.03 (3 H, m), 2.20–2.09 (3 H, m), 2.03 (6 H, d, *J* = 3.0 Hz), 1.77–1.59 (6 H, m) ppm. – ^{13}C NMR (125 MHz, $CDCl_3$): 138.1, 128.9, 127.9, 127.8, 61.3, 48.6, 36.0, 35.9, 28.3 ppm. – IR: 3054, 2985, 2865, 1265, 896, 737, 703 cm^{-1}. – HRMS: calcd for $C_{17}H_{23}NO_2SNa$: 328.1342, found 328.1343 [M+Na^{+}].

Evaluation Only. Created with Aspose.Pdf. Copyright 2002-2014 Aspose Pty Ltd.

N-(4-Fluorophenethyl)-4-oxo-4-phenylbutane-1-sulfonamide (5a)

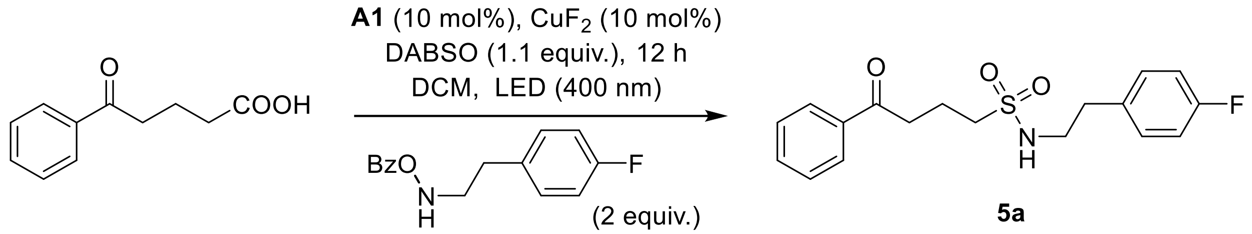

5a

According to **GP1**, the reaction was carried out with 5-oxo-5-phenylpentanoic acid (58 mg, 0.3 mmol), DABSO (79 mg, 0.33 mmol, 1.1 equiv.), *O*-benzoyl-*N*-(4-fluorophenethyl)hydroxylamine (155 mg, 0.6 mmol, 2 equiv.), acridine catalyst **A1** (9 mg, 0.03 mmol, 10 mol%), copper difluoride (3 mg, 0.03 mmol, 10 mol%) in degassed dichloromethane (6 mL). The test-tube was capped and the reaction mixture was irradiated with LED light (λ = 400 nm) while stirring for 12 h. The reaction mixture was then quenched with saturated solution of sodium hydrogen carbonate (3 mL) and extracted with ethyl acetate (3 × 10 mL). The organic layer was collected, dried over anhydrous sodium sulfate, concentrated under reduced pressure, and the remaining material was purified by flash chromatography on silica gel (hexane/ethyl acetate 2 : 3 v/v) to give the sulfonamide product **5a** (52 mg, 50%) as a white solid.

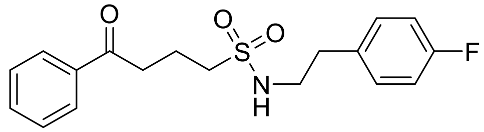

m.p.: 44–46°C. – ^{1}H NMR (500 MHz, $CDCl_3$): 7.97–7.89 (2 H, m), 7.61–7.52 (1 H, m), 7.46 (2 H, dd, *J* = 8.4, 7.2 Hz), 7.22–7.13 (2 H, m), 7.02–6.93 (2 H, m), 4.53 (1 H, t, *J* = 6.3 Hz), 3.38 (2 H, q, *J* = 6.8 Hz), 3.15 (2 H, t, *J* = 6.7 Hz), 3.11–3.01 (2 H, m), 2.86 (2 H, t, *J* = 7.0 Hz), 2.17 (2 H, dq, *J* = 8.8, 6.8 Hz) ppm. – ^{13}C NMR (125 MHz, $CDCl_3$): 198.8, 161.9 (d, *J* = 244.9 Hz), 136.6, 133.7 (d, *J* = 3.3 Hz), 133.5, 130.5 (d, *J* = 8.1 Hz), 128.8, 128.1, 115.7 (d, *J* = 21.4 Hz), 51.7, 44.6, 36.3, 36.0, 18.3 ppm. – ^{19}F NMR (470 MHz, $CDCl_3$): -116.04 (tt, *J* = 9.2, 5.3 Hz). – IR: 3233, 2937, 1672, 1598, 1510, 1449, 1374, 1313, 1247, 1226, 1149, 1139, 1065 cm^{-1}. – HRMS: calcd for $C_{18}H_{20}FNO_3SNa$: 372.1040, found 372.1041 [M+Na^{+}].

Go back to table of contents

Evaluation Only. Created with Aspose.Pdf. Copyright 2002-2014 Aspose Pty Ltd.

N-(4-Fluorophenethyl)heptane-4-sulfonamide (5b)

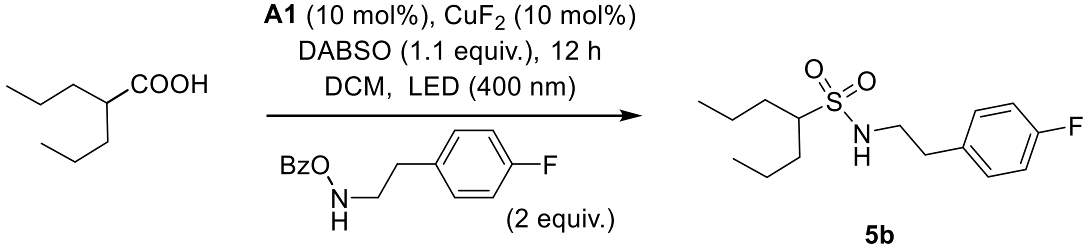

According to **GP1**, the reaction was carried out with 2-propylpentanoic acid (43 mg, 0.3 mmol), DABSO (79 mg, 0.33 mmol, 1.1 equiv.), *O*-benzoyl-*N*-(4-fluorophenethyl)hydroxylamine (155 mg, 0.6 mmol, 2 equiv.), acridine catalyst **A1** (9 mg, 0.03 mmol, 10 mol%), copper difluoride (3 mg, 0.03 mmol, 10 mol%) in degassed dichloromethane (6 mL). The test-tube was capped and the reaction mixture was irradiated with LED light (λ = 400 nm) while stirring for 12 h. The reaction mixture was then quenched with saturated solution of sodium hydrogen carbonate (3 mL) and extracted with ethyl acetate (3 × 10 mL). The organic layer was collected, dried over anhydrous sodium sulfate, concentrated under reduced pressure, and the remaining material was purified by flash chromatography on silica gel (hexane/ethyl acetate 7 : 3 v/v) to give the sulfonamide product **5b** (61 mg, 67%) as a yellow liquid.

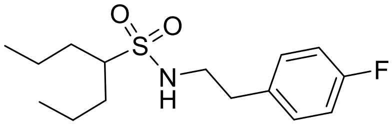

^{1}H NMR (500 MHz, $CDCl_3$): 7.21–7.12 (2 H, m), 7.05–6.95 (2 H, m), 4.25 (1 H, t, *J* = 6.3 Hz), 3.34 (2 H, q, *J* = 6.8 Hz), 2.91–2.73 (3 H, m), 1.77 (2 H, dddd, *J* = 14.0, 10.6, 5.8, 4.9 Hz), 1.61–1.31 (6 H, m), 0.91 (6 H, t, *J* = 7.3 Hz) ppm. – ^{13}C NMR (125 MHz, $CDCl_3$): 161.9 (d, *J* = 245.1 Hz), 133.8 (d, *J* = 3.5 Hz), 130.4 (d, *J* = 7.9 Hz), 115.7 (d, *J* = 21.4 Hz), 62.2, 45.0, 36.3, 31.1, 20.2, 14.2 ppm. – ^{19}F NMR (470 MHz, $CDCl_3$): -116.10 (td, *J* = 8.6, 4.4 Hz). – IR: 3025, 2962, 2865, 1737, 1510, 1420, 1365, 1318, 1264, 1223, 1136, 1081, 895 cm^{-1}. – HRMS: calcd for $C_{15}H_{25}FNO_2S$: 302.1585, found 302.1589 [M+H$^+$].

Go back to table of contents

Evaluation Only. Created with Aspose.Pdf. Copyright 2002-2014 Aspose Pty Ltd.

4,4-Difluoro-*N*-(4-fluorophenethyl)cyclohexane-1-sulfonamide (5c)

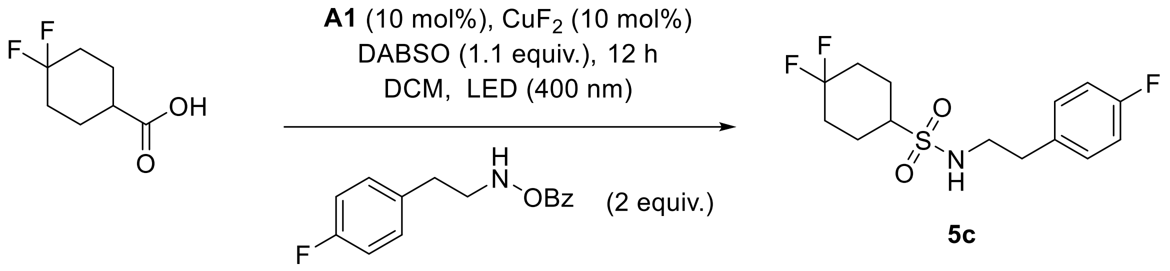

According to **GP1**, the reaction was carried out with 4,4-difluorocyclohexane-1-carboxylic acid (49 mg, 0.3 mmol), DABSO (79 mg, 0.33 mmol, 1.1 equiv.), *O*-benzoyl-*N*-(4-fluorophenethyl)hydroxylamine (156 mg, 0.6 mmol, 2 equiv.), acridine catalyst **A1** (9 mg, 0.03 mmol, 10 mol%), copper difluoride (3 mg, 0.03 mmol, 10 mol%), and degassed dichloromethane (6 mL) were added. The test-tube was capped and the reaction mixture was irradiated with LED light (λ = 400 nm) while stirring for 12 h. The reaction mixture was then quenched with saturated solution of sodium hydrogen carbonate (3 mL) and extracted with ethyl acetate (3 × 10 mL). The organic layer was collected, dried over anhydrous sodium sulfate, concentrated under reduced pressure, and the remaining material was purified by flash chromatography on silica gel (hexane/ ethyl acetate 3 : 1 v/v) to give the sulfone product **5c** (70 mg, 73%) as a colorless solid.

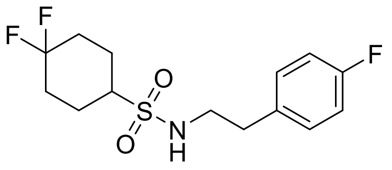

m.p.: 45–47 °C. – ^{1}H NMR (500 MHz, $CDCl_3$): 7.22–7.14 (2 H, m), 7.05–6.95 (2 H, m), 4.39 (1 H, t, *J* = 6.3 Hz), 3.37 (2 H, q, *J* = 6.8 Hz), 2.90–2.74 (3 H, m), 2.20 (2 H, tdd, *J* = 10.8, 5.6, 3.0 Hz), 2.15–2.07 (2 H, m), 1.89–1.77 (2 H, m), 1.70 (2 H, dtt, *J* = 31.2, 13.4, 3.9 Hz) ppm. – ^{13}C NMR (125 MHz, $CDCl_3$) 162.0 (d, $^{1}J_{C\text{-}F}$ = 245.3 Hz), 133.6 (d, $^{4}J_{C\text{-}F}$ = 3.5 Hz), 130.5 (d, $^{3}J_{C\text{-}F}$ = 8.1 Hz), 122.0 (dd, $^{1}J_{C\text{-}F}$ = 242.9, 240.9 Hz), 115.7 (d, $^{2}J_{C\text{-}F}$ = 21.4 Hz), 59.0, 45.1, 36.4, 32.3 (t, $^{2}J_{C\text{-}F}$ = 25.1 Hz), 23.2 (d, $^{3}J_{C\text{-}F}$ = 9.6 Hz) ppm. – ^{19}F NMR (470 MHz, $CDCl_3$): –94.40 (d, *J* = 239.8 Hz), –102.00 (d, *J* = 238.7 Hz), –115.71 (q, *J* = 7.9, 7.2 Hz) ppm. – IR: 3281, 3089, 2967, 2934, 2857, 1601, 1509, 1452, 1311, 1269, 1221, 1141, 1081, 894, 834 cm^{-1}. – HRMS: calcd for $C_{14}H_{18}F_3NNaO_2S$: 344.0903, found 344.0900 [M+Na^{+}].

Go back to table of contents

Evaluation Only. Created with Aspose.Pdf. Copyright 2002-2014 Aspose Pty Ltd.

N-(4-Fluorophenethyl)adamantane-1-sulfonamide (5d)

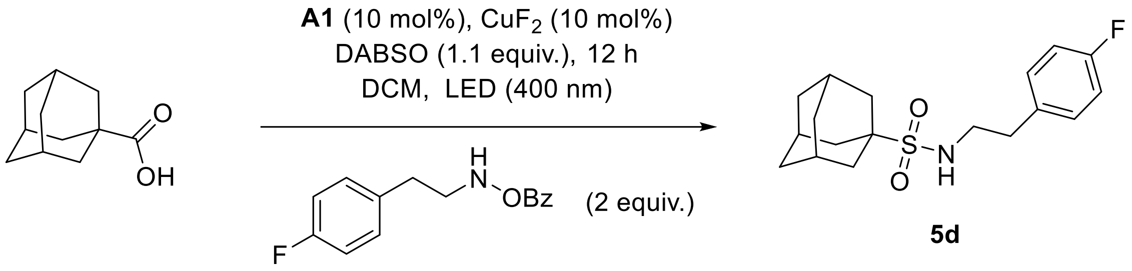

According to **GP1**, the reaction was carried out with adamantane-1-carboxylic acid (54 mg, 0.3 mmol), DABSO (79 mg, 0.33 mmol, 1.1 equiv.), *O*-benzoyl-*N*-(4-fluorophenethyl)hydroxylamine (156 mg, 0.6 mmol, 2 equiv.), acridine catalyst **A1** (9 mg, 0.03 mmol, 10 mol%), copper difluoride (3 mg, 0.03 mmol, 10 mol%), and degassed dichloromethane (6 mL) were added. The test-tube was capped and the reaction mixture was irradiated with LED light (λ = 400 nm) while stirring for 12 h. The reaction mixture was then quenched with saturated solution of sodium hydrogen carbonate (3 mL) and extracted with ethyl acetate (3 × 10 mL). The organic layer was collected, dried over anhydrous sodium sulfate, concentrated under reduced pressure, and the remaining material was purified by flash chromatography on silica gel (hexane/ ethyl acetate 3 : 2 v/v) to give the sulfone product **5d** (53 mg, 52%) as a colorless solid.

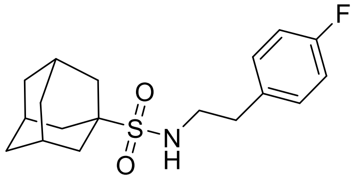

m.p.: 65–67 °C. – ^{1}H NMR (500 MHz, $CDCl_3$): 7.20–7.13 (2 H, m), 7.03–6.96 (2 H, m), 4.06 (1 H, t, *J* = 6.2 Hz), 3.37 (2 H, q, *J* = 6.8 Hz), 2.83 (2 H, t, *J* = 7.0 Hz), 2.13–2.07 (3 H, m), 1.94 (6 H, s), 1.70 (3 H, dt, *J* = 12.6, 2.9 Hz), 1.62 (3 H, dq, *J* = 12.7, 2.1 Hz) ppm. – ^{13}C NMR (125 MHz, $CDCl_3$): 161.7 (d, $^1J_{C\text{-}F}$ = 245.0 Hz), 134.0 (d, $^4J_{C\text{-}F}$ = 3.5 Hz), 130.5 (d, $^3J_{C\text{-}F}$ = 8.0 Hz), 115.6 (d, $J_{C\text{-}F}$ = 21.4 Hz), 61.2, 46.1, 37.0, 35.9, 35.8, 28.2 ppm. – ^{19}F NMR (470 MHz, $CDCl_3$): -116.13– -116.26 (m) ppm. – IR: 3271, 2910, 2851, 1598, 1508, 1440, 1417, 1375, 1287, 1215, 1165, 1142, 1102, 1080, 1052, 976, 909, 820, 796 cm^{-1}. – HRMS: calcd for $C_{18}H_{24}FNNaO_2S$: 360.1404, found 360.1403 [M+Na$^+$].

Go back to table of contents

Evaluation Only. Created with Aspose.Pdf. Copyright 2002-2014 Aspose Pty Ltd.

5-(2,5-Dimethylphenoxy)-*N*-(4-fluorophenethyl)-2-methylpentane-2-sulfonamide (5e)

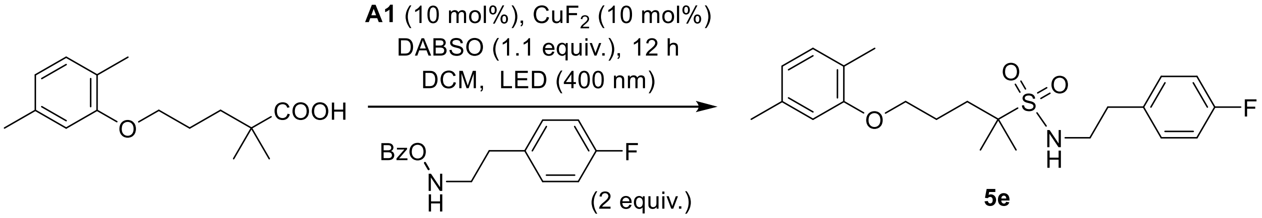

According to **GP1**, the reaction was carried out with 5-(2,5-dimethylphenoxy)-2,2-dimethylpentanoic acid (75 mg, 0.3 mmol), DABSO (79 mg, 0.33 mmol, 1.1 equiv.), *O*-benzoyl-*N*-(4-fluorophenethyl)hydroxylamine (155 mg, 0.6 mmol, 2 equiv.), acridine catalyst **A1** (9 mg, 0.03 mmol, 10 mol%), copper difluoride (3 mg, 0.03 mmol, 10 mol%) in degassed dichloromethane (6 mL). The test-tube was capped and the reaction mixture was irradiated with LED light (λ = 400 nm) while stirring for 12 h. The reaction mixture was then quenched with saturated solution of sodium hydrogen carbonate (3 mL) and extracted with ethyl acetate (3 × 10 mL). The organic layer was collected, dried over anhydrous sodium sulfate, concentrated under reduced pressure, and the remaining material was purified by flash chromatography on silica gel (hexane/ethyl acetate 2 : 3 v/v) to give the sulfonamide product **5e** (75 mg, 61%) as a white solid.

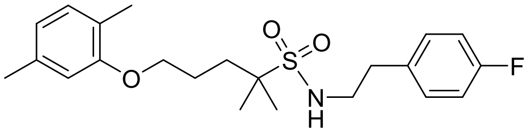

m.p.: 40–41°C. – ^{1}H NMR (500 MHz, $CDCl_3$): 7.20–7.13 (2 H, m), 7.04–6.96 (3 H, m), 6.70–6.64 (1 H, m), 6.61 (1 H, d, *J* = 1.5 Hz), 3.94 (2 H, t, *J* = 5.6 Hz), 3.86 (1 H, t, *J* = 6.3 Hz), 3.41 (2 H, q, *J* = 6.7 Hz), 2.85 (2 H, t, *J* = 6.9 Hz), 2.31 (3 H, s), 2.16 (3 H, s), 1.97–1.81 (4 H, m), 1.35 (6 H, s) ppm. – ^{13}C NMR (125 MHz, $CDCl_3$): 162.0 (d, *J* = 245.2 Hz), 156.9, 136.7, 133.7, 130.5 (d, *J* = 3.4 Hz), 130.5, 123.6, 121.1, 115.8 (d, *J* = 21.1 Hz), 112.1, 67.8, 63.0, 46.2, 37.0, 33.0, 24.5, 21.7, 21.5, 15.9 ppm. – ^{19}F NMR (470 MHz, $CDCl_3$): -116.05 (td, *J* = 8.6, 4.3 Hz) ppm. – IR: 2956, 2924, 2873, 2859, 2216, 2062, 1462, 1381 cm^{-1}. – HRMS: calcd for $C_{22}H_{30}FNO_3SNa$: 430.1823, found 430.1801 [M+Na^{+}].

Evaluation Only. Created with Aspose.Pdf. Copyright 2002-2014 Aspose Pty Ltd.

Methyl (1*R**,2*S**)-2-(*N*-((*R*)-1-phenylethyl)sulfamoyl)cyclohexane-1-carboxylate (6a)

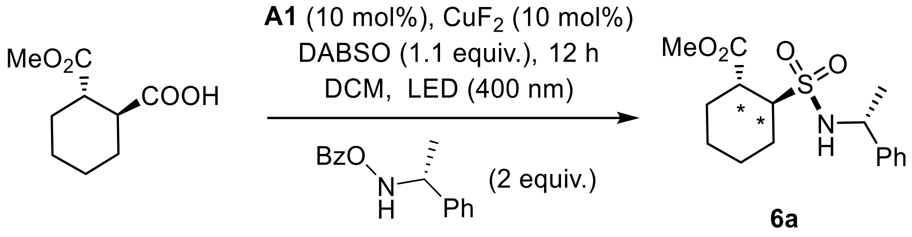

According to **GP1**, the reaction was carried out with (1*S*,2*S*)-2-(methoxycarbonyl)cyclohexane-1-carboxylic acid (56 mg, 0.3 mmol), DABSO (79 mg, 0.33 mmol, 1.1 equiv.), (*R*)-*O*-benzoyl-*N*-(1-phenylethyl)hydroxylamine (145 mg, 0.6 mmol, 2 equiv.), acridine catalyst **A1** (9 mg, 0.03 mmol, 10 mol%), copper difluoride (3 mg, 0.03 mmol, 10 mol%) in degassed dichloromethane (6 mL). The test-tube was capped and the reaction mixture was irradiated with LED light (λ = 400 nm) while stirring for 12 h. The reaction mixture was then quenched with saturated solution of sodium hydrogen carbonate (3 mL) and extracted with ethyl acetate (3 × 10 mL). The organic layer was collected, dried over anhydrous sodium sulfate, concentrated under reduced pressure, and the remaining material was purified by flash chromatography on silica gel (hexane/ethyl acetate 3 : 2 v/v) to give the inseparable diasisomer sulfonamide products **6a** (85 mg, 87%, 1 : 1 ratio) as a yellow liquid.

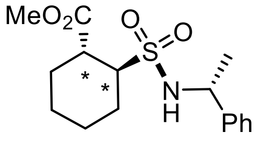

$[\alpha]_D^{23}$ = -100 (*c* 0.25, chloroform). –^{1}H NMR (500 MHz, $CDCl_3$): 7.40–7.31 (4 H, m), 7.31–7.24 (1 H, m), 4.92 (1 H, t, *J* = 8.4 Hz), 4.76–4.52 (1 H, m), 3.67 (3 H, d, *J* = 18.1 Hz), 3.03 (1 H, dddd, *J* = 100.2, 11.9, 10.8, 4.1 Hz), 2.71–2.42 (1 H, m), 2.15–1.58 (4 H, m), 1.55 (3 H, dd, *J* = 6.9, 5.6 Hz), 1.51–1.30 (2 H, m), 1.22–0.90 (2 H, m) ppm. – ^{13}C NMR (125 MHz, $CDCl_3$): 175.1, 174.8, 143.0, 128.9, 128.9, 128.9, 127.9, 127.8, 126.4, 126.3, 126.3, 62.8, 62.0, 54.1, 53.9, 52.2, 44.0, 43.6, 29.8, 29.8, 25.8, 25.5, 24.5, 24.4, 24.3, 24.2, 24.1 ppm. – IR: 2935, 2870, 1738, 1452, 1318, 1254, 1169, 1091, 1042, 880 cm^{-1}. – HRMS: calcd for $C_{16}H_{24}NO_4S$: 326.1426, found 326.1426 [M+H$^+$].

Go back to table of contents

Evaluation Only. Created with Aspose.Pdf. Copyright 2002-2014 Aspose Pty Ltd.

N-((*R*)-1-Phenylethyl)adamantane-1-sulfonamide (6b)

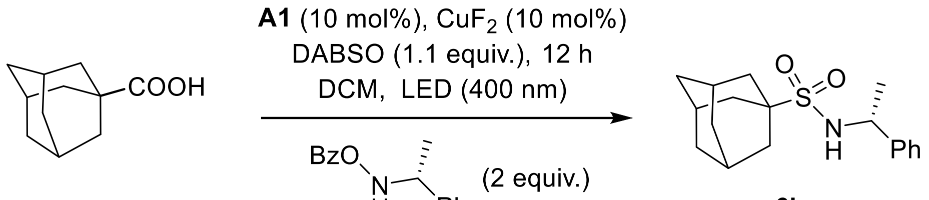

6b

According to **GP1**, the reaction was carried out with adamantane-1-carboxylic acid (54 mg, 0.3 mmol), DABSO (79 mg, 0.33 mmol, 1.1 equiv.), (*R*)-*O*-benzoyl-*N*-(1-phenylethyl)hydroxylamine (145 mg, 0.6 mmol, 2 equiv.), acridine catalyst **A1** (9 mg, 0.03 mmol, 10 mol%), copper difluoride (3 mg, 0.03 mmol, 10 mol%) in degassed dichloromethane (6 mL). The test-tube was capped and the reaction mixture was irradiated with LED light (λ = 400 nm) while stirring for 12 h. The reaction mixture was then quenched with saturated solution of sodium hydrogen carbonate (3 mL) and extracted with ethyl acetate (3 × 10 mL). The organic layer was collected, dried over anhydrous sodium sulfate, concentrated under reduced pressure, and the remaining material was purified by flash chromatography on silica gel (hexane/ethyl acetate 2 : 3 v/v) to give the sulfonamide product **6b** (81 mg, 85%) as a yellow solid.

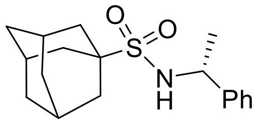

m.p.: 78–80°C. – ^{1}H NMR (500 MHz, $CDCl_3$): 7.44–7.32 (4 H, m), 7.31–7.25 (1 H, m), 4.65 (1 H, dq, *J* = 9.0, 6.9 Hz), 4.38 (1 H, d, *J* = 8.9 Hz), 2.09 (3 H, p, *J* = 3.2 Hz), 2.01–1.89 (6 H, m), 1.69 (3 H, d, *J* = 12.8 Hz), 1.64–1.56 (6 H, m) ppm. – ^{13}C NMR (125 MHz, $CDCl_3$): 143.7, 128.7, 127.4, 125.9, 60.9, 54.2, 35.8, 35.6, 28.1, 25.5 ppm. – IR: 3028, 2971, 2907, 2850, 2678, 1603, 1453, 1433, 1298, 1147 cm^{-1}. – HRMS: calcd for $C_{18}H_{25}NO_2SNa$: 342.1498, found 342.1497 [$M+Na^+$].

Go back to table of contents

Evaluation Only. Created with Aspose.Pdf. Copyright 2002-2014 Aspose Pty Ltd.

N,*N*-Dibenzyl-4-(5-methylthiophen-2-yl)-4-oxobutane-1-sulfonamide (7a)

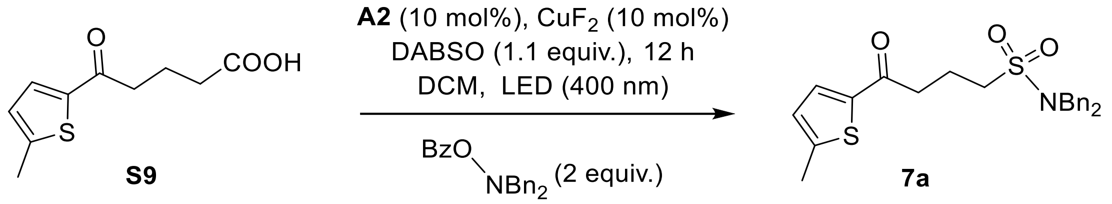

According to **GP1**, the reaction was carried out with acid **S9** (64 mg, 0.3 mmol), DABSO (79 mg, 0.33 mmol, 1.1 equiv.), *O*-benzoyl-*N*,*N*-dibenzylhydroxylamine (190 mg, 0.6 mmol, 2 equiv.), acridine catalyst **A1** (9 mg, 0.03 mmol, 10 mol%), copper difluoride (3 mg, 0.03 mmol, 10 mol%) in degassed dichloromethane (6 mL). The test-tube was capped and the reaction mixture was irradiated with LED light (λ = 400 nm) while stirring for 12 h. The reaction mixture was then quenched with saturated solution of sodium hydrogen carbonate (3 mL) and extracted with ethyl acetate (3 × 10 mL). The organic layer was collected, dried over anhydrous sodium sulfate, concentrated under reduced pressure, and the remaining material was purified by flash chromatography on silica gel (hexane/ethyl acetate 2 : 3 v/v) to give the sulfonamide product **7a** (77 mg, 60%) as a white solid.

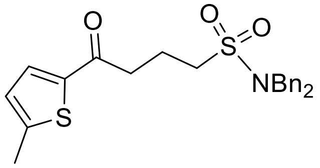

m.p.: 73–75°C. – ^{1}H NMR (500 MHz, $CDCl_3$): 7.56 (1 H, d, *J* = 3.7 Hz), 7.41–7.27 (10 H, m), 6.82 (1 H, dd, *J* = 3.8, 1.2 Hz), 4.37 (4 H, s), 3.04 (4 H, q, *J* = 7.3 Hz), 2.55 (3 H, d, *J* = 1.0 Hz), 2.32–2.15 (2 H, m) ppm. – ^{13}C NMR (125 MHz, $CDCl_3$): 191.3, 150.1, 141.7, 135.8, 132.9, 130.3, 129.3, 129.1, 128.8, 128.7, 128.6, 128.0, 127.0, 52.5, 50.3, 36.5, 18.6, 16.1 ppm. – IR: 3062, 2920, 2784, 2737, 2597, 1655, 1606, 1570, 1516, 1494, 1455, 1320, 1142 cm^{-1}. – HRMS: calcd for $C_{23}H_{25}NO_3S_2Na$: 450.1168, found 450.1163 [$M+Na^+$].

Evaluation Only. Created with Aspose.Pdf. Copyright 2002-2014 Aspose Pty Ltd.

N,*N*-Dibenzylcyclohexanesulfonamide (7b)

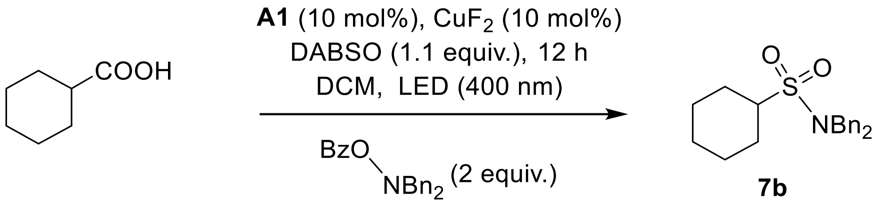

According to **GP1**, the reaction was carried out with cyclohexanecarboxylic acid (38 mg, 0.3 mmol), DABSO (79 mg, 0.33 mmol, 1.1 equiv.), *O*-benzoyl-*N*,*N*-dibenzylhydroxylamine (190 mg, 0.6 mmol, 2 equiv.), acridine catalyst **A1** (9 mg, 0.03 mmol, 10 mol%), copper difluoride (3 mg, 0.03 mmol, 10 mol%) in degassed dichloromethane (6 mL). The test-tube was capped and the reaction mixture was irradiated with LED light (λ = 400 nm) while stirring for 12 h. The reaction mixture was then quenched with saturated solution of sodium hydrogen carbonate (3 mL) and extracted with ethyl acetate (3 × 10 mL). The organic layer was collected, dried over anhydrous sodium sulfate, concentrated under reduced pressure, and the remaining material was purified by flash chromatography on silica gel (hexane/ethyl acetate 2 : 3 v/v) to give the sulfonamide product **7b** (69 mg, 67%) as a white solid.

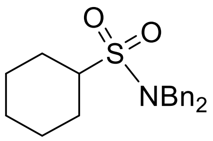

m.p.: 85–87°C. – ^{1}H NMR (500 MHz, $CDCl_3$): 7.49–7.08 (10 H, m), 4.36 (4 H, s), 2.78 (1 H, tt, *J* = 12.1, 3.4 Hz), 2.08 (2 H, dd, *J* = 12.4, 3.2 Hz), 1.85 (2 H, dt, *J* = 13.6, 3.7 Hz), 1.70–1.45 (3 H, m), 1.23 – 1.07 (3 H, m) ppm. – ^{13}C NMR (125 MHz, $CDCl_3$): 136.3, 128.8, 128.0, 63.1, 50.9, 26.7, 25.4, 25.3 ppm. – IR: 3029, 2936, 2854, 2616, 1711, 1602, 1583, 1494, 1452, 1379, 1265, 1228, 1142, 1053 cm^{-1}. – HRMS: calcd for $C_{20}H_{26}NO_2S$: 344.1684, found 344.1683 [M+H^{+}].

Evaluation Only. Created with Aspose.Pdf. Copyright 2002-2014 Aspose Pty Ltd.

N,*N*-Dibenzyltetrahydro-2*H*-pyran-4-sulfonamide (7c)

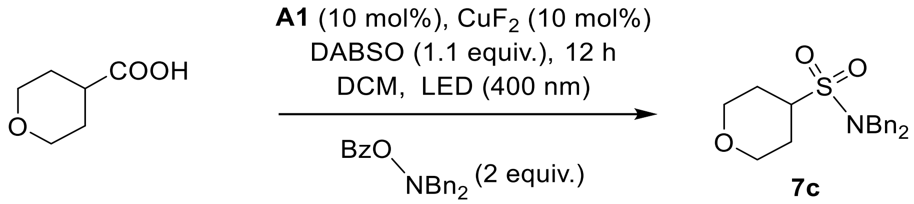

According to **GP1**, the reaction was carried out with tetrahydro-2*H*-pyran-4-carboxylic acid (39 mg, 0.3 mmol), DABSO (79 mg, 0.33 mmol, 1.1 equiv.), *O*-benzoyl-*N*,*N*-dibenzylhydroxylamine (190 mg, 0.6 mmol, 2 equiv.), acridine catalyst **A1** (9 mg, 0.03 mmol, 10 mol%), copper difluoride (3 mg, 0.03 mmol, 10 mol%) in degassed dichloromethane (6 mL). The test-tube was capped and the reaction mixture was irradiated with LED light (λ = 400 nm) while stirring for 12 h. The reaction mixture was then quenched with saturated solution of sodium hydrogen carbonate (3 mL) and extracted with ethyl acetate (3 × 10 mL). The organic layer was collected, dried over anhydrous sodium sulfate, concentrated under reduced pressure, and the remaining material was purified by flash chromatography on silica gel (hexane/ethyl acetate 2 : 3 v/v) to give the sulfonamide product **7c** (59 mg, 57%) as a white solid.

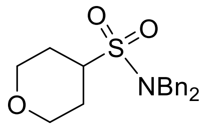

m.p.: 89–90°C. – ^{1}H NMR (500 MHz, $CDCl_3$): 7.43–7.24 (10 H, m), 4.40 (4 H, s), 4.06 (2 H, ddd, *J* = 11.8, 4.5, 1.9 Hz), 3.24 (2 H, td, *J* = 11.6, 2.5 Hz), 2.96 (1 H, tt, *J* = 11.6, 4.2 Hz), 2.02–1.83 (4 H, m) ppm. – ^{13}C NMR (125 MHz, $CDCl_3$): 136.1, 128.9, 128.8, 128.2, 66.8, 60.2, 51.0, 26.9 ppm. – IR: 3053, 2951, 2310, 2174, 1468, 1426, 1366, 1264, 1123, 894 cm^{-1}. – HRMS: calcd for $C_{19}H_{24}NO_3S$: 346.1477, found 346.1475 [$M+H^+$].

Go back to table of contents

Evaluation Only. Created with Aspose.Pdf. Copyright 2002-2014 Aspose Pty Ltd.

N- (*p*-Tolyl)cyclohexanesulfonamide (8a)

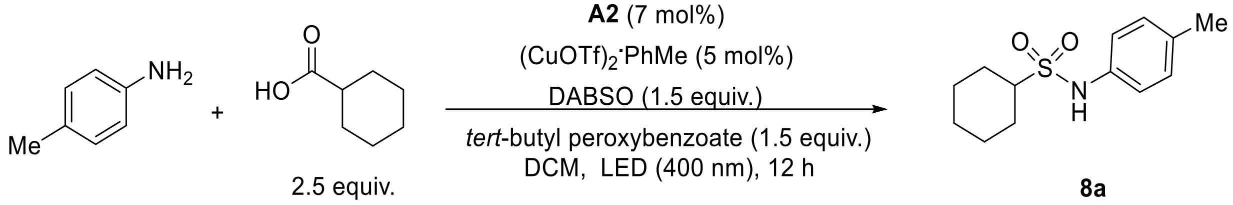

According to **GP2**, the reaction was carried out with *p*-toluidine (32 mg, 0.3 mmol), cyclohexanecarboxylic acid (96 mg, 0.75 mmol, 2.5 equiv.), DABSO (108 mg, 0.45 mmol, 1.5 equiv.), acridine **A2** (6 mg, 0.02 mmol, 7 mol%), *tert*-butyl peroxybenzoate (87 mg, 0.45 mmol, 1.5 equiv.), $(CuOTf)_2$•PhMe (7 mg, 0.015 mmol, 5 mol%) in degassed dichloromethane (3 mL). The test-tube was capped and the reaction mixture was irradiated with LED light (λ = 400 nm) while stirring for 12 h. The reaction mixture was then quenched with saturated solution of sodium hydrogen carbonate (3 mL), extracted with ethyl acetate (3 × 10 mL). The organic layer was collected, dried over anhydrous sodium sulfate, concentrated under reduced pressure, and the remaining material was purified by flash chromatography on silica gel (hexane/ ethyl acetate 3 : 2 v/v) to give the sulfonamide **8a** (60 mg, 79%) as a yellow oil.

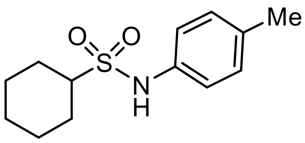

^{1}H NMR (500 MHz, $CDCl_3$): 7.03 (2 H, dd, *J* = 7.0, 5.3 Hz), 6.97–6.91 (2 H, m), 2.90 (1 H, tt, *J* = 11.3, 3.8 Hz), 2.29 (3 H, s), 2.20–2.12 (1 H, m), 2.10–2.00 (1 H, m), 1.94–1.79 (2 H, m), 1.73–1.62 (1 H, m), 1.59–1.21 (5 H, m) ppm. – ^{13}C NMR (125 MHz, $CDCl_3$): 139.4, 132.3, 129.9, 118.6, 62.7, 26.6, 26.4, 25.6, 25.2, 25.1, 20.7 ppm. – IR: 3455, 3015, 2969, 2945, 2887, 1783, 1693, 1517, 1424, 1366, 1228, 1216, 1018, 908, 732 cm^{-1}. – HRMS: calcd for $C_{13}H_{20}NO_2S$: 254.1209, found 254.1203 [M+H$^+$].

Go back to table of contents

Evaluation Only. Created with Aspose.Pdf. Copyright 2002-2014 Aspose Pty Ltd.

N-(3,5-Dimethylphenyl)cyclohexanesulfonamide (8b)

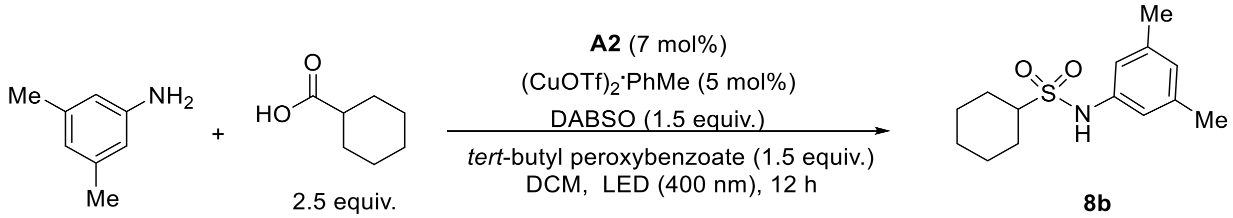

8b

According to **GP2**, the reaction was carried out with 3,5-dimethylaniline (36 mg, 0.3 mmol), cyclohexanecarboxylic acid (96 mg, 0.75 mmol, 2.5 equiv.), DABSO (108 mg, 0.45 mmol, 1.5 equiv.), acridine **A2** (6 mg, 0.02 mmol, 7 mol%), *tert*-butyl peroxybenzoate (87 mg, 0.45 mmol, 1.5 equiv.), $(CuOTf)_2$•PhMe (7 mg, 0.015 mmol, 5 mol%) in degassed dichloromethane (3 mL). The test-tube was capped and the reaction mixture was irradiated with LED light (λ = 400 nm) while stirring for 12 h. The reaction mixture was then quenched with saturated solution of sodium hydrogen carbonate (3 mL), extracted with ethyl acetate (3 × 10 mL). The organic layer was collected, dried over anhydrous sodium sulfate, concentrated under reduced pressure, and the remaining material was purified by flash chromatography on silica gel (hexane/ ethyl acetate 3 : 2 v/v) to give the sulfonamide **8b** (65 mg, 81%) as a yellow oil.

Gram scale for compound 13b: According to **GP2**, the reaction was carried out in two parallel test tubes with the same amount of starting materials: 3,5-dimethylaniline (0.61 g, 5 mmol), cyclohexanecarboxylic acid (1.60 g, 12.5 mmol, 2.5 equiv.), DABSO (1.80 g, 7.5 mmol, 1.5 equiv.), acridine **A2** (104 mg, 0.35 mmol, 7 mol%), *tert*-butyl peroxybenzoate (1.46 g, 7.5 mmol, 1.5 equiv.), $(CuOTf)_2$•PhMe (129.3 mg, 0.25 mmol, 5 mol%) in degassed dichloromethane (30 mL). The test-tube was capped and the reaction mixture was irradiated with LED light (λ = 400 nm) while stirring for 16 h. The reaction mixtures were combined and then quenched with saturated solution of sodium hydrogen carbonate (30 mL), extracted with ethyl acetate (3 × 50 mL). The organic layer was collected, dried over anhydrous sodium sulfate, concentrated under reduced pressure, and the remaining

Evaluation Only. Created with Aspose.Pdf. Copyright 2002-2014 Aspose Pty Ltd.

material was purified by flash chromatography on silica gel (hexane/ ethyl acetate 3 : 2 v/v) to give the sulfonamide **8b** (1.73 g, 65%) as a yellow oil.

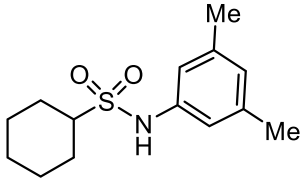

^{1}H NMR (500 MHz, $CDCl_3$): 6.97–6.84 (1 H, m), 6.70–6.61 (3 H, m), 2.90 (1 H, ddt, *J* = 14.8, 11.2, 3.5 Hz), 2.34–2.16 (7H, m), 2.09–2.01 (1 H, m), 1.98–1.75 (2 H, m), 1.74–1.62 (1 H, m), 1.59–1.21 (5 H, m) ppm. – ^{13}C NMR (125 MHz, $CDCl_3$): 141.9, 139.2, 124.6, 115.9, 62.8, 26.6, 26.4, 25.6, 25.3, 25.2, 21.4 ppm. – IR: 3455, 3015, 2969, 2937, 2855, 1738, 1601, 1448, 1365, 1228, 1216, 1157, 1047, 895, 734 cm^{-1}. – HRMS: calcd for $C_{14}H_{22}NO_2S$: 268.1366, found 286.1364 [M+H^{+}].

N-(3,4,5-Trimethylphenyl)cyclohexanesulfonamide (8c)

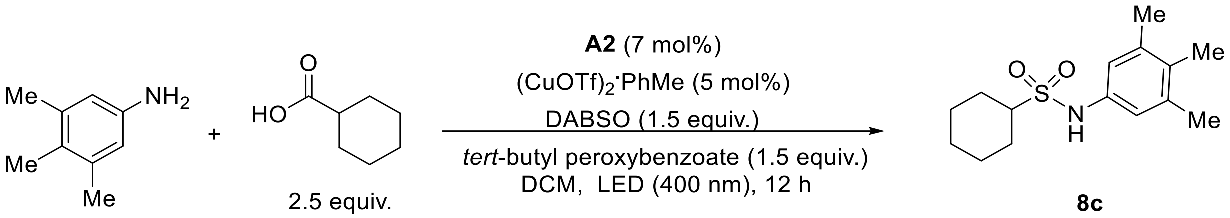

According to **GP2**, the reaction was carried out with 3,4,5-trimethylaniline (41 mg, 0.3 mmol), cyclohexanecarboxylic acid (96 mg, 0.75 mmol, 2.5 equiv.), DABSO (108 mg, 0.45 mmol, 1.5 equiv.), acridine **A2** (6 mg, 0.02 mmol, 7 mol%), *tert*-butyl peroxybenzoate (87 mg, 0.45 mmol, 1.5 equiv.), $(CuOTf)_2$•PhMe (7 mg, 0.015 mmol, 5 mol%) in degassed dichloromethane (3 mL). The test-tube was capped and the reaction mixture was irradiated with LED light (λ = 400 nm) while stirring for 12 h. The reaction mixture was then quenched with saturated solution of sodium hydrogen carbonate (3 mL), extracted with ethyl acetate (3 × 10 mL). The organic layer was collected, dried over anhydrous sodium sulfate, concentrated under reduced pressure, and the remaining material was purified by flash chromatography on silica gel (hexane/ ethyl acetate 3 : 2 v/v) to give the sulfonamide **8c** (58 mg, 69%) as a yellow oil.

Evaluation Only. Created with Aspose.Pdf. Copyright 2002-2014 Aspose Pty Ltd.

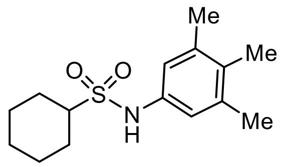

^{1}H NMR (300 MHz, $CDCl_3$): 6.69 (2 H, s), 6.36 (1 H, s), 2.81 (1 H, tt, *J* = 11.2, 3.7 Hz), 2.20–2.02 (11 H, m), 1.97–1.61 (3 H, m), 1.61–1.18 (5 H, m) ppm. – ^{13}C NMR (125 MHz, $CDCl_3$): 138.8, 137.6, 129.9, 118.1, 62.9, 26.5, 26.3, 25.7, 25.3, 25.0, 20.7, 14.8 ppm. – IR: 3454, 3015, 2969, 2928, 2853, 1738, 1485, 1448, 1365, 1227, 1216, 1162, 1047, 995, 894, 767 cm^{-1}. – HRMS: calcd for $C_{15}H_{23}NO_2S$: 281.1450, found 281.1445 [M^+].

N-(4-Methoxyphenyl)cyclohexanesulfonamide (8d)

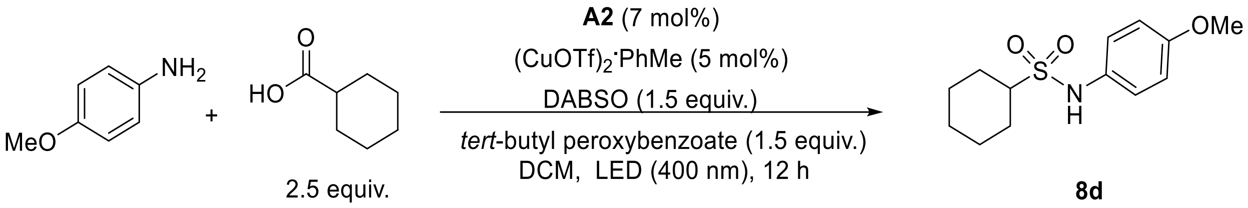

According to **GP2**, the reaction was carried out with 3,4,5-trimethylaniline (41 mg, 0.3 mmol), cyclohexanecarboxylic acid (96 mg, 0.75 mmol, 2.5 equiv.), DABSO (108 mg, 0.45 mmol, 1.5 equiv.), acridine **A2** (6 mg, 0.02 mmol, 7 mol%), *tert*-butyl peroxybenzoate (87 mg, 0.45 mmol, 1.5 equiv.), $(CuOTf)_2$•PhMe (7 mg, 0.015 mmol, 5 mol%) in degassed dichloromethane (3 mL). The test-tube was capped and the reaction mixture was irradiated with LED light (λ = 400 nm) while stirring for 12 h. The reaction mixture was then quenched with saturated solution of sodium hydrogen carbonate (3 mL), extracted with ethyl acetate (3 × 10 mL). The organic layer was collected, dried over anhydrous sodium sulfate, concentrated under reduced pressure, and the remaining material was purified by flash chromatography on silica gel (hexane/ ethyl acetate 3 : 2 v/v) to give the sulfonamide **8d** (53 mg, 66%) as a yellow oil.

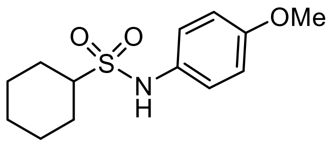

^{1}H NMR (300 MHz, $CDCl_3$): 7.03–6.93 (2 H, m), 6.83–6.73 (2 H, m), 6.38 (1 H, brs), 3.75 (3 H, s), 2.79 (1 H, tt, *J* = 11.4, 3.8 Hz), 2.15–2.02 (2 H, m), 1.93–1.76 (2 H, m), 1.67 (1 H, dd, *J* = 11.2, 4.4

Evaluation Only. Created with Aspose.Pdf. Copyright 2002-2014 Aspose Pty Ltd.

Hz), 1.57–1.20 (5 H, m) ppm. – ^{13}C NMR (125 MHz, $CDCl_3$): 156.3, 134.7, 121.6, 114.7, 62.7, 55.7, 26.4, 26.4, 25.7, 25.3, 25.2 ppm. – IR: 3452, 3009, 2980, 2937, 2867, 1762, 1622, 1504, 1442, 1352, 1214, 1120, 912 cm^{-1}. – HRMS: calcd for $C_{13}H_{19}NO_3S$: 269.1086, found 269.1490 [M+H$^+$].

N-(2-Methoxyphenyl)cyclohexanesulfonamide (8e)

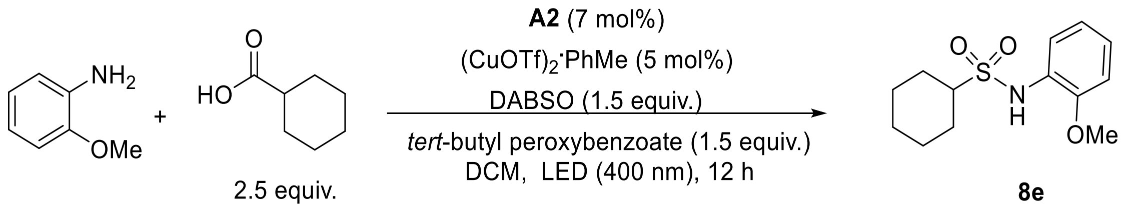

According to **GP2**, the reaction was carried out with 2-methoxyaniline (37 mg, 0.3 mmol), cyclohexanecarboxylic acid (96 mg, 0.75 mmol, 2.5 equiv.), DABSO (108 mg, 0.45 mmol, 1.5 equiv.), acridine **A2** (6 mg, 0.02 mmol, 7 mol%), *tert*-butyl peroxybenzoate (87 mg, 0.45 mmol, 1.5 equiv.), $(CuOTf)_2$•PhMe (7 mg, 0.015 mmol, 5 mol%) in degassed dichloromethane (3 mL). The test-tube was capped and the reaction mixture was irradiated with LED light (λ = 400 nm) while stirring for 12 h. The reaction mixture was then quenched with saturated solution of sodium hydrogen carbonate (3 mL), extracted with ethyl acetate (3 × 10 mL). The organic layer was collected, dried over anhydrous sodium sulfate, concentrated under reduced pressure, and the remaining material was purified by flash chromatography on silica gel (hexane/ ethyl acetate 3 : 2 v/v) to give the sulfonamide **8e** (57 mg, 71%) as a yellow oil.

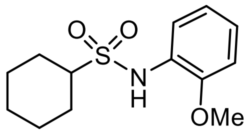

^{1}H NMR (300 MHz, $CDCl_3$): 7.21 (1 H, dd, *J* = 7.6, 1.9 Hz), 7.08–6.77 (3 H, m), 6.28 (1 H, s), 3.83 (3 H, s), 2.77 (1 H, tt, *J* = 11.3, 3.7 Hz), 2.24–2.01 (2 H, m), 1.90 (2 H, tq, *J* = 11.7, 3.5 Hz), 1.78–1.64 (1 H, m), 1.63–1.18 (5 H, m) ppm. – ^{13}C NMR (75 MHz, $CDCl_3$): 148.7, 131.4, 122.8, 121.2, 116.8, 110.9, 63.1, 55.8, 26.0, 25.7, 25.4, 25.2 ppm. – IR: 3377, 2964, 2931, 2854, 2624, 1703, 1601, 1504,

Evaluation Only. Created with Aspose.Pdf. Copyright 2002-2014 Aspose Pty Ltd.

1466, 1453, 1328, 1293, 1266, 1223, 1160, 1128, 1046, 1027, 995, 760 cm^{-1}. – HRMS: calcd for $C_{13}H_{19}NO_3S$: 269.1086, found 269.1082 [M$^+$].

N-(4-(Trifluoromethoxy)phenyl)cyclohexanesulfonamide (8f)

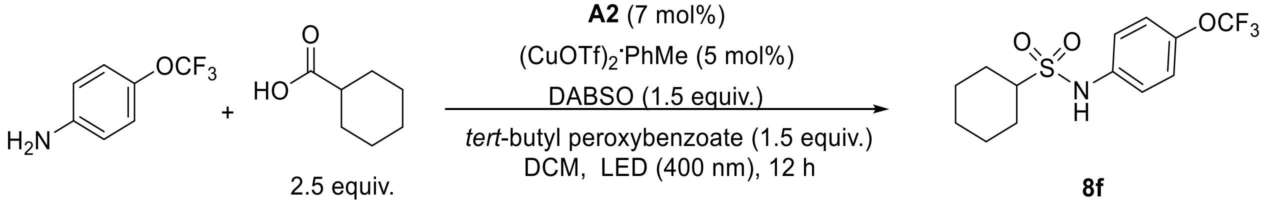

According to **GP2**, the reaction was carried out with 4-(trifluoromethoxy)aniline (53 mg, 0.3 mmol), cyclohexanecarboxylic acid (96 mg, 0.75 mmol, 2.5 equiv.), DABSO (108 mg, 0.45 mmol, 1.5 equiv.), acridine **A2** (6 mg, 0.02 mmol, 7 mol%), *tert*-butyl peroxybenzoate (87 mg, 0.45 mmol, 1.5 equiv.), $(CuOTf)_2$•PhMe (7 mg, 0.015 mmol, 5 mol%) in degassed dichloromethane (3 mL). The test-tube was capped and the reaction mixture was irradiated with LED light (λ = 400 nm) while stirring for 12 h. The reaction mixture was then quenched with saturated solution of sodium hydrogen carbonate (3 mL), extracted with ethyl acetate (3 × 10 mL). The organic layer was collected, dried over anhydrous sodium sulfate, concentrated under reduced pressure, and the remaining material was purified by flash chromatography on silica gel (hexane/ ethyl acetate 3 : 2 v/v) to give the sulfonamide **8f** (52 mg, 54%) as a yellow oil.

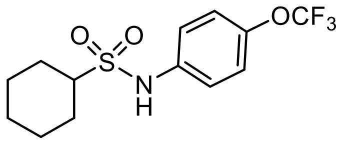

^{1}H NMR (500 MHz, $CDCl_3$): 7.61 (1 H, s), 7.02 (2 H, d, *J* = 8.8 Hz), 7.00–6.95 (2 H, m), 2.93 (1 H, tt, *J* = 11.3, 3.8 Hz), 2.16–2.07 (1 H, m), 2.04–1.96 (1 H, m), 1.92–1.76 (2 H, m), 1.72–1.62 (1 H, m), 1.55–1.45 (1 H, m), 1.44–1.35 (1 H, m), 1.34–1.16 (3 H, m) ppm. – ^{13}C NMR (125 MHz, $CDCl_3$): 144.4, 141.1, 122.3, 120.6 (d, $^{1}J_{C\text{-}F}$ = 256.6 Hz), 118.7, 62.9, 26.7, 26.4, 25.5, 25.2, 25.1 ppm. – ^{19}F NMR (470 MHz, $CDCl_3$): -58.33 (s). – IR: 3129, 3050, 2939, 2857, 1607, 1505, 1447,

Evaluation Only. Created with Aspose.Pdf. Copyright 2002-2014 Aspose Pty Ltd.

1254, 1220, 1159, 1036, 883, 842, 723 cm^{-1}. – HRMS: calcd for $C_{13}H_{17}F_3NO_3S$: 324.0876, found 324.0855 [M+H$^+$].

N-(3-(Methylthio)phenyl)cyclohexanesulfonamide (8g)

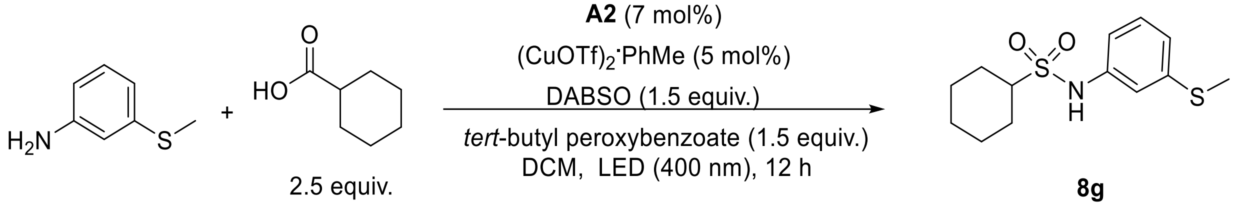

According to **GP2**, the reaction was carried out with 3-(methylthio)aniline (42 mg, 0.3 mmol), cyclohexanecarboxylic acid (96 mg, 0.75 mmol, 2.5 equiv.), DABSO (108 mg, 0.45 mmol, 1.5 equiv.), acridine **A2** (6 mg, 0.02 mmol, 7 mol%), *tert*-butyl peroxybenzoate (87 mg, 0.45 mmol, 1.5 equiv.), $(CuOTf)_2$•PhMe (7 mg, 0.015 mmol, 5 mol%) in degassed dichloromethane (3 mL). The test-tube was capped and the reaction mixture was irradiated with LED light (λ = 400 nm) while stirring for 12 h. The reaction mixture was then quenched with saturated solution of sodium hydrogen carbonate (3 mL), extracted with ethyl acetate (3 × 10 mL). The organic layer was collected, dried over anhydrous sodium sulfate, concentrated under reduced pressure, and the remaining material was purified by flash chromatography on silica gel (hexane/ ethyl acetate 3 : 2 v/v) to give the sulfonamide **8g** (51 mg, 60%) as a yellow oil.

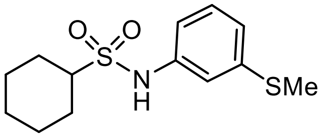

^{1}H NMR (300 MHz, $CDCl_3$): 7.11 (1 H, t, *J* = 7.9 Hz), 7.00 (1 H, s), 6.90–6.80 (2 H, m), 6.77 (1 H, ddd, *J* = 8.0, 2.2, 0.9 Hz), 2.87 (1 H, tt, *J* = 11.2, 3.7 Hz), 2.41 (3 H, s), 2.15 (1 H, d, *J* = 14.0 Hz), 2.07–1.97 (1 H, m), 1.85 (2 H, ddd, *J* = 15.7, 6.4, 3.2 Hz), 1.58–1.14 (6 H, m) ppm. – ^{13}C NMR (75 MHz, $CDCl_3$): 142.7, 140.1, 129.7, 120.7, 115.6, 114.5, 63.0, 26.6, 26.3, 25.6, 25.25, 25.15, 15.6 ppm. – IR: 3061, 2995, 2919, 2857, 1709, 1592, 1499, 1382, 1266, 1222, 1149, 1046, 911, 859, 734, 703 cm^{-1}. – HRMS: calcd for $C_{13}H_{20}NO_2S_2$: 286.0930, found 286.0929 [M+H$^+$].

Go back to table of contents

Evaluation Only. Created with Aspose.Pdf. Copyright 2002-2014 Aspose Pty Ltd.

Ethyl 2-(4-(cyclohexanesulfonamido)phenyl)acetate (8h)

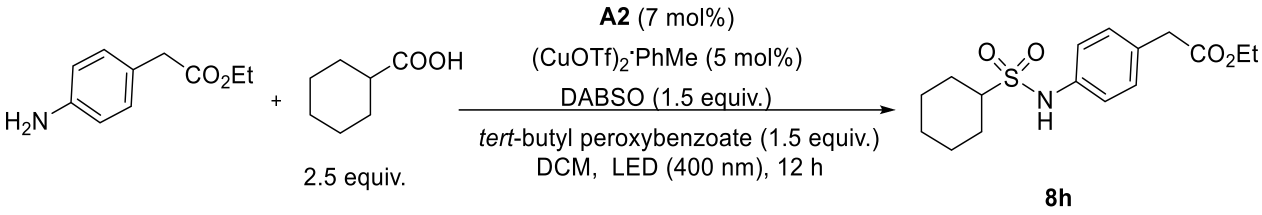

According to **GP2**, the reaction was carried out with ethyl 2-(4-aminophenyl)acetate (54 mg, 0.3 mmol), cyclohexanecarboxylic acid (96 mg, 0.75 mmol, 2.5 equiv.), DABSO (108 mg, 0.45 mmol, 1.5 equiv.), acridine **A2** (6 mg, 0.02 mmol, 7 mol%), *tert*-butyl peroxybenzoate (87 mg, 0.45 mmol, 1.5 equiv.), $(CuOTf)_2$•PhMe (7 mg, 0.015 mmol, 5 mol%) in degassed dichloromethane (3 mL). The test-tube was capped and the reaction mixture was irradiated with LED light (λ = 400 nm) while stirring for 12 h. The reaction mixture was then quenched with saturated solution of sodium hydrogen carbonate (3 mL), extracted with ethyl acetate (3 × 10 mL). The organic layer was collected, dried over anhydrous sodium sulfate, concentrated under reduced pressure, and the remaining material was purified by flash chromatography on silica gel (hexane/ ethyl acetate 2 : 3 v/v) to give the sulfonamide product **8h** (50 mg, 51%) as a yellow liquid.

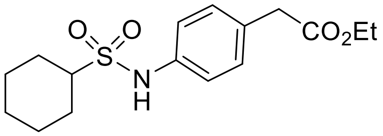

^{1}H NMR (500 MHz, $CDCl_3$): 7.18–7.10 (2 H, m), 7.02–6.92 (2 H, m), 6.70 (1 H, s), 4.13 (2 H, q, *J* = 7.1 Hz), 3.52 (2 H, s), 2.83 (1 H, tt, *J* = 11.2, 3.7 Hz), 2.24–1.12 (13 H, m) ppm. – ^{13}C NMR (125 MHz, $CDCl_3$): 171.8, 141.1, 130.2, 128.4, 118.2, 62.7, 60.9, 40.7, 26.6, 26.3, 25.5, 25.2, 25.0, 14.2 ppm. – IR: 2979, 2932, 2854, 2630, 1732, 1614, 1514, 1450, 1368, 1226, 1150, 1031 cm^{-1}. – HRMS: calcd for $C_{16}H_{23}NO_4SNa$: 348.1240, found 348.1237 [M+Na$^+$].

Evaluation Only. Created with Aspose.Pdf. Copyright 2002-2014 Aspose Pty Ltd.

N-Methyl-*N*-(*p*-tolyl)cyclohexanesulfonamide (8i)

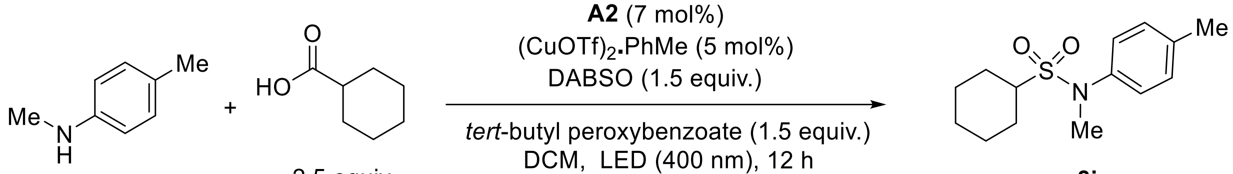

8i

According to **GP2**, the reaction was carried out with *N*,4-dimethylaniline (36 mg, 0.3 mmol), cyclohexanecarboxylic acid (96 mg, 0.75 mmol, 2.5 equiv.), DABSO (108 mg, 0.45 mmol, 1.5 equiv.), acridine **A2** (6 mg, 0.02 mmol, 7 mol%), *tert*-butyl peroxybenzoate (87 mg, 0.45 mmol, 1.5 equiv.), $(CuOTf)_2$•PhMe (7 mg, 0.015 mmol, 5 mol%) in degassed dichloromethane (3 mL). The test-tube was capped and the reaction mixture was irradiated with LED light (λ = 400 nm) while stirring for 12 h. The reaction mixture was then quenched with saturated solution of sodium hydrogen carbonate (3 mL), extracted with ethyl acetate (3 × 10 mL). The organic layer was collected, dried over anhydrous sodium sulfate, concentrated under reduced pressure, and the remaining material was purified by flash chromatography on silica gel (hexane/ ethyl acetate 3 : 2 v/v) to give the sulfonamide **8i** (50 mg, 63%) as a brown solid.

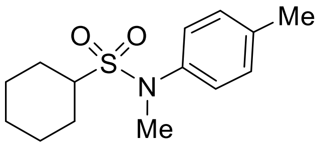

m.p.: 60–62 °C. – ^{1}H NMR (500 MHz, $CDCl_3$): 7.11 (2 H, d, *J* = 8.3 Hz), 7.07–7.03 (2 H, m), 3.10 (3 H, s), 2.77 (1 H, tt, *J* = 11.1, 3.8 Hz), 2.30 (3 H, s), 2.19–2.06 (1 H, m), 1.89 (1 H, dt, *J* = 12.8, 4.0 Hz), 1.84–1.76 (2 H, m), 1.71–1.62 (1 H, m), 1.46 (1 H, dtd, *J* = 12.9, 11.4, 3.6 Hz), 1.37–1.17 (4 H, m) ppm. – ^{13}C NMR (125 MHz, $CDCl_3$): 144.1, 133.6, 129.8, 121.1, 60.4, 31.2, 27.3, 26.4, 25.6, 25.3, 25.3, 20.8 ppm. – IR: 2969, 2930, 2852, 2702, 2488, 1737, 1623, 1516, 1452, 1378, 1225, 1199 cm^{-1}. – HRMS: calcd for $C_{14}H_{21}NO_2S$: 267.1293, found 267.1298 [M+H$^+$].

Evaluation Only. Created with Aspose.Pdf. Copyright 2002-2014 Aspose Pty Ltd.

N-(4-Fluoro-2-methylphenyl)cyclohexanesulfonamide (8j)

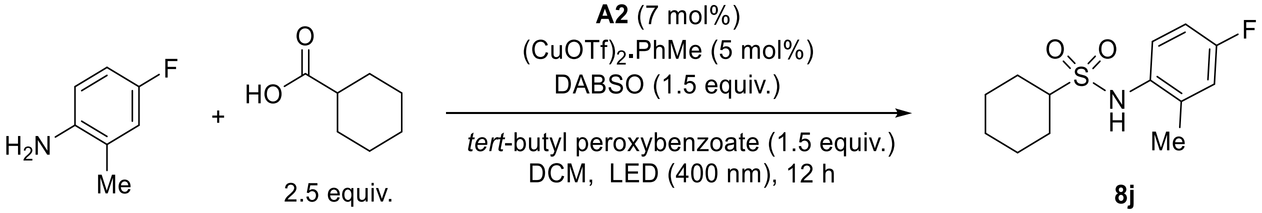

8j

According to **GP2**, the reaction was carried out with 4-fluoro-2-methylaniline (38 mg, 0.3 mmol), cyclohexanecarboxylic acid (96 mg, 0.75 mmol, 2.5 equiv.), DABSO (108 mg, 0.45 mmol, 1.5 equiv.), acridine **A2** (6 mg, 0.02 mmol, 7 mol%), *tert*-butyl peroxybenzoate (87 mg, 0.45 mmol, 1.5 equiv.), $(CuOTf)_2$•PhMe (7 mg, 0.015 mmol, 5 mol%) in degassed dichloromethane (3 mL). The test-tube was capped and the reaction mixture was irradiated with LED light (λ = 400 nm) while stirring for 12 h. The reaction mixture was then quenched with saturated solution of sodium hydrogen carbonate (3 mL), extracted with ethyl acetate (3 × 10 mL). The organic layer was collected, dried over anhydrous sodium sulfate, concentrated under reduced pressure, and the remaining material was purified by flash chromatography on silica gel (hexane/ ethyl acetate 3 : 2 v/v) to give the sulfonamide **8j** (43 mg, 53%) as a yellow oil.

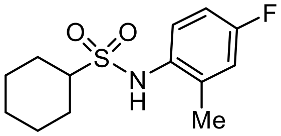

^{1}H NMR (500 MHz, $CDCl_3$): 7.18 (1 H, dd, *J* = 8.7, 5.1 Hz), 6.86 (2 H, ddd, *J* = 17.9, 8.8, 4.2 Hz), 6.02 (1 H, s), 2.86 (1 H, tt, *J* = 11.4, 3.8 Hz), 2.27 (3 H, s), 2.11 (2 H, dt, *J* = 12.6, 3.8 Hz), 1.90 (2 H, tt, *J* = 12.0, 4.1 Hz), 1.71 (1 H, dq, *J* = 10.5, 5.3, 4.6 Hz), 1.58–1.22 (5 H, m) ppm. – ^{13}C NMR (125 MHz, $CDCl_3$): 159.6 (d, $^1J_{C\text{-}F}$ = 242.9 Hz), 135.8, 132.0 (d, $^3J_{C\text{-}F}$ = 8.0 Hz), 122.6 (d, $^3J_{C\text{-}F}$ = 8.4 Hz), 117.5 (d, $^2J_{C\text{-}F}$ = 22.7 Hz), 113.5 (d, $^2J_{C\text{-}F}$ = 22.1 Hz), 63.2, 26.5, 26.4, 25.6, 25.4, 25.2, 18.3 ppm. – ^{19}F NMR (470 MHz, $CDCl_3$): -119.34 (m). – IR: 3045, 2938, 2855, 2642, 1648, 1601, 1551, 1501, 1443, 1322, 1273, 1157, 1128, 1049, 994, 955, 811, 722 cm^{-1}. – HRMS: calcd for $C_{13}H_{18}FNO_2S$: 271.1042, found 271.1040 [M$^+$].

Go back to table of contents

Evaluation Only. Created with Aspose.Pdf. Copyright 2002-2014 Aspose Pty Ltd.

1-(Cyclohexylsulfonyl)indoline (8k)

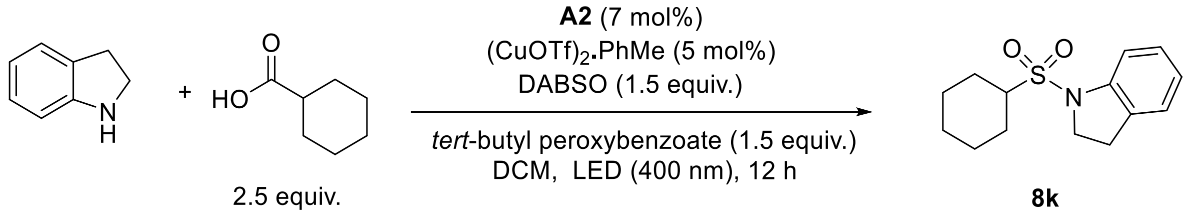

According to **GP2**, the reaction was carried out with indoline (36 mg, 0.3 mmol), cyclohexanecarboxylic acid (96 mg, 0.75 mmol, 2.5 equiv.), DABSO (108 mg, 0.45 mmol, 1.5 equiv.), acridine **A2** (6 mg, 0.02 mmol, 7 mol%), *tert*-butyl peroxybenzoate (87 mg, 0.45 mmol, 1.5 equiv.), $(CuOTf)_2$•PhMe (7 mg, 0.015 mmol, 5 mol%) in degassed dichloromethane (3 mL). The test-tube was capped and the reaction mixture was irradiated with LED light (λ = 400 nm) while stirring for 12 h. The reaction mixture was then quenched with saturated solution of sodium hydrogen carbonate (3 mL), extracted with ethyl acetate (3 × 10 mL). The organic layer was collected, dried over anhydrous sodium sulfate, concentrated under reduced pressure, and the remaining material was purified by flash chromatography on silica gel (hexane/ ethyl acetate 7 : 3 v/v) to give the sulfonamide product **8k** (53 mg, 67%) as a yellow liquid.

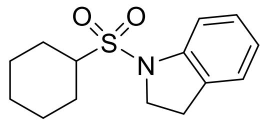

^{1}H NMR (500 MHz, $CDCl_3$): 7.17–7.09 (2 H, m), 6.99 (1 H, d, *J* = 7.7 Hz), 6.90 (1 H, td, *J* = 7.4, 1.0 Hz), 4.25–4.13 (1 H, m), 3.54 (1 H, td, *J* = 10.0, 5.3 Hz), 3.22 (1 H, ddd, *J* = 15.7, 10.5, 5.3 Hz), 3.11 (1 H, ddd, *J* = 15.6, 10.2, 8.4 Hz), 2.85 (1 H, tt, *J* = 11.1, 3.9 Hz), 2.26–2.15 (1 H, m), 1.98–1.79 (3 H, m), 1.75–1.65 (1 H, m), 1.59–1.20 (5 H, m) ppm. – ^{13}C NMR (125 MHz, $CDCl_3$): 147.7, 131.0, 127.7, 125.3, 122.5, 111.7, 60.4, 40.6, 28.9, 27.4, 26.4, 25.6, 25.4, 25.2 ppm. – IR: 2960, 2931, 2871, 1646, 1456, 1264, 1167, 1046 cm^{-1}. – HRMS: calcd for $C_{14}H_{20}NO_2S$: 266.1209, found 266.1208 [M+H$^+$].
– HRMS: calcd for $C_{14}H_{22}NO_2S$: 268.1366, found 268.1365 [M+H$^+$].

Evaluation Only. Created with Aspose.Pdf. Copyright 2002-2014 Aspose Pty Ltd.

4-Phenyl-*N*-(3,4,5-trimethylphenyl)butane-1-sulfonamide (8l)

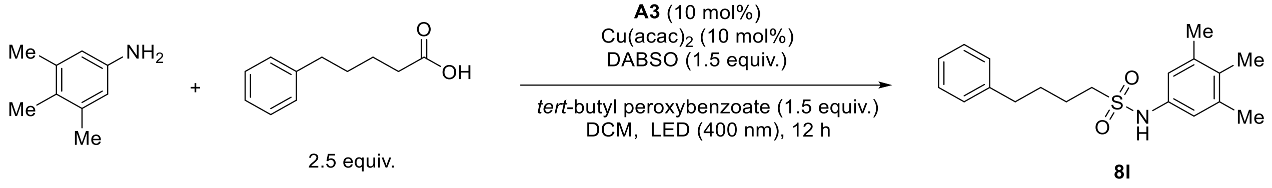

According to **GP2**, the reaction was carried out with 3,4,5-trimethylaniline (41 mg, 0.3 mmol), 5-phenylpentanoic acid (134 mg, 0.75 mmol, 2.5 equiv.), DABSO (108 mg, 0.45 mmol, 1.5 equiv.), acridine **A3** (9 mg, 0.03 mmol, 10 mol%), *tert*-butyl peroxybenzoate (87 mg, 0.45 mmol, 1.5 equiv.), Cu(acac)$_2$ (8 mg, 0.03 mmol, 10 mol%) in degassed dichloromethane (3 mL). The test-tube was capped and the reaction mixture was irradiated with LED light (λ = 400 nm) while stirring for 12 h. The reaction mixture was then quenched with saturated solution of sodium hydrogen carbonate (3 mL), extracted with ethyl acetate (3 × 10 mL). The organic layer was collected, dried over anhydrous sodium sulfate, concentrated under reduced pressure, and the remaining material was purified by flash chromatography on silica gel (hexane/ ethyl acetate 1 : 4 v/v) to give the sulfonamide **8l** (87 mg, 71%) as a yellow oil.

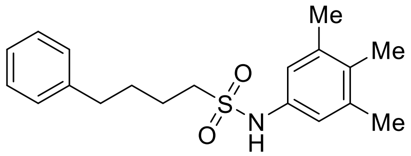

^{1}H NMR (500 MHz, CDCl$_3$): 6.82–6.65 (3 H, m), 2.96 (2 H, t, *J* = 7.7 Hz), 2.19 (6 H, s), 2.09 (3 H, s), 1.72 (2 H, quint., *J* = 7.6 Hz), 1.49–1.13 (24 H, m), 0.89 (3 H, t, *J* = 6.7 Hz) ppm. – ^{13}C NMR (125 MHz, CDCl$_3$): 138.3, 137.6, 129.9, 118.1, 56.0, 32.1, 29.82, 29.79, 29.76, 29.7, 29.52, 29.49, 29.4, 28.8, 23.5, 22.8, 20.7, 14.8, 14.2 ppm. – IR: 3063, 3029, 2939, 2919, 2865, 2631, 1706, 1596, 1552, 1485, 1453, 1265, 1169, 1037, 909, 736, 701 cm^{-1}. – HRMS: calcd for $C_{19}H_{25}NO_2S$: 331.1606, found 331.1598 [M$^+$].

Go back to table of contents

Evaluation Only. Created with Aspose.Pdf. Copyright 2002-2014 Aspose Pty Ltd.

N-(3-Isopropylphenyl)butane-1-sulfonamide (8m)

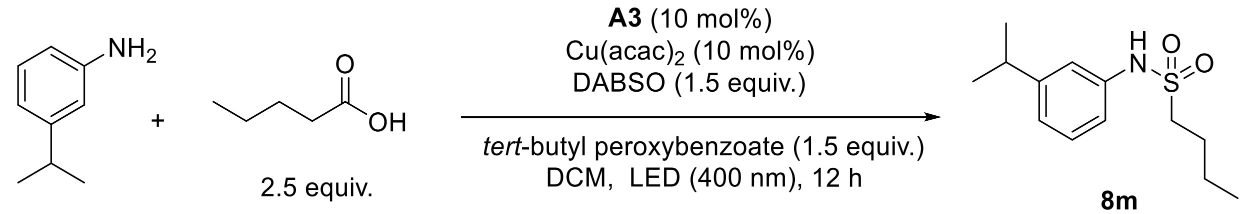

According to **GP2**, the reaction was carried out with 3-isopropylaniline (41 mg, 0.3 mmol), valeric acid (77 mg, 0.75 mmol, 2.5 equiv.), DABSO (108 mg, 0.45 mmol, 1.5 equiv.), acridine **A3** (9 mg, 0.03 mmol, 10 mol%), *tert*-butyl peroxybenzoate (70 mg, 0.36 mmol, 1.2 equiv.), $Cu(acac)_2$ (8 mg, 0.03 mmol, 10 mol%) in degassed dichloromethane (3 mL). The test-tube was capped and the reaction mixture was irradiated with LED light (λ = 400 nm) while stirring for 12 h. The reaction mixture was then quenched with saturated solution of sodium hydrogen carbonate (3 mL), extracted with ethyl acetate (3 × 10 mL). The organic layer was collected, dried over anhydrous sodium sulfate, concentrated under reduced pressure, and the remaining material was purified by flash chromatography on silica gel (hexane/ ethyl acetate 3 : 2 v/v) to give the sulfonamide **8m** (53 mg, 69%) as a yellow oil.

Gram scale for compound 13m: According to **GP2**, the reaction was carried out with 3-isopropylaniline (1.08 g, 8 mmol), valeric acid (2.04 g, 20 mmol, 2.5 equiv.), DABSO (2.88 g, 12 mmol, 1.5 equiv.), acridine **A3** (238 mg, 0.8 mmol, 10 mol%), *tert*-butyl peroxybenzoate (1.86 mg, 0.36 mmol, 1.2 equiv.), $Cu(acac)_2$ (211 mg, 0.8 mmol, 10 mol%) in degassed dichloromethane (80 mL). The test-tube was capped and the reaction mixture was irradiated with LED light (λ = 400 nm) while stirring for 12 h. The reaction mixture was then quenched with saturated solution of sodium hydrogen carbonate (30 mL), extracted with ethyl acetate (3 × 50 mL). The organic layer was collected, dried over anhydrous sodium sulfate, concentrated under reduced pressure, and the remaining material was purified by flash chromatography on silica gel (hexane/ ethyl acetate 3 : 2 v/v) to give the sulfonamide **8m** (1.34 g, 66%) as a yellow oil.

Evaluation Only. Created with Aspose.Pdf. Copyright 2002-2014 Aspose Pty Ltd.

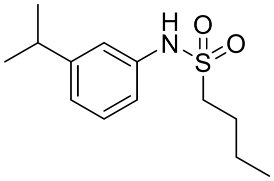

^{1}H NMR (500 MHz, $CDCl_3$): 7.36–7.22 (1 H, m), 7.14 (1 H, t, *J* = 7.8 Hz), 6.92–6.81 (3 H, m), 3.05–2.94 (2 H, m), 2.82 (1 H, dq, *J* = 13.9, 6.9 Hz), 1.69 (2 H, quint., *J* = 7.6 Hz), 1.50–1.34 (2 H, m), 1.20 (6 H, dd, *J* = 6.9, 3.3 Hz), 0.92 (3 H, t, *J* = 7.3 Hz) ppm. – ^{13}C NMR (125 MHz, $CDCl_3$): 150.6, 141.6, 129.4, 121.1, 116.4, 115.6, 55.7, 34.2, 25.5, 24.0, 21.9, 13.8 ppm. – IR: 3438, 2960, 2929, 2872, 1737, 1607, 1591, 1539, 1499, 1319, 1157, 1036, 791, 733 cm^{-1}. – HRMS: calcd for $C_{13}H_{22}NO_2S$: 256.1366, found 256.1375 [$M+H^+$].

N-(3-Acetylphenyl)butane-1-sulfonamide (8n)

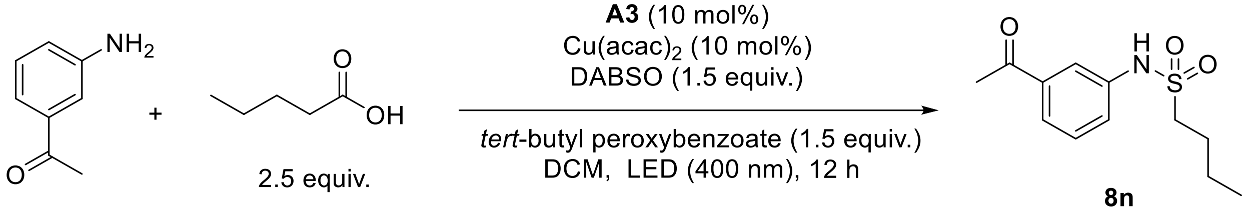

According to **GP2**, the reaction was carried out with 1-(3-aminophenyl)ethan-1-one (41 mg, 0.3 mmol), valeric acid (77 mg, 0.75 mmol, 2.5 equiv.), DABSO (108 mg, 0.45 mmol, 1.5 equiv.), acridine **A3** (9 mg, 0.03 mmol, 10 mol%), *tert*-butyl peroxybenzoate (87 mg, 0.45 mmol, 1.5 equiv.), $Cu(acac)_2$ (8 mg, 0.03 mmol, 10 mol%) in degassed dichloromethane (3 mL). The test-tube was capped and the reaction mixture was irradiated with LED light (λ = 400 nm) while stirring for 12 h. The reaction mixture was then quenched with saturated solution of sodium hydrogen carbonate (3 mL), extracted with ethyl acetate (3 × 10 mL). The organic layer was collected, dried over anhydrous sodium sulfate, concentrated under reduced pressure, and the remaining material was purified by flash chromatography on silica gel (hexane/ ethyl acetate 3 : 2 v/v) to give the sulfonamide **8n** (41 mg, 53%) as a yellow oil.

Evaluation Only. Created with Aspose.Pdf. Copyright 2002-2014 Aspose Pty Ltd.

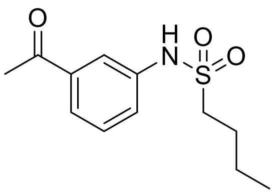

^{1}H NMR (500 MHz, $CDCl_3$): 7.82 (1 H, s), 7.53 (1 H, t, *J* = 2.0 Hz), 7.48 (1 H, dt, *J* = 7.6, 1.4 Hz), 7.23 (1 H, t, *J* = 7.8 Hz), 7.18 (1 H, ddd, *J* = 8.0, 2.4, 1.1 Hz), 3.10–3.00 (2 H, m), 2.48 (3 H, s), 1.70 (2 H, quint., *J* = 7.6 Hz, 2H), 1.51–1.35 (2 H, m), 0.91 (3 H, t, *J* = 7.4 Hz) ppm. – ^{13}C NMR (125 MHz, $CDCl_3$): 197.8, 142.3, 138.2, 129.7, 122.7, 122.1, 117.2, 55.6, 26.7, 25.4, 21.9, 13.8 ppm. – IR: 3365, 3076, 3053, 3008, 2967, 2901, 1712, 1423, 1361, 1272, 1222, 1157, 916, 735 cm^{-1}. – HRMS: calcd for $C_{12}H_{18}NO_3S$: 256.1002, found 256.1009 [M+H$^+$].

1-Cyclopentyl-*N*-(2,3-dihydro-1*H*-inden-5-yl)methanesulfonamide (8o)

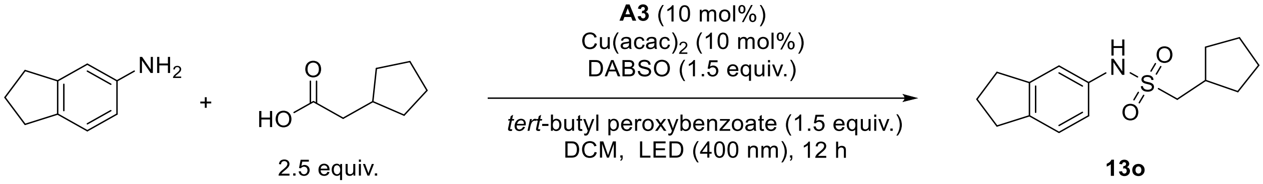

According to **GP2**, the reaction was carried out with 2,3-dihydro-1*H*-inden-5-amine (40 mg, 0.3 mmol), 2-cyclopentylacetic acid (96 mg, 0.75 mmol, 2.5 equiv.), DABSO (108 mg, 0.45 mmol, 1.5 equiv.), acridine **A3** (9 mg, 0.03 mmol, 10 mol%), *tert*-butyl peroxybenzoate (87 mg, 0.45 mmol, 1.5 equiv.), $Cu(acac)_2$ (8 mg, 0.03 mmol, 10 mol%) in degassed dichloromethane (3 mL). The test-tube was capped and the reaction mixture was irradiated with LED light (λ = 400 nm) while stirring for 12 h. The reaction mixture was then quenched with saturated solution of sodium hydrogen carbonate (3 mL), extracted with ethyl acetate (3 × 10 mL). The organic layer was collected, dried over anhydrous sodium sulfate, concentrated under reduced pressure, and the remaining material was purified by flash chromatography on silica gel (hexane/ ethyl acetate 3 : 2 v/v) to give the sulfonamide **8o** (66 mg, 79%) as a yellow solid.

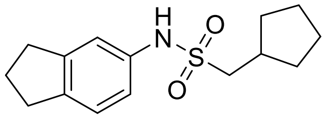

m.p.: 50–52 °C. – ^{1}H NMR (500 MHz, $CDCl_3$): 7.14 (1 H, s), 7.05 (1 H, d, *J* = 7.9 Hz), 6.92 (1 H, d, *J* = 2.1 Hz), 6.80 (1 H, dd, *J* = 8.0, 2.1 Hz), 3.10–2.93 (2 H, m), 2.80 (4 H, tq, *J* = 15.8, 8.4, 7.3 Hz), 2.23 (1 H, quint., *J* = 7.9 Hz),

Evaluation Only. Created with Aspose.Pdf. Copyright 2002-2014 Aspose Pty Ltd.

2.09–1.90 (3 H, m), 1.87–1.76 (1 H, m), 1.72–1.50 (4 H, m), 1.33–1.19 (2 H, m) ppm. – ^{13}C NMR (125 MHz, $CDCl_3$): 145.7, 139.7, 139.0, 124.9, 116.9, 115.1, 62.1, 35.3, 33.0, 32.6, 32.2, 25.7, 25.1, 25.0 ppm. – IR: 2949, 2927, 2867, 2849, 1613, 1490, 1451, 1326, 1266, 1154, 1037, 907, 733 cm^{-1}. – HRMS: calcd for $C_{15}H_{22}NO_2S$: 280.1366, found 280.1371 [$M+H^+$].

N-(2-(Methylthio)phenyl)tetrahydro-2*H*-pyran-4-sulfonamide (8p)

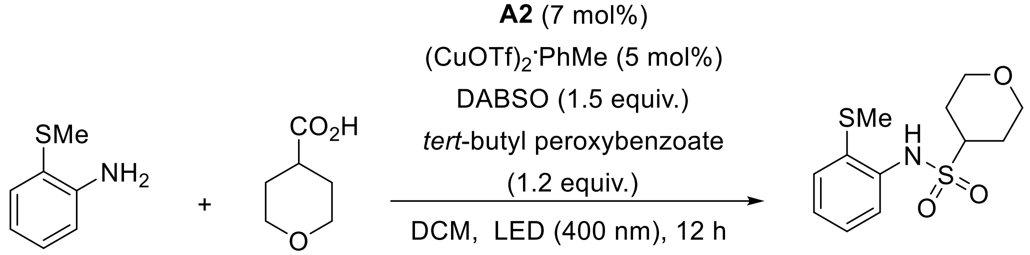

8p

According to **GP2**, the reaction was carried out with 2-(methylthio)aniline (42 mg, 0.3 mmol), tetrahydro-2*H*-pyran-4-carboxylic acid (98 mg, 0.75 mmol, 2.5 equiv.), DABSO (108 mg, 0.45 mmol, 1.5 equiv.), acridine **A2** (6 mg, 0.02 mmol, 7 mol%), *tert*-butyl peroxybenzoate (87 mg, 0.45 mmol, 1.5 equiv.), $(CuOTf)_2$•PhMe (7 mg, 0.015 mmol, 5 mol%) in degassed dichloromethane (3 mL). The test-tube was capped and the reaction mixture was irradiated with LED light (λ = 400 nm) while stirring for 12 h. The reaction mixture was then quenched with saturated solution of sodium hydrogen carbonate (3 mL), extracted with ethyl acetate (3 × 10 mL). The organic layer was collected, dried over anhydrous sodium sulfate, concentrated under reduced pressure, and the remaining material was purified by flash chromatography on silica gel (hexane/ ethyl acetate 3 : 2 v/v) to give the sulfonamide **8p** (48 mg, 56%) as a yellow oil.

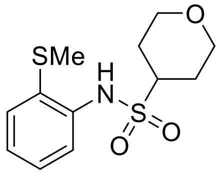

^{1}H NMR (500 MHz, $CDCl_3$): 7.44 (1 H, d, *J* = 7.8 Hz), 7.29–7.17 (2 H, m), 7.06–6.94 (1 H, m), 6.91 (1 H, brs), 4.19–4.00 (3 H, m), 3.45 (2 H, td, *J* = 11.7, 2.6 Hz), 3.04 (1 H, tt, *J* = 11.8, 4.2 Hz), 2.34 (3 H, d, *J* = 1.3 Hz), 2.06 (1 H, d, *J* = 13.5 Hz), 2.00–1.69 (3 H, m) ppm. – ^{13}C NMR (125 MHz, $CDCl_3$): 142.2, 133.8,

Evaluation Only. Created with Aspose.Pdf. Copyright 2002-2014 Aspose Pty Ltd.

129.4, 126.1, 123.8, 117.6, 67.0, 66.9, 60.4, 26.4, 26.1, 19.3 ppm. – IR: 3418, 2956, 2850, 1626, 1479, 1447, 1318, 1268, 1238, 1199, 1159, 1130, 1105, 1085, 1048, 1012 cm^{-1}. – HRMS: calcd for $C_{12}H_{16}NO_3S_2$: 286.0577, found 286.0578 [M-H]$^-$.

N-(3,5-Dimethylphenyl)heptane-4-sulfonamide (8q)

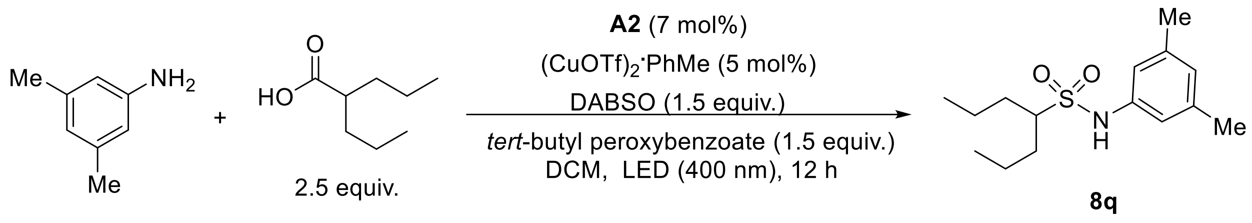

According to **GP2**, the reaction was carried out with 3,5-dimethylaniline (36 mg, 0.3 mmol), valproic acid (108 mg, 0.75 mmol, 2.5 equiv.), DABSO (108 mg, 0.45 mmol, 1.5 equiv.), acridine **A2** (6 mg, 0.02 mmol, 7 mol%), *tert*-butyl peroxybenzoate (87 mg, 0.45 mmol, 1.5 equiv.), $(CuOTf)_2$•PhMe (7 mg, 0.015 mmol, 5 mol%) in degassed dichloromethane (3 mL). The test-tube was capped and the reaction mixture was irradiated with LED light (λ = 400 nm) while stirring for 12 h. The reaction mixture was then quenched with saturated solution of sodium hydrogen carbonate (3 mL), extracted with ethyl acetate (3 × 10 mL). The organic layer was collected, dried over anhydrous sodium sulfate, concentrated under reduced pressure, and the remaining material was purified by flash chromatography on silica gel (hexane/ ethyl acetate 3 : 2 v/v) to give the sulfonamide **8q** (43 mg, 51%) as a yellow oil.

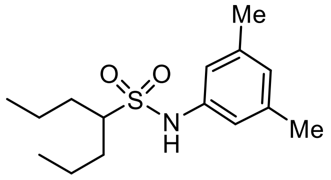

^{1}H NMR (500 MHz, $CDCl_3$): 6.66–6.61 (3 H, m), 6.56 (1 H, s), 2.86 (1 H, ddd, *J* = 12.1, 7.4, 5.0 Hz), 2.23 (6 H, s), 1.92–1.64 (3 H, m), 1.62–1.35 (5 H, m), 1.00–0.90 (6 H, m) ppm. – ^{13}C NMR (125 MHz, $CDCl_3$): 141.9, 139.2, 124.7, 116.0, 64.1, 30.1, 28.8, 21.4, 20.2, 19.5, 14.3, 14.2 ppm. – IR: 2958, 2930, 2872, 2655, 1722, 1604, 1546, 1463, 1384, 1335, 1227, 1191, 1157, 1034, 856, 691 cm^{-1}. – HRMS: calcd for $C_{15}H_{25}NO_2S$: 283.1606, found 283.1599 [M+H$^+$].

Evaluation Only. Created with Aspose.Pdf. Copyright 2002-2014 Aspose Pty Ltd.

N-(2,3-Dihydro-1*H*-inden-5-yl)-4,4-difluorocyclohexane-1-sulfonamide (8r)

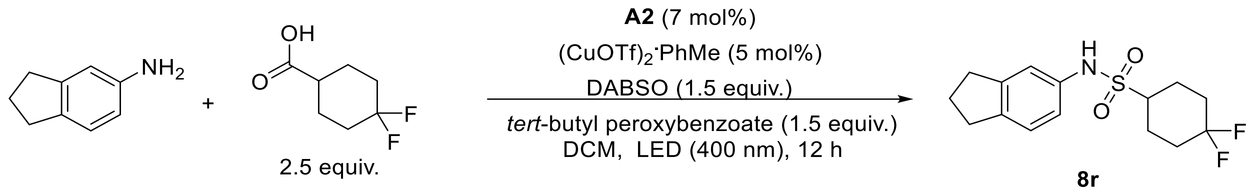

According to **GP2**, the reaction was carried out with 2,3-dihydro-1*H*-inden-5-amine (40 mg, 0.3 mmol), 4,4-difluorocyclohexane-1-carboxylic acid (123 mg, 0.75 mmol, 2.5 equiv.), DABSO (108 mg, 0.45 mmol, 1.5 equiv.), acridine **A2** (6 mg, 0.02 mmol, 7 mol%), *tert*-butyl peroxybenzoate (87 mg, 0.45 mmol, 1.5 equiv.), $(CuOTf)_2$•PhMe (7 mg, 0.015 mmol, 5 mol%) in degassed dichloromethane (3 mL). The test-tube was capped and the reaction mixture was irradiated with LED light (λ = 400 nm) while stirring for 12 h. The reaction mixture was then quenched with saturated solution of sodium hydrogen carbonate (3 mL), extracted with ethyl acetate (3 × 10 mL). The organic layer was collected, dried over anhydrous sodium sulfate, concentrated under reduced pressure, and the remaining material was purified by flash chromatography on silica gel (hexane/ ethyl acetate 3 : 2 v/v) to give the sulfonamide **8r** (79 mg, 83%) as a yellow solid.

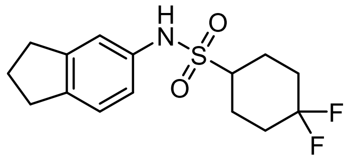

m.p.: 60–62 °C. – ^{1}H NMR (500 MHz, $CDCl_3$): 7.13–7.05 (2 H, m), 6.93 (1 H, d, *J* = 2.1 Hz), 6.81 (1 H, dd, *J* = 8.0, 2.2 Hz), 2.91 (1 H, td, *J* = 9.9, 9.1, 5.5 Hz), 2.82 (4 H, dt, *J* = 13.1, 7.1 Hz), 2.22–2.01 (6 H, m), 1.95–1.56 (4 H, m) ppm. – ^{13}C NMR (125 MHz, $CDCl_3$): 145.9, 139.5, 139.4, 125.0, 122.3 (t, $^1J_{C\text{-}F}$ = 241.3 Hz), 116.8, 114.9, 60.1, 32.9, 32.2 (q, $^2J_{C\text{-}F}$ = 24.7 Hz), 32.1, 25.6, 23.1 (dd, $^2J_{C\text{-}F}$, $^3J_{C\text{-}F}$ = 32.8, 8.3 Hz) ppm. – ^{19}F NMR (470 MHz, $CDCl_3$): –95.38 (d, *J* = 235.9 Hz), –101.25 (d, *J* = 242.2 Hz) ppm. – IR: 3156, 2940, 2870, 2845, 1717, 1613, 1596, 1489, 1448, 1374, 1292, 1269, 1122, 1106, 1051, 960, 857, 816 cm^{-1}. – HRMS: calcd for $C_{15}H_{20}F_2NO_2S$: 316.1177, found 316.1182 [M+H$^+$].

Evaluation Only. Created with Aspose.Pdf. Copyright 2002-2014 Aspose Pty Ltd.

N-(2-Chloro-4-methoxyphenyl)tetrahydro-2*H*-pyran-4-sulfonamide (8s)

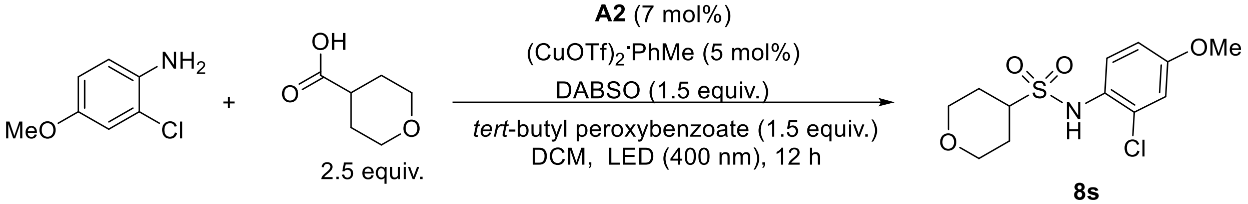

8s

According to **GP2**, the reaction was carried out with 2,3-dihydro-1*H*-inden-5-amine (40 mg, 0.3 mmol), 4,4-difluorocyclohexane-1-carboxylic acid (123 mg, 0.75 mmol, 2.5 equiv.), DABSO (108 mg, 0.45 mmol, 1.5 equiv.), acridine **A2** (6 mg, 0.02 mmol, 7 mol%), *tert*-butyl peroxybenzoate (87 mg, 0.45 mmol, 1.5 equiv.), $(CuOTf)_2$•PhMe (7 mg, 0.015 mmol, 5 mol%) in degassed dichloromethane (3 mL). The test-tube was capped and the reaction mixture was irradiated with LED light (λ = 400 nm) while stirring for 12 h. The reaction mixture was then quenched with saturated solution of sodium hydrogen carbonate (3 mL), extracted with ethyl acetate (3 × 10 mL). The organic layer was collected, dried over anhydrous sodium sulfate, concentrated under reduced pressure, and the remaining material was purified by flash chromatography on silica gel (hexane/ ethyl acetate 3 : 2 v/v) to give the sulfonamide **8s** (75 mg, 82%) as a yellow oil.

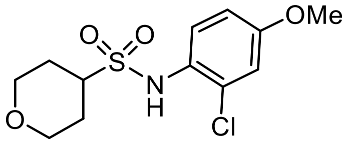

^{1}H NMR (500 MHz, $CDCl_3$): 7.08 (1 H, d, *J* = 2.7 Hz), 6.95–6.87 (2 H, m), 6.77 (1 H, d, *J* = 8.8 Hz), 4.12–4.00 (2 H, m), 3.84 (3 H, s), 3.36 (2 H, dtd, *J* = 22.8, 11.5, 2.4 Hz), 3.04 (1 H, tt, *J* = 11.7, 4.2 Hz), 2.05–1.66 (4 H, m) ppm. – ^{13}C NMR (125 MHz, $CDCl_3$): 151.8, 135.0, 123.2, 121.9, 118.9, 113.0, 66.9, 66.7, 59.9, 56.6, 26.9, 26.4 ppm. – IR: 3058, 3024, 2982, 2844, 1796, 1573, 1504, 1460, 1264, 1152, 1064, 909, 731, 705 cm^{-1}. – HRMS: calcd for $C_{12}H_{16}ClNO_4S$: 305.0489, found 305.0489 [M$^+$].

Evaluation Only. Created with Aspose.Pdf. Copyright 2002-2014 Aspose Pty Ltd.

N-(4-Chlorophenyl)-*N*-methylbutane-1-sulfonamide (8t)

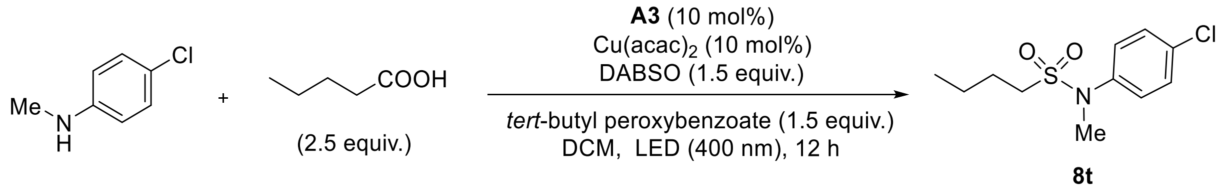

According to **GP2**, the reaction was carried out with 4-chloro-*N*-methylaniline (42 mg, 0.3 mmol), pentanoic acid (77 mg, 0.75 mmol, 2.5 equiv.), DABSO (108 mg, 0.45 mmol, 1.5 equiv.), acridine **A3** (9 mg, 0.03 mmol, 10 mol%), *tert*-butyl peroxybenzoate (87 mg, 0.45 mmol, 1.5 equiv.), Cu(acac)$_2$ (8 mg, 0.03 mmol, 10 mol%) in degassed dichloromethane (3 mL). The test-tube was capped and the reaction mixture was irradiated with LED light (λ = 400 nm) while stirring for 12 h. The reaction mixture was then quenched with saturated solution of sodium hydrogen carbonate (3 mL), extracted with ethyl acetate (3 × 10 mL). The organic layer was collected, dried over anhydrous sodium sulfate, concentrated under reduced pressure, and the remaining material was purified by flash chromatography on silica gel (hexane/ ethyl acetate 7 : 3 v/v) to give the sulfonamide product **8t** (42 mg, 54%) as a yellow liquid.

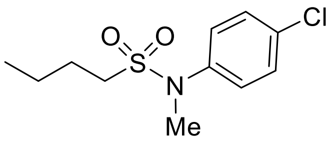

^{1}H NMR (500 MHz, CDCl$_3$): 7.32–7.27 (2 H, m), 7.14–7.08 (2 H, m), 3.11 (3 H, s), 2.81 (2 H, ddt, *J* = 8.7, 6.3, 4.6 Hz), 1.60 (2 H, ddt, *J* = 40.6, 13.5, 6.9 Hz), 1.45 (2 H, h, *J* = 7.4 Hz), 0.93 (3 H, t, *J* = 7.4 Hz) ppm. – ^{13}C NMR (125 MHz, CDCl$_3$): 144.5, 129.7, 129.4, 122.6, 53.4, 31.5, 25.4, 22.1, 13.9 ppm. – IR: 3055, 2972, 2308, 1739, 1421, 1264, 895 cm^{-1}. – HRMS: calcd for $C_{11}H_{16}ClNO_2S$: 361.0590, found 361.0592 [M$^+$].

Evaluation Only. Created with Aspose.Pdf. Copyright 2002-2014 Aspose Pty Ltd.

1-((4,4-Difluorocyclohexyl)sulfonyl)indoline (8u)

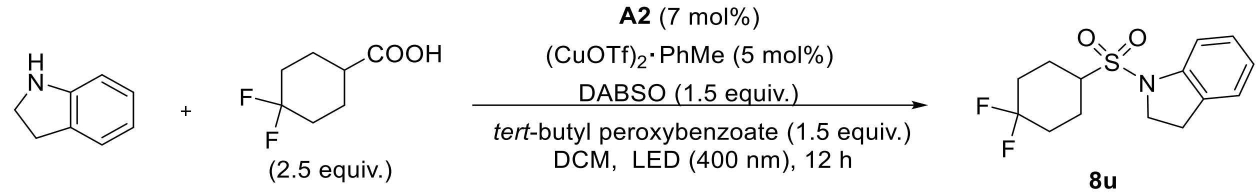

According to **GP2**, the reaction was carried out with indoline (36 mg, 0.3 mmol), 4,4-difluorocyclohexane-1-carboxylic acid (123 mg, 0.75 mmol, 2.5 equiv.), DABSO (108 mg, 0.45 mmol, 1.5 equiv.), acridine **A2** (6 mg, 0.02 mmol, 7 mol%), *tert*-butyl peroxybenzoate (87 mg, 0.45 mmol, 1.5 equiv.), $(CuOTf)_2$•PhMe (7 mg, 0.015 mmol, 5 mol%) in degassed dichloromethane (3 mL). The test-tube was capped and the reaction mixture was irradiated with LED light (λ = 400 nm) while stirring for 12 h. The reaction mixture was then quenched with saturated solution of sodium hydrogen carbonate (3 mL), extracted with ethyl acetate (3 × 10 mL). The organic layer was collected, dried over anhydrous sodium sulfate, concentrated under reduced pressure, and the remaining material was purified by flash chromatography on silica gel (hexane/ ethyl acetate 3 : 2 v/v) to give the sulfonamide product **8u** (61 mg, 68%) as a white solid.

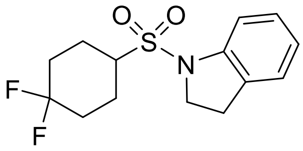

m.p.: 45–47 °C. – ^{1}H NMR (500 MHz, $CDCl_3$): 7.63 (1 H, d, *J* = 7.8 Hz), 7.44–7.34 (3 H, m), 3.98 (2 H, t, *J* = 7.7 Hz), 3.33 (2 H, t, *J* = 7.7 Hz), 2.66 (1 H, td, *J* = 10.9, 5.4 Hz), 2.13–2.02 (4 H, m), 1.77–1.55 (4 H, m) ppm. – ^{13}C NMR (125 MHz, $CDCl_3$): 136.2, 135.0, 129.8, 128.4, 125.8, 122.8 (t, *J* = 241.2 Hz), 120.0, 56.4, 46.0, 32.5 (t, *J* = 24.5 Hz), 29.3, 24.0 (d, *J* = 9.5 Hz) ppm. – ^{19}F NMR (470 MHz, $CDCl_3$): –94.00 (d, *J* = 241.8 Hz), –102.07 (d, *J* = 238.1 Hz), ppm. – IR: 2960, 2922, 2853, 2522, 1723, 1621, 1593, 1466, 1466, 1375, 1207, 1149 cm^{-1}. – HRMS: calcd for $C_{14}H_{18}F_2NO_2S$: 302.1021, found 302.1021 [M+H$^+$].

Go back to table of contents

Evaluation Only. Created with Aspose.Pdf. Copyright 2002-2014 Aspose Pty Ltd.

tert-Butyl 4-(*N*-(3,4,5-trimethylphenyl)sulfamoyl)piperidine-1-carboxylate (8v)

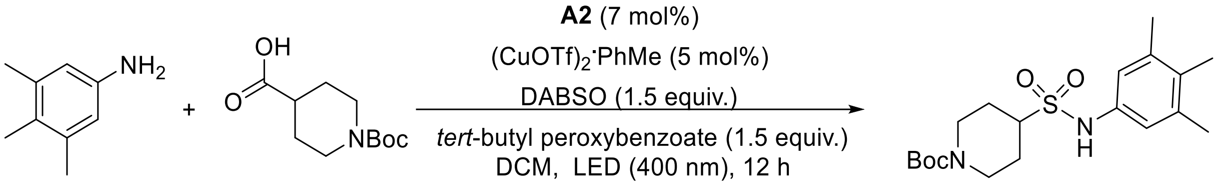

2.5 equiv.

8v

According to **GP2**, the reaction was carried out with 3,4,5-trimethylaniline (41 mg, 0.3 mmol), 1-(*tert*-butoxycarbonyl)piperidine-4-carboxylic acid (172 mg, 0.75 mmol, 2.5 equiv.), DABSO (108 mg, 0.45 mmol, 1.5 equiv.), acridine **A2** (6 mg, 0.02 mmol, 7 mol%), *tert*-butyl peroxybenzoate (87 mg, 0.45 mmol, 1.5 equiv.), $(CuOTf)_2$•PhMe (7 mg, 0.015 mmol, 5 mol%) in degassed dichloromethane (3 mL). The test-tube was capped and the reaction mixture was irradiated with LED light (λ = 400 nm) while stirring for 12 h. The reaction mixture was then quenched with saturated solution of sodium hydrogen carbonate (3 mL), extracted with ethyl acetate (3 × 10 mL). The organic layer was collected, dried over anhydrous sodium sulfate, concentrated under reduced pressure, and the remaining material was purified by flash chromatography on silica gel (hexane/ ethyl acetate 3 : 2 v/v) to give the sulfonamide **8v** (85 mg, 74%) as a yellow oil.

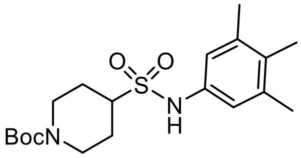

^{1}H NMR (500 MHz, $CDCl_3$): 6.69–6.53 (3 H, m), 4.13 (2 H, s), 2.96 (1 H, tt, *J* = 11.5, 4.0 Hz), 2.87–2.64 (2 H, m), 2.19 (6 H, s), 2.08 (4 H, s), 2.03–1.92 (1 H, m), 1.78–1.51 (2 H, m), 1.46 (9 H, s) ppm. – ^{13}C NMR (125 MHz, $CDCl_3$): 154.7, 138.3, 137.8, 130.3, 118.2, 80.1, 61.1, 43.2, 28.5, 26.3, 25.8, 20.7, 14.9 ppm. – IR: 3055, 2956, 2308, 1738, 1424, 1365, 1265, 1216, 908 cm^{-1}. – HRMS: calcd for $C_{19}H_{31}N_2O_4S$: 383.1999, found 383.2010 [M+H$^+$].

Evaluation Only. Created with Aspose.Pdf. Copyright 2002-2014 Aspose Pty Ltd.

N-(2-Methylbenzo[d]thiazol-6-yl)butane-1-sulfonamide (8w)

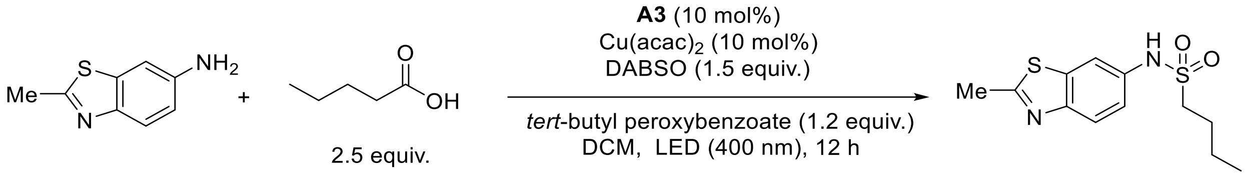

8w

According to **GP2**, the reaction was carried out with 2-methylbenzo[*d*]thiazol-6-amine (49 mg, 0.3 mmol), valeric acid (77 mg, 0.75 mmol, 2.5 equiv.), DABSO (108 mg, 0.45 mmol, 1.5 equiv.), acridine **A3** (9 mg, 0.03 mmol, 10 mol%), *tert*-butyl peroxybenzoate (70 mg, 0.36 mmol, 1.2 equiv.), $Cu(acac)_2$ (8 mg, 0.03 mmol, 10 mol%) in degassed dichloromethane (3 mL). The test-tube was capped and the reaction mixture was irradiated with LED light (λ = 400 nm) while stirring for 12 h. The reaction mixture was then quenched with saturated solution of sodium hydrogen carbonate (3 mL), extracted with ethyl acetate (3 × 10 mL). The organic layer was collected, dried over anhydrous sodium sulfate, concentrated under reduced pressure, and the remaining material was purified by flash chromatography on silica gel (hexane/ ethyl acetate 3 : 2 v/v) to give the sulfonamide **8w** (60 mg, 70%) as a yellow oil.

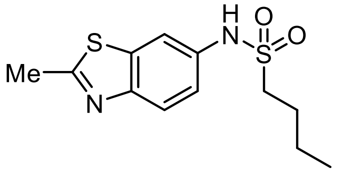

^{1}H NMR (500 MHz, $CDCl_3$): 8.77 (1 H, s), 7.93–7.76 (2 H, m), 7.50 (1 H, d, *J* = 2.2 Hz), 7.16 (1 H, dd, *J* = 8.8, 2.3 Hz), 3.07 (2 H, ddd, *J* = 9.3, 6.5, 1.6 Hz), 1.73 (2 H, quint., *J* = 7.6 Hz), 1.53–1.36 (2 H, m), 0.92 (3 H, t, *J* = 7.3 Hz) ppm. – ^{13}C NMR (125 MHz, $CDCl_3$): 152.5, 149.4, 139.8, 135.2, 124.1, 117.8, 110.2, 55.7, 25.5, 21.9, 13.8 ppm. – IR: 3346, 2958, 2930, 2872, 2637, 1738, 1606, 1560, 1526, 1464, 1433, 1378, 1322, 1204, 1163, 1041, 817, 736 cm^{-1}. – HRMS: calcd for $C_{12}H_{16}N_2O_2S_2$: 284.0653, found 284.0649 [M$^+$].

Evaluation Only. Created with Aspose.Pdf. Copyright 2002-2014 Aspose Pty Ltd.

1-((3,3,3-Trifluoropropyl)sulfonyl)indoline (8x)

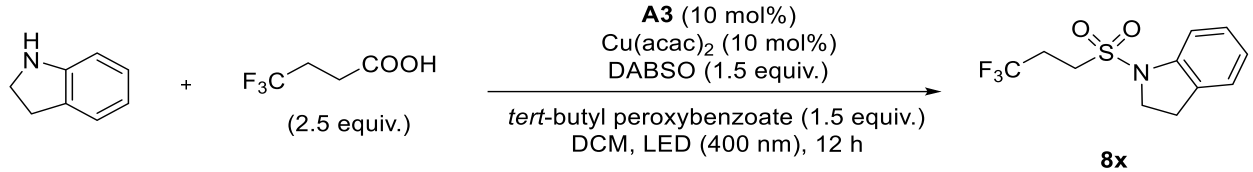

8x

According to **GP2**, the reaction was carried out with indoline (36 mg, 0.3 mmol), 4,4,4-trifluorobutanoic acid (107 mg, 0.75 mmol, 2.5 equiv.), DABSO (108 mg, 0.45 mmol, 1.5 equiv.), acridine **A3** (9 mg, 0.03 mmol, 10 mol%), *tert*-butyl peroxybenzoate (87 mg, 0.45 mmol, 1.5 equiv.), $Cu(acac)_2$ (8 mg, 0.03 mmol, 10 mol%) in degassed dichloromethane (3 mL). The test-tube was capped and the reaction mixture was irradiated with LED light (λ = 400 nm) while stirring for 12 h. The reaction mixture was then quenched with saturated solution of sodium hydrogen carbonate (3 mL), extracted with ethyl acetate (3 × 10 mL). The organic layer was collected, dried over anhydrous sodium sulfate, concentrated under reduced pressure, and the remaining material was purified by flash chromatography on silica gel (hexane/ ethyl acetate 7 : 3 v/v) to give the sulfonamide product **8x** (52 mg, 62%) as a yellow liquid.

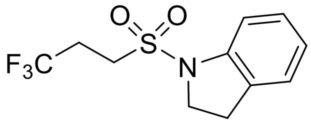

^{1}H NMR (500 MHz, $CDCl_3$): 7.17 (2 H, ddd, *J* = 8.9, 7.6, 1.1 Hz), 7.06–6.86 (2 H, m), 4.24 (1 H, ddd, *J* = 10.5, 9.7, 8.2 Hz), 3.59 (1 H, td, *J* = 10.0, 5.3 Hz), 3.26 (1 H, ddd, *J* = 15.8, 10.5, 5.3 Hz), 3.21–3.03 (3 H, m), 2.67–2.40 (2 H, m) ppm. – ^{13}C NMR (125 MHz, $CDCl_3$): 146.5, 131.3, 127.9, 126.1 (q, *J* = 276.4 Hz), 125.6, 123.4, 112.3, 53.6, 46.0, 40.4, 28.9 (q, *J* = 30.8 Hz), 28.9 ppm. – ^{19}F NMR (470 MHz, $CDCl_3$) –66.6 ppm. – IR: 3053, 3002, 2944, 2885, 1750, 1419, 1265, 911, 750 cm^{-1}. – HRMS: calcd for $C_{11}H_{12}NO_2F_3S$: 279.0541, found 279.0542 [M^+].

Go back to table of contents

Evaluation Only. Created with Aspose.Pdf. Copyright 2002-2014 Aspose Pty Ltd.

Sulfonyl azides

2-(3,4-Dimethoxyphenyl)ethane-1-sulfonyl azide (9a)

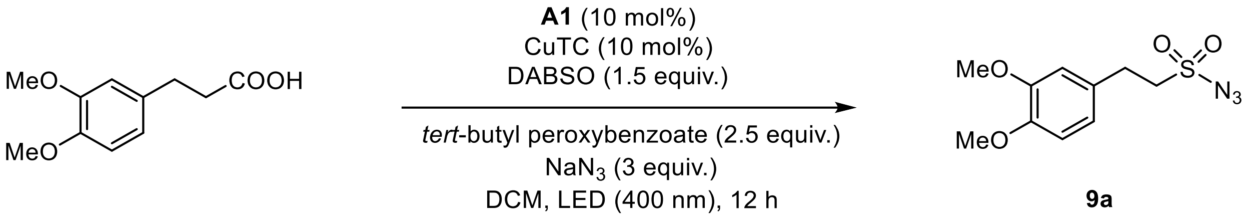

According to **GP3**, the reaction was carried out with 3-(3,4-dimethoxyphenyl)propanoic acid (63 mg, 0.3 mmol), DABSO (108 mg, 0.45 mmol, 1.5 equiv.), acridine catalyst **A1** (9 mg, 0.03 mmol, 10 mol%), potassium azide (73 mg, 0.9 mmol, 3.0 equiv.), *tert*-butyl peroxybenzoate (145 mg, 0.75 mmol, 2.5 equiv.), CuOTf·0.5PhMe (8 mg, 0.03 mmol, 10 mol%) in degassed $PhCF_3$/MeCN (3 mL, 3 : 1 v/v). The test-tube was capped, and the reaction mixture was irradiated with LED light (λ = 400 nm) while stirring at room temperature for 12 h. The reaction mixture was treated with saturated solution of sodium carbonate (3 mL) and extracted with DCM (3 × 10 mL). The organic layer was collected, dried over anhydrous sodium sulfate, concentrated under reduced pressure, and the remaining material was purified by flash chromatography on silica gel (hexane/ ethyl acetate 4 : 1 v/v) to give the sulfonyl azide product **9a** (73 mg, 90%) as a colorless oil.

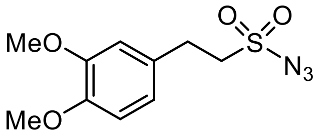

^{1}H NMR (500 MHz, $CDCl_3$): 6.83 (1 H, d, *J* = 8.2 Hz), 6.76 (1 H, dd, *J* = 8.2, 2.1 Hz), 6.72 (1 H, d, *J* = 2.1 Hz), 3.87 (6 H, d, *J* = 8.5 Hz), 3.66–3.52 (2 H, m), 3.21–3.11 (2 H, m) ppm. – ^{13}C NMR (125 MHz, $CDCl_3$): 149.5, 148.5 128.8, 120.5, 111.7, 111.6, 57.4, 56.1, 29.3 ppm. – IR: 2983, 2941, 2907, 2876, 1737, 1478, 1446, 1372, 1237, 1045 cm^{-1}. – HRMS: calcd for $C_{10}H_{13}N_3O_4SNa$: 294.0519, found 294.0514 [M+Na$^+$].

Evaluation Only. Created with Aspose.Pdf. Copyright 2002-2014 Aspose Pty Ltd.

4-Oxo-4-phenylbutane-1-sulfonyl azide (9b)

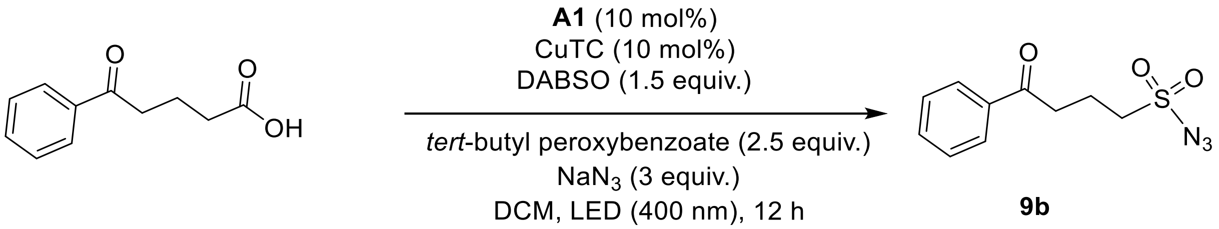

According to **GP3**, the reaction was carried out with 5-oxo-5-phenylpentanoic acid (58 mg, 0.3 mmol), DABSO (108 mg, 0.45 mmol, 1.5 equiv.), acridine catalyst **A1** (9 mg, 0.03 mmol, 10 mol%), sodium azide (59 mg, 0.9 mmol, 3.0 equiv.), *tert*-butyl peroxybenzoate (145 mg, 0.75 mmol, 2.5 equiv.), copper(I) thiophene-2-carboxylate (5 mg, 0.03 mmol, 10 mol%) in degassed $PhCF_3$/MeCN (3 mL, 3 : 1 v/v). The test-tube was capped, and the reaction mixture was irradiated with LED light (λ = 400 nm) while stirring at room temperature for 12 h. The reaction mixture was treated with saturated solution of sodium carbonate (3 mL) and extracted with DCM (3 × 10 mL). The organic layer was collected, dried over anhydrous sodium sulfate, concentrated under reduced pressure, and the remaining material was purified by flash chromatography on silica gel (hexane/ ethyl acetate 7 : 3 v/v) to give the sulfonyl azide product **9b** (51 mg, 67%) as a colorless solid.

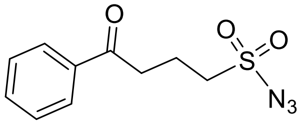

m.p.: 58–60°C. – ^{1}H NMR (500 MHz, $CDCl_3$): 7.98–7.93 (2 H, m), 7.63–7.56 (1 H, m), 7.48 (2 H, t, *J* = 7.7 Hz), 3.50 (2 H, dd, *J* = 8.0, 6.6 Hz), 3.25 (2 H, t, *J* = 6.5 Hz), 2.42–2.33 (2 H, m) ppm. – ^{13}C NMR (125 MHz, $CDCl_3$): 198.1, 136.4, 133.7, 128.9, 128.1, 55.0, 35.7, 18.1 ppm. – IR: 2136, 1683, 1597, 1449, 1361, 1251, 1226, 1192, 1158 cm^{-1}. – HRMS: calcd for $C_{10}H_{11}N_3O_3SNa$: 276.0413, found 276.0413 [M+Na$^+$].

Go back to table of contents

Evaluation Only. Created with Aspose.Pdf. Copyright 2002-2014 Aspose Pty Ltd.

Methyl 5-(azidosulfonyl)pentanoate (9c)

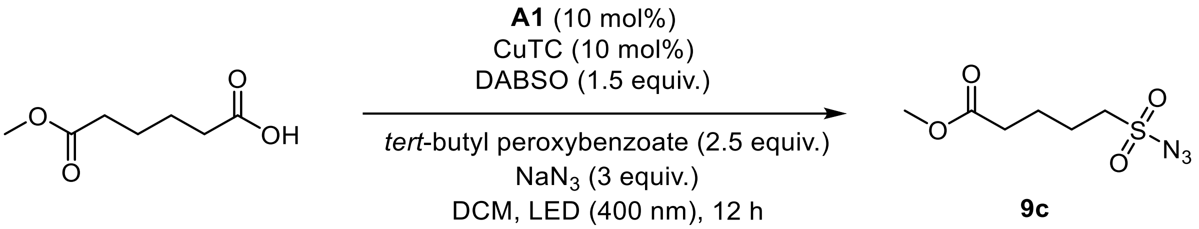

According to **GP3**, the reaction was carried out with 6-methoxy-6-oxohexanoic acid (58 mg, 0.3 mmol), DABSO (108 mg, 0.45 mmol, 1.5 equiv.), acridine catalyst **A1** (9 mg, 0.03 mmol, 10 mol%), sodium azide (59 mg, 0.9 mmol, 3.0 equiv.), *tert*-butyl peroxybenzoate (145 mg, 0.75 mmol, 2.5 equiv.), copper(I) thiophene-2-carboxylate (5 mg, 0.03 mmol, 10 mol%) in degassed $PhCF_3$/MeCN (3 mL, 3 : 1 v/v). The test-tube was capped, and the reaction mixture was irradiated with LED light (λ = 400 nm) while stirring at room temperature for 12 h. The reaction mixture was treated with saturated solution of sodium carbonate (3 mL) and extracted with DCM (3 × 10 mL). The organic layer was collected, dried over anhydrous sodium sulfate, concentrated under reduced pressure, and the remaining material was purified by flash chromatography on silica gel (hexane/ ethyl acetate 9 : 1 v/v) to give the sulfonyl azide product **9c** (50 mg, 75%) as a colorless solid.

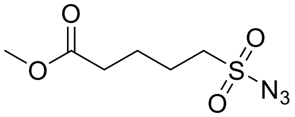

^{1}H NMR (500 MHz, $CDCl_3$): 3.67 (3 H, s), 3.36–3.30 (2 H, m), 2.38 (2 H, t, *J* = 7.2 Hz), 2.00–1.88 (2 H, m), 1.85–1.73 (2 H, m) ppm. – ^{13}C NMR (125 MHz, $CDCl_3$): 173.1, 55.6, 51.9, 33.2, 23.3, 23.0 ppm. – IR: 2990, 2956, 2927, 2854, 2331, 2139, 1736, 1486, 1371, 1267, 1204, 1060, 898, 793 cm^{-1}. – HRMS: calcd for $C_6H_{11}N_3NaO_4S$: 244.0362, found 244.0363 [M+Na$^+$].

Evaluation Only. Created with Aspose.Pdf. Copyright 2002-2014 Aspose Pty Ltd.

4-(5-Methylthiophen-2-yl)-4-oxobutane-1-sulfonyl azide (9d)

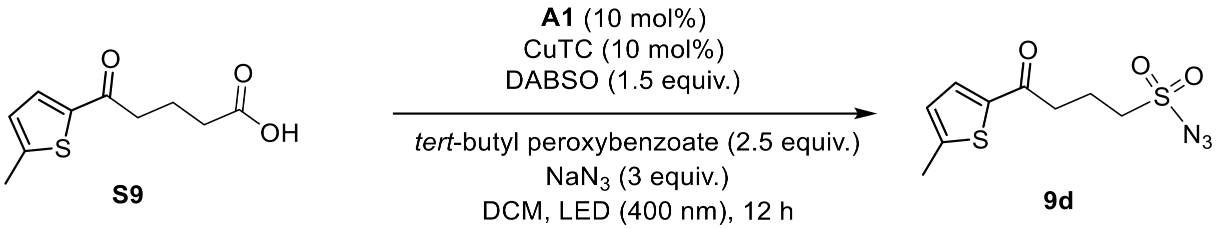

S9 **9d**

According to **GP3**, the reaction was carried out with acid **S9** (64 mg, 0.3 mmol), DABSO (108 mg, 0.45 mmol, 1.5 equiv.), acridine catalyst **A1** (9 mg, 0.03 mmol, 10 mol%), sodium azide (59 mg, 0.9 mmol, 3.0 equiv.), *tert*-butyl peroxybenzoate (145 mg, 0.75 mmol, 2.5 equiv.), copper(I) thiophene-2-carboxylate (5 mg, 0.03 mmol, 10 mol%) in degassed $PhCF_3$/MeCN (3mL, 3 : 1 v/v). The test-tube was capped, and the reaction mixture was irradiated with LED light (λ = 400 nm) while stirring at room temperature for 12 h. The reaction mixture was treated with saturated solution of sodium carbonate (3 mL) and extracted with DCM (3 × 10 mL). The organic layer was collected, dried over anhydrous sodium sulfate, concentrated under reduced pressure, and the remaining material was purified by flash chromatography on silica gel (hexane/ ethyl acetate 7 : 3 v/v) to give the sulfonyl azide product **9d** (59 mg, 72%) as a colorless solid.

Gramscale synthesis of compound 14d: According to **GP3**, the reaction was carried out with acid **S9** (1.49 g, 7 mmol), DABSO (2.52 g, 10.5 mmol, 1.5 equiv.), acridine catalyst **A2** (210 mg, 0.07 mmol, 10 mol%), sodium azide (1.38 g, 21 mmol, 3.0 equiv.), *tert*-butyl peroxybenzoate (3.38 g, 17.5 mmol, 2.5 equiv.), copper(I) thiophene-2-carboxylate (98 mg, 0.07 mmol, 10 mol%) in degassed $PhCF_3$/MeCN (70 mL, 3 : 1 v/v). The test-tube was capped, and the reaction mixture was irradiated with LED light (λ = 400 nm) while stirring at room temperature for 12 h. The reaction mixture was treated with saturated solution of sodium carbonate (20 mL) and extracted with DCM (3 × 50 mL). The organic layer was collected, dried over anhydrous sodium sulfate, concentrated under reduced pressure, and the remaining material was purified by flash chromatography on silica gel

Evaluation Only. Created with Aspose.Pdf. Copyright 2002-2014 Aspose Pty Ltd.

(hexane/ ethyl acetate 7 : 3 v/v) to give the sulfonyl azide product **9d** (1.05 mg, 55%) as a colorless solid.

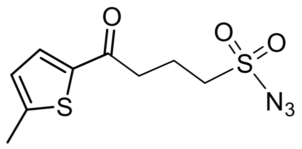

m.p.: 53–55°C. – ^{1}H NMR (500 MHz, $CDCl_3$): 7.55 (1 H, d, *J* = 3.8 Hz), 6.81 (1 H, dd, *J* = 3.8, 1.1 Hz), 3.48 (2 H, dd, *J* = 8.0, 6.6 Hz), 3.11 (2 H, t, *J* = 6.7 Hz), 2.54 (3 H, d, *J* = 0.9 Hz), 2.38–2.29 (2 H, m) ppm. – ^{13}C NMR (125 MHz, $CDCl_3$): 190.5, 150.6, 141.3, 133.0, 127.1, 55.0, 35.8, 18.4, 16.2 ppm. – IR: 2922, 2134, 1655, 1455, 1360, 1241, 1158, 1072 808 cm^{-1}. – HRMS: calcd for $C_9H_{11}N_3O_3S_2Na$: 296.0134, found 296.0134 [M+Na$^+$].

4-Oxo-4-((3-(4,4,5,5-tetramethyl-1,3,2-dioxaborolan-2-yl)phenyl)amino)butane-1-sulfonyl azide (9e)

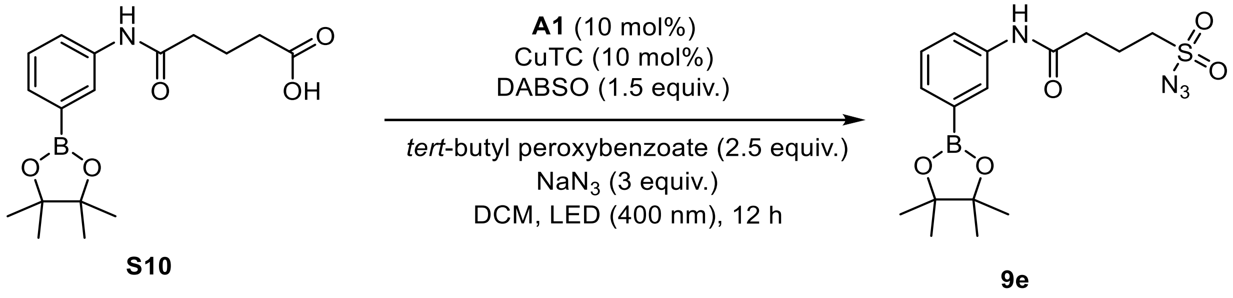

According to **GP3**, the reaction was carried out with acid **S10** (50 mg, 0.15 mmol), DABSO (54 mg, 0.23 mmol, 1.5 equiv.), acridine catalyst **A1** (4.5 mg, 0.015 mmol, 10 mol%), sodium azide (30 mg, 0.45 mmol, 3.0 equiv.), *tert*-butyl peroxybenzoate (73 mg, 0.38 mmol, 2.5 equiv.), copper(I) thiophene-2-carboxylate (5 mg, 0.03 mmol, 10 mol%) in degassed $PhCF_3$/MeCN (3 mL, 3 : 1 v/v). The test-tube was capped, and the reaction mixture was irradiated with LED light (λ = 400 nm) while stirring at room temperature for 12 h. The reaction mixture was treated with saturated solution of sodium carbonate (3 mL) and extracted with DCM (3 × 10 mL). The organic layer was collected, dried over anhydrous sodium sulfate, concentrated under reduced pressure, and the remaining

Evaluation Only. Created with Aspose.Pdf. Copyright 2002-2014 Aspose Pty Ltd.

material was purified by flash chromatography on silica gel (hexane/ ethyl acetate 2 : 1 v/v) to give the sulfonyl azide product **9e** (49 mg, 83%) as a colorless solid.

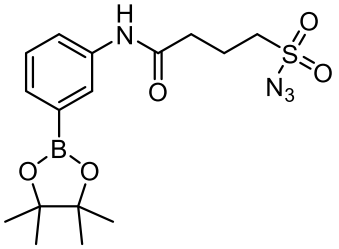

m.p.: 102–105°C. – ^{1}H NMR (500 MHz, $CDCl_3$): 7.77 (1 H, dt, *J* = 8.1, 1.6 Hz), 7.71 (1 H, d, *J* = 2.4 Hz), 7.56 (1 H, d, *J* = 7.3 Hz), 7.43–7.29 (2 H, m), 3.50 (2 H, t, *J* = 7.1 Hz), 2.59 (2 H, t, *J* = 6.8 Hz), 2.32 (2 H, p, *J* = 7.0 Hz), 1.33 (12 H, s) ppm. – ^{13}C NMR (125 MHz, $CDCl_3$): 169.1, 137.1, 131.1, 128.8, 126.0, 123.2, 84.1, 54.8, 34.2, 25.0, 19.4 ppm. – ^{11}B NMR (160 MHz, $CDCl_3$): –5.1 ppm. – IR: 3291, 2977, 2135, 1667, 1584, 1552, 1428, 1357, 1318, 1268, 1191, 1159, 1143, 1076, 1027 cm^{-1}. – HRMS: calcd for $C_{16}H_{23}BN_4O_4SNa$: 417.1377, found 417.1372 [M+Na^{+}].

5-(4-Chlorobenzamido)pentane-1-sulfonyl azide (9f)

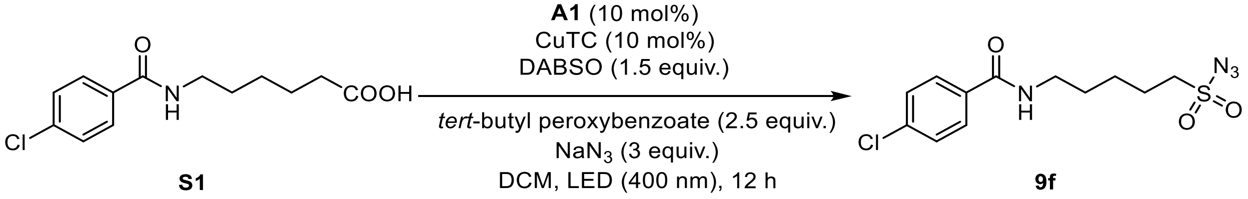

According to **GP3**, the reaction was carried out with acid **S1** (81 mg, 0.3 mmol), DABSO (108 mg, 0.45 mmol, 1.5 equiv.), acridine catalyst **A1** (9 mg, 0.03 mmol, 10 mol%), potassium azide (73 mg, 0.9 mmol, 3.0 equiv.), *tert*-butyl peroxybenzoate (145 mg, 0.75 mmol, 2.5 equiv.), copper(I) thiophene-2-carboxylate (5 mg, 0.03 mmol, 10 mol%) in degassed $PhCF_3$/MeCN (3 mL, 3 : 1 v/v). The test-tube was capped, and the reaction mixture was irradiated with LED light (λ = 400 nm) while stirring at room temperature for 12 h. The reaction mixture was treated with saturated solution of sodium carbonate (3 mL) and extracted with DCM (3 × 10 mL). The organic layer was collected, dried over anhydrous sodium sulfate, concentrated under reduced pressure, and the remaining material was purified by flash chromatography on silica gel (hexane/ ethyl acetate 1 : 1 v/v) to give the sulfonyl azide product **9f** (61 mg, 62%) as a colorless oil.

Evaluation Only. Created with Aspose.Pdf. Copyright 2002-2014 Aspose Pty Ltd.

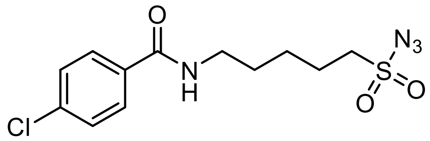

^{1}H NMR (500 MHz, $CDCl_3$): 7.69 (2 H, d, *J* = 8.5 Hz), 7.40 (2 H, d, *J* = 8.5 Hz), 6.27 (1 H, s), 3.46 (2 H, q, *J* = 6.7 Hz), 3.39–3.27 (2 H, m), 2.07–1.86 (2 H, m), 1.77–1.46 (4 H, m) ppm. – ^{13}C NMR (125 MHz, $CDCl_3$): 166.7, 137.9, 133.0, 129.0, 128.4, 55.8, 39.7, 29.2, 25.4, 23.2 ppm. – IR: 2983, 2942, 2908, 2256, 2030, 1885, 1736, 1558, 1465, 1446, 1393, 1372, 1300, 1235, 1097, 1044, 938, 917 cm^{-1}. – HRMS: calcd for $C_{12}H_{15}ClN_4O_3SNa$: 353.0446, found 353.0444 [M+Na$^+$].

Pentadecane-1-sulfonyl azide (9g)

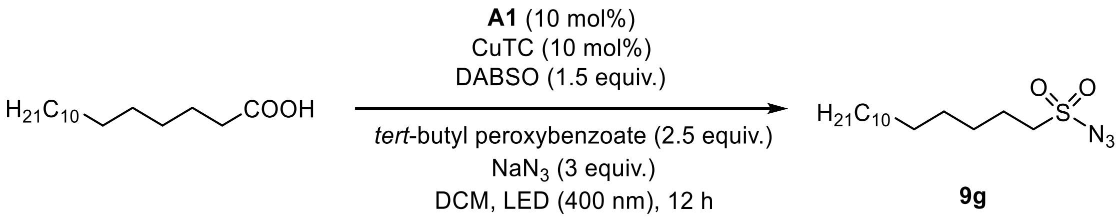

According to **GP3**, the reaction was carried out with palmitic acid (77 mg, 0.3 mmol), DABSO (108 mg, 0.45 mmol, 1.5 equiv.), acridine catalyst **A1** (9 mg, 0.03 mmol, 10 mol%), potassium azide (73 mg, 0.9 mmol, 3.0 equiv.), *tert*-butyl peroxybenzoate (145 mg, 0.75 mmol, 2.5 equiv.), copper(I) thiophene-2-carboxylate (5 mg, 0.03 mmol, 10 mol%) in degassed $PhCF_3$/MeCN (3 mL, 3 : 1 v/v). The test-tube was capped, and the reaction mixture was irradiated with LED light (λ = 400 nm) while stirring at room temperature for 12 h. The reaction mixture was treated with saturated solution of sodium carbonate (3 mL) and extracted with DCM (3 × 10 mL). The organic layer was collected, dried over anhydrous sodium sulfate, concentrated under reduced pressure, and the remaining material was purified by flash chromatography on silica gel (hexane/ ethyl acetate 9 : 1 v/v) to give the sulfonyl azide product **9g** (71 mg, 75%) as a white solid.

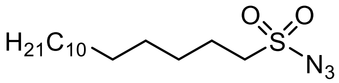

m.p.: 73–75°C. – ^{1}H NMR (500 MHz, $CDCl_3$): 3.41–3.25 (2 H, m), 1.99–1.78 (2 H, m), 1.51–1.40 (2 H, m), 1.35–1.15 (22 H, m), 0.88

Go back to table of contents

Evaluation Only. Created with Aspose.Pdf. Copyright 2002-2014 Aspose Pty Ltd.

(2 H, t, J = 6.9 Hz) ppm. – ^{13}C NMR (125 MHz, $CDCl_3$): 56.1, 32.1, 29.8, 29.8, 29.8, 29.7, 29.6, 29.5, 29.3, 29.1, 28.1, 23.5, 22.8, 14.3 ppm. – IR: 3466, 2984, 2941, 2908, 2877, 2256, 1982, 1736, 1464, 1392, 1300, 1233, 1097, 1043 cm^{-1}. – HRMS: calcd for $C_{15}H_{32}N_3O_2S$: 318.2215, found 318.2214 [M+H$^+$].

tert-Butyl 4-(azidosulfonyl)piperidine-1-carboxylate (9h)

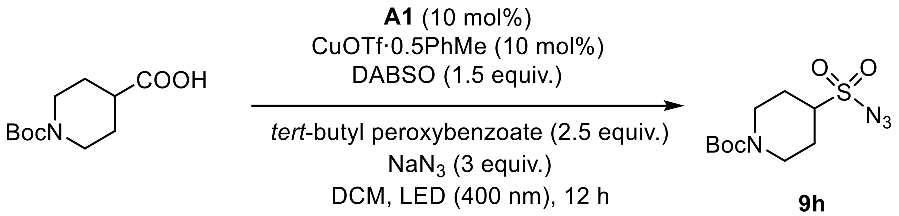

9h

According to **GP3**, the reaction was carried out with 1-(*tert*-butoxycarbonyl)piperidine-4-carboxylic acid (69 mg, 0.3 mmol), DABSO (108 mg, 0.45 mmol, 1.5 equiv.), acridine catalyst **A1** (9 mg, 0.03 mmol, 10 mol%), potassium azide (73 mg, 0.9 mmol, 3.0 equiv.), *tert*-butyl peroxybenzoate (145 mg, 0.75 mmol, 2.5 equiv.), CuOTf·0.5PhMe (8 mg, 0.03 mmol, 10 mol%) in degassed $PhCF_3$/MeCN (3 mL, 3 : 1 v/v). The test-tube was capped, and the reaction mixture was irradiated with LED light (λ = 400 nm) while stirring at room temperature for 12 h. The reaction mixture was treated with saturated solution of sodium carbonate (3 mL) and extracted with DCM (3 × 10 mL). The organic layer was collected, dried over anhydrous sodium sulfate, concentrated under reduced pressure, and the remaining material was purified by flash chromatography on silica gel (hexane/ethyl acetate 4 : 1 v/v) to give the sulfonyl azide product **9h** (48 mg, 55%) as a colorless oil.

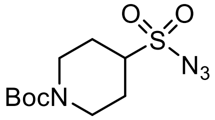

^{1}H NMR (500 MHz, $CDCl_3$): 4.30 (2 H, s), 3.34 (1 H, tt, J = 12.0, 3.7 Hz), 2.89–2.68 (2 H, m), 2.23–2.04 (2 H, m), 1.81 (2 H, qd, J = 12.4, 4.6 Hz), 1.46 (9 H, s) ppm. – ^{13}C NMR (125 MHz, $CDCl_3$): 154.3, 80.6, 63.7, 42.5, 28.5, 25.8 ppm. – IR:

Evaluation Only. Created with Aspose.Pdf. Copyright 2002-2014 Aspose Pty Ltd.

2983, 2941, 2908, 2515, 2203, 1741, 1465, 1447, 1373, 1300, 1241, 1046, 917 cm^{-1}. – HRMS: calcd for $C_{10}H_{18}N_4O_4SNa$: 313.0941, found 313.0939 [$M+Na^+$].

Tetrahydro-2*H*-pyran-4-sulfonyl azide (9i)

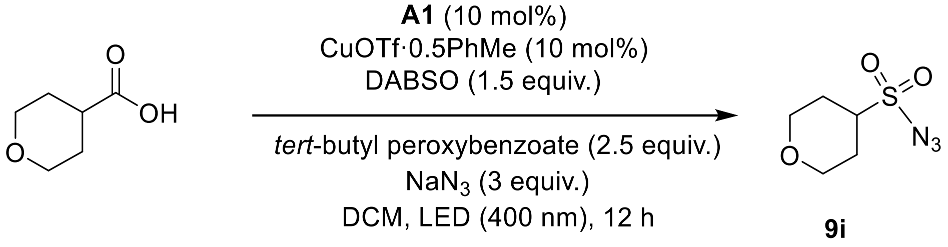

According to **GP3**, the reaction was carried out with tetrahydro-2*H*-pyran-4-carboxylic acid (39 mg, 0.3 mmol), DABSO (108 mg, 0.45 mmol, 1.5 equiv.), acridine catalyst **A1** (9 mg, 0.03 mmol, 10 mol%), sodium azide (59 mg, 0.9 mmol, 3.0 equiv.), *tert*-butyl peroxybenzoate (145 mg, 0.75 mmol, 2.5 equiv.), CuOTf·0.5PhMe (8 mg, 0.03 mmol, 10 mol%) in degassed $PhCF_3$/MeCN (3 mL, 3 : 1 v/v). The test-tube was capped, and the reaction mixture was irradiated with LED light (λ = 400 nm) while stirring at room temperature for 12 h. The reaction mixture was treated with saturated solution of sodium carbonate (3 mL) and extracted with DCM (3 × 10 mL). The organic layer was collected, dried over anhydrous sodium sulfate, concentrated under reduced pressure, and the remaining material was purified by flash chromatography on silica gel (hexane/ ethyl acetate 4 : 1 v/v) to give the sulfonyl azide product **9i** (37 mg, 65%) as a colorless oil.

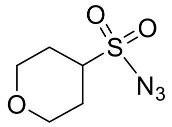

^{1}H NMR (500 MHz, $CDCl_3$): 4.13 (2 H, ddd, *J* = 12.0, 4.8, 1.9 Hz), 3.50–3.37 (3 H, m), 2.12 (2 H, ddq, *J* = 12.8, 4.2, 2.2 Hz), 1.99 (2 H, dtd, *J* = 13.1, 11.8, 4.7 Hz) ppm. – ^{13}C NMR (125 MHz, $CDCl_3$): 66.3, 62.5, 26.4 ppm. – IR: 2933, 2855, 2133, 1732, 1449, 1361, 1306, 1240, 1195, 1155, 1108, 1083, 1023, 1008 cm^{-1}. – HRMS: calcd for $C_5H_{11}N_3O_3S$: 192.0443, found 192.0438 [$M+H^+$].

Evaluation Only. Created with Aspose.Pdf. Copyright 2002-2014 Aspose Pty Ltd.

4-Methyltetrahydro-2*H*-pyran-4-sulfonyl azide (9j)

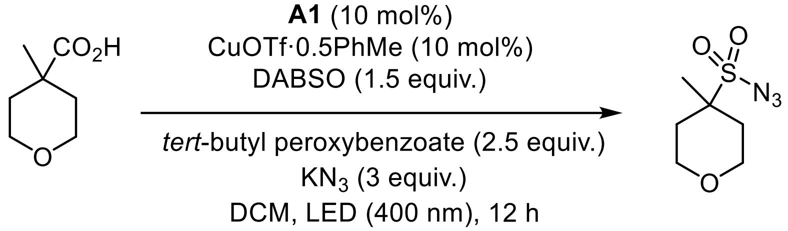

9j

According to **GP3**, the reaction was carried out with 4-methyltetrahydro-2*H*-pyran-4-carboxylic acid (43 mg, 0.3 mmol), DABSO (108 mg, 0.45 mmol, 1.5 equiv.), acridine catalyst **A1** (9 mg, 0.03 mmol, 10 mol%), potassium azide (73 mg, 0.9 mmol, 3.0 equiv.), *tert*-butyl peroxybenzoate (145 mg, 0.75 mmol, 2.5 equiv.), CuOTf·0.5PhMe (8 mg, 0.03 mmol, 10 mol%) in degassed $PhCF_3$/MeCN (3 mL, 3 : 1 v/v). The test-tube was capped, and the reaction mixture was irradiated with LED light (λ = 400 nm) while stirring at room temperature for 12 h. The reaction mixture was treated with saturated solution of sodium carbonate (3 mL) and extracted with DCM (3 × 10 mL). The organic layer was collected, dried over anhydrous sodium sulfate, concentrated under reduced pressure, and the remaining material was purified by flash chromatography on silica gel (hexane/ ethyl acetate 4 : 1 v/v) to give the sulfonyl azide product **9j** (31 mg, 50%) as a colorless oil.

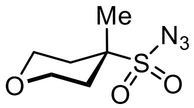

^{1}H NMR (500 MHz, $CDCl_3$): 4.00 (2 H, ddd, *J* = 12.1, 5.2, 2.5 Hz), 3.56 (2 H, td, *J* = 11.8, 2.4 Hz), 2.32 (2 H, ddd, *J* = 13.1, 11.3, 5.0 Hz), 1.71 (2 H, dq, *J* = 13.2, 2.5 Hz), 1.64 (3 H, s) ppm. – ^{13}C NMR (125 MHz, $CDCl_3$): 65.7, 63.1, 30.9, 17.8 ppm. – IR: 2865, 2133, 1693, 1475, 1392, 1344, 1266, 1223, 1174, 1144, 1104, 1038, 1010 cm^{-1}. – HRMS: calcd for $C_6H_{12}N_3O_3S$: 206.0599, found 206.0606 [M+H$^+$].

Go back to table of contents

Evaluation Only. Created with Aspose.Pdf. Copyright 2002-2014 Aspose Pty Ltd.

Heptane-4-sulfonyl azide (9k)

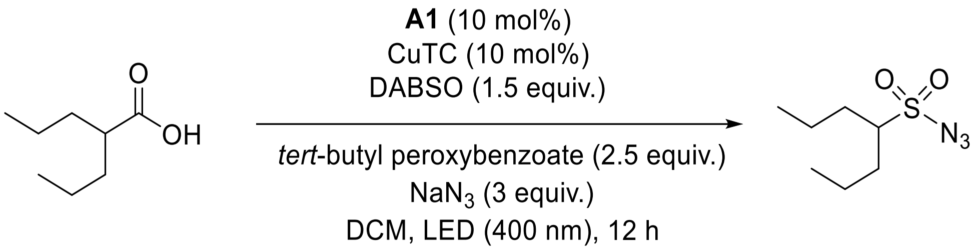

9k

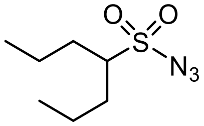

According to **GP3**, the reaction was carried out with 2-propylpentanoic acid (43 mg, 0.3 mmol), DABSO (108 mg, 0.45 mmol, 1.5 equiv.), acridine catalyst **A1** (9 mg, 0.03 mmol, 10 mol%), potassium azide (73 mg, 0.9 mmol, 3.0 equiv.), *tert*-butyl peroxybenzoate (145 mg, 0.75 mmol, 2.5 equiv.), CuOTf·0.5PhMe (8 mg, 0.03 mmol, 10 mol%) in degassed dichloromethane (3 mL). The test-tube was capped, and the reaction mixture was irradiated with LED light (λ = 400 nm) while stirring at room temperature for 12 h. The reaction mixture was treated with saturated solution of sodium carbonate (3 mL) and extracted with DCM (3 × 10 mL). The organic layer was collected, dried over anhydrous sodium sulfate, concentrated under reduced pressure, and the remaining material was purified by flash chromatography on silica gel (hexane/ ethyl acetate 9 : 1 v/v) to give the sulfonyl azide product **9k** (40 mg, 65%) as a colorless oil.

^{1}H NMR (500 MHz, DMSO): 3.20 (1 H, tt, *J* = 7.0, 5.1 Hz), 1.95 (2 H, dddd, *J* = 14.3, 10.1, 6.1, 5.1 Hz), 1.74 (2 H, dddd, *J* = 14.3, 10.2, 7.0, 5.5 Hz), 1.62–1.43 (4 H, m), 0.97 (6 H, t, *J* = 7.4 Hz) ppm. – ^{13}C NMR (125 MHz, DMSO): 66.8, 31.0, 20.0, 14.0 ppm. – IR: 3054, 3015, 2988, 2969, 2876, 2252, 2134, 1738, 1365, 1265, 1228, 1216, 1205, 908 cm^{-1}. – HRMS: calcd for $C_7H_{16}N_3O_2S$: 206.0963, found 206.0961 [M+H$^+$].

Go back to table of contents

Evaluation Only. Created with Aspose.Pdf. Copyright 2002-2014 Aspose Pty Ltd.

(3*R*,7*R*,8*R*,9*S*,10*S*,13*R*,14*S*,17*R*)-10,13-Dimethyl-17-((*R*)-4-(morpholinosulfonyl)butan-2-yl)hexadecahydro-1*H*-cyclopenta[*a*]phenanthrene-3,7-diyl diacetate (10a)

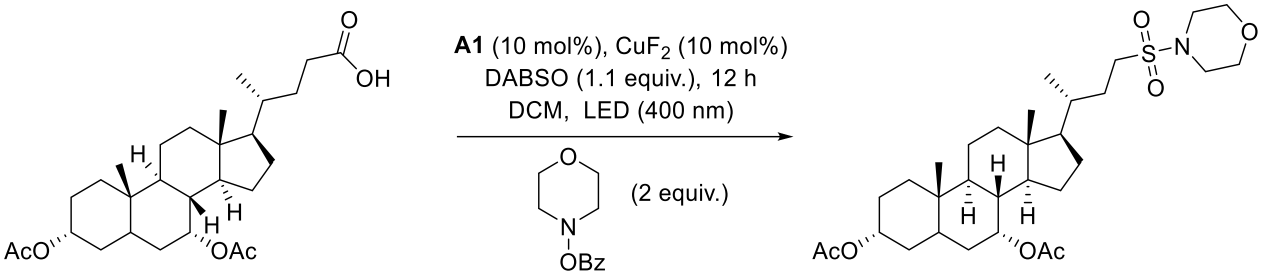

S12 **10a**

According to **GP1**, the reaction was carried out with **S12** (71 mg, 0.3 mmol), DABSO (40 mg, 0.165 mmol, 1.1 equiv.), *N*-(benzoyloxy)morpholine (62 mg, 0.3 mmol, 2 equiv.), acridine catalyst **A1** (5 mg, 0.015 mmol, 10 mol%), copper difluoride (1.5 mg, 0.015 mmol, 10 mol%), and degassed dichloromethane (6 mL). The test-tube was capped and the reaction mixture was irradiated with LED light (λ = 400 nm) while stirring for 12 h. The reaction mixture was then quenched with saturated solution of sodium hydrogen carbonate (3 mL) and extracted with ethyl acetate (3 × 10 mL). The organic layer was collected, dried over anhydrous sodium sulfate, concentrated under reduced pressure, and the remaining material was purified by flash chromatography on silica gel (hexane/ ethyl acetate 2 : 3 v/v) to give the sulfone product **10a** (52 mg, 60%) as a colorless solid.

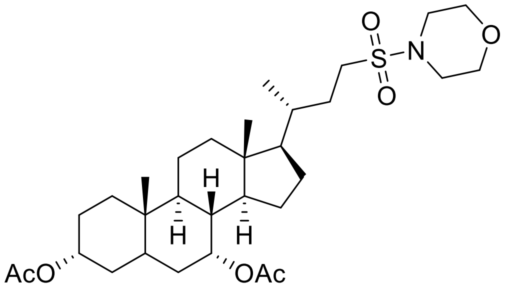

m.p.: 107–110 °C. – $[\alpha]_D^{23}$ = + 20.0 (c 0.1, $CHCl_3$). – ^1H NMR (500 MHz, $CDCl_3$): 4.86 (1 H, q, *J* = 3.1 Hz), 4.57 (1 H, tt, *J* = 11.4, 4.5 Hz), 3.76–3.70 (4 H, m), 3.28–3.22 (4 H, m), 2.96 (1 H, ddd, *J* = 13.6, 11.8, 4.2 Hz), 2.81 (1 H, ddd, *J* = 13.5, 11.0, 4.9 Hz), 2.09–1.99 (7 H, m), 1.98–1.78 (5 H, m), 1.74–1.66 (1 H, m), 1.63–1.21 (12 H, m), 1.21–1.00 (4 H, m), 0.98–0.81 (7 H, m), 0.65 (3 H, s) ppm. – ^{13}C NMR (125 MHz, $CDCl_3$): 170.7, 170.5, 74.2, 71.3, 66.8, 55.6, 50.5, 46.6, 46.0, 42.9, 41.0, 39.6, 38.0, 35.1, 35.0, 34.9, 34.7, 34.2, 31.7, 31.4, 28.9, 28.2, 26.9, 23.6, 22.8, 21.7, 21.6, 20.7, 18.5, 11.9 ppm. – IR: 2974, 2936, 2865, 1725, 1447, 1375,

Evaluation Only. Created with Aspose.Pdf. Copyright 2002-2014 Aspose Pty Ltd.

1362, 1342, 1232, 1141, 1112, 1067, 1019. 848, 795, 731 cm^{-1}. – HRMS: calcd for $C_{31}H_{51}NNaO_7S$: 604.3278, found 604.3283 [M+H$^+$].

15-(Morpholinosulfonyl)pentadecane-1,7,8-triyl triacetate (10b)

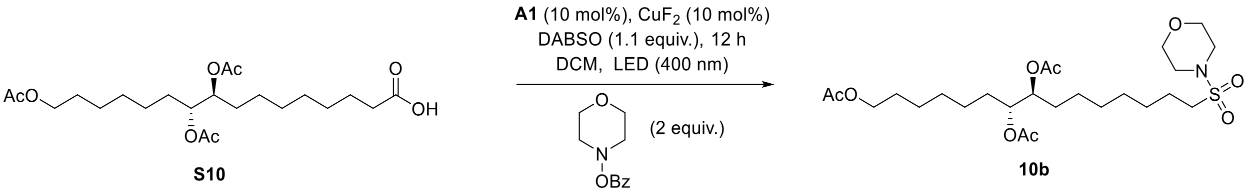

According to **GP1**, the reaction was carried out with acid **S10** (65 mg, 0.3 mmol), DABSO (40 mg, 0.165 mmol, 1.1 equiv.), *N*-(benzoyloxy)morpholine (62 mg, 0.3 mmol, 2 equiv.), acridine catalyst **A1** (5 mg, 0.015 mmol, 10 mol%), copper difluoride (1.5 mg, 0.015 mmol, 10 mol%), and degassed dichloromethane (6 mL). The test-tube was capped and the reaction mixture was irradiated with LED light (λ = 400 nm) while stirring for 12 h. The reaction mixture was then quenched with saturated solution of sodium hydrogen carbonate (3 mL) and extracted with ethyl acetate (3 × 10 mL). The organic layer was collected, dried over anhydrous sodium sulfate, concentrated under reduced pressure, and the remaining material was purified by flash chromatography on silica gel (hexane/ ethyl acetate 1 : 1 v/v) to give the sulfone product **10b** (52 mg, 65%) as a colorless liquid.

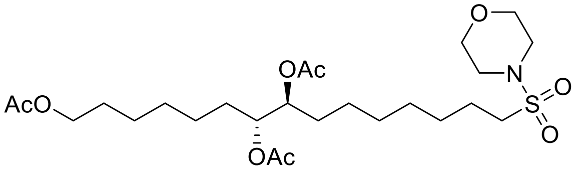

^{1}H NMR (500 MHz, $CDCl_3$): 4.99–4.89 (2 H, m), 4.00 (2 H, td, *J* = 6.7, 1.4 Hz), 3.74–3.69 (4 H, m), 3.24–3.20 (4 H, m), 2.90–2.79 (2 H, m,), 2.05 (6 H, s), 2.00 (3 H, s), 1.81–1.70 (2 H, m), 1.57 (2 H, quint., *J* = 6.9 Hz), 1.52–1.15 (18 H, .m) ppm. – ^{13}C NMR (125 MHz, $CDCl_3$): 171.2, 170.7, 73.7, 66.7, 64.5, 48.8, 45.9, 30.7, 29.1, 28.9, 28.5, 28.4, 25.8, 25.14, 25.11, 22.9, 21.1, 21.0 ppm. – IR: 3063, 3003, 2964, 2933, 1711, 1418, 1361, 1270, 1221, 1188, 1152, 1091, 961, 901, 736, 703 cm^{-1}. – HRMS: calcd for $C_{25}H_{45}NNaO_9S$: 558.2707, found 558.2719 [M+H$^+$].

Evaluation Only. Created with Aspose.Pdf. Copyright 2002-2014 Aspose Pty Ltd.

(1*S*,2*S*,4a*R*,4b*R*,7*S*,9a*S*,10*S*,10a*R*)-1-Methyl-8-methylene-10-(morpholinosulfonyl)-13-oxo-1,2,5,6,8,9,10,10a-octahydro-4a,1-(epoxymethano)-7,9a-methanobenzo[*a*]azulene-2,7(4b*H*)-diyl diacetate (10c)

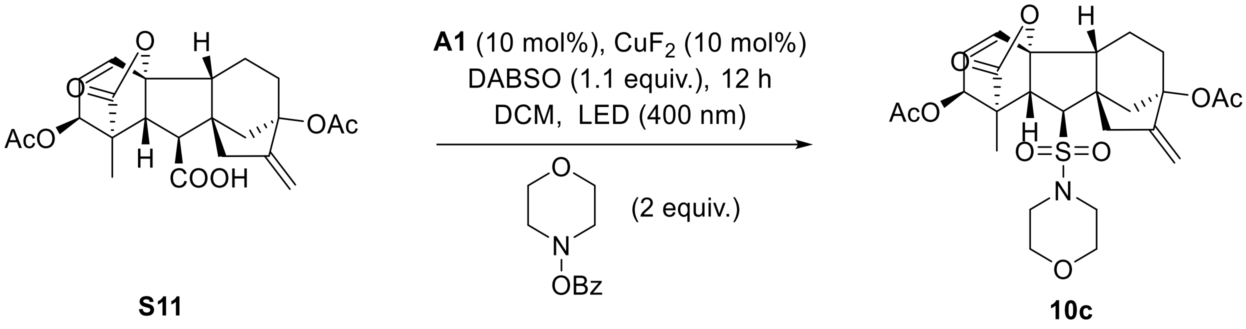

According to **GP1**, the reaction was carried out with **S11** (71 mg, 0.3 mmol), DABSO (40 mg, 0.165 mmol, 1.1 equiv.), *N*-(benzoyloxy)morpholine (62 mg, 0.3 mmol, 2 equiv.), acridine catalyst **A1** (5 mg, 0.015 mmol, 10 mol%), copper difluoride (1.5 mg, 0.015 mmol, 10 mol%), and degassed dichloromethane (6 mL). The test-tube was capped and the reaction mixture was irradiated with LED light (λ = 400 nm) while stirring for 12 h. The reaction mixture was then quenched with saturated solution of sodium hydrogen carbonate (3 mL) and extracted with ethyl acetate (3 × 10 mL). The organic layer was collected, dried over anhydrous sodium sulfate, concentrated under reduced pressure, and the remaining material was purified by flash chromatography on silica gel (hexane/ ethyl acetate 1 : 4 v/v) to give the sulfone product **10c** (72 mg, 89%) as a colorless solid.

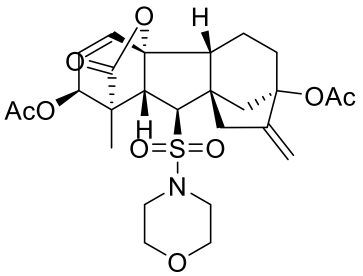

m.p.: 157–160 °C. – $[\alpha]_D^{23}$ = + 81.8 (c 0.55, $CHCl_3$). – ^1H NMR (500 MHz, $CDCl_3$): 6.45 (1 H, d, *J* = 9.3 Hz), 5.87 (1 H, dd, *J* = 9.2, 3.7 Hz), 5.41 (1 H, d, *J* = 3.7 Hz), 5.07 (1 H, d, *J* = 2.8 Hz), 5.00 (1 H, dd, *J* = 3.4, 1.5 Hz), 3.87–3.62 (5 H, m), 3.43–3.26 (5 H, m), 3.26–3.19 (2 H, m), 2.79 (1 H, s), 2.63–2.53 (2 H, m), 2.22 (1 H, dd, *J* = 10.3, 3.1 Hz), 2.17–2.08 (4 H, m), 2.04–1.93 (4 H, m), 1.72 (1 H, ddt, *J* = 12.1, 6.2, 2.8 Hz), 1.33 (3 H, s) ppm. – ^{13}C NMR (125 MHz, $CDCl_3$): 176.7, 169.8, 169.6, 149.5, 133.9, 129.5, 106.4, 90.1, 83.9, 71.8, 67.0, 66.4, 64.8, 54.0, 52.3, 52.1, 47.1, 46.4, 46.0, 45.0, 41.5,

Go back to table of contents

Evaluation Only. Created with Aspose.Pdf. Copyright 2002-2014 Aspose Pty Ltd.

36.3, 22.2, 21.0, 17.9, 15.3 ppm. – IR: 2957, 2921, 2867, 2850, 1652, 1455, 1408, 1361, 1334, 1260, 1240, 1161, 1128, 1107, 1073, 945, 913, 873, 802, 785, 770, 710 cm^{-1}. – HRMS: calcd for $C_{26}H_{33}NNaO_9S$: 558.1768, found 558.1767 [$M+Na^+$].

N-(4-(3-Ethyl-2,6-dioxopiperidin-3-yl)phenyl)-4-oxo-4-phenylbutane-1-sulfonamide (10d)

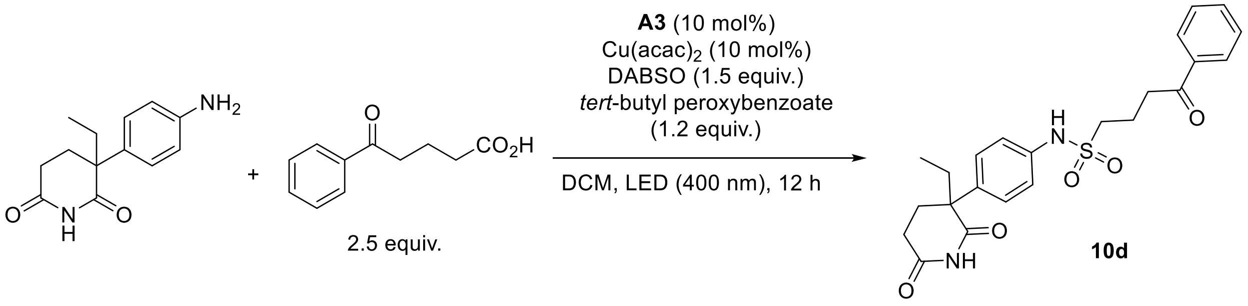

According to **GP2**, the reaction was carried out with DL-aminoglutethimide (70 mg, 0.3 mmol), 5-oxo-5-phenylpentanoic acid (144 mg, 0.75 mmol, 2.5 equiv.), DABSO (108 mg, 0.45 mmol, 1.5 equiv.), acridine **A3** (9 mg, 0.03 mmol, 10 mol%), *tert*-butyl peroxybenzoate (87 mg, 0.45 mmol, 1.5 equiv.), $Cu(acac)_2$ (8 mg, 0.03 mmol, 10 mol%) in degassed dichloromethane (3 mL). The test-tube was capped and the reaction mixture was irradiated with LED light (λ = 400 nm) while stirring for 12 h. The reaction mixture was then quenched with saturated solution of sodium hydrogen carbonate (3 mL), extracted with ethyl acetate (3 × 10 mL). The organic layer was collected, dried over anhydrous sodium sulfate, concentrated under reduced pressure, and the remaining material was purified by flash chromatography on silica gel (DCM/ MeOH 19 : 1 v/v) to give the sulfonamide **10d** (81 mg, 61%) as a white solid. ·

Evaluation Only. Created with Aspose.Pdf. Copyright 2002-2014 Aspose Pty Ltd.

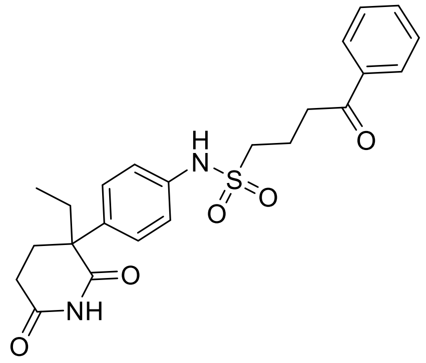

m.p.: 135–137 °C. – ^{1}H NMR (500 MHz, $CDCl_3$, rotamers): 8.94–8.61 (1 H, m), 7.94 (2 H, dt, *J* = 8.3, 1.5 Hz), 7.56 (1 H, td, *J* = 7.2, 1.4 Hz), 7.45 (2 H, td, *J* = 7.9, 1.6 Hz), 7.31–7.19 (1 H, m), 7.15–7.07 (2 H, m), 7.04–6.97 (2 H, m), 3.25–3.04 (4 H, m), 2.62–2.51 (1 H, m), 2.46–2.11 (5 H, m), 2.06–1.93 (1 H, m), 1.92–1.77 (1 H, m), 0.94–0.78 (3 H, m) ppm. – ^{13}C NMR (125 MHz, $CDCl_3$, rotamers): 198.8, 175.7, 175.5, 172.9, 172.7, 140.9, 136.6, 133.7, 133.5, 133.4, 128.8, 128.1, 127.5, 119.0, 118.7, 54.98, 54.95, 50.7, 50.6, 36.8, 33.0, 32.9, 29.41, 29.37, 27.3, 27.0, 17.8, 9.2, 9.1 ppm. – IR: 3224, 2926, 1683, 1597, 1514, 1494, 1449, 1409, 1353, 1269, 1223, 1189, 1151, 1038 cm^{-1}. – HRMS: calcd for $C_{23}H_{26}N_2O_5SNa$: 465.1455, found 465.1417 [M+Na$^+$].

Benzyl (*S*)-4-(azidosulfonyl)-2-(((benzyloxy)carbonyl)amino)butanoate (10e)

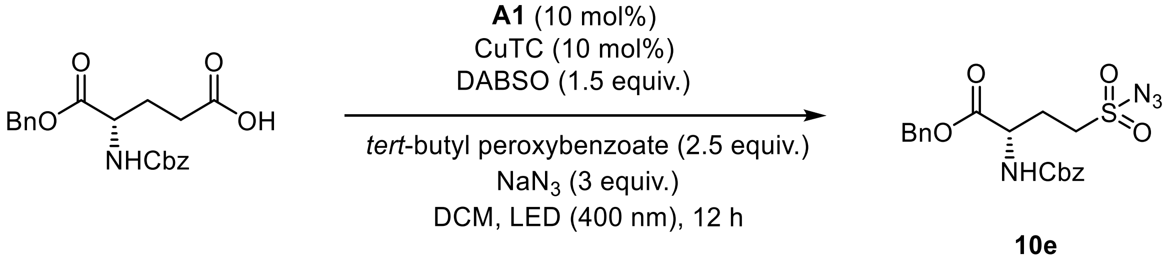

According to **GP3**, the reaction was carried out with (*S*)-5-(benzyloxy)-4-(((benzyloxy)carbonyl)amino)-5-oxopentanoic acid (111 mg, 0.3 mmol), DABSO (108 mg, 0.45 mmol, 1.5 equiv.), acridine catalyst **A1** (9 mg, 0.03 mmol, 10 mol%), sodium azide (59 mg, 0.9 mmol, 3.0 equiv.), *tert*-butyl peroxybenzoate (145 mg, 0.75 mmol, 2.5 equiv.), copper(I) thiophene-2-carboxylate (5 mg, 0.03 mmol, 10 mol%) in degassed $PhCF_3$/MeCN (3 mL, 3 : 1 v/v). The test-tube was capped, and the reaction mixture was irradiated with LED light (λ = 400 nm) while stirring at room temperature for 12 h. The reaction mixture was treated with saturated solution of sodium carbonate (3 mL) and extracted with DCM

Evaluation Only. Created with Aspose.Pdf. Copyright 2002-2014 Aspose Pty Ltd.

(3 × 10 mL). The organic layer was collected, dried over anhydrous sodium sulfate, concentrated under reduced pressure, and the remaining material was purified by flash chromatography on silica gel (hexane/ ethyl acetate 4 : 1 v/v) to give the sulfonyl azide product **10e** (74 mg, 57%) as a colorless oil.

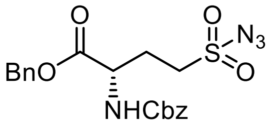

m.p.: 55–77°C. – $[\alpha]_D^{23}$ = + 6.8 (c 0.22, $CHCl_3$). –^{1}H NMR (500 MHz, $CDCl_3$): 7.42–7.30 (10 H, m), 5.55 (1 H, d, *J* = 7.7 Hz), 5.26–5.15 (2 H, m), 5.11 (2 H, s), 4.53 (1 H, q, *J* = 7.1 Hz), 3.40 (1 H, ddd, *J* = 15.9, 10.9, 5.2 Hz), 3.26 (1 H, ddd, *J* = 15.0, 11.2, 4.9 Hz), 2.51 (1 H, td, *J* = 10.7, 5.9 Hz), 2.24 (1 H, dddd, *J* = 13.6, 10.8, 8.0, 4.9 Hz) ppm. – ^{13}C NMR (125 MHz, $CDCl_3$): 170.5, 156.1, 135.9, 134.7, 129.0, 128.9, 128.8, 128.7, 128.6, 128.3, 68.2, 67.6, 52.4, 52.1, 27.0 ppm. – IR: 3032, 3011, 2964, 2140, 1764, 1708, 1421, 1360, 1220, 1092, 929, 793, 735 cm^{-1}. – HRMS: calcd for $C_{19}H_{20}N_4NaO_6S$: 455.0996, found 455.0999 [M+Na$^+$].

((3a*S*,5a*R*,8a*R*,8b*S*)-2,2,7,7-Tetramethyltetrahydro-3a*H*-bis([1,3]dioxolo)[4,5-*b*:4',5'-*d*]pyran-3a-yl)methyl 4-(azidosulfonyl)butanoate (10f)

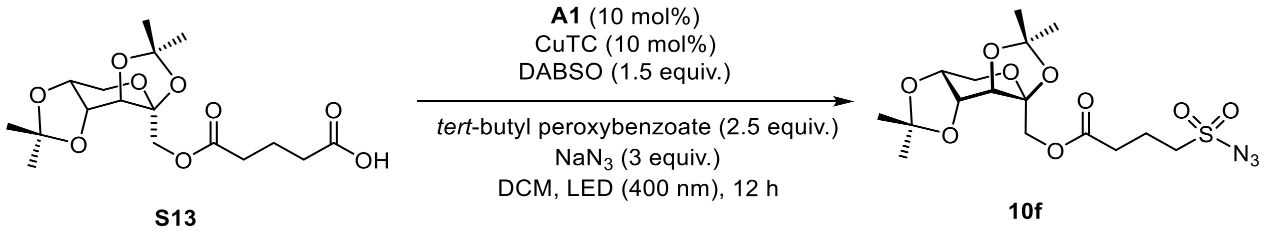

According to **GP3**, the reaction was carried out with acid **S13** (112 mg, 0.3 mmol), DABSO (108 mg, 0.45 mmol, 1.5 equiv.), acridine catalyst **A1** (9 mg, 0.03 mmol, 10 mol%), sodium azide (59 mg, 0.9 mmol, 3.0 equiv.), *tert*-butyl peroxybenzoate (145 mg, 0.75 mmol, 2.5 equiv.), copper(I) thiophene-2-carboxylate (5 mg, 0.03 mmol, 10 mol%) in degassed $PhCF_3$/MeCN (3 mL, 3 : 1 v/v). The test-tube was capped, and the reaction mixture was irradiated with LED light (λ = 400 nm) while stirring at room temperature for 12 h. The

Evaluation Only. Created with Aspose.Pdf. Copyright 2002-2014 Aspose Pty Ltd.

reaction mixture was treated with saturated solution of sodium carbonate (3 mL) and extracted with DCM (3 × 10 mL). The organic layer was collected, dried over anhydrous sodium sulfate, concentrated under reduced pressure, and the remaining material was purified by flash chromatography on silica gel (hexane/ ethyl acetate 4 : 1 v/v) to give the sulfonyl azide product **10f** (74 mg, 57%) as a colorless oil.

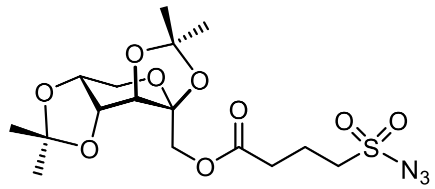

$[\alpha]_D^{23}$ = –20.0 (c 0.4, $CHCl_3$).–^1H NMR (500 MHz, $CDCl_3$): 4.59 (1 H, dq, *J* = 8.3, 2.7 Hz), 4.44 (1 H, dd, *J* = 11.6, 6.2 Hz), 4.24 (2 H, ddd, *J* = 14.2, 7.1, 4.3 Hz), 4.06 (1 H, dd, *J* = 11.6, 6.2 Hz), 3.88 (1 H, ddd, *J* = 13.0, 6.2, 2.1 Hz), 3.75 (1 H, dd, *J* = 13.1, 5.8 Hz), 3.45 (2 H, dt, *J* = 8.3, 6.0 Hz), 2.60 (2 H, q, *J* = 6.6 Hz), 2.23 (2 H, hex, *J* = 6.9 Hz), 1.53 (3 H, d, *J* = 5.6 Hz), 1.47 (3 H, d, *J* = 5.9 Hz), 1.38 (2 H, d, *J* = 5.7 Hz), 1.33 (2 H, d, *J* = 5.5 Hz) ppm. – ^{13}C NMR (125 MHz, $CDCl_3$): 171.3, 109.3, 109.0, 101.5, 70.8, 70.1, 66.0, 61.4, 54.7, 31.6, 26.6, 26.0, 25.3, 24.2, 19.0 ppm. – IR: 3428, 3014, 2334, 2145, 1743, 1657, 1364, 1267, 1214, 911, 744 cm^{-1}. – HRMS: calcd for $C_{16}H_{25}N_3NaO_9S$: 458.1204, found 458.1200 [$M+Na^+$].

(1*S*,2*S*,4a*R*,4b*R*,7*S*,9a*S*,10*S*,10a*R*)-10-(Azidosulfonyl)-1-methyl-8-methylene-13-oxo-1,2,5,6,8,9,10,10a-octahydro-4a,1-(epoxymethano)-7,9a-methanobenzo[*a*]azulene-2,7(4b*H*)-diyl diacetate (10g)

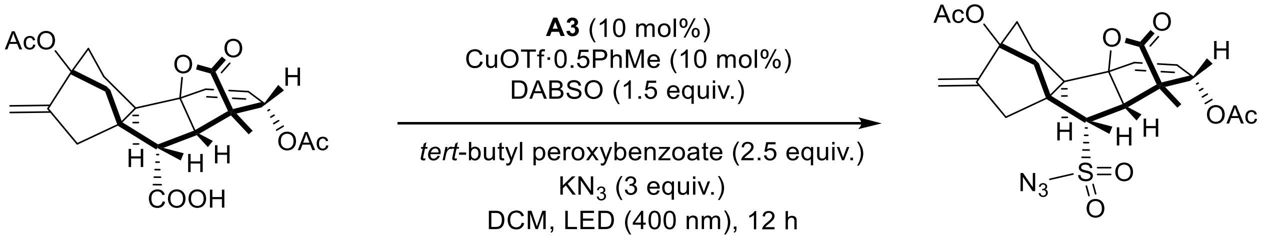

S11 **10g**

According to **GP3**, the reaction was carried out with acid **S11** (129 mg, 0.3 mmol), DABSO (108 mg, 0.45 mmol, 1.5 equiv.), acridine catalyst **A3** (9 mg, 0.03 mmol, 10 mol%), potassium azide (73 mg, 0.9 mmol, 3.0 equiv.), *tert*-butyl peroxybenzoate (145 mg, 0.75

Evaluation Only. Created with Aspose.Pdf. Copyright 2002-2014 Aspose Pty Ltd.

mmol, 2.5 equiv.), CuOTf·0.5PhMe (8 mg, 0.03 mmol, 10 mol%) in degassed $PhCF_3$/MeCN (3 mL, 3 : 1 v/v). The test-tube was capped, and the reaction mixture was irradiated with LED light (λ = 400 nm) while stirring at room temperature for 12 h. The reaction mixture was treated with saturated solution of sodium carbonate (3 mL) and extracted with DCM (3 × 10 mL). The organic layer was collected, dried over anhydrous sodium sulfate, concentrated under reduced pressure, and the remaining material was purified by flash chromatography on silica gel (hexane/ ethyl acetate 4 : 1 v/v) to give the sulfonyl azide product **10g** (102 mg, 69%) as a colorless oil.

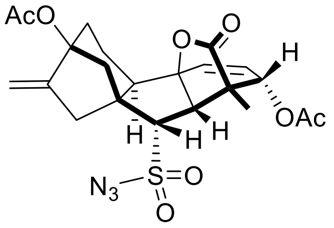

$[\alpha]_D^{23}$ = –20.0 (c 0.4, $CHCl_3$). – ^{1}H NMR (500 MHz, $CDCl_3$): 6.46 (1 H, d, *J* = 9.3 Hz), 5.89 (1 H, dd, *J* = 9.3, 3.7 Hz), 5.42 (1 H, d, *J* = 3.6 Hz), 5.12–5.05 (1 H, m), 5.04 (1 H, dd, *J* = 3.5, 1.6 Hz), 3.58 (1 H, d, *J* = 7.4 Hz), 3.42 (1 H, dt, *J* = 15.7, 3.1 Hz), 3.33 (1 H, d, *J* = 7.4 Hz), 2.66–2.53 (2 H, m), 2.33 (1 H, dd, *J* = 10.5, 3.1 Hz), 2.20–2.08 (5 H, m), 2.03–1.88 (5 H, m), 1.73 (1 H, ddt, *J* = 12.1, 7.4, 2.6 Hz), 1.39 (3 H, s) ppm. – ^{13}C NMR (125 MHz, $CDCl_3$): 175.9, 169.9, 169.5, 148.7, 133.6, 129.7, 107.2, 89.8, 83.7, 71.5, 69.8, 54.8, 52.6, 51.9, 47.4, 44.8, 41.4, 36.0, 22.1, 20.8, 17.8, 14.9 ppm. – IR: 2974, 2878, 2136, 1780, 1736, 1710, 1463, 1364, 1221, 1157, 1088, 1022, 911, 732 cm^{-1}. – HRMS: calcd for $C_{22}H_{25}N_3NaO_8S$: 514.1255, found 514.1250 [M+Na^+].

((1*S*,3*R*)-3-Acetyl-2,2-dimethylcyclobutyl)methanesulfonyl azide (10h)

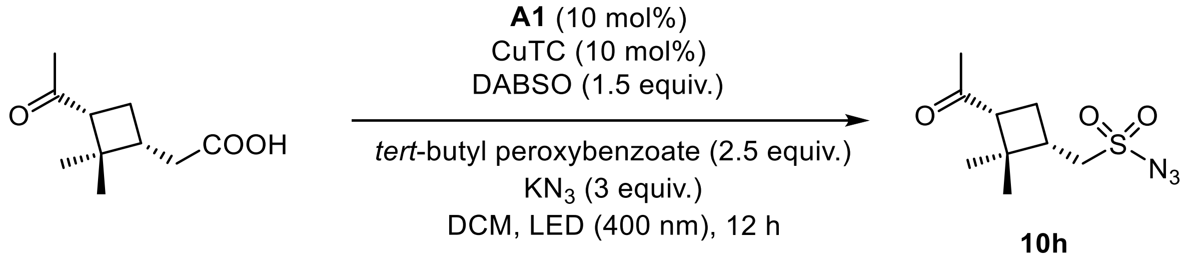

10h

According to **GP3**, the reaction was carried out with 2-((1*R*,3*R*)-3-acetyl-2,2-dimethylcyclobutyl)acetic acid (55 mg, 0.3 mmol), DABSO (108 mg, 0.45 mmol, 1.5

Evaluation Only. Created with Aspose.Pdf. Copyright 2002-2014 Aspose Pty Ltd.

equiv.), acridine catalyst **A1** (9 mg, 0.03 mmol, 10 mol%), potassium azide (73 mg, 0.9 mmol, 3.0 equiv.), *tert*-butyl peroxybenzoate (145 mg, 0.75 mmol, 2.5 equiv.), copper(I) thiophene-2-carboxylate (5 mg, 0.03 mmol, 10 mol%) in degassed $PhCF_3$/MeCN (3 mL, 3 : 1 v/v). The test-tube was capped, and the reaction mixture was irradiated with LED light (λ = 400 nm) while stirring at room temperature for 12 h. The reaction mixture was treated with saturated solution of sodium carbonate (3 mL) and extracted with DCM (3 × 10 mL). The organic layer was collected, dried over anhydrous sodium sulfate, concentrated under reduced pressure, and the remaining material was purified by flash chromatography on silica gel (hexane/ ethyl acetate 4 : 1 v/v) to give the sulfonyl azide product **10h** (53 mg, 72%) as a colorless oil.

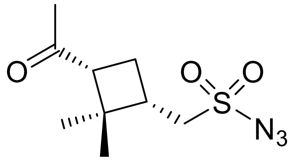

^{1}H NMR (500 MHz, $CDCl_3$): 3.37–3.17 (2 H, m), 2.96 (1 H, dd, *J* = 10.2, 7.5 Hz), 2.56 (1 H, dtd, *J* = 10.5, 8.1, 6.3 Hz), 2.20 (1 H, dt, *J* = 11.7, 10.4 Hz), 2.13–1.99 (4 H, m), 1.38 (3 H, s), 0.92 (3 H, s) ppm. – ^{13}C NMR (125 MHz, $CDCl_3$): 206.5, 56.5, 54.0, 43.7, 36.1, 30.3, 29.9, 23.1, 17.5 ppm. – IR: 3041, 2955, 2926, 2869, 2257, 2135, 1704, 1358, 1186, 1159, 735 cm^{-1}. – HRMS: calcd for $C_9H_{16}N_3O_3S$: 246.0912, found 246.0921 [M+H$^+$].

(3*R*,7*R*,8*R*,9*S*,10*S*,12*S*,13*R*,14*S*,17*R*)-17-((*R*)-4-(Azidosulfonyl)butan-2-yl)-10,13-dimethylhexadecahydro-1*H*-cyclopenta[*a*]phenanthrene-3,7,12-triyl triacetate (10i)

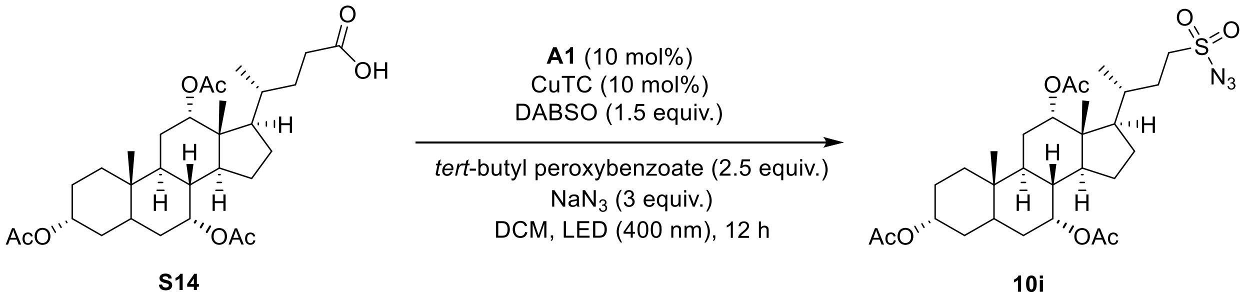

Go back to table of contents

Evaluation Only. Created with Aspose.Pdf. Copyright 2002-2014 Aspose Pty Ltd.

According to **GP3**, the reaction was carried out with acid **S14** (80 mg, 0.15 mmol), DABSO (54 mg, 0.23 mmol, 1.5 equiv.), acridine catalyst **A1** (4.5 mg, 0.015 mmol, 10 mol%), sodium azide (30 mg, 0.45 mmol, 3.0 equiv.), *tert*-butyl peroxybenzoate (73 mg, 0.37 mmol, 2.5 equiv.), copper(I) thiophene-2-carboxylate (5 mg, 0.03 mmol, 10 mol%) in degassed $PhCF_3$/MeCN (3 mL, 3 : 1 v/v). The test-tube was capped, and the reaction mixture was irradiated with LED light (λ = 400 nm) while stirring at room temperature for 12 h. The reaction mixture was treated with saturated solution of sodium carbonate (3 mL) and extracted with DCM (3 × 10 mL). The organic layer was collected, dried over anhydrous sodium sulfate, concentrated under reduced pressure, and the remaining material was purified by flash chromatography on silica gel (hexane/ ethyl acetate 4 : 1 v/v) to give the sulfonyl azide product **10i** (110 mg, 62%) as a colorless solid.

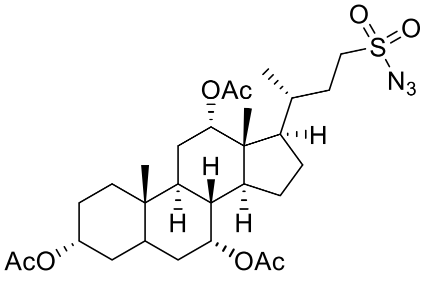

m.p.: 75–77 °C. – $[\alpha]_D^{23}$ = + 23.3 (c 0.14, $CHCl_3$). – ^{1}H NMR (500 MHz, $CDCl_3$): 5.08 (1 H, t, *J* = 2.9 Hz), 4.92 (1 H, q, *J* = 3.1 Hz), 4.58 (1 H, tt, *J* = 11.4, 4.3 Hz), 3.36 (1 H, ddd, *J* = 14.0, 11.5, 4.4 Hz), 3.21 (1 H, ddd, *J* = 14.0, 11.0, 5.0 Hz), 2.15 (3 H, s), 2.12–1.84 (10 H, m), 1.83–1.41 (13 H, m), 1.39–1.02 (5 H, m), 0.99–0.82 (6 H, m), 0.76 (3 H, s) ppm. – ^{13}C NMR (125 MHz, $CDCl_3$): 170.6, 170.5, 170.4, 75.3, 74.1, 70.7, 53.6, 47.3, 45.3, 43.5, 41.0, 37.9, 34.8, 34.7, 34.4, 34.2, 31.3, 29.2, 29.0, 27.3, 27.0, 25.7, 22.9, 22.7, 21.7, 21.59, 21.55, 17.7, 12.4 ppm. – IR: 2948, 2111, 1714, 1653, 1510, 1474, 1241, 1056, 801 cm^{-1}. – HRMS: calcd for $C_{29}H_{45}N_3NaO_8S$: 618.2820, found 618.2816 [M+H$^+$].

15-(Azidosulfonyl)pentadecane-1,7,8-triyl triacetate (10j)

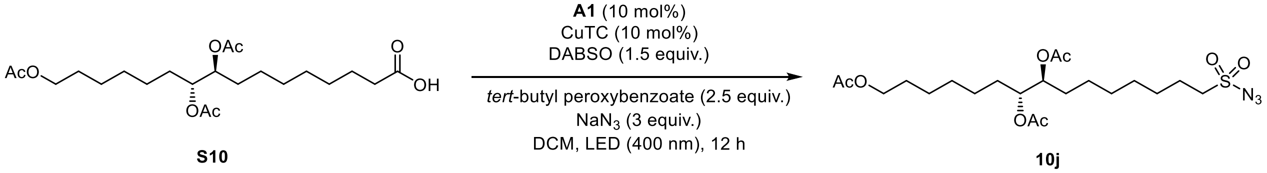

Evaluation Only. Created with Aspose.Pdf. Copyright 2002-2014 Aspose Pty Ltd.

According to **GP3**, the reaction was carried out with acid **S10** (65 mg, 0.15 mmol), DABSO (54 mg, 0.23 mmol, 1.5 equiv.), acridine catalyst **A1** (4.5 mg, 0.015 mmol, 10 mol%), sodium azide (30 mg, 0.45 mmol, 3.0 equiv.), *tert*-butyl peroxybenzoate (73 mg, 0.37 mmol, 2.5 equiv.), copper(I) thiophene-2-carboxylate (5 mg, 0.03 mmol, 10 mol%) in degassed $PhCF_3$/MeCN (3 mL, 3 : 1 v/v). The test-tube was capped, and the reaction mixture was irradiated with LED light (λ = 400 nm) while stirring at room temperature for 12 h. The reaction mixture was treated with saturated solution of sodium carbonate (3 mL) and extracted with DCM (3 × 10 mL). The organic layer was collected, dried over anhydrous sodium sulfate, concentrated under reduced pressure, and the remaining material was purified by flash chromatography on silica gel (hexane/ ethyl acetate 4 : 1 v/v) to give the sulfonyl azide product **10j** (38 mg, 52%) as a colorless oil.

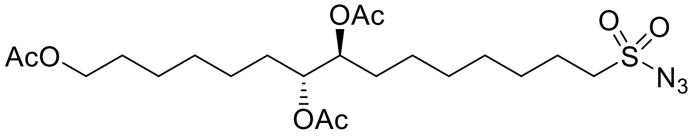

^{1}H NMR (500 MHz, $CDCl_3$): 5.01–4.93 (2 H, m), 4.02 (2 H, t, *J* = 6.7 Hz), 3.32–3.25 (2 H, m), 2.07 (6 H, s), 2.02 (3 H, s), 1.88 (2 H, p, *J* = 7.7 Hz), 1.63–1.37 (8 H, m), 1.37–1.24 (12 H, m) ppm. – ^{13}C NMR (125 MHz, $CDCl_3$): 171.3, 170.7, 73.8, 64.5, 56.0, 30.8, 30.8, 29.1, 29.0, 28.8, 28.6, 27.9, 25.8, 25.2, 25.1, 23.4, 21.1, 21.0 ppm. – IR: 3647, 2936, 2136, 1738, 1369, 1239, 1160, 1026 cm^{-1}. – HRMS: calcd for $C_{21}H_{37}N_3O_8SNa$: 514.2194, found 514.2186 [$M+Na^+$].

Evaluation Only. Created with Aspose.Pdf. Copyright 2002-2014 Aspose Pty Ltd.

Computational Data

Software

All geometry optimizations, vertical excitations, vibrational frequency calculations, and IRCs were conducted using the Gaussian 16 program.[10] Quantum chemical calculations were performed using the Stampede2 supercomputer at the Texas Advanced Computing Center (TACC) hosted by the University of Texas in Austin, Texas.[11] The CREST utility[12] of the xTB software suite[13,14] was used in conjunction with manual conformational searching to locate initial starting geometries for optimization via DFT. General day-to-day visualization and monitoring of calculations was performed with Chemcraft.[15] The quasi-harmonic approximation from Grimme[16] was applied via GoodVibes[17] to all structures to correct for potential errors associated with low magnitude vibrational frequencies using a cut-off frequency of 50 cm^{-1}. Final images of minima and transition state geometries were rendered using CYLview.[18] Spin density images were generated from the optimized .chk files (converted to .fch) with the Gaussian Cubegen utility (with spin=SCF and npts = 300). VMD[19] was used to render the final images from the generated .cube files with a green isosurface and an isovalue of 0.03 au for all radical species.

Details of Gaussian DFT calculations

Geometries of ground state minima and transition states were optimized without constraints using the D3(BJ)[20,21] dispersion-corrected PW6B95[22] DFA. The PW6B95-D3(BJ) DFA was selected on the basis of its excellent general performance in both thermochemical and NCI benchmarking studies.[23] DFT calculations were performed using the def2-TZVP[24] basis set in the SMD solvation model[25] using the "dichloromethane" keyword. Convergence criteria for these calculations was set to "tight" and an ultrafine grid was selected. Frequency calculations at the same level of

Evaluation Only. Created with Aspose.Pdf. Copyright 2002-2014 Aspose Pty Ltd.

theory were used to confirm the nature of the isolated stationary points. Geometries with zero imaginary frequencies were deemed minima whereas those with exactly one imaginary frequency along the chemical path of interest were deemed transition states. IRC calculations were performed to further corroborate that the located transition states connected reactants to products. Single point calculations were performed at the def2-TZVP-optimized stationary points at the PW6B95-D3(BJ) / def2-TZVPPD / SMD (DCM) level of theory. The electronic energy calculated at the def2-TZVPPD level replaced that calculated at the def2-TZVP level and is reported in the final calculated thermodynamic values. The def2-TZVPPD basis set was implemented in G16 by appending diffuse functions obtained from the EMSL BSE[26] to the G16-available def2-TZVPP basis set.

4. Calculation of Reduction Potential of HA1^{+}/HA1

The standard potential of the HA^{+}/HA redoc couple was calculated at the PW6B95-D3(BJ) / def2-TZVP // def2-TZVPPD / SMD (MeCN) level of theory relative to the absolute hydrogen potential in MeCN (4.43 V vs SCE).[27]

HA1^{+} (MeCN)

E(RPW6B95D3) = -1248.42706771

Charge = 1 Multiplicity = 1

C	-7.9418585362	-1.3235911305	-0.7662815109
C	-6.8099935092	-1.7880630874	-0.1683675303
C	-5.7553462106	-0.8972552283	0.0775471654
C	-5.8575341068	0.4718253461	-0.2795137085
C	-7.0505630163	0.9047916643	-0.9090507387
C	-8.0632731981	0.0299230088	-1.1438092199
C	-4.7784433498	1.323031213	-0.0125994616

Go back to table of contents

Evaluation Only. Created with Aspose.Pdf. Copyright 2002-2014 Aspose Pty Ltd.

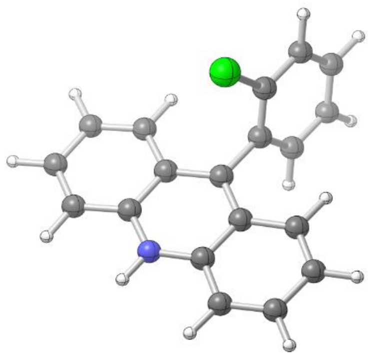

C	-3.6197697224	0.8206836395	0.5931005449
C	-3.5605915665	-0.5564502907	0.9236964259
C	-2.418825345	-1.106062717	1.5251040254
H	-2.4042928695	-2.1584736009	1.7622755298
C	-1.3582230232	-0.2955909095	1.7934125796
C	-1.3956418221	1.0797912533	1.4822607975
C	-2.4955915035	1.625080683	0.900556561
H	-8.7575197402	-2.0020558751	-0.9599371333
H	-6.7027809366	-2.8223782726	0.1184295574
H	-7.1389709476	1.9372365436	-1.2027552287
H	-8.9672031126	0.3659629733	-1.6253142252
H	-0.4758918205	-0.7097219461	2.2550015612
H	-0.5431258743	1.6976962055	1.7130935557
H	-2.5279652204	2.6770705941	0.6693273185
N	-4.6229908711	-1.3346489432	0.653918827
C	-4.8736092634	2.7591803007	-0.3493731614
C	-4.1956699339	3.3089248796	-1.4297039579
C	-5.6599118172	3.5980818926	0.4318738382
C	-4.2868114248	4.6551206542	-1.727854417
C	-5.7522500337	4.9468217254	0.1482959831
H	-6.1939055253	3.1790967295	1.2704434256
C	-5.0651408529	5.4740613336	-0.9314192232
H	-3.7546907691	5.0523802708	-2.5766735174
H	-6.361929765	5.5835130949	0.7691774238
H	-5.134807222	6.5254584304	-1.1614508001
Cl	-3.2300552513	2.2920804794	-2.4506623806
H	-4.5655083431	-2.31548848	0.8992554125

Evaluation Only. Created with Aspose.Pdf. Copyright 2002-2014 Aspose Pty Ltd.

HA1 (MeCN)

E(UPW6B95D3) = -1248.56334669

	Charge = 0	Multiplicity = 2	
C	-7.9997188128	-1.3146341236	-0.7522645474
C	-6.8396255511	-1.7706163625	-0.1566789414
C	-5.7843471678	-0.8983740306	0.077024311
C	-5.8759433836	0.4691296437	-0.2794341948
C	-7.0678700471	0.8923985634	-0.8948741759
C	-8.1092673621	0.0210935784	-1.1248920097
C	-4.7728531576	1.3257674102	-0.0242746304
C	-3.599296355	0.8085056021	0.5849731395
C	-3.5434913713	-0.564926179	0.9209921744
C	-2.4125738759	-1.1103158586	1.5155621036
H	-2.4049193278	-2.163587951	1.7550195926
C	-1.3209566969	-0.311390867	1.7948738937
C	-1.3557751666	1.0444777211	1.4848945052
C	-2.4730777592	1.5910779734	0.8943942013
H	-8.8144239108	-1.9989278466	-0.9301336574
H	-6.7349703755	-2.8060468985	0.1322848249
H	-7.1588671785	1.9243213262	-1.19380791
H	-9.0105558885	0.3777412725	-1.5988239771
H	-0.4464921653	-0.7425922249	2.2564537652
H	-0.5067756259	1.6716932915	1.7083834341
H	-2.4935831412	2.6441163194	0.6613463199
N	-4.6305121463	-1.3509195513	0.6545240111

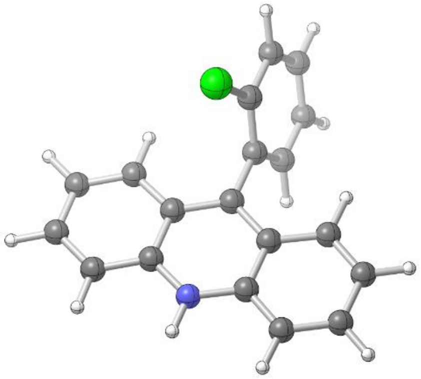

Evaluation Only. Created with Aspose.Pdf. Copyright 2002-2014 Aspose Pty Ltd.

C	-4.8631724031	2.7587431658	-0.3583541686
C	-4.1442219563	3.3382603742	-1.3997818231
C	-5.6950486175	3.5991979544	0.3790779378
C	-4.227847663	4.6876363216	-1.6917965828
C	-5.7940609602	4.9488136534	0.100944701
H	-6.266701741	3.1711928222	1.1885925893
C	-5.0557010125	5.4954506082	-0.9353527325
H	-3.6534590749	5.0954035331	-2.5077775419
H	-6.4433085073	5.5733187975	0.6943742814
H	-5.1231283748	6.5482109861	-1.1607443839
Cl	-3.1186886149	2.3485000398	-2.3944003108
H	-4.5781529374	-2.3260628851	0.8999770519

5. Calculation of the pK_{BH^+} of the lowest singlet excited state of acridine

The ground electronic states of acridinium cation, **AcrH⁺**, and acridine, **Acr**, were optimized without constraints at the PW6B95-D3(BJ) / def2-TZVP / SMD (MeCN) level of theory. TD-DFT calculations were employed to optimize the first singlet excited state of both acridinium cation, **AcrH⁺***, and acridine, **Acr***. Frequency calculations at the same level of theory confirmed the nature of the isolated stationary points.

The Förster equation was used[28] to estimate the p$K^*_{BH^+}$ of the first singlet excited state of acridine according to equation 1:

$$\mathrm{p}K^*_{BH^+} = \mathrm{p}K_{BH^+} - (hv_1 - hv_2)/2.3RT \qquad (1)$$

where pK_{BH^+} = 12.7 (ground state acidity of acridinium in MeCN)[29], $hv_1$is the computed Gibbs free energy of the 0–0 electronic transition of **AcrH⁺** to the first singlet excited state **AcrH⁺***, and hv_2 is the computed Gibbs free energy of the 0–0 electronic transition of **Acr** to the first singlet excited state **Acr***.

Evaluation Only. Created with Aspose.Pdf. Copyright 2002-2014 Aspose Pty Ltd.

AcrH+

E(RPW6B95D3) = -556.993480131

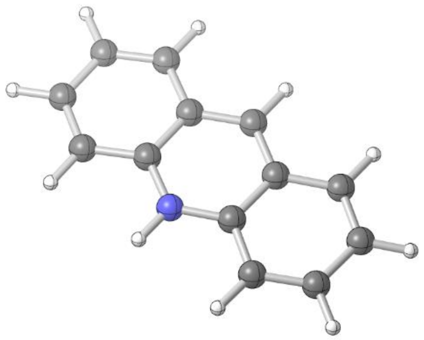

	Charge = 0	Multiplicity = 1	
C	-7.9226881823	-1.3275996813	-0.8335494745
C	-6.806156719	-1.8109775053	-0.2190830222
C	-5.7505292837	-0.930310867	0.05522086
C	-5.8415121	0.4395342933	-0.2997351241
C	-7.0199765955	0.8967313209	-0.9363138852
C	-8.035158194	0.0322705777	-1.1962096763
C	-4.770582814	1.2728924383	-0.0078786448
C	-3.6283185652	0.7906659383	0.6162402582
C	-3.5718072656	-0.5848758976	0.956435713
C	-2.4391217959	-1.1187992826	1.5869057256
H	-2.4168233042	-2.1680552	1.8367208245
C	-1.3921357345	-0.2925360453	1.8674752394
C	-1.4264044443	1.079919521	1.5379966799
C	-2.5174693432	1.6107681961	0.9271868689
H	-8.740034693	-1.997613357	-1.0489916374
H	-6.7159145506	-2.8492823677	0.0589749207
H	-0.5164221731	-0.6943954719	2.3522737789
H	-2.56476682	2.6563812957	0.66653653
N	-4.6286898169	-1.3647864237	0.6599994454
H	-8.9340954168	0.3764122577	-1.6813154923
H	-0.5781625626	1.7008393643	1.7763697562
H	-4.8270578204	2.3185214495	-0.272960859
H	-7.0847152539	1.9393851524	-1.2049726214

Evaluation Only. Created with Aspose.Pdf. Copyright 2002-2014 Aspose Pty Ltd.

H -4.5761537315 -2.3450421659 0.9087310065

AcrH+*

E(RPW6B95D3) = -556.985211426

Charge = 0 Multiplicity = 1

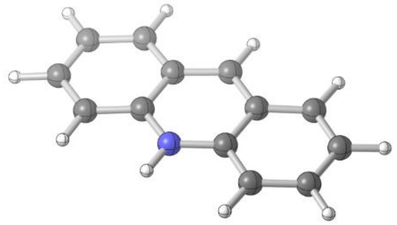

C	3.61794	-0.72835	0.07355
C	2.38516	-1.38441	0.07815
C	1.20323	-0.6497	0.0735
C	1.21971	0.76442	0.06426
C	2.46629	1.38736	0.05976
C	3.65635	0.64723	0.06433
C	-0.00001	1.46713	0.05991
C	-1.21972	0.76439	0.06424
C	-1.20321	-0.64973	0.07347
C	-2.38513	-1.38446	0.07809
H	-2.3341	-2.4628	0.08541
C	-3.61792	-0.72843	0.07347
C	-3.65635	0.64715	0.06426
C	-2.46631	1.38731	0.05972
H	4.52751	-1.30551	0.07709
H	2.33416	-2.46275	0.08548
H	-4.52747	-1.30561	0.07698
H	-2.50725	2.46567	0.05235
N	0.00002	-1.28533	0.07813
H	4.60014	1.16769	0.0604
H	-4.60016	1.16759	0.06031

Evaluation Only. Created with Aspose.Pdf. Copyright 2002-2014 Aspose Pty Ltd.

H	-0.00002	2.54517	0.0535
H	2.5072	2.46572	0.05239
H	0.00003	-2.29614	0.08319

Acr

E(RPW6B95D3) = -556.530476251

Charge = 0 Multiplicity = 1

C	-7.9118610261	-1.3330171753	-0.8267851625
C	-6.790673068	-1.79672161	-0.2159680708
C	-5.7102729722	-0.9219549039	0.070124274
C	-5.8327352086	0.453858284	-0.3001337671
C	-7.0195820138	0.8990156355	-0.9366469403
C	-8.0309332565	0.0304034908	-1.192885049
C	-4.7714721148	1.298604301	-0.0156307854
C	-3.6377741405	0.8021048365	0.6082363443
C	-3.6114129542	-0.5890823838	0.9386716161
C	-2.4558465797	-1.1094376904	1.5776899722
H	-2.444665191	-2.1601976917	1.8234461718
C	-1.4027890203	-0.3012819451	1.865694408
C	-1.4309202353	1.0765491691	1.5372866418
C	-2.5179948829	1.6128349334	0.9261766974
H	-8.7272834268	-2.0072547279	-1.0394926316
H	-6.689898809	-2.8333266498	0.0667864555
H	-0.5290720317	-0.7079289483	2.3515147964
H	-2.5531783192	2.6614483985	0.6704515384
N	-4.6254947161	-1.4124635022	0.6708272464

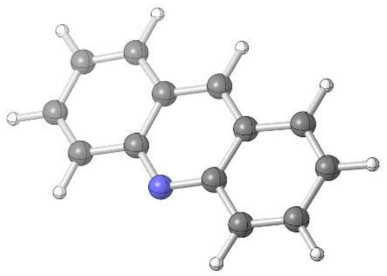

Evaluation Only. Created with Aspose.Pdf. Copyright 2002-2014 Aspose Pty Ltd.

H	-8.9325787907	0.3713678822	-1.67725628
H	-0.5803880919	1.6950488322	1.7779610834
H	-4.8279772273	2.344981082	-0.2806436647
H	-7.0966417136	1.940880093	-1.2102078142

Acr*

E(RPW6B95D3) = -556.521138939

Charge = 0 Multiplicity = 1

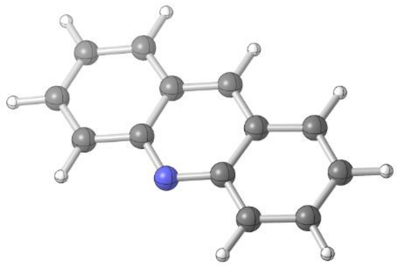

C	3.60349	-0.74524	-0.09507
C	2.36537	-1.39916	-0.11067
C	1.16226	-0.6868	-0.0936
C	1.21485	0.74245	-0.05956
C	2.46039	1.36824	-0.04445
C	3.65158	0.62913	-0.06212
C	0.00000	1.44632	-0.04297
C	-1.21484	0.74243	-0.05955
C	-1.16223	-0.68682	-0.0936
C	-2.36532	-1.39921	-0.11065
H	-2.31606	-2.47723	-0.13651
C	-3.60346	-0.74531	-0.09505
C	-3.65157	0.62906	-0.0621
C	-2.46039	1.36819	-0.04443
H	4.51258	-1.32509	-0.10895
H	2.31613	-2.47718	-0.13652
H	-4.51253	-1.32518	-0.10892
H	-2.50044	2.44733	-0.01856

Evaluation Only. Created with Aspose.Pdf. Copyright 2002-2014 Aspose Pty Ltd.

N	0.00002	-1.37043	-0.11003
H	4.5983	1.14527	-0.04972
H	-4.5983	1.14518	-0.04969
H	-0.00002	2.52633	-0.01797
H	2.50041	2.44738	-0.01858

15

E(UPW6B95D3) = -589.147663368

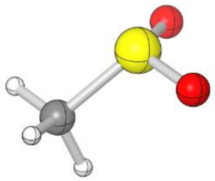

Charge = 0 Multiplicity = 2

S	0.4239244662	2.5686412502	-0.0171923999
O	1.8293933433	2.6031427341	0.34951688
O	-0.3894414089	3.7649801265	0.1185446536
C	0.3144596886	2.0231343337	-1.7204794792
H	0.8476154663	1.0834128967	-1.801226835
H	-0.7355208223	1.9135115998	-1.9644632167
H	0.7831186866	2.7995308091	-2.3195526728

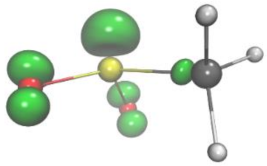

16

E(RPW6B95D3) = -2297.12450657

Charge = 0 Multiplicity = 1

Cu	-1.2785700548	1.3516227369	0.5179281706
O	-3.6261765575	0.0628104158	0.8623485524
O	-3.3106486043	0.4186232806	-1.3330957346
C	-3.7910656596	-0.2747364669	-0.2792024706
C	-4.5358615106	-1.4702791494	-0.7176945926

Go back to table of contents

Evaluation Only. Created with Aspose.Pdf. Copyright 2002-2014 Aspose Pty Ltd.

C	-4.6811748327	-1.8062689403	-2.0592256135
C	-5.1025244168	-2.270837931	0.2668786997
C	-5.391096969	-2.9389437995	-2.4080131093
H	-4.2425427513	-1.1868606732	-2.8236015963
C	-5.8104797489	-3.4016698264	-0.0878956174
H	-4.9792944753	-1.9958146969	1.3020732692
C	-5.9554196751	-3.7357614555	-1.4250852685
H	-5.5043975816	-3.200854345	-3.44822177
H	-6.2495903766	-4.0231309095	0.6765120729
H	-6.5095574532	-4.6193890116	-1.7018729966
N	-2.5325364863	1.5851132994	-0.9931568049
C	-3.4870024696	2.6669257943	-0.7264394053
H	-2.9109403811	3.5698062157	-0.5545529274
H	-4.1471057451	2.8052159803	-1.5811917691
H	-4.0578117467	2.4319198306	0.1625704624
C	-1.7828282009	1.8465144097	-2.2243230872
H	-1.1829921866	2.7334492796	-2.0534855717
H	-1.1332915927	1.0050968414	-2.4306075421
H	-2.4629883109	2.0145633034	-3.0581838417
F	0.0099425874	1.3056774376	1.8225697418

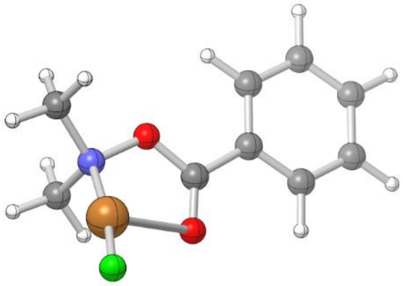

17

E(RPW6B95D3) = -2786.30600663

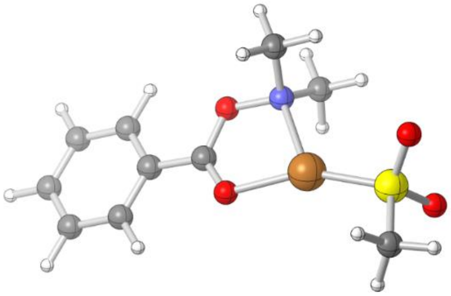

Charge = 0 Multiplicity = 1

Cu	-0.7457822168	-0.1214502144	0.1941405497
O	-2.3850536542	-0.7393910319	-0.9500723087

Evaluation Only. Created with Aspose.Pdf. Copyright 2002-2014 Aspose Pty Ltd.

O	-1.6402009823	0.637305068	-2.5607843163
C	-2.4829358784	-0.2719533159	-2.0678991818
C	-3.5368942715	-0.6459148267	-3.0179348644
C	-3.5855768265	-0.1177002	-4.3039432601
C	-4.4984909086	-1.5582997731	-2.5973616834
C	-4.5952036495	-0.5055585391	-5.162153134
H	-2.8390163108	0.5874121973	-4.6292372673
C	-5.5056775495	-1.9393082925	-3.4600696083
H	-4.4456057122	-1.9579744937	-1.5976559384
C	-5.5537391885	-1.4137198654	-4.7416881218
H	-4.6350873458	-0.100337706	-6.1607369963
H	-6.2529872953	-2.6456767928	-3.1352241914
H	-6.3412149727	-1.7135073847	-5.4156974268
N	-0.5826338673	1.0167207144	-1.6542034577
C	-0.7534889911	2.4483209756	-1.439086549
H	0.047407628	2.7700836554	-0.7815402548
H	-0.7022969691	2.9983007745	-2.3783140965
H	-1.7051565155	2.6265555916	-0.9513118161
C	0.654740827	0.7094867381	-2.3618905118
H	1.4741853359	1.0107083482	-1.7176777193
H	0.7139708193	-0.3593595236	-2.5330267784
H	0.7133450425	1.2471638712	-3.3077265149
S	0.6740770243	-0.1397661267	1.8716459316
O	1.0316415726	1.2381780365	2.2659263953
O	1.8337877777	-1.005673442	1.5811162335
C	-0.027563973	-0.8403024201	3.3616170208
H	0.7422133038	-0.7901630668	4.1293563805

Evaluation Only. Created with Aspose.Pdf. Copyright 2002-2014 Aspose Pty Ltd.

H	-0.8950346038	-0.2563209722	3.655144101
H	-0.3107632887	-1.871537943	3.1725816449

18

E(RPW6B95D3) = -2786.30980720

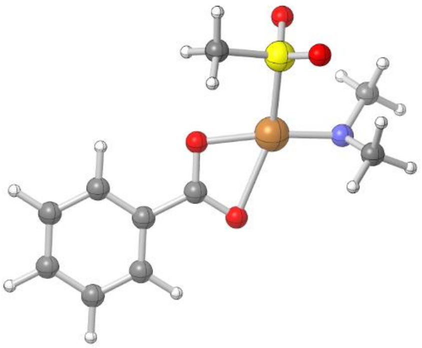

Charge = 0 Multiplicity = 1

Cu	-1.3151733783	0.6558420073	-0.3660863476
O	-2.4343036424	-0.9099575279	-0.5233703723
O	-2.3151651852	0.1870525822	-2.4158522062
C	-2.7470190269	-0.7742294199	-1.7596662043
C	-3.6263046128	-1.7985210515	-2.3709251251
C	-4.0043395797	-1.6712334641	-3.7012826707
C	-4.0696362568	-2.8845282475	-1.6268774577
C	-4.8199624719	-2.622991301	-4.2831994631
H	-3.6520091888	-0.8227636015	-4.2658118032
C	-4.8842758287	-3.8365266609	-2.2109778908
H	-3.7708415901	-2.9735921094	-0.5948960854
C	-5.2597042447	-3.7062199208	-3.53853467
H	-5.1133656378	-2.5224939557	-5.316617951
H	-5.2268604974	-4.680543632	-1.6326449792
H	-5.8958135393	-4.4499437747	-3.9936177554
N	-0.4828265812	2.2753440304	-0.4102219825
C	-0.4714207289	3.1158121968	0.7543785183
H	0.4180582614	2.9735040036	1.3744896535
H	-0.4674990353	4.1549655675	0.4128936866
H	-1.3641009421	2.9627718086	1.351128638
C	0.7807067875	2.2761514751	-1.0896052071

Evaluation Only. Created with Aspose.Pdf. Copyright 2002-2014 Aspose Pty Ltd.

H	1.5995879234	1.9599384638	-0.436216381
H	0.7466052722	1.6447786994	-1.9717827828
H	0.9979939499	3.3004866401	-1.405348047
S	-0.0427388605	0.0596785075	1.3852946101
O	-0.65962438	0.6193163536	2.5701587777
O	1.3766163948	0.2686457796	1.1867162066
C	-0.3032105058	-1.697022522	1.4152141779
H	0.2820295149	-2.0565784158	2.2584069971
H	-1.3590369719	-1.8915016432	1.5509487546
H	0.0595597027	-2.1135144783	0.4826650632

19

E(RPW6B95D3) = -2786.36719013

Charge = 0 Multiplicity = 1

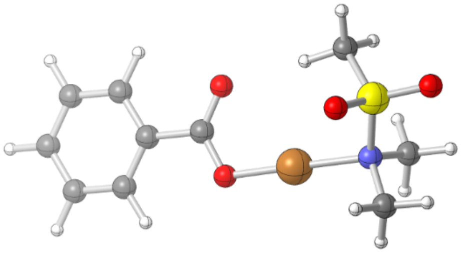

Cu	-2.3878588935	-0.7991634935	0.9716063654
O	-3.7148663016	-1.8787990245	0.1632969635
O	-4.4616335524	-0.006884972	-0.7862702496
C	-4.5222174794	-1.2197174048	-0.5808863943
C	-5.6045829639	-2.0313821312	-1.2278166575
C	-6.5110611654	-1.4123852275	-2.0783008969
C	-5.7183234873	-3.3946715348	-0.9879986201
C	-7.5167875806	-2.1447390019	-2.6821176073
H	-6.4133056743	-0.3532229596	-2.2558222313
C	-6.7251222187	-4.1283871774	-1.5896119826
H	-5.0123491855	-3.8688909899	-0.3258303342
C	-7.6256817116	-3.5046440283	-2.4380098725

Evaluation Only. Created with Aspose.Pdf. Copyright 2002-2014 Aspose Pty Ltd.

H	-8.2170563254	-1.656710995	-3.3427333031
H	-6.8087212832	-5.1871286771	-1.3969491131
H	-8.4111388971	-4.0768711213	-2.9078470093
N	-0.9365074625	0.3172672271	1.766848124
S	-1.5514798067	1.8804393248	2.1534259016
O	-0.4445427362	2.737248842	2.4625941573
O	-2.5601684965	1.6648235994	3.1453735653
C	-2.2984895215	2.3898234087	0.6526684828
H	-1.538627839	2.5662341052	-0.0994725054
H	-3.0222685766	1.6439996392	0.3292049285
H	-2.7990790801	3.3242964836	0.894158036
C	0.1787131159	0.4061504859	0.7998129086
H	0.5206404089	-0.6039714707	0.6054116016
H	-0.1663149713	0.8407489615	-0.1303703278
H	0.996094858	0.9955421502	1.209583648
C	-0.4647300802	-0.3210989298	3.0195271827
H	-1.2835172598	-0.3960516072	3.7230138827
H	-0.1163117468	-1.3145186374	2.7626253335
H	0.3554325043	0.2471144064	3.4553572434

20

E(RPW6B95D3) = -2350.50110917

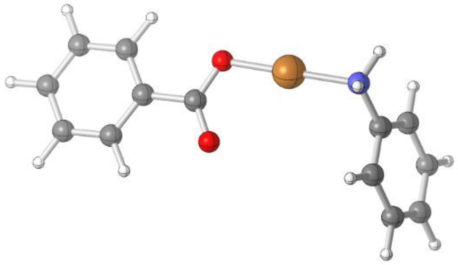

Charge = 0 Multiplicity = 1

Cu	-1.1784377153	-0.6277178183	0.6855533559
O	-3.5374861456	-1.9368862502	1.5004279524
O	-2.7603443224	-0.9153871627	-0.3207302511

Evaluation Only. Created with Aspose.Pdf. Copyright 2002-2014 Aspose Pty Ltd.

C	-3.6547256989	-1.5554720034	0.336339632
C	-4.9245527695	-1.8389797559	-0.411889571
C	-5.0940827035	-1.428086002	-1.7275720603
C	-5.9501414707	-2.5273078612	0.2226583793
C	-6.2728668267	-1.7018417571	-2.3987324148
H	-4.2939721592	-0.8949386984	-2.2150511257
C	-7.1289613321	-2.8015037254	-0.4461252366
H	-5.8048124196	-2.841507375	1.2439880324
C	-7.2921631811	-2.3887438087	-1.7591312009
H	-6.3976763636	-1.3800363593	-3.421467526
H	-7.9213014691	-3.3368487884	0.0545621174
H	-8.2116500956	-2.6024383451	-2.2827157943
N	0.463683592	-0.3401327945	1.7151231553
H	0.2581225674	0.3707086467	2.4070390453
H	1.1702665057	0.0352701953	1.0946234694
C	0.9225511567	-1.5406170482	2.3423094901
C	0.4134579476	-1.9090727586	3.5766480805
C	1.8500339298	-2.3464886095	1.7031044135
C	0.8369064685	-3.084012423	4.1702487209
H	-0.3162345552	-1.2797412911	4.0638438022
C	2.2665173805	-3.5215809378	2.3029281057
H	2.2398708599	-2.0540297518	0.7393309061
C	1.7634990096	-3.8960205368	3.5374562978
H	0.4373035364	-3.3651902388	5.1322935614
H	2.9898595978	-4.1452382361	1.8006742864
H	2.090220126	-4.8127801045	4.0023230767

Evaluation Only. Created with Aspose.Pdf. Copyright 2002-2014 Aspose Pty Ltd.

21

E(UPW6B95D3) = -2349.86283224

Charge = 0 Multiplicity = 2

Cu	-1.4756524341	0.3107474418	0.9961379614
O	-3.111388565	-0.0990954704	-0.2217896356
O	-1.4328007282	1.1174573774	-0.8967632775
C	-2.5477826705	0.5737831641	-1.1265056626
C	-3.1807574073	0.7259732967	-2.4573215831
C	-2.5664319915	1.5054231553	-3.4293814599
C	-4.3813926795	0.0877929526	-2.7401816139
C	-3.1492542411	1.6465438927	-4.6744388378
H	-1.6342766893	1.9945388429	-3.1965316124
C	-4.9614481056	0.2271909502	-3.9873159663
H	-4.8476570056	-0.5153960543	-1.9776686476
C	-4.3463369952	1.0065886476	-4.9543000314
H	-2.6712469142	2.2535013751	-5.4276929456
H	-5.8925458929	-0.2716491608	-4.2073876544
H	-4.8000807568	1.1151471272	-5.9275579186
N	-0.6811315962	0.1459813387	2.6423327044
H	-0.8453528752	0.8576263473	3.3428891168
C	0.1021157185	-0.8524702927	3.1283868145
C	0.5924006115	-0.8317125981	4.4454529151
C	0.4356641585	-1.9452733516	2.3103582643
C	1.3784089611	-1.8603645647	4.9167097584
H	0.3394586501	0.0032270006	5.0828414266
C	1.2223933215	-2.9663788818	2.791593742

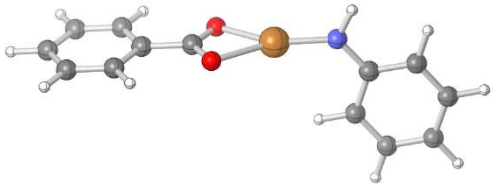

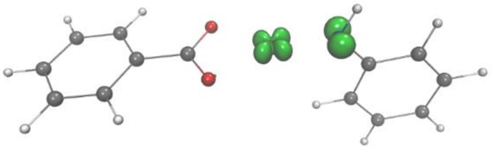

Evaluation Only. Created with Aspose.Pdf. Copyright 2002-2014 Aspose Pty Ltd.

H	0.0576138376	-1.9660738218	1.2979207013
C	1.6998474282	-2.9333969008	4.0965112871
H	1.744275949	-1.8295018507	5.9316153005
H	1.4681871572	-3.7983623892	2.1496587665
H	2.3153363452	-3.7366851735	4.4696221777

22

E(RPW6B95D3) = -2939.02963014

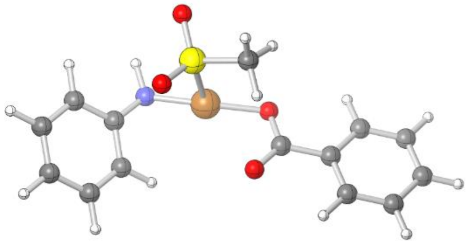

Charge = 0 Multiplicity = 1

Cu	-0.8319047715	0.1856034429	0.6235819614
O	-1.9696599969	-0.0477034092	-0.8671314613
O	-1.9215790353	2.1670703935	-0.9105549336
C	-2.2846231106	1.0908183974	-1.3807900457
C	-3.1434475138	1.0334186205	-2.599084589
C	-3.5008608997	2.2145732059	-3.2358002474
C	-3.5902211743	-0.1810905517	-3.1040033517
C	-4.2960122564	2.1825868812	-4.3660572093
H	-3.1466538452	3.1498217512	-2.8324746619
C	-4.3876911148	-0.2126707102	-4.2335204349
H	-3.3073511752	-1.0935880768	-2.6048165727
C	-4.7405537609	0.9685852195	-4.8658674199
H	-4.5699134208	3.102716678	-4.8588418514
H	-4.7335904008	-1.1581302159	-4.6221062963
H	-5.3615164995	0.9434073613	-5.7483360605
N	0.4535224346	0.1979002884	1.9244240314
S	-2.27848172	0.4975894996	2.3715778454

Evaluation Only. Created with Aspose.Pdf. Copyright 2002-2014 Aspose Pty Ltd.

O	-2.0314848715	1.7649637683	3.0222924008
O	-2.3304493548	-0.7080666165	3.1736364169
C	-3.8277122465	0.6478939641	1.5130559837
H	-3.7780553345	1.5267746952	0.881744753
H	-3.9925212885	-0.2549724248	0.9383026145
H	-4.5762196393	0.7647692404	2.2936364194
C	0.8670690962	1.3654136219	2.4905156374
C	1.1002062985	2.4818634935	1.6710502809
C	1.067085776	1.4812189274	3.874969506
C	1.5072251365	3.6726111875	2.2244197384
H	0.9505145654	2.3859603119	0.6056861479
C	1.5049146434	2.6690124445	4.4135778609
H	0.8679579935	0.6269968589	4.5050052096
C	1.7188580067	3.769913727	3.5944504868
H	1.6741164304	4.52947183	1.5909242006
H	1.6653033741	2.7493795315	5.4772771325
H	2.0507091459	4.7027710696	4.0224984945
H	0.3311381095	-0.5221661861	2.6292257135

23

E(RPW6B95D3) = -2939.07278236

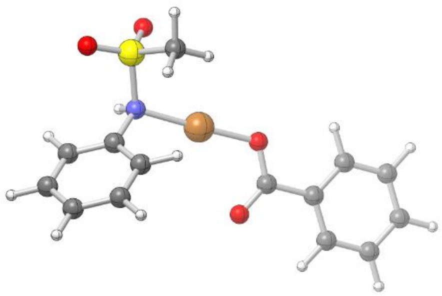

Charge = 0 Multiplicity = 1

Cu	-1.5119479772	1.0742226949	-0.1747295544
O	-2.3042457263	-0.607483139	-0.5215221609
O	-3.2971772399	0.3275590383	-2.2832856418
C	-3.0802780374	-0.6288326385	-1.5414520561

Evaluation Only. Created with Aspose.Pdf. Copyright 2002-2014 Aspose Pty Ltd.

C	-3.752031188	-1.945262277	-1.8012352066
C	-4.639700961	-2.0645353055	-2.8621831413
C	-3.5041377024	-3.0484490414	-0.9945248009
C	-5.2730551875	-3.2681034617	-3.1132130119
H	-4.8235050488	-1.2009914602	-3.481468943
C	-4.1352401869	-4.2538374751	-1.2454231599
H	-2.8138946384	-2.949243976	-0.1726064679
C	-5.021222392	-4.3653839738	-2.3047828047
H	-5.9629391222	-3.35248588	-3.9390161056
H	-3.9368118257	-5.1071700786	-0.614911674
H	-5.5147280377	-5.3053418847	-2.5001101045
N	-0.6073012182	2.8143754882	0.2256980791
S	-0.0112340855	2.9124888708	1.8455839564
O	0.3369256863	4.269745078	2.1274893435
O	0.9904240232	1.8938226092	1.911119479
C	-1.4128133174	2.4599834992	2.7897712992
H	-1.0906179757	2.4968092653	3.8272052848
H	-2.2056219595	3.1806709082	2.6236296159
H	-1.716452922	1.4547938799	2.5177552334
H	0.2448379076	2.7495515578	-0.3325282689
C	-1.389108815	3.9518227667	-0.2112151924
C	-2.7632252485	3.9171355113	-0.0620549645
C	-0.7602289333	5.0479246806	-0.7737100624
C	-3.5185384012	4.9994228546	-0.4757706584
H	-3.2303949175	3.0372392859	0.3568980155
C	-1.5233350012	6.1201705147	-1.1980268898
H	0.3135439293	5.0562822201	-0.8813181523

Evaluation Only. Created with Aspose.Pdf. Copyright 2002-2014 Aspose Pty Ltd.

C	-2.899873451	6.0997738313	-1.0445405007
H	-4.5910306958	4.9746393566	-0.3655674278
H	-1.0399053857	6.9738465498	-1.6461788398
H	-3.4911002904	6.9391149312	-1.3755519685

24

E(RPW6B95D3) = -2710.34829951

Charge = 0 Multiplicity = 1

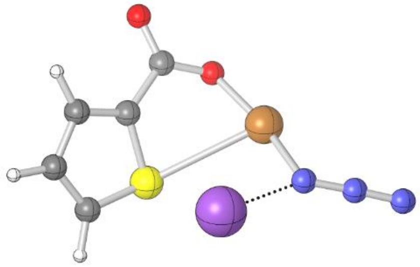

Cu	-0.2907046406	-0.7214379863	-0.5046487652
O	-1.777361211	0.4297139874	-0.6256680555
C	-2.4694628015	0.6105106225	-1.6897387177
O	-3.6839048997	0.7398726285	-1.7244187007
S	-0.0199594024	0.9604386048	-3.0574747465
C	-1.7235280913	0.6994452795	-2.9842095048
C	-2.2488471224	0.6520433427	-4.2388599982
C	-1.2822514278	0.7944409599	-5.2611833612
H	-1.5091391287	0.7777962725	-6.3143364218
C	-0.0268916418	0.9694447509	-4.7703104714
H	0.8864775131	1.1324794011	-5.3151216874
H	-3.3041413858	0.5191991182	-4.4089858289
N	2.3946014281	-3.1039828133	0.8015488022
N	1.7669884749	-2.525461167	0.0493718726
N	1.1353331177	-1.9370904329	-0.775450007
Na	0.9656582193	-1.8140911487	-3.0521659185

Evaluation Only. Created with Aspose.Pdf. Copyright 2002-2014 Aspose Pty Ltd.

25

E(UPW6B95D3) = -2547.77031162

Charge = 0 Multiplicity = 2

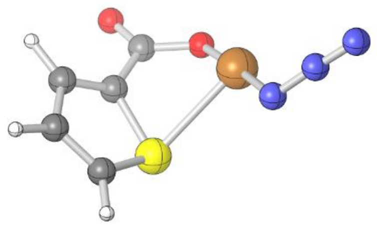

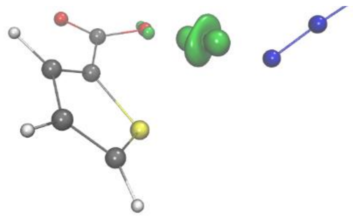

Cu	-0.3632655039	-0.2693976265	-0.9349602137
O	-1.9975253366	0.5556254884	-0.5130425785
C	-2.7478122045	0.872464448	-1.5271264859
O	-3.9409383948	1.0674437873	-1.4721182506
S	-0.3692334655	1.5666274383	-2.7917175872
C	-1.996081437	0.9590734597	-2.8073326531
C	-2.3257889617	0.5028601223	-4.0415816601
C	-1.2603126562	0.6151844147	-4.9681873756
H	-1.3296505262	0.3134938467	-6.0001244831
C	-0.1326442044	1.1464775111	-4.4270492518
H	0.8008942626	1.3659781721	-4.913337843
H	-3.2875655131	0.0735815407	-4.2676455661
N	1.2955905439	-1.0292141746	-1.3994981799
N	1.7223397024	-1.9820395347	-0.8116681582
N	2.188514555	-2.8834410735	-0.2993593632

26

E(RPW6B95D3) = -3136.93280048

Charge = 0 Multiplicity = 1

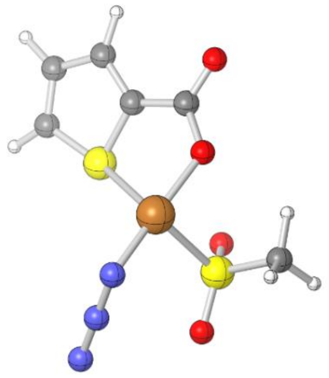

Cu	0.0237390754	-0.582227709	-0.9737585513
O	-1.7364722847	-0.0159493288	-0.8452991622
C	-2.559671176	0.2245658883	-1.8296827835

Evaluation Only. Created with Aspose.Pdf. Copyright 2002-2014 Aspose Pty Ltd.

O	-3.7634600504	0.1508775519	-1.6889741995
S	-0.3594729816	1.1137188131	-3.3352368683
C	-1.9866337201	0.6032804465	-3.1370258977
C	-2.6419035896	0.6105218098	-4.3341826536
C	-1.8211437564	1.0092694884	-5.4082307615
H	-2.1542696969	1.0834479796	-6.4298930274
C	-0.5507309032	1.3030264809	-5.0143302982
H	0.2716070598	1.6496764029	-5.614609605
H	-3.6775192158	0.3289515136	-4.4240086984
N	1.6695356402	-1.3809773553	-0.9655806331
N	2.7409222394	-0.8448501584	-1.001399253
N	3.7810307398	-0.3991120464	-1.0257134844
S	0.585916265	1.1227065085	0.4656340088
O	0.0057195059	2.2901822972	-0.123047402
O	1.9768709476	1.1027352977	0.8066059613
C	-0.376295145	0.5258068815	1.8264911776
H	-0.2414822726	1.2648379924	2.6148346093
H	-1.40528944	0.4620834417	1.4953742757
H	0.0333504895	-0.4369882961	2.1119898363

27

E(RPW6B95D3) = -3136.97570826

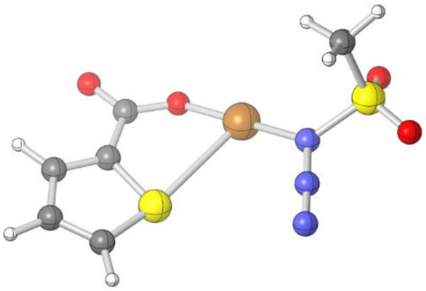

Charge = 0 Multiplicity = 1

Cu	-1.1507227266	-0.6265385763	-0.4442329463
O	-2.7364325236	0.1660609278	-1.0424234432
C	-3.1723352056	0.4351537684	-2.2195817688

Evaluation Only. Created with Aspose.Pdf. Copyright 2002-2014 Aspose Pty Ltd.

O -4.3544919587 0.585318085 -2.4940170691

S -0.4770842364 0.6116964722 -3.0394137313

C -2.1723376478 0.592015474 -3.3128211764

C -2.4410818117 0.7599922951 -4.6395007006

C -1.2810650514 0.8899565686 -5.4323672494

H -1.2897409852 1.0274018897 -6.5010679065

C -0.1380319248 0.8269418214 -4.6953019681

H 0.8796769734 0.9040837728 -5.0350415713

H -3.4495697874 0.7864989015 -5.0165139326

N 0.5085771604 -1.4991070772 0.1067144128

N 1.5472196795 -1.3482209215 -0.5604725212

N 2.4569323571 -1.1757883787 -1.1719142544

S 0.6763657818 -2.6465927103 1.4074629529

O -0.1214097383 -2.100067046 2.4489150442

O 2.0762287355 -2.8613453896 1.5708875981

C -0.0982602331 -4.0553820465 0.7236152822

H -0.0779023506 -4.8174552357 1.4993206716

H -1.1205264175 -3.7960909318 0.4705143731

H 0.462756881 -4.377986043 -0.146885096

S3

E(RPW6B95D3) = -2786.30321878

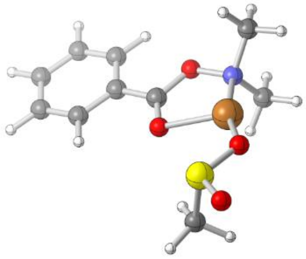

Charge = 0 Multiplicity = 1

Cu 0.7929297244 0.3745244982 0.5369577767

O -1.4766371484 -0.3090145502 0.3875328634

O -1.3902218525 1.2650438512 -1.2166944365

Go back to table of contents

Evaluation Only. Created with Aspose.Pdf. Copyright 2002-2014 Aspose Pty Ltd.

C	-2.0356414421	0.4101623007	-0.4016048131
C	-3.494280973	0.4655862255	-0.5968874647
C	-4.0860704998	1.2869984455	-1.5502931073
C	-4.2809063997	-0.3480516807	0.2104280239
C	-5.460308727	1.2900182568	-1.690475984
H	-3.4765407256	1.9157962628	-2.1775560741
C	-5.6534082519	-0.3395475684	0.0656827405
H	-3.8051351697	-0.9790086043	0.9438013615
C	-6.2430247779	0.4788876975	-0.8847878586
H	-5.9220425764	1.9253919827	-2.4296385622
H	-6.2641908204	-0.9703575197	0.6919671022
H	-7.3160365562	0.4840155881	-0.9985873925
N	0.0490757266	1.2537594765	-1.1176933902
C	0.4129696968	2.6706760014	-1.1651693684
H	1.4958689352	2.7264370119	-1.1498711839
H	0.0315900762	3.1353083667	-2.0733892708
H	0.0147080863	3.1702082085	-0.2904580983
C	0.5099832265	0.5471893026	-2.3158438007
H	1.5944254158	0.5730579377	-2.3113872607
H	0.1765621327	-0.4830757629	-2.2750276308
H	0.1312455338	1.0329130167	-3.2138368984
S	1.3379350218	-1.0450939737	3.1150345352
O	2.475032967	-1.5020872533	3.9484201941
O	1.9133710484	-0.227980407	1.9290143214
C	0.8419255586	-2.5424108136	2.2397792815
H	1.7226849803	-2.9168355878	1.7225372065
H	0.4901950575	-3.2696216223	2.9669038031

Evaluation Only. Created with Aspose.Pdf. Copyright 2002-2014 Aspose Pty Ltd.

H	0.0486747326	-2.3076870873	1.5346093854

S4

E(RPW6B95D3) = -2786.28907255

Charge = 0 Multiplicity = 1

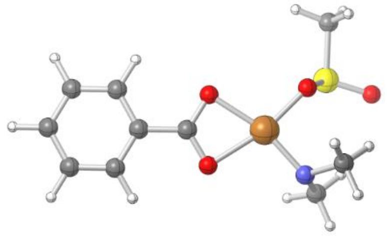

Cu	-0.7217651504	0.414257556	-0.6415475556
O	-1.7008372754	-1.3449292458	-0.6773624432
O	-2.2139878736	0.2861939466	-2.0076071402
C	-2.4269601381	-0.8969808643	-1.6109706389
C	-3.482054442	-1.7259364372	-2.2148148306
C	-4.2598902463	-1.2205655066	-3.2489628997
C	-3.696902862	-3.0157894231	-1.7452602641
C	-5.2489687593	-2.004886322	-3.810658683
H	-4.0812739234	-0.2177384921	-3.6027811038
C	-4.6873337296	-3.7965946522	-2.3087120825
H	-3.0846111849	-3.3932131628	-0.9420229106
C	-5.4624914169	-3.2913734516	-3.3407950077
H	-5.8541067402	-1.6155563016	-4.6143449526
H	-4.8560297159	-4.7982571987	-1.9454480643
H	-6.2356716631	-3.9024137212	-3.78071362
N	-0.070573273	2.0995837837	-1.0539457634
C	-0.782649675	3.0831693556	-0.2861512587
H	-0.4035726889	3.1945874703	0.7323576584
H	-0.640707381	4.046106833	-0.7889033498
H	-1.8480379651	2.872060211	-0.2792680708
C	1.3512921094	2.2583269379	-0.9503514593

Evaluation Only. Created with Aspose.Pdf. Copyright 2002-2014 Aspose Pty Ltd.

H	1.6998557378	2.4296765486	0.0683891108
H	1.8649203135	1.40846318	-1.3868813262
H	1.6024939289	3.1415373791	-1.5484917879
S	0.0937029994	0.6458162826	2.1651735471
O	0.9562336537	1.7799831373	2.5481007718
O	0.4896063899	0.1550808395	0.7224837349
C	0.7779280037	-0.764178233	3.0295660742
H	1.825615088	-0.84183576	2.7517609978
H	0.6754144426	-0.5750320875	4.0941263068
H	0.2301494373	-1.6573246012	2.7463790109

S5

E(RPW6B95D3) = -2618.06018254

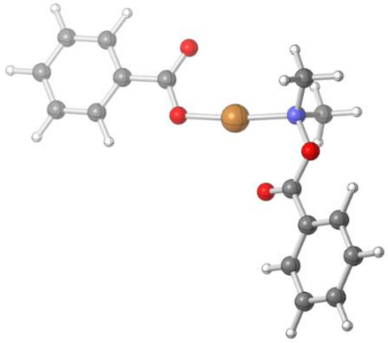

Charge = 0 Multiplicity = 1

Cu	-1.4017348177	1.3653953674	0.6365944416
O	0.7375354472	3.1598544183	1.3873806644
O	-0.2302657362	1.2915347543	2.1259893539
C	0.6302660116	2.2363651879	2.1936064768
C	1.5625961377	2.1696367783	3.3696724927
C	1.4565485239	1.1600995432	4.317534662
C	2.550399128	3.1348784056	3.5136594683
C	2.3255466087	1.1174070796	5.3936726431
H	0.6877290901	0.4136116419	4.200571257
C	3.4208074532	3.0932586256	4.5874571945
H	2.6223726444	3.9134971664	2.7710267486
C	3.3092209077	2.0836486333	5.5303543176

Evaluation Only. Created with Aspose.Pdf. Copyright 2002-2014 Aspose Pty Ltd.

H	2.2361769439	0.3302218675	6.1268565095
H	4.1864176317	3.8470937381	4.691074053
H	3.9872606982	2.0499269503	6.3696415732
O	-3.4931729408	-0.0062100408	0.8384686883
O	-3.3036304536	0.4263036503	-1.3560616424
C	-3.728343042	-0.3026763026	-0.3045215906
C	-4.5045874899	-1.4790822508	-0.7338416691
C	-4.7558511055	-1.7555454207	-2.0733402247
C	-4.9932367304	-2.322683893	0.2569053876
C	-5.4927816087	-2.8732606805	-2.4139024633
H	-4.3793389987	-1.1012798563	-2.8418710808
C	-5.7284641756	-3.4381615373	-0.0900462253
H	-4.7893004754	-2.0923984982	1.2902080448
C	-5.9785754256	-3.7135231135	-1.4251110462
H	-5.6889831398	-3.0895314483	-3.4522049989
H	-6.1072839445	-4.093062589	0.6786554503
H	-6.5541603594	-4.5852488154	-1.6957320806
N	-2.5336034506	1.5931953682	-1.0026807988
C	-3.5002790263	2.6757665399	-0.797476353
H	-2.9363954959	3.578277627	-0.5869776245
H	-4.107392657	2.8170246116	-1.6902584196
H	-4.1263482867	2.43903532	0.0542566392
C	-1.7139958745	1.8334236635	-2.1898235839
H	-1.1232603843	2.7221867626	-1.9971584563
H	-1.0536065094	0.9889897735	-2.3433364195
H	-2.340963095	1.988622972	-3.0667683889

Evaluation Only. Created with Aspose.Pdf. Copyright 2002-2014 Aspose Pty Ltd.

S6

E(RPW6B95D3) = -2939.01193791

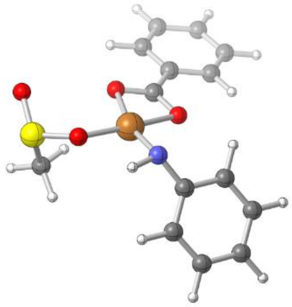

Charge = 0		Multiplicity = 1	
Cu	0.3656969222	-0.7207227415	0.0381641203
O	-1.5238213806	-1.3075222839	0.2979308973
O	-0.9491385416	-0.0089207499	-1.3379521449
C	-1.850406907	-0.6654500375	-0.7481600562
C	-3.229797976	-0.6982892791	-1.2557161107
C	-3.5635386907	0.026039692	-2.3933849589
C	-4.191239681	-1.4618709581	-0.6056272281
C	-4.8564780632	-0.0146689694	-2.8781125872
H	-2.8046797013	0.6113462006	-2.887633912
C	-5.4828026369	-1.5006758553	-1.0942342937
H	-3.9159813126	-2.0206513336	0.274536863
C	-5.8147006409	-0.7779421471	-2.2295364308
H	-5.1183522524	0.5457432648	-3.7618737369
H	-6.2311339195	-2.0948066268	-0.5935384969
H	-6.8236911833	-0.8107954054	-2.6109996466
N	1.2713883162	-1.7709375197	1.3215145514
H	2.1839598827	-1.3227678065	1.3430136749
C	0.7245310203	-1.745791645	2.5692986039
C	1.3298900391	-1.0471170106	3.6325202796
C	-0.4436248348	-2.4909535599	2.8211998712
C	0.7950709642	-1.1077643966	4.8964788759
H	2.2221888999	-0.4716091817	3.4341620109
C	-0.9764173715	-2.5318115572	4.0876448124

Evaluation Only. Created with Aspose.Pdf. Copyright 2002-2014 Aspose Pty Ltd.

H	-0.8933517918	-3.0336578159	2.0071456212
C	-0.3624357343	-1.8434988648	5.1269481712
H	1.2691549705	-0.5806773689	5.7093760783
H	-1.867394441	-3.1092146047	4.2778554662
H	-0.7821815687	-1.8847077884	6.1199037575
S	2.3007478905	1.3875611608	-1.0529636225
O	2.0669750202	0.0414324881	-0.3152984542
O	1.8343855209	1.3566857022	-2.4461958026
C	1.1289646988	2.4441418228	-0.1954168313
H	1.3539561596	2.4328301057	0.8666736292
H	1.2348959277	3.4491236128	-0.5926076829
H	0.1303503964	2.0648754576	-0.3960892881

S7

E(RPW6B95D3) = -3136.91069658

Charge = 0 Multiplicity = 1

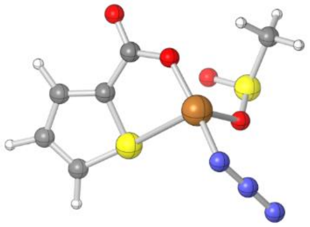

Cu	0.3035023118	-0.5020030109	-0.6710483788
O	-1.3163639755	0.3758250344	-0.4882924585
C	-2.1171007224	0.884393129	-1.3810221972
O	-3.2560983872	1.2014301375	-1.1000776804
S	0.027985631	1.0260743023	-3.2149273905
C	-1.6313656147	1.0391771221	-2.768247868
C	-2.4240089775	1.1677410631	-3.8724012306
C	-1.6904559671	1.2340371975	-5.0737062103
H	-2.1337623686	1.3377932678	-6.0499179335
C	-0.3459727277	1.1581954756	-4.8671949463

Evaluation Only. Created with Aspose.Pdf. Copyright 2002-2014 Aspose Pty Ltd.

H	0.4440913988	1.2031165148	-5.5957449289
H	-3.4984580862	1.1986629914	-3.8062451456
N	1.7528087061	-1.6237341826	-0.8882681479
N	2.8802640519	-1.2377547385	-0.79252319
N	3.9650229703	-0.9132016149	-0.7117934036
S	0.9664014261	2.295160257	0.3852919703
O	1.4636886377	0.9809365649	-0.1563354804
O	0.0753325364	3.0574840953	-0.4653448204
C	0.1221576775	1.9042116741	1.9070346342
H	-0.7508240367	1.3194637385	1.6283455307
H	0.8137464175	1.3489646305	2.5305590676
H	-0.1588330514	2.8458943911	2.3657041883

HA1^{+}

E(RPW6B95D3) = -1248.42422538

Charge = 1 Multiplicity = 1

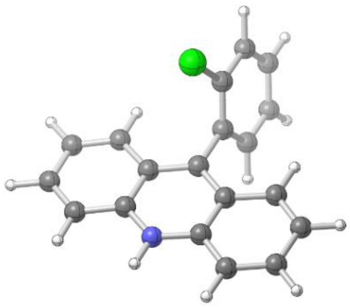

C	-7.9428478785	-1.3211033737	-0.7664793289
C	-6.8123268605	-1.7857480325	-0.1656122015
C	-5.7569147963	-0.8967149859	0.0802412081
C	-5.8568902398	0.4718776889	-0.2791555469
C	-7.0480559417	0.9048951974	-0.9120852234
C	-8.061734952	0.0312686265	-1.1472275797
C	-4.7766763885	1.3226596278	-0.0133005536
C	-3.6175471237	0.8197428729	0.5920453778
C	-3.559914826	-0.5566948543	0.9261007578
C	-2.4189315581	-1.1060541341	1.5283064333

Evaluation Only. Created with Aspose.Pdf. Copyright 2002-2014 Aspose Pty Ltd.

H	-2.4040443391	-2.1579380588	1.7684313871
C	-1.3566387381	-0.2963335343	1.7933395708
C	-1.3918775864	1.0777357845	1.4778722298
C	-2.4914876318	1.6229075088	0.8954945922
H	-8.7585257608	-1.9993760858	-0.9605924719
H	-6.7091528684	-2.8202166213	0.122216982
H	-7.133112578	1.9365391568	-1.2092969096
H	-8.9636078257	0.3673585766	-1.6323483189
H	-0.4745536513	-0.7104986703	2.2552624746
H	-0.5377960978	1.6946870197	1.7050545965
H	-2.5219028732	2.6736848267	0.6589614294
N	-4.6247469379	-1.3340787172	0.6590606827
C	-4.8731719438	2.7591403828	-0.348822053
C	-4.1940513766	3.3104827759	-1.4278598309
C	-5.662632841	3.5960680411	0.4317123999
C	-4.2880148767	4.6568584219	-1.7247769566
C	-5.7569274914	4.9447355685	0.149159584
H	-6.1977330276	3.1761885512	1.2691737756
C	-5.069050128	5.4737210101	-0.9290755659
H	-3.7559580098	5.0552223968	-2.5730359397
H	-6.3687183649	5.5798120801	0.7695015773
H	-5.1405351632	6.5250831619	-1.1584463192
Cl	-3.2263711487	2.2959389565	-2.4477027334
H	-4.5682346787	-2.3138187308	0.9058867919

HA1

E(UPW6B95D3) = -1248.56334669

Evaluation Only. Created with Aspose.Pdf. Copyright 2002-2014 Aspose Pty Ltd.

Charge = 0 Multiplicity = 2

C	-8.0003303956	-1.3126863304	-0.7524693466
C	-6.8409264465	-1.7687007243	-0.1557495129
C	-5.7852085222	-0.8977571906	0.0780534727
C	-5.8753618892	0.4694145728	-0.2792650641
C	-7.0662372146	0.8926393157	-0.8962628123
C	-8.1081819992	0.0223791164	-1.1263006767
C	-4.7719054923	1.325474141	-0.0235768285
C	-3.598312903	0.8079710015	0.5855550829
C	-3.5428686034	-0.5650621928	0.9223017121
C	-2.4121950064	-1.110347076	1.5163682665
H	-2.4035419293	-2.1635717601	1.7565005154
C	-1.319969259	-0.3120197262	1.7946300702
C	-1.3545395503	1.0432751243	1.484020674
C	-2.4717142535	1.589794174	0.8939109657
H	-8.8152066829	-1.9966274479	-0.9305481342
H	-6.7386188739	-2.8044046008	0.1334091304
H	-7.1551049304	1.9240338205	-1.1972252108
H	-9.0084096494	0.3793437883	-1.6018586221
H	-0.4453315161	-0.7434262594	2.255552415
H	-0.5050291627	1.6702104137	1.7059796196
H	-2.4921458407	2.6424215313	0.6596159978
N	-4.6315416654	-1.3502384444	0.6570910814
C	-4.8623803511	2.7586027008	-0.3576354433
C	-4.1436022045	3.3383107956	-1.3993589983
C	-5.6950402468	3.5983817414	0.379531088

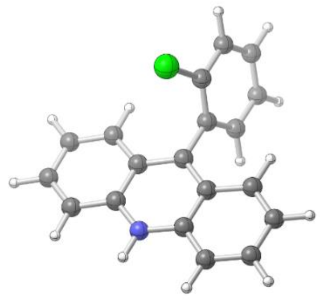

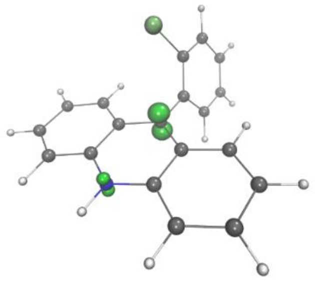

Evaluation Only. Created with Aspose.Pdf. Copyright 2002-2014 Aspose Pty Ltd.

C	-4.2298959285	4.6876484336	-1.6912885094
C	-5.7961525546	4.9476527424	0.1010244382
H	-6.2656970138	3.1695582916	1.1892462403
C	-5.058850317	5.4947027455	-0.9355729354
H	-3.6559511016	5.0951816718	-2.5076437451
H	-6.4460061434	5.5714315221	0.6945323372
H	-5.127931306	6.5472376842	-1.1615118399
Cl	-3.1161108839	2.3507679686	-2.3921851666
H	-4.5790884925	-2.3249353645	0.9014309888

NaN_3

E(RPW6B95D3) = -326.982037351

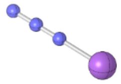

Charge = 0 Multiplicity = 1

N	-0.3001636274	0.3370935541	1.4317159644
N	0.846065844	0.5421730688	1.5871540705
N	1.9726925196	0.7434783161	1.7398998803
Na	-2.4087338562	-0.013259469	1.1617228948

NaOBz

E(RPW6B95D3) = -583.476036956

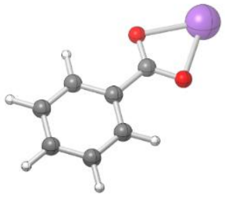

Charge = 0 Multiplicity = 1

C	-1.1860576412	3.1930307325	0.7011325236
C	-0.5368649894	1.8452866382	0.5606563864
C	-1.3128798949	0.7096293182	0.371914094
C	0.8448972913	1.72272864	0.6183380997

Evaluation Only. Created with Aspose.Pdf. Copyright 2002-2014 Aspose Pty Ltd.

C	-0.7167459261	-0.5320586068	0.2426758468
H	-2.3849731249	0.817050946	0.3288134043
C	1.4435792999	0.4822472603	0.4891753895
H	1.4373029072	2.6117015156	0.7651593104
C	0.6630945137	-0.6474236594	0.3010879349
H	-1.3262079309	-1.4108745758	0.0962635685
H	2.5184882368	0.3944755199	0.5347886534
H	1.1294133928	-1.6157325843	0.2000904757
O	-0.4423532842	4.1912903119	0.8744981757
O	-2.4398482824	3.2538458393	0.6382732776
Na	-2.2822295678	5.4701937045	0.9381628594

$MeSO_2N_3$

E(RPW6B95D3) = -753.632418892

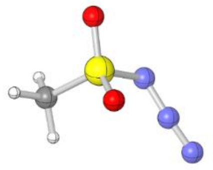

Charge = 0 Multiplicity = 1

S	0.5939091706	2.3341077126	0.1161365338
O	2.0004024962	2.5206330245	0.241802793
O	-0.320227738	3.2954208173	0.6511020931
C	0.1861212227	1.993563626	-1.5526232353
H	0.8134348415	1.181942276	-1.9036764076
H	-0.8660102941	1.7355782454	-1.6118070468
H	0.3864073155	2.9036793309	-2.1120392011
N	0.3072411023	0.8375801277	0.8695227408
N	-0.8332519374	0.7052377379	1.3176538014
N	-1.8350601794	0.5051541018	1.7610329287

Evaluation Only. Created with Aspose.Pdf. Copyright 2002-2014 Aspose Pty Ltd.

$MeSO_2NMe_2$

E(RPW6B95D3) = -723.985046360

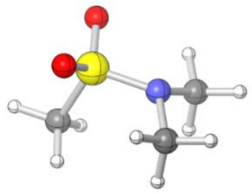

Charge = 0 Multiplicity = 1

S	0.4130438969	2.5512468419	-0.0153193732
O	1.7965155521	2.6040055903	0.3606127867
O	-0.3992443349	3.7256196171	0.1233272531
C	0.3298195112	2.0338710543	-1.6973915083
H	0.8761334525	1.1030165897	-1.8108061228
H	-0.7106744509	1.90934049	-1.979733668
H	0.7885896147	2.8106373533	-2.3013593706
N	-0.2842223688	1.3745477338	0.8750156159
C	-1.7257039749	1.2214069686	0.7505229856
H	-1.9993805307	0.6339150481	-0.1284398698
H	-2.0882900146	0.706408728	1.635503848
H	-2.1973209936	2.1944940796	0.6981901572
C	0.4407562603	0.1182680952	0.9892161733
H	0.0780797183	-0.4014595399	1.8713623034
H	0.2843201947	-0.5238743984	0.119828511
H	1.4986943377	0.3126166985	1.1117969187

MeSO₂NHPh

E(RPW6B95D3) = -876.693466911

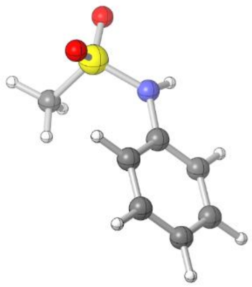

Charge = 0 Multiplicity = 1

S	1.0379182079	2.0616131631	0.3069542454
O	2.1849289657	1.5803613758	-0.4036218391

Go back to table of contents

Evaluation Only. Created with Aspose.Pdf. Copyright 2002-2014 Aspose Pty Ltd.

O	1.2129457666	2.7484594342	1.5509682896
C	0.0921424282	3.0952064593	-0.752951508
H	-0.0731411624	2.5773673821	-1.6914814901
H	-0.8497159595	3.3311539298	-0.2677571714
H	0.6658665759	4.0034406864	-0.9116514647
N	0.1216337827	0.7295781639	0.5647385317
H	0.3321256886	-0.0044509365	-0.0953789292
C	-1.1779719621	0.7028337275	1.1011224717
C	-1.6627260197	1.6882044252	1.9523547421
C	-1.9895137505	-0.3765952505	0.7683455762
C	-2.9499075317	1.5865157759	2.4516632596
H	-1.0404790177	2.5197012895	2.2358307292
C	-3.2674318834	-0.4704612217	1.2832755242
H	-1.610887263	-1.1409420379	0.1055981157
C	-3.7591579488	0.5130125395	2.1258058501
H	-3.3157959825	2.3587065024	3.1108404167
H	-3.8824585651	-1.3165044277	1.0177402204
H	-4.7586695892	0.4410118597	2.5243985701

$MeSO_2H$

E(RPW6B95D3) = -589.781494852

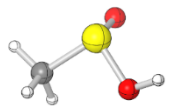

Charge = 0 Multiplicity = 1

S	-0.5874389593	2.1694317384	0.0412973061
O	0.9734947852	1.8586404008	-0.3561569044
O	-1.2766042293	2.686230697	-1.1449597032
C	-0.9928226249	0.440894007	0.1283504227

Evaluation Only. Created with Aspose.Pdf. Copyright 2002-2014 Aspose Pty Ltd.

H	-2.0616776009	0.3735451344	0.3073192712
H	-0.7356324869	-0.0129970869	-0.8248159422
H	-0.4441679617	-0.0189518925	0.9442267836
H	1.4949784577	2.6563745617	-0.1920464938

CuOBz

E(RPW6B95D3) = -2062.35333283

Charge = 0 Multiplicity = 1

Cu	-2.1258657771	-0.2683748098	1.1617188466
O	-3.6518662287	-1.6857241966	1.0148894424
O	-3.451544875	-0.263900443	-0.6580351908
C	-4.0169303461	-1.2349494449	-0.1072113415
C	-5.1675662426	-1.8925314744	-0.7963093878
C	-5.5347907242	-1.4883951792	-2.0732687501
C	-5.8771088316	-2.9089392065	-0.1704583093
C	-6.5966044163	-2.0953082902	-2.718680566
H	-4.9762338189	-0.6980849671	-2.5486527186
C	-6.9435753522	-3.5117510155	-0.8128494626
H	-5.584195087	-3.2158660023	0.8207211218
C	-7.3034880693	-3.1068527328	-2.0883000203
H	-6.8740929638	-1.7801655853	-3.7129604251
H	-7.4947313101	-4.2982662935	-0.3204812741
H	-8.133842077	-3.5791545291	-2.5908510447

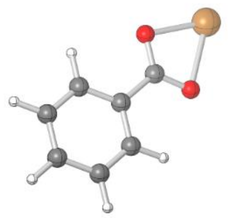

CuTC

E(RPW6B95D3) = -2383.31824229

Evaluation Only. Created with Aspose.Pdf. Copyright 2002-2014 Aspose Pty Ltd.

Charge = 0 Multiplicity = 1

Cu	-0.1555170837	-0.6751860911	-0.7113634955
O	-1.7821456914	0.3502678093	-0.6307785127
C	-2.4976031079	0.6293933925	-1.6585134803
O	-3.6949690253	0.8800044835	-1.6294051264
S	-0.0845099323	0.5877993073	-3.0721684663
C	-1.7966166329	0.6649709188	-2.9742661348
C	-2.3410368305	0.768666699	-4.2183849037
C	-1.3747573404	0.7672411915	-5.2499041947
H	-1.6093305972	0.8429824628	-6.2990145284
C	-0.1028853264	0.6658799628	-4.7768909573
H	0.8180419698	0.6583779836	-5.3321381661
H	-3.4044309019	0.8368005401	-4.3757219038

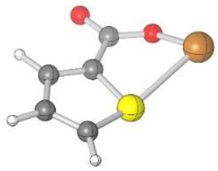

PhNH$_2$

E(RPW6B95D3) = -288.108815802

Charge = 0 Multiplicity = 1

C	-0.6814176689	1.184318352	-0.0744068048
C	0.7066847329	1.2184799337	0.0626465414
C	1.3349626975	2.4608240223	0.1563207284
C	0.5945031746	3.6268678545	0.1158089083
C	-0.7844311729	3.5889779878	-0.0189105559
C	-1.4119343297	2.3567356648	-0.1138807579
H	-1.1805150728	0.2289231773	-0.1487060542
H	2.409413501	2.5018378885	0.2625313941

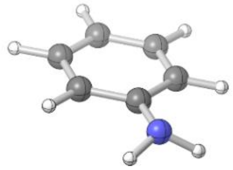

Evaluation Only. Created with Aspose.Pdf. Copyright 2002-2014 Aspose Pty Ltd.

H	1.1036450481	4.5763666794	0.1887299254
H	-1.3585865763	4.5013964879	-0.0509521099
H	-2.4850400572	2.3045159939	-0.2226994826
N	1.435962484	0.0494520394	0.1590375843
H	1.0032191163	-0.7657513996	-0.2392712206
H	2.4096734954	0.124304789	-0.079027208

BzOH

E(RPW6B95D3) = -421.539540494

Charge = 0 Multiplicity = 1

C	-1.2455510036	3.1390775623	0.6794979151
C	-0.5514812129	1.8413881246	0.55123079
C	-1.3239761931	0.6995860239	0.3779255169
C	0.8339872918	1.7425789945	0.6000112626
C	-0.7154829064	-0.5334556423	0.2532828002
H	-2.3975007483	0.7944064796	0.3424602201
C	1.4391845782	0.5061468719	0.475490636
H	1.4312560126	2.6291215378	0.7352070378
C	0.6664393291	-0.6304522385	0.3020667725
H	-1.3158899046	-1.4193818194	0.1180712895
H	2.5144499902	0.4286157597	0.5135681341
H	1.1420070171	-1.5942501778	0.2044635316
O	-0.4087548633	4.1724409546	0.8278319918
O	-2.4425070295	3.2800584661	0.655822122
H	-0.9434946272	4.9759823427	0.9055884996

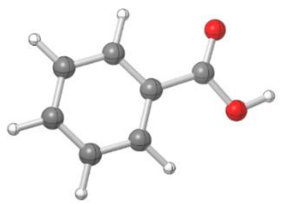

Evaluation Only. Created with Aspose.Pdf. Copyright 2002-2014 Aspose Pty Ltd.

HF

E(RPW6B95D3) = -100.602577177

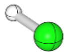

Charge = 0 Multiplicity = 1

F	-0.5693549466	1.173134	0.02310689
H	-1.4944581934	1.173134	0.02310689

***t*BuO₂Bz**

E(RPW6B95D3) = -654.298568104

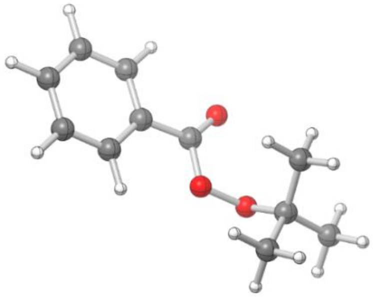

Charge = 0 Multiplicity = 1

C	-1.6105658961	3.3045679898	-0.0710394326
C	-0.7611445054	2.2166935891	0.4682102611
C	-1.3944086072	1.0341661115	0.8296559758
C	0.615250522	2.3427383328	0.62071536
C	-0.6581059398	-0.0158513653	1.3416737485
H	-2.4621774669	0.953269868	0.7039092251
C	1.3476430655	1.2881755421	1.1323423019
H	1.1112358631	3.2562966018	0.3379273276
C	0.7134725768	0.1106089499	1.4932455276
H	-1.1524149167	-0.9327169941	1.6221599959
H	2.4156498376	1.3851567974	1.2489109832
H	1.2892839391	-0.710121569	1.8924343452
O	-0.8620089348	4.4082723925	-0.3048458939
O	-2.7872933232	3.227980205	-0.2745153608
C	-1.7263901234	6.5485456969	0.0733186212
C	-2.5271817791	7.5613261549	-0.7195130046

Evaluation Only. Created with Aspose.Pdf. Copyright 2002-2014 Aspose Pty Ltd.

H	-3.5029527085	7.1613256891	-0.9821052507
H	-2.6705140814	8.4537334567	-0.1165733745
H	-2.0027575651	7.840735058	-1.629618589
C	-2.4700887175	6.1025141598	1.3146319462
H	-2.607093133	6.9516918892	1.9794847124
H	-3.4453390053	5.7013363632	1.0550495664
H	-1.9106126579	5.344833197	1.8580894452
C	-0.3413990561	7.0669446475	0.4001048422
H	-0.4196866433	7.9495290223	1.0299304691
H	0.2381770518	6.3203128291	0.9357315026
H	0.1890422215	7.3375785981	-0.509507049
O	-1.6334956468	5.4576877168	-0.8791756421

_t_BuO Radical

E(UPW6B95D3) = -233.390589832

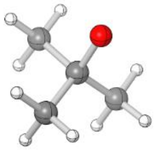

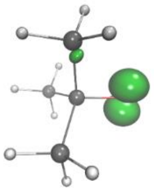

Charge = 0 Multiplicity = 2

O	-0.4875714228	4.4196324795	0.9547522457
C	-1.0600196365	5.5217686673	0.3898609742
C	-1.4287410413	5.2716191077	-1.067936132
H	-2.0751699904	4.4019533871	-1.1503420489
H	-1.943578719	6.1332364123	-1.4860733824
H	-0.5297712125	5.0934404227	-1.6535509984
C	-2.3509964261	5.6926431905	1.2314685645
H	-2.8759741856	6.5621673711	0.8433379494
H	-2.9862661996	4.8178451049	1.1382054992
H	-2.1069240467	5.85670066	2.2760102434

Evaluation Only. Created with Aspose.Pdf. Copyright 2002-2014 Aspose Pty Ltd.

C	-0.1757226273	6.7526947994	0.5528219929
H	-0.6718651016	7.6359319448	0.157841525
H	0.0525016267	6.9178115288	1.6023975222
H	0.7574685527	6.6142863639	0.0116651952

***t*BuOH**

E(RPW6B95D3) = -234.066727416

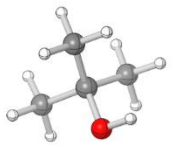

	Charge = 0	Multiplicity = 1	
O	-0.4100383995	4.4314479017	1.0181893764
C	-1.0774575818	5.5568522667	0.435252363
C	-1.3822767005	5.2630922106	-1.0238070453
H	-1.9934906021	4.3675668784	-1.1090406545
H	-1.9159659169	6.0909267038	-1.485574016
H	-0.4601872745	5.1049487344	-1.5820120072
C	-2.3551613419	5.7223353918	1.2312464992
H	-2.9339998006	6.561832157	0.8548283535
H	-2.9639273884	4.8238800386	1.160563692
H	-2.1273487903	5.9025759329	2.2792929092
C	-0.1962586899	6.787728621	0.5626945425
H	-0.6894612232	7.6654928686	0.1508001513
H	0.0351266762	6.9801355924	1.6078223876
H	0.7397306283	6.644509085	0.0238620052
H	0.413515845	4.2977656773	0.5393364131

A1

E(RPW6B95D3) = -1247.96760393

Evaluation Only. Created with Aspose.Pdf. Copyright 2002-2014 Aspose Pty Ltd.

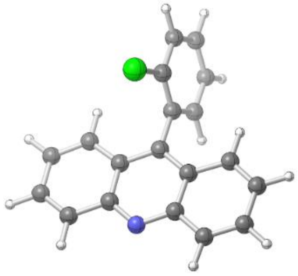

Charge = 0 Multiplicity = 1

C	-7.9348919406	-1.3268358786	-0.7669072648
C	-6.803945557	-1.7766712762	-0.16670795
C	-5.721067333	-0.896537858	0.0935284215
C	-5.8476853263	0.4782942227	-0.2802285898
C	-7.0441570563	0.905293237	-0.9141807869
C	-8.0552355729	0.0302729443	-1.1495558175
C	-4.7771352669	1.3344054275	-0.0155293525
C	-3.6313061258	0.8173627722	0.5922391302
C	-3.6084164929	-0.5742938721	0.917538606
C	-2.4471013676	-1.1124496658	1.5313570506
H	-2.4498243572	-2.1650981159	1.7680242398
C	-1.3781526629	-0.3224620816	1.8048332166
C	-1.402403183	1.0563611654	1.4869669203
C	-2.4950326221	1.6094238809	0.9010795748
H	-8.7510118798	-2.0055952898	-0.9611647163
H	-6.6899700475	-2.8085619794	0.1270841847
H	-7.137126932	1.937424022	-1.211943432
H	-8.9583321822	0.3663373179	-1.6346609732
H	-0.4998808535	-0.7425149737	2.2703352705
H	-0.543740793	1.6681656429	1.7155169445
H	-2.5109820953	2.6615757758	0.6639162039
N	-4.6302078094	-1.3923717232	0.6731609312
C	-4.8670007089	2.7731536129	-0.3526496461
C	-4.2073320626	3.324457055	-1.4440680747
C	-5.6328697428	3.623807419	0.4374169308

Evaluation Only. Created with Aspose.Pdf. Copyright 2002-2014 Aspose Pty Ltd.

C	-4.2958326234	4.671678968	-1.7432080096
C	-5.7289481256	4.9725730252	0.1533648052
H	-6.153404637	3.2098125915	1.2872213838
C	-5.0584602812	5.4966693867	-0.9383348215
H	-3.7728990441	5.0641399376	-2.6000391298
H	-6.3262051146	5.6122136452	0.7836478519
H	-5.1275931826	6.5480364836	-1.1692743129
Cl	-3.2539759499	2.3090115106	-2.4797310886

TS1

E(RPW6B95D3) = -2786.27577992

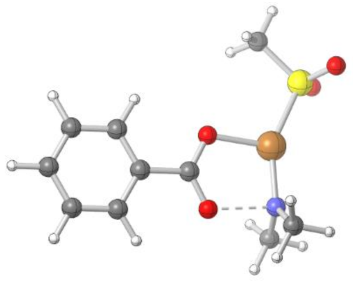

Charge = 0 Multiplicity = 1

Cu	1.9943157407	2.2445122407	0.6300881457
O	0.4209071584	1.320135665	-0.1792898051
O	0.5456761339	2.9825698046	-1.6673252057
C	0.0260849147	1.9420981547	-1.2011902793
C	-1.165164487	1.4035009153	-1.9234213156
C	-1.6416556537	2.0445233297	-3.0598432471
C	-1.8009512914	0.2622467682	-1.4527404372
C	-2.7480162422	1.5464178081	-3.7209771116
H	-1.1408721026	2.9297796088	-3.4160444419
C	-2.9080049282	-0.2327200882	-2.1165682978
H	-1.42214008	-0.2258064563	-0.569798447
C	-3.3821055195	0.4081283854	-3.2498177394
H	-3.1168583884	2.0459868083	-4.6032674793
H	-3.4021202163	-1.1187858597	-1.7494802116

Evaluation Only. Created with Aspose.Pdf. Copyright 2002-2014 Aspose Pty Ltd.

H	-4.2469676348	0.0203957782	-3.7658992238
N	2.1971939253	3.6584986934	-0.5405284586
C	1.6197844386	4.9455914445	-0.2961619494
H	1.3215500721	5.4435715389	-1.2153841391
H	0.7928208402	4.8653019577	0.3992755894
H	2.405154054	5.5413313715	0.1802246909
C	3.1032385096	3.618883622	-1.6486492878
H	4.0272163347	4.0912194299	-1.2999320456
H	3.3317327214	2.5940335015	-1.9158322281
H	2.732997408	4.1809125314	-2.5024437604
S	2.590017976	1.1667438613	2.4838052295
O	2.4062649388	2.0108844503	3.6716541512
O	3.911159365	0.5398129135	2.3479531683
C	1.4515708058	-0.1928657146	2.7007726109
H	1.7515344614	-0.7113290011	3.608785891
H	0.4449665378	0.2003826902	2.7999741863
H	1.5216052979	-0.8532705424	1.8424616563

TS2

E(RPW6B95D3) = -2786.30432234

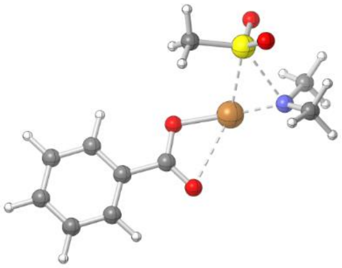

Charge = 0 Multiplicity = 1

Cu	-1.2530666675	0.8539608681	-0.370810643
O	-2.4135495978	-0.587006042	-0.753096206
O	-2.3673677236	0.3208641701	-2.7755484865
C	-2.741178274	-0.5545599412	-1.997958048
C	-3.6140449632	-1.6702280511	-2.4704347234

Evaluation Only. Created with Aspose.Pdf. Copyright 2002-2014 Aspose Pty Ltd.

C	-3.9913246977	-1.7189662018	-3.8060819121
C	-4.0459597674	-2.6627353513	-1.6004688708
C	-4.789931686	-2.748444579	-4.2679859511
H	-3.6476775392	-0.9422717804	-4.4705498253
C	-4.8460807625	-3.6924850667	-2.0620229974
H	-3.7496845044	-2.6188695908	-0.5650210422
C	-5.2181614725	-3.7369076814	-3.3959314046
H	-5.0785696243	-2.7820408294	-5.3073073893
H	-5.1796692449	-4.4615808458	-1.3824495866
H	-5.8412393271	-4.5413321135	-3.75606054
N	-0.1278564094	2.2233541899	0.0462474955
C	-0.320823208	3.0963709702	1.1709392005
H	0.549352001	3.1426364396	1.8264703136
H	-0.5039375107	4.0988170802	0.7751123579
H	-1.1943475776	2.8032882912	1.7421773614
C	1.1300158228	2.3937992745	-0.6273724696
H	1.9749647004	2.4381590044	0.0587842939
H	1.2869821257	1.6054132893	-1.3555632452
H	1.0792782186	3.3407498242	-1.1712081038
S	0.3345190376	0.1855736168	1.2929590875
O	-0.0586078872	0.671566432	2.5939346304
O	1.7311689287	0.1342768525	0.9344899923
C	-0.268453949	-1.4868286981	1.1662942471
H	0.3055151556	-2.041302819	1.9060850536
H	-1.3264336518	-1.494180927	1.3945278892
H	-0.0752556547	-1.8490921244	0.1640246821

Evaluation Only. Created with Aspose.Pdf. Copyright 2002-2014 Aspose Pty Ltd.

TS3

E(RPW6B95D3) = -2786.26523090

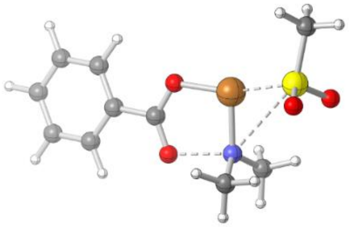

	Charge = 0	Multiplicity = 1	
Cu	1.5692513722	1.7714413633	0.3645342413
O	0.0772204611	1.1346889711	-0.8022474649
O	0.0262740203	3.0706677301	-1.941990607
C	-0.3633674655	1.9114610912	-1.7054522538
C	-1.4645956555	1.3706796354	-2.5688072115
C	-1.9597742273	2.1294367512	-3.6210407048
C	-1.9945744899	0.1120874787	-2.3205392444
C	-2.9743136111	1.6338142887	-4.4186877381
H	-1.5406284674	3.1056613408	-3.8046516281
C	-3.0124041156	-0.3818166769	-3.1163428756
H	-1.6023726052	-0.4685867437	-1.5015346952
C	-3.502654606	0.3777662009	-4.1663991789
H	-3.3542272852	2.2260534579	-5.2369422474
H	-3.4238107581	-1.3597599349	-2.9189050276
H	-4.2958768658	-0.0087010569	-4.7880077306
N	1.6542047636	3.5021304349	-0.4440166425
C	1.1462044454	4.5957475909	0.3050495961
H	1.7833269142	4.6815544299	1.2001307657
H	1.2013149356	5.5373589188	-0.2369332216
H	0.1385388496	4.3836409901	0.6483873411
C	2.7761957087	3.8138164195	-1.2606563099
H	2.9674661232	3.0122946008	-1.9657624687
H	2.6612855059	4.7728599757	-1.7626638161

Evaluation Only. Created with Aspose.Pdf. Copyright 2002-2014 Aspose Pty Ltd.

H	3.6487282034	3.8943415875	-0.5960692392
S	3.1224167061	2.0166652613	1.9227304029
O	2.745511332	3.1227158745	2.8087767044
O	4.4379797089	2.1276742292	1.2881908343
C	3.2046359753	0.5742428475	2.9688707582
H	3.9800794534	0.7656229234	3.7069988911
H	2.2409235226	0.4380329064	3.4481760622
H	3.4583266081	-0.2843245873	2.3561937076

TS4

E(RPW6B95D3) = -2939.02508232

Charge = 0 Multiplicity = 1

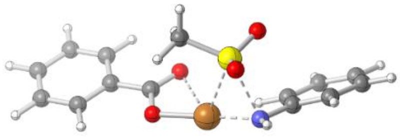

Cu	-2.004252321	-1.188145027	1.546089479
O	-3.494437993	-2.121380787	0.834981745
O	-3.521930147	-0.324211119	-0.460136633
C	-3.985777361	-1.414245695	-0.118603412
C	-5.187461883	-1.975337412	-0.802799824
C	-5.827617325	-1.22368209	-1.779568249
C	-5.679299472	-3.231627116	-0.47302785
C	-6.949060972	-1.719993004	-2.417512877
H	-5.43491967	-0.249898966	-2.025217579
C	-6.79783182	-3.730903441	-1.115588772
H	-5.177430486	-3.808823406	0.28652291
C	-7.434840124	-2.975189809	-2.086712356
H	-7.445196356	-1.129904064	-3.172678824
H	-7.174767499	-4.708950076	-0.858767371

Evaluation Only. Created with Aspose.Pdf. Copyright 2002-2014 Aspose Pty Ltd.

H	-8.309535883	-3.363962642	-2.585481415
N	-0.568387374	-0.303583129	2.277514385
S	-2.569616523	0.658252639	3.089522662
O	-2.329921076	1.984223621	2.583145485
O	-2.282508648	0.338237352	4.468596422
C	-4.289317242	0.294427522	2.798052829
H	-4.493702948	0.477626037	1.750237815
H	-4.475029876	-0.734927827	3.079508466
H	-4.835023588	0.98562705	3.437717033
H	-0.345821993	-0.418713608	3.258671068
C	0.074166597	0.760583595	1.700819446
C	0.88638162	1.610420283	2.458808054
C	-0.096414293	1.006056668	0.334310205
C	1.539506896	2.660043875	1.850454394
H	0.998130988	1.42768319	3.517427451
C	0.532365409	2.079382756	-0.255189574
H	-0.738770918	0.349851703	-0.235689625
C	1.359403756	2.903768077	0.496326613
H	2.178156583	3.303599289	2.435016479
H	0.388406964	2.272889004	-1.306566574
H	1.860533088	3.736027688	0.027503714

TS5

E(RPW6B95D3) = -3136.92853840

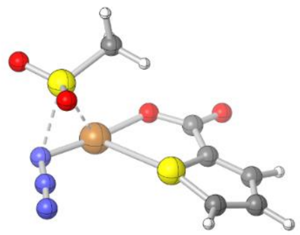

Charge = 0 Multiplicity = 1

Cu	-0.1153319829	-0.9437905853	-0.5199113439

Go back to table of contents

Evaluation Only. Created with Aspose.Pdf. Copyright 2002-2014 Aspose Pty Ltd.

O	-1.8069345832	-0.1813694663	-0.4873758437
C	-2.647466509	0.0379742292	-1.4454051831
O	-3.8292497261	0.2642306502	-1.2542885897
S	-0.4718265629	-0.0453093507	-3.2533555581
C	-2.1396668608	0.030761702	-2.8422133346
C	-2.9013916293	0.1008152856	-3.9713924455
C	-2.1409520855	0.082522457	-5.159392575
H	-2.5634813935	0.1273430628	-6.1493907788
C	-0.8029467337	0.0049406895	-4.9229349171
H	0.0035279752	-0.0166851032	-5.6341711763
H	-3.9758893661	0.1603022582	-3.9306504869
N	1.6707408902	-1.477055077	-0.5298810729
N	2.2377705111	-1.5530223868	-1.6044379434
N	2.8284119975	-1.6280630339	-2.5576594888
S	1.6874558113	0.7919058707	-0.137565526
O	2.4809490901	1.0902628984	-1.2871003603
O	2.2303775615	0.6139998091	1.1676358315
C	0.3507199449	1.962737072	-0.0467909824
H	-0.1302332253	2.0112088461	-1.0152624479
H	-0.3225540929	1.649620263	0.7409890839
H	0.8519810696	2.8994932694	0.1988561388

TS6

E(RPW6B95D3) = -2786.27859772

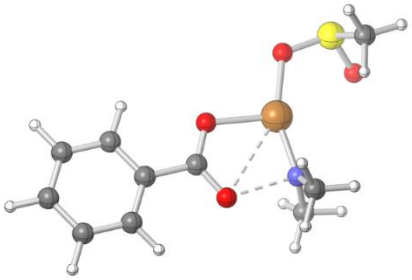

Charge = 0 Multiplicity = 1

Cu	1.1029272001	1.4666494312	0.9937309473

Go back to table of contents

Evaluation Only. Created with Aspose.Pdf. Copyright 2002-2014 Aspose Pty Ltd.

O	-0.3934040166	0.8185435382	-0.1809663846
O	0.2792726157	2.5119520816	-1.4690009377
C	-0.4676777406	1.5368768027	-1.2074536185
C	-1.5301111318	1.2119598255	-2.2067607502
C	-1.6548602198	1.9539716846	-3.3740665916
C	-2.3982154243	0.156697977	-1.9596136535
C	-2.6427858756	1.6400357981	-4.288557471
H	-0.9762440707	2.7711965129	-3.5566182597
C	-3.3855428548	-0.1535062916	-2.8757642553
H	-2.2907620796	-0.4100491069	-1.0495170027
C	-3.508542399	0.5870525068	-4.0402399366
H	-2.7379724669	2.2166480719	-5.195486512
H	-4.0598350452	-0.9730307553	-2.6814588746
H	-4.2799209635	0.3438946061	-4.754724242
N	1.7463052504	2.8635028369	0.0059989601
C	1.3169269889	4.2138237527	0.2184502422
H	1.2690379465	4.7824322102	-0.7062910085
H	0.3726284279	4.2322429452	0.7487917726
H	2.0745306332	4.6640814171	0.8677065947
C	2.8207999975	2.7270091085	-0.932531116
H	3.7218160525	3.0413071633	-0.3962036427
H	2.9463208345	1.6893765718	-1.2175495431
H	2.6948684492	3.3670653155	-1.8018334188
S	1.917717843	0.5581281037	3.7159030476
O	1.9894034046	2.0119008031	4.0082281607
O	1.0024000293	0.3026239269	2.4968644056
C	3.5200879484	0.2149675229	2.9747273593

Evaluation Only. Created with Aspose.Pdf. Copyright 2002-2014 Aspose Pty Ltd.

H	3.638611836	0.9001158785	2.1353557835
H	4.2942189693	0.3902279876	3.7165452748
H	3.5494067815	-0.8168613468	2.6363967706

TS7

E(RPW6B95D3) = -2786.28375048

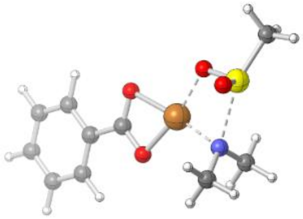

Charge = 0 Multiplicity = 1

Cu	-1.094162	0.581598	0.260502
O	-2.286171	-1.179606	0.292917
O	-1.98853	0.1157	-1.434992
C	-2.52161	-0.915715	-0.909447
C	-3.404909	-1.773969	-1.72559
C	-3.665426	-1.451375	-3.051405
C	-3.977257	-2.906951	-1.161537
C	-4.494707	-2.257982	-3.807553
H	-3.214692	-0.569498	-3.477464
C	-4.805454	-3.712215	-1.91992
H	-3.765236	-3.144457	-0.131376
C	-5.064543	-3.387826	-3.242325
H	-4.697518	-2.007606	-4.837209
H	-5.24964	-4.592494	-1.482004
H	-5.711664	-4.017176	-3.833905
N	-0.261809	2.280858	0.005027
C	0.710995	2.344806	-1.049039
H	0.239294	2.201101	-2.026838
H	1.171012	3.335289	-1.066572

Evaluation Only. Created with Aspose.Pdf. Copyright 2002-2014 Aspose Pty Ltd.

H	1.477732	1.591585	-0.903826
C	-1.256113	3.314573	-0.122582
H	-1.815742	3.204018	-1.056174
H	-1.945068	3.285622	0.716697
H	-0.774027	4.293336	-0.157319
S	0.601996	1.821461	2.276468
O	1.993668	1.443118	2.011048
O	-0.375564	0.67582	2.109534
C	0.4706	2.037052	4.061322
H	0.790033	1.111132	4.53044
H	1.125651	2.857384	4.333562
H	-0.560268	2.267877	4.305363

TS8

E(RPW6B95D3) = -2786.26209095

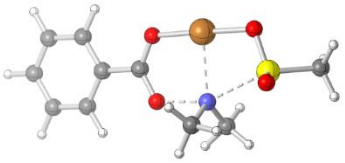

Charge = 0 Multiplicity = 1

Cu	1.4804661197	0.8026253346	0.810302858
O	0.1436510258	0.5074630062	-0.5270778753
O	0.3217722319	2.6168068879	-1.2662904985
C	-0.1789506138	1.4570308783	-1.2751932371
C	-1.2580718157	1.2185683824	-2.2782256307
C	-1.658795228	2.2281444738	-3.143904103
C	-1.8690014533	-0.027156082	-2.3393260099
C	-2.6638790355	1.9922331169	-4.0630042761
H	-1.1794914169	3.1920445485	-3.0906214063
C	-2.8738371609	-0.2602293345	-3.2593314613

Evaluation Only. Created with Aspose.Pdf. Copyright 2002-2014 Aspose Pty Ltd.

H	-1.5497213073	-0.802439034	-1.6622030673
C	-3.2724305851	0.7487826108	-4.1213453021
H	-2.9736649212	2.778205905	-4.7341220107
H	-3.3472393743	-1.2287172825	-3.3044064754
H	-4.0576161386	0.5661952052	-4.8388818523
N	1.7622136315	3.0075812279	0.02048252
C	1.1253233821	4.2235435296	0.4982340767
H	1.8892255928	4.9357886698	0.7983973635
H	0.550195912	4.6831359142	-0.3009087275
H	0.4810558594	3.9939298037	1.3397700637
C	2.7322448271	3.2674590565	-1.0174286385
H	2.9322164384	2.3540196991	-1.5654572
H	2.4164677315	4.0612148247	-1.6907432497
H	3.6593858222	3.5729275189	-0.5276040257
S	3.397390712	2.529356057	1.92042405
O	4.7561175349	2.5051496223	1.3534604853
O	2.8273930504	1.1389599179	2.1542294409
C	3.6092107532	3.1023603387	3.6053845483
H	4.2817856242	2.4044935261	4.0979924369
H	4.0417064689	4.0972088148	3.5760064683
H	2.6412048027	3.1187537124	4.0952620459

TS9

E(RPW6B95D3) = -2939.00099263

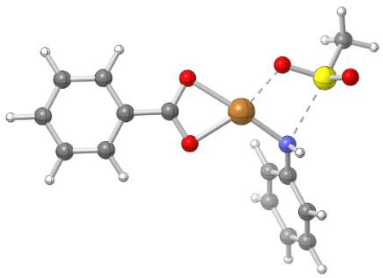

Charge = 0 Multiplicity = 1

Cu	-1.1353421886	0.5663606698	0.0828562892

Evaluation Only. Created with Aspose.Pdf. Copyright 2002-2014 Aspose Pty Ltd.

O	-2.0288199135	-1.3468278523	0.1496139962
O	-2.3444737836	0.0786737569	-1.4710923714
C	-2.5821440866	-1.0458977195	-0.9371981259
C	-3.5140828879	-1.9892224021	-1.5960274415
C	-4.1231506744	-1.6478203772	-2.7966745032
C	-3.784200341	-3.2182302233	-1.0080011655
C	-4.99686929	-2.5301658599	-3.4040532503
H	-3.9048197396	-0.6908265675	-3.2428413891
C	-4.6580176328	-4.0992170114	-1.6167273117
H	-3.3040501311	-3.4695824897	-0.0758623163
C	-5.2648435296	-3.7554878774	-2.8144431232
H	-5.4699011864	-2.2641548392	-4.3366295178
H	-4.8674024524	-5.0534155779	-1.1585922506
H	-5.9476549537	-4.4435917019	-3.2889516505
N	-0.6433728118	2.4196853224	0.0785108047
S	0.77499361	1.717538482	2.0928093886
O	2.2146650237	1.776433622	1.8213245271
O	0.1257833297	0.4189674723	1.7231885206
C	0.5813187204	1.7358740479	3.8780018189
H	1.1182532494	0.8764066954	4.2698925085
H	1.0067585334	2.6635329976	4.2440987216
H	-0.4773004771	1.676213255	4.1047021177
H	0.244639591	2.5272437513	-0.4096209084
C	-1.4716034867	3.4399489873	-0.2798456681
C	-2.7973452849	3.4280641981	0.1861936792
C	-1.037532205	4.5082666812	-1.0829167495
C	-3.6562685758	4.4473119136	-0.148014667

Evaluation Only. Created with Aspose.Pdf. Copyright 2002-2014 Aspose Pty Ltd.

H	-3.1205890349	2.6009045759	0.8004602946
C	-1.9010241742	5.5314427116	-1.3974363892
H	-0.0190303406	4.5105623105	-1.4424573266
C	-3.2111755655	5.503207231	-0.9353564586
H	-4.6757174516	4.4303354109	0.2034886718
H	-1.5635523748	6.352066249	-2.0107320322
H	-3.8869284835	6.3044311577	-1.1903527218

TS10

E(RPW6B95D3) = -3136.90288346

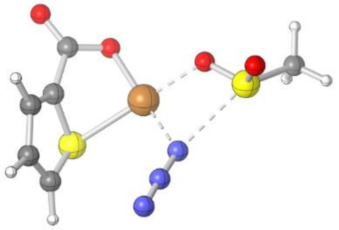

Charge = 0 Multiplicity = 1

Cu	-0.3772762341	0.1260710399	-0.8788705435
O	-2.0910259295	0.8674874599	-0.4060273588
C	-2.9253067067	0.6112771315	-1.3564775215
O	-4.134475152	0.6241694519	-1.2756357691
S	-0.7989204439	1.1749858079	-3.0685784015
C	-2.2320382783	0.2613084837	-2.6331446462
C	-2.4382565771	-0.7419478086	-3.5225702173
C	-1.4528160882	-0.7868469643	-4.5373350449
H	-1.4543295738	-1.5033961682	-5.3419755737
C	-0.4962194184	0.1761323087	-4.4081872446
H	0.3361815338	0.3768791994	-5.0580018632
H	-3.259240909	-1.4344423463	-3.4409720683
N	1.3000574779	-0.6501051449	-1.3125237825
N	1.5016380467	-1.2278732225	-2.347624655
N	1.7271719308	-1.7851738999	-3.3099293568

Evaluation Only. Created with Aspose.Pdf. Copyright 2002-2014 Aspose Pty Ltd.

S	1.2179532191	-1.2508510117	1.2906674222
O	0.9705611206	-2.6730585675	1.4917294775
O	-0.0015553447	-0.4142353468	1.0471997245
C	1.7950538913	-0.5562695712	2.8503104413
H	2.7089718064	-1.0801807134	3.1049220444
H	1.9719941435	0.5009394285	2.6921502144
H	1.0127873953	-0.7308474663	3.5828665727

DABCO

E(RPW6B95D3) = -345.917772549

Charge = 0 Multiplicity = 1

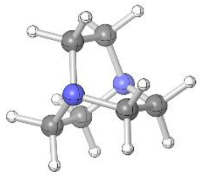

C	-1.2483988393	0.8851799918	-0.0375017948
C	-1.7729443768	2.3369918865	0.0379336382
H	-1.5627935762	0.4001806773	-0.9597323737
H	-1.6168913339	0.2889287388	0.795076316
H	-2.4375222353	2.5596413913	-0.7947162955
H	-2.3245012202	2.5092039231	0.9602537429
C	0.7167452385	1.5947708627	-1.1683681456
H	1.8031108317	1.6164948795	-1.1058030842
H	0.4452967866	1.0333032144	-2.0603150962
C	0.1301241634	3.0241985622	-1.2065088136
H	0.9183936384	3.7722382282	-1.266487875
H	-0.5234524227	3.158876609	-2.0662705792
C	0.19204921	3.0471128713	1.1680797132
H	1.0409663051	3.7253314495	1.1057089596
H	-0.3760902632	3.30501868	2.0598500962

Evaluation Only. Created with Aspose.Pdf. Copyright 2002-2014 Aspose Pty Ltd.

C	0.6550130356	1.5730376982	1.2063274682
H	1.7393934688	1.5018084317	1.2660179906
H	0.2387124682	1.0518133936	2.0662638985
N	-0.6536075066	3.2734419033	-0.0003810737
N	0.2113634279	0.8798450076	0.0002173084

***DABCO-H⁺**

E(RPW6B95D3) = -346.383740101

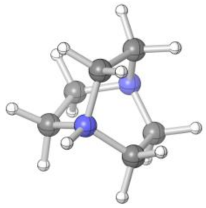

Charge = 1 Multiplicity = 1

C	-0.1205497968	3.8568035179	0.7088539856
C	-0.542667199	5.3072554563	0.9886870518
H	-0.3979797112	3.5220576678	-0.284699055
H	-0.4970601482	3.1550229055	1.4438309259
H	-1.2987272749	5.6140941689	0.2731842889
H	-0.9608444598	5.3985708217	1.9864704908
C	1.9479461289	4.5760977474	-0.3779367958
H	3.0198106244	4.6168768431	-0.2190270609
H	1.7382287111	4.0108473094	-1.2784404514
C	1.290154223	5.9643480719	-0.3738991919
H	2.0463947847	6.7285925454	-0.5217560736
H	0.562426719	6.045846866	-1.1754497551
C	1.5231805339	5.9231792806	1.9896014501
H	2.4297907717	6.4984756224	1.8286939361
H	1.0754354512	6.2466266105	2.9237711104
C	1.8396948713	4.4217888097	2.0581807893
H	2.899593034	4.2145399169	2.1477665718

Evaluation Only. Created with Aspose.Pdf. Copyright 2002-2014 Aspose Pty Ltd.

H	1.3062120574	3.9152578929	2.8544689474
N	0.6068935914	6.1965676261	0.8910888501
N	1.370880145	3.821902987	0.773608009
H	1.6812453029	2.8561380326	0.7267847166

***Br- anion**

E(RPW6B95D3) = -2575.44819749

Charge = -1   Multiplicity = 1

Br	-1.57958556	2.34217649	0.00000000

4-Bromo-1-(methylsulfonyl)butane-2-sulfinic acid

E(RPW6B95D3) = -3871.20157154

Charge = 0   Multiplicity = 1

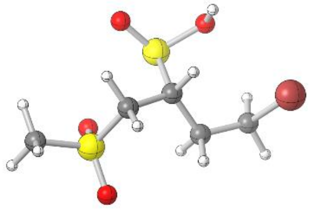

S	-2.7765567174	-0.6094864324	-0.1033405084
O	-2.6698060909	-2.0118072768	-0.4190180606
O	-2.7031669691	0.3383801661	-1.1912280521
C	-1.5111681599	-0.166950412	1.0718055731
H	-1.8804945602	0.6951802644	1.6206553168
H	-1.4055100303	-1.0059156798	1.754543094
C	-0.1797655921	0.1639292268	0.4237542174
H	0.5224278342	0.3239251689	1.2429094493
S	-0.2737735225	1.809494181	-0.4070362397
O	-1.067371286	2.6377038663	0.5069639666
O	1.2900132474	2.2105683204	-0.2245163007
H	1.5476093966	2.2069301087	0.7118748123

Evaluation Only. Created with Aspose.Pdf. Copyright 2002-2014 Aspose Pty Ltd.

C	0.3410092097	-0.9121512417	-0.5172740497
H	-0.2724495476	-0.9367155292	-1.4193403948
H	0.2081711592	-1.876850881	-0.0320193289
C	1.7701800619	-0.7729432618	-0.9749015913
H	1.9706930168	0.1507507188	-1.4992769329
H	2.0519570978	-1.6114863671	-1.5973904477
Br	3.0392567668	-0.7969866054	0.5212029675
C	-4.2709133103	-0.3471889485	0.7844409492
H	-4.2677625555	-0.9675905689	1.6750210452
H	-4.3581335181	0.7042229699	1.0351829737
H	-5.0765271081	-0.648708468	0.120736985

cis-3-((Methylsulfonyl)methyl)-1,2-oxathiolane 2-oxide

E(RPW6B95D3) = -1295.71285890

Charge = 1 Multiplicity = 1

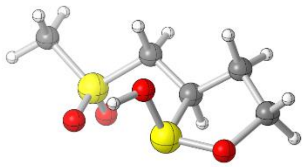

C	0.7425663485	0.7846359492	-0.0112895446
S	1.353728182	-0.8874508889	-0.376240264
O	1.304893297	-1.4527618965	1.1048033954
H	1.184520322	-2.418620742	1.0969097226
O	2.8266377647	-0.4250415282	-0.5871697401
C	3.1662002524	0.901210942	-0.0250411206
H	3.4217751987	1.5110339054	-0.88292571
H	4.027682124	0.7530696082	0.6122566635
C	1.9426192577	1.3831259722	0.7096521603
H	1.8781369384	2.4658624481	0.6780847162
H	1.9505714479	1.0612824928	1.7465074974

Evaluation Only. Created with Aspose.Pdf. Copyright 2002-2014 Aspose Pty Ltd.

H	0.6200778772	1.2254384039	-1.000465645
C	-0.5385623811	0.8150788817	0.7865788042
H	-0.4092896053	0.3463548901	1.7586062761
H	-0.8549481464	1.8457722591	0.9232833567
S	-1.8288276196	-0.0562603934	-0.0802644009
O	-2.2457305123	0.7241627844	-1.2118446624
O	-1.2907505154	-1.3798583103	-0.3265166928
C	-3.1404869436	-0.1725886958	1.0740856187
H	-2.8037901179	-0.7195461976	1.9477848955
H	-3.9364735393	-0.7143423018	0.5697692188
H	-3.4719265252	0.8280236689	1.3314882983

***trans*-3-((Methylsulfonyl)methyl)-1,2-oxathiolane 2-oxide**

E(RPW6B95D3) = -1295.71132693

Charge = 1 Multiplicity = 1

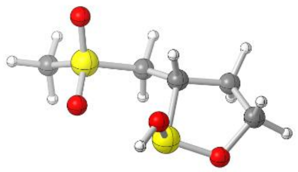

C	0.6258237579	-0.7784335611	-0.339040461
S	1.2877795764	0.8051776636	0.3215745429
O	1.3160014231	1.587850145	-1.0556927925
H	1.3236899077	2.5488210196	-0.8977256418
O	2.7483824549	0.2801677252	0.5290680778
C	3.0411548941	-0.926239392	-0.2598647269
H	3.271424178	-0.6186538406	-1.2746588735
H	3.9066616915	-1.3739267958	0.209443235
C	1.7845701143	-1.7442785948	-0.147790796
H	1.7271600271	-2.2130209299	0.8302299385
H	1.7457179708	-2.5118444071	-0.9146248688

Evaluation Only. Created with Aspose.Pdf. Copyright 2002-2014 Aspose Pty Ltd.

H	0.4560482595	-0.5603953671	-1.3919399459
C	-0.6516721844	-1.1900093157	0.3569827929
H	-0.9636623867	-2.1660800256	-0.0038279276
H	-0.5352924255	-1.2315197231	1.4371736548
S	-1.9330630191	-0.0134934072	-0.0350275898
O	-2.408429815	-0.2675080935	-1.366287721
O	-1.3472572675	1.2835896121	0.2429275741
C	-3.2095278475	-0.3303497347	1.1215741776
H	-2.8331827767	-0.16653538	2.1253791465
H	-4.0054061671	0.3736950933	0.8932759291
H	-3.5597236655	-1.3489022707	0.9884786153

TScis

E(RPW6B95D3) = -4217.12644010

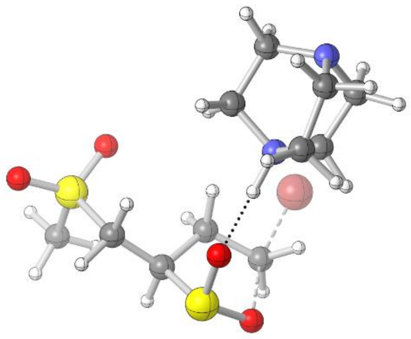

Charge = 0 Multiplicity = 1

O	-0.3045227022	0.0794667308	1.6522219157
C	-0.1994138983	2.1046319369	0.243316323
C	-1.6384392611	2.0248798606	0.7238839116
H	0.4391916723	2.5026072942	1.0276228198
H	-0.1606416007	2.7901073173	-0.5964883602
H	-2.3116748633	1.8627437589	-0.1165674233
S	-1.768473558	0.4785223582	1.7473684043
C	-2.0382582125	3.25445784	1.5141433294
H	-1.7288732998	3.1848343208	2.555840131
H	-1.6243235534	4.1644185253	1.0869011854
S	-3.8048153696	3.4420091813	1.4886698329

Evaluation Only. Created with Aspose.Pdf. Copyright 2002-2014 Aspose Pty Ltd.

O	-4.3899633243	2.1646255597	1.8280081238
O	-4.1878517479	4.031315378	0.2282974427
C	-4.14396942	4.5868926203	2.7777019869
H	-3.6083763784	5.5101415787	2.5817170091
H	-3.8465823938	4.1530175105	3.7262325379
O	-2.5936813289	-0.421157164	0.8734868535
H	-4.0978729018	-0.1396807487	0.2008824818
H	-5.215727775	4.7635443741	2.7601475556
H	1.2979289742	0.4430161491	0.086433853
H	-0.3399668753	0.0527106722	-0.6362344353
Br	1.1732686888	1.2271357097	-2.4539117155
C	0.3051305015	0.7592614825	-0.1548917295
C	-7.311502351	-0.4388715614	-0.7266352742
C	-6.1972709667	-0.0837818059	0.2693575019
H	-8.1187263067	0.2833648458	-0.6502349388
H	-7.7179522574	-1.4241809638	-0.5169992773
H	-6.2606200562	0.937340681	0.6238794205
H	-6.1646667024	-0.7527978319	1.1221421368
C	-6.076645413	0.8050957004	-2.3290887441
H	-5.8278483364	0.8815445263	-3.3831405871
H	-6.7362301346	1.6301109593	-2.0756326936
C	-4.7960251107	0.8565413639	-1.4824995978
H	-3.9027272797	0.6458272505	-2.0613345577
H	-4.6715647104	1.8015914596	-0.9669208264
C	-4.8301024362	-1.5452869937	-1.1099422114
H	-3.814204836	-1.6785278493	-1.463472976
H	-5.0364117765	-2.2842136075	-0.3431174335

Evaluation Only. Created with Aspose.Pdf. Copyright 2002-2014 Aspose Pty Ltd.

C	-5.8672973929	-1.5577210761	-2.242010665
H	-5.3818169924	-1.4665812378	-3.2093393398
H	-6.4217992483	-2.4911517206	-2.2274513211
N	-6.792808093	-0.4418666972	-2.0892116941
N	-4.906074912	-0.211841738	-0.4544910951

TStrans

E(RPW6B95D3) = -4217.12099351

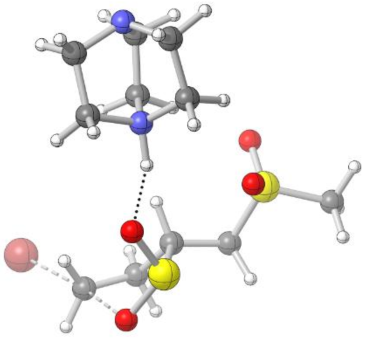

Charge = 0 Multiplicity = 1

O	-0.3045227022	0.0794667308	1.6522219157
C	-0.1994138983	2.1046319369	0.243316323
C	-1.6384392611	2.0248798606	0.7238839116
H	0.4391916723	2.5026072942	1.0276228198
H	-0.1606416007	2.7901073173	-0.5964883602
H	-2.3116748633	1.8627437589	-0.1165674233
S	-1.768473558	0.4785223582	1.7473684043
C	-2.0382582125	3.25445784	1.5141433294
H	-1.7288732998	3.1848343208	2.555840131
H	-1.6243235534	4.1644185253	1.0869011854
S	-3.8048153696	3.4420091813	1.4886698329
O	-4.3899633243	2.1646255597	1.8280081238
O	-4.1878517479	4.031315378	0.2282974427
C	-4.14396942	4.5868926203	2.7777019869
H	-3.6083763784	5.5101415787	2.5817170091
H	-3.8465823938	4.1530175105	3.7262325379
O	-2.5936813289	-0.421157164	0.8734868535

Evaluation Only. Created with Aspose.Pdf. Copyright 2002-2014 Aspose Pty Ltd.

H	-4.0978729018	-0.1396807487	0.2008824818
H	-5.215727775	4.7635443741	2.7601475556
H	1.2979289742	0.4430161491	0.086433853
H	-0.3399668753	0.0527106722	-0.6362344353
Br	1.1732686888	1.2271357097	-2.4539117155
C	0.3051305015	0.7592614825	-0.1548917295
C	-7.311502351	-0.4388715614	-0.7266352742
C	-6.1972709667	-0.0837818059	0.2693575019
H	-8.1187263067	0.2833648458	-0.6502349388
H	-7.7179522574	-1.4241809638	-0.5169992773
H	-6.2606200562	0.937340681	0.6238794205
H	-6.1646667024	-0.7527978319	1.1221421368
C	-6.076645413	0.8050957004	-2.3290887441
H	-5.8278483364	0.8815445263	-3.3831405871
H	-6.7362301346	1.6301109593	-2.0756326936
C	-4.7960251107	0.8565413639	-1.4824995978
H	-3.9027272797	0.6458272505	-2.0613345577
H	-4.6715647104	1.8015914596	-0.9669208264
C	-4.8301024362	-1.5452869937	-1.1099422114
H	-3.814204836	-1.6785278493	-1.463472976
H	-5.0364117765	-2.2842136075	-0.3431174335
C	-5.8672973929	-1.5577210761	-2.242010665
H	-5.3818169924	-1.4665812378	-3.2093393398
H	-6.4217992483	-2.4911517206	-2.2274513211
N	-6.792808093	-0.4418666972	-2.0892116941
N	-4.906074912	-0.211841738	-0.4544910951

Evaluation Only. Created with Aspose.Pdf. Copyright 2002-2014 Aspose Pty Ltd.

Anilinium

E(RPW6B95D3) = -288.549609718

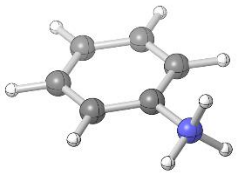

	Charge = 1	Multiplicity = 1	
C	-0.7004496776	1.1921768662	-0.0559599866
C	0.6672518271	1.2743996801	0.077916682
C	1.334361294	2.4779867638	0.1586576609
C	0.5943163295	3.6446213364	0.1041781496
C	-0.7835593128	3.5896832188	-0.0283551592
C	-1.4289977229	2.3682959245	-0.1089245829
H	-1.1881799918	0.2310669072	-0.1158280501
H	2.4080760309	2.5048898323	0.2617383089
H	1.0972195912	4.5963381454	0.164506696
H	-1.3553573136	4.5030843992	-0.0701287239
H	-2.5010582444	2.3250985511	-0.2144072305
N	1.445630853	0.0351855082	0.155029104
H	0.8854705201	-0.7697577189	-0.1233047472
H	2.2622446612	0.0668448606	-0.4556229892
H	1.7821601561	-0.139236275	1.1030346802

PhNH Anion

E(RPW6B95D3) = -287.580271143

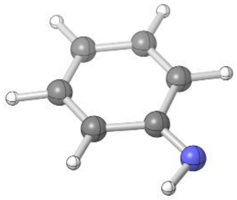

	Charge = -1	Multiplicity = 1	
C	-0.6769184853	1.1903995803	-0.1267049428
C	0.7483156046	1.1731289609	0.0043767464

Evaluation Only. Created with Aspose.Pdf. Copyright 2002-2014 Aspose Pty Ltd.

C	1.3376427404	2.4701851154	0.1322712661
C	0.5903454718	3.6259035689	0.1300064623
C	-0.7978822717	3.6039028799	0.0018237776
C	-1.4078222013	2.358825263	-0.1271517064
H	-1.1882783774	0.2407954735	-0.2290425867
H	2.4134699395	2.5228847258	0.2350531514
H	1.1002718697	4.5753772247	0.232136925
H	-1.3760157453	4.5146329083	0.001377137
H	-2.4840076362	2.3001330824	-0.2300584171
N	1.5056422349	0.080081245	0.0143512859
H	0.8994513864	-0.7288953181	-0.0867939285

$MeSO_2NHPh$ radical anion

E(UPW6B95D3) = -876.724883577

Charge = -1 Multiplicity = 2

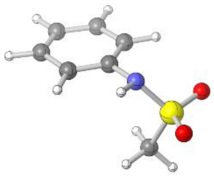

S	1.0072846419	2.122504285	0.3059041389
O	2.194507939	1.6803153853	-0.3764675657
O	1.146883875	3.0648581468	1.3781889111
C	-0.1030074104	2.8167372306	-0.871492465
H	-0.22664061	2.1199777613	-1.6931575071
H	-1.0515553754	3.0068915149	-0.3797607544
H	0.3348078804	3.74715344	-1.2192685947
N	0.2956951181	0.7610430275	0.8585842318
H	0.5433399887	-0.0186843952	0.2653813565
C	-1.0783863444	0.7415975831	1.2652547022
C	-1.51708997	1.5882350547	2.2591742092

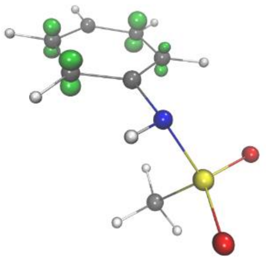

Evaluation Only. Created with Aspose.Pdf. Copyright 2002-2014 Aspose Pty Ltd.

C	-1.9118928462	-0.167897569	0.614329288
C	-2.914271718	1.5495641203	2.6290157051
H	-0.8182910611	2.2373195004	2.7598056569
C	-3.2879006647	-0.2554857259	1.0463322208
H	-1.5283868735	-0.7666877943	-0.1983307319
C	-3.7440335084	0.6092763281	2.0221266735
H	-3.2917010375	2.206442427	3.3972649383
H	-3.9569129107	-0.96850192	0.5880852904
H	-4.782744313	0.5535544395	2.3258244462

MeSO2NH2Ph radical

E(UPW6B95D3) = -877.260419517

Charge = 0 Multiplicity = 2

S	1.2571284745	1.9083544959	0.1291602938
O	1.1830236344	2.7572816023	1.3091367944
O	0.2351722502	2.0408024867	-0.8984266819
C	2.8363492859	2.2470327914	-0.6599561343
H	3.6167476963	2.0938561916	0.0761108337
H	2.9410697288	1.569513617	-1.4992915149
H	2.8019278774	3.2819434865	-0.9900799503
N	0.0705433929	-0.4633301788	1.4576752235
H	0.1906104483	-1.2518609268	0.8462707
C	-1.217763773	0.0052930013	1.609676912
C	-1.5570945437	0.8030603073	2.7022140823
C	-2.1851513715	-0.2518009637	0.6383698401

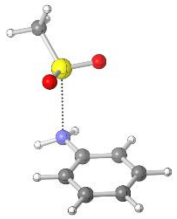

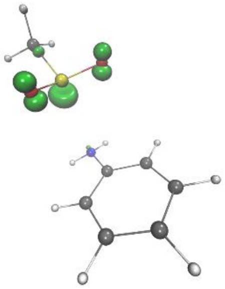

Evaluation Only. Created with Aspose.Pdf. Copyright 2002-2014 Aspose Pty Ltd.

C	-2.8328175494	1.3199333371	2.8184253059
H	-0.8115591889	1.0126898606	3.4551150219
C	-3.4576233199	0.2696597106	0.7659768136
H	-1.9270013524	-0.8609996737	-0.2155584338
C	-3.7940486943	1.0596409606	1.8543148945
H	-3.0770536385	1.934068626	3.6721296394
H	-4.1931143897	0.0579849403	0.004511078
H	-4.7886040356	1.4659927388	1.9491135645
H	0.6278280684	-0.516810411	2.2923187176

Evaluation Only. Created with Aspose.Pdf. Copyright 2002-2014 Aspose Pty Ltd.

X-Ray Crystallographic Data

4-((3,4-Dimethoxyphenethyl)sulfonyl)morpholine (1c)

CCDC 2042738

Bond precision: C–C = 0.0019 A Wavelength = 1.54184

Cell: a = 26.9877(4) b = 5.7256(1) c = 9.8987(2)

α = 90 β = 90.078(2) γ = 90

Temperature: 100 K

	Calculated	Reported
Volume	1529.55(5)	1529.55(5)
Space group	P 21/c	P 1 21/c 1
Hall group	-P 2ybc	-P 2ybc
Moiety formula	$C_{14}H_{21}NO_5S$	$C_{14}H_{21}NO_5S$
Sum formula	$C_{14}H_{21}NO_5S$	$C_{14}H_{21}NO_5S$
M_r	315.38	315.38
D_x,g cm^{-3}	1.370	1.370
Z	4	4
Mu (mm^{-1})	2.075	2.075
F000	672.0	672.0
F000'	675.38	
h,k,l_{max}	34,7,12	33,7,12
N_{ref}	3203	2942
T_{min},T_{max}	0.748,0.849	0.563,1.000
T_{min}'	0.624	

Correction method = # Reported T Limits: T_{min} = 0.563 T_{max} = 1.000 AbsCorr = GAUSSIAN

Data completeness = 0.919 Theta(max) = 76.280

R(reflections) = 0.0326(2713) wR2(reflections) = 0.0863(2942)

S = 1.051 N_{par} = 192

Evaluation Only. Created with Aspose.Pdf. Copyright 2002-2014 Aspose Pty Ltd.

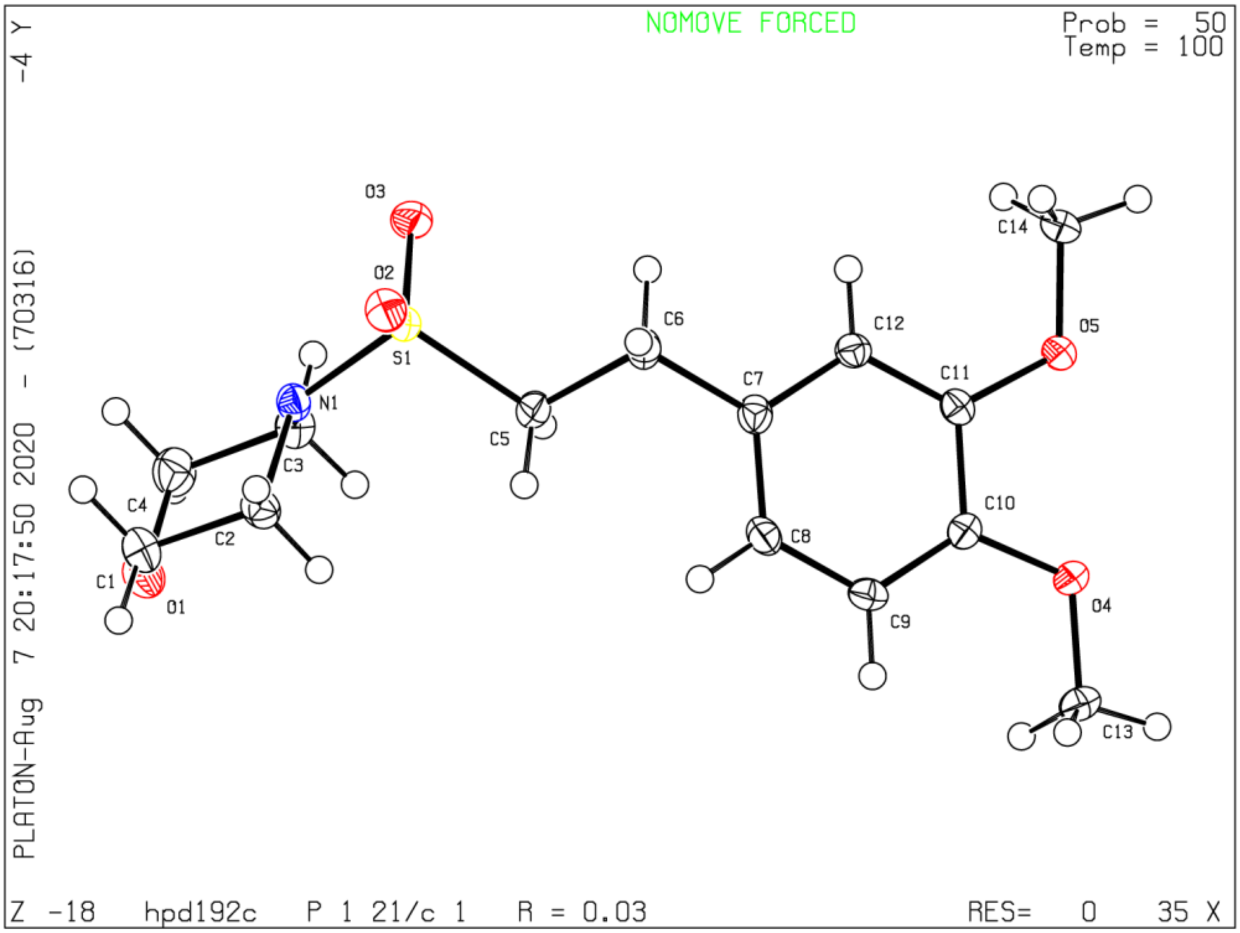

Evaluation Only. Created with Aspose.Pdf. Copyright 2002-2014 Aspose Pty Ltd.

4-((2,3-Dihydro-1*H*-inden-2-yl)sulfonyl)morpholine (1i)

CCDC 2042739

Bond precision: C–C = 0.0021 Å Wavelength = 1.54184

Cell: a = 23.1972(2) b = 6.10055(7) c = 9.24200(9)
α = 90 β = 90.2932(9) γ = 90

Temperature: 100 K

	Calculated	Reported
Volume	1307.87(2)	1307.87(2)
Space group	P 21/c	P 1 21/c 1
Hall group	-P 2ybc	-P 2ybc
Moiety formula	$C_{13}H_{17}NO_3S$	$C_{13}H_{17}NO_3S$
Sum formula	$C_{13}H_{17}NO_3S$	$C_{13}H_{17}NO_3S$
M_r	267.34	267.33
D_x,g cm^{-3}	1.358	1.358
Z	4	4
Mu (mm^{-1})	2.213	2.213
F000	568.0	568.0
F000'	570.92	
h,k,l_{max}	29,7,11	29,7,11
N_{ref}	2738	2668
T_{min},T_{max}	0.658,0.885	0.515,1.000
T_{min}'	0.576	

Correction method = # Reported T Limits: T_{min} = 0.515 T_{max} = 1.000
AbsCorr = GAUSSIAN

Data completeness = 0.974 Theta(max) = 76.443

R(reflections) = 0.0343(2450) wR2(reflections) = 0.0974(2668)

S = 1.090 N_{par} = 163

Evaluation Only. Created with Aspose.Pdf. Copyright 2002-2014 Aspose Pty Ltd.

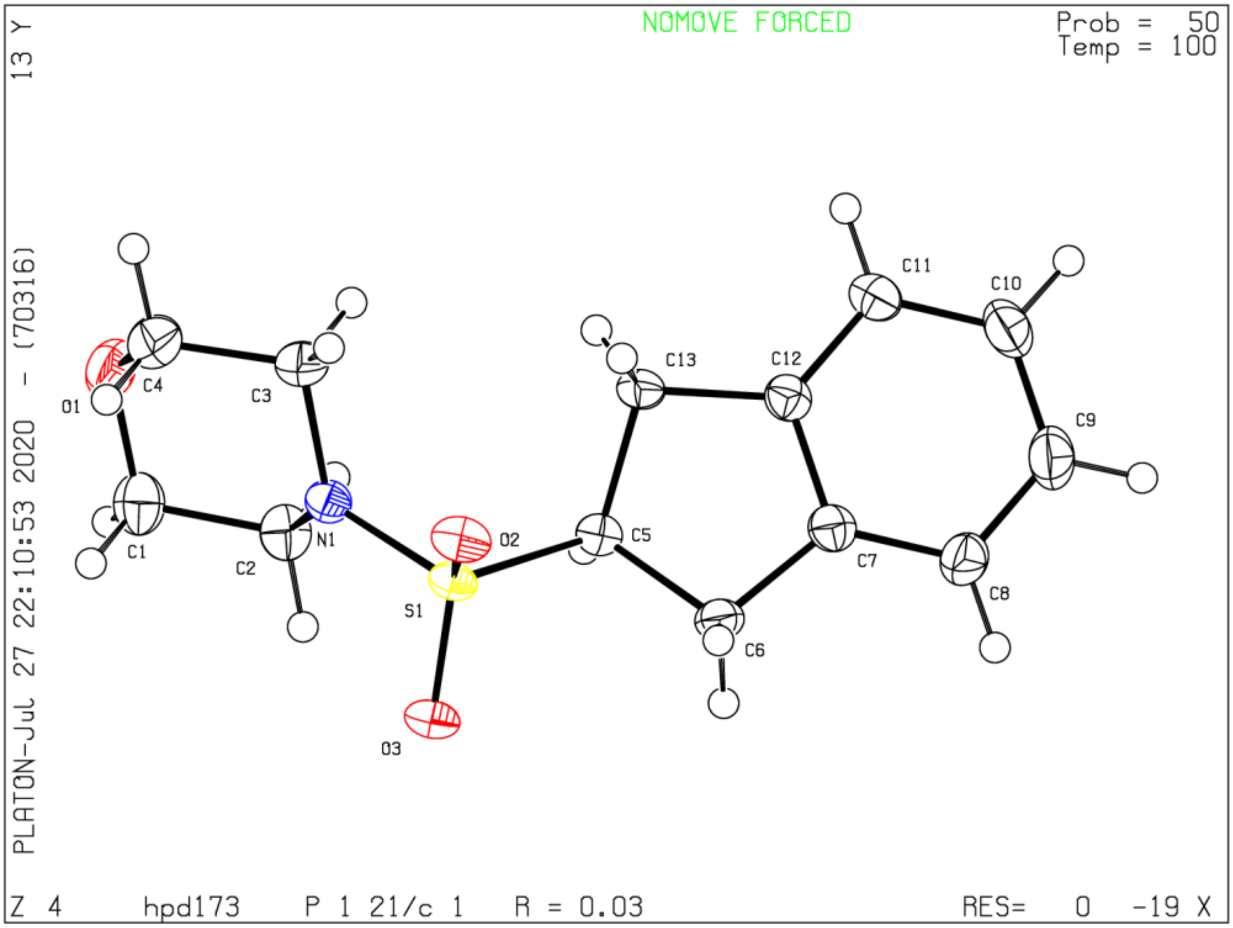

Evaluation Only. Created with Aspose.Pdf. Copyright 2002-2014 Aspose Pty Ltd.

4-((4,4-Difluorocyclohexyl)sulfonyl)morpholine (1j)

CCDC 2042740

Bond precision: C–C = 0.0020 Å Wavelength = 1.54184

Cell:	a = 11.83783(11)	b = 10.04452(10)	c = 10.01996(9)
	α = 90	β = 96.4304(8)	γ = 90

Temperature: 100 K

	Calculated	Reported
Volume	1183.931(19)	1183.93(2)
Space group	P 21/c	P 1 21/c 1
Hall group	-P 2ybc	-P 2ybc
Moiety formula	$C_{10}H_{17}F_2NO_3S$	$C_{10}H_{17}F_2NO_3S$
Sum formula	$C_{10}H_{17}F_2NO_3S$	$C_{10}H_{17}F_2NO_3S$
M_r	269.31	269.30
D_x,g cm^{-3}	1.511	1.511
Z	4	4
Mu (mm^{-1})	2.690	2.690
F000	568.0	568.0
F000'	571.29	
h,k,l_{max}	14,12,12	14,12,12
N_{ref}	2486	2397
T_{min},T_{max}	0.597,0.701	0.534,1.000
T_{min}'	0.497	

Correction method = # Reported T Limits: T_{min} = 0.534 T_{max} = 1.000 AbsCorr = GAUSSIAN

Data completeness = 0.964 Theta(max) = 76.457

R(reflections) = 0.0298(2287) wR2(reflections) = 0.0759(2397)

S = 1.075 N_{par} = 154

Go back to table of contents

Evaluation Only. Created with Aspose.Pdf. Copyright 2002-2014 Aspose Pty Ltd.

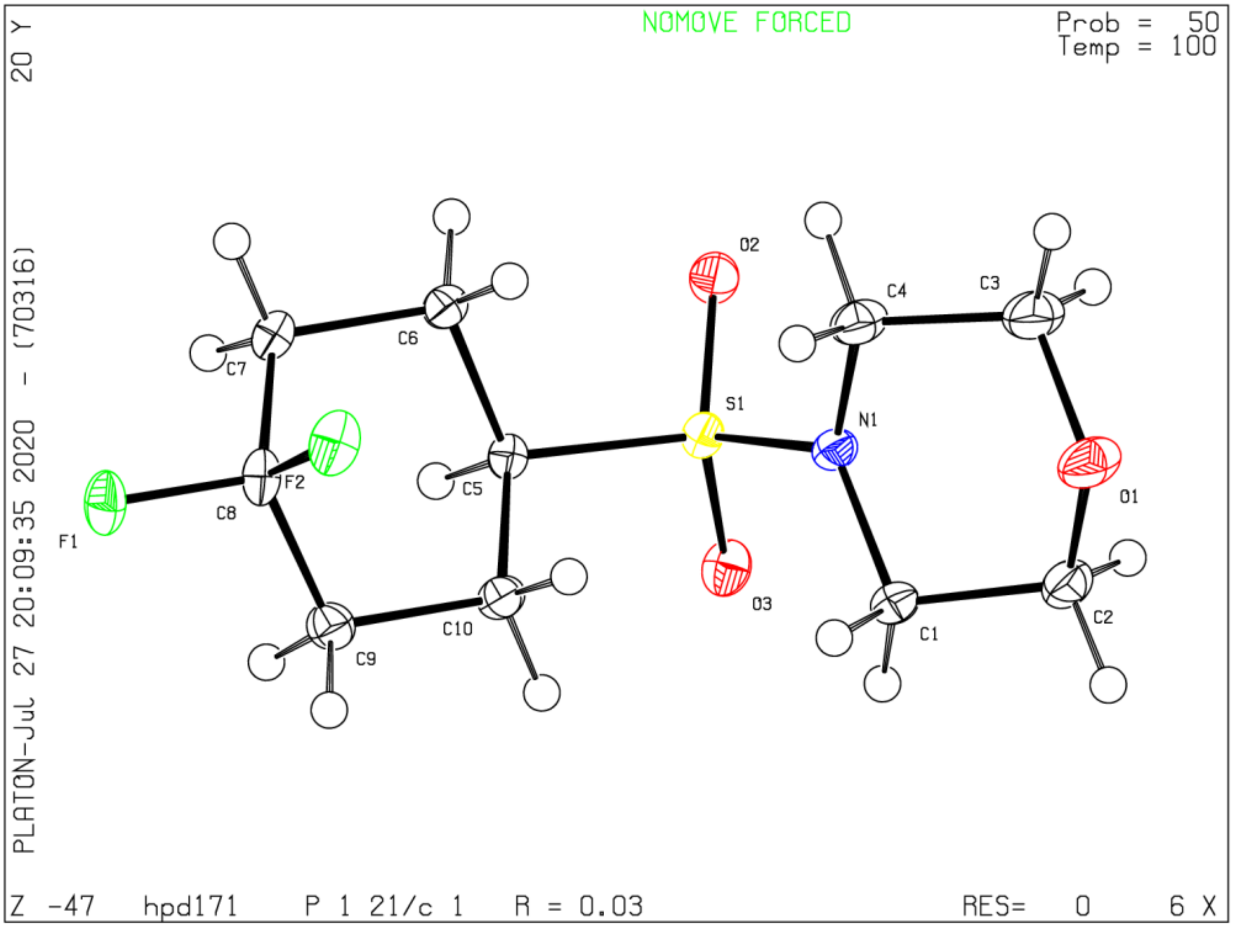

Go back to table of contents

Evaluation Only. Created with Aspose.Pdf. Copyright 2002-2014 Aspose Pty Ltd.

4-((Tetrahydro-2*H*-pyran-4-yl)sulfonyl)morpholine (1l)

CCDC 2042741

Bond precision: C–C = 0.0023 Å Wavelength = 1.54184

Cell: a = 18.4344(2) b = 6.1010(1) c = 9.7772(1)

α = 90 β = 91.605(1) γ = 90

Temperature: 100 K

	Calculated	Reported
Volume	1099.19(2)	1099.19(2)
Space group	P 21/c	P 1 21/c 1
Hall group	-P 2ybc	-P 2ybc
Moiety formula	$C_9H_{17}NO_4S$	$C_9H_{17}NO_4S$
Sum formula	$C_9H_{17}NO_4S$	$C_9H_{17}NO_4S$
M_r	235.30	235.29
D_x,g cm^{-3}	1.422	1.422
Z	4	4
Mu (mm^{-1})	2.613	2.613
F000	504.0	504.0
F000'	506.84	
h,k,l_{max}	23,7,12	22,7,12
N_{ref}	2315	2219
T_{min},T_{max}	0.532,0.760	0.397,1.000
T_{min}'	0.434	

Correction method = # Reported T Limits: T_{min} = 0.397 T_{max} = 1.000
AbsCorr = GAUSSIAN

Data completeness = 0.959 Theta(max) = 76.521

R(reflections) = 0.0369(2062) wR2(reflections) = 0.1034(2219)

S = 1.094 N_{par} = 163

Evaluation Only. Created with Aspose.Pdf. Copyright 2002-2014 Aspose Pty Ltd.

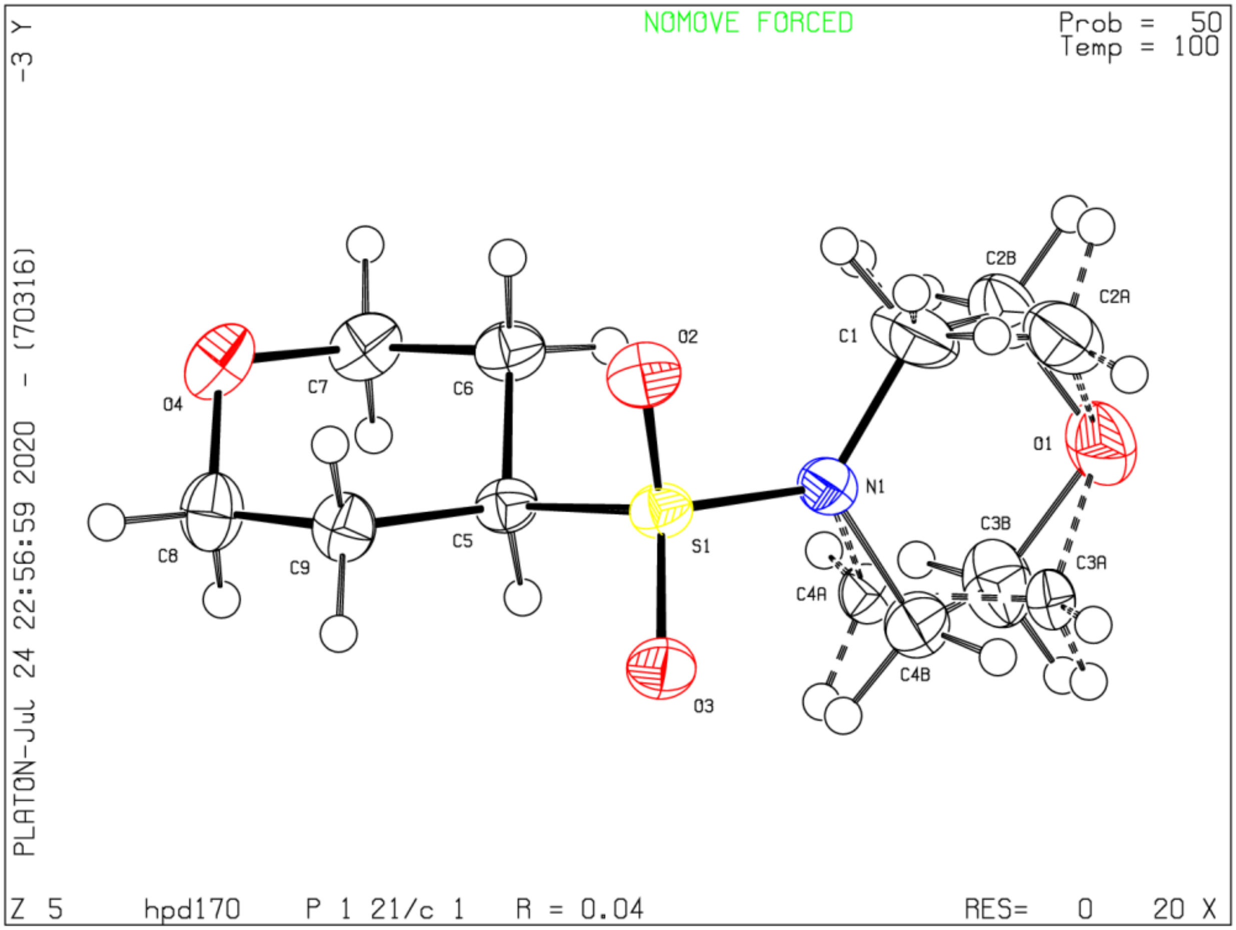

Go back to table of contents

Evaluation Only. Created with Aspose.Pdf. Copyright 2002-2014 Aspose Pty Ltd.

(1*S*,2*S*,4*aR*,4*bR*,7*S*,9*aS*,10*S*,10*aR*)-1-Methyl-8-methylene-10-(morpholinosulfonyl)-13-oxo-1,2,5,6,8,9,10,10a-octahydro-4a,1-(epoxymethano)-7,9a-methanobenzo[*a*]azulene-2,7(4*bH*)-diyl diacetate (10c)

CCDC 2042742

Bond precision:	C–C = 0.0042 Å		Wavelength = 1.54184
Cell:	a = 22.5244(2)	b = 10.26708(10)	c = 12.02634(11)
	α = 90	β = 96.6693(9)	γ = 90
Temperature:	100 K		

	Calculated	Reported
Volume	2762.39(4)	2762.38(5)
Space group	C 2	C 1 2 1
Hall group	C 2y	C 2y
Moiety formula	$C_{26}H_{33}NO_9S$ [+ solvent]	$C_{26}H_{33}NO_9S$
Sum formula	$C_{26}H_{33}NO_9S$ [+ solvent]	$C_{26}H_{33}NO_9S$
M_r	535.59	535.59
D_x,g cm^{-3}	1.288	1.288
Z	4	4
Mu (mm^{-1})	1.482	1.482
F000	1136.0	1136.0
F000'	1140.99	
h,k,l_{max}	28,12,15	27,12,15
N_{ref}	5797[3066]	4895
T_{min},T_{max}	0.771,0.927	0.506,1.000
T_{min}'	0.682	

Correction method = # Reported T Limits: T_{min} = 0.506 T_{max} = 1.000
AbsCorr = GAUSSIAN

Data completeness = 1.60/0.84 Theta(max) = 76.582

R(reflections) = 0.0355(4809) wR2(reflections) = 0.0944(4895)

S = 1.012 N_{par} = 339

Evaluation Only. Created with Aspose.Pdf. Copyright 2002-2014 Aspose Pty Ltd.

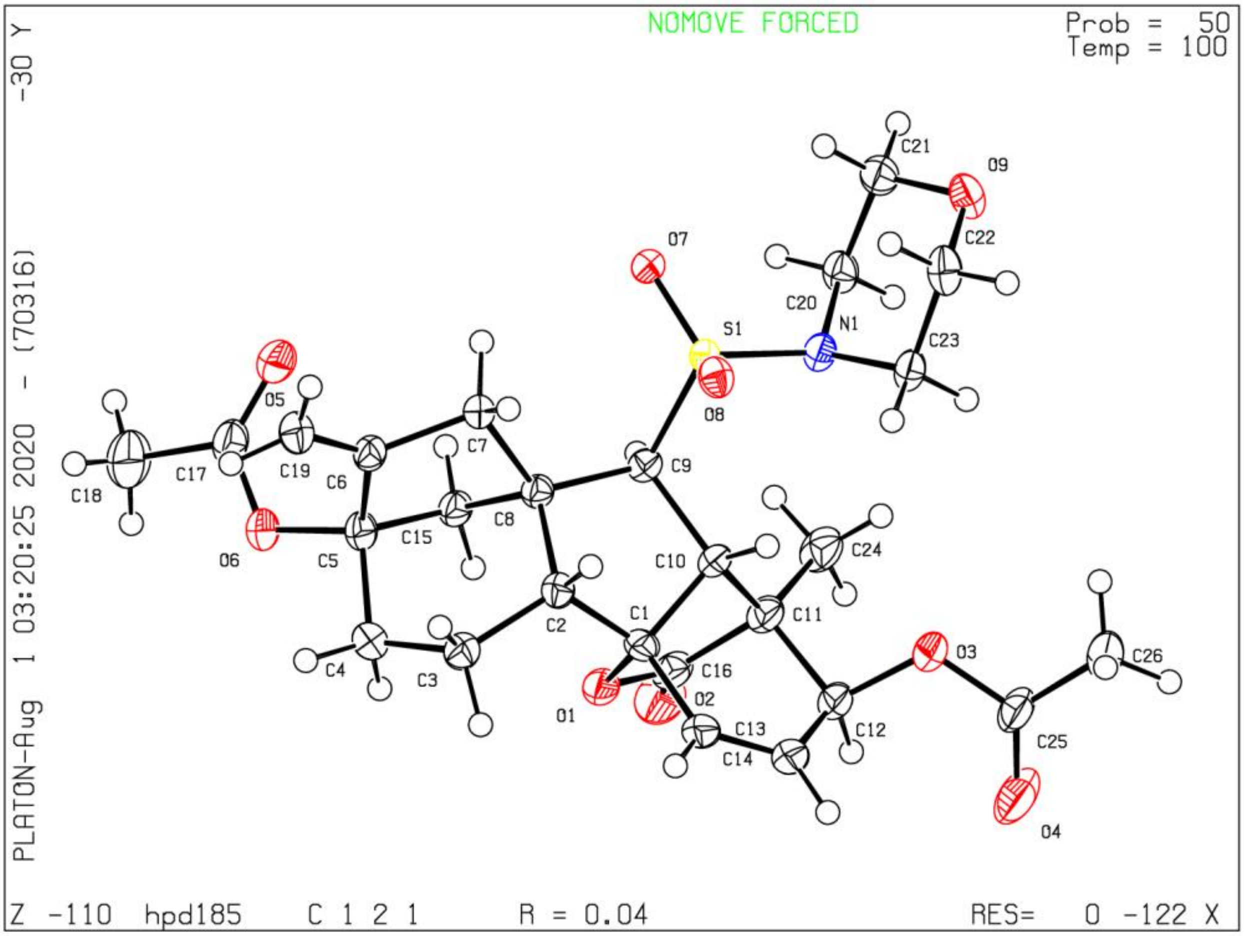

Go back to table of contents

Evaluation Only. Created with Aspose.Pdf. Copyright 2002-2014 Aspose Pty Ltd.

N-(*tert*-Butyl)adamantane-1-sulfonamide (3e)

CCDC 2042743

Bond precision: C–C = 0.0018 A Wavelength = 1.54184

Cell: a = 6.4731(1) b = 19.8101(2) c = 11.0428(1)

α = 90 β = 94.993(1) γ = 90

Temperature: 100 K

	Calculated	Reported
Volume	1410.68(3)	1410.68(3)
Space group	P 21/n	P 1 21/n 1
Hall group	-P 2yn	-P 2yn
Moiety formula	$C_{14}H_{25}NO_2S$	$C_{14}H_{25}NO_2S$
Sum formula	$C_{14}H_{25}NO_2S$	$C_{14}H_{25}NO_2S$
M_r	271.41	271.41
D_x,g cm^{-3}	1.278	1.278
Z	4	4
Mu (mm^{-1})	1.992	1.992
F000	592.0	592.0
F000'	594.80	
h,k,l_{max}	8,24,13	8,24,13
N_{ref}	2956	2875
T_{min},T_{max}	0.776,0.896	0.676,1.000
T_{min}'	0.596	

Correction method = # Reported T Limits: T_{min} = 0.676 T_{max} = 1.000
AbsCorr = GAUSSIAN

Data completeness = 0.973 Theta(max) = 76.467

R(reflections) = 0.0321(2706) wR2(reflections) = 0.0871(2875)

S = 1.048 N_{par} = 169

Evaluation Only. Created with Aspose.Pdf. Copyright 2002-2014 Aspose Pty Ltd.

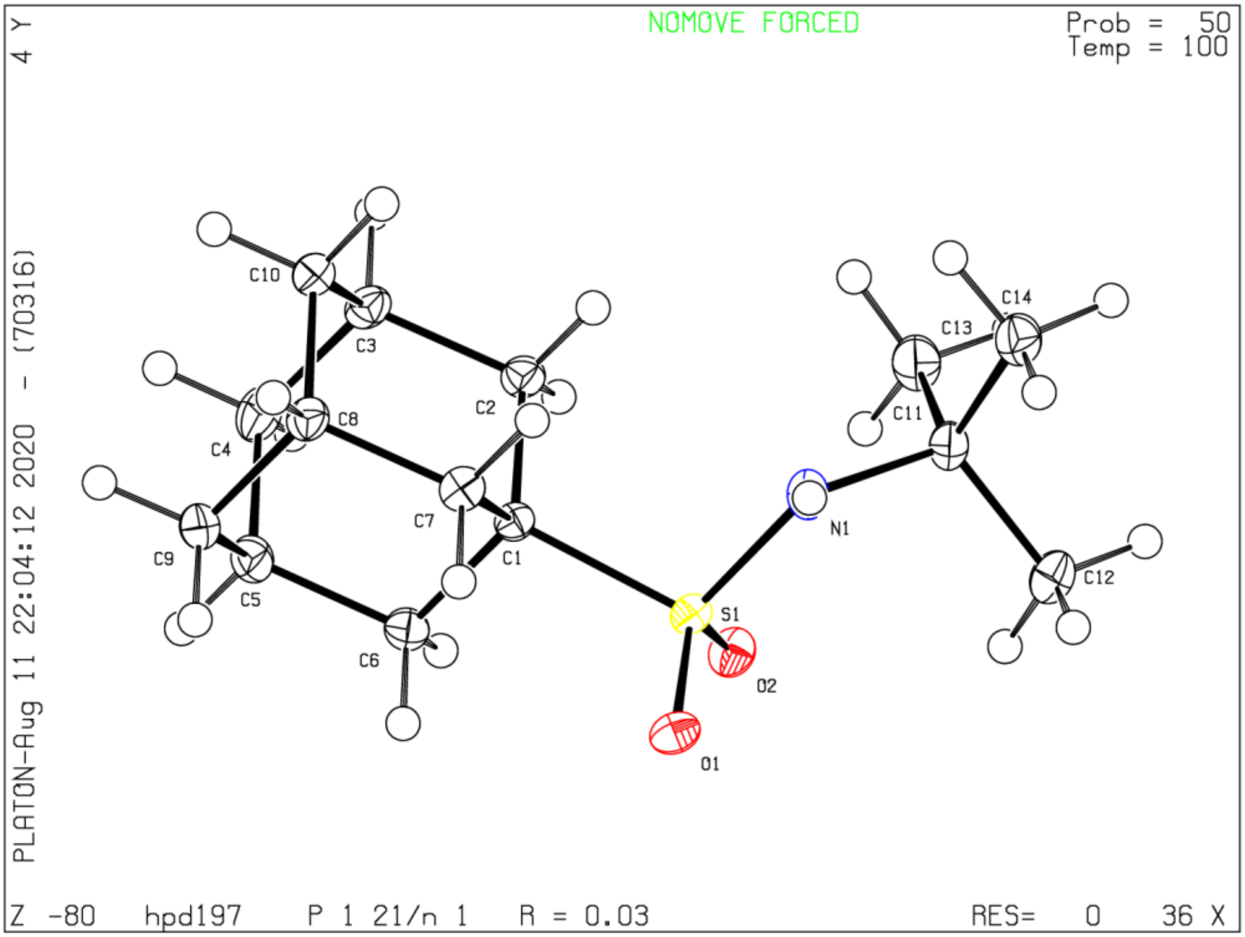

Evaluation Only. Created with Aspose.Pdf. Copyright 2002-2014 Aspose Pty Ltd.

N-Benzyltetrahydro-2*H*-pyran-4-sulfonamide (4b)

CCDC 2042744

Bond precision: C–C = 0.0035 Å Wavelength = 1.54184

Cell: a = 5.4965(2) b = 24.4849(12) c = 9.7713(4)

α = 90 β = 105.544(4) γ = 90

Temperature: 100 K

	Calculated	Reported
Volume	1266.94(10)	1266.94(10)
Space group	P 21/n	P 1 21/n 1
Hall group	-P 2yn	-P 2yn
Moiety formula	$C_{12}H_{17}NO_3S$	$C_{12}H_{17}NO_3S$
Sum formula	$C_{12}H_{17}NO_3S$	$C_{12}H_{17}NO_3S$
M_r	255.33	255.32
D_x,g cm^{-3}	1.339	1.339
Z	4	4
Mu (mm^{-1})	2.256	2.256
F000	544.0	544.0
F000'	546.85	
h,k,l_{max}	6,30,12	6,30,12
N_{ref}	2666	2554
T_{min},T_{max}	0.709,0.906	0.533,1.000
T_{min}'	0.643	

Correction method = # Reported T Limits: T_{min} = 0.533 T_{max} = 1.000

AbsCorr = GAUSSIAN

Data completeness = 0.958 Theta(max) = 76.662

R(reflections) = 0.0486(2308) wR2(reflections) = 0.1239(2554)

S = 1.084 N_{par} = 157

Evaluation Only. Created with Aspose.Pdf. Copyright 2002-2014 Aspose Pty Ltd.

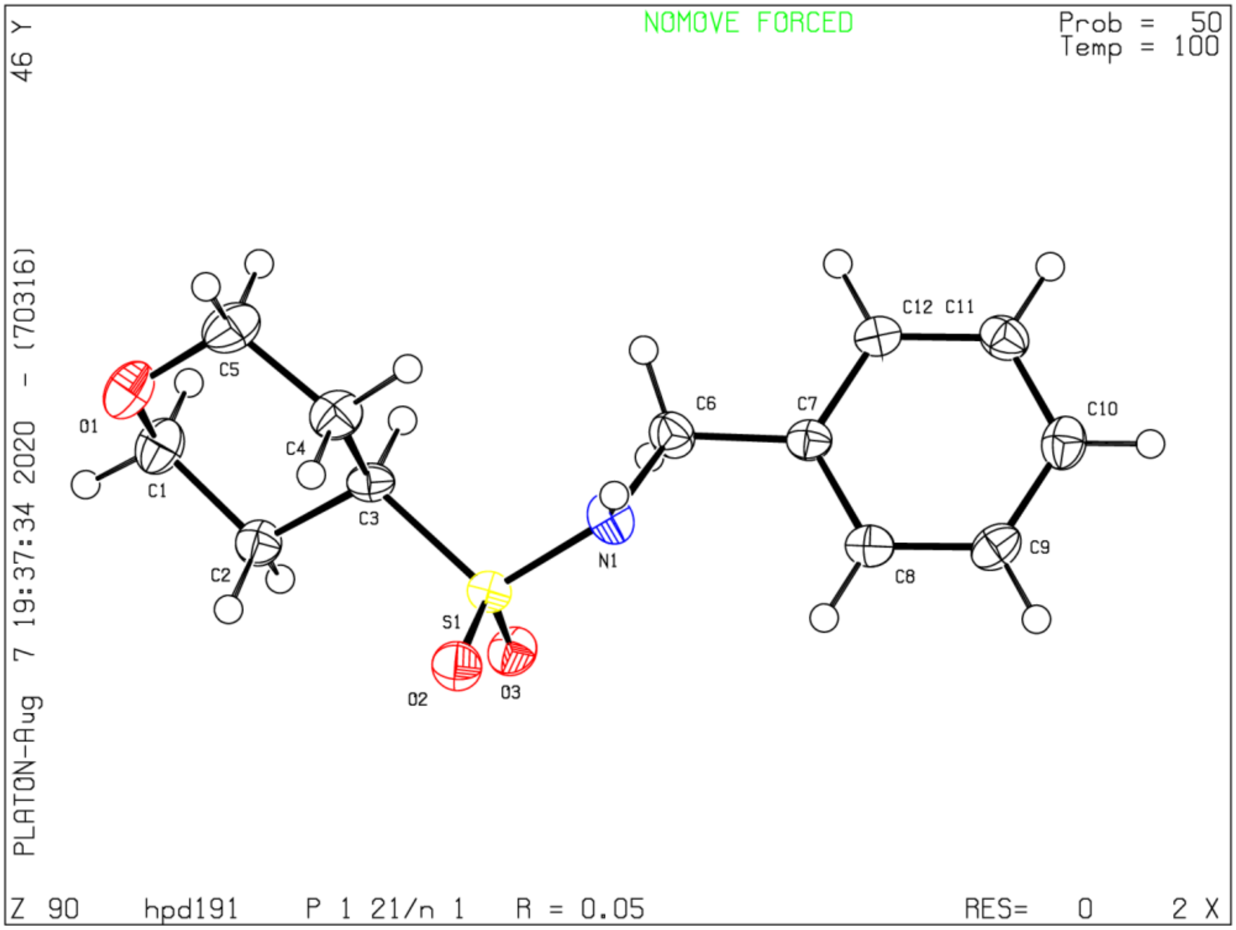

Evaluation Only. Created with Aspose.Pdf. Copyright 2002-2014 Aspose Pty Ltd.

N-Benzyladamantane-1-sulfonamide (4d)

CCDC 2042745

Bond precision: C–C = 0.0033 Å Wavelength = 1.54184

Cell: a = 14.0325(4) b = 6.2686(1) c = 18.2532(5)

α = 90 β = 110.571(3) γ = 90

Temperature: 100 K

	Calculated	Reported
Volume	1503.25(7)	1503.25(7)
Space group	P 21/n	P 1 21/n 1
Hall group	-P 2yn	-P 2yn
Moiety formula	$C_{17}H_{23}NO_2S$	$C_{17}H_{23}NO_2S$
Sum formula	$C_{17}H_{23}NO_2S$	$C_{17}H_{23}NO_2S$
M_r	305.42	305.42
D_x,g cm^{-3}	1.349	1.350
Z	4	4
Mu (mm^{-1})	1.941	1.941
F000	656.0	656.0
F000'	659.00	
h,k,l_{max}	17,7,23	17,7,23
N_{ref}	3159	5689
T_{min},T_{max}	0.760,0.956	0.807,1.000
T_{min}'	0.746	

Correction method = # Reported T Limits: T_{min} = 0.807 T_{max} = 1.000
AbsCorr = GAUSSIAN

Data completeness = 1.801 Theta($_{Max}$) = 76.687

R(reflections) = 0.0445(5110) wR2(reflections) = 0.1096(5689)

S = 1.082 N_{par} = 191

Go back to table of contents

Evaluation Only. Created with Aspose.Pdf. Copyright 2002-2014 Aspose Pty Ltd.

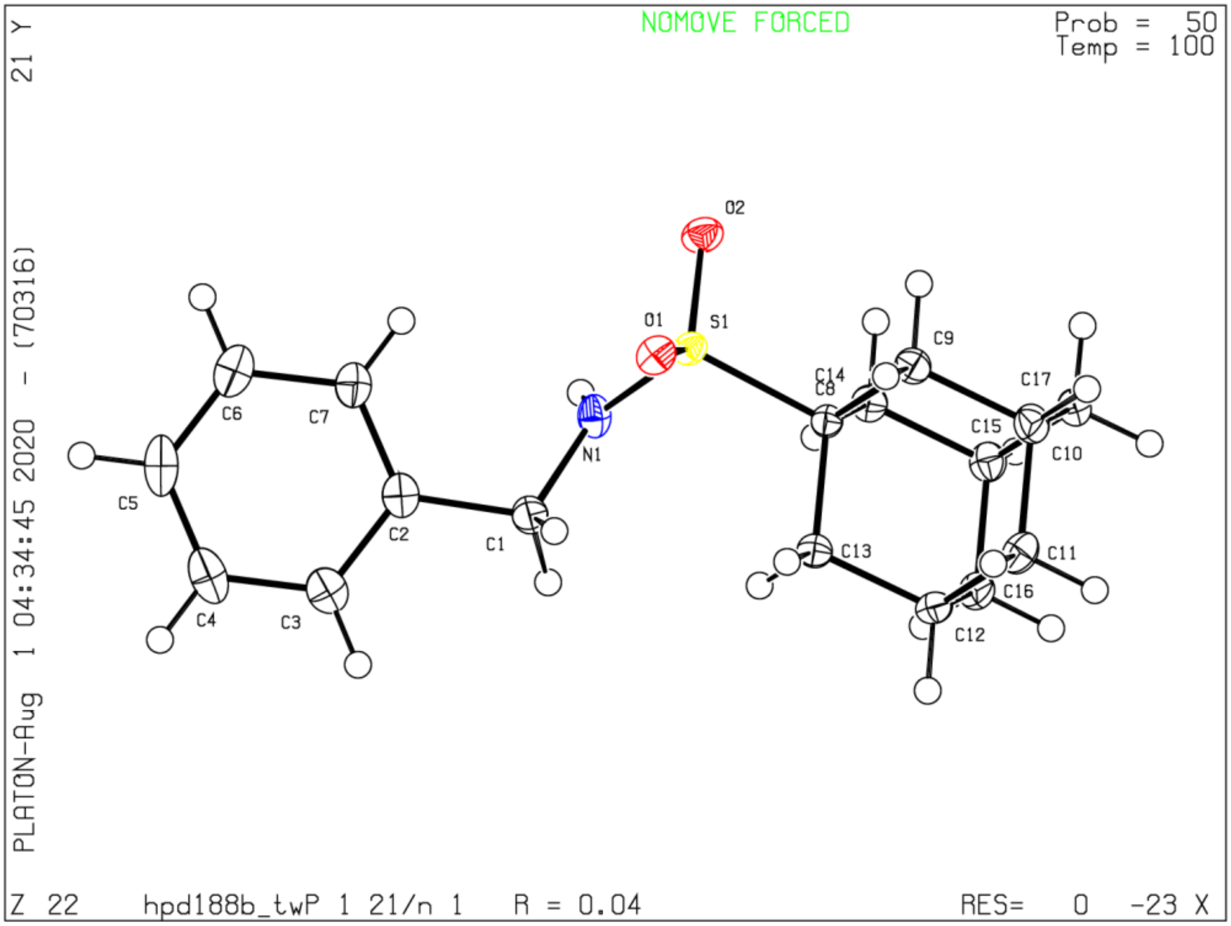

Go back to table of contents

Evaluation Only. Created with Aspose.Pdf. Copyright 2002-2014 Aspose Pty Ltd.

4-(5-Methylthiophen-2-yl)-4-oxobutane-1-sulfonyl azide (9d)

CCDC 2042746

Bond precision: C–C = 0.0024 A Wavelength = 1.54184

Cell: a = 5.4957(1) b = 5.8614(2) c = 18.6487(6)

α = 83.542(3) β = 88.913(2) γ = 82.753(2)

Temperature: 100 K

	Calculated	Reported
Volume	592.13(3)	592.13(3)
Space group	P -1	P -1
Hall group	-P 1	-P 1
Moiety formula	$C_9H_{11}N_3O_3S_2$	$C_9H_{11}N_3O_3S_2$
Sum formula	$C_9H_{11}N_3O_3S_2$	$C_9H_{11}N_3O_3S_2$
M_r	273.33	273.33
D_x,g cm^{-3}	1.533	1.533
Z	2	2
Mu (mm^{-1})	4.116	4.116
F000	284.0	284.0
F000'	286.10	
h,k,l_{max}	6,7,23	6,7,23
N_{ref}	2498	2363
T_{min},T_{max}	0.628,0.913	0.568,1.000
T_{min}'	0.495	

Correction method = # Reported T Limits: T_{min} = 0.568 T_{max} = 1.000

AbsCorr = GAUSSIAN

Data completeness = 0.946 Theta(max) = 76.591

R(reflections) = 0.0375(2181) wR2(reflections) = 0.1002(2363)

S = 1.080 N_{par} = 155

Evaluation Only. Created with Aspose.Pdf. Copyright 2002-2014 Aspose Pty Ltd.

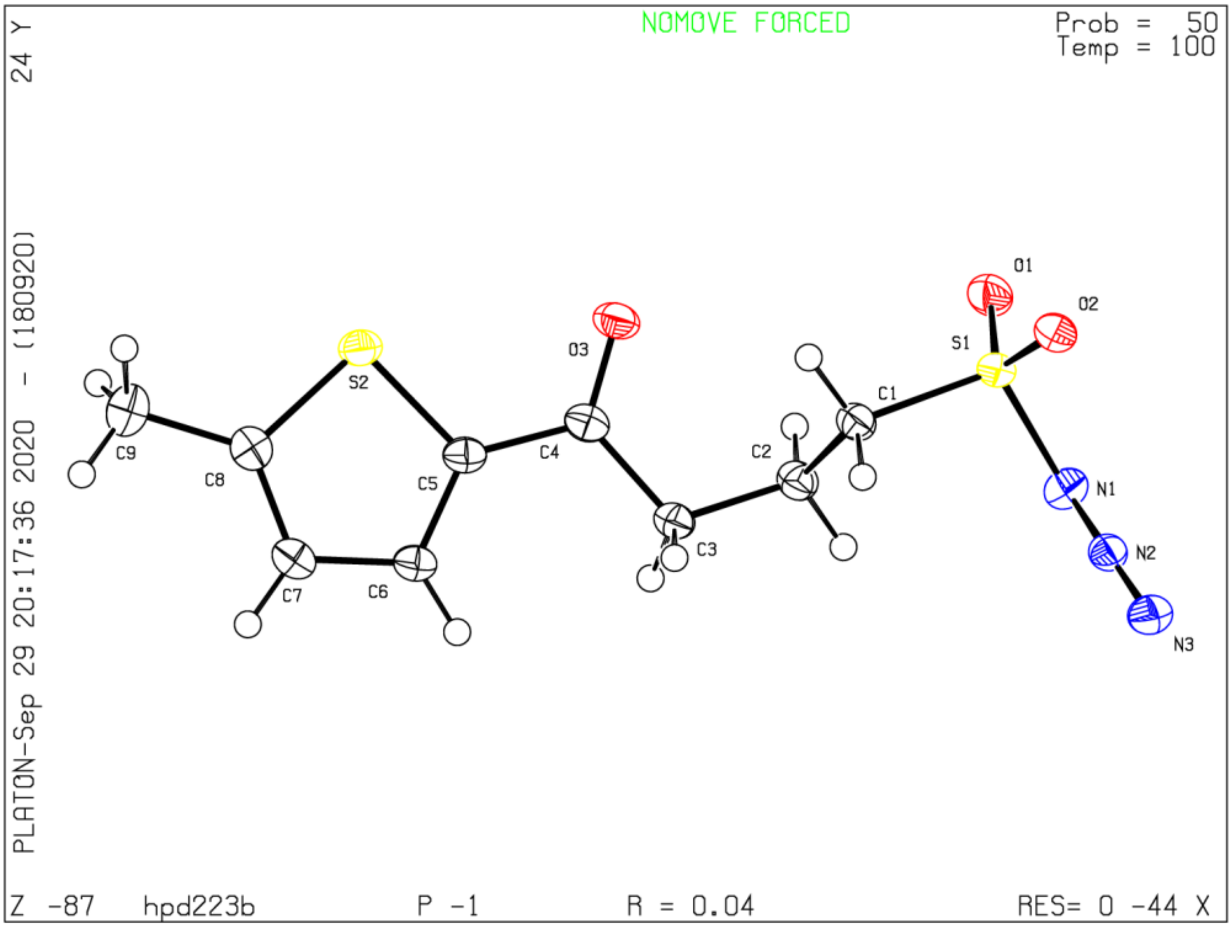

Evaluation Only. Created with Aspose.Pdf. Copyright 2002-2014 Aspose Pty Ltd.

NMR Spectroscopic Data

2,7-Dimethyl-9-(*o*-tolyl)acridine (A3)

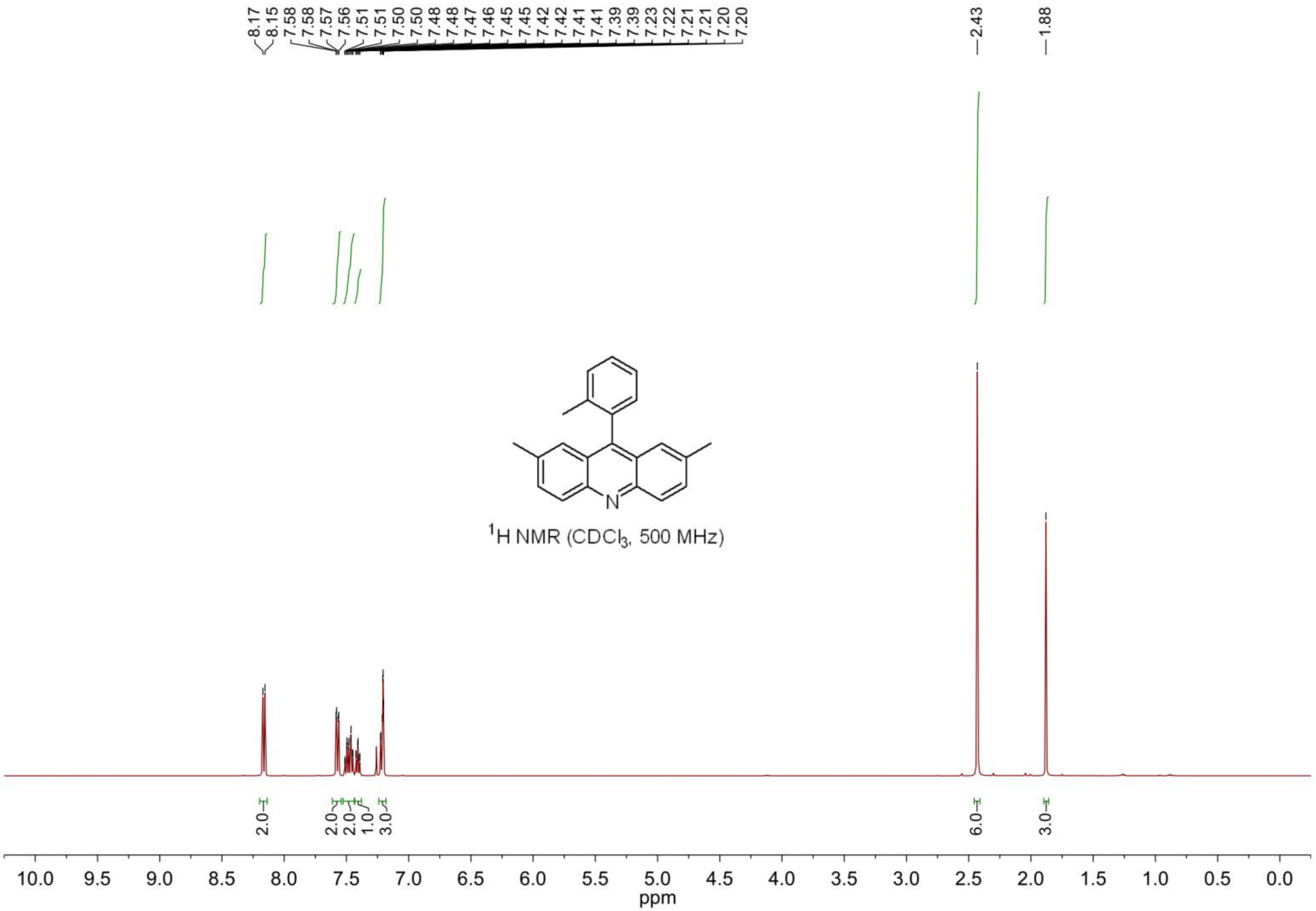

Go back to table of contents

Evaluation Only. Created with Aspose.Pdf. Copyright 2002-2014 Aspose Pty Ltd.

2,7-Dimethyl-9-(*o*-tolyl)acridine (A3)

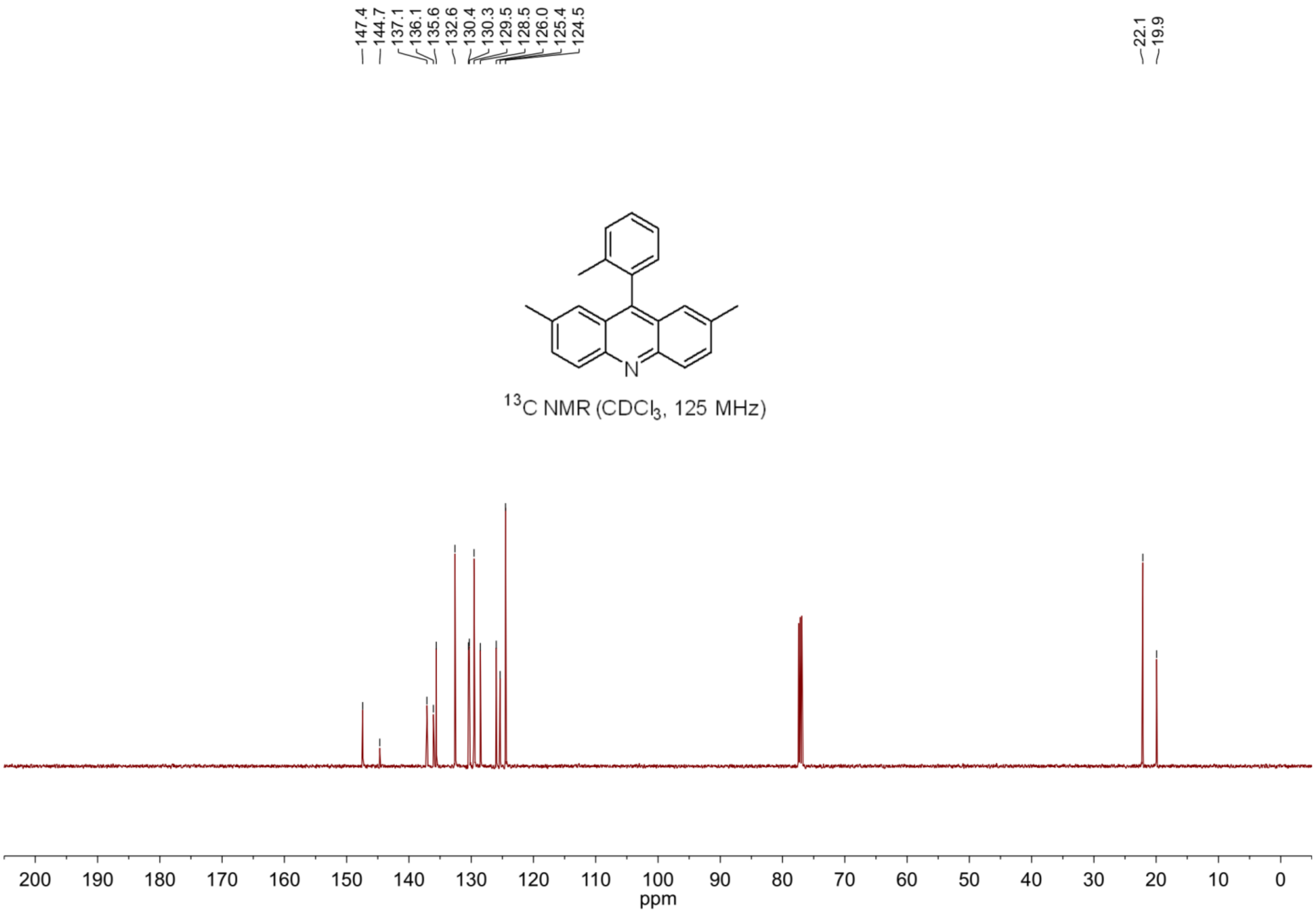

Go back to table of contents

Evaluation Only. Created with Aspose.Pdf. Copyright 2002-2014 Aspose Pty Ltd.

4-(Pentadecylsulfonyl)morpholine (1a)

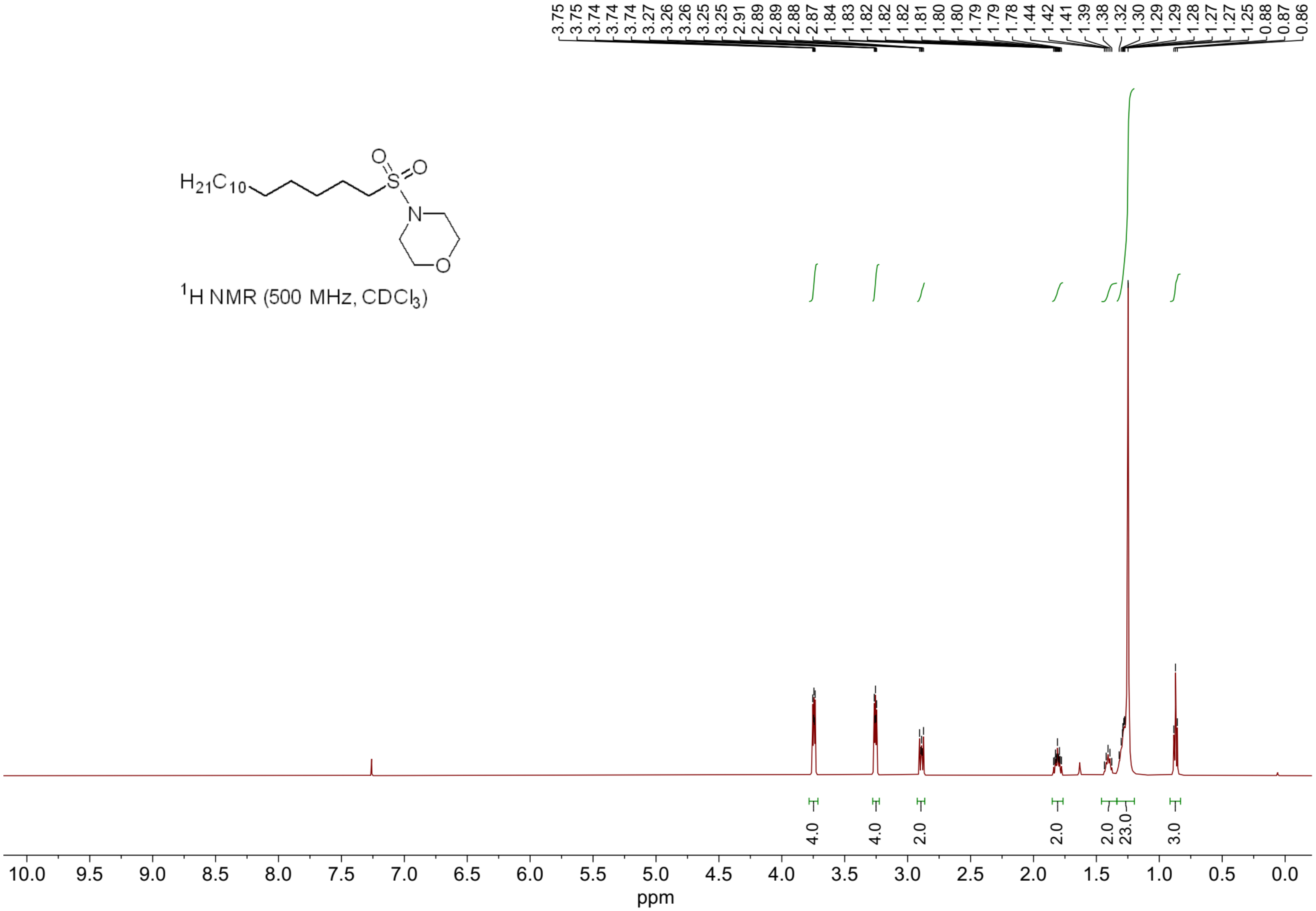

Evaluation Only. Created with Aspose.Pdf. Copyright 2002-2014 Aspose Pty Ltd.

4-(Pentadecylsulfonyl)morpholine (1a)

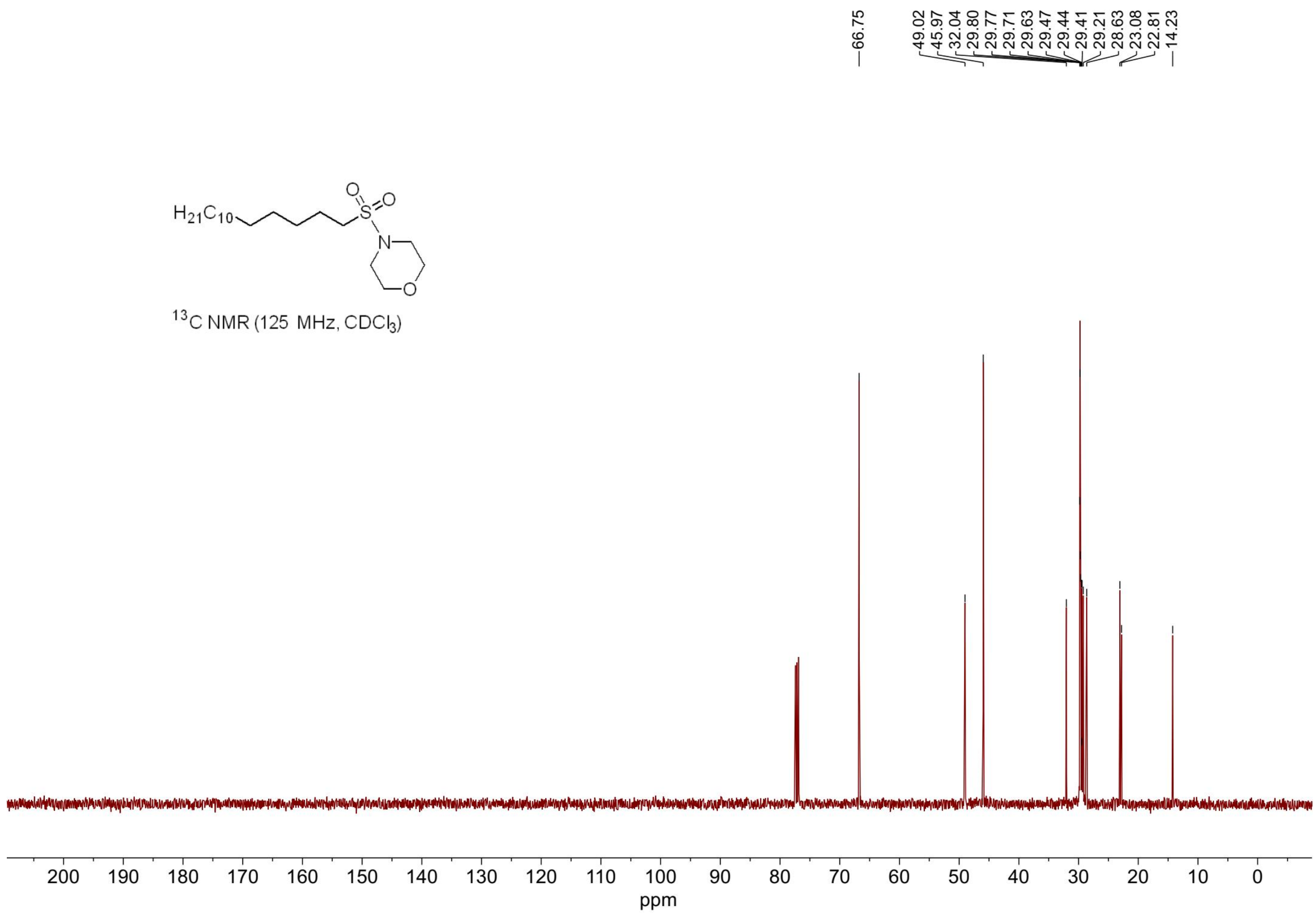

Go back to table of contents

Evaluation Only. Created with Aspose.Pdf. Copyright 2002-2014 Aspose Pty Ltd.

4-(Morpholinosulfonyl)-1-phenylbutan-1-one (1b)

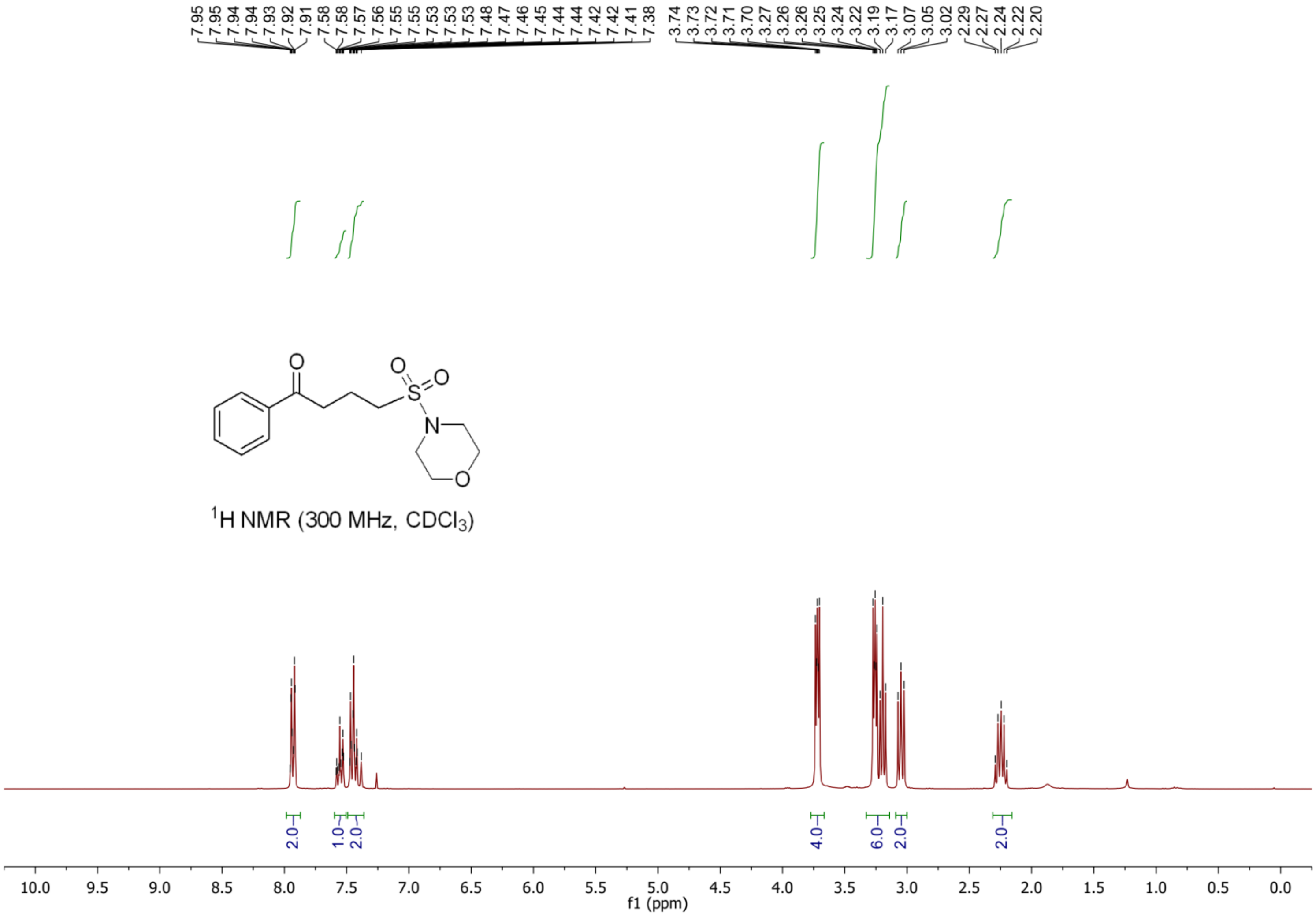

Go back to table of contents

Evaluation Only. Created with Aspose.Pdf. Copyright 2002-2014 Aspose Pty Ltd.

4-(Morpholinosulfonyl)-1-phenylbutan-1-one (1b)

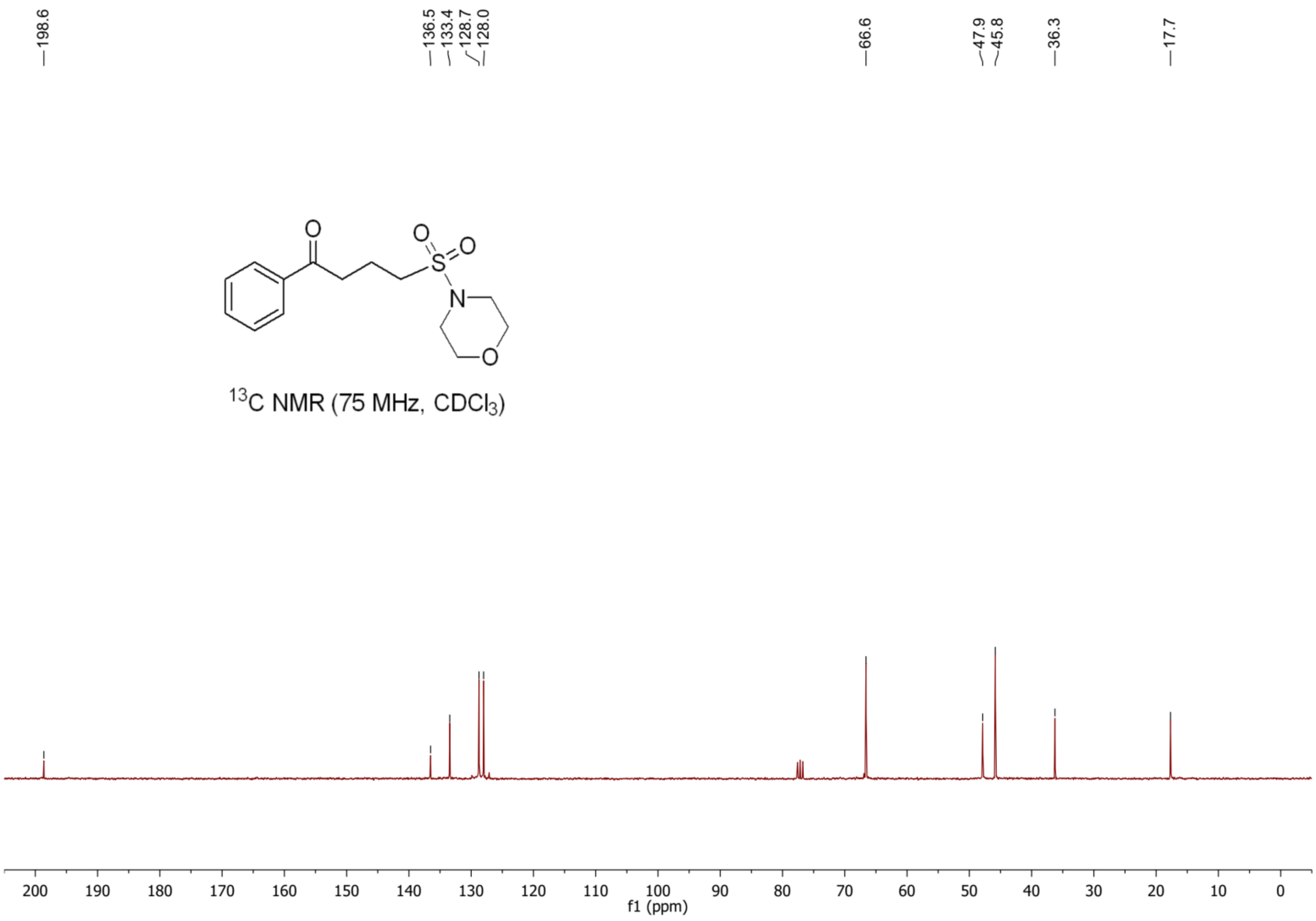

Go back to table of contents

Evaluation Only. Created with Aspose.Pdf. Copyright 2002-2014 Aspose Pty Ltd.

4-((3,4-Dimethoxyphenethyl)sulfonyl)morpholine (1c)

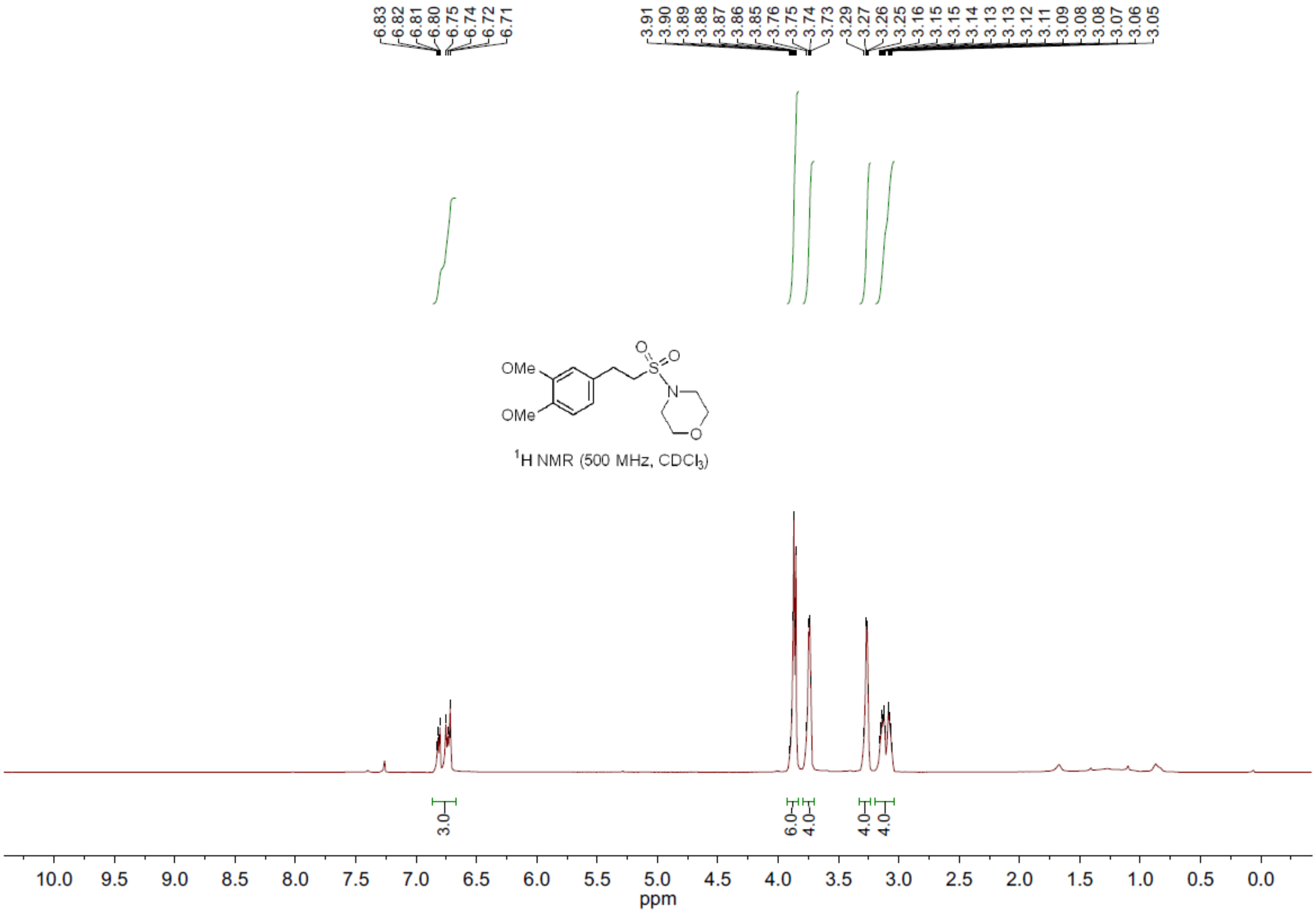

Go back to table of contents

Evaluation Only. Created with Aspose.Pdf. Copyright 2002-2014 Aspose Pty Ltd.

4-((3,4-Dimethoxyphenethyl)sulfonyl)morpholine (1c)

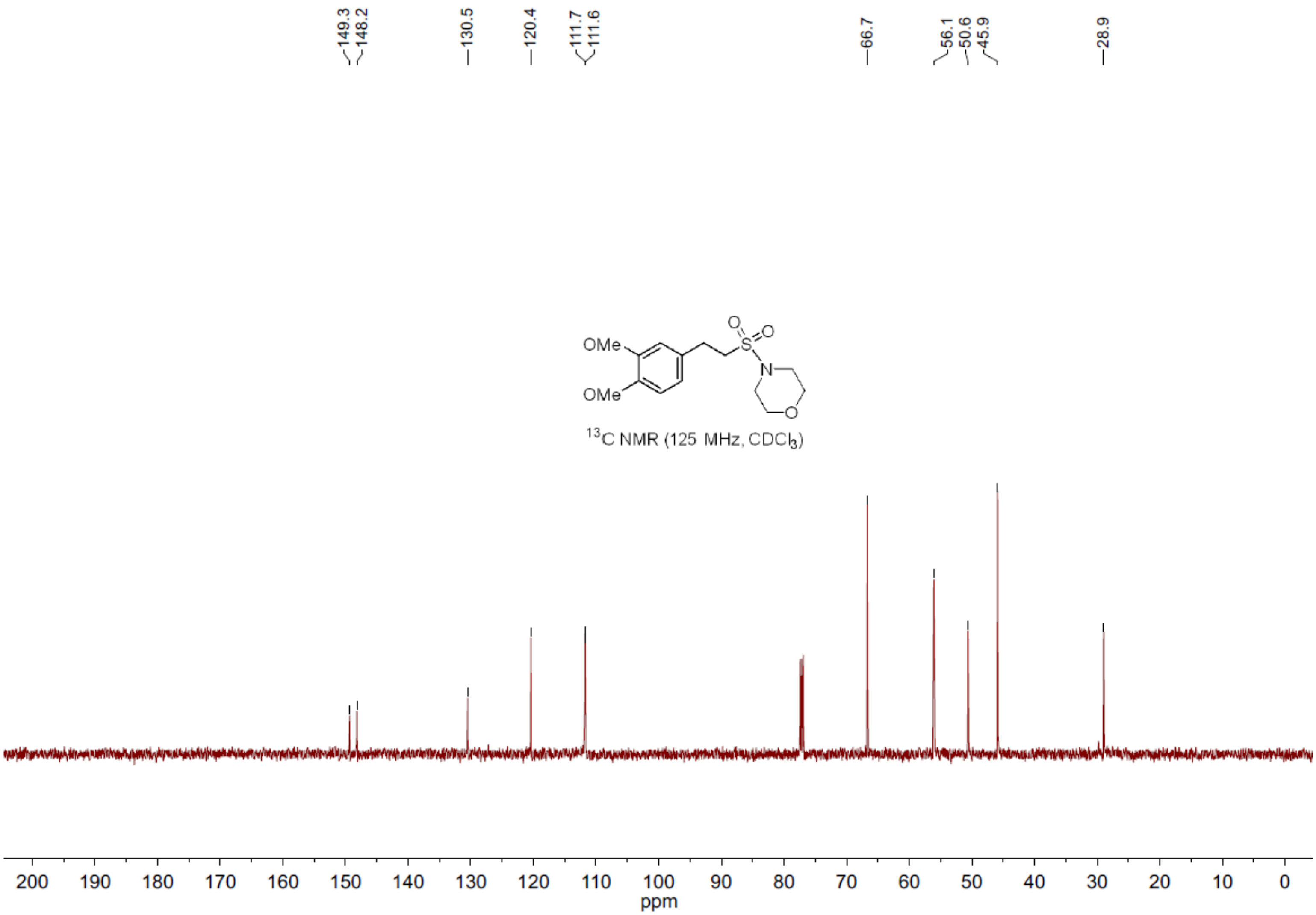

Go back to table of contents

Evaluation Only. Created with Aspose.Pdf. Copyright 2002-2014 Aspose Pty Ltd.

N-(3-(Morpholinosulfonyl)propyl)-3-(4,4,5,5-tetramethyl-1,3,2-dioxaborolan-2-yl)benzamide (1d)

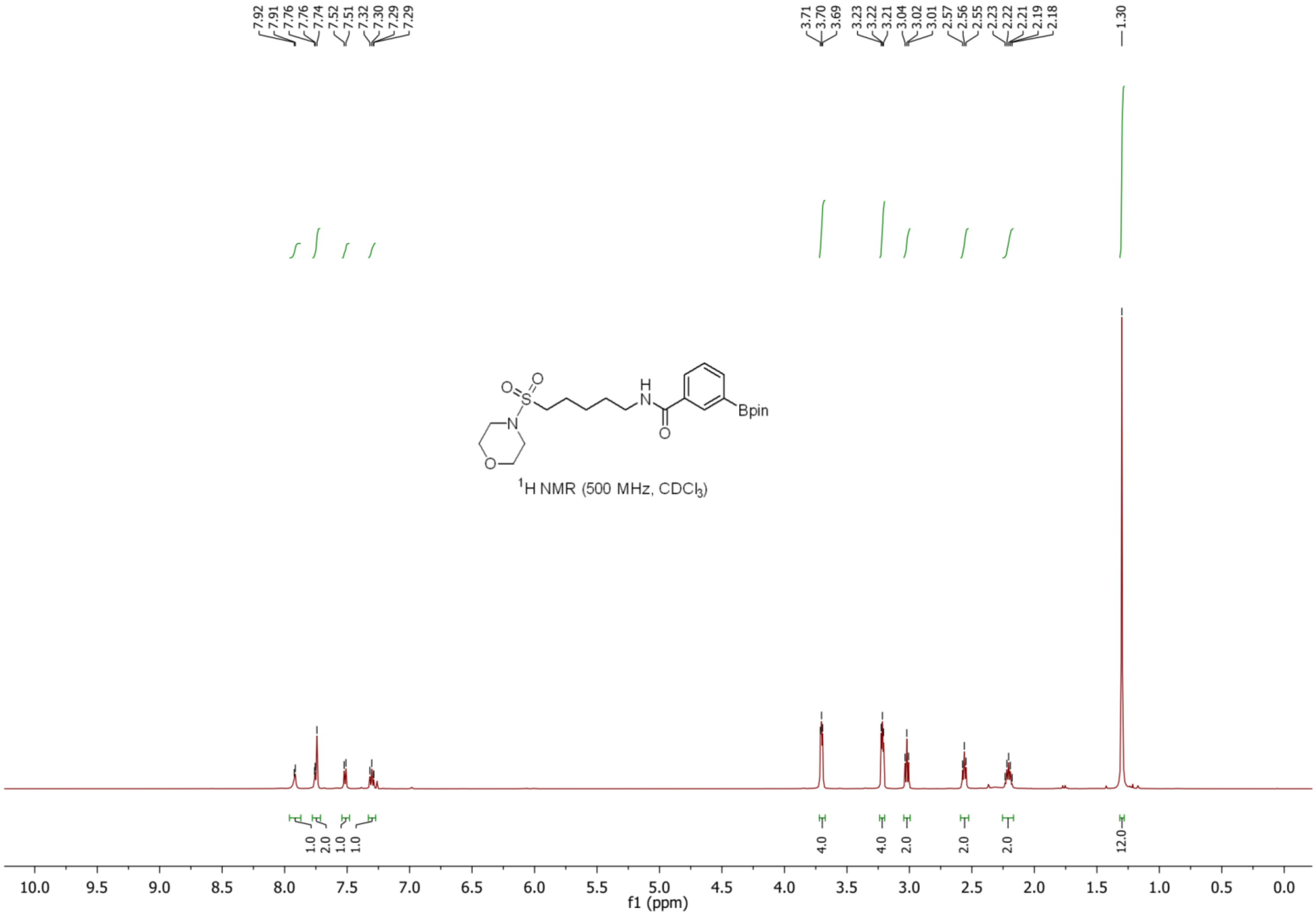

Go back to table of contents

Evaluation Only. Created with Aspose.Pdf. Copyright 2002-2014 Aspose Pty Ltd.

N-(3-(Morpholinosulfonyl)propyl)-3-(4,4,5,5-tetramethyl-1,3,2-dioxaborolan-2-yl)benzamide (1d)

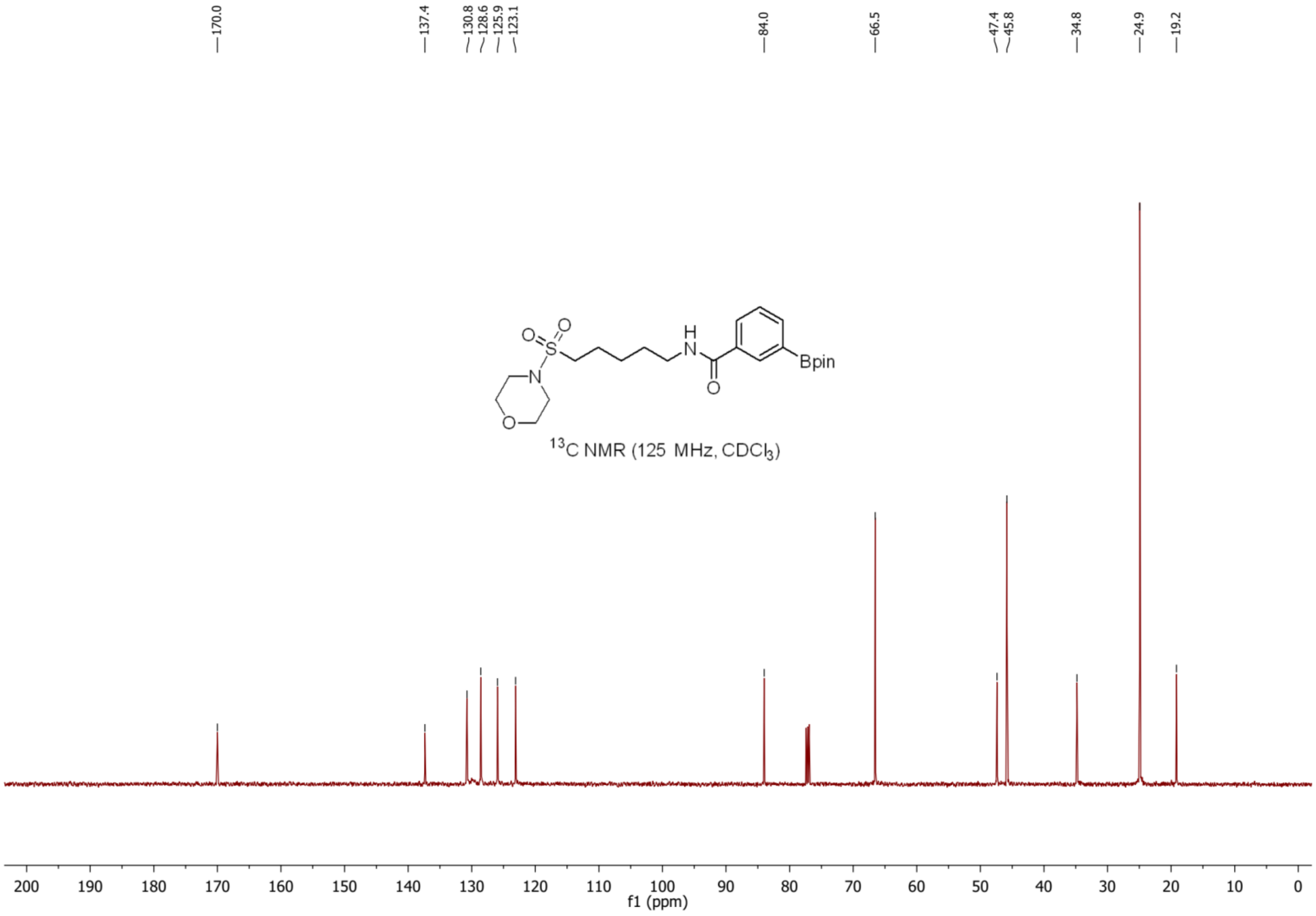

Go back to table of contents

Evaluation Only. Created with Aspose.Pdf. Copyright 2002-2014 Aspose Pty Ltd.

3-(Morpholinosulfonyl)-1-phenylpropan-1-one (1e)

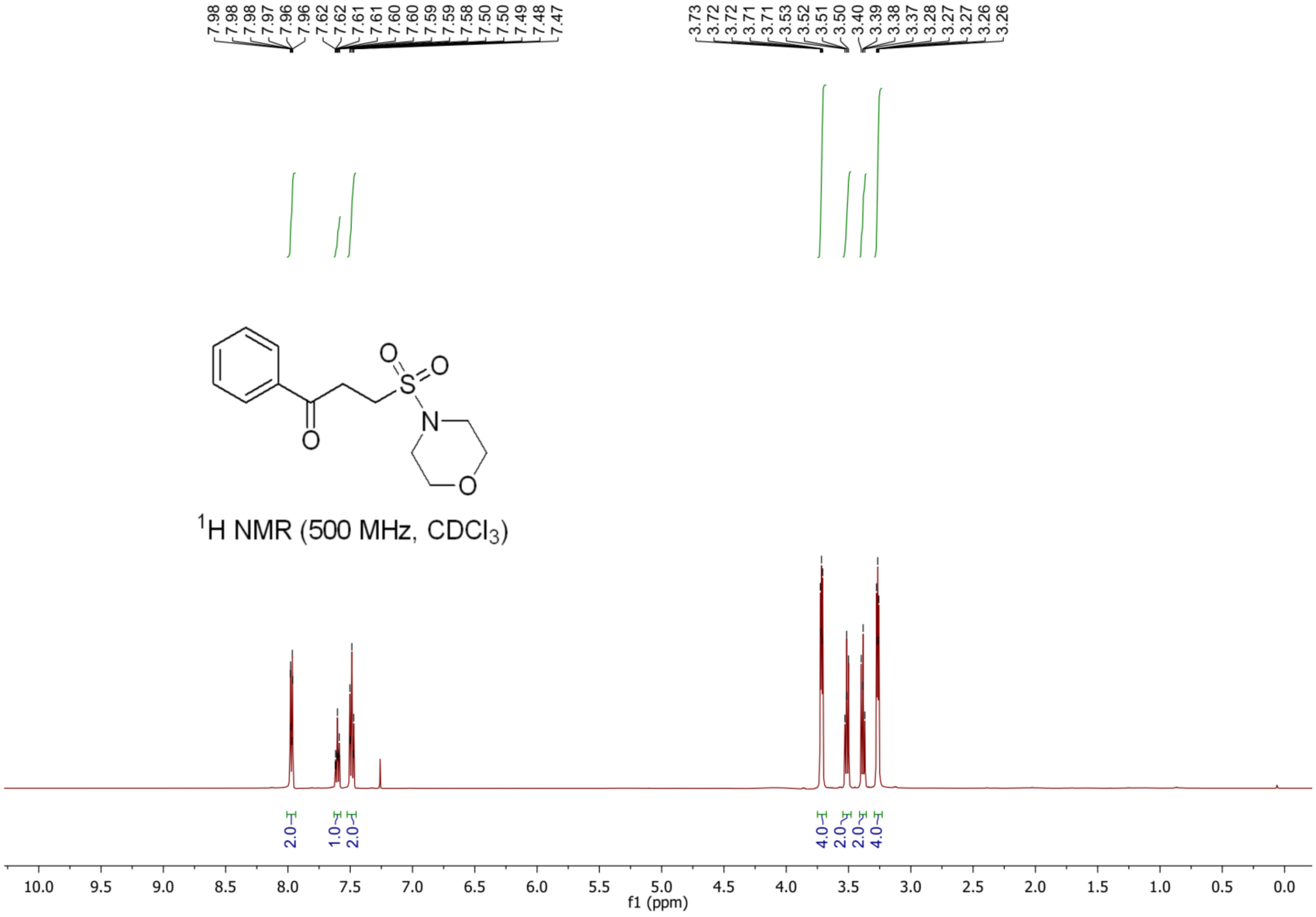

Go back to table of contents

Evaluation Only. Created with Aspose.Pdf. Copyright 2002-2014 Aspose Pty Ltd.

3-(Morpholinosulfonyl)-1-phenylpropan-1-one (1e)

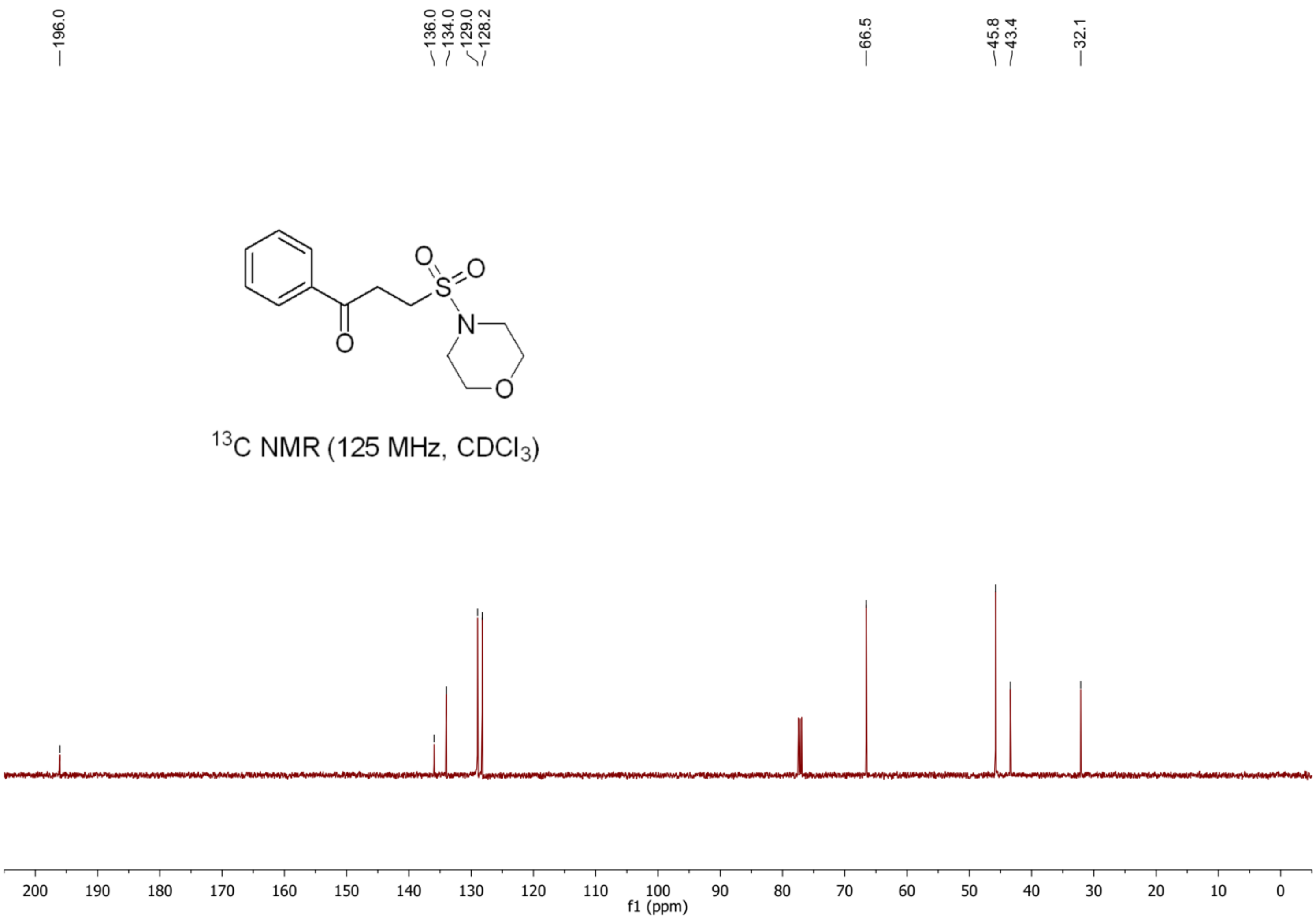

Go back to table of contents

Evaluation Only. Created with Aspose.Pdf. Copyright 2002-2014 Aspose Pty Ltd.

1-(5-Methylthiophen-2-yl)-4-(morpholinosulfonyl)butan-1-one (1f)

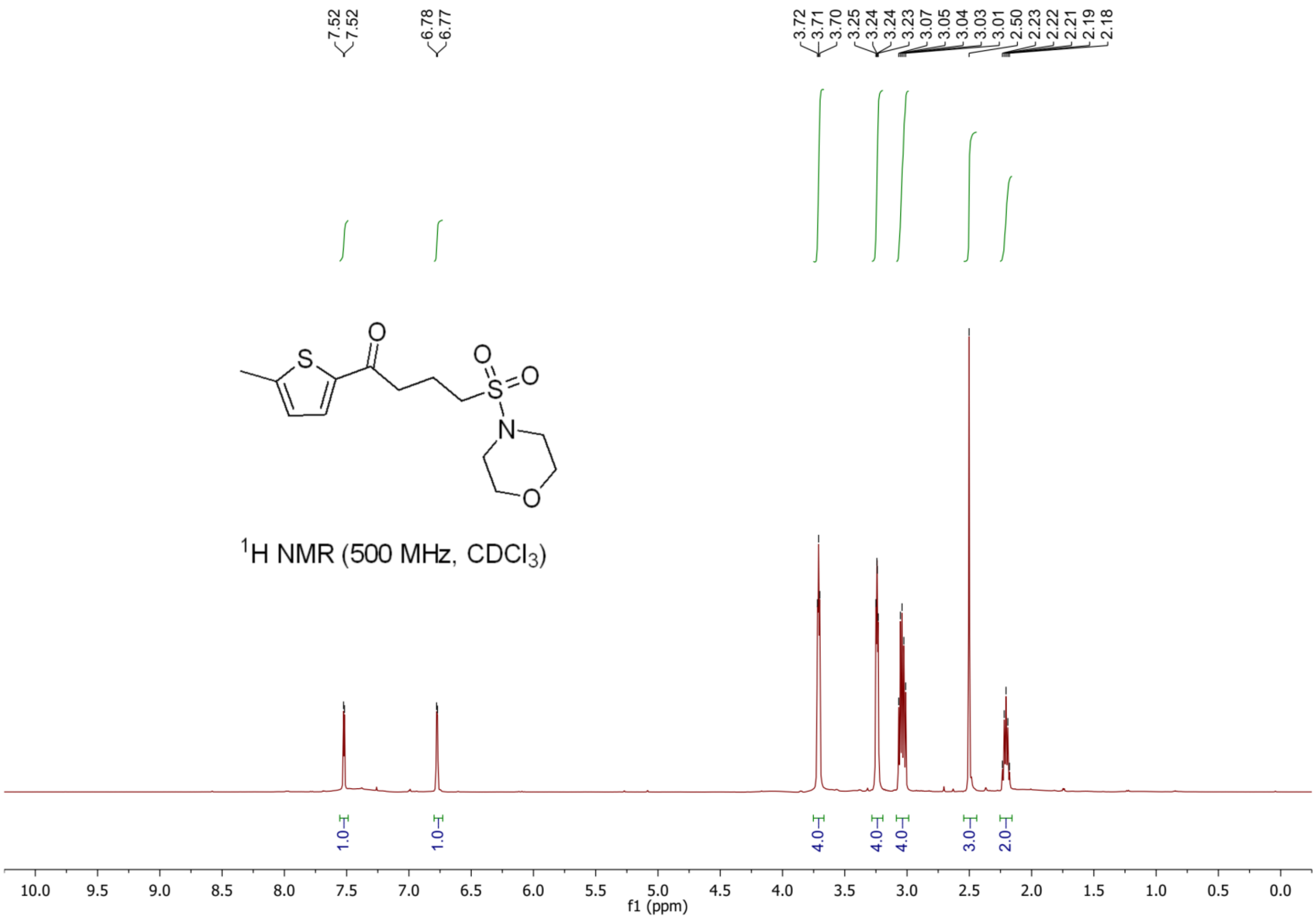

Go back to table of contents

Evaluation Only. Created with Aspose.Pdf. Copyright 2002-2014 Aspose Pty Ltd.

1-(5-Methylthiophen-2-yl)-4-(morpholinosulfonyl)butan-1-one (1f)

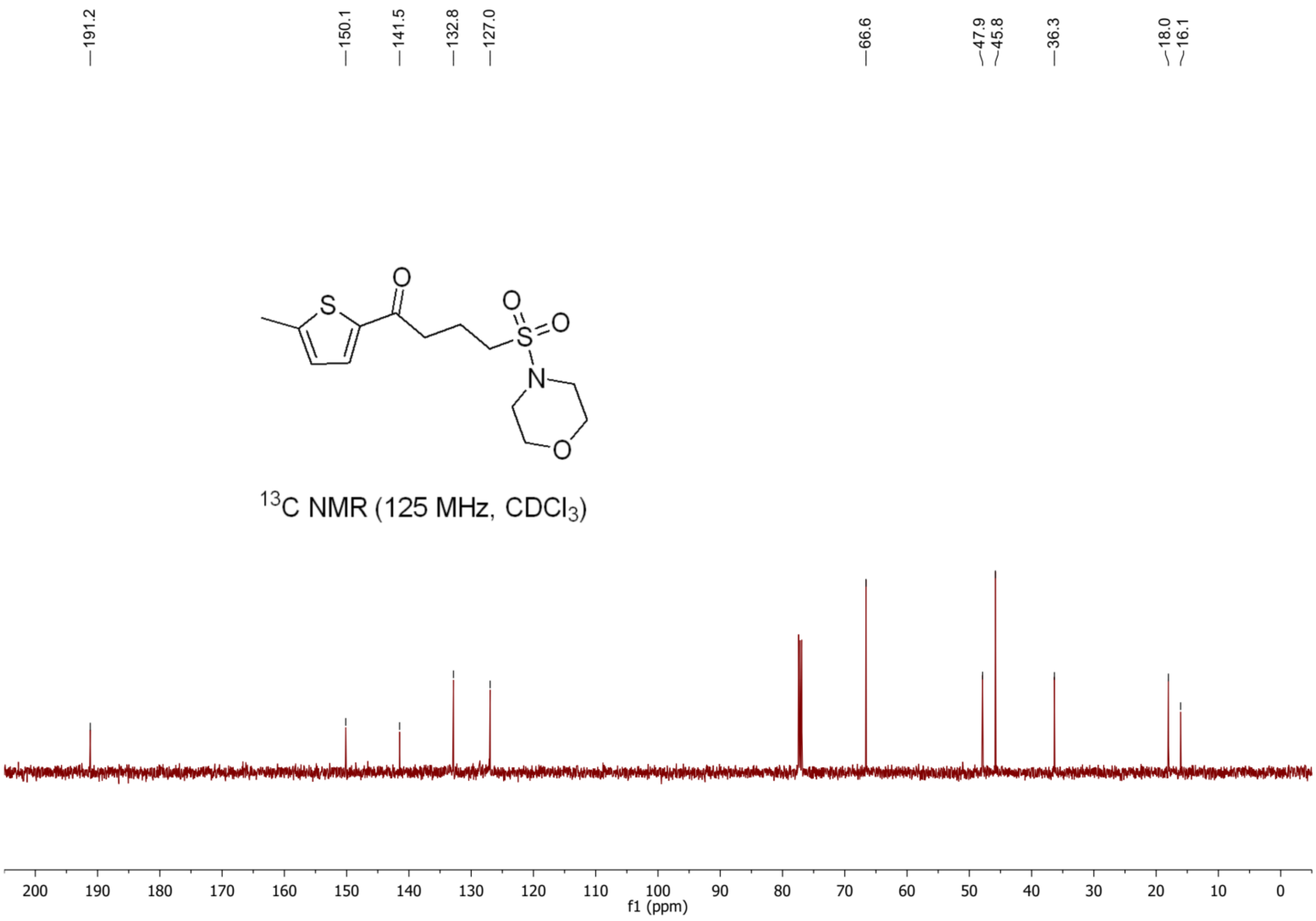

Go back to table of contents

Evaluation Only. Created with Aspose.Pdf. Copyright 2002-2014 Aspose Pty Ltd.

1,7-Bis(morpholinosulfonyl)heptane (1g)

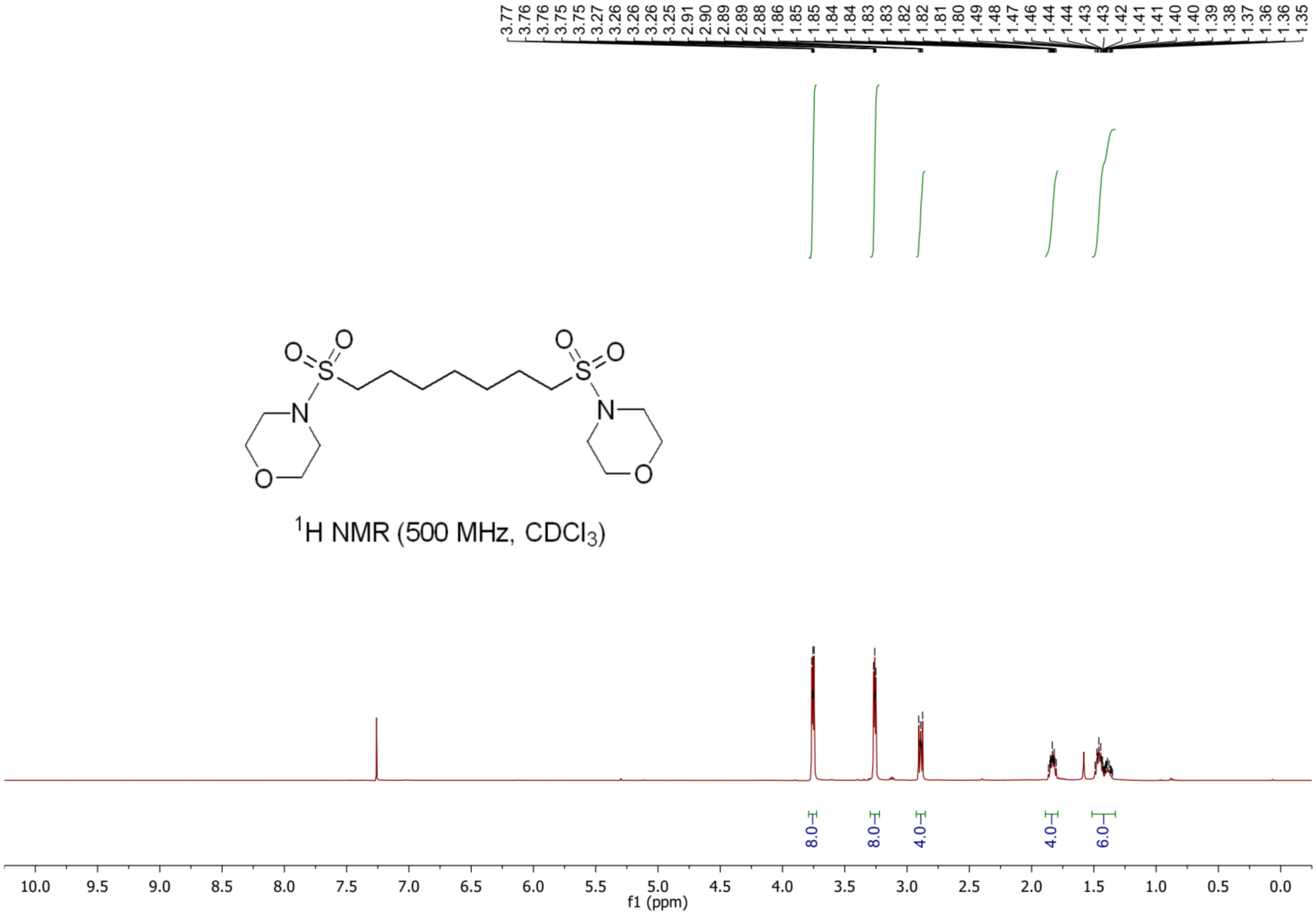

^{1}H NMR (500 MHz, $CDCl_3$)

Go back to table of contents

Evaluation Only. Created with Aspose.Pdf. Copyright 2002-2014 Aspose Pty Ltd.

1,7-Bis(morpholinosulfonyl)heptane (1g)

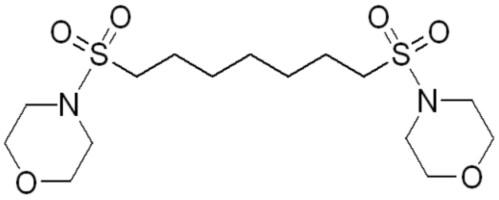

^{13}C NMR (125 MHz, $CDCl_3$)

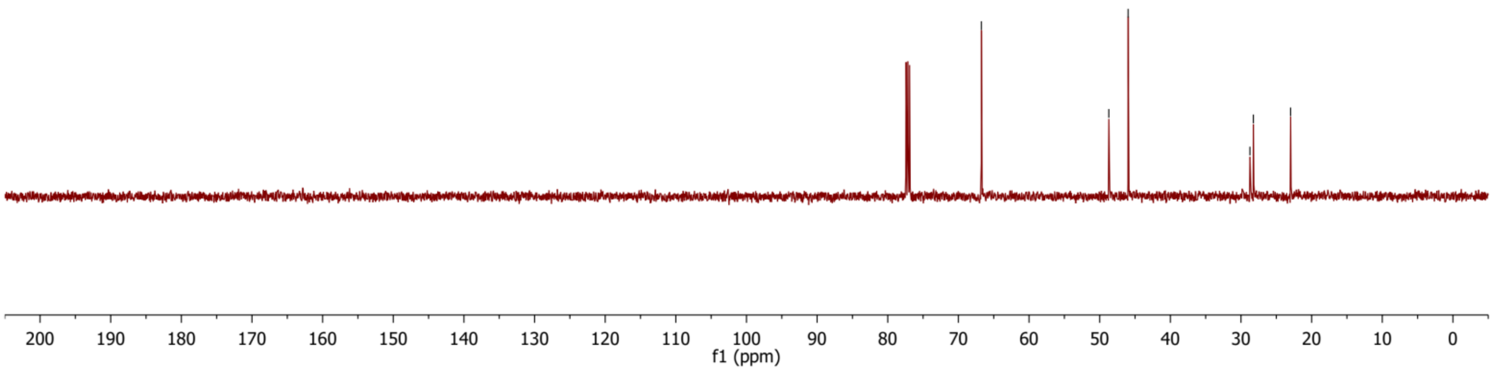

Go back to table of contents

Evaluation Only. Created with Aspose.Pdf. Copyright 2002-2014 Aspose Pty Ltd.

4-(Heptan-4-ylsulfonyl)morpholine (1h)

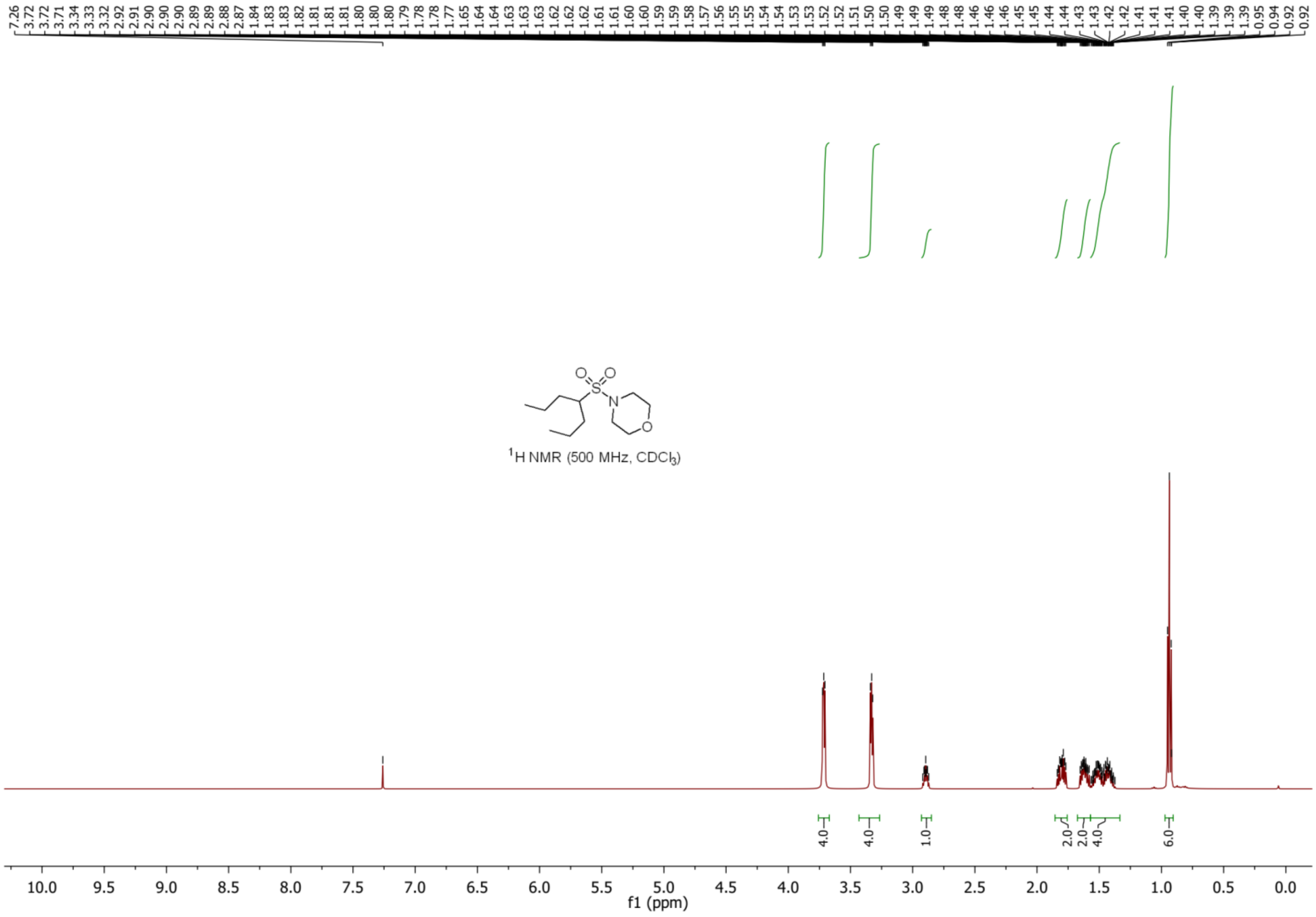

Go back to table of contents

Evaluation Only. Created with Aspose.Pdf. Copyright 2002-2014 Aspose Pty Ltd.

4-(Heptan-4-ylsulfonyl)morpholine (1h)

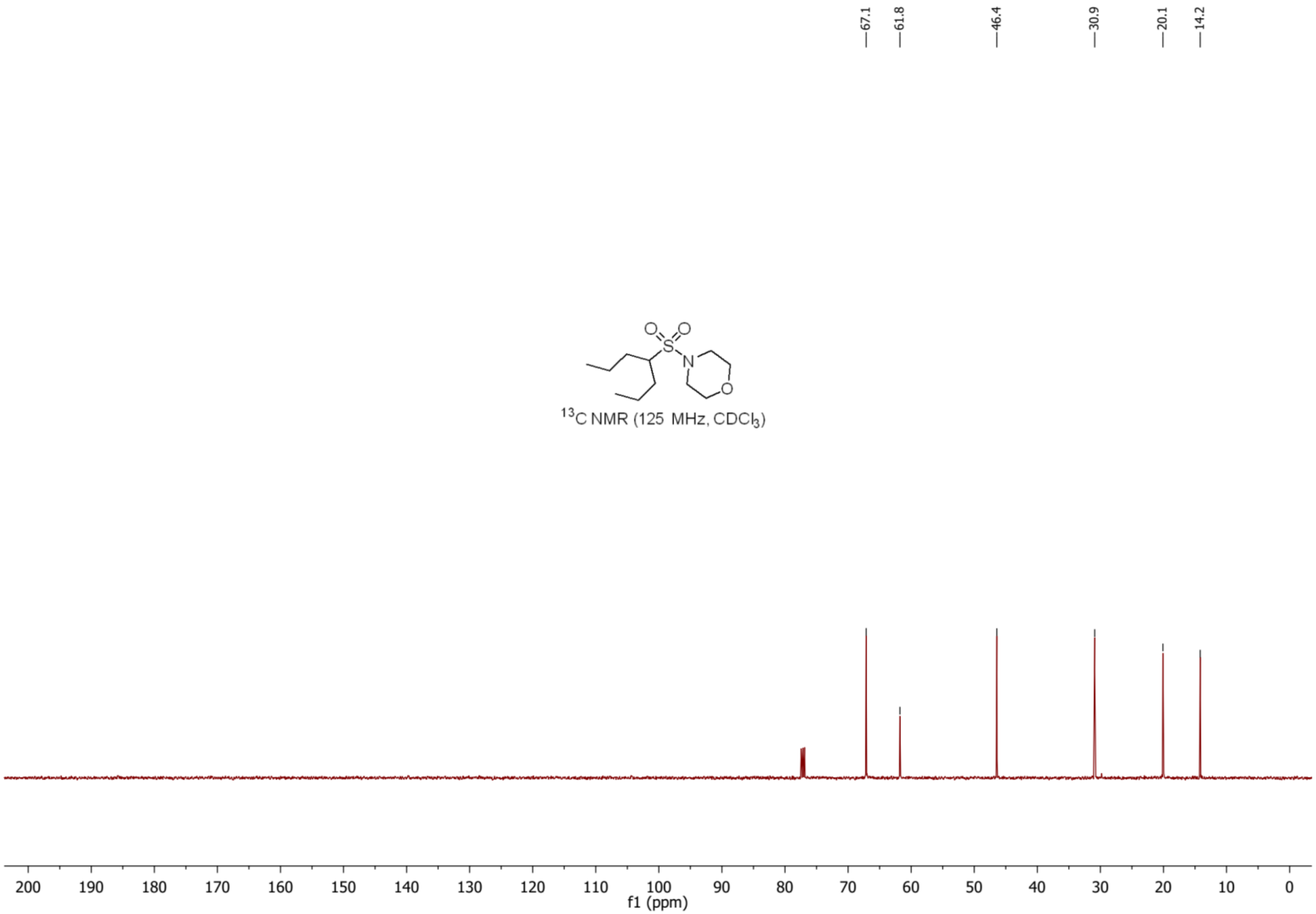

Go back to table of contents

Evaluation Only. Created with Aspose.Pdf. Copyright 2002-2014 Aspose Pty Ltd.

4-((2,3-Dihydro-1*H*-inden-2-yl)sulfonyl)morpholine (1i)

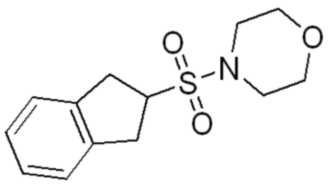

^{1}H NMR (500 MHz, $CDCl_3$)

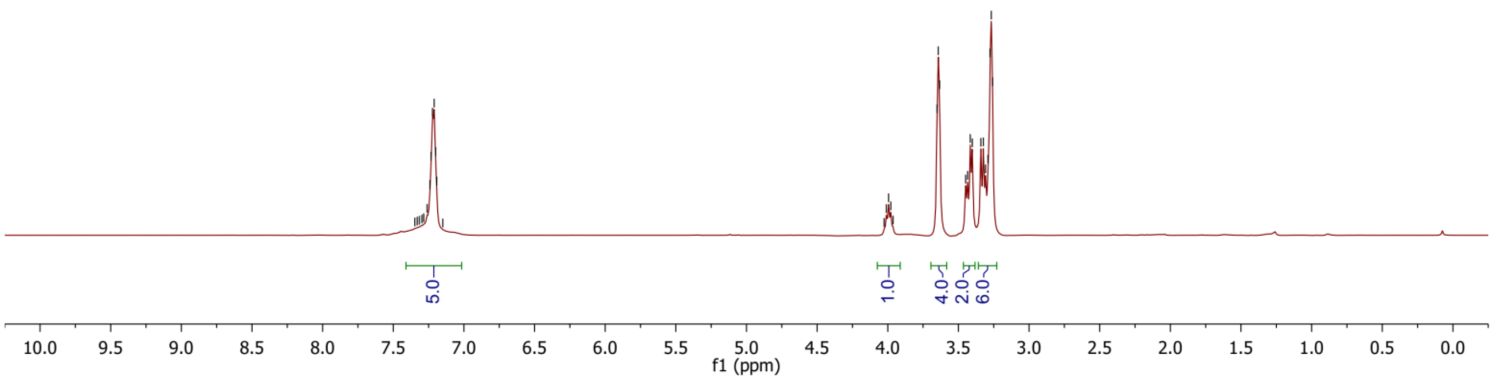

Go back to table of contents

Evaluation Only. Created with Aspose.Pdf. Copyright 2002-2014 Aspose Pty Ltd.

4-((2,3-Dihydro-1*H*-inden-2-yl)sulfonyl)morpholine (1i)

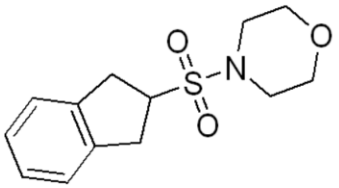

^{13}C NMR (125 MHz, $CDCl_3$)

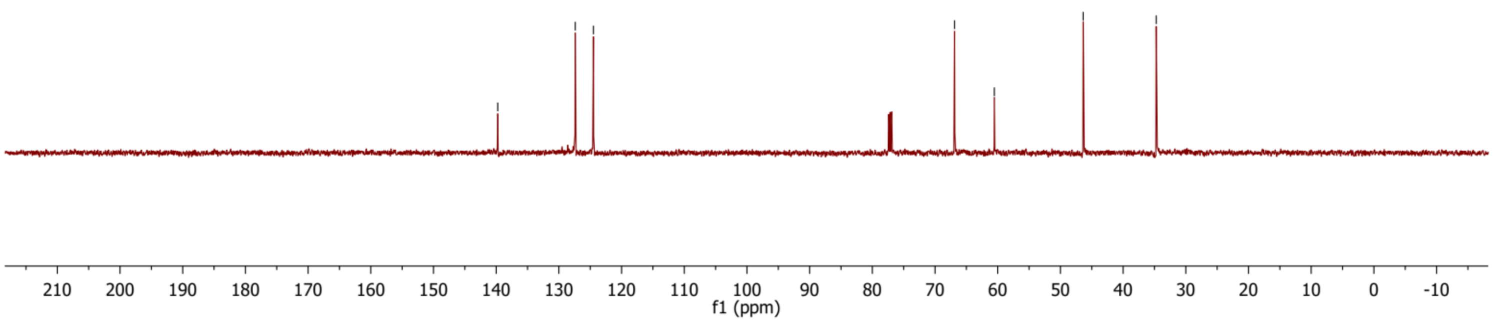

Go back to table of contents

Evaluation Only. Created with Aspose.Pdf. Copyright 2002-2014 Aspose Pty Ltd.

4-((4,4-Difluorocyclohexyl)sulfonyl)morpholine (1j)

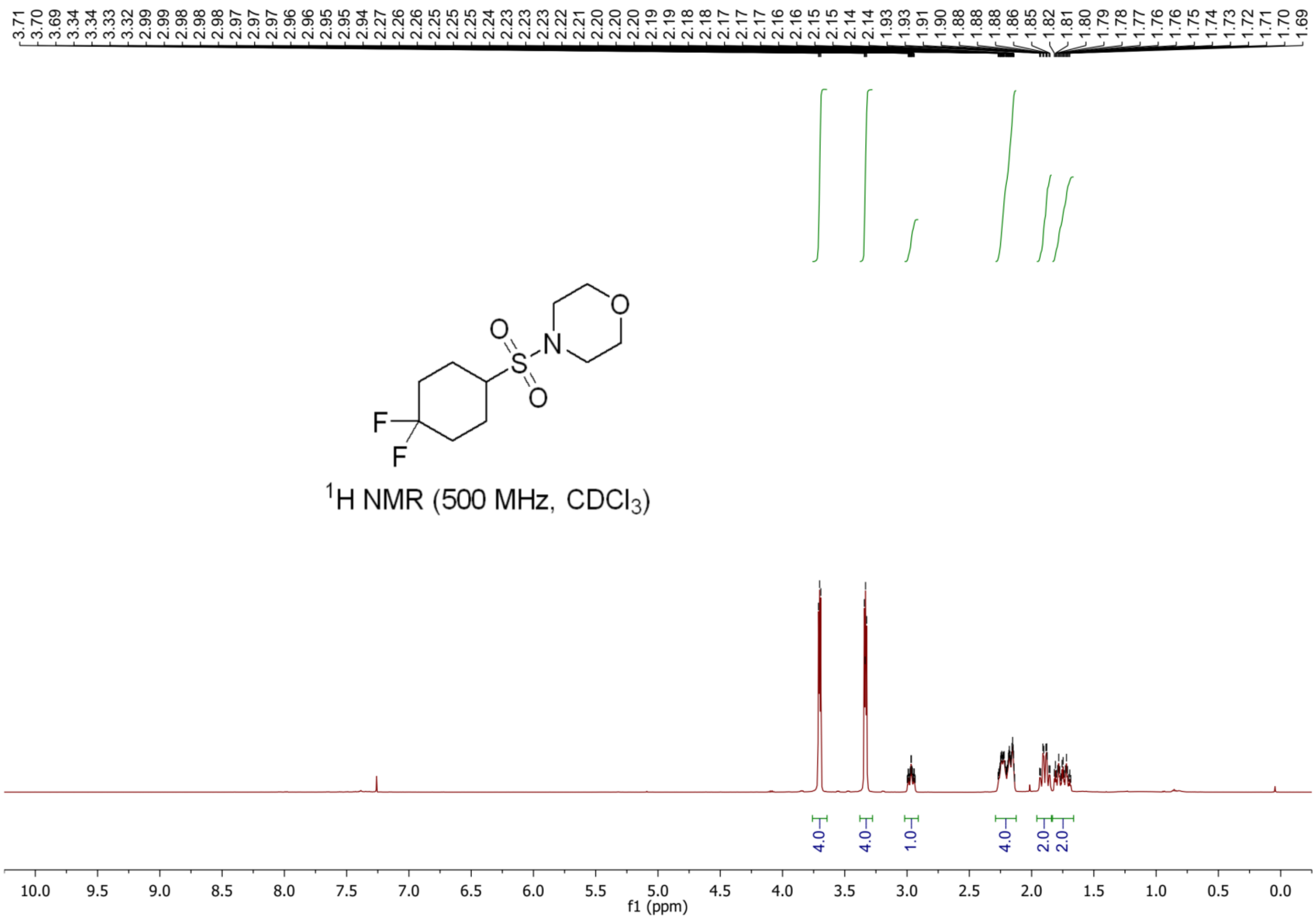

Go back to table of contents

Evaluation Only. Created with Aspose.Pdf. Copyright 2002-2014 Aspose Pty Ltd.

4-((4,4-Difluorocyclohexyl)sulfonyl)morpholine (1j)

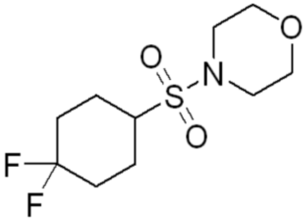

^{13}C NMR (125 MHz, $CDCl_3$)

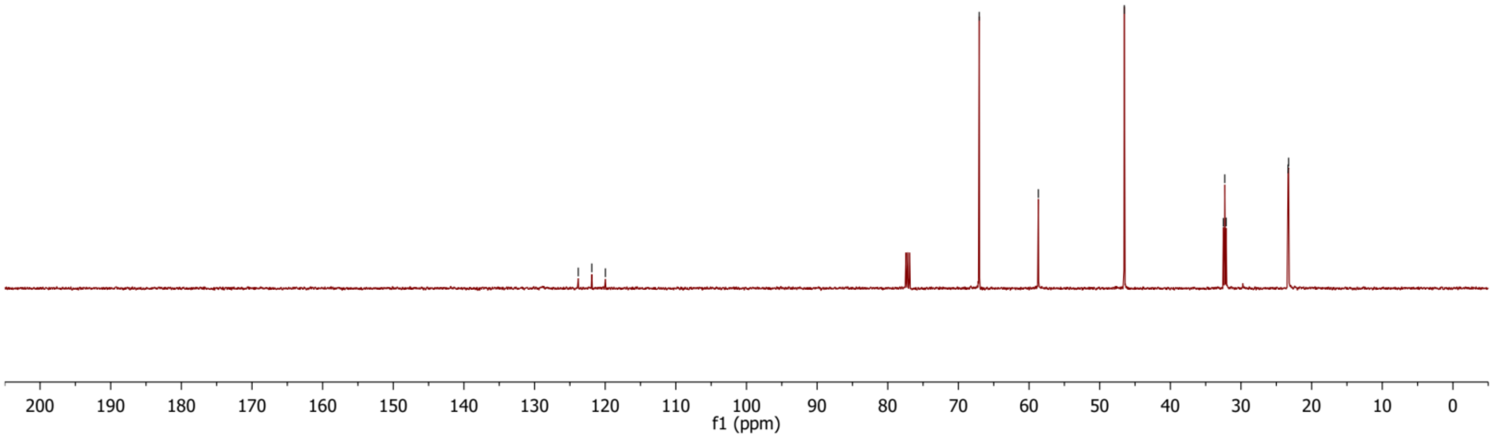

Go back to table of contents

Evaluation Only. Created with Aspose.Pdf. Copyright 2002-2014 Aspose Pty Ltd.

tert-Butyl 4-(morpholinosulfonyl)piperidine-1-carboxylate (1k)

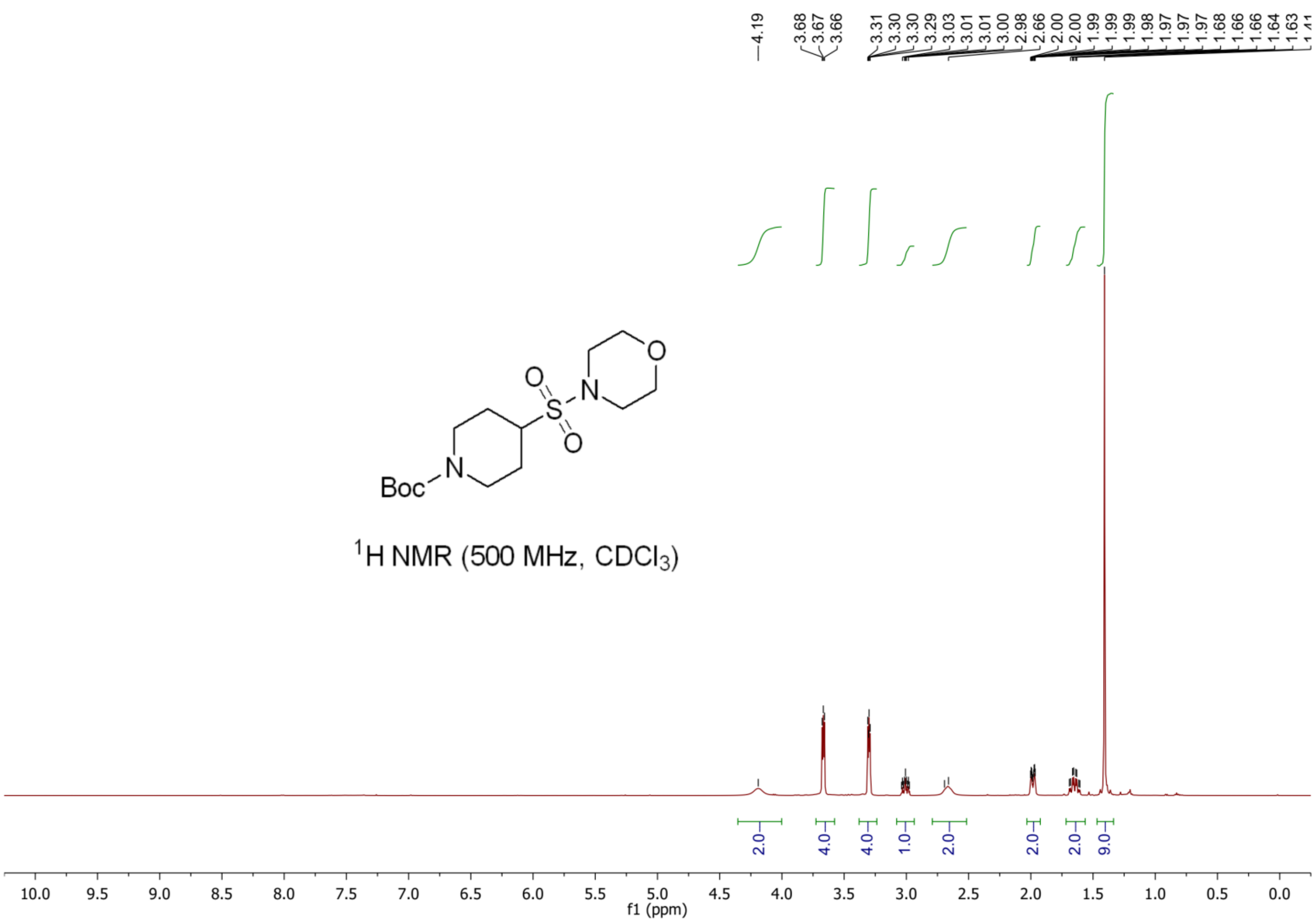

Go back to table of contents

Evaluation Only. Created with Aspose.Pdf. Copyright 2002-2014 Aspose Pty Ltd.

tert-Butyl 4-(morpholinosulfonyl)piperidine-1-carboxylate (1k)

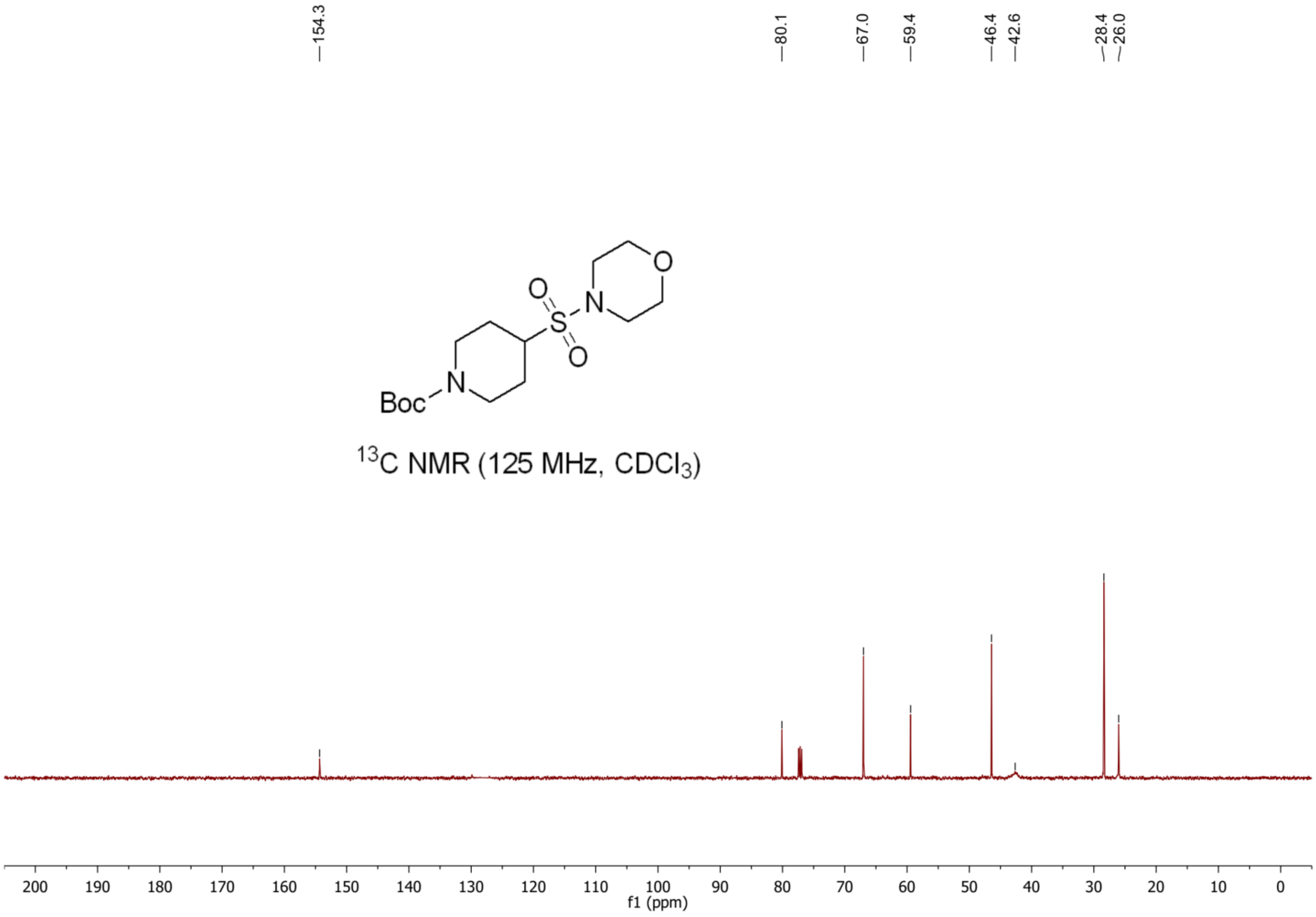

Go back to table of contents

Evaluation Only. Created with Aspose.Pdf. Copyright 2002-2014 Aspose Pty Ltd.

4-((Tetrahydro-2*H*-pyran-4-yl)sulfonyl)morpholine (1l)

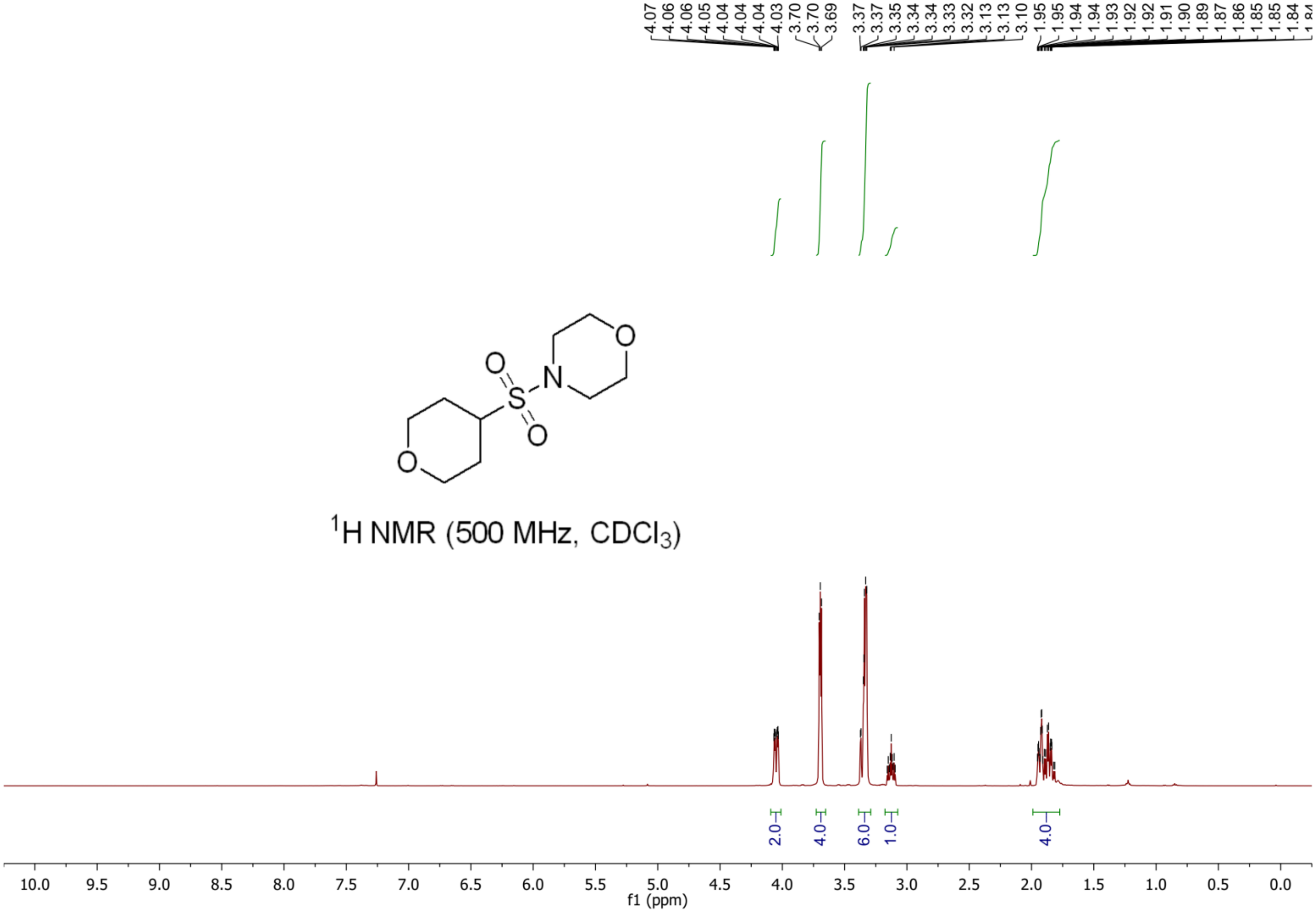

Go back to table of contents

Evaluation Only. Created with Aspose.Pdf. Copyright 2002-2014 Aspose Pty Ltd.

4-((Tetrahydro-2*H*-pyran-4-yl)sulfonyl)morpholine (1l)

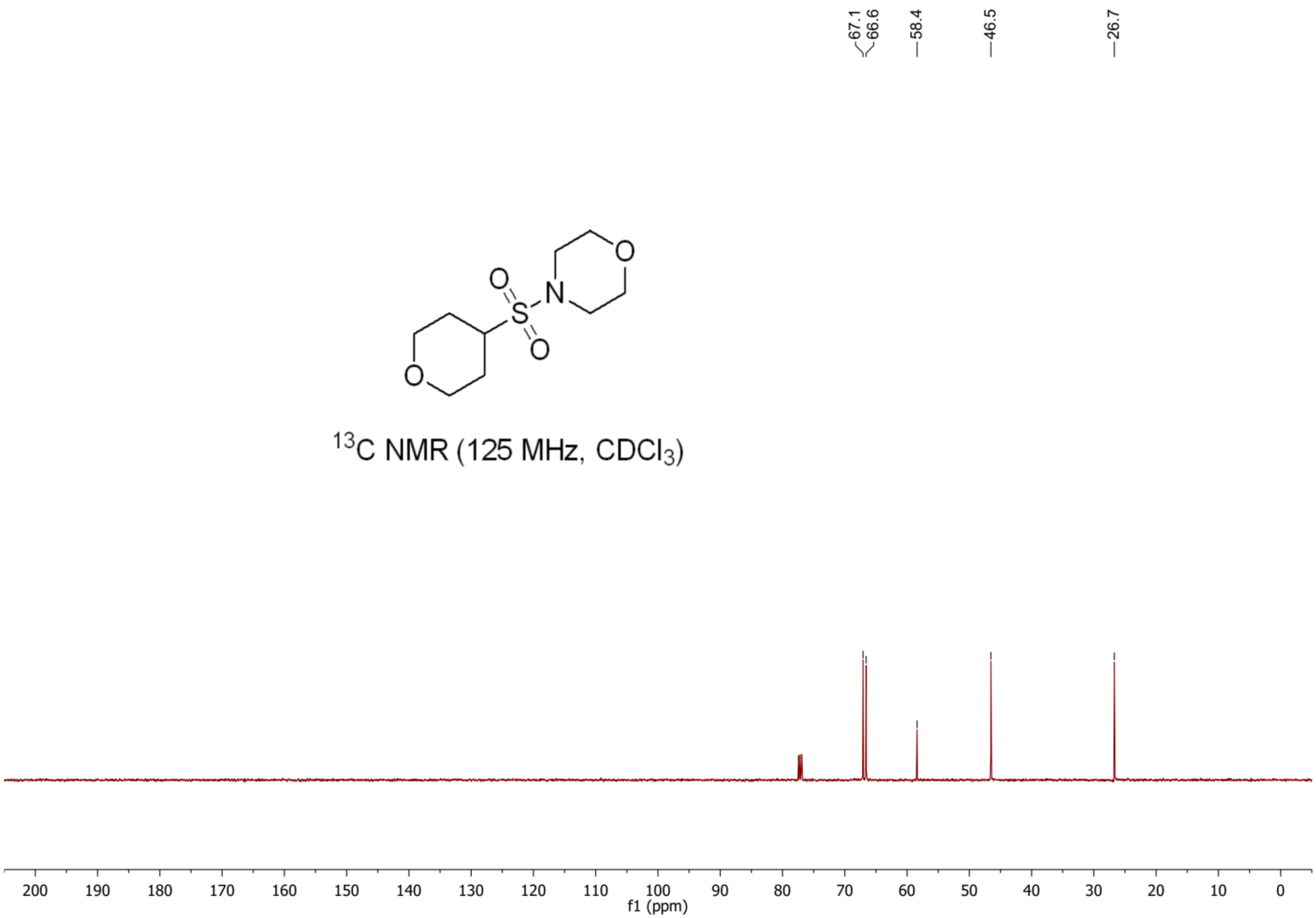

Go back to table of contents

Evaluation Only. Created with Aspose.Pdf. Copyright 2002-2014 Aspose Pty Ltd.

Methyl (1*S*,2*R*)-2-(morpholinosulfonyl)cyclohexane-1-carboxylate (1m)

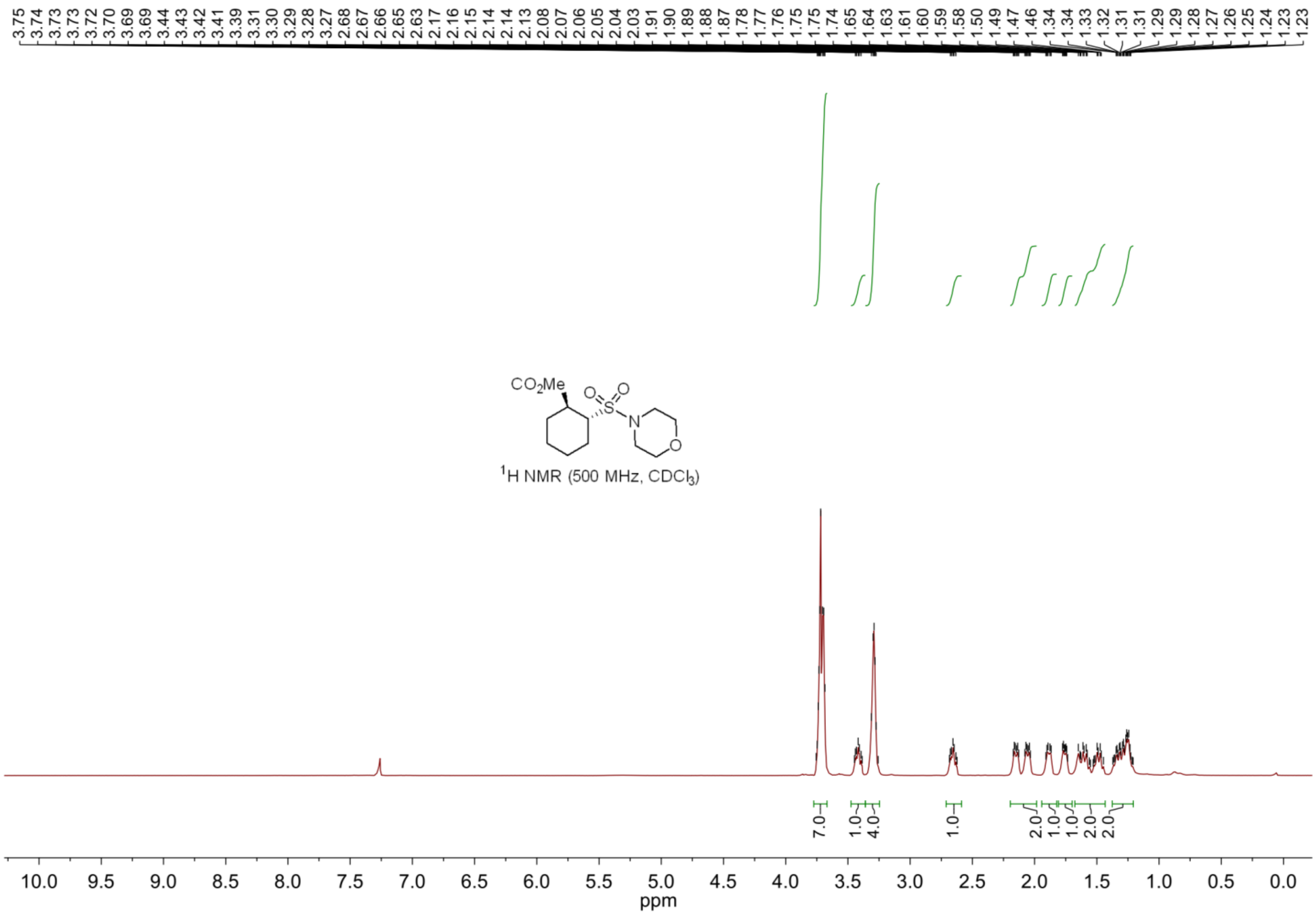

Evaluation Only. Created with Aspose.Pdf. Copyright 2002-2014 Aspose Pty Ltd.

Methyl (1*S*,2*R*)-2-(morpholinosulfonyl)cyclohexane-1-carboxylate (1m)

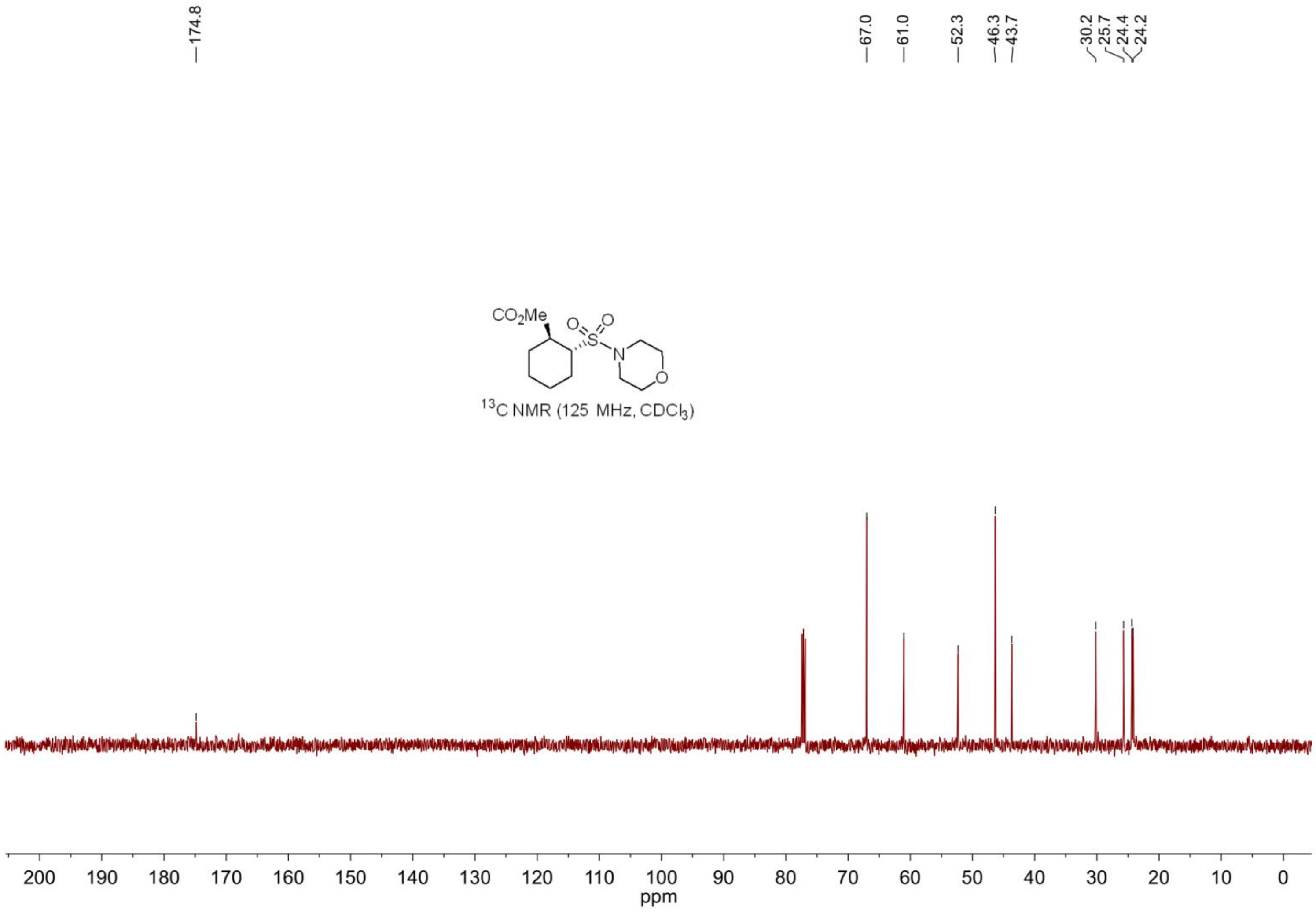

Go back to table of contents

Evaluation Only. Created with Aspose.Pdf. Copyright 2002-2014 Aspose Pty Ltd.

Ethyl (1*R*,2*S*,4*S*)-3-(morpholinosulfonyl)bicyclo[2.2.1]heptane-2-carboxylate (1n)

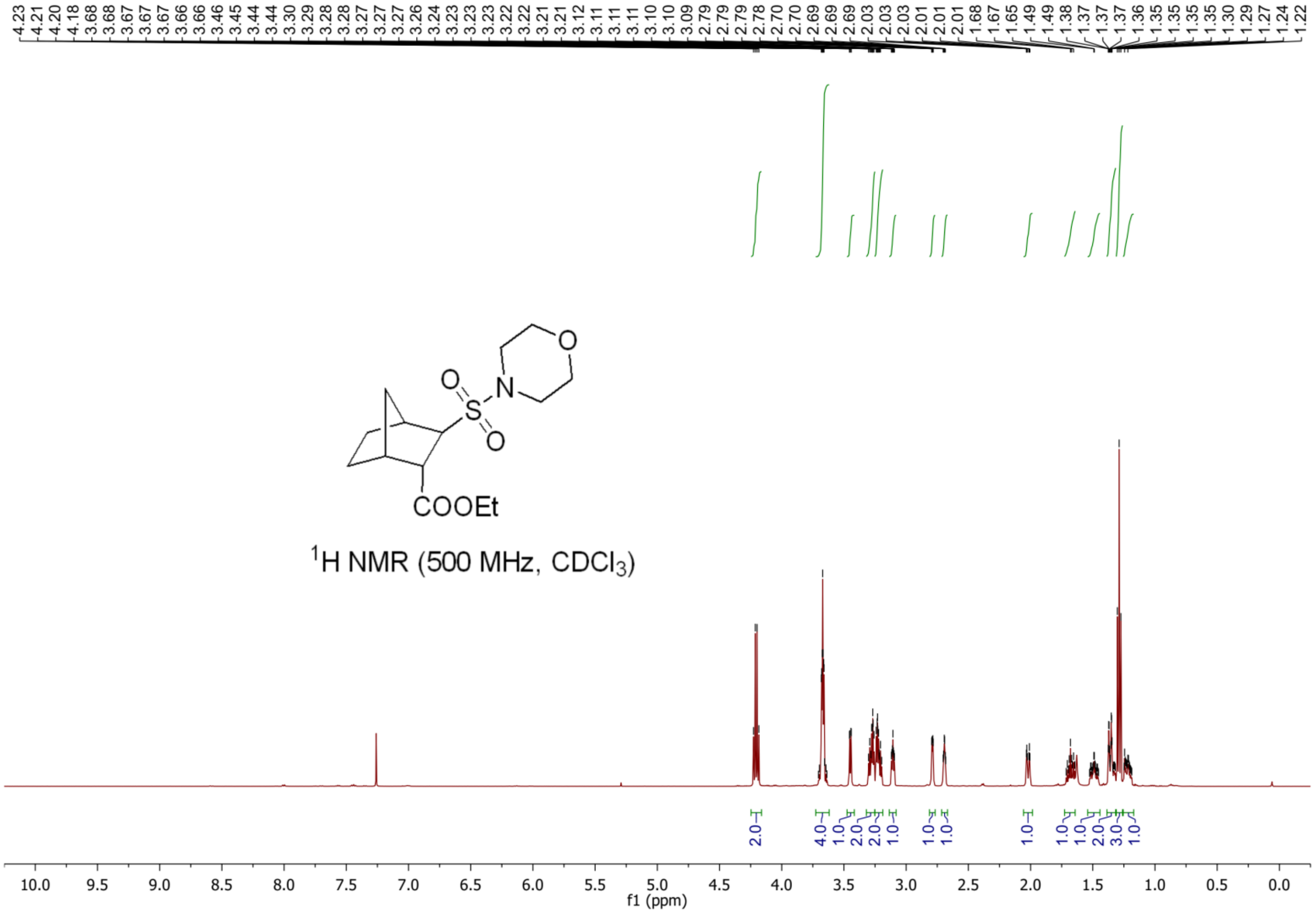

Go back to table of contents

Evaluation Only. Created with Aspose.Pdf. Copyright 2002-2014 Aspose Pty Ltd.

Ethyl (1*R*,2*S*,4*S*)-3-(morpholinosulfonyl)bicyclo[2.2.1]heptane-2-carboxylate (1n)

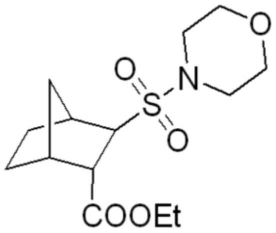

^{13}C NMR (125 MHz, CDCl$_3$)

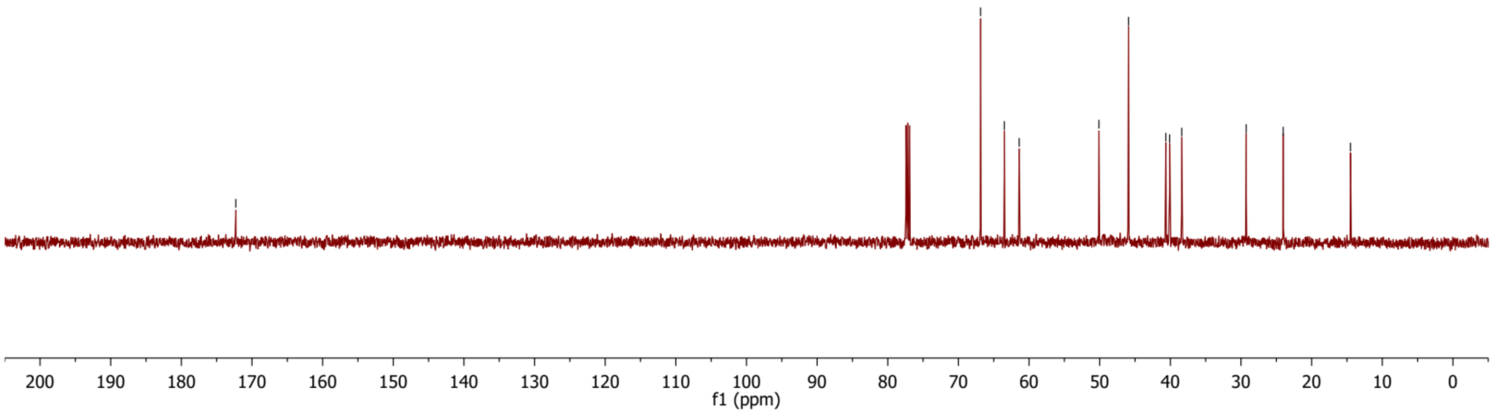

Go back to table of contents

Evaluation Only. Created with Aspose.Pdf. Copyright 2002-2014 Aspose Pty Ltd.

4-((1-Phenylcyclopropyl)sulfonyl)morpholine (1o)

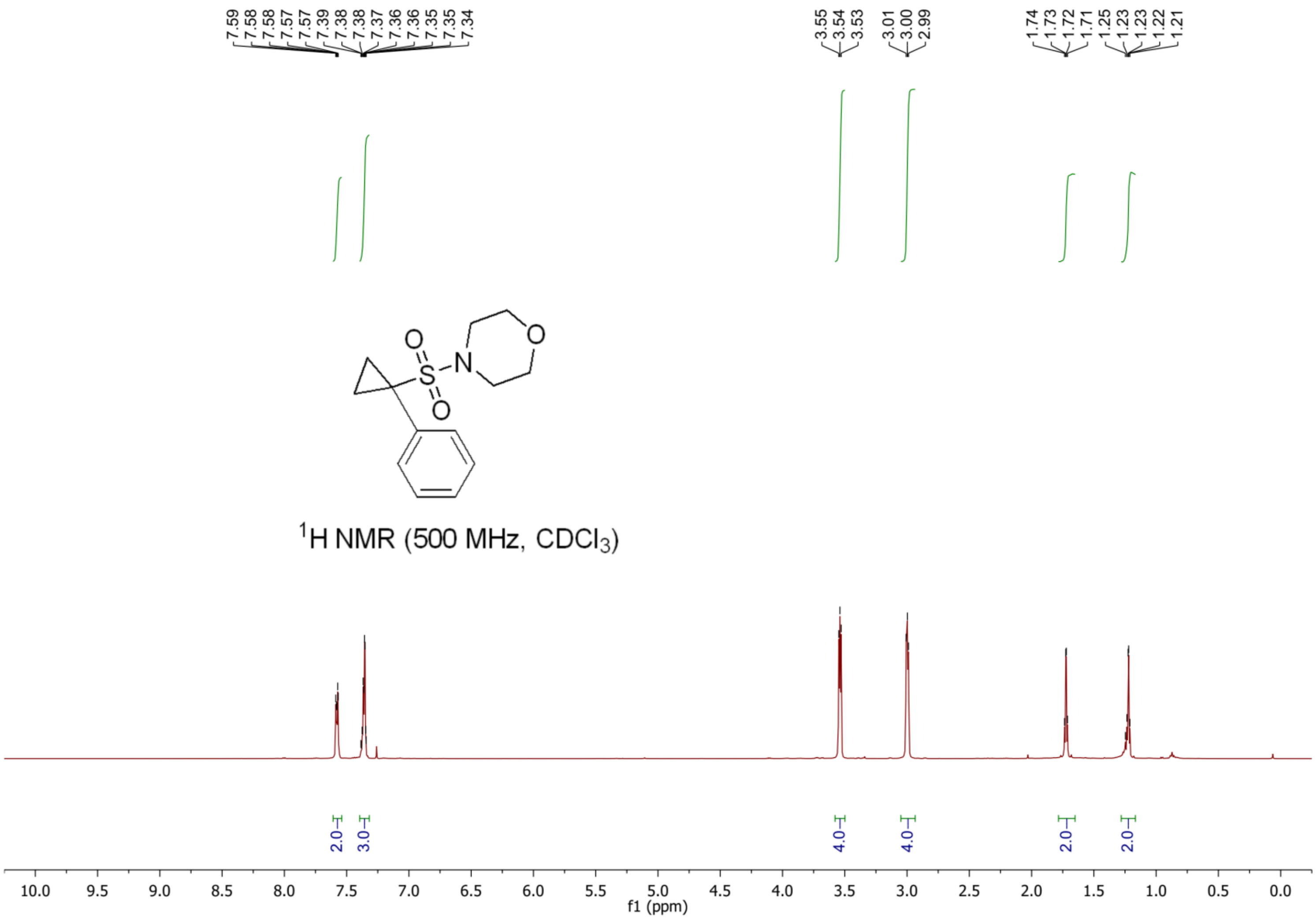

Go back to table of contents

Evaluation Only. Created with Aspose.Pdf. Copyright 2002-2014 Aspose Pty Ltd.

4-((1-Phenylcyclopropyl)sulfonyl)morpholine (1o)

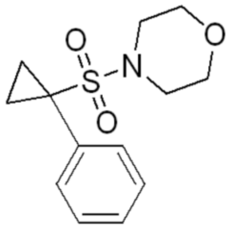

^{13}C NMR (125 MHz, $CDCl_3$)

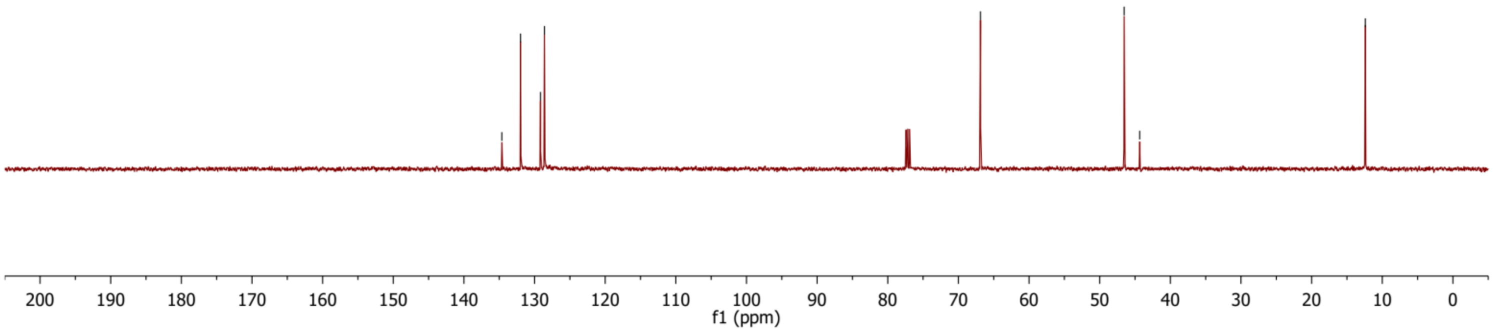

Go back to table of contents

Evaluation Only. Created with Aspose.Pdf. Copyright 2002-2014 Aspose Pty Ltd.

4-((4-Methyltetrahydro-2*H*-pyran-4-yl)sulfonyl)morpholine (1p)

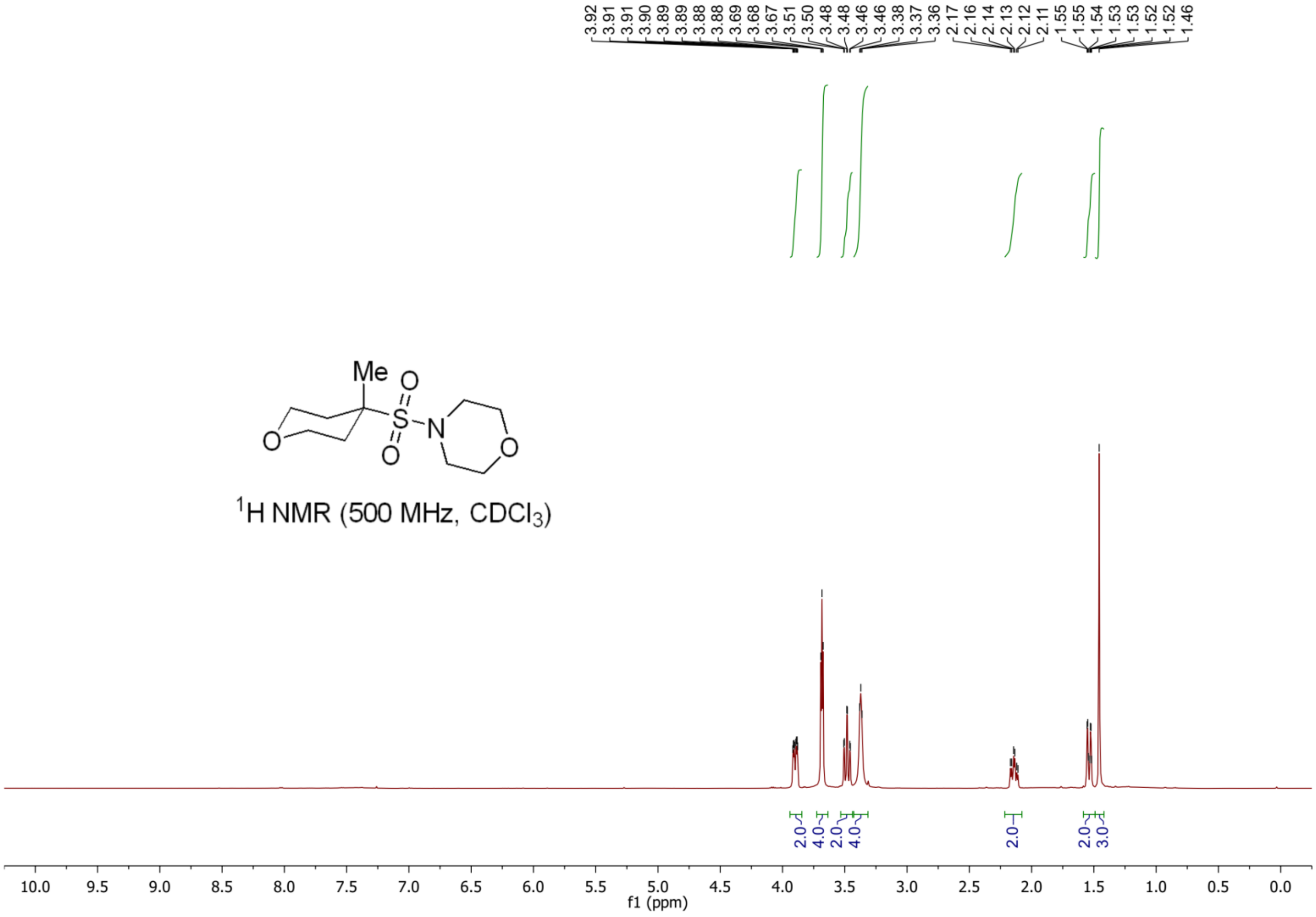

Go back to table of contents

Evaluation Only. Created with Aspose.Pdf. Copyright 2002-2014 Aspose Pty Ltd.

4-((4-Methyltetrahydro-2*H*-pyran-4-yl)sulfonyl)morpholine (1p)

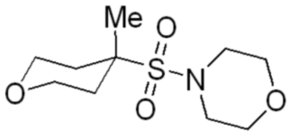

^{13}C NMR (125 MHz, $CDCl_3$)

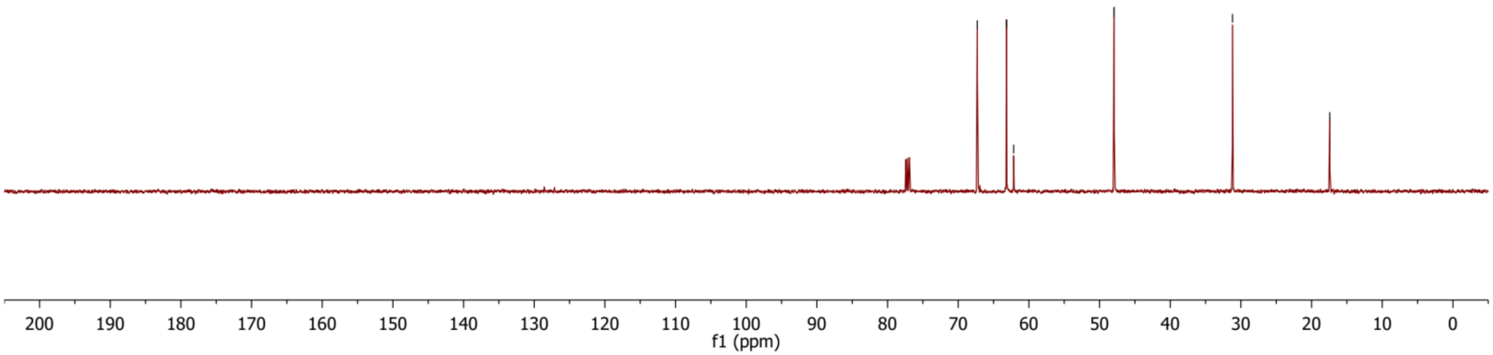

Go back to table of contents

Evaluation Only. Created with Aspose.Pdf. Copyright 2002-2014 Aspose Pty Ltd.

1-Phenyl-4-(piperidin-1-ylsulfonyl)butan-1-one (2a)

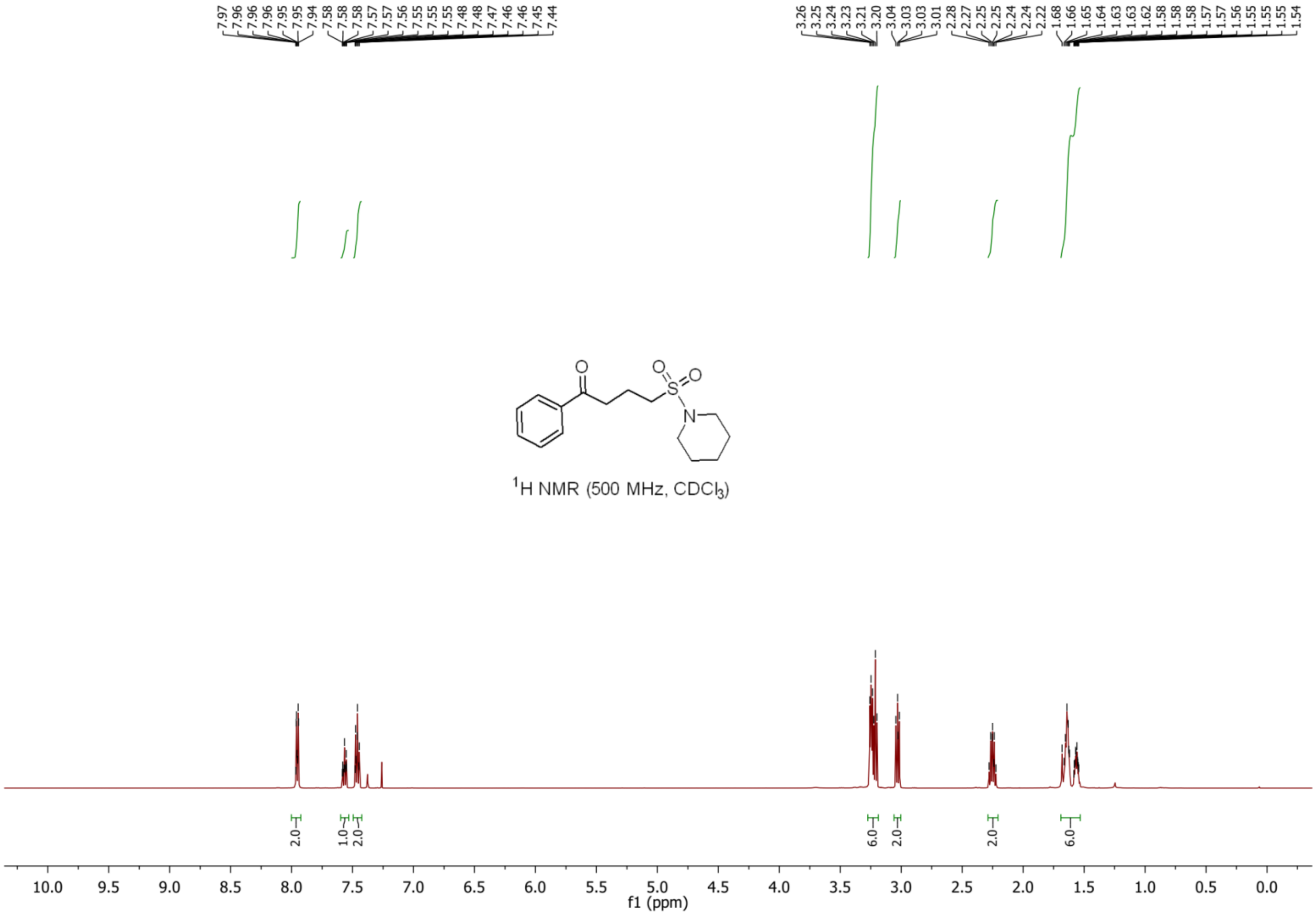

Go back to table of contents

Evaluation Only. Created with Aspose.Pdf. Copyright 2002-2014 Aspose Pty Ltd.

1-Phenyl-4-(piperidin-1-ylsulfonyl)butan-1-one (2a)

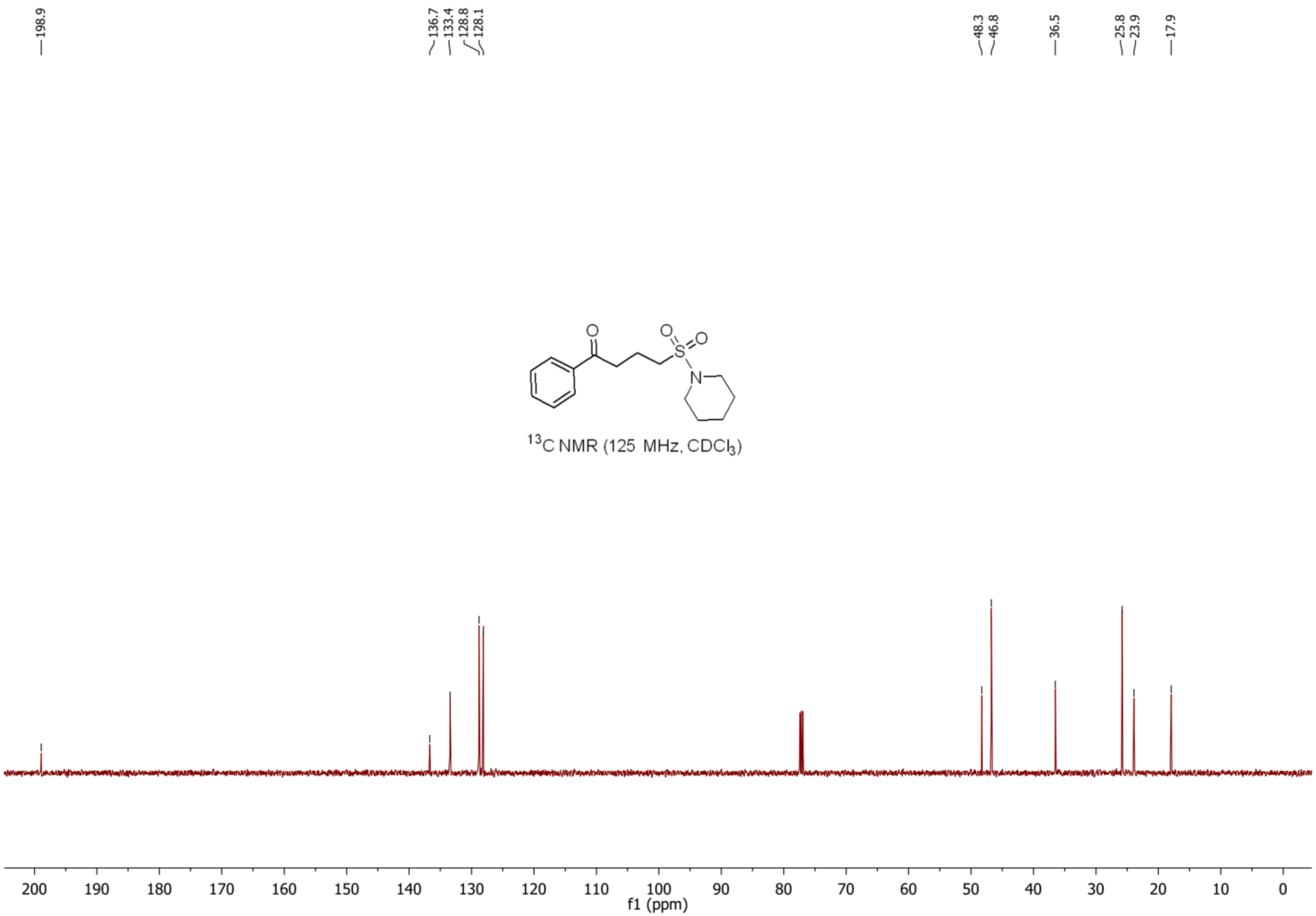

Go back to table of contents

Evaluation Only. Created with Aspose.Pdf. Copyright 2002-2014 Aspose Pty Ltd.

1-((Tetrahydro-2*H*-pyran-4-yl)sulfonyl)piperidine (2b)

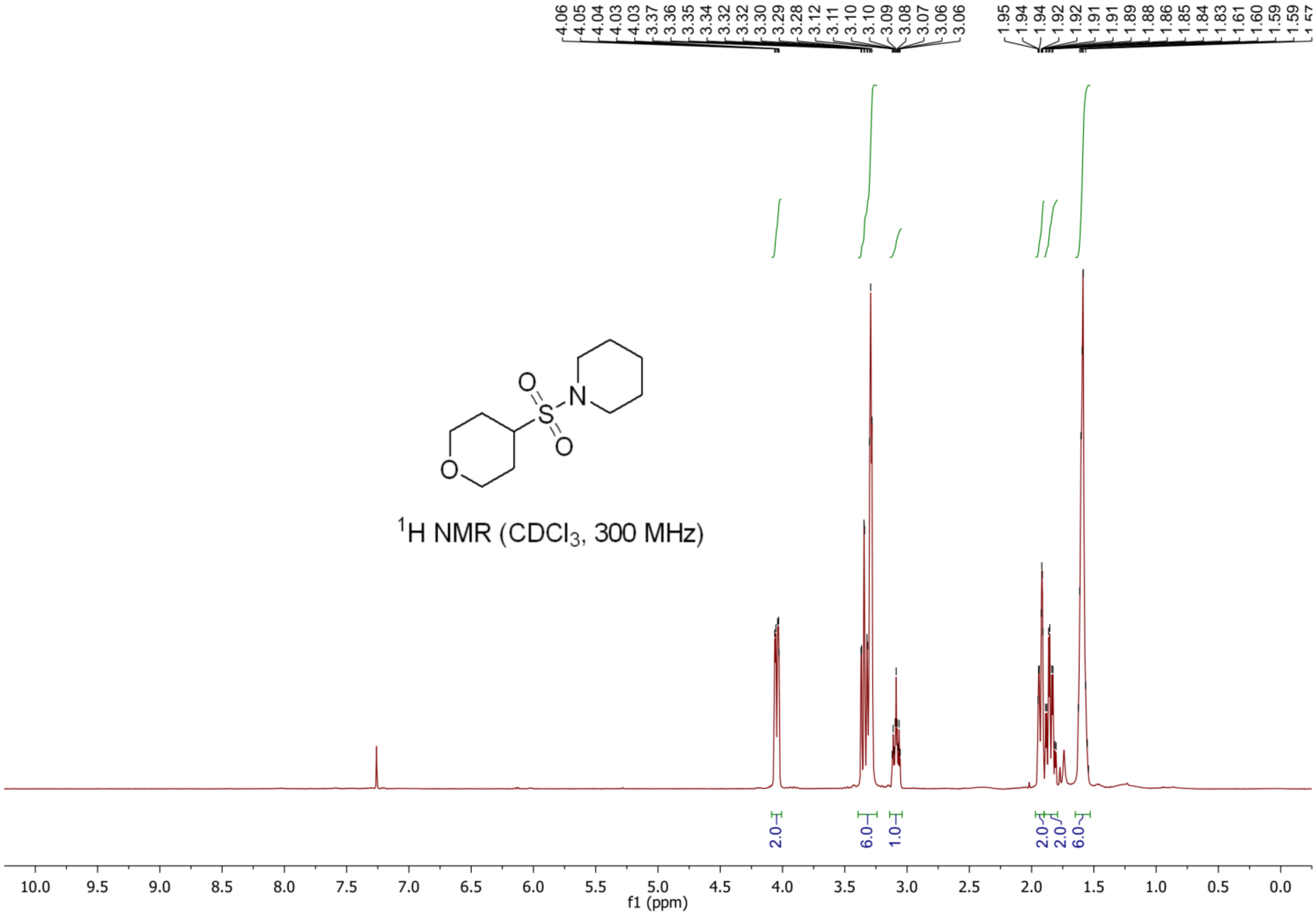

Go back to table of contents

Evaluation Only. Created with Aspose.Pdf. Copyright 2002-2014 Aspose Pty Ltd.

1-((Tetrahydro-2*H*-pyran-4-yl)sulfonyl)piperidine (2b)

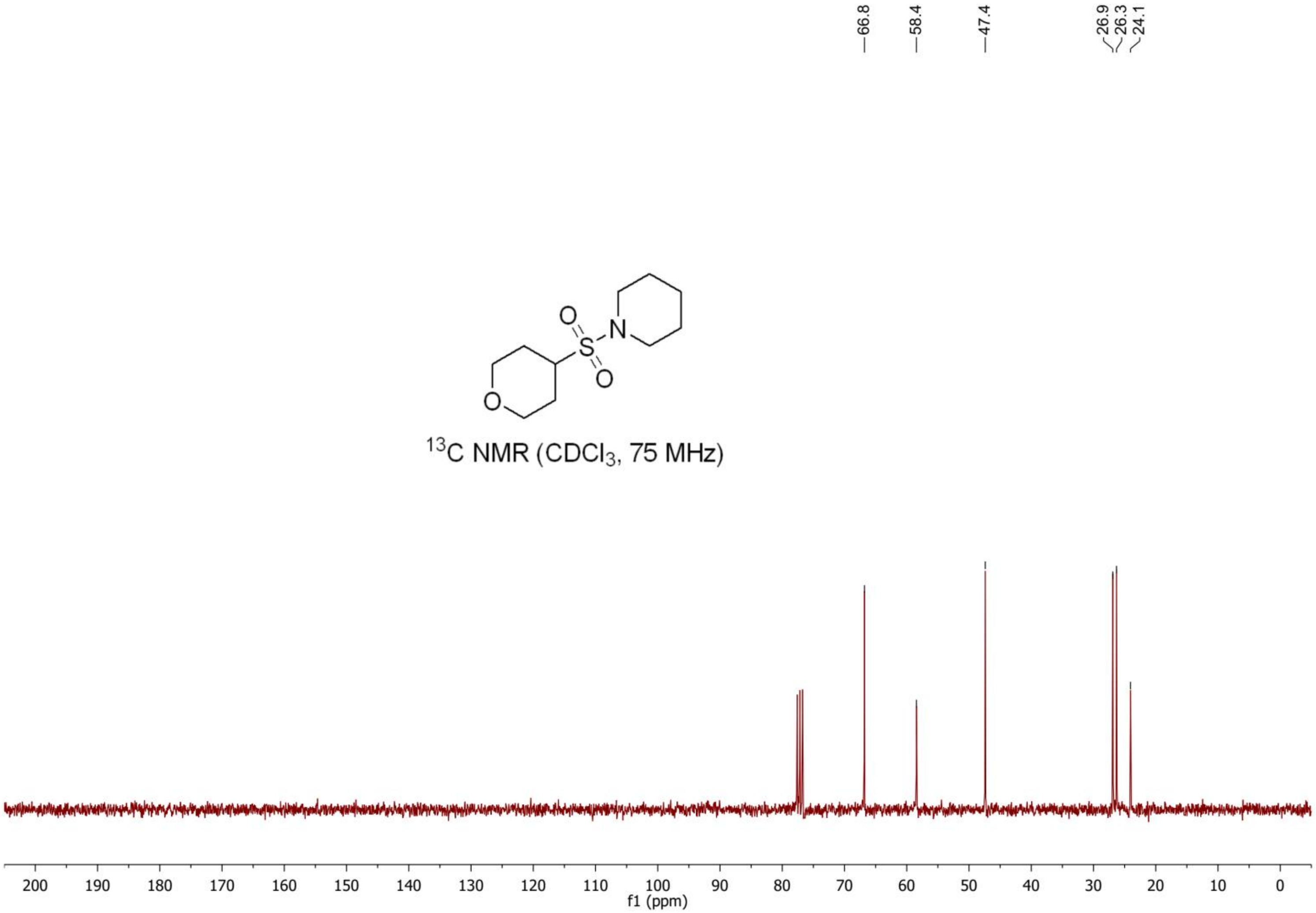

^{13}C NMR ($CDCl_3$, 75 MHz)

Go back to table of contents

Evaluation Only. Created with Aspose.Pdf. Copyright 2002-2014 Aspose Pty Ltd.

1-(Heptan-4-ylsulfonyl)piperidine (2c)

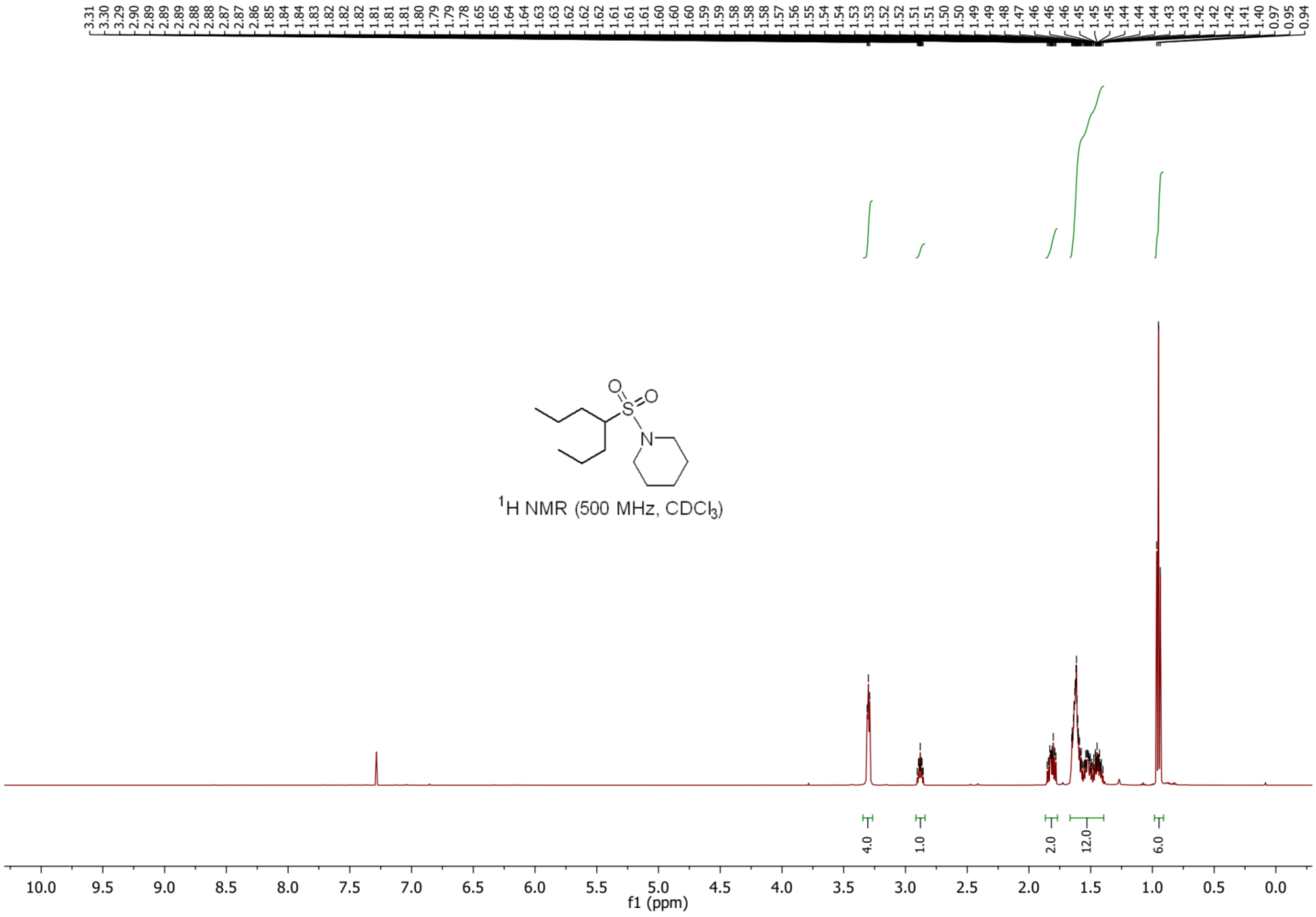

Go back to table of contents

Evaluation Only. Created with Aspose.Pdf. Copyright 2002-2014 Aspose Pty Ltd.

1-(Heptan-4-ylsulfonyl)piperidine (2c)

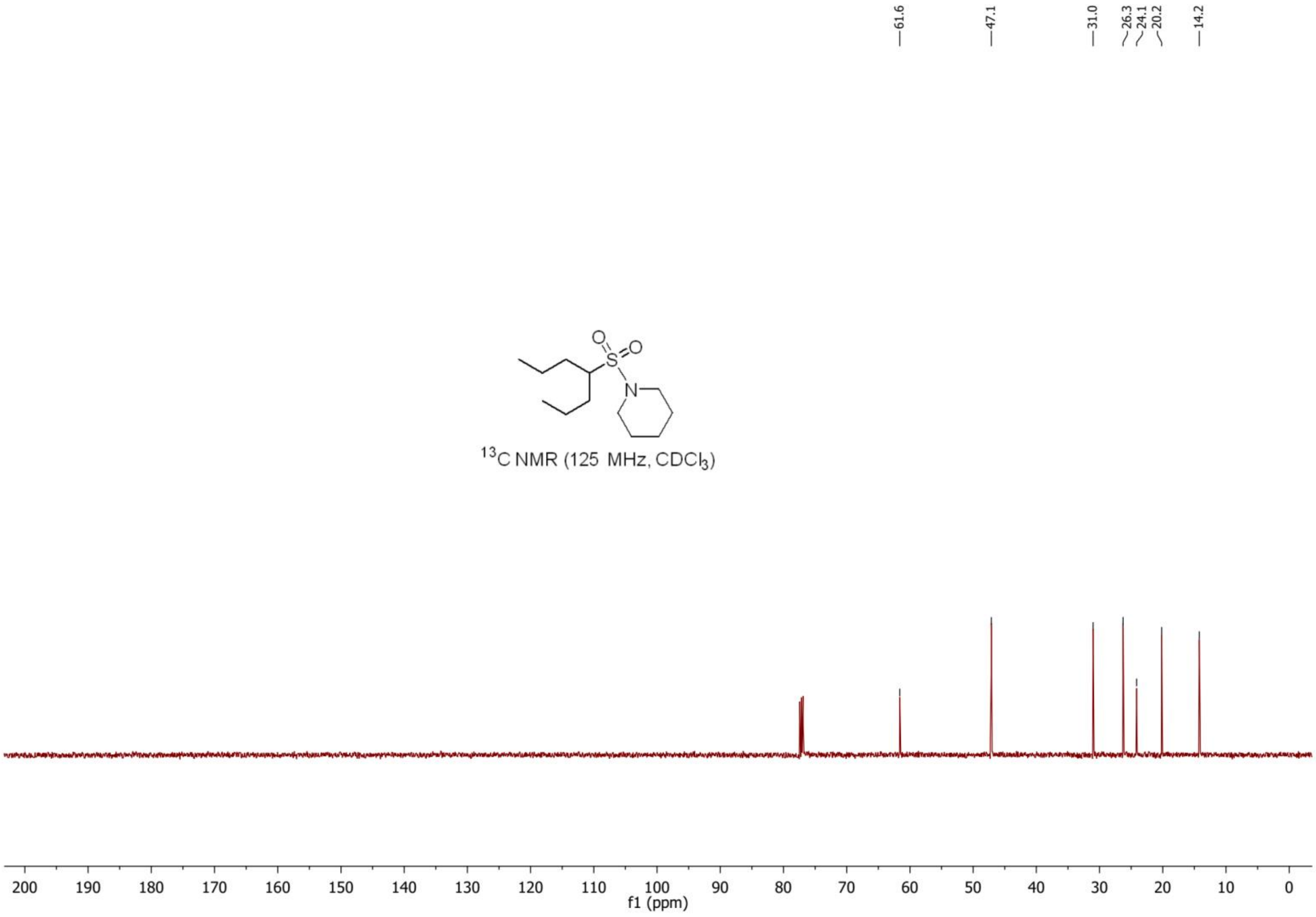

Go back to table of contents

Evaluation Only. Created with Aspose.Pdf. Copyright 2002-2014 Aspose Pty Ltd.

Methyl (1*S,2*R**)-2-(piperidin-1-ylsulfonyl)cyclohexane-1-carboxylate (2d)**

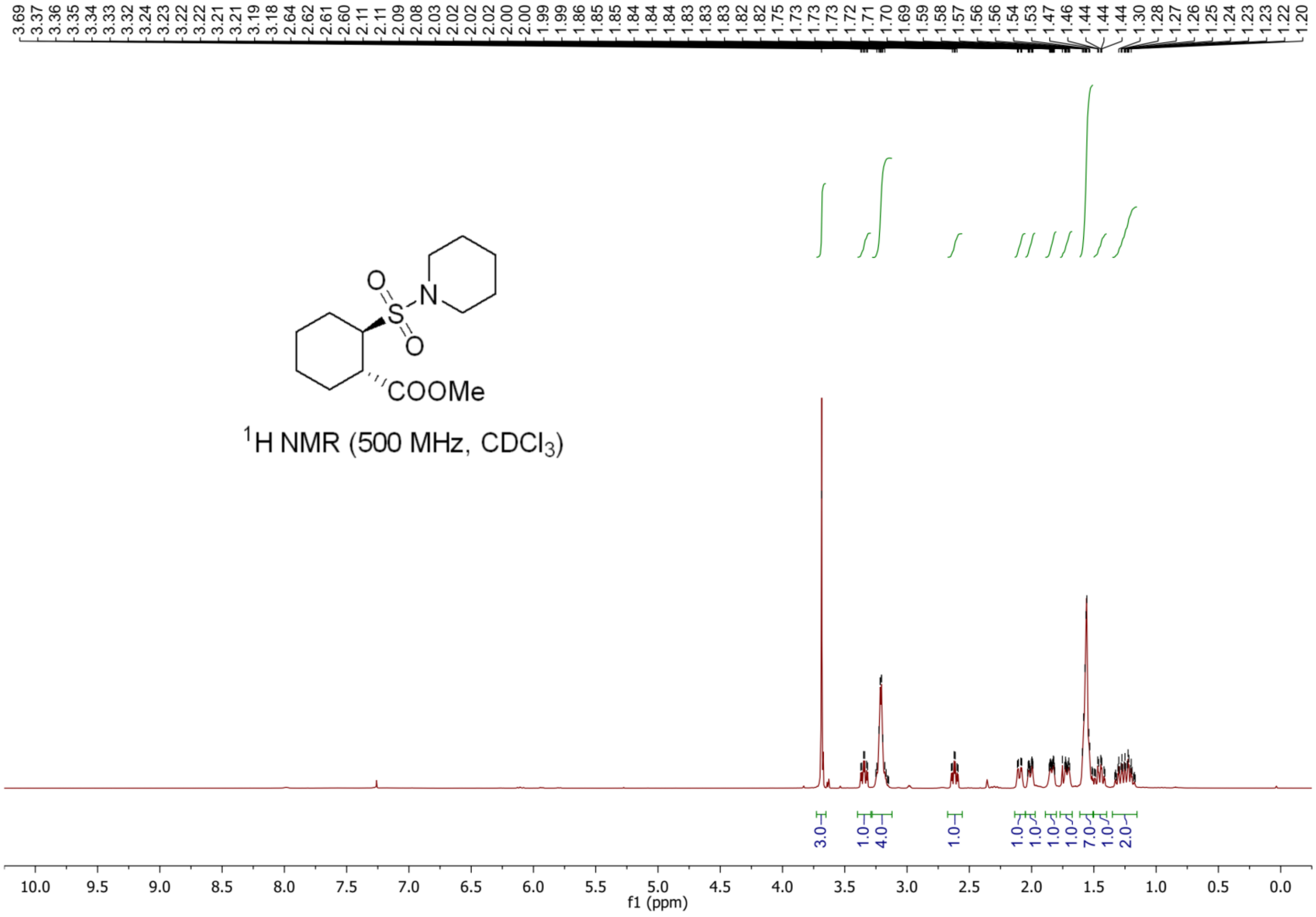

Go back to table of contents

Evaluation Only. Created with Aspose.Pdf. Copyright 2002-2014 Aspose Pty Ltd.

Methyl (1*S,2*R**)-2-(piperidin-1-ylsulfonyl)cyclohexane-1-carboxylate (2d)**

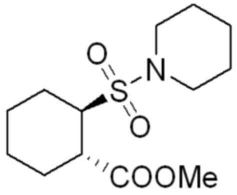

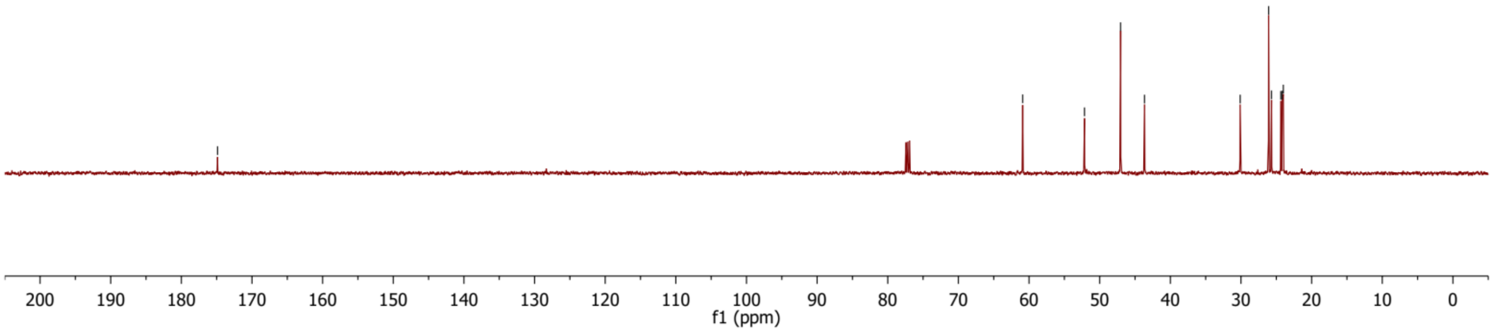

Go back to table of contents

Evaluation Only. Created with Aspose.Pdf. Copyright 2002-2014 Aspose Pty Ltd.

1-(((3*s*,5*s*,7*s*)-Adamantan-1-yl)sulfonyl)piperidine (2e)

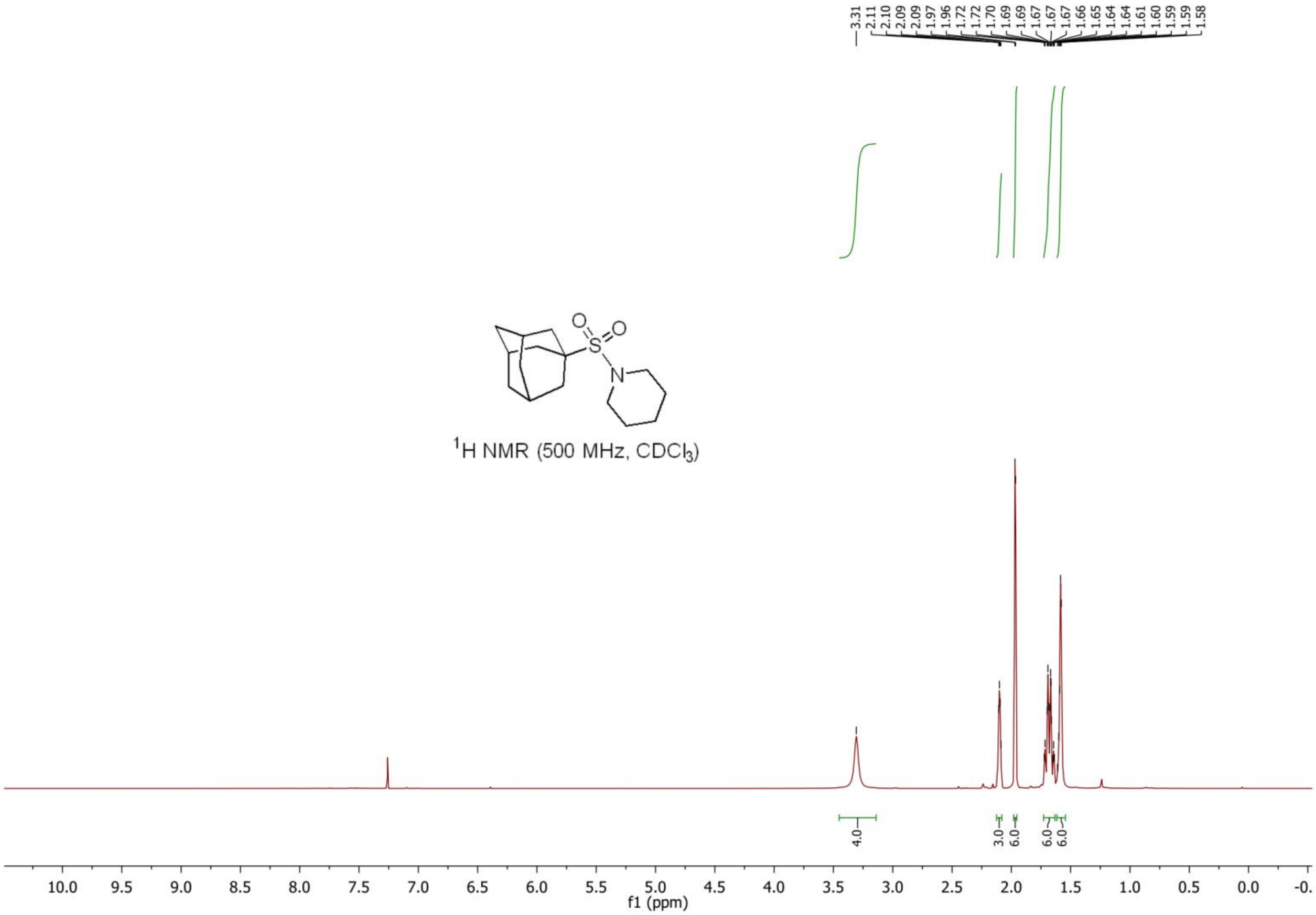

Go back to table of contents

Evaluation Only. Created with Aspose.Pdf. Copyright 2002-2014 Aspose Pty Ltd.

1-(((3*s*,5*s*,7*s*)-Adamantan-1-yl)sulfonyl)piperidine (2e)

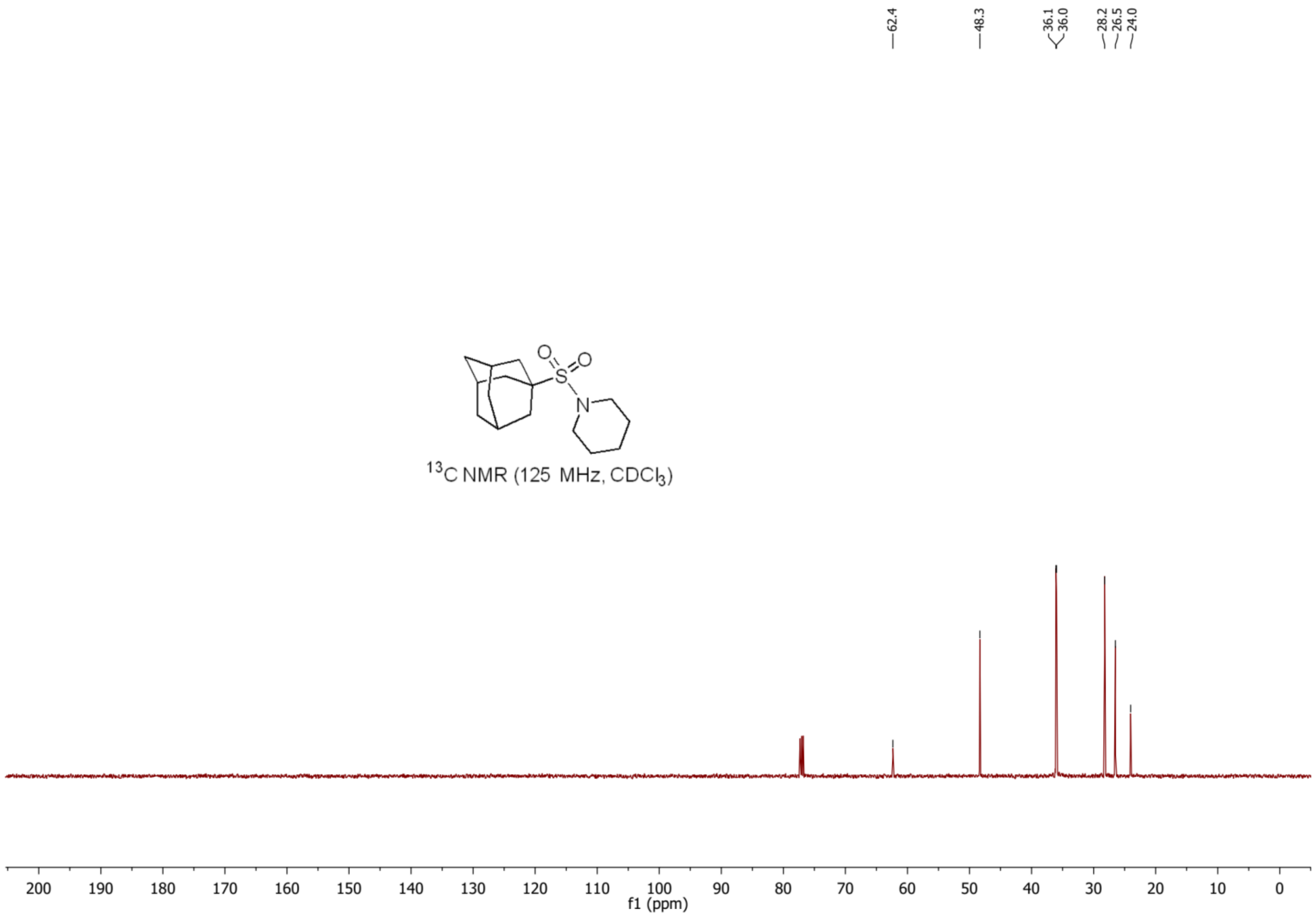

Go back to table of contents

Evaluation Only. Created with Aspose.Pdf. Copyright 2002-2014 Aspose Pty Ltd.

1-(*tert*-Pentylsulfonyl)piperidine (2f)

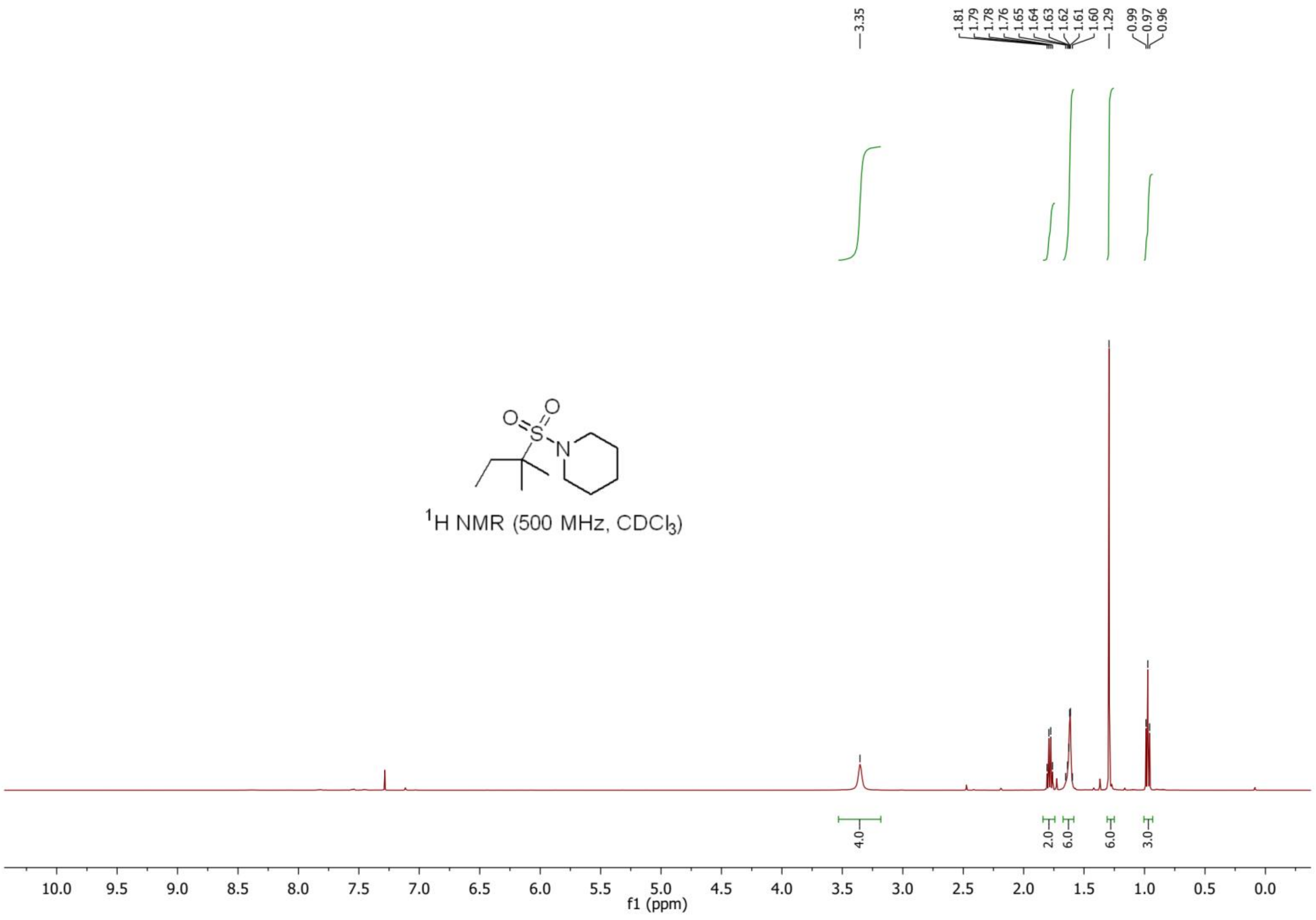

Go back to table of contents

Evaluation Only. Created with Aspose.Pdf. Copyright 2002-2014 Aspose Pty Ltd.

1-(*tert*-Pentylsulfonyl)piperidine (2f)

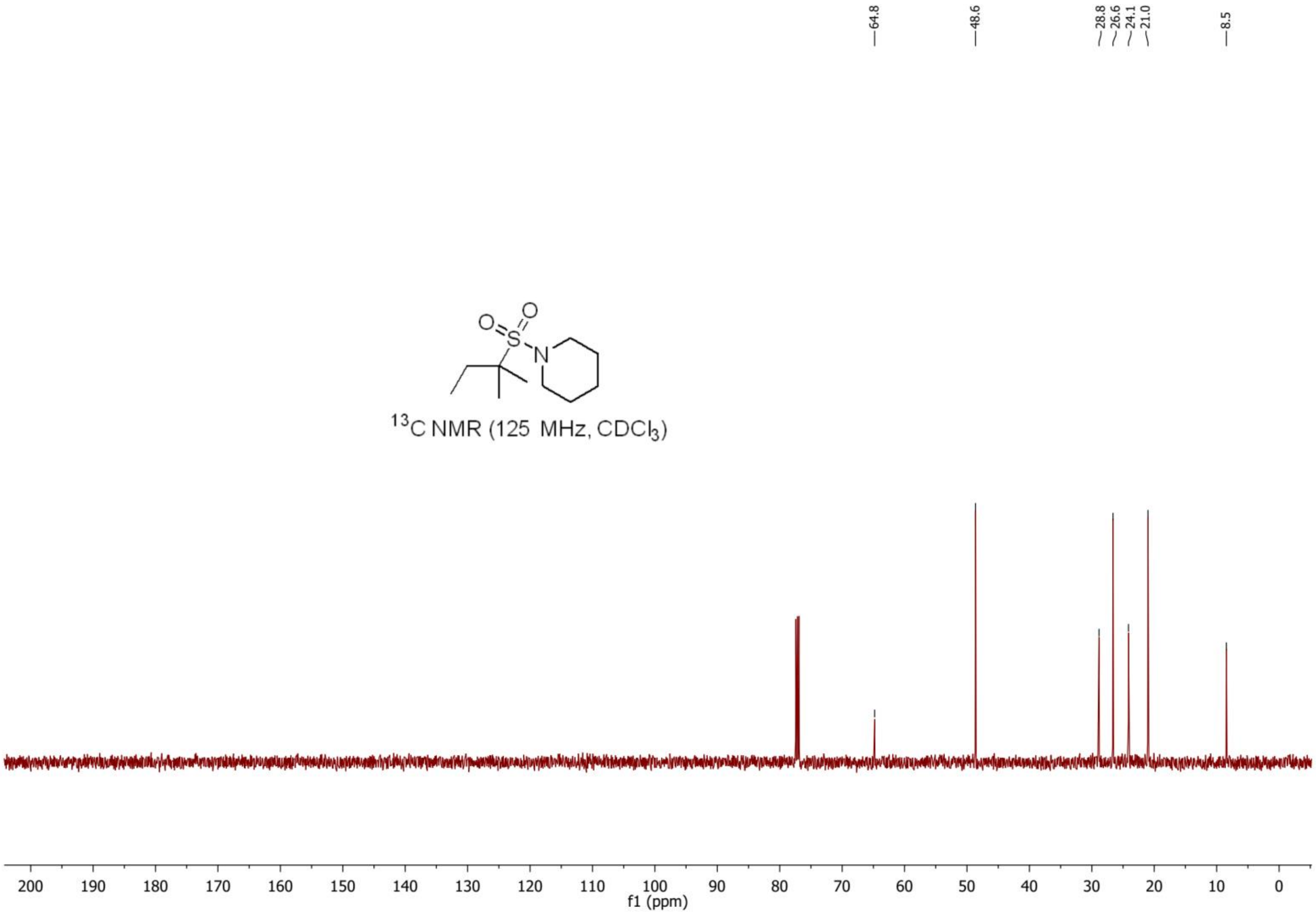

Go back to table of contents

Evaluation Only. Created with Aspose.Pdf. Copyright 2002-2014 Aspose Pty Ltd.

N-(*Tert*-butyl)-4-phenylbutane-1-sulfonamide (3a)

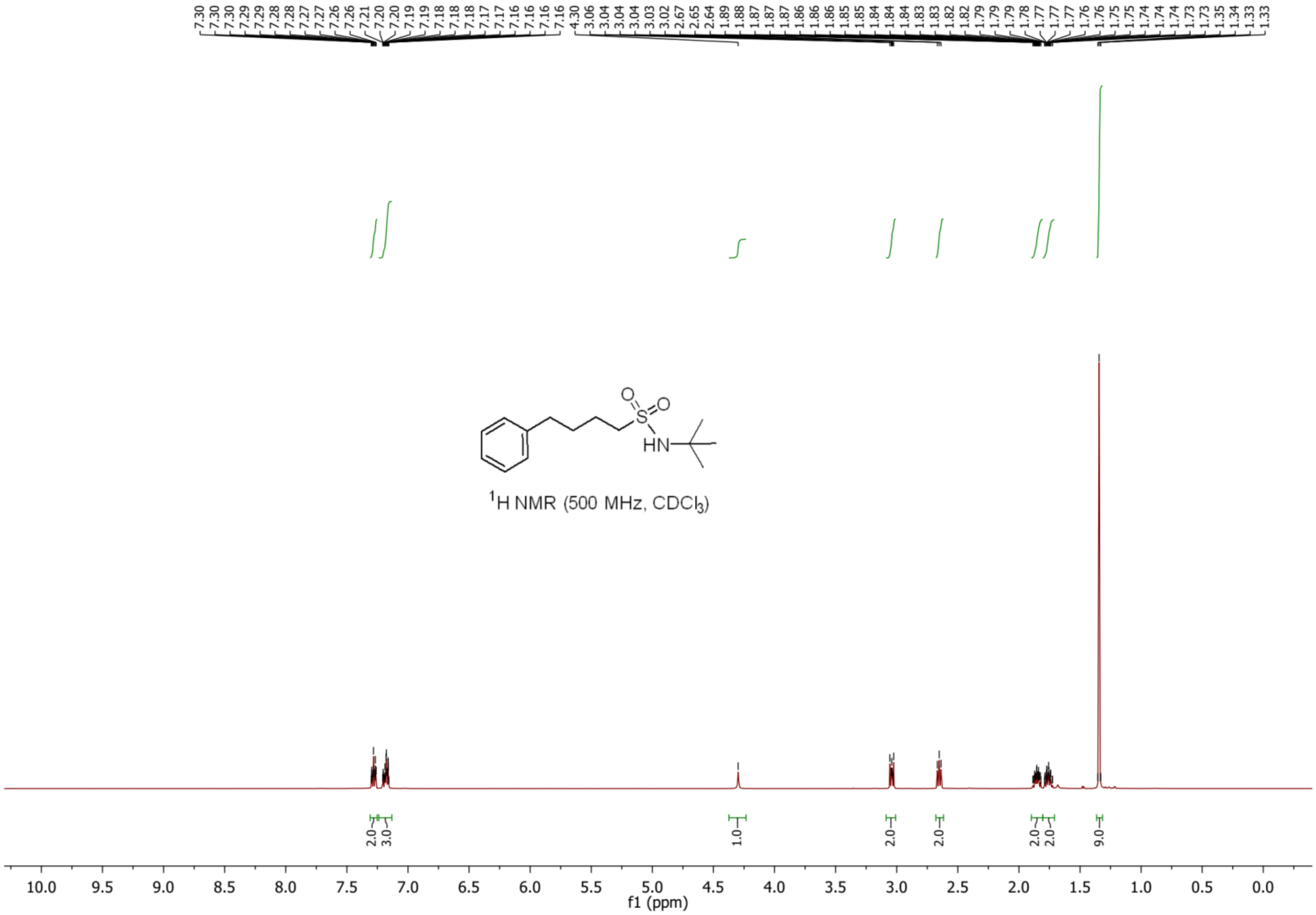

Go back to table of contents

Evaluation Only. Created with Aspose.Pdf. Copyright 2002-2014 Aspose Pty Ltd.

N-(*Tert*-butyl)-4-phenylbutane-1-sulfonamide (3a)

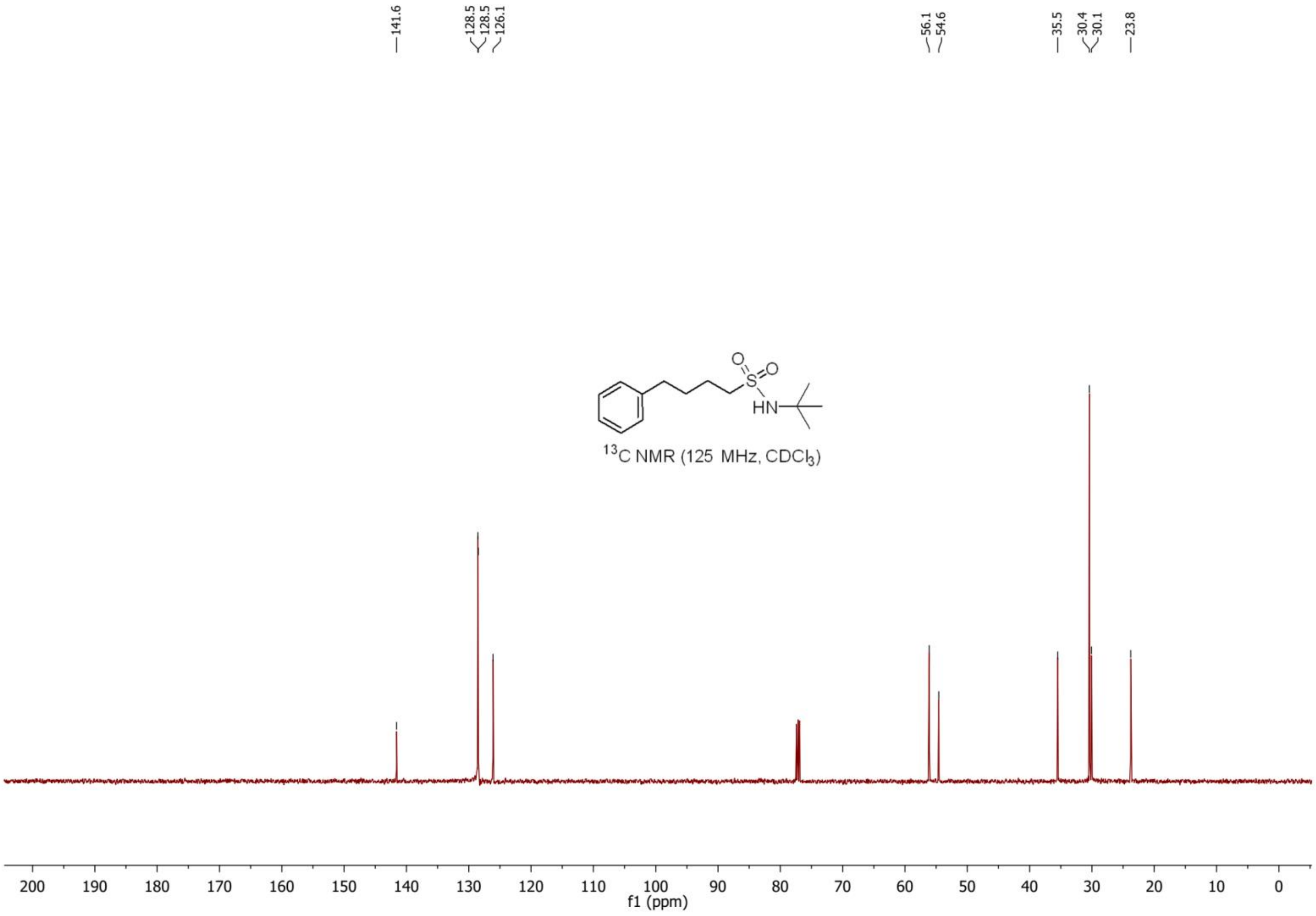

Go back to table of contents

Evaluation Only. Created with Aspose.Pdf. Copyright 2002-2014 Aspose Pty Ltd.

_N_-(_tert_-Butyl)tetrahydro-2_H_-pyran-4-sulfonamide (3b)

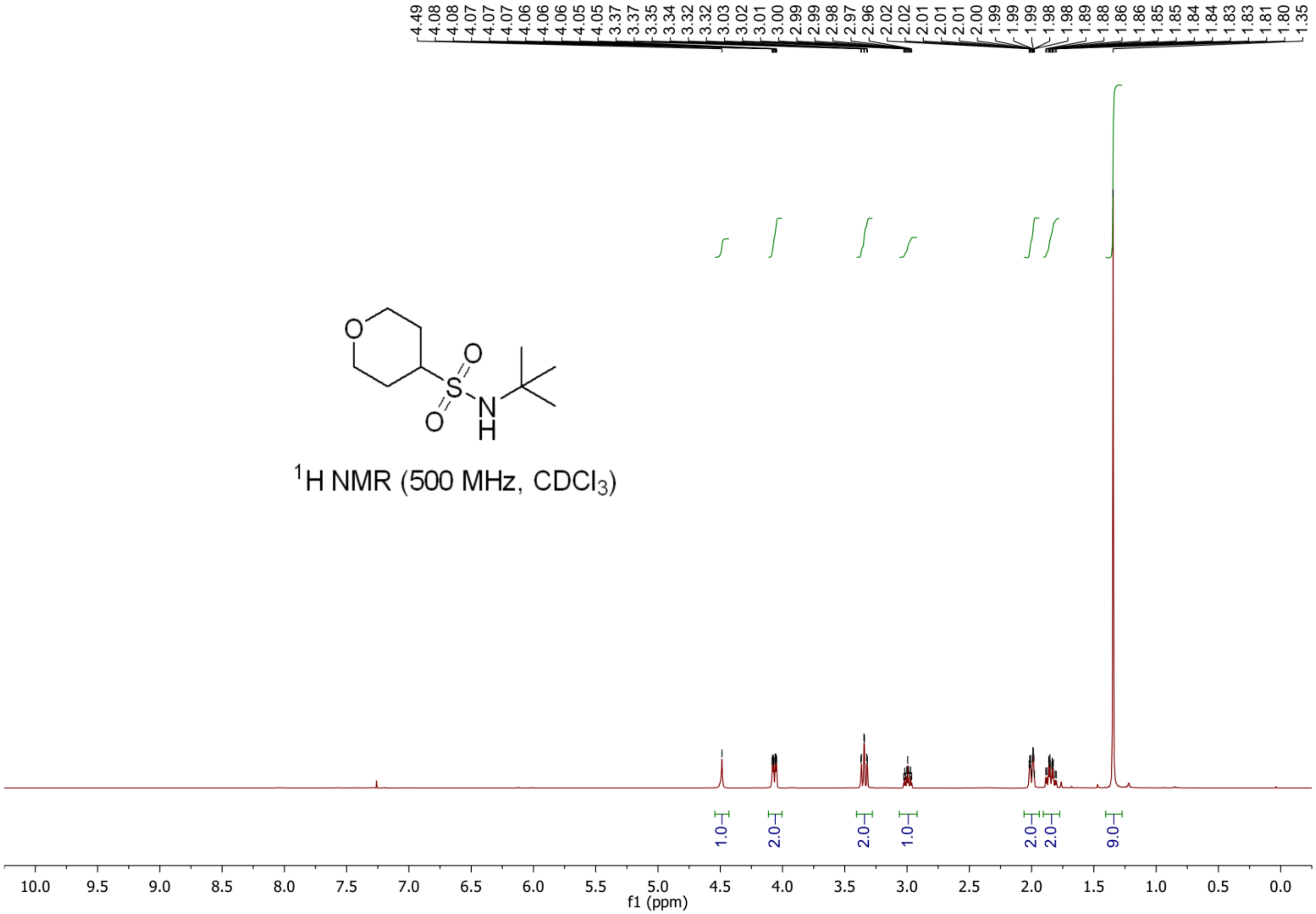

Go back to table of contents

Evaluation Only. Created with Aspose.Pdf. Copyright 2002-2014 Aspose Pty Ltd.

N-(*tert*-Butyl)tetrahydro-2*H*-pyran-4-sulfonamide (3b)

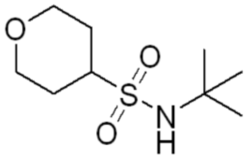

^{13}C NMR (125 MHz, $CDCl_3$)

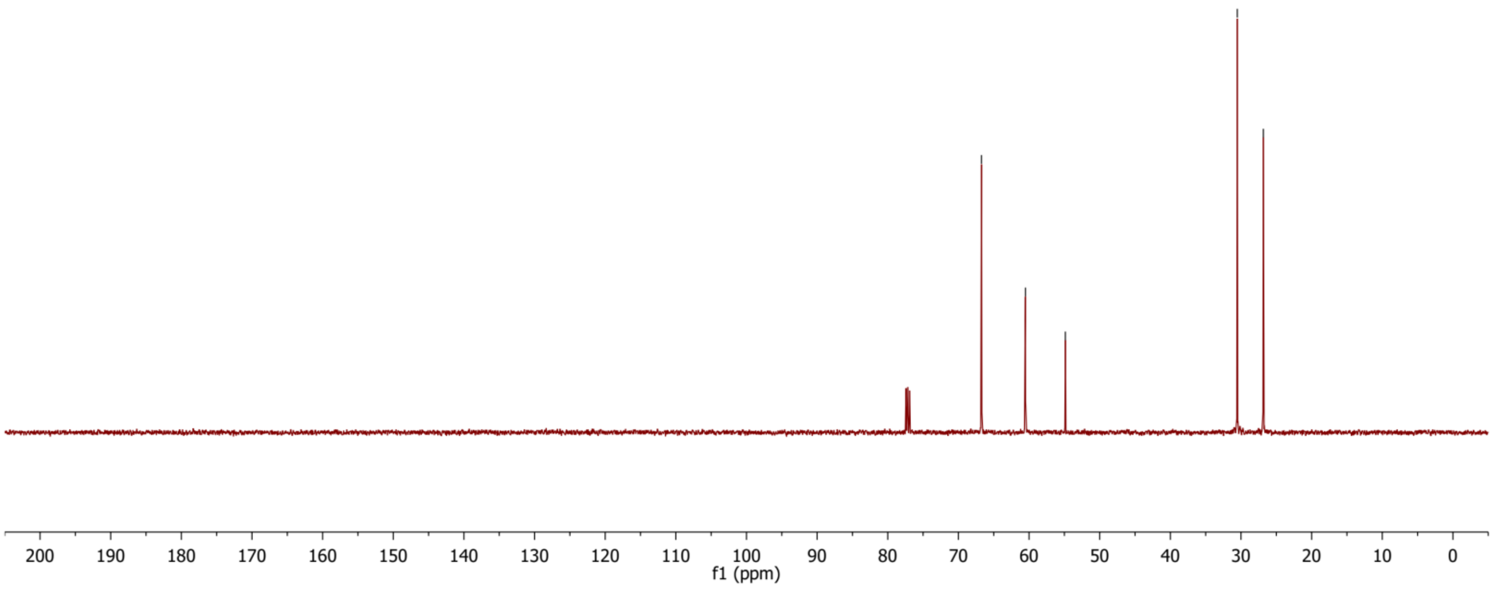

Evaluation Only. Created with Aspose.Pdf. Copyright 2002-2014 Aspose Pty Ltd.

tert-Butyl 4-(_N_-(_tert_-butyl)sulfamoyl)piperidine-1-carboxylate (3c)

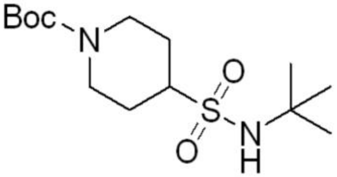

^{1}H NMR (500 MHz, $CDCl_3$)

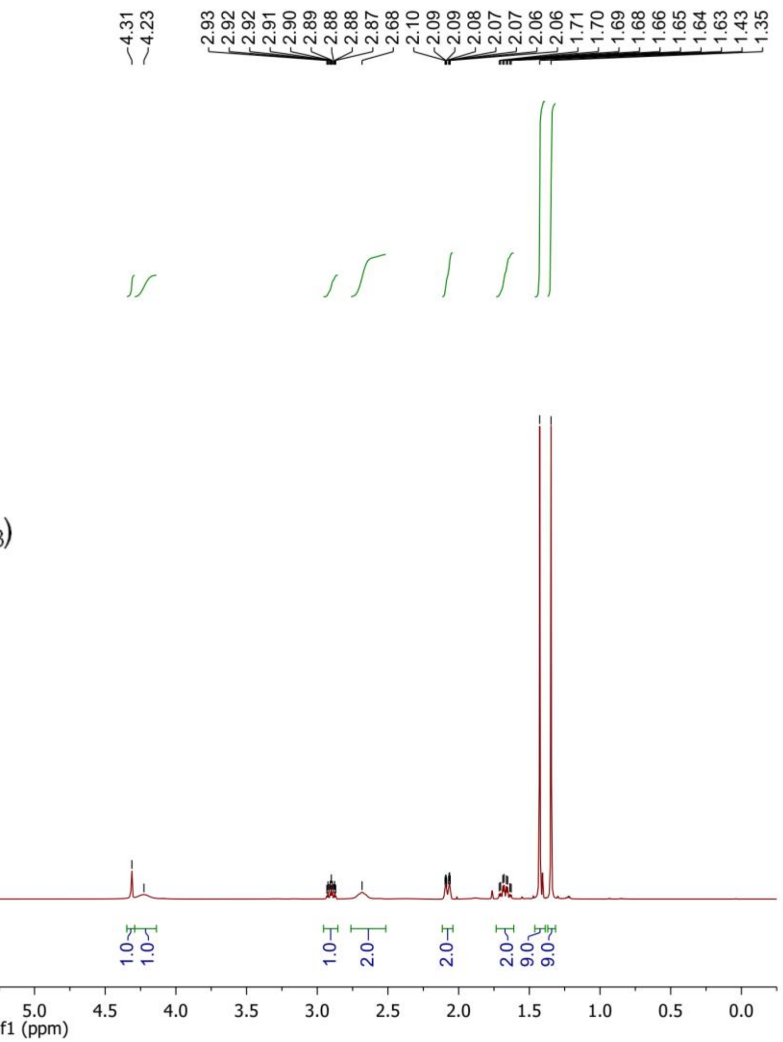

Go back to table of contents

Evaluation Only. Created with Aspose.Pdf. Copyright 2002-2014 Aspose Pty Ltd.

tert-Butyl 4-(*N*-(*tert*-butyl)sulfamoyl)piperidine-1-carboxylate (3c)

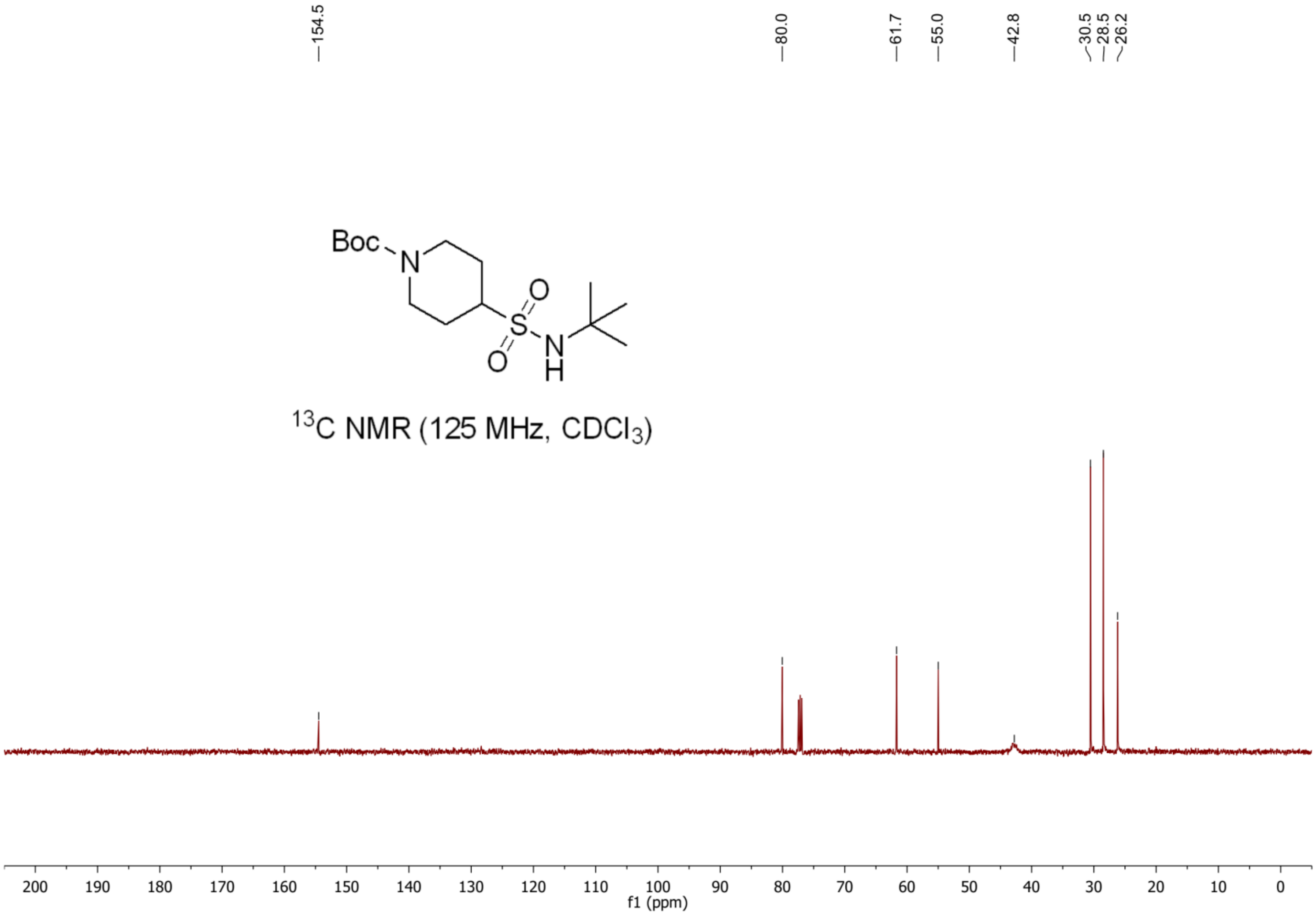

Go back to table of contents

Evaluation Only. Created with Aspose.Pdf. Copyright 2002-2014 Aspose Pty Ltd.

N-(*tert*-Butyl)-2-methylpropane-2-sulfonamide (3d)

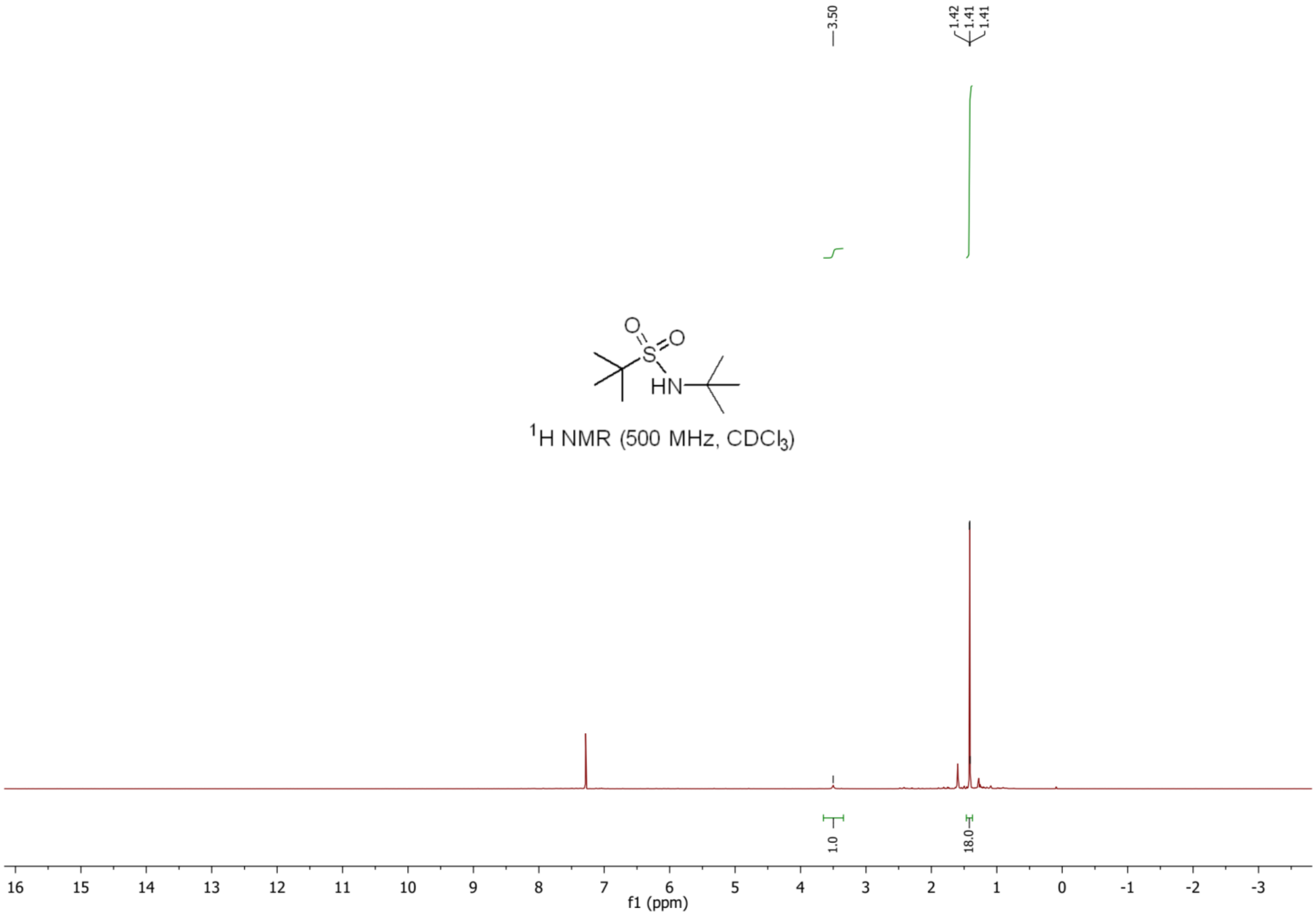

Go back to table of contents

Evaluation Only. Created with Aspose.Pdf. Copyright 2002-2014 Aspose Pty Ltd.

N-(*tert*-Butyl)-2-methylpropane-2-sulfonamide (3d)

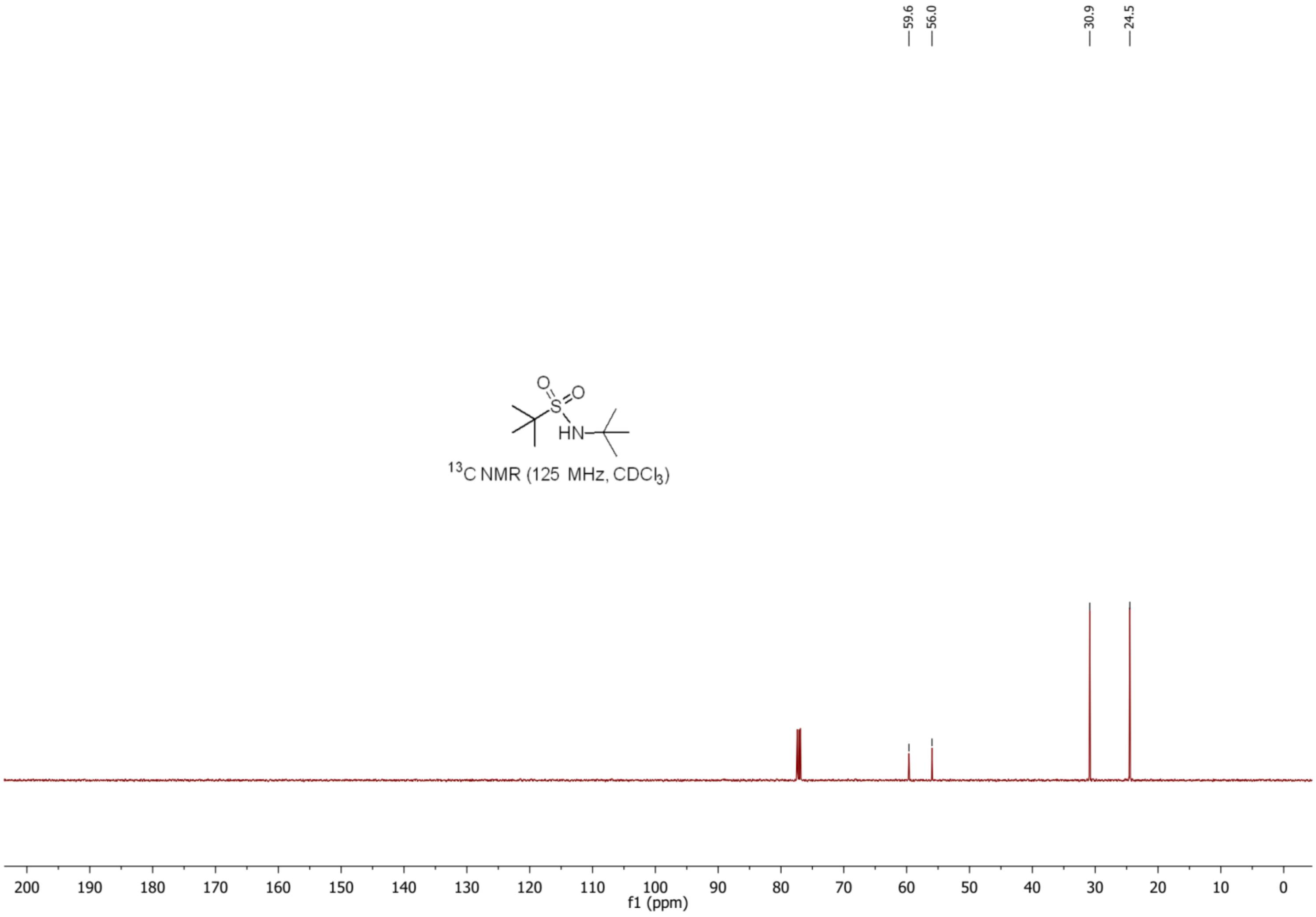

Go back to table of contents

Evaluation Only. Created with Aspose.Pdf. Copyright 2002-2014 Aspose Pty Ltd.

(3*s*,5*s*,7*s*)-N-(*tert*-Butyl)adamantane-1-sulfonamide (3e)

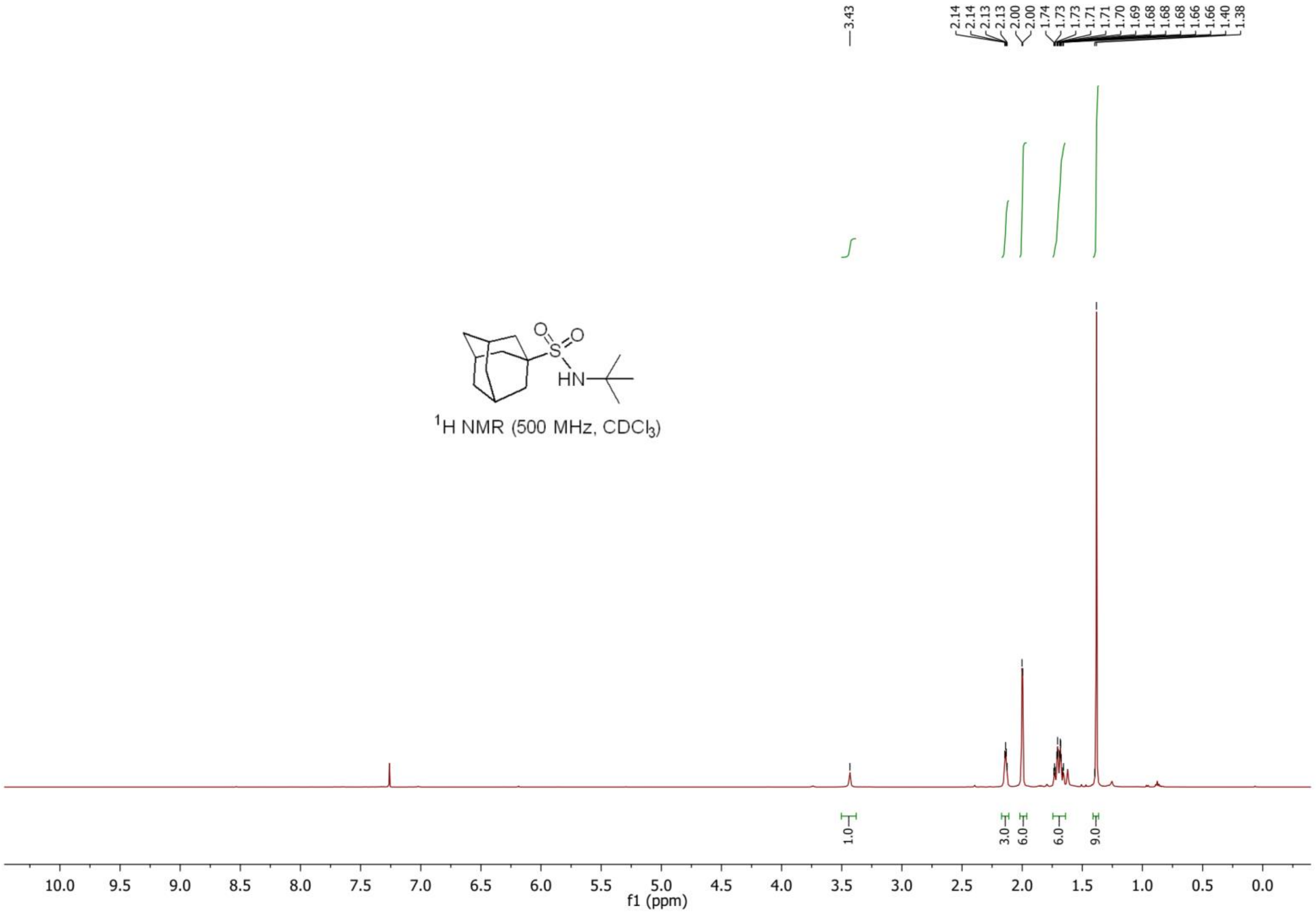

Go back to table of contents

Evaluation Only. Created with Aspose.Pdf. Copyright 2002-2014 Aspose Pty Ltd.

(3*s*,5*s*,7*s*)-N-(*tert*-Butyl)adamantane-1-sulfonamide (3e)

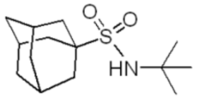

^{13}C NMR (125 MHz, $CDCl_3$)

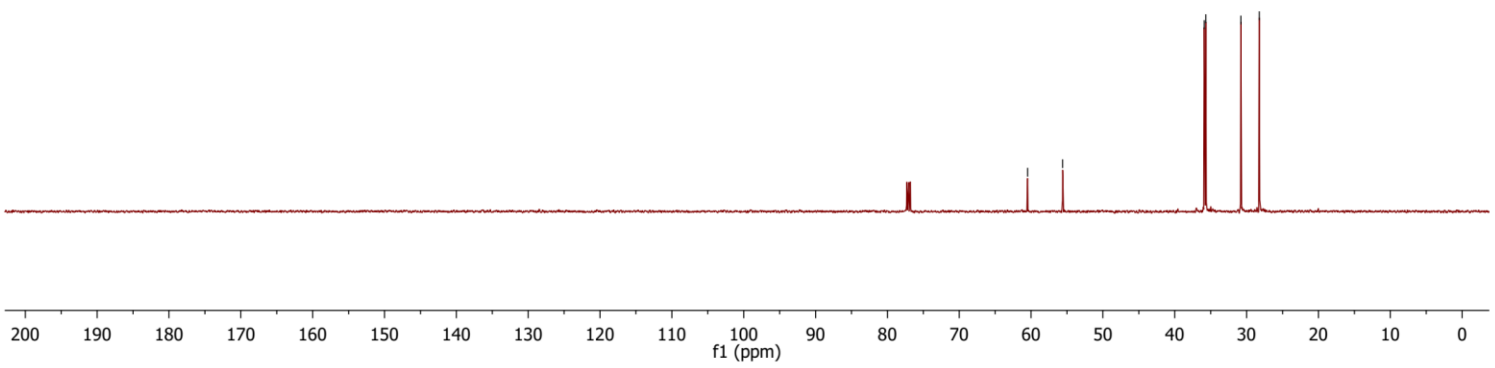

Go back to table of contents

Evaluation Only. Created with Aspose.Pdf. Copyright 2002-2014 Aspose Pty Ltd.

N-(*tert*-Butyl)-5-(2,5-dimethylphenoxy)-2-methylpentane-2-sulfonamide (3f)

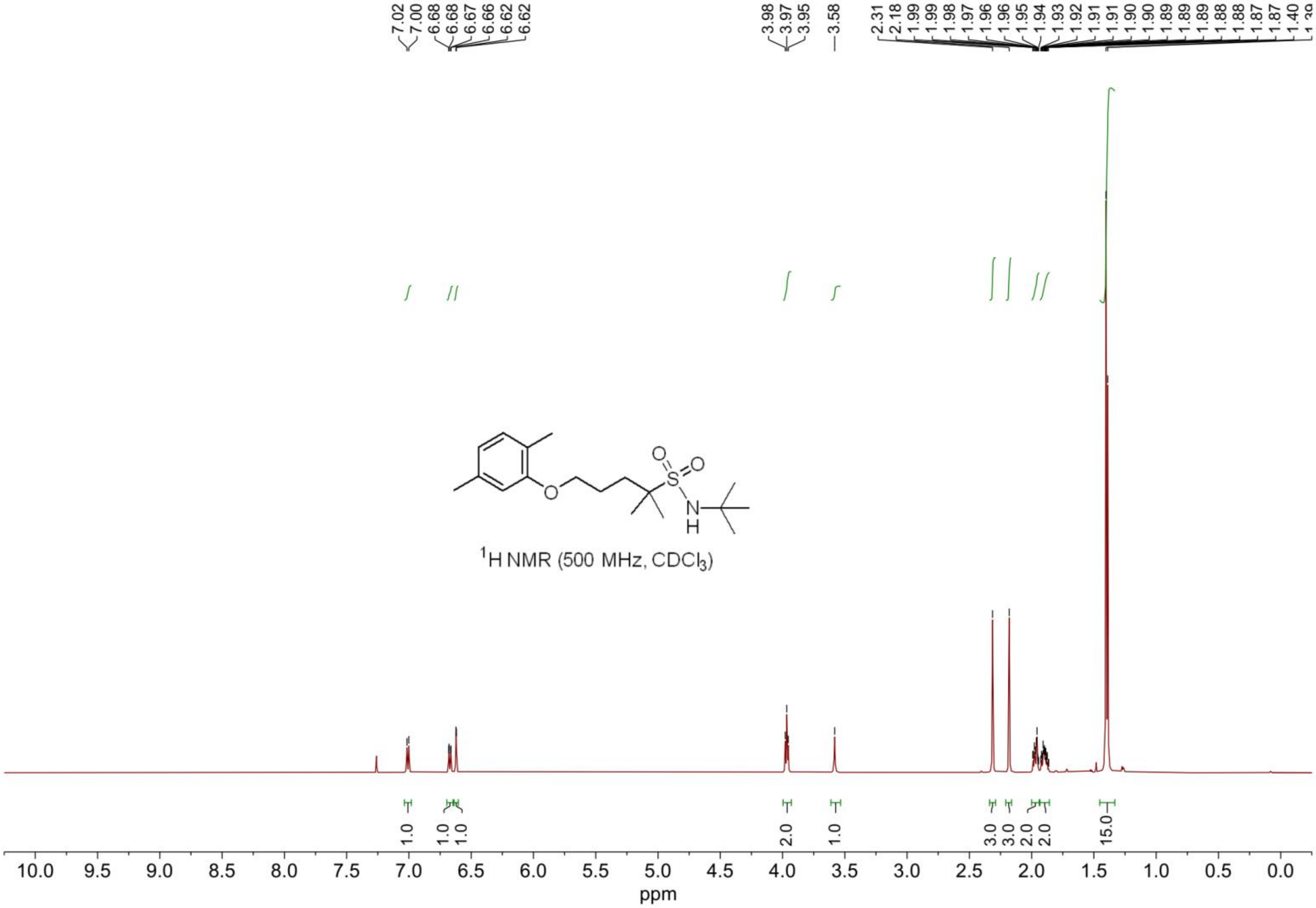

Go back to table of contents

Evaluation Only. Created with Aspose.Pdf. Copyright 2002-2014 Aspose Pty Ltd.

N-(*tert*-Butyl)-5-(2,5-dimethylphenoxy)-2-methylpentane-2-sulfonamide (3f)

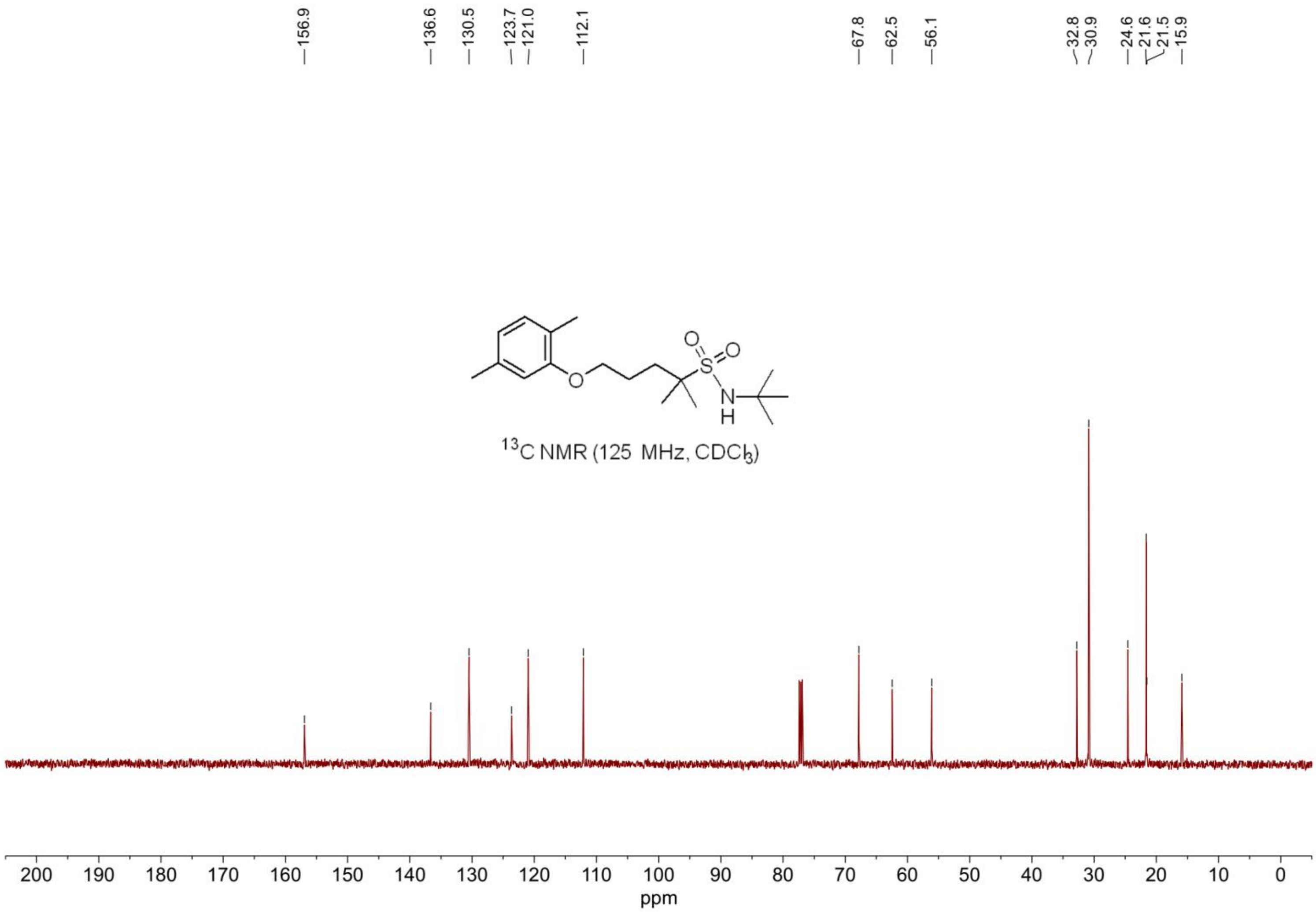

Go back to table of contents

Evaluation Only. Created with Aspose.Pdf. Copyright 2002-2014 Aspose Pty Ltd.

N-Benzylpentadecane-1-sulfonamide (4a)

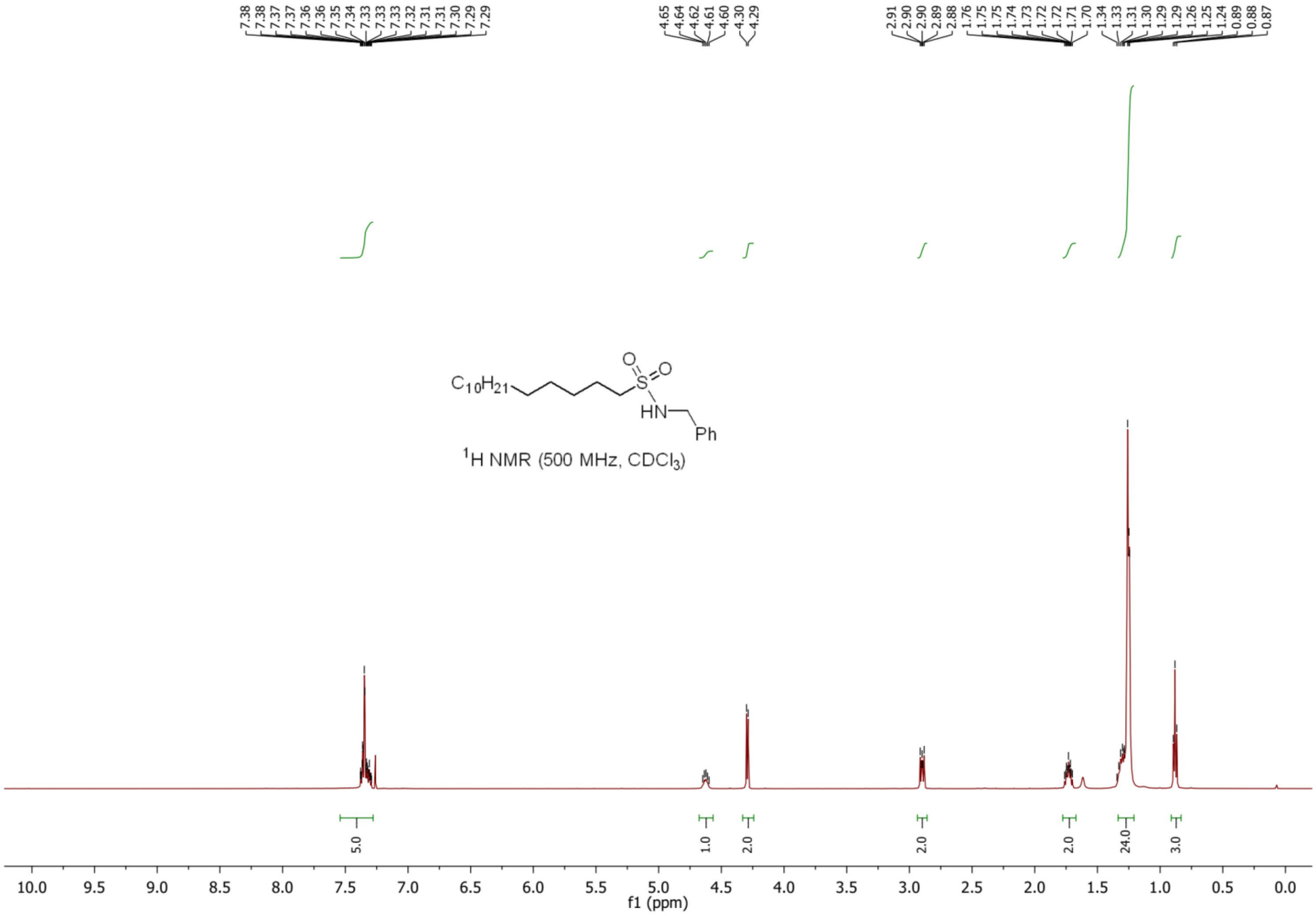

Go back to table of contents

Evaluation Only. Created with Aspose.Pdf. Copyright 2002-2014 Aspose Pty Ltd.

N-Benzylpentadecane-1-sulfonamide (4a)

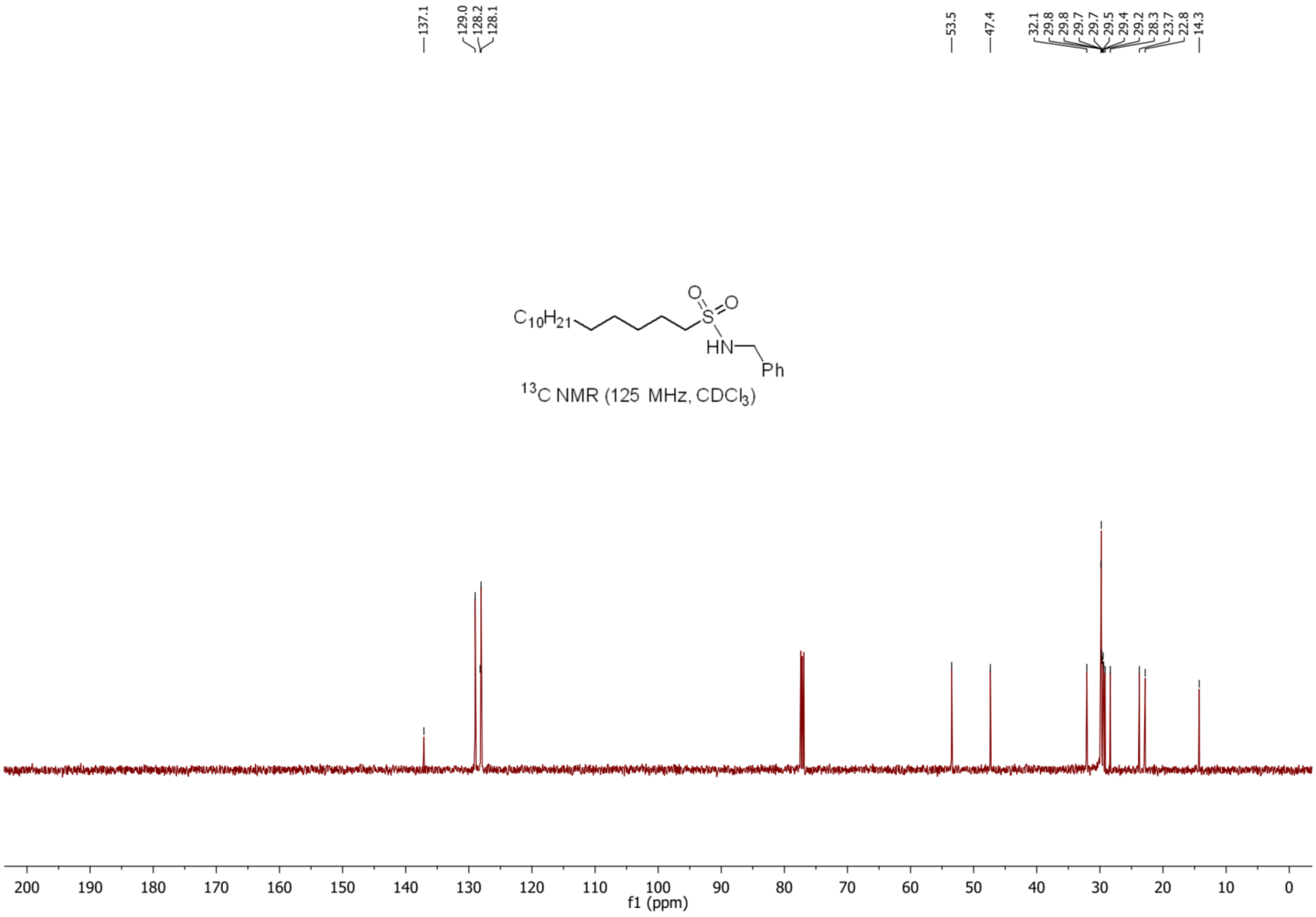

Go back to table of contents

Evaluation Only. Created with Aspose.Pdf. Copyright 2002-2014 Aspose Pty Ltd.

N-Benzyltetrahydro-2H-pyran-4-sulfonamide (4b)

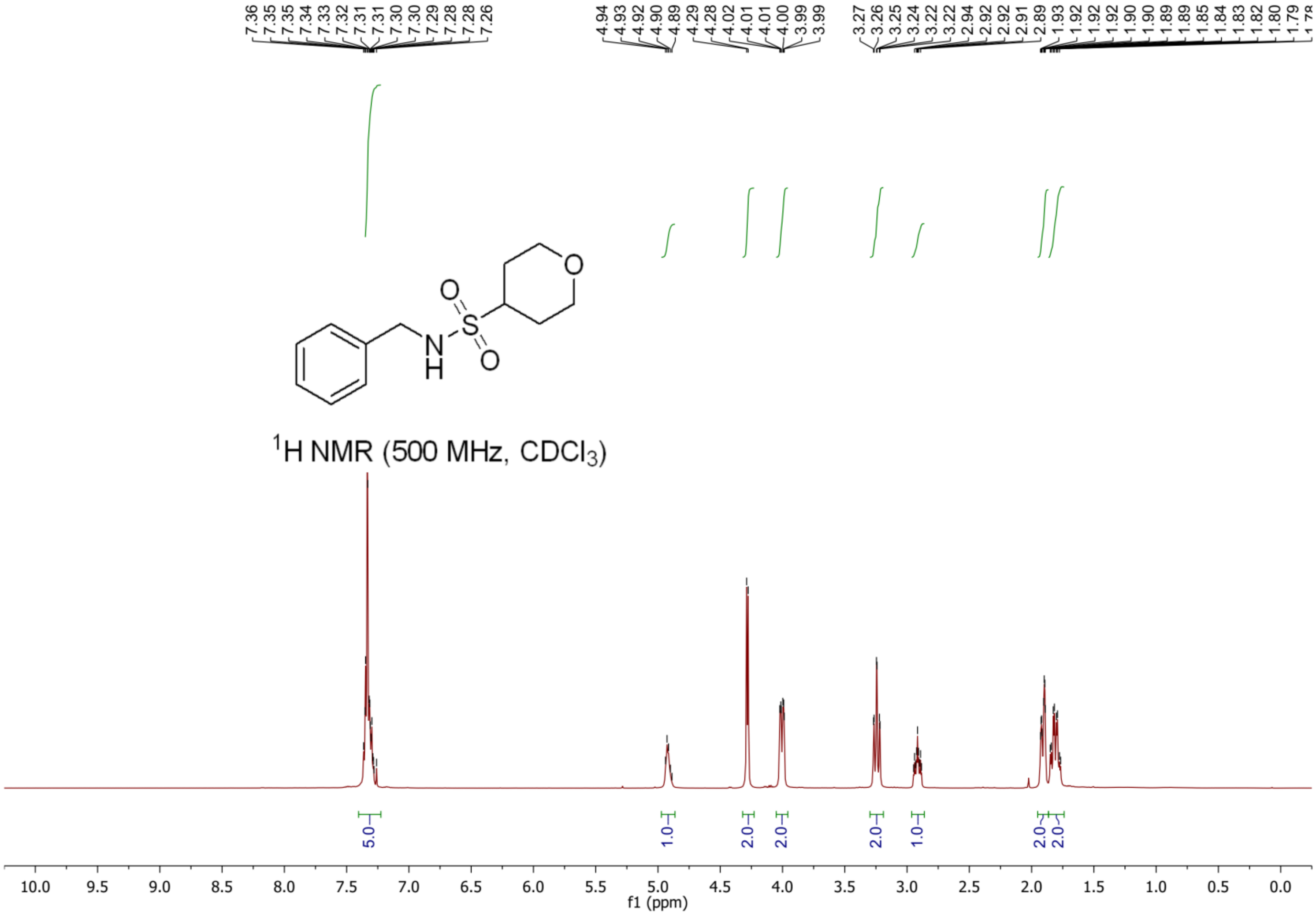

Go back to table of contents

Evaluation Only. Created with Aspose.Pdf. Copyright 2002-2014 Aspose Pty Ltd.

N-Benzyltetrahydro-2*H*-pyran-4-sulfonamide (4b)

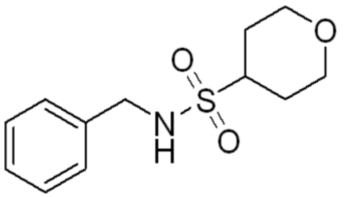

^{13}C NMR (125 MHz, CDCl$_3$)

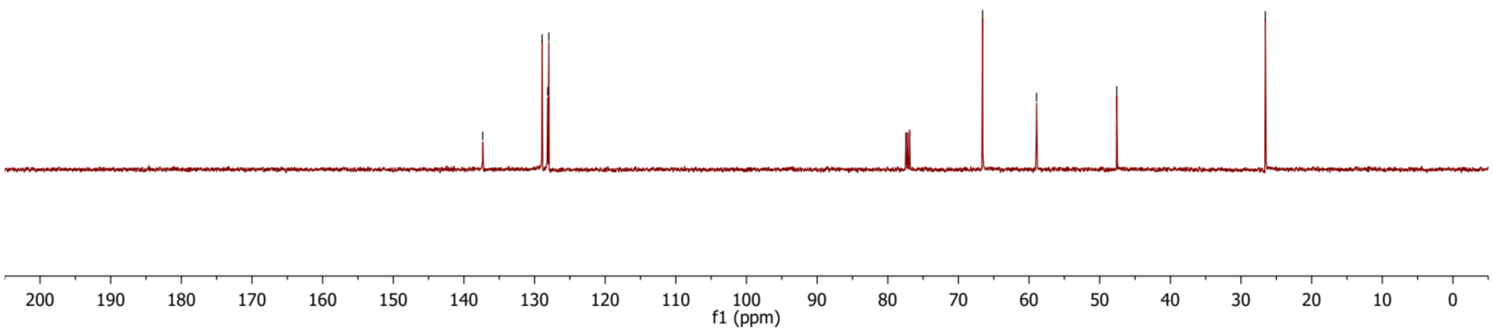

Go back to table of contents

Evaluation Only. Created with Aspose.Pdf. Copyright 2002-2014 Aspose Pty Ltd.

N-Benzyl-2-methylpropane-2-sulfonamide (4c)

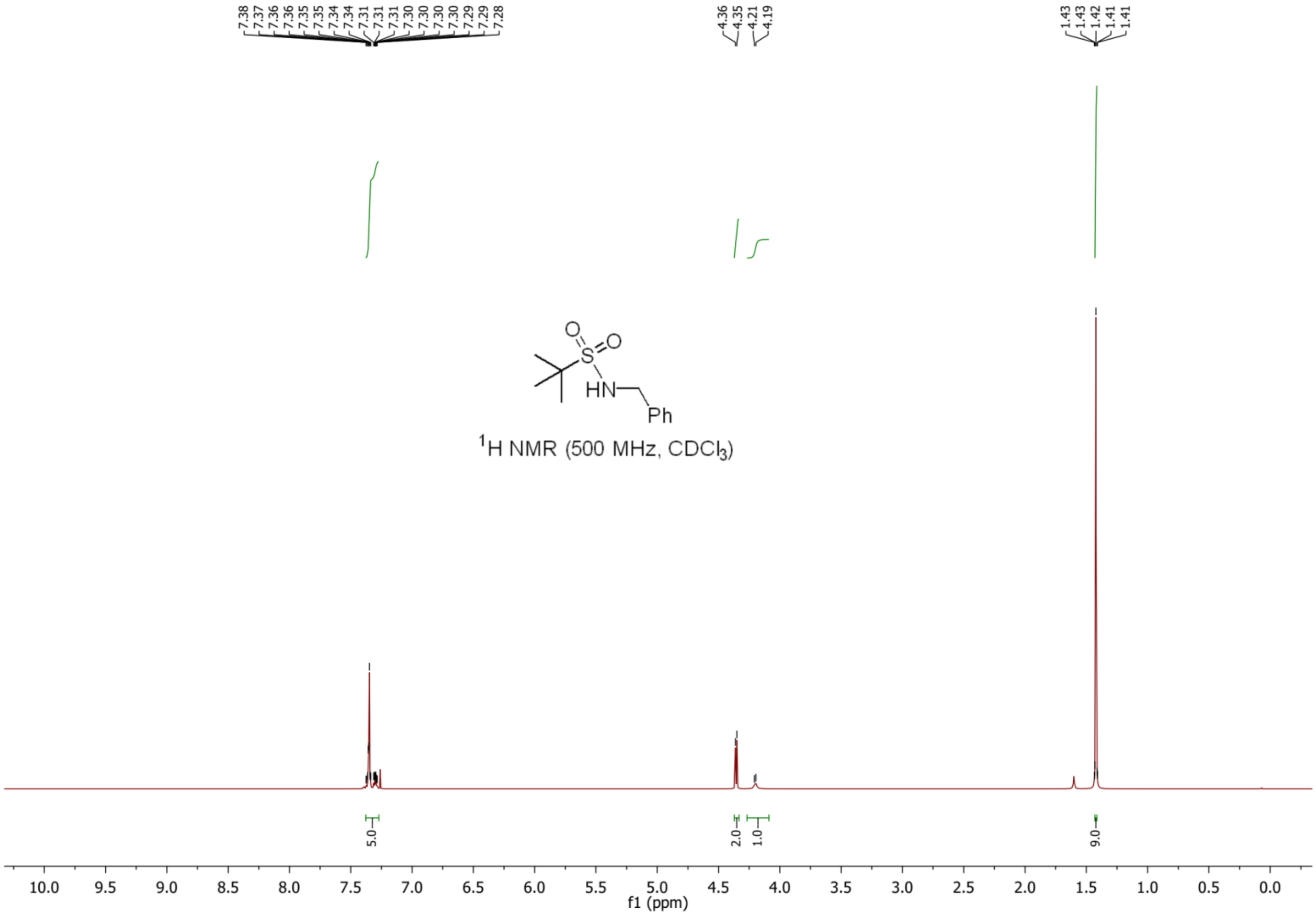

Go back to table of contents

Evaluation Only. Created with Aspose.Pdf. Copyright 2002-2014 Aspose Pty Ltd.

N-Benzyl-2-methylpropane-2-sulfonamide (4c)

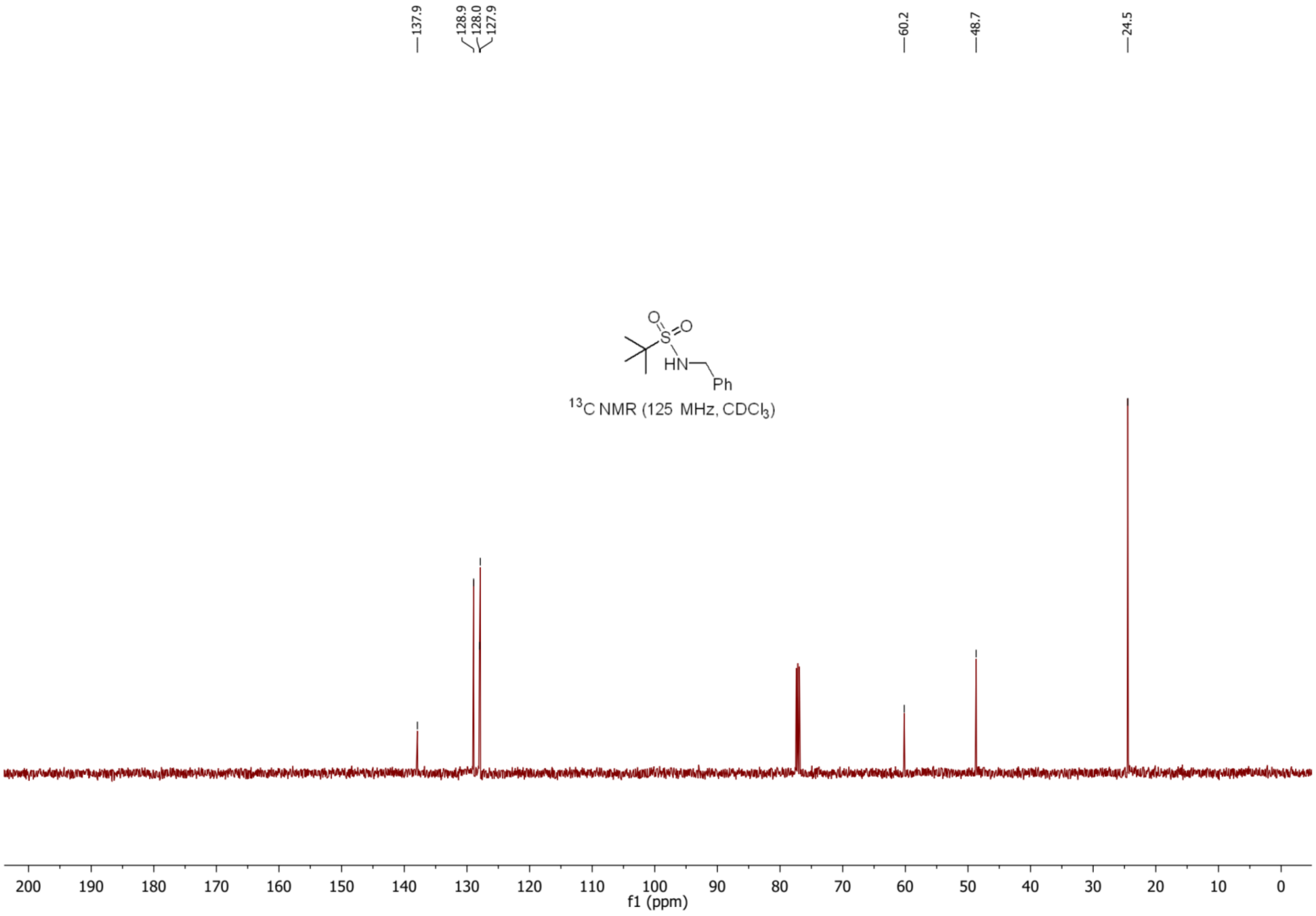

Go back to table of contents

Evaluation Only. Created with Aspose.Pdf. Copyright 2002-2014 Aspose Pty Ltd.

N-Benzyladamantane-1-sulfonamide (4d)

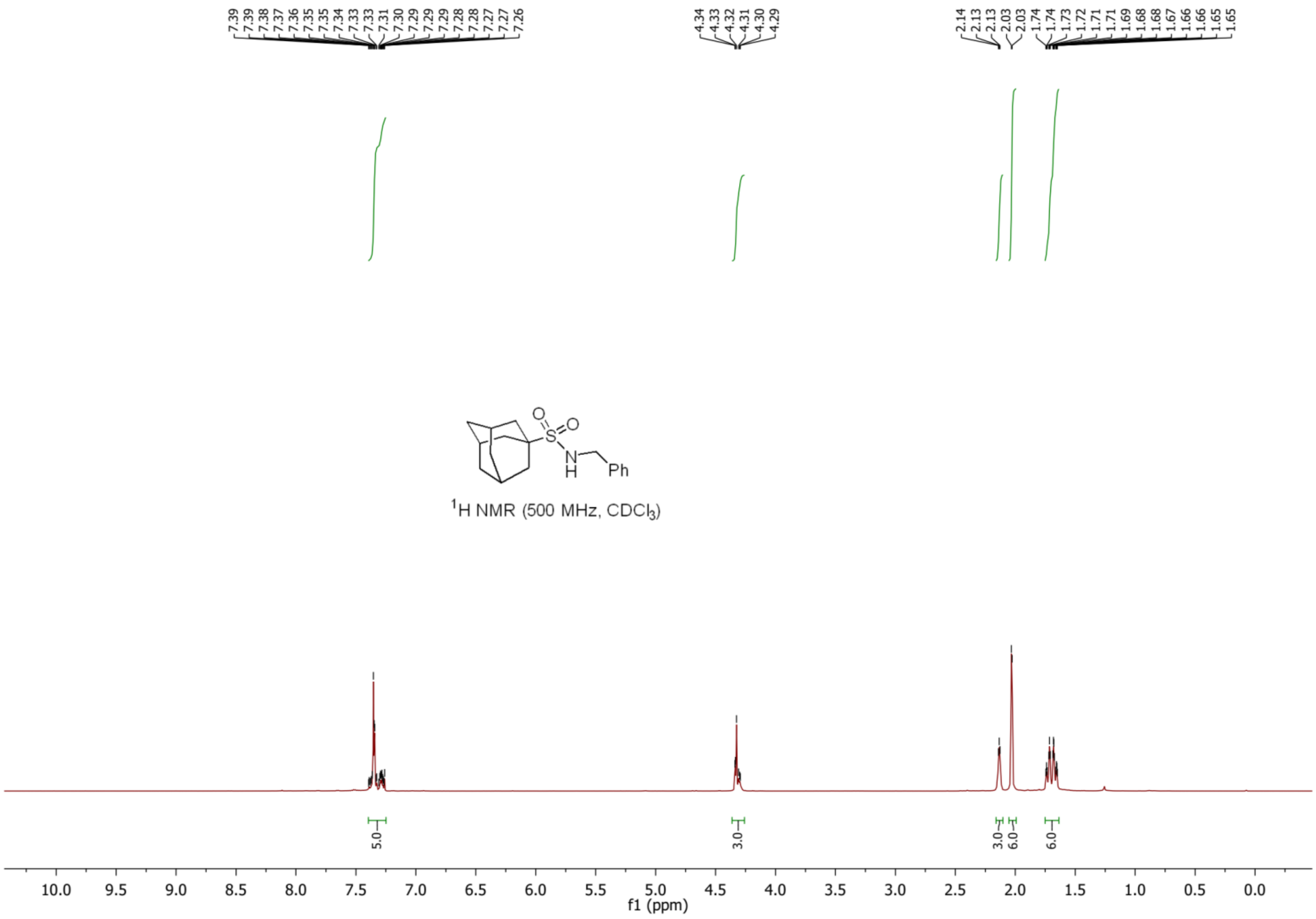

Go back to table of contents

Evaluation Only. Created with Aspose.Pdf. Copyright 2002-2014 Aspose Pty Ltd.

N-Benzyladamantane-1-sulfonamide (4d)

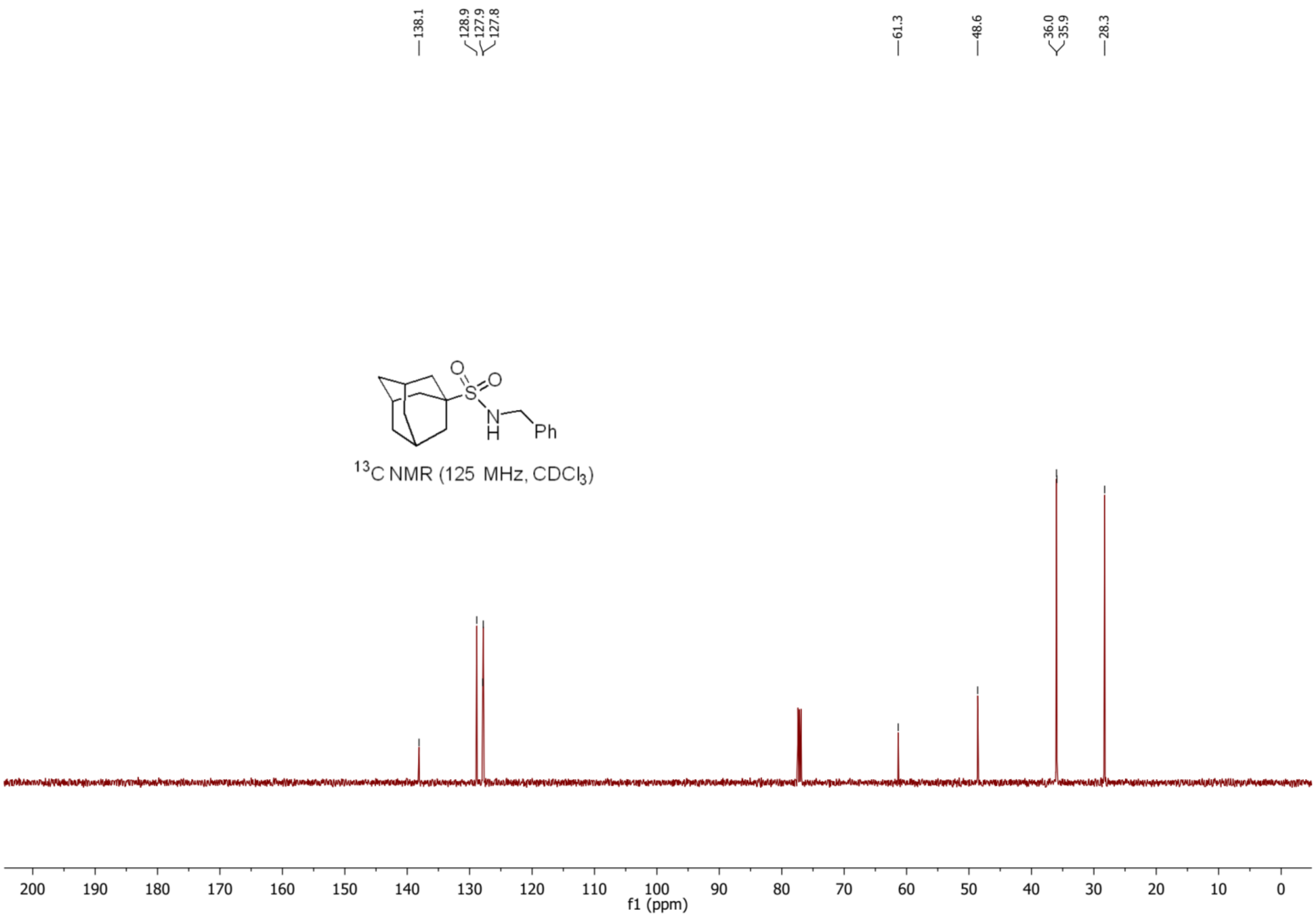

Go back to table of contents

Evaluation Only. Created with Aspose.Pdf. Copyright 2002-2014 Aspose Pty Ltd.

N-(4-Fluorophenethyl)-4-oxo-4-phenylbutane-1-sulfonamide (5a)

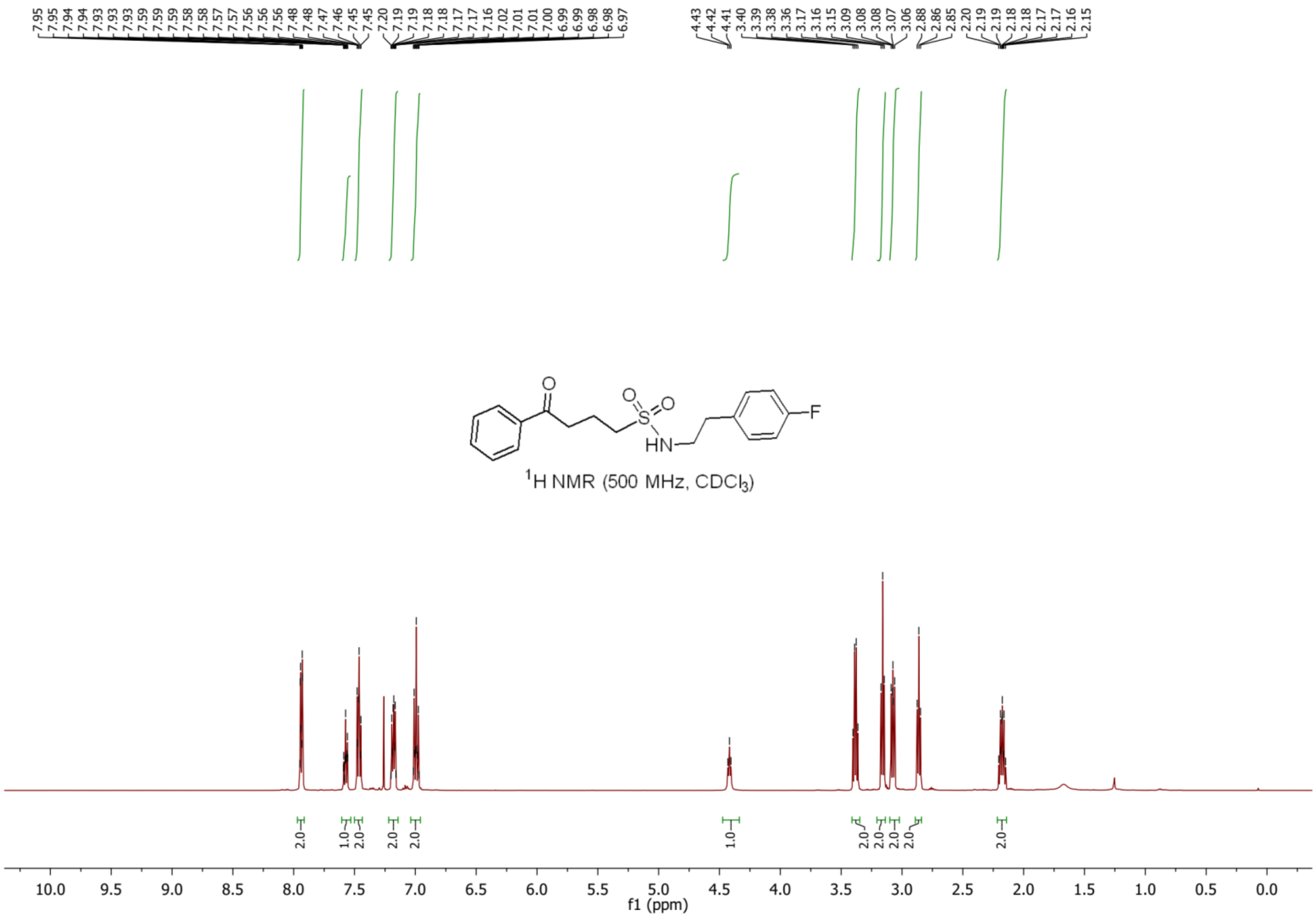

Go back to table of contents

Evaluation Only. Created with Aspose.Pdf. Copyright 2002-2014 Aspose Pty Ltd.

N-(4-Fluorophenethyl)-4-oxo-4-phenylbutane-1-sulfonamide (5a)

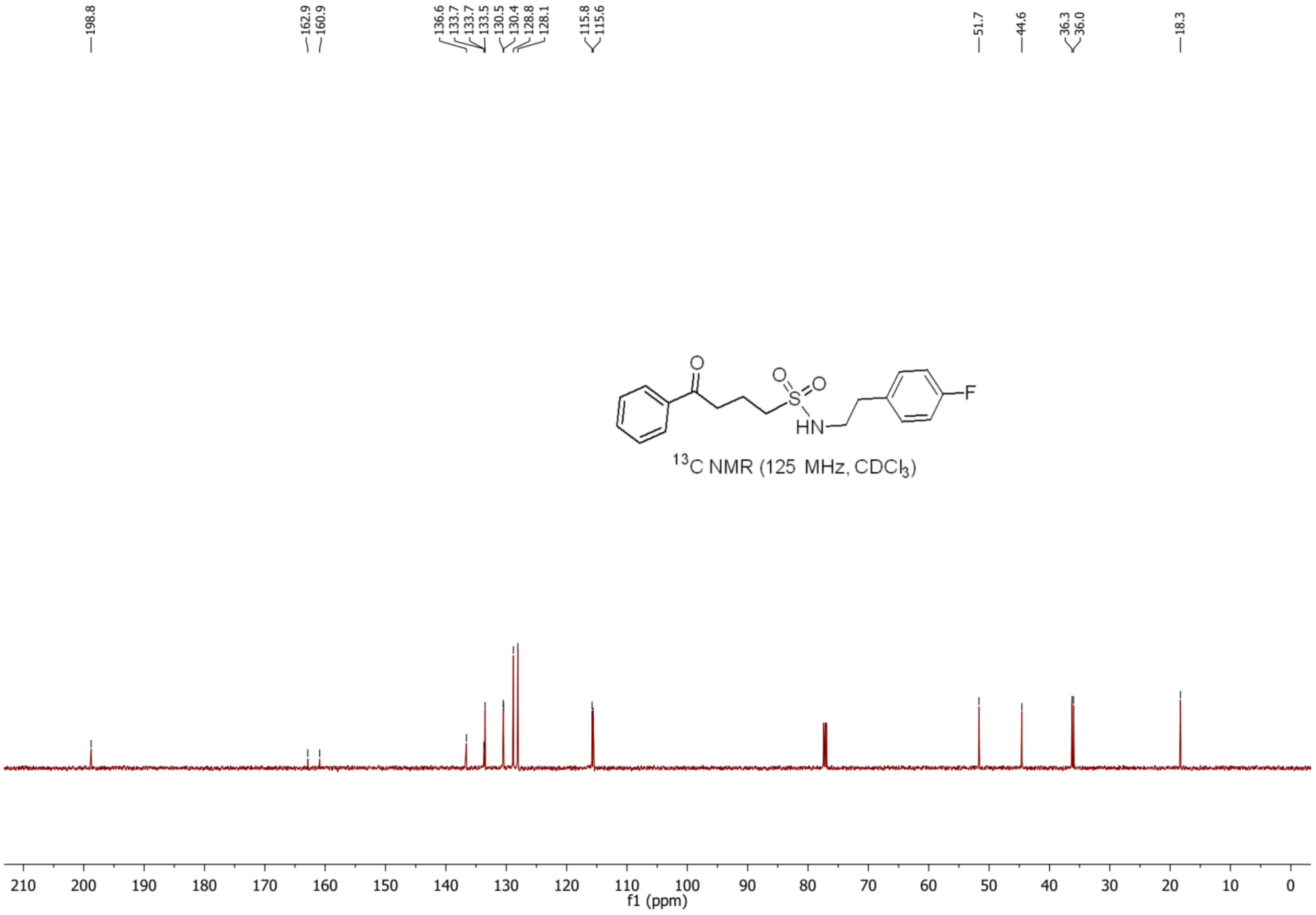

Go back to table of contents

Evaluation Only. Created with Aspose.Pdf. Copyright 2002-2014 Aspose Pty Ltd.

N-(4-Fluorophenethyl)heptane-4-sulfonamide (5b)

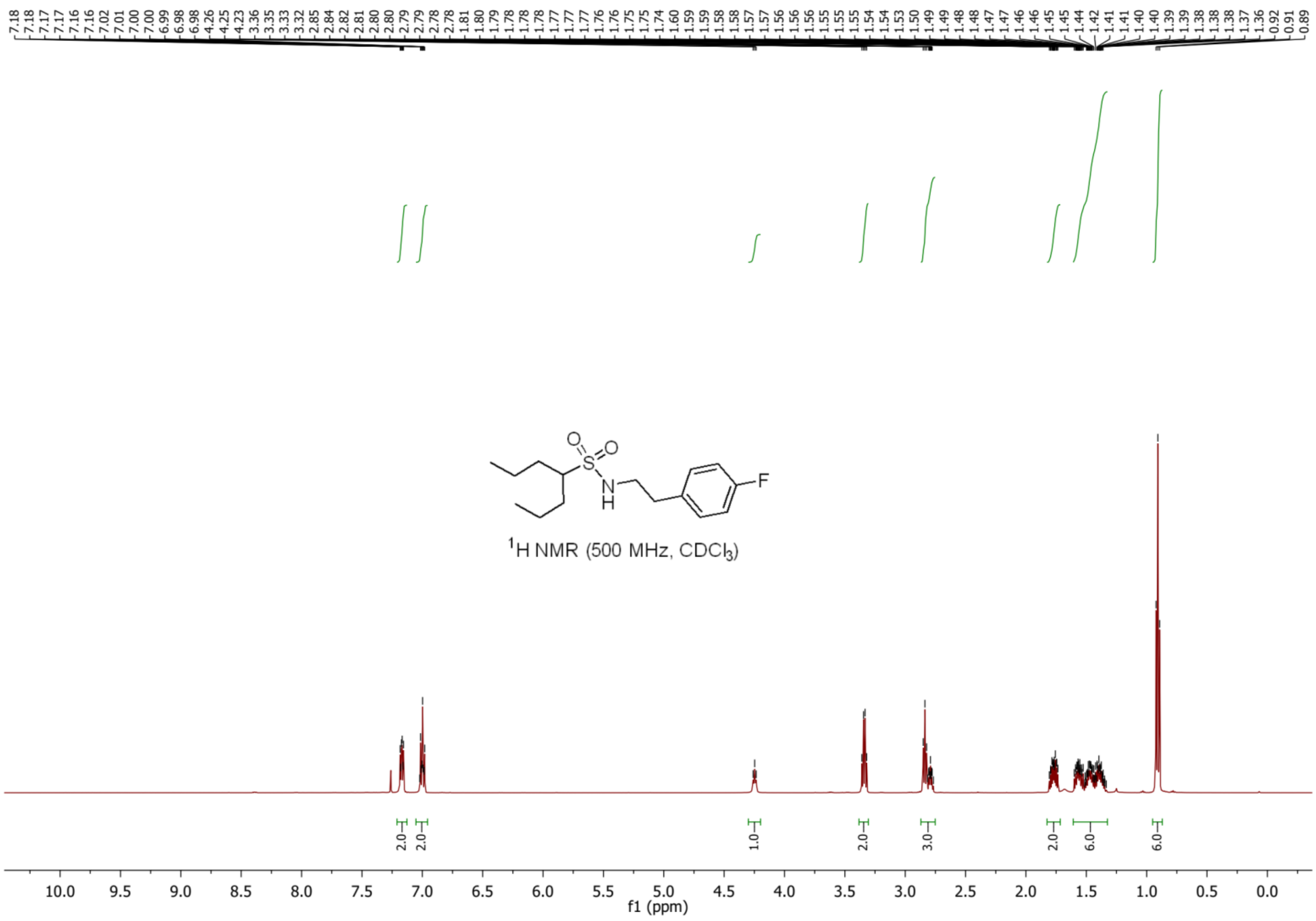

Go back to table of contents

Evaluation Only. Created with Aspose.Pdf. Copyright 2002-2014 Aspose Pty Ltd.

N-(4-Fluorophenethyl)heptane-4-sulfonamide (5b)

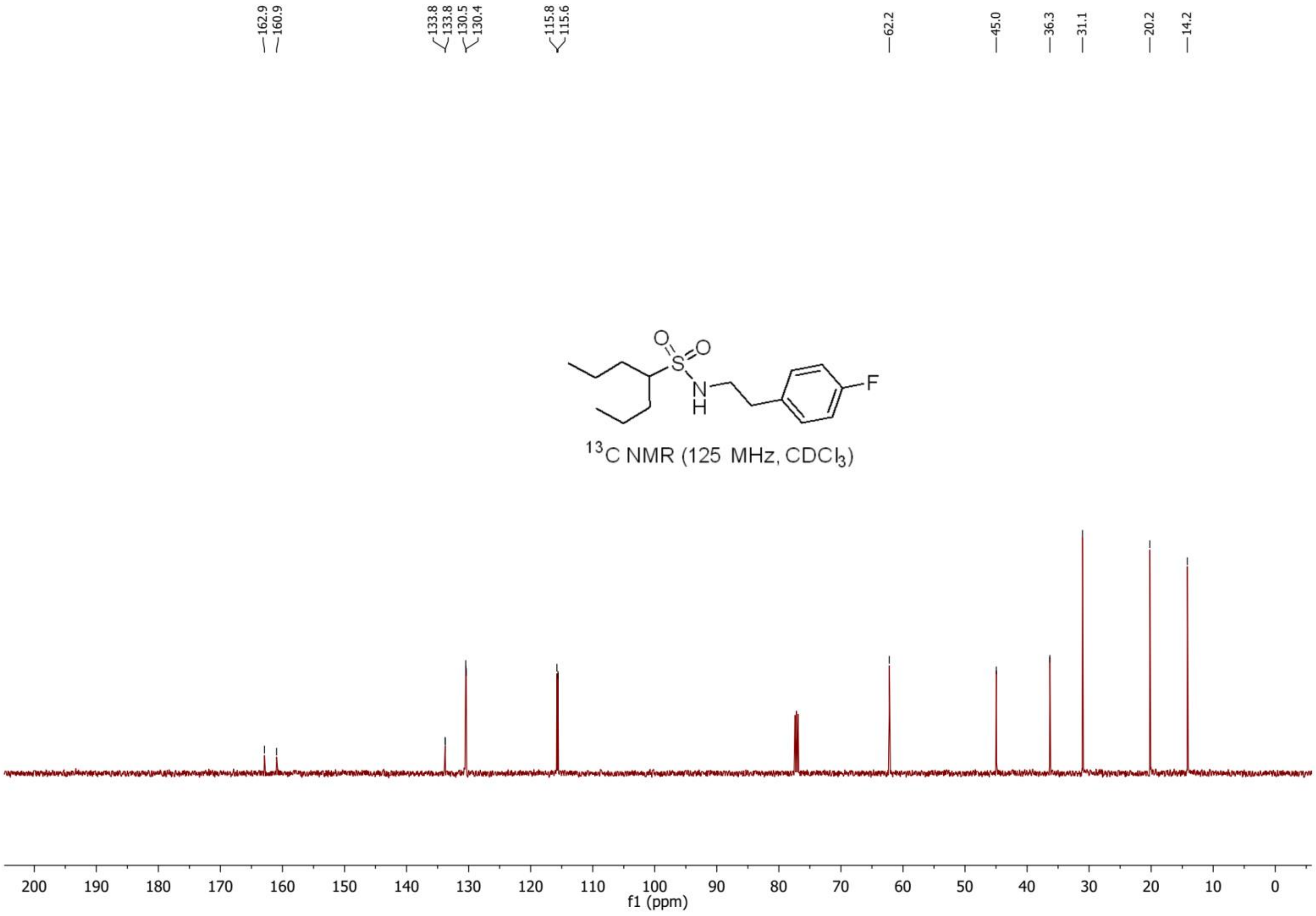

Go back to table of contents

Evaluation Only. Created with Aspose.Pdf. Copyright 2002-2014 Aspose Pty Ltd.

4,4-Difluoro-*N*-(4-fluorophenethyl)cyclohexane-1-sulfonamide (5c)

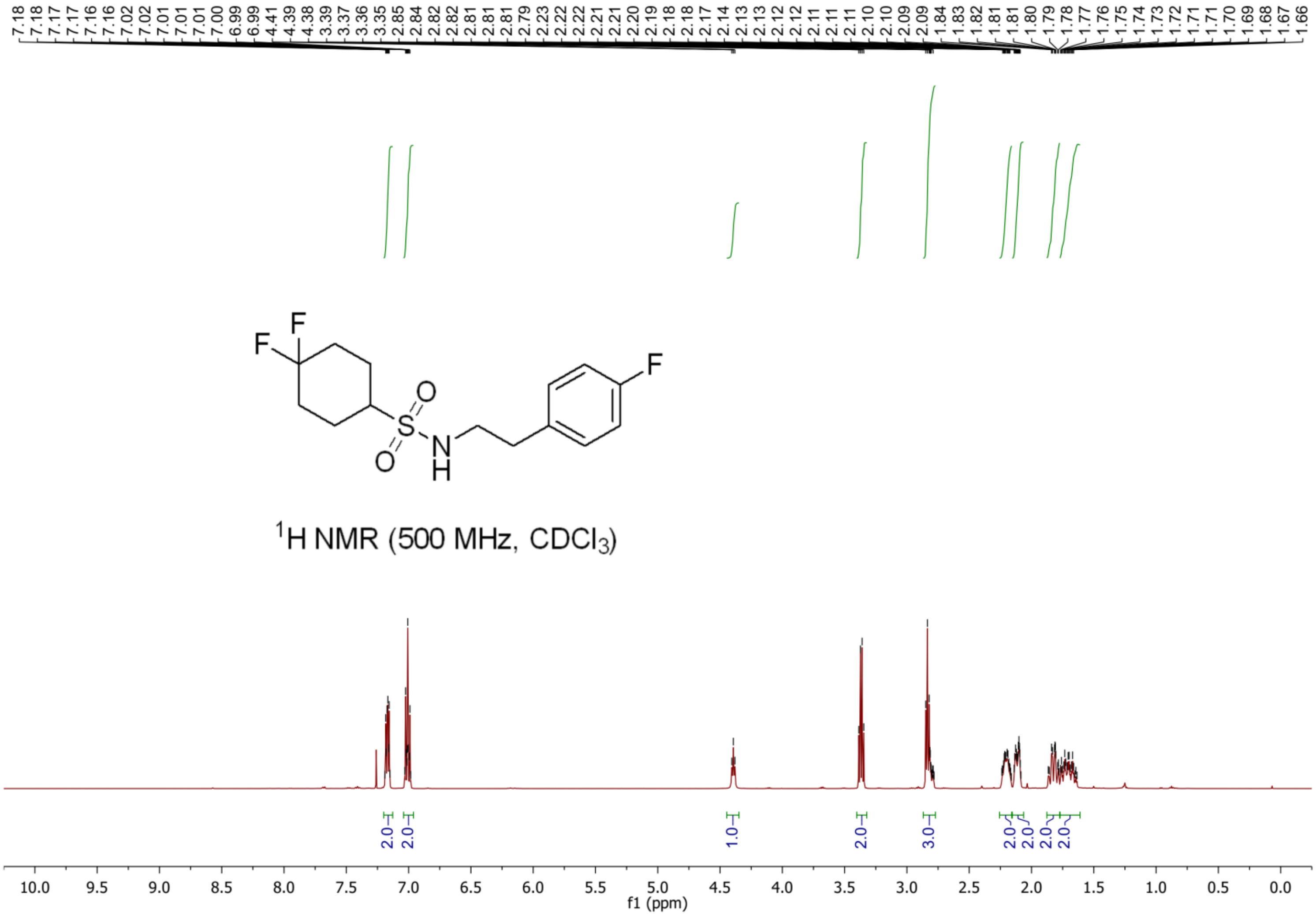

Evaluation Only. Created with Aspose.Pdf. Copyright 2002-2014 Aspose Pty Ltd.

4,4-Difluoro-*N*-(4-fluorophenethyl)cyclohexane-1-sulfonamide (5c)

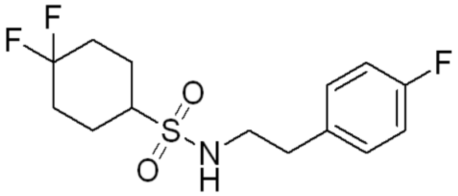

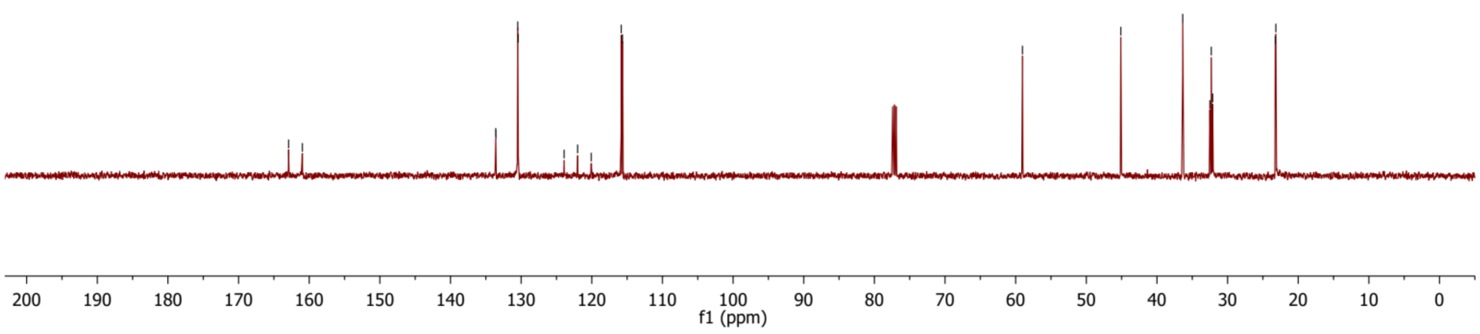

Go back to table of contents

Evaluation Only. Created with Aspose.Pdf. Copyright 2002-2014 Aspose Pty Ltd.

N-(4-Fluorophenethyl)adamantane-1-sulfonamide (5d)

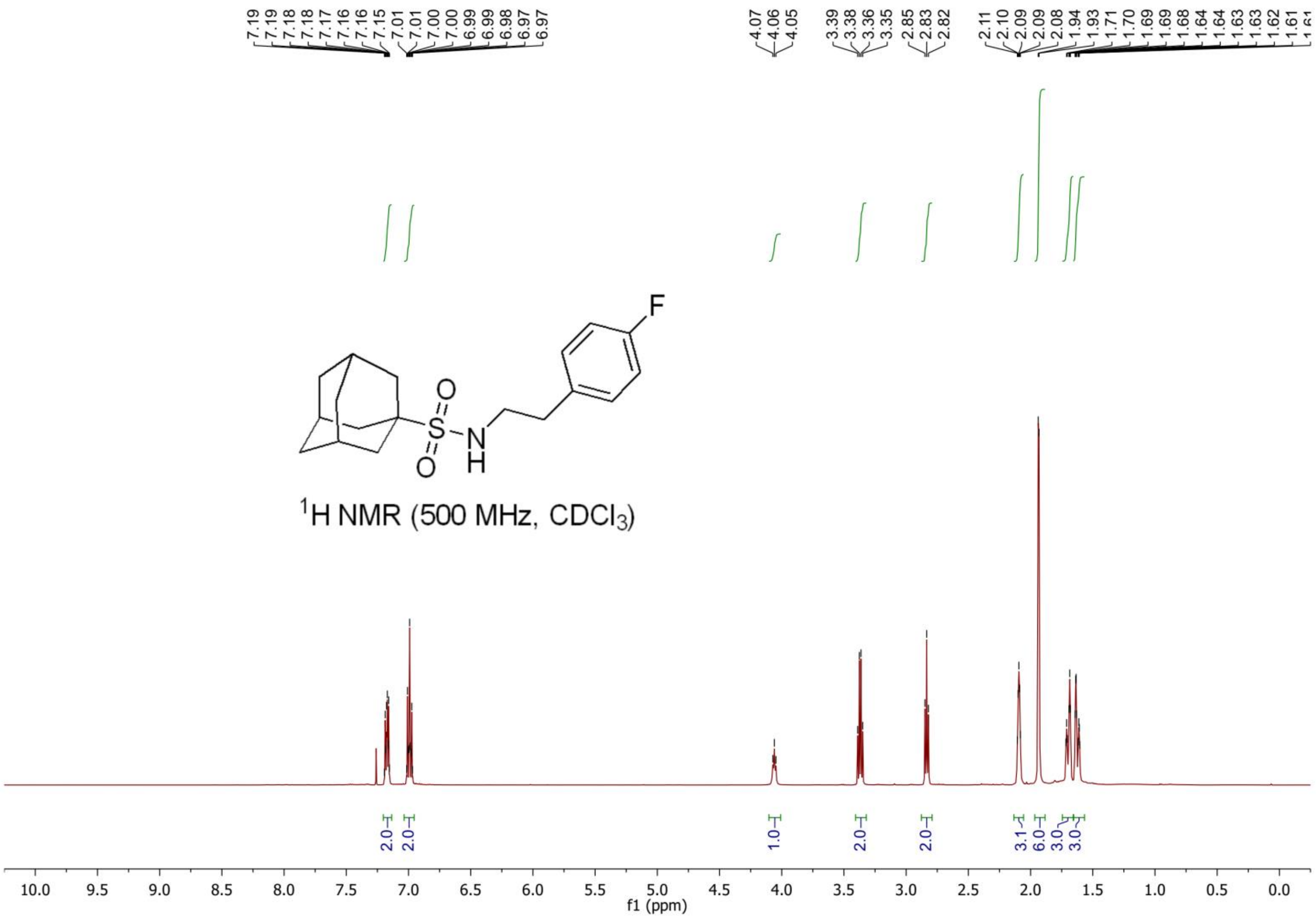

Go back to table of contents

Evaluation Only. Created with Aspose.Pdf. Copyright 2002-2014 Aspose Pty Ltd.

N-(4-Fluorophenethyl)adamantane-1-sulfonamide (5d)

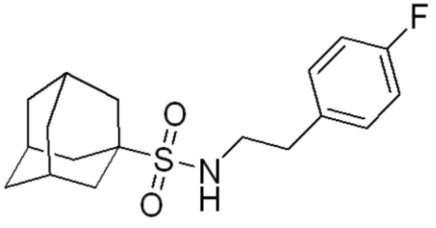

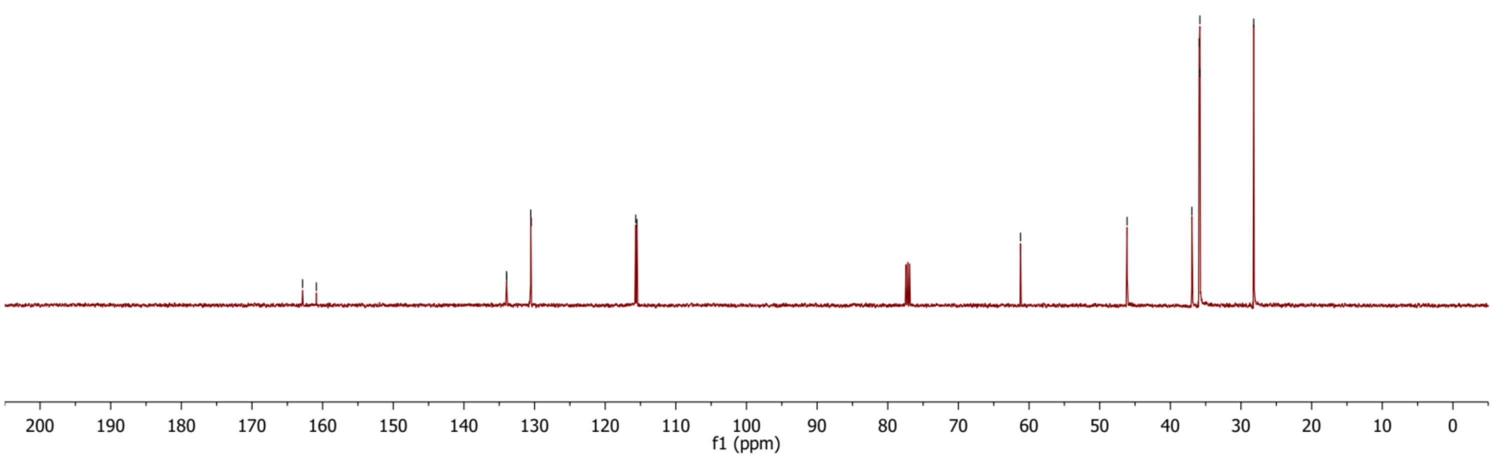

Go back to table of contents

Evaluation Only. Created with Aspose.Pdf. Copyright 2002-2014 Aspose Pty Ltd.

5-(2,5-Dimethylphenoxy)-*N*-(4-fluorophenethyl)-2-methylpentane-2-sulfonamide (5e)

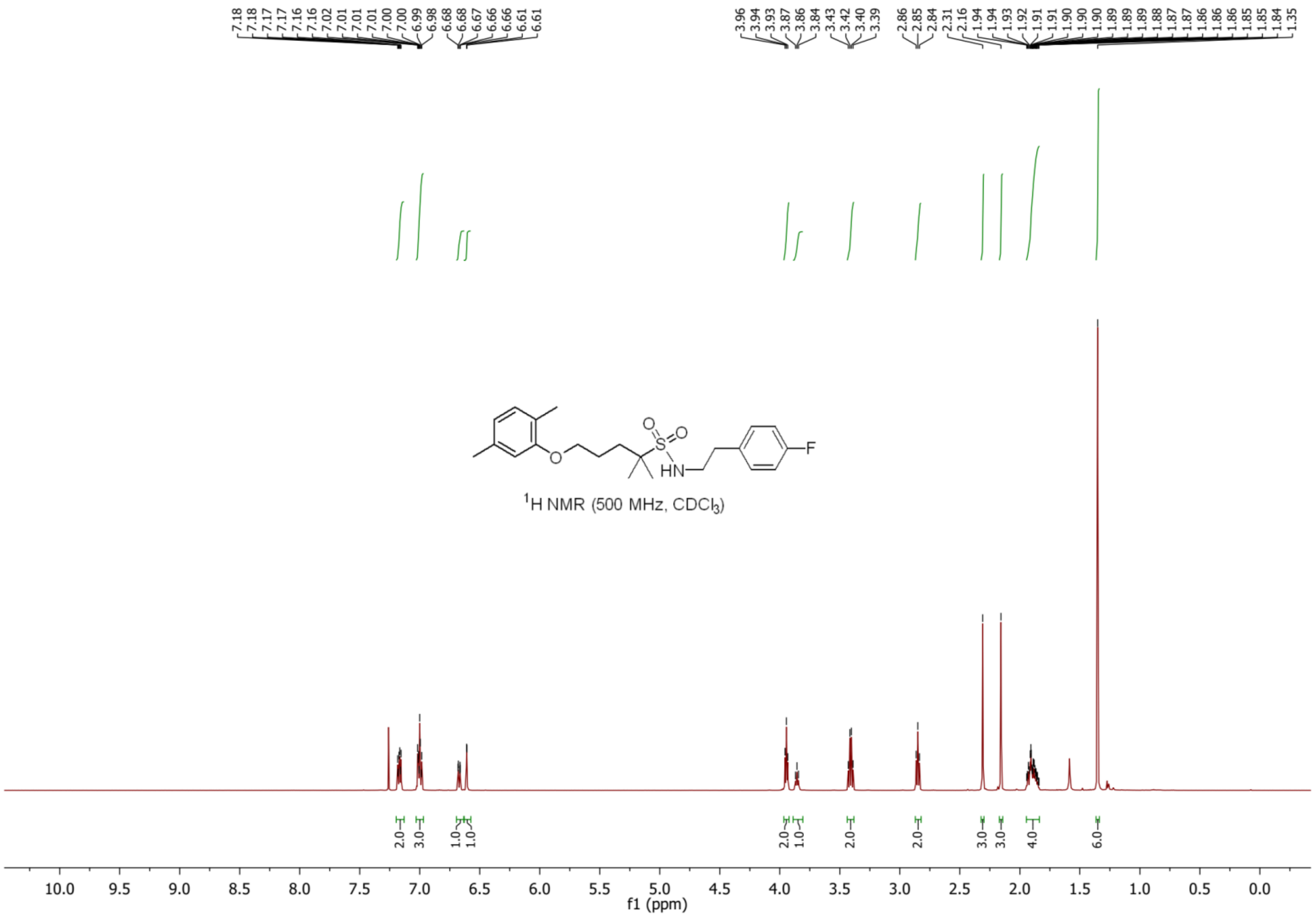

Evaluation Only. Created with Aspose.Pdf. Copyright 2002-2014 Aspose Pty Ltd.

5-(2,5-dimethylphenoxy)-*N*-(4-fluorophenethyl)-2-methylpentane-2-sulfonamide (5e)

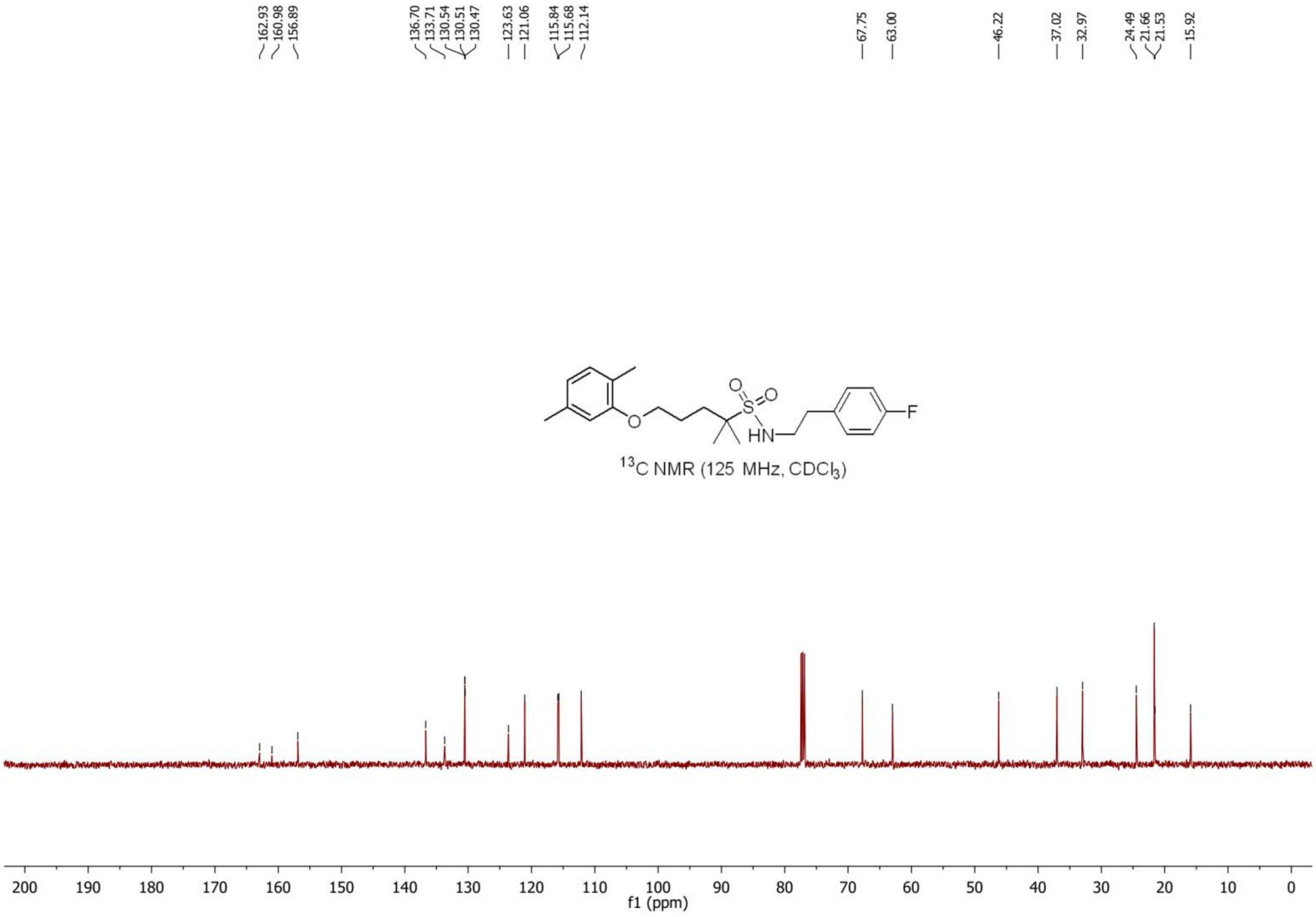

Go back to table of contents

Evaluation Only. Created with Aspose.Pdf. Copyright 2002-2014 Aspose Pty Ltd.

Methyl (1*R**,2*S**)-2-(*N*-((*R*)-1-phenylethyl)sulfamoyl)cyclohexane-1-carboxylate (6a)

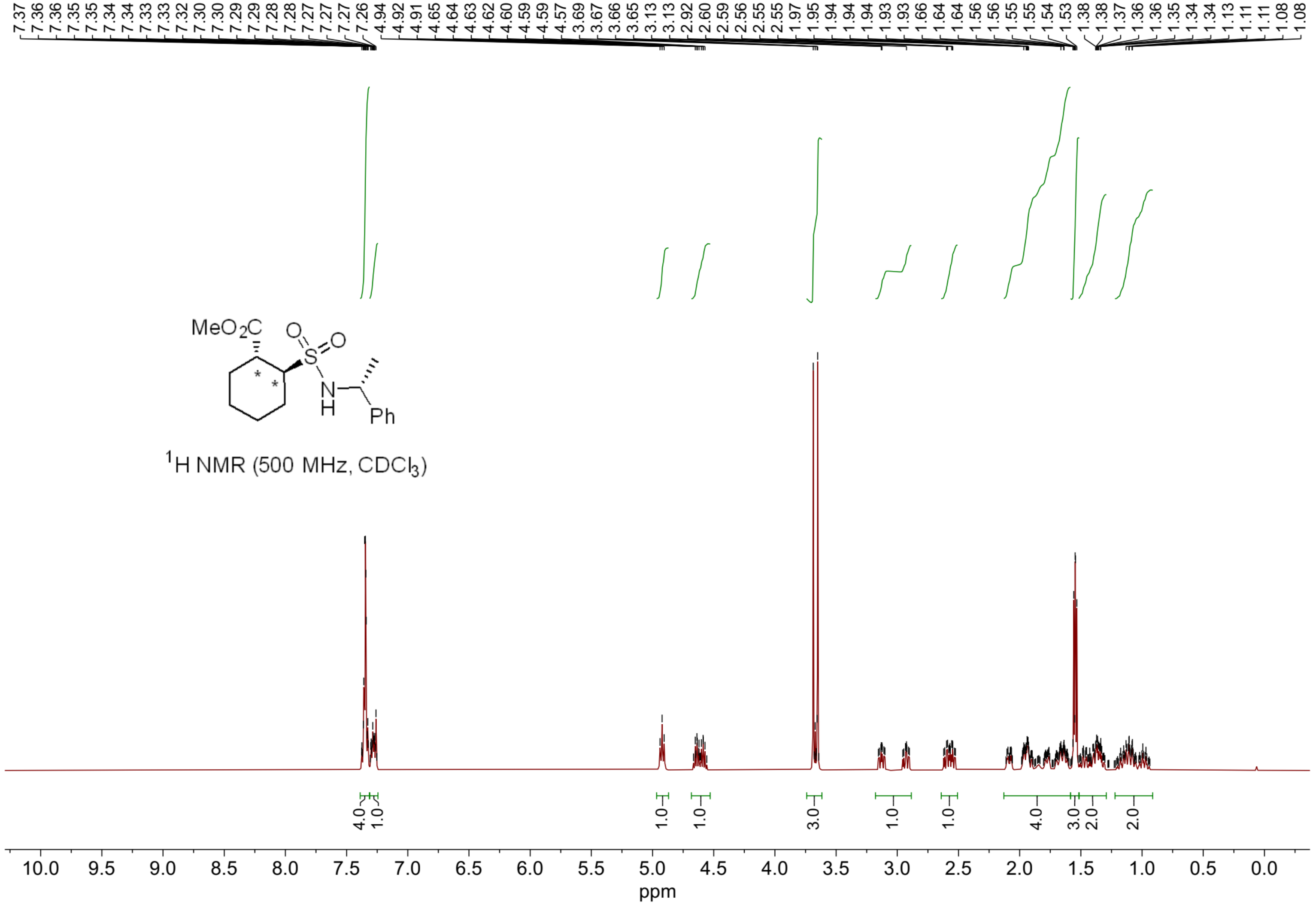

Go back to table of contents

Evaluation Only. Created with Aspose.Pdf. Copyright 2002-2014 Aspose Pty Ltd.

Methyl (1*R**,2*S**)-2-(*N*-((*R*)-1-phenylethyl)sulfamoyl)cyclohexane-1-carboxylate (6a)

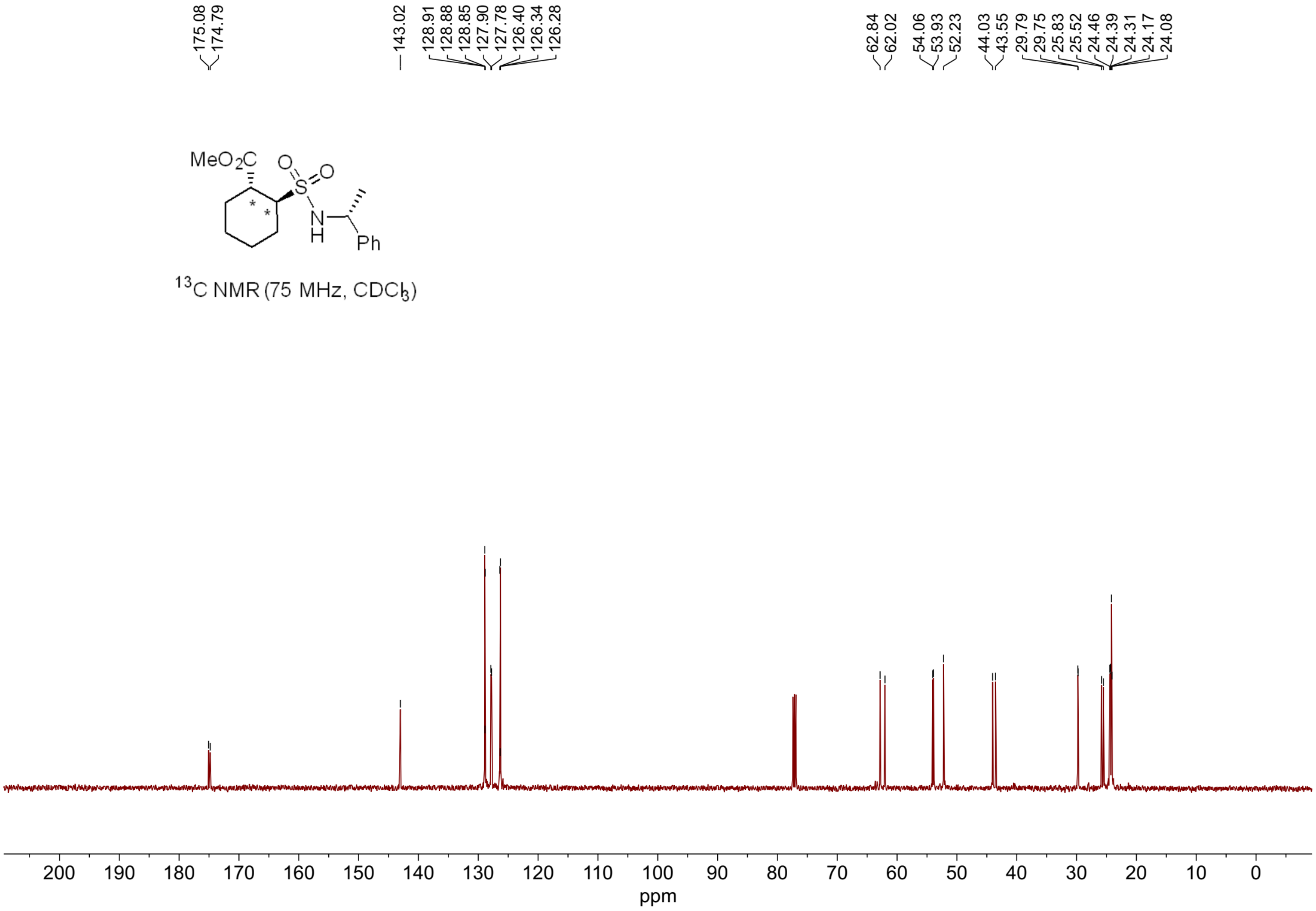

Go back to table of contents

Evaluation Only. Created with Aspose.Pdf. Copyright 2002-2014 Aspose Pty Ltd.

(3*S*,5*S*,7*S*)-*N*-((*R*)-1-Phenylethyl)adamantane-1-sulfonamide (6b)

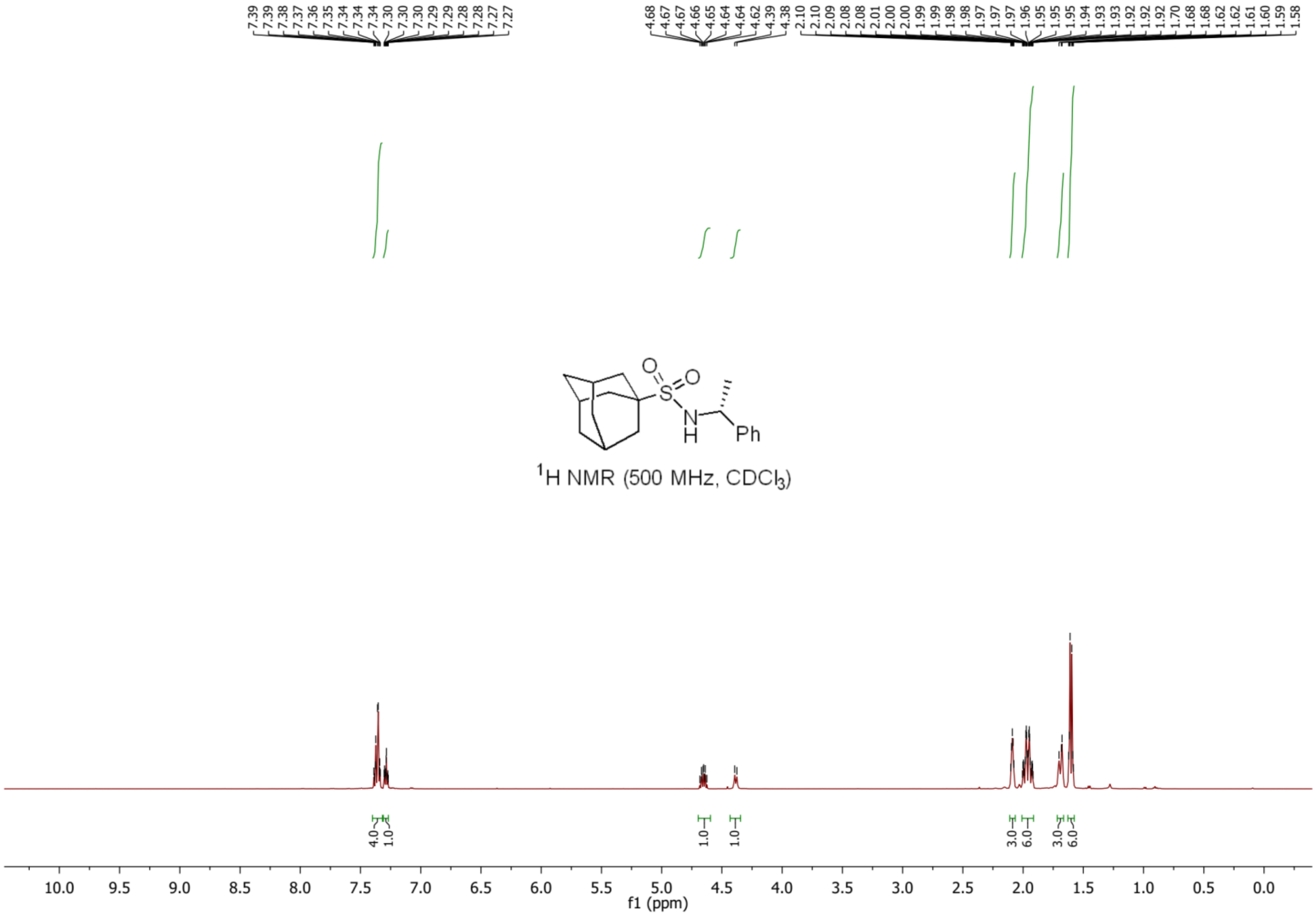

Go back to table of contents

Evaluation Only. Created with Aspose.Pdf. Copyright 2002-2014 Aspose Pty Ltd.

(3*S*,5*S*,7*S*)-*N*-((*R*)-1-Phenylethyl)adamantane-1-sulfonamide (6b)

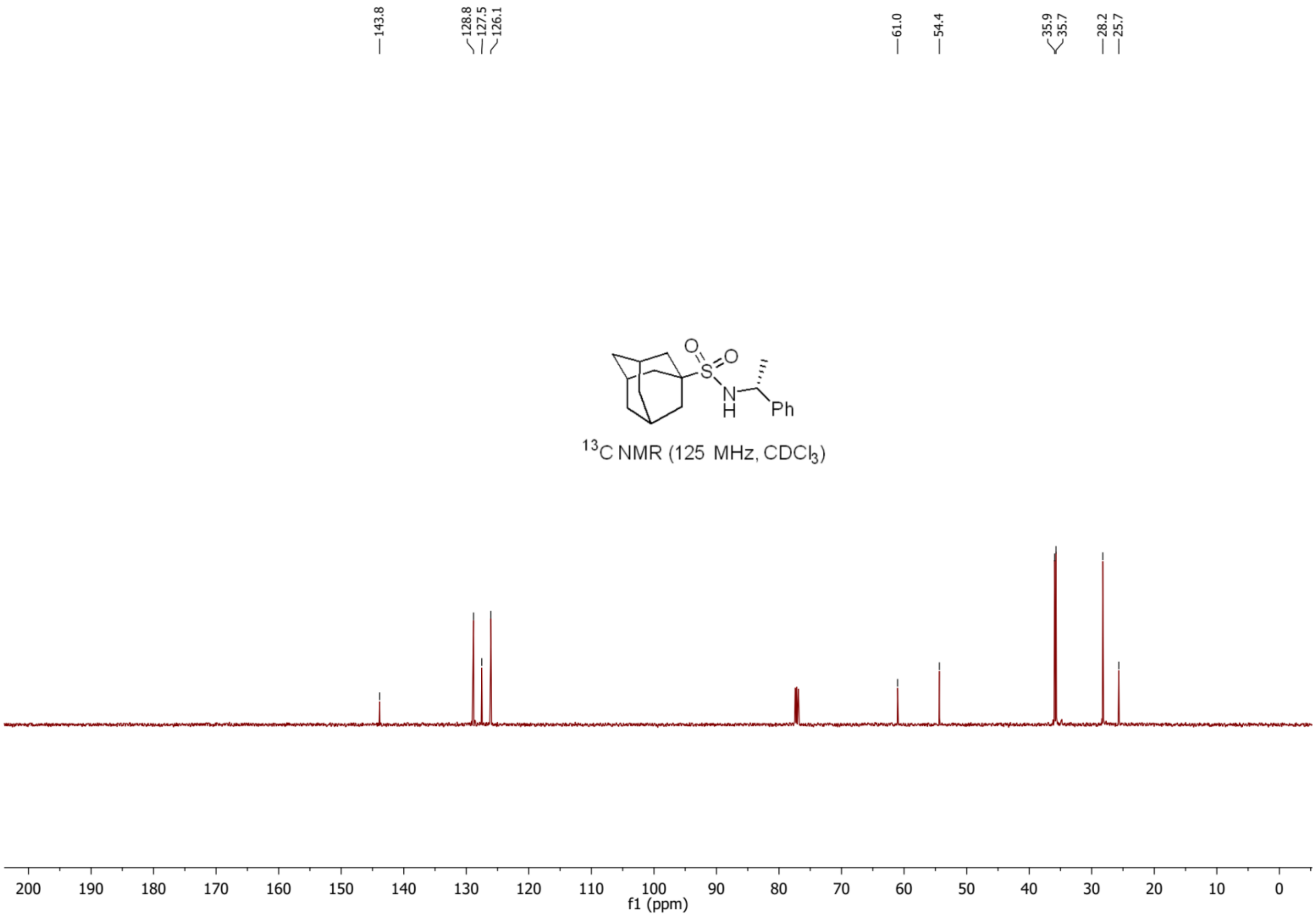

Go back to table of contents

Evaluation Only. Created with Aspose.Pdf. Copyright 2002-2014 Aspose Pty Ltd.

N,N-Dibenzyl-4-(5-methylthiophen-2-yl)-4-oxobutane-1-sulfonamide (7a)

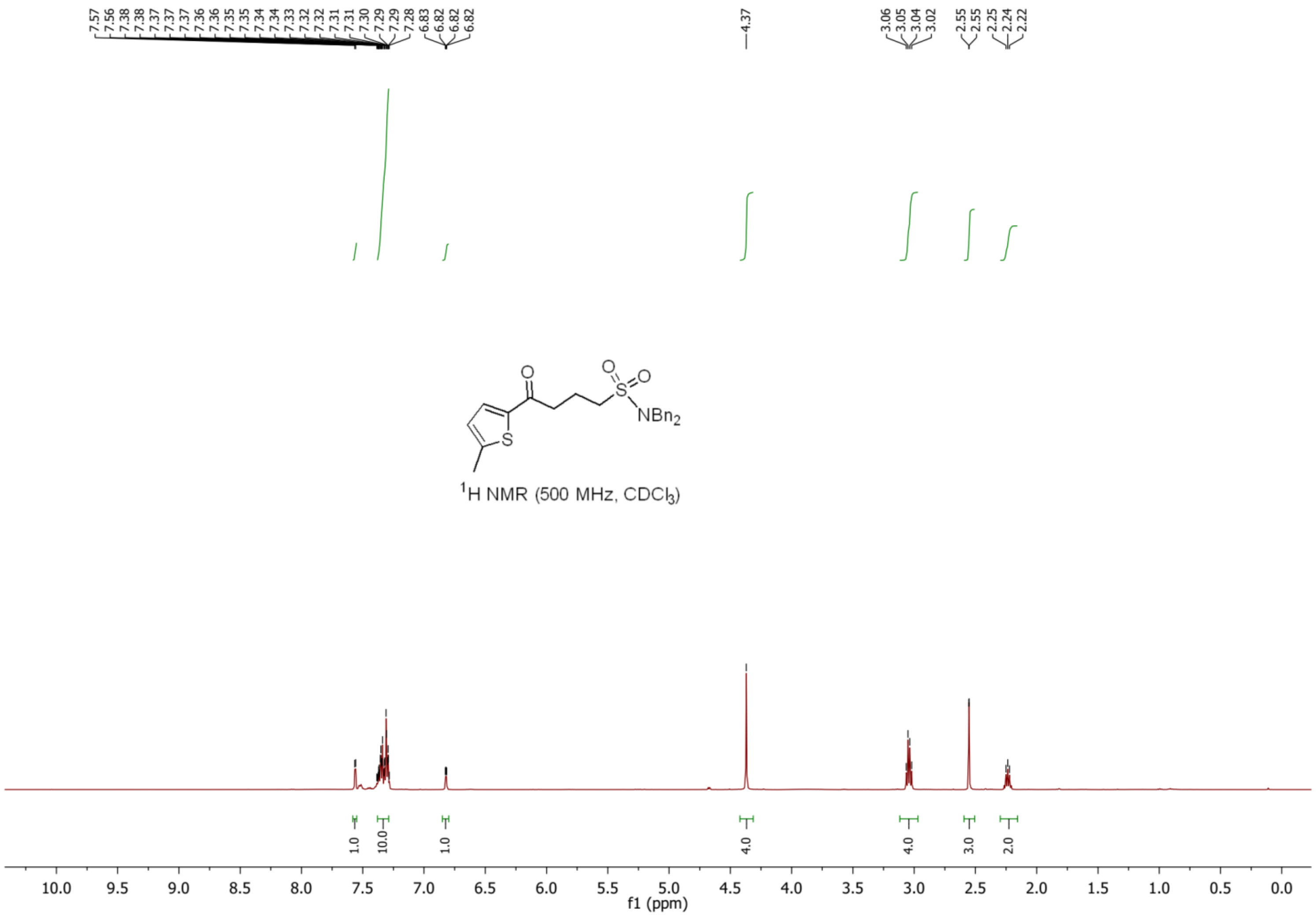

Go back to table of contents

Evaluation Only. Created with Aspose.Pdf. Copyright 2002-2014 Aspose Pty Ltd.

N,N-Dibenzyl-4-(5-methylthiophen-2-yl)-4-oxobutane-1-sulfonamide (7a)

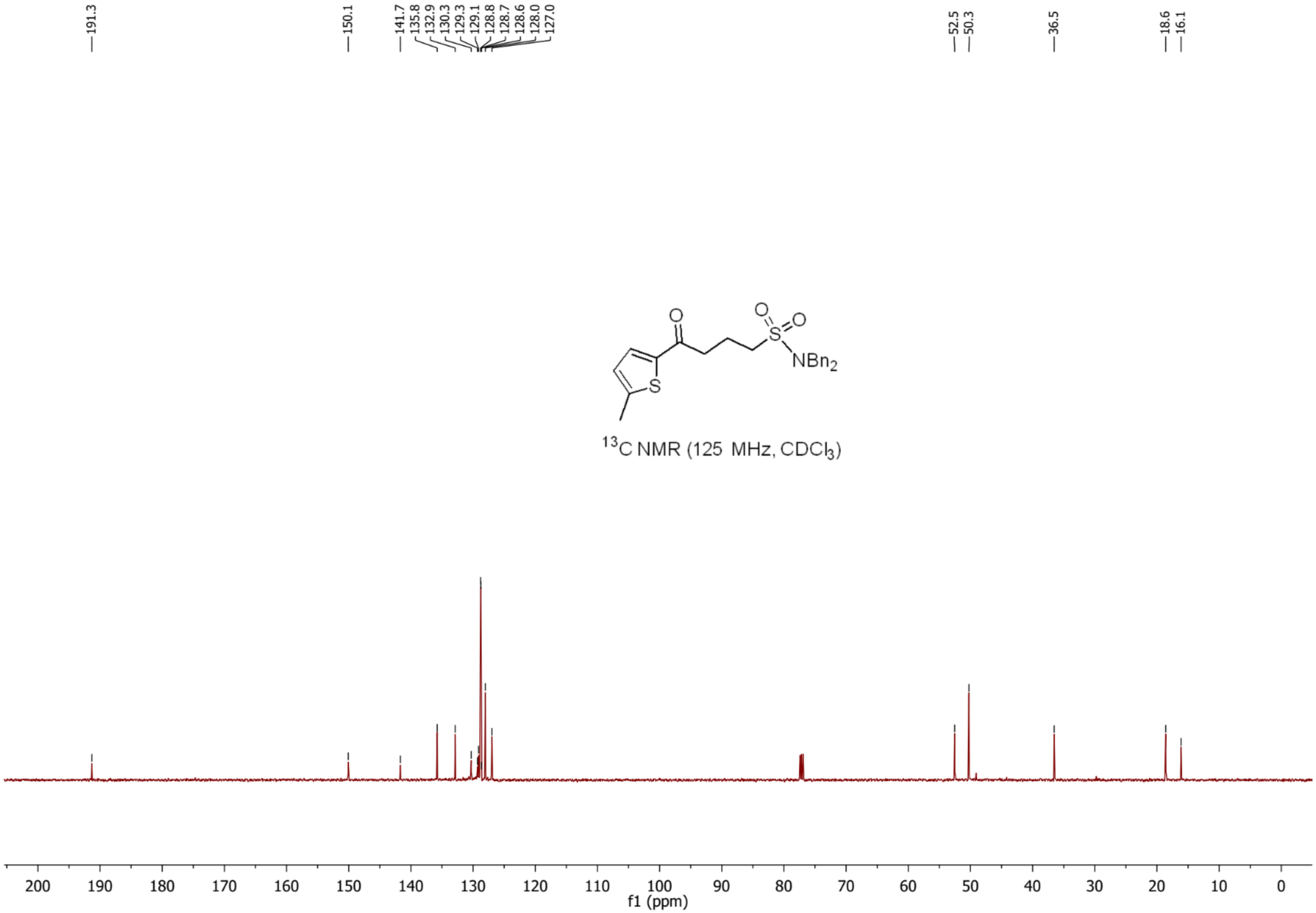

Go back to table of contents

Evaluation Only. Created with Aspose.Pdf. Copyright 2002-2014 Aspose Pty Ltd.

N,N-Dibenzylcyclohexanesulfonamide (7b)

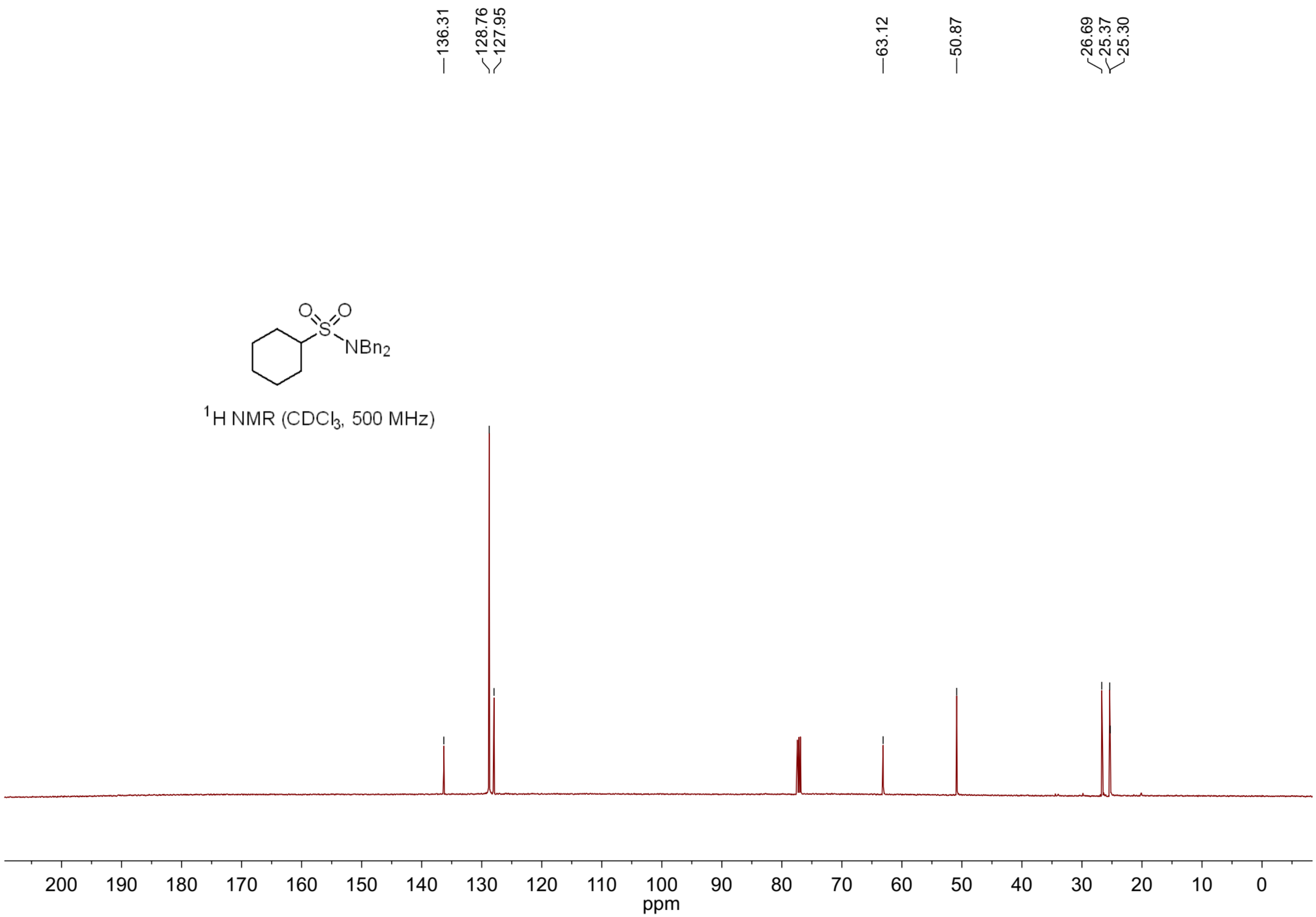

Go back to table of contents

Evaluation Only. Created with Aspose.Pdf. Copyright 2002-2014 Aspose Pty Ltd.

N,N-Dibenzylcyclohexanesulfonamide (7b)

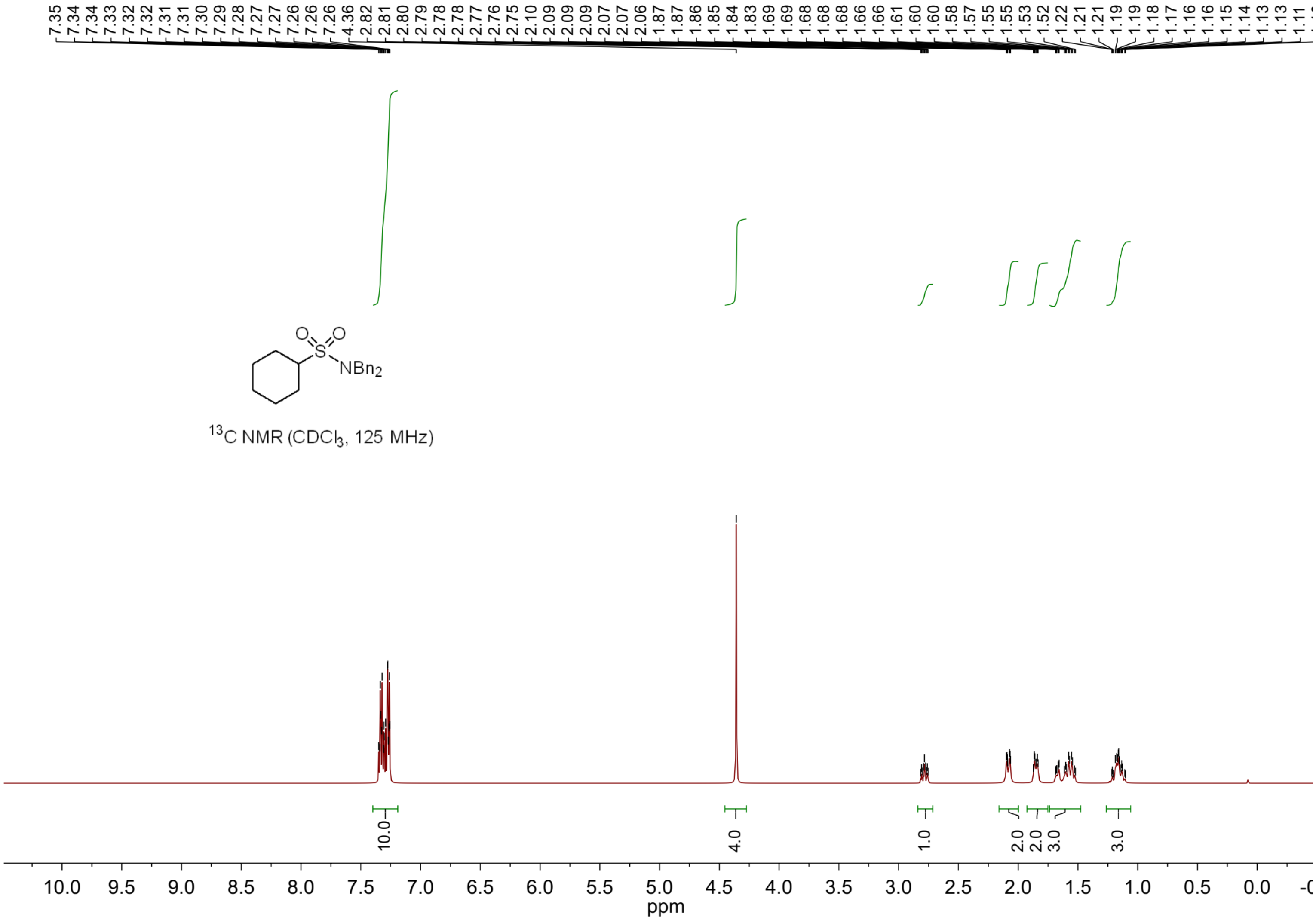

Go back to table of contents

Evaluation Only. Created with Aspose.Pdf. Copyright 2002-2014 Aspose Pty Ltd.

N,N-Dibenzyltetrahydro-2*H*-pyran-4-sulfonamide (7c)

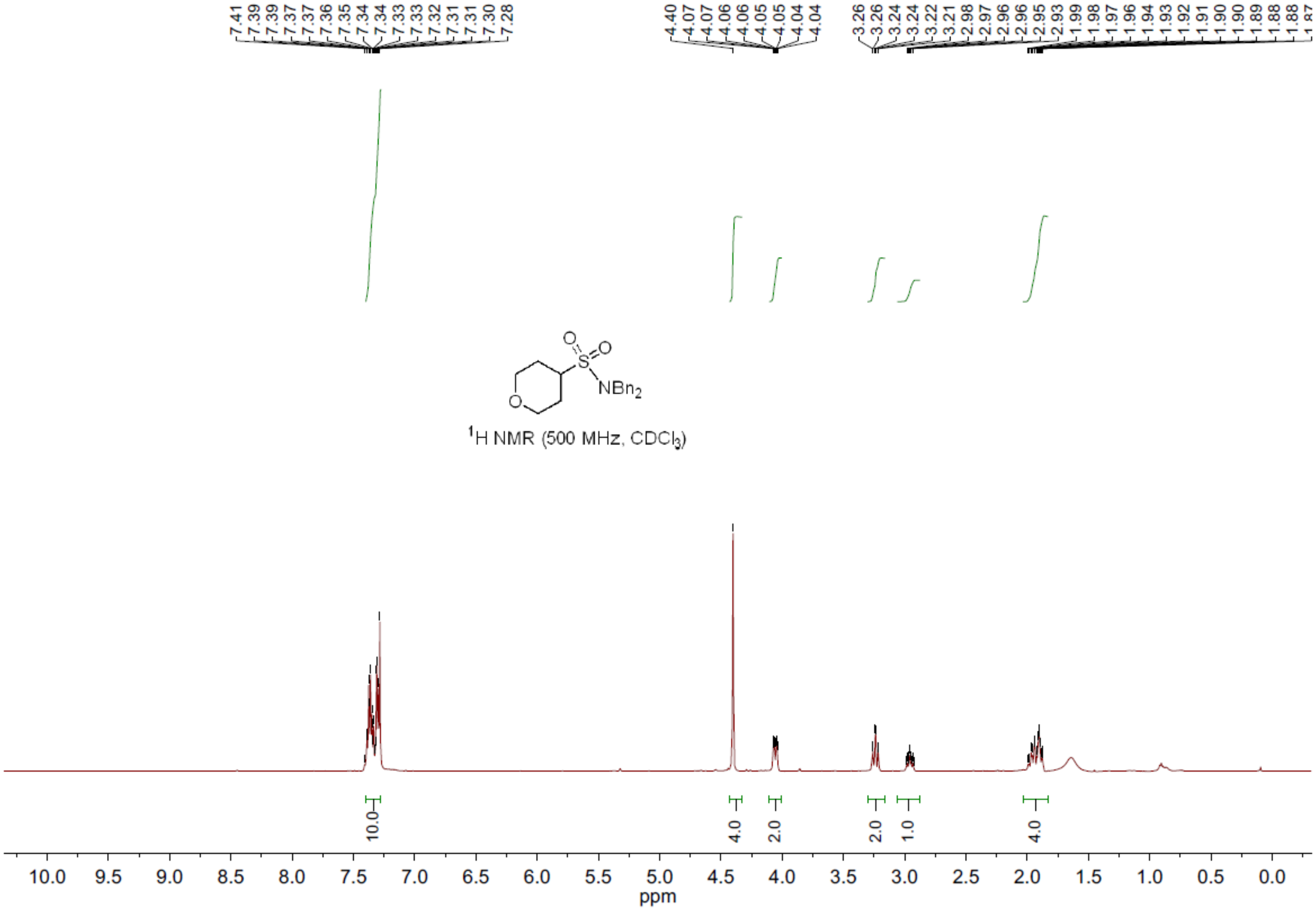

Go back to table of contents

Evaluation Only. Created with Aspose.Pdf. Copyright 2002-2014 Aspose Pty Ltd.

N,N-Dibenzyltetrahydro-2*H*-pyran-4-sulfonamide (7c)

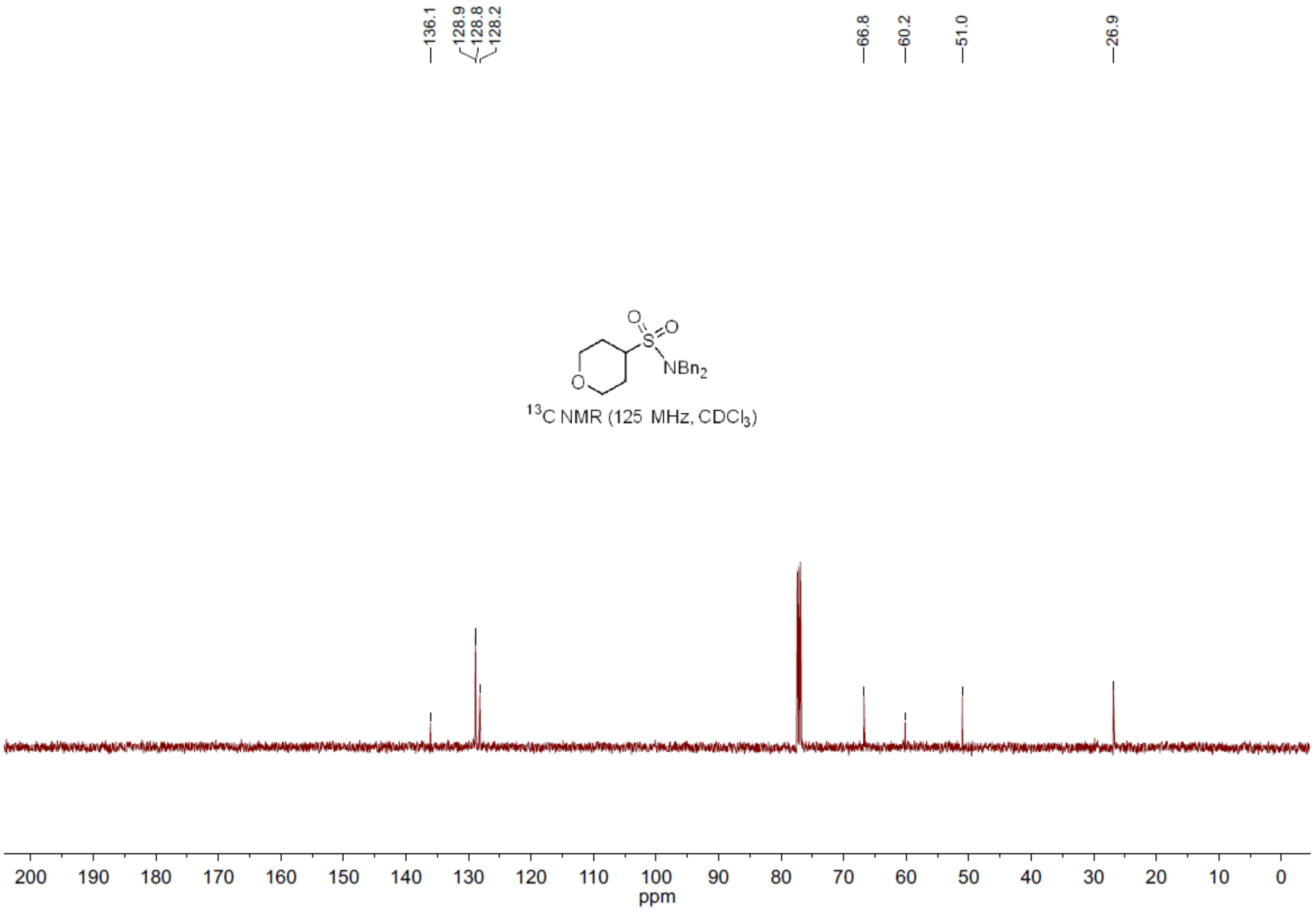

Go back to table of contents

Evaluation Only. Created with Aspose.Pdf. Copyright 2002-2014 Aspose Pty Ltd.

N- (*p*-Tolyl)cyclohexanesulfonamide (8a)

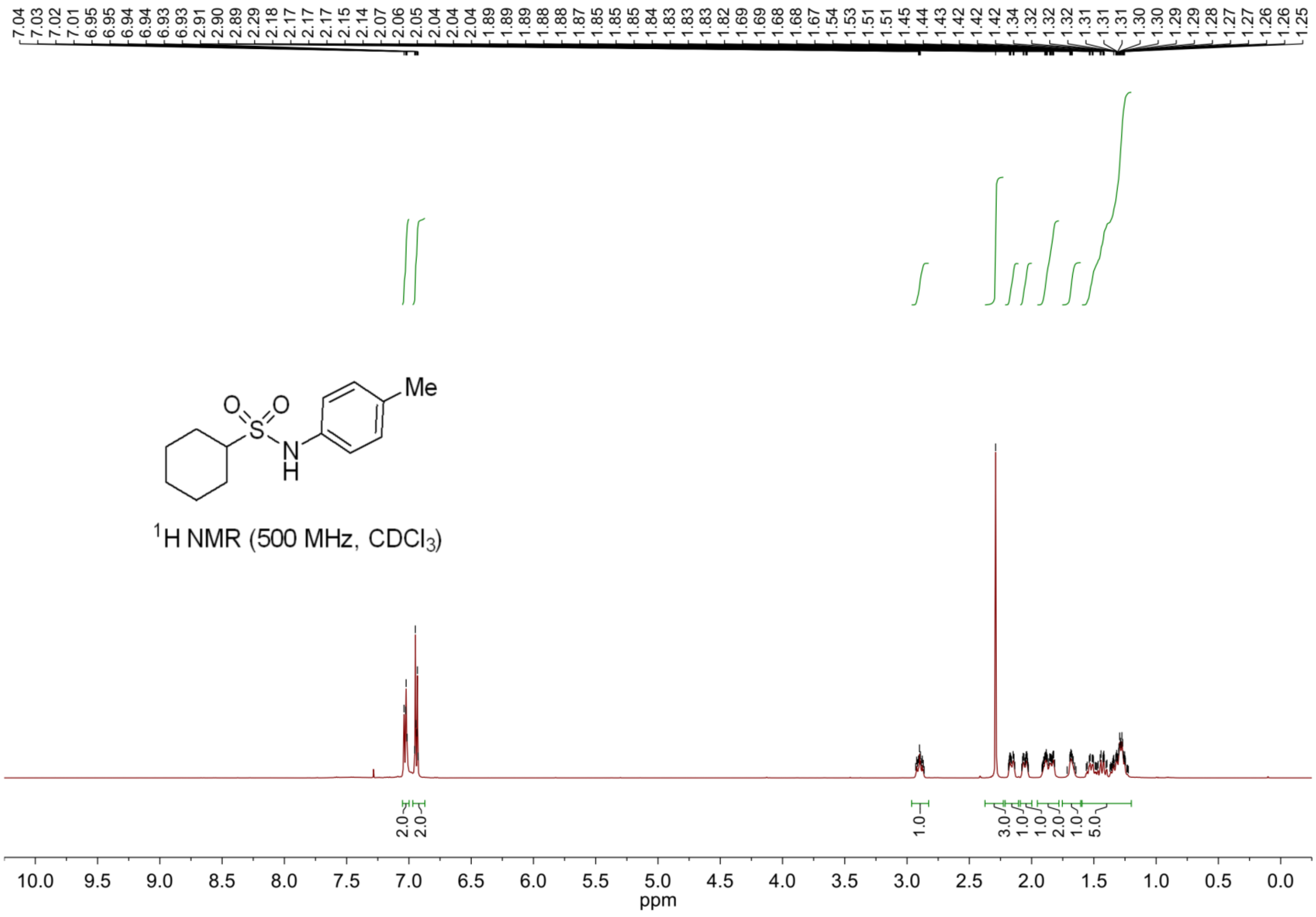

Go back to table of contents

Evaluation Only. Created with Aspose.Pdf. Copyright 2002-2014 Aspose Pty Ltd.

N- (*p*-Tolyl)cyclohexanesulfonamide (8a)

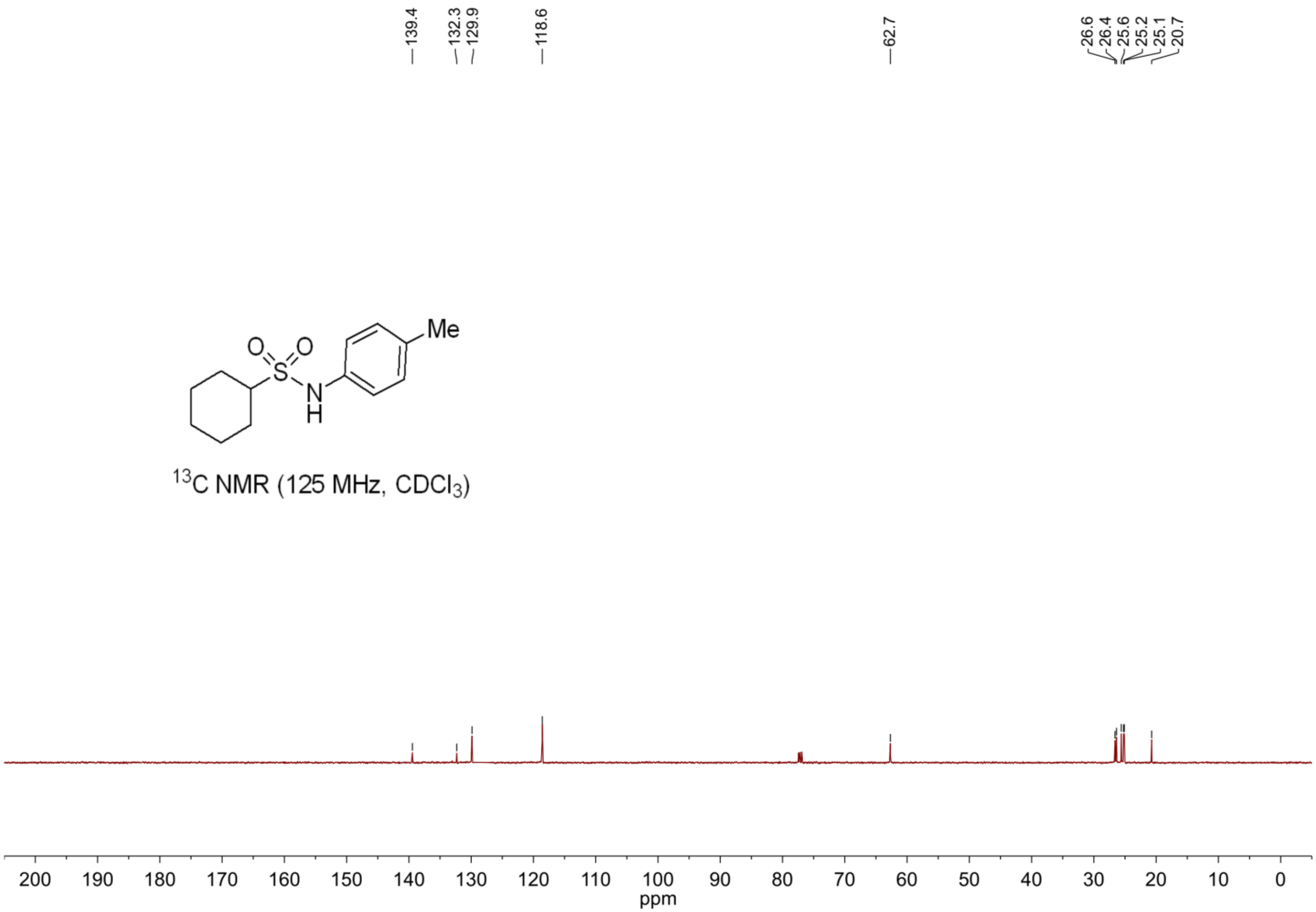

^{13}C NMR (125 MHz, $CDCl_3$)

Go back to table of contents

Evaluation Only. Created with Aspose.Pdf. Copyright 2002-2014 Aspose Pty Ltd.

N-(3,5-Dimethylphenyl)cyclohexanesulfonamide (8b)

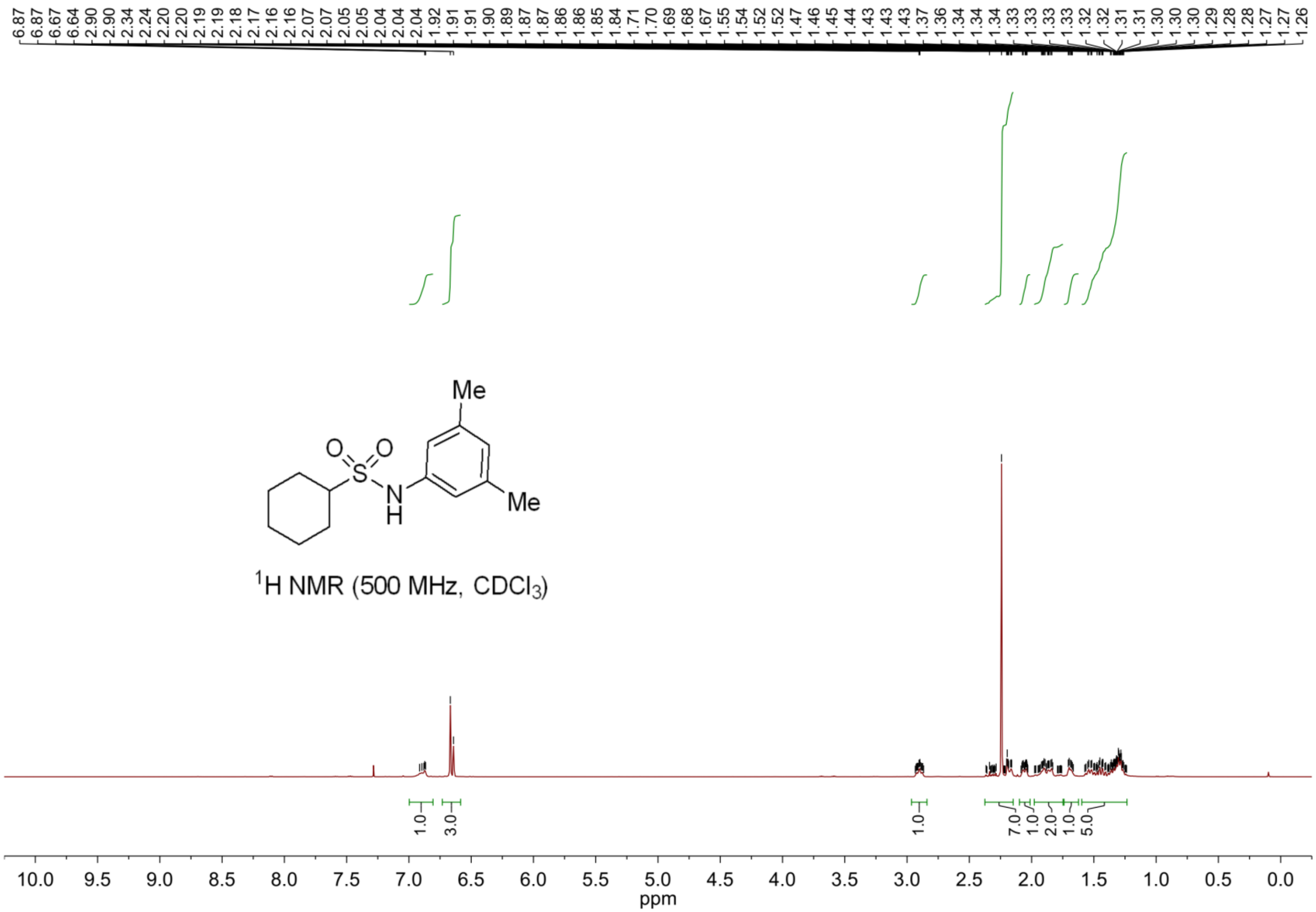

Go back to table of contents

Evaluation Only. Created with Aspose.Pdf. Copyright 2002-2014 Aspose Pty Ltd.

N-(3,5-Dimethylphenyl)cyclohexanesulfonamide (8b)

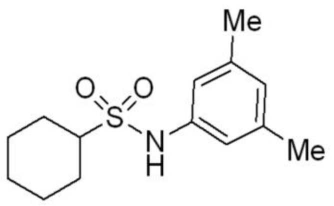

^{13}C NMR (125 MHz, $CDCl_3$)

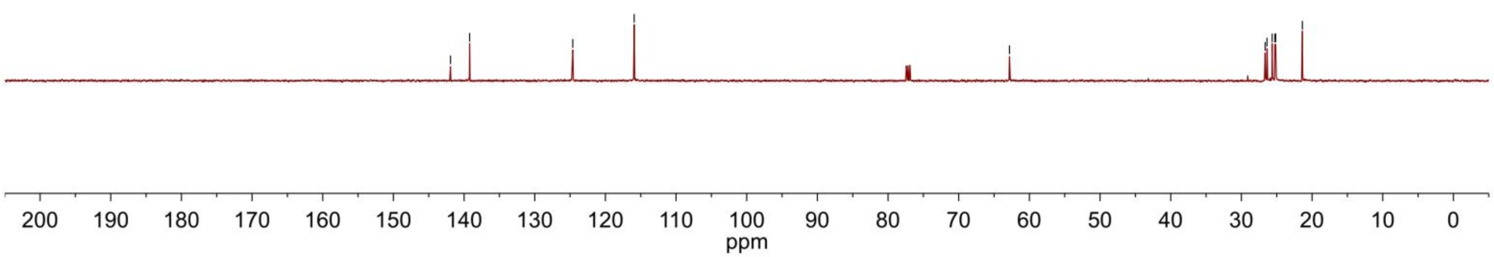

Go back to table of contents

Evaluation Only. Created with Aspose.Pdf. Copyright 2002-2014 Aspose Pty Ltd.

N-(3,4,5-Trimethylphenyl)cyclohexanesulfonamide (8c)

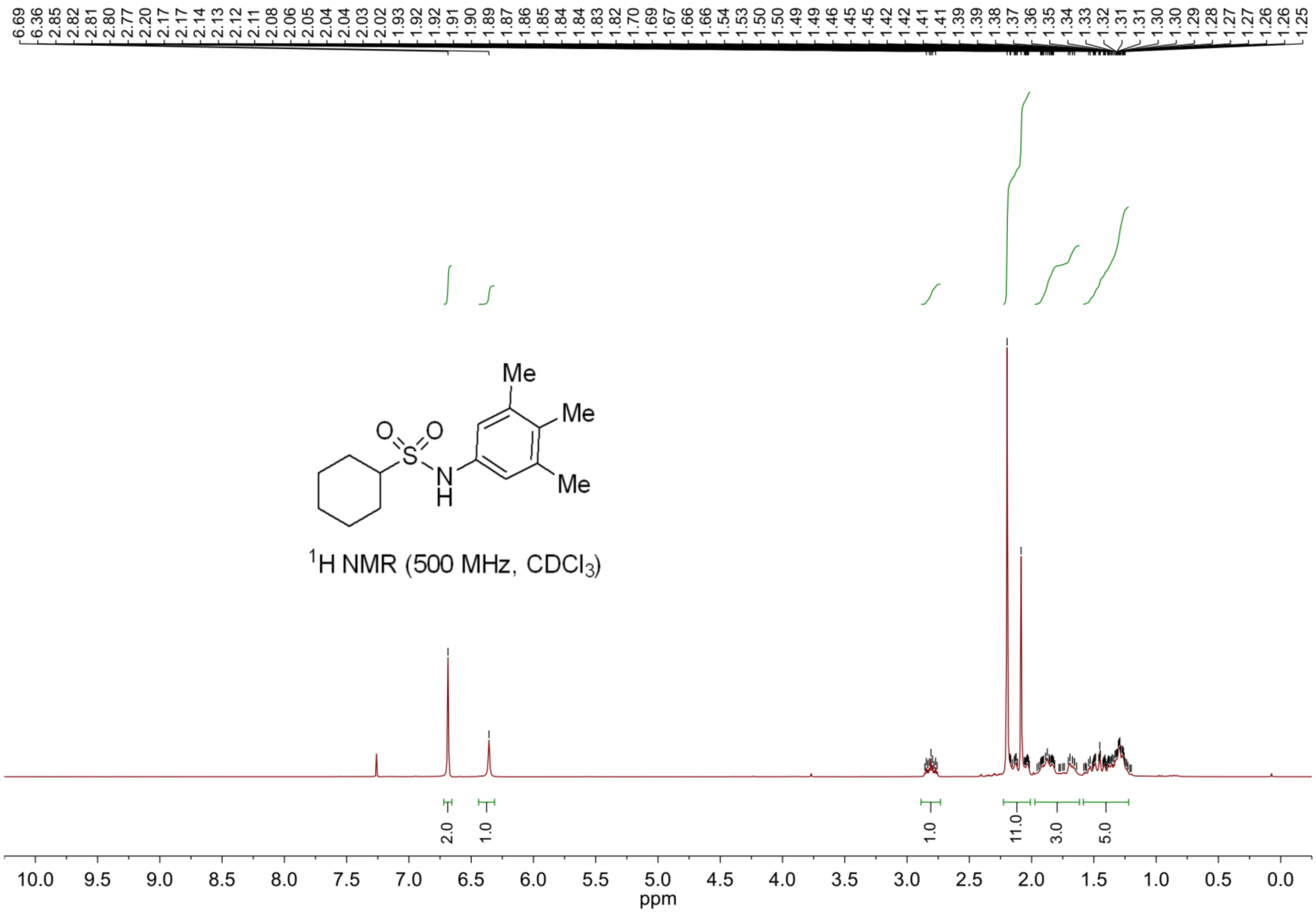

Go back to table of contents

Evaluation Only. Created with Aspose.Pdf. Copyright 2002-2014 Aspose Pty Ltd.

N-(3,4,5-Trimethylphenyl)cyclohexanesulfonamide (8c)

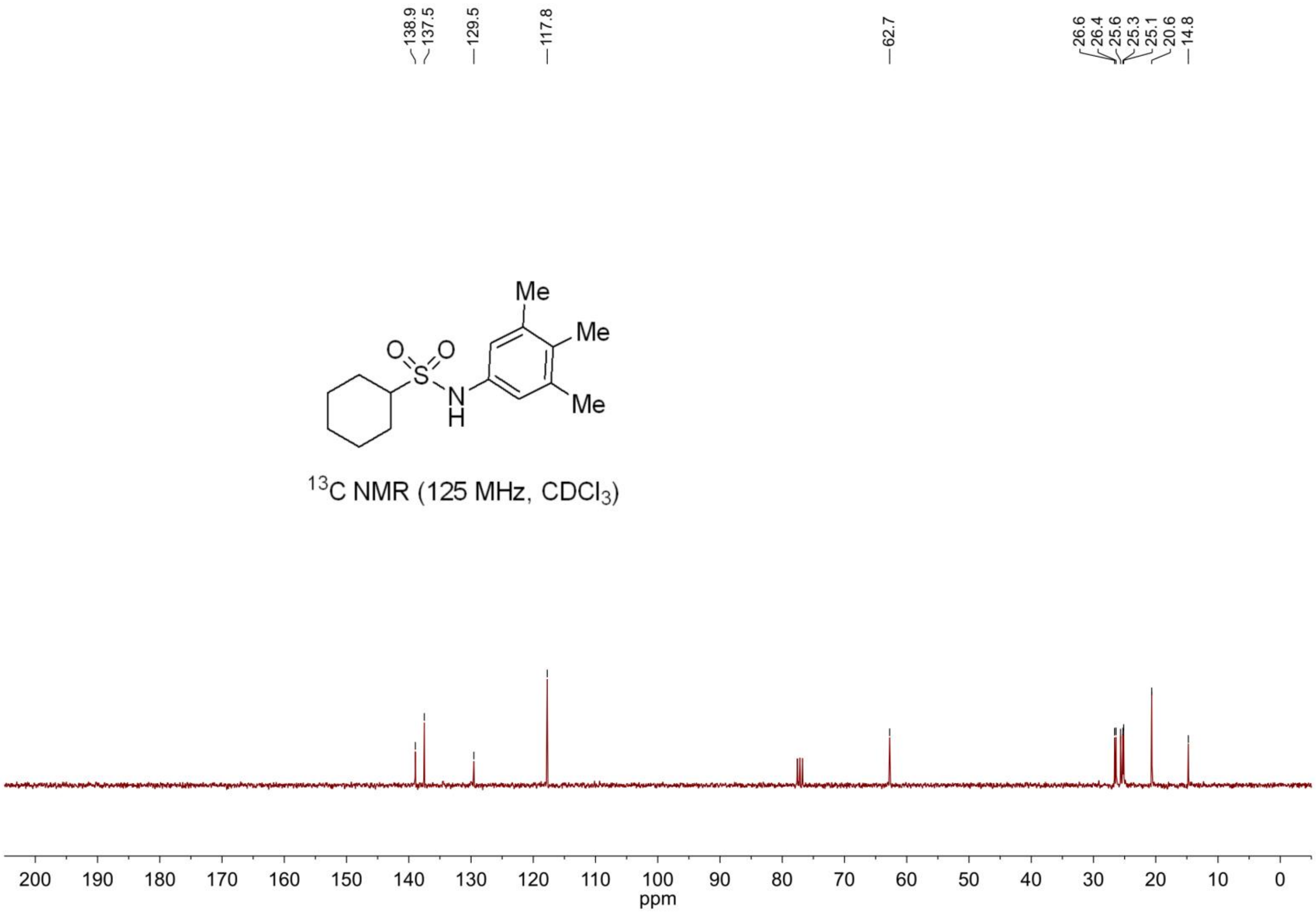

Go back to table of contents

Evaluation Only. Created with Aspose.Pdf. Copyright 2002-2014 Aspose Pty Ltd.

N-(4-Methoxyphenyl)cyclohexanesulfonamide (8d)

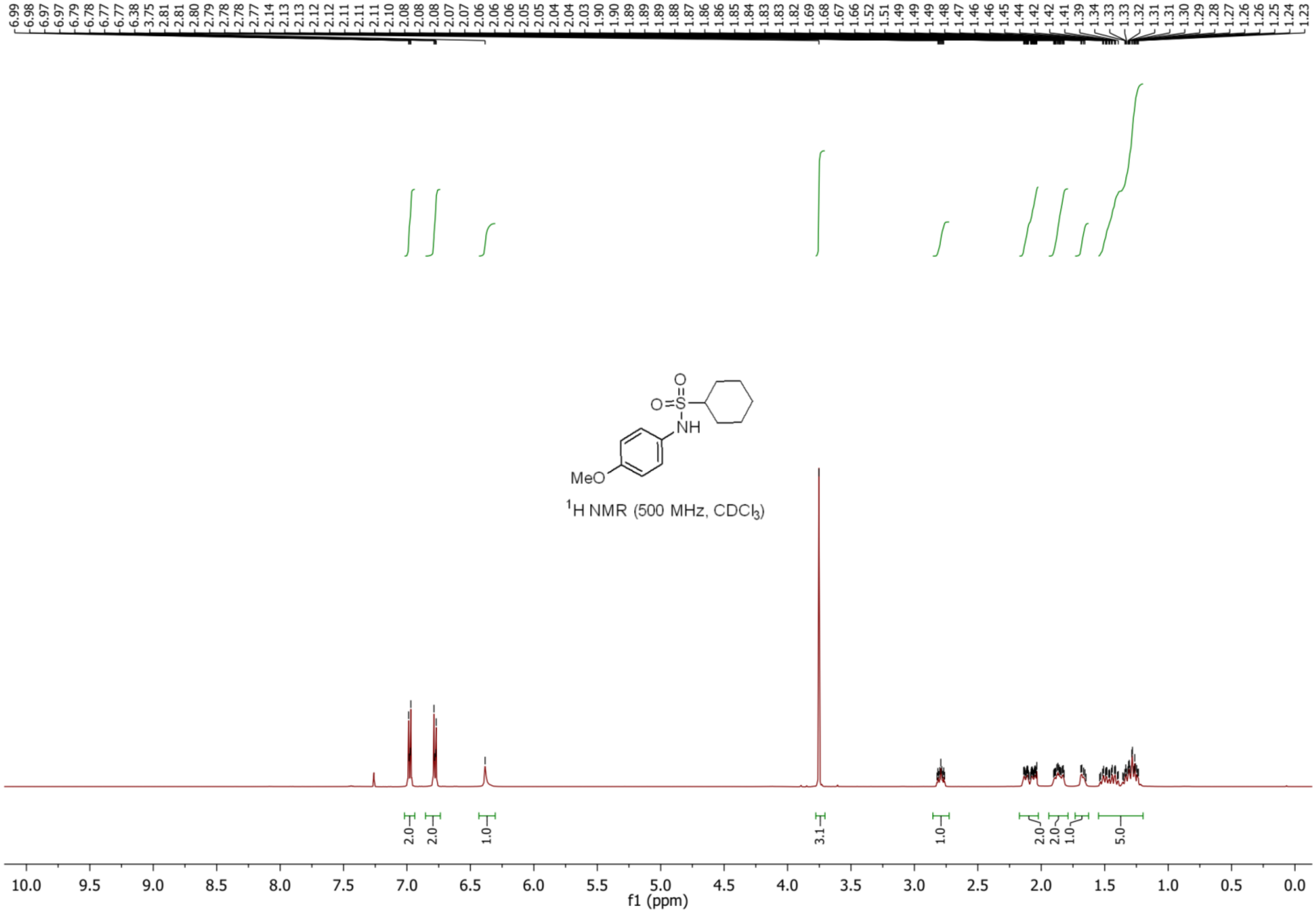

Go back to table of contents

Evaluation Only. Created with Aspose.Pdf. Copyright 2002-2014 Aspose Pty Ltd.

N-(4-Methoxyphenyl)cyclohexanesulfonamide (8d)

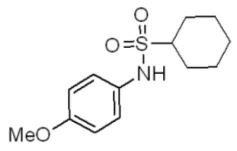

^{13}C NMR (125 MHz, $CDCl_3$)

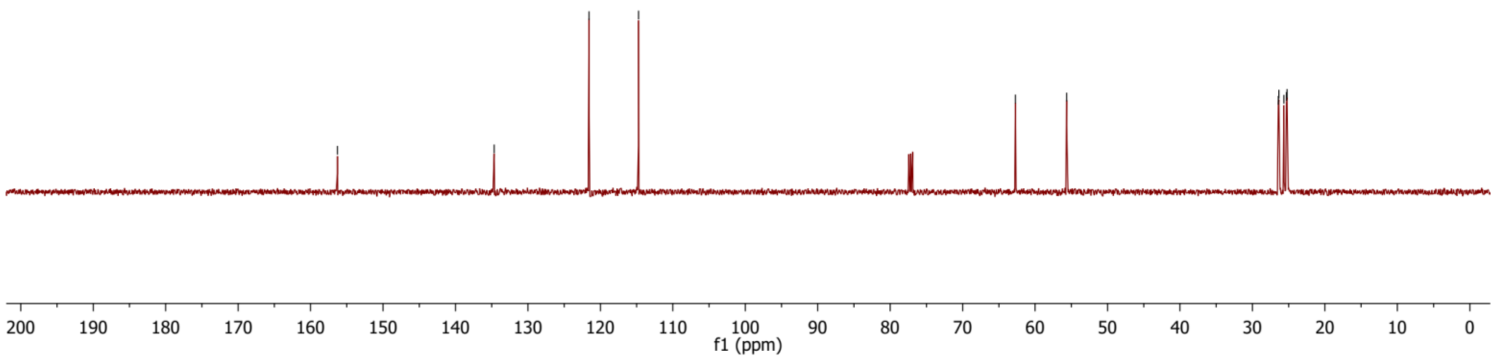

Go back to table of contents

Evaluation Only. Created with Aspose.Pdf. Copyright 2002-2014 Aspose Pty Ltd.

N-(2-Methoxyphenyl)cyclohexanesulfonamide (8e)

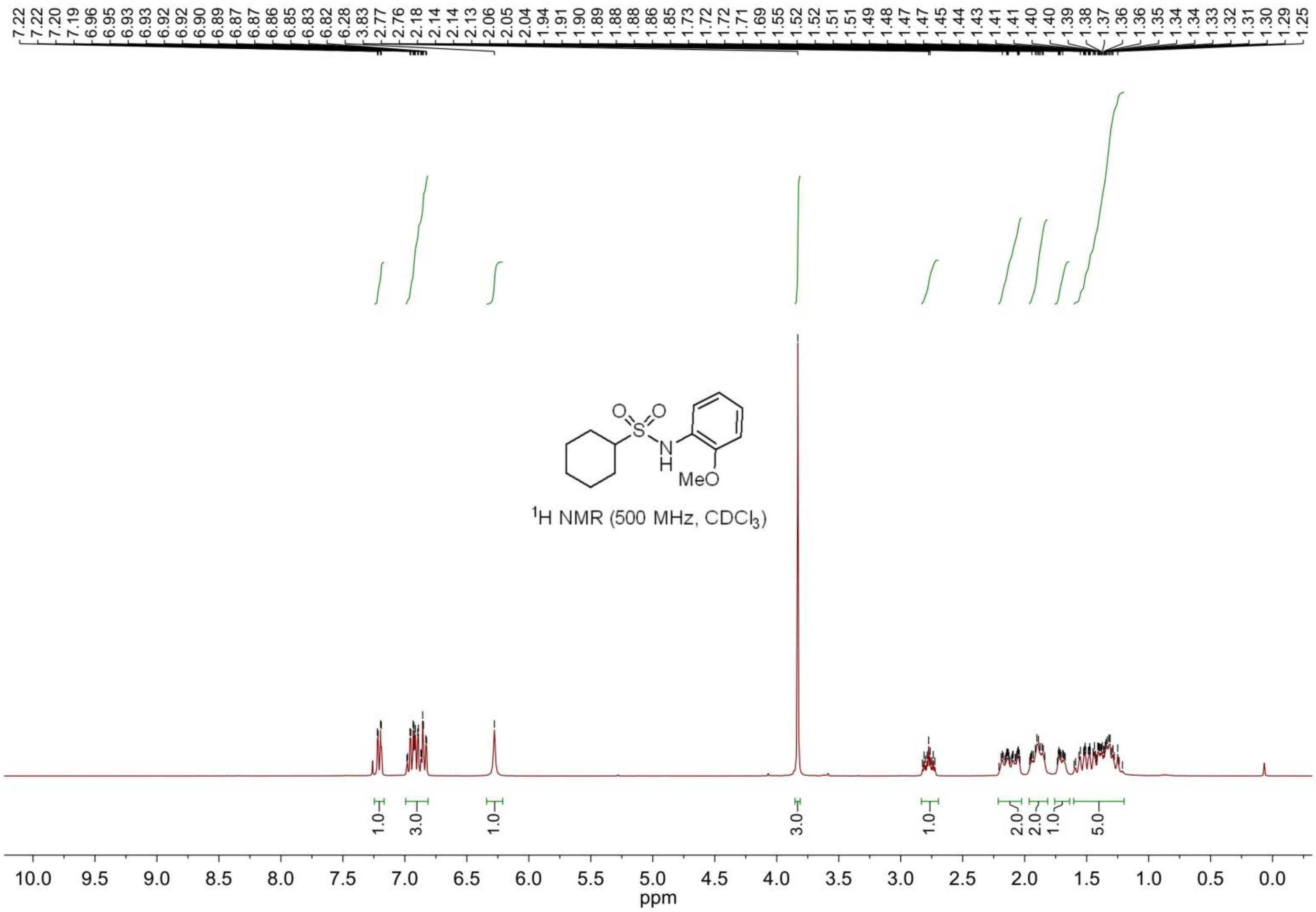

Go back to table of contents

Evaluation Only. Created with Aspose.Pdf. Copyright 2002-2014 Aspose Pty Ltd.

N-(2-Methoxyphenyl)cyclohexanesulfonamide (8e)

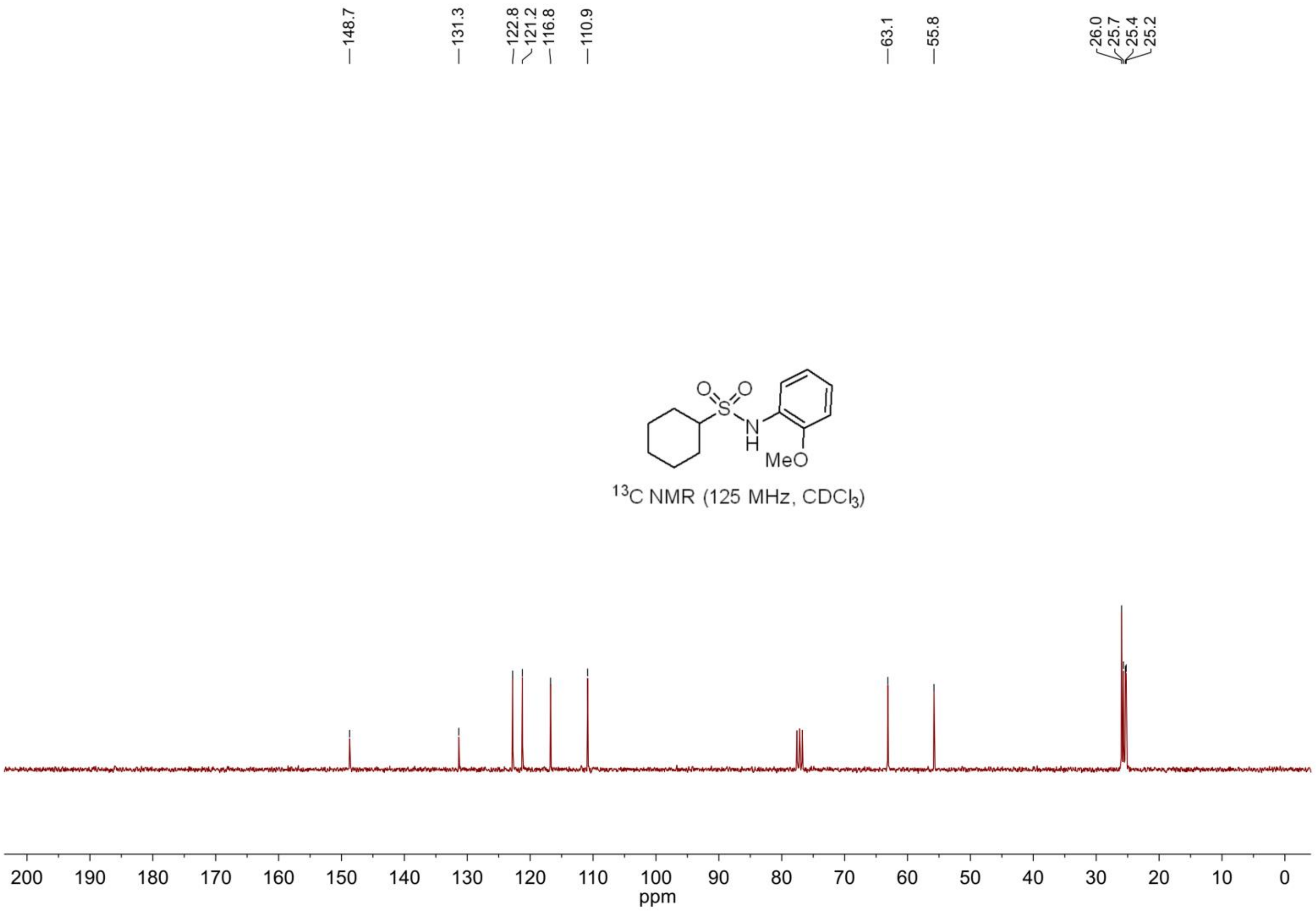

Go back to table of contents

Evaluation Only. Created with Aspose.Pdf. Copyright 2002-2014 Aspose Pty Ltd.

N-(4-(Trifluoromethoxy)phenyl)cyclohexanesulfonamide (8f)

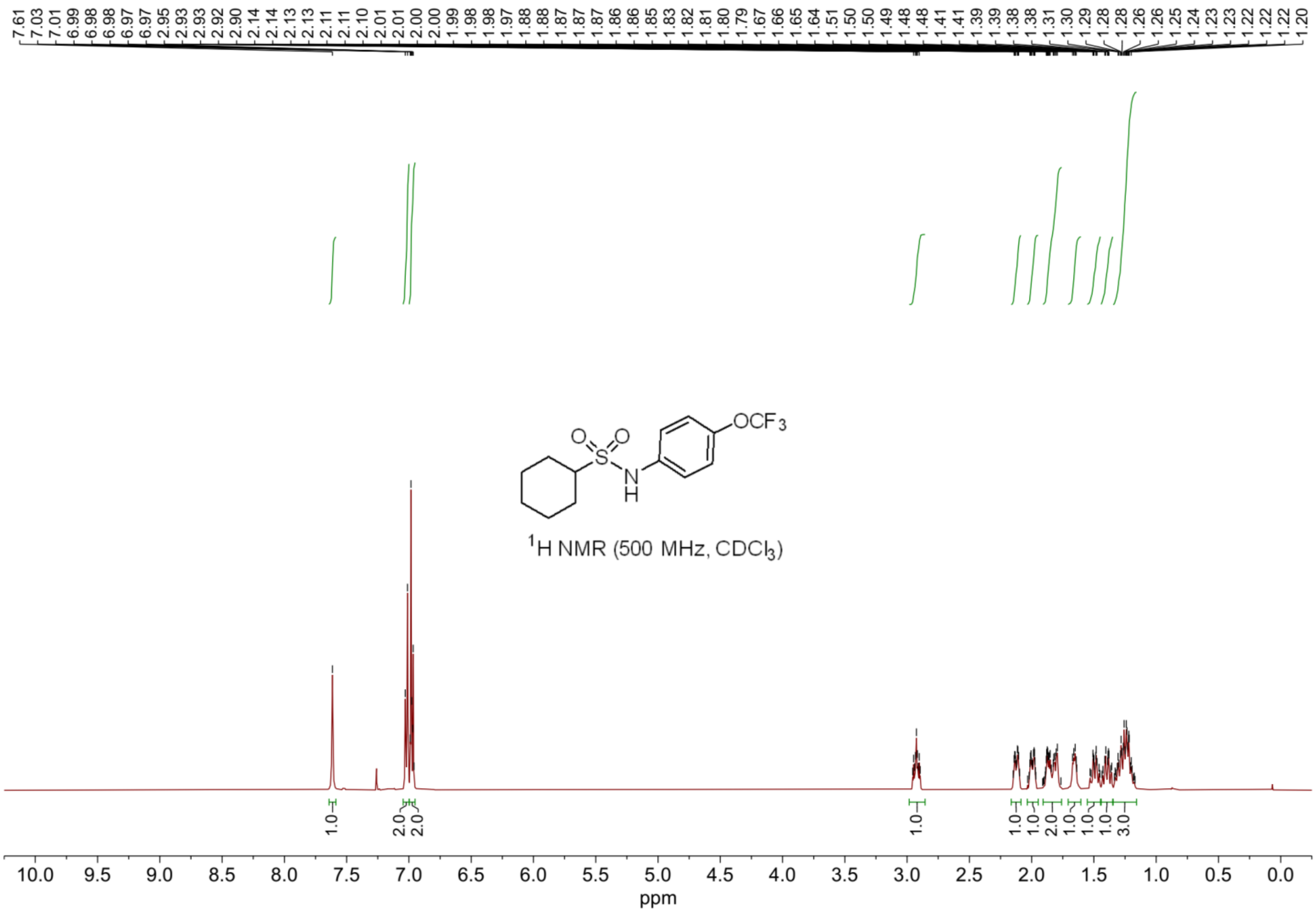

Go back to table of contents

Evaluation Only. Created with Aspose.Pdf. Copyright 2002-2014 Aspose Pty Ltd.

N-(4-(Trifluoromethoxy)phenyl)cyclohexanesulfonamide (8f)

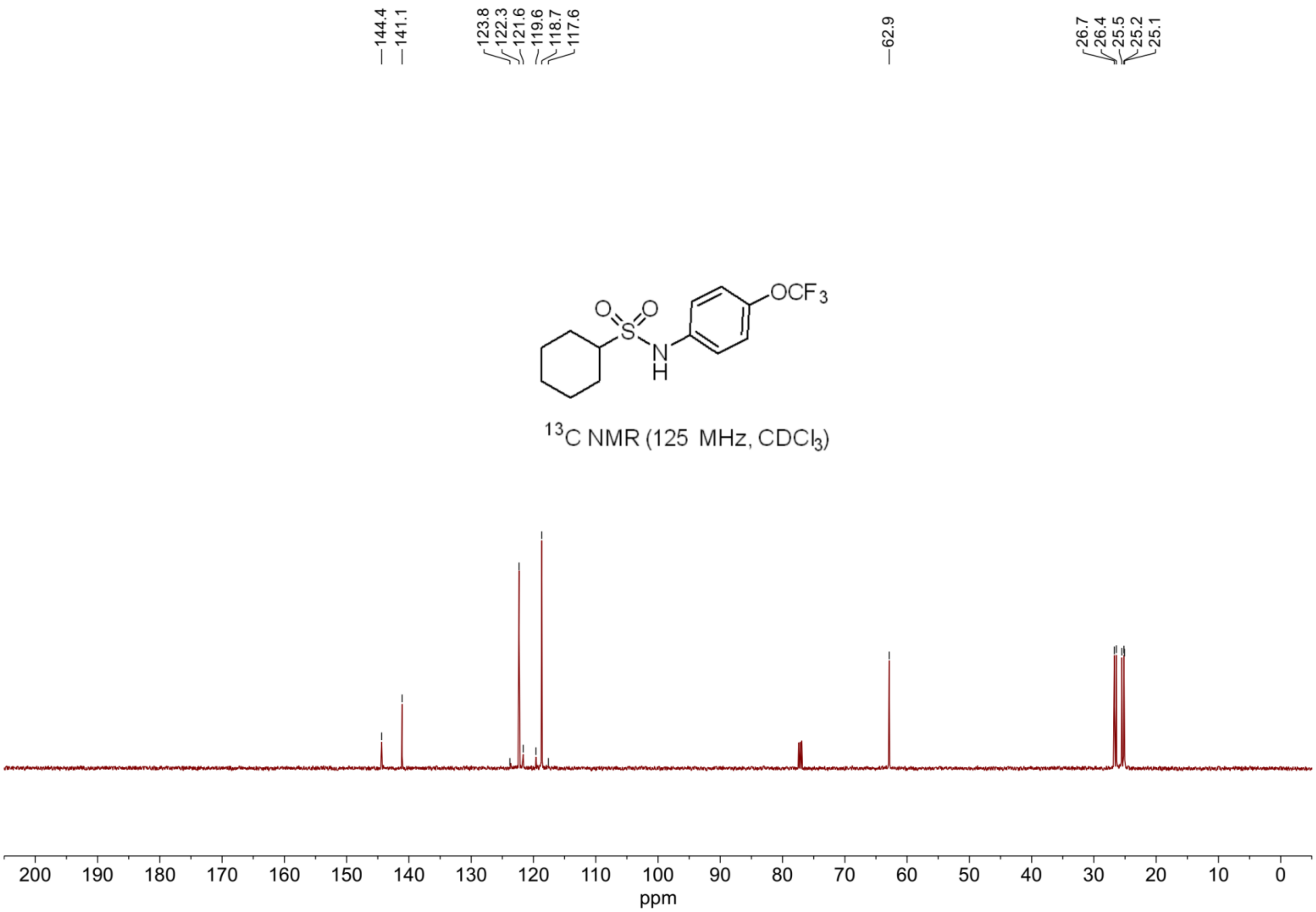

Go back to table of contents

Evaluation Only. Created with Aspose.Pdf. Copyright 2002-2014 Aspose Pty Ltd.

N-(3-(Methylthio)phenyl)cyclohexanesulfonamide (8g)

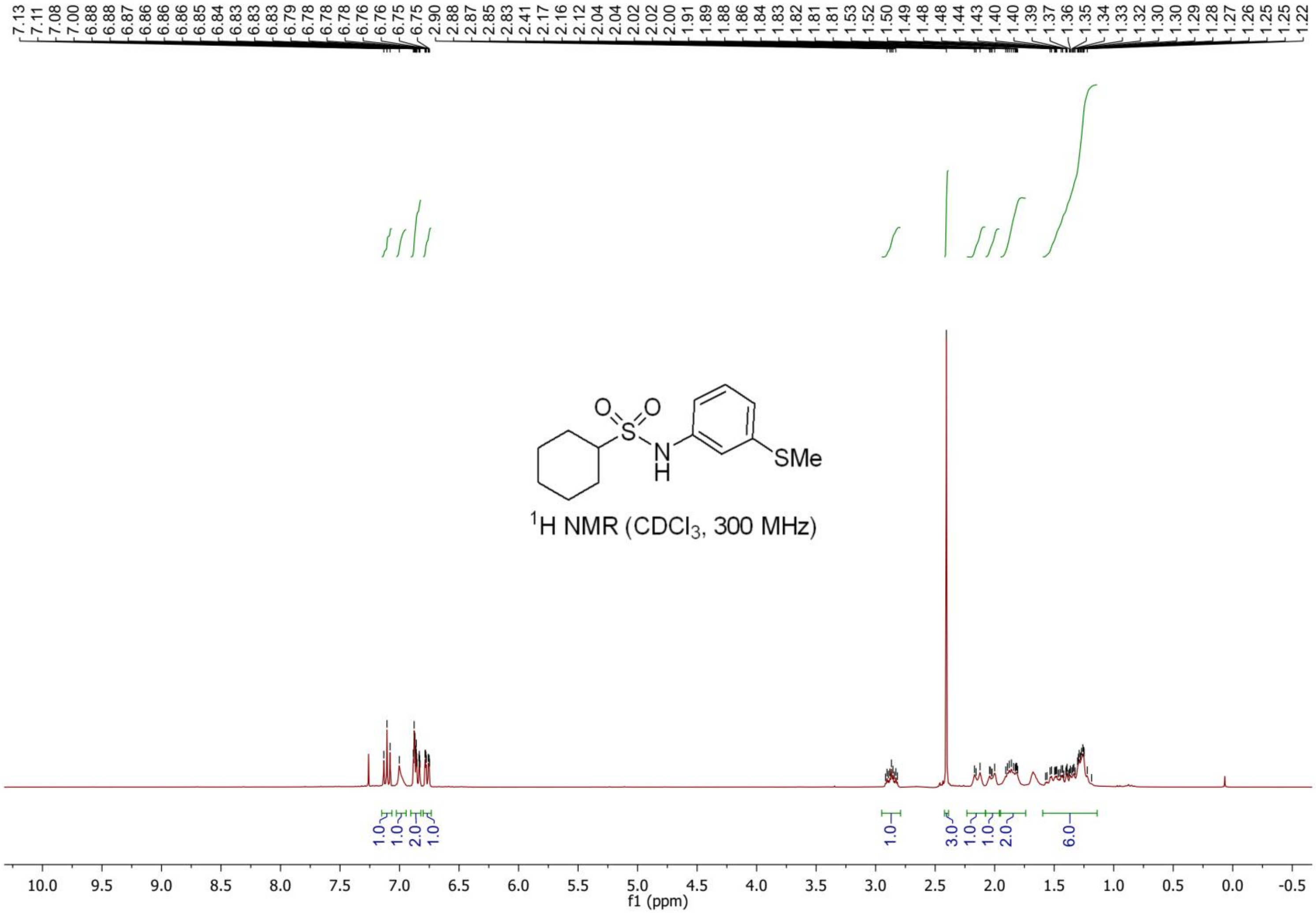

Go back to table of contents

Evaluation Only. Created with Aspose.Pdf. Copyright 2002-2014 Aspose Pty Ltd.

N-(3-(Methylthio)phenyl)cyclohexanesulfonamide (8g)

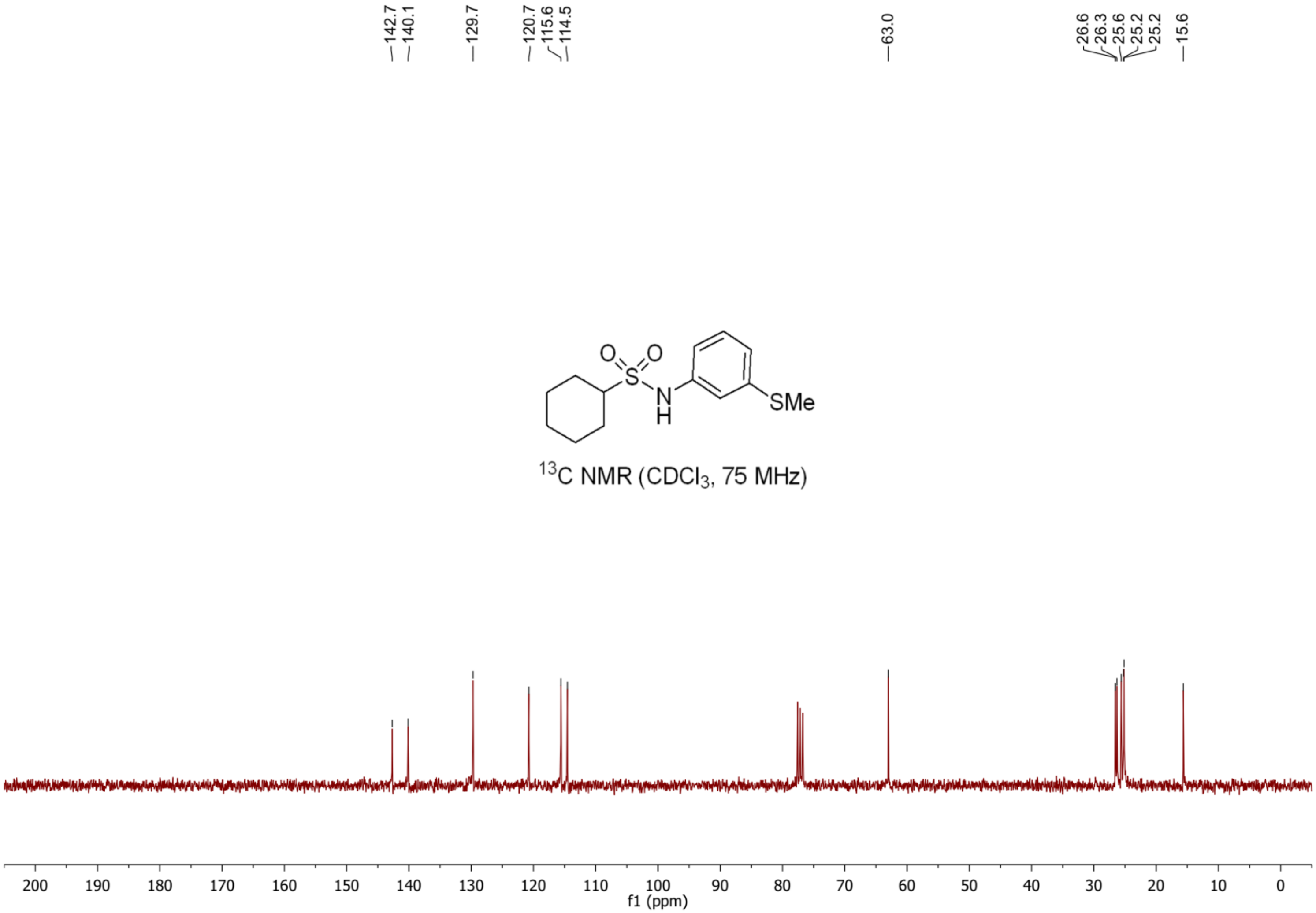

^{13}C NMR ($CDCl_3$, 75 MHz)

Go back to table of contents

Evaluation Only. Created with Aspose.Pdf. Copyright 2002-2014 Aspose Pty Ltd.

Ethyl 2-(4-(cyclohexanesulfonamido)phenyl)acetate (8h)

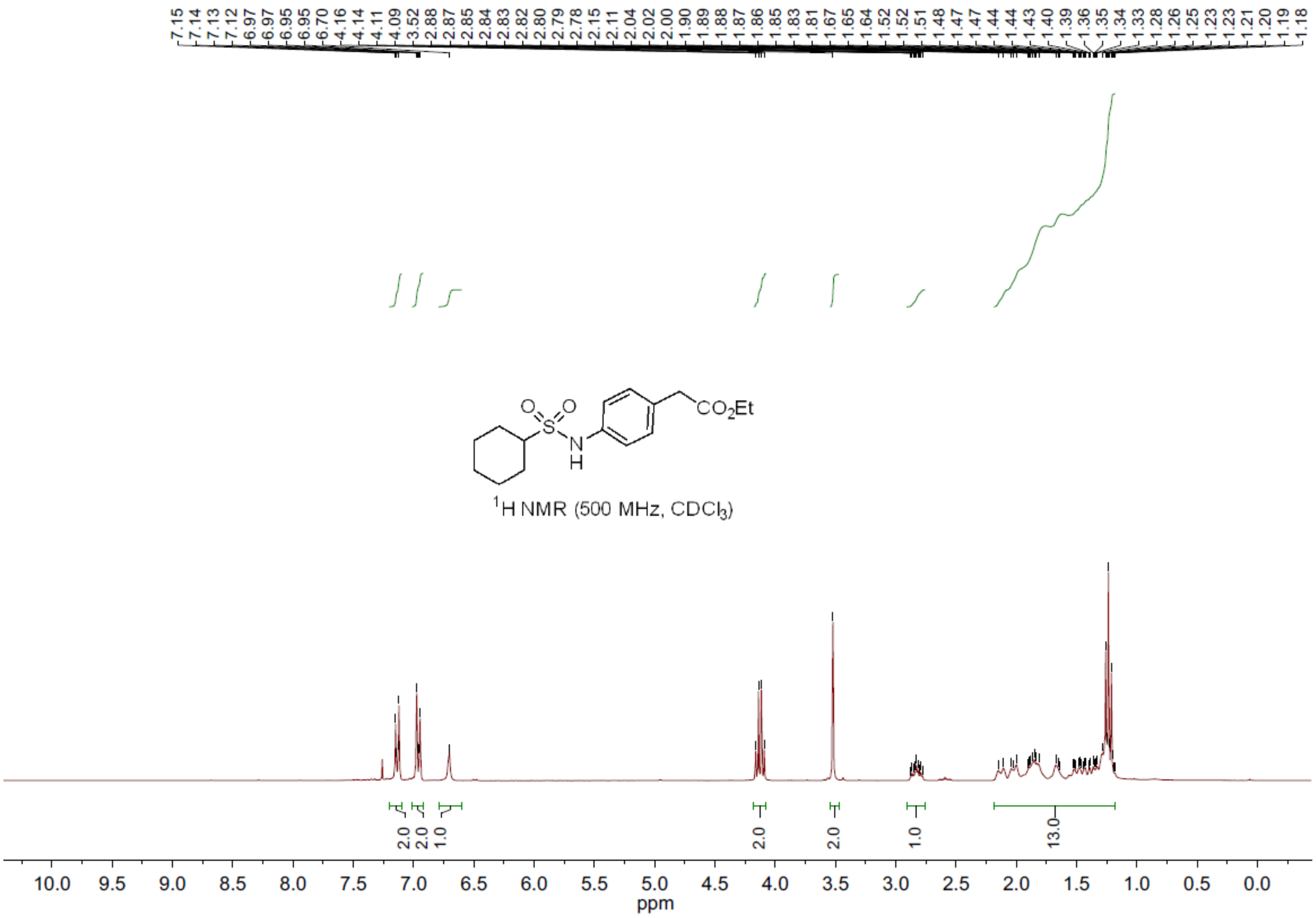

Go back to table of contents

Evaluation Only. Created with Aspose.Pdf. Copyright 2002-2014 Aspose Pty Ltd.

Ethyl 2-(4-(cyclohexanesulfonamido)phenyl)acetate (8h)

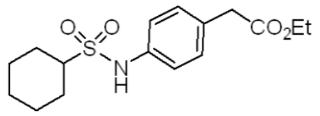

^{13}C NMR (125 MHz, $CDCl_3$)

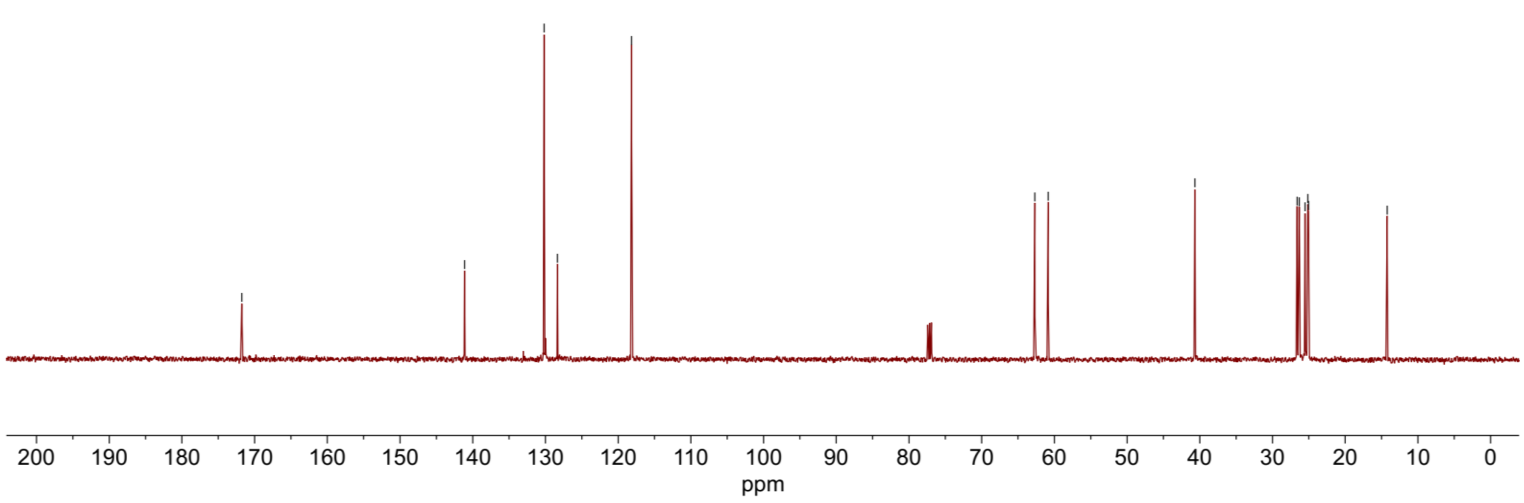

Go back to table of contents

Evaluation Only. Created with Aspose.Pdf. Copyright 2002-2014 Aspose Pty Ltd.

N-Methyl-*N*-(*p*-tolyl)cyclohexanesulfonamide (8i)

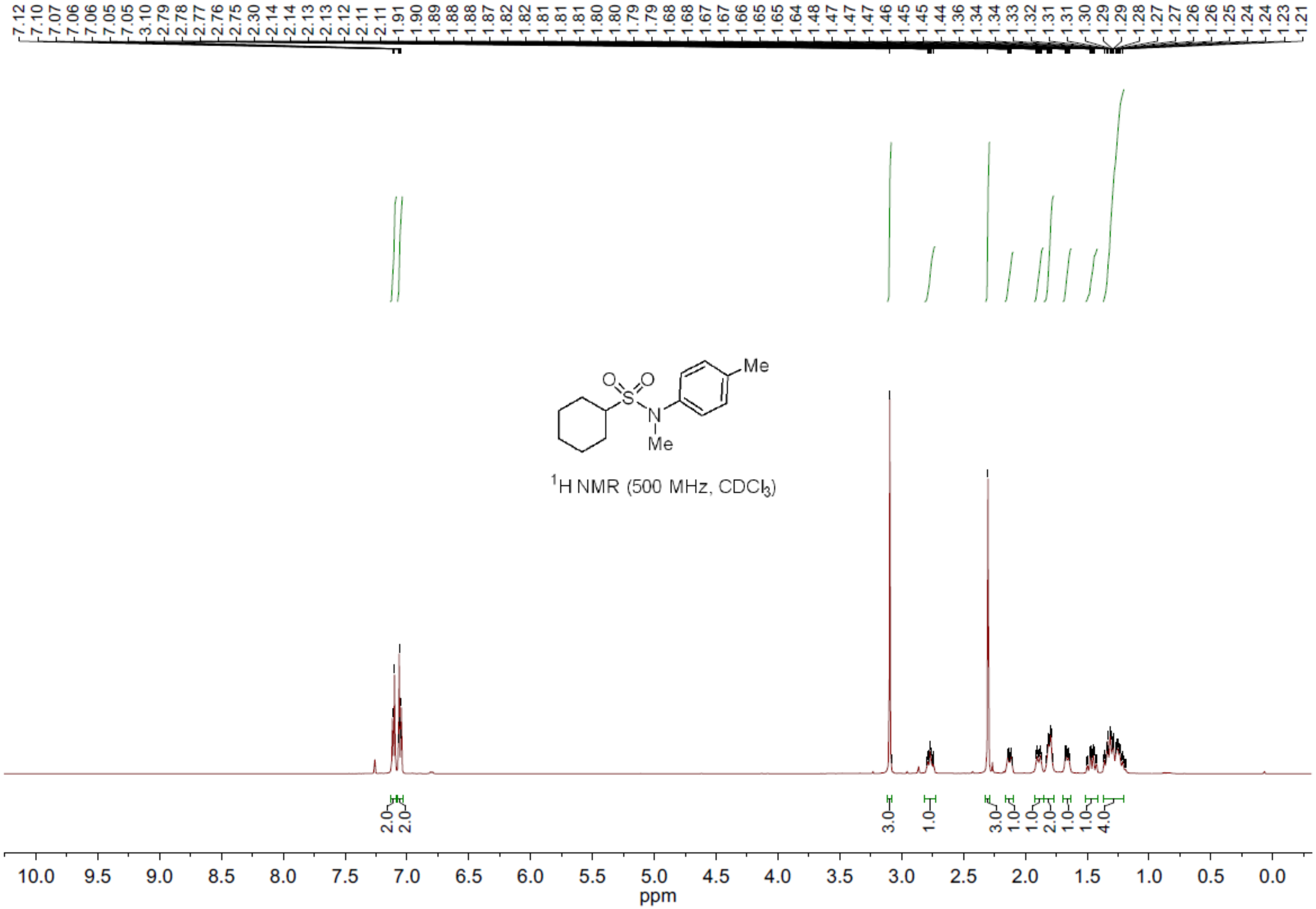

Evaluation Only. Created with Aspose.Pdf. Copyright 2002-2014 Aspose Pty Ltd.

N-Methyl-*N*-(*p*-tolyl)cyclohexanesulfonamide (8i)

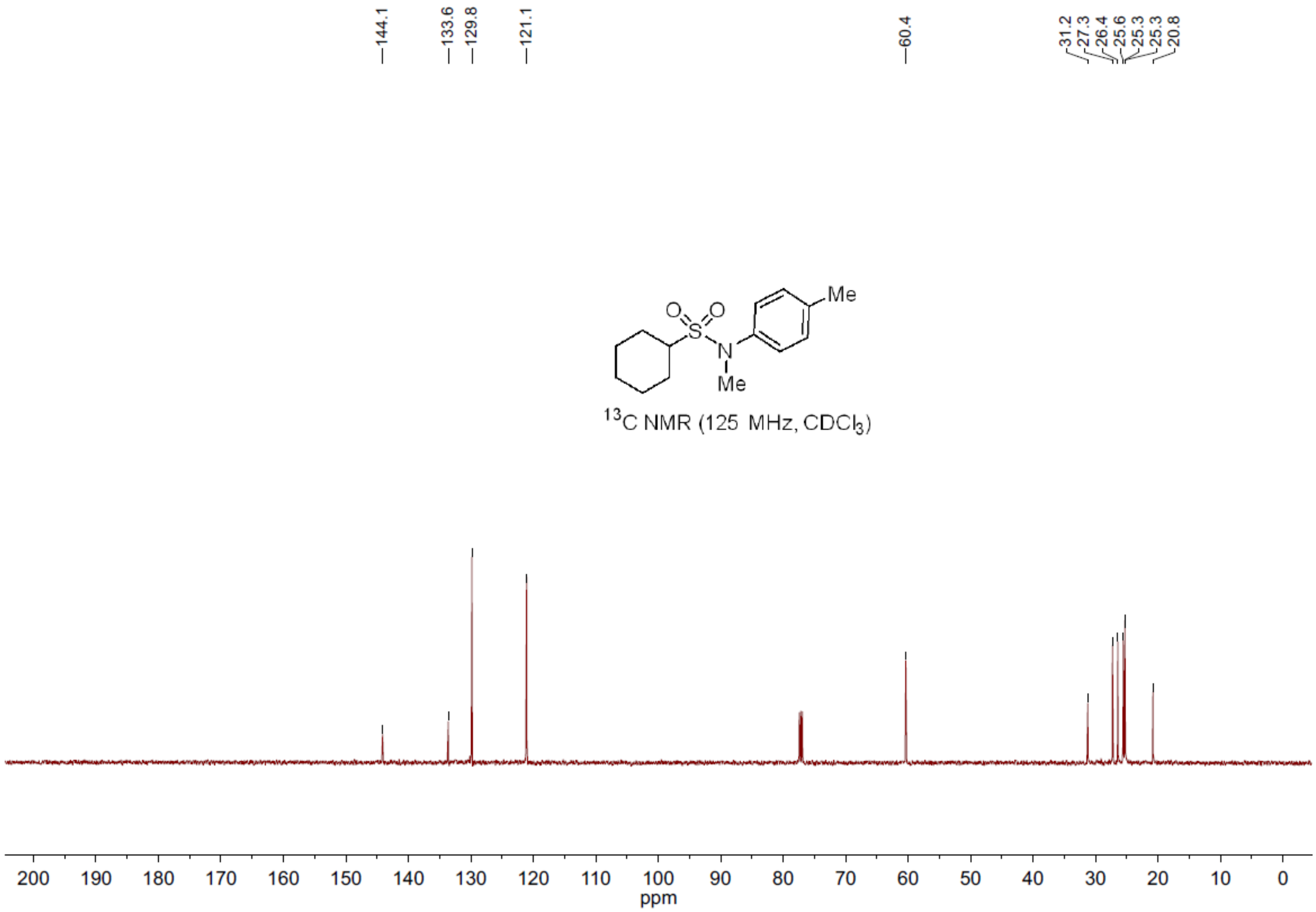

Go back to table of contents

Evaluation Only. Created with Aspose.Pdf. Copyright 2002-2014 Aspose Pty Ltd.

N-(4-Fluoro-2-methylphenyl)cyclohexanesulfonamide (8j)

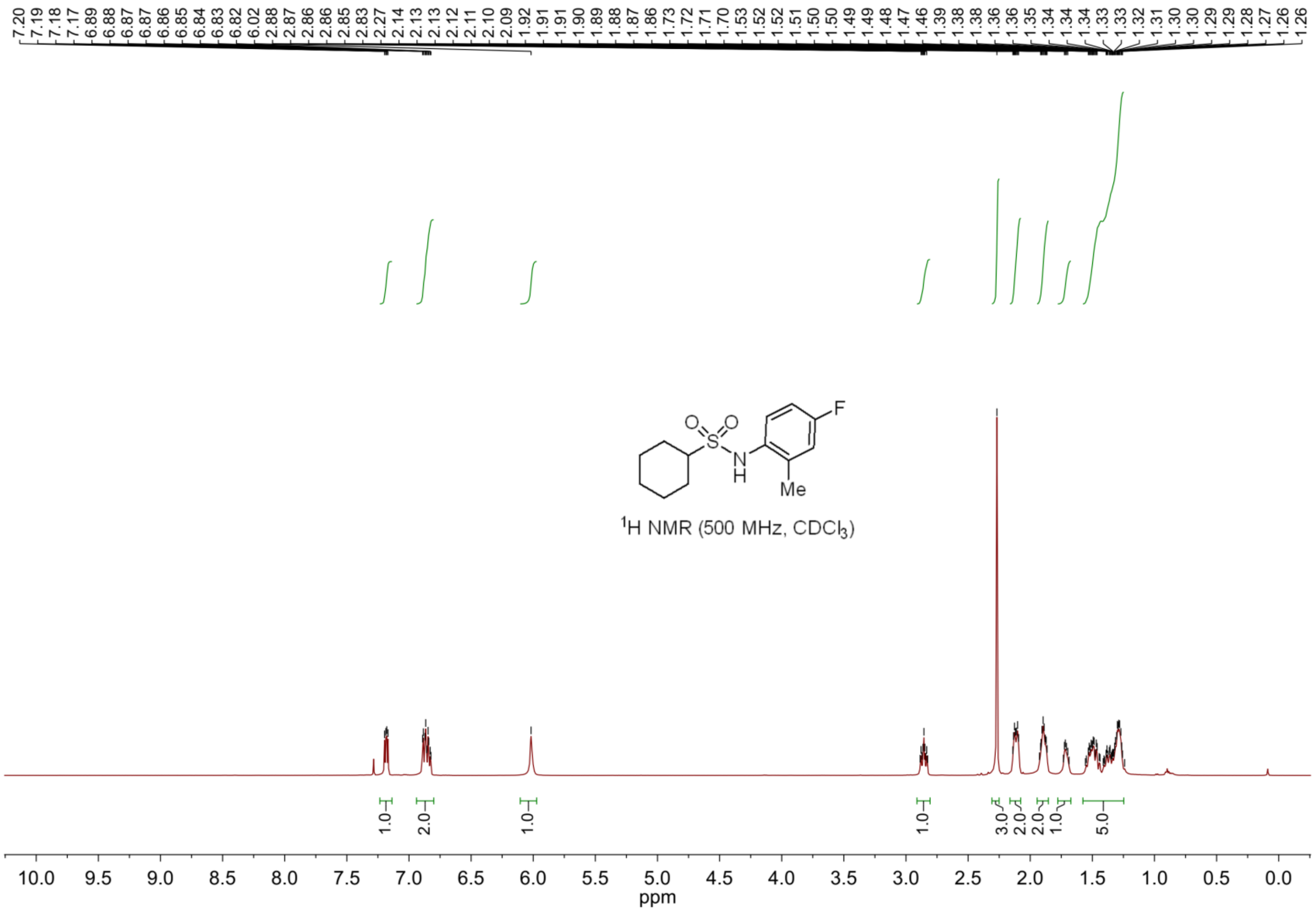

Go back to table of contents

Evaluation Only. Created with Aspose.Pdf. Copyright 2002-2014 Aspose Pty Ltd.

N-(4-Fluoro-2-methylphenyl)cyclohexanesulfonamide (8j)

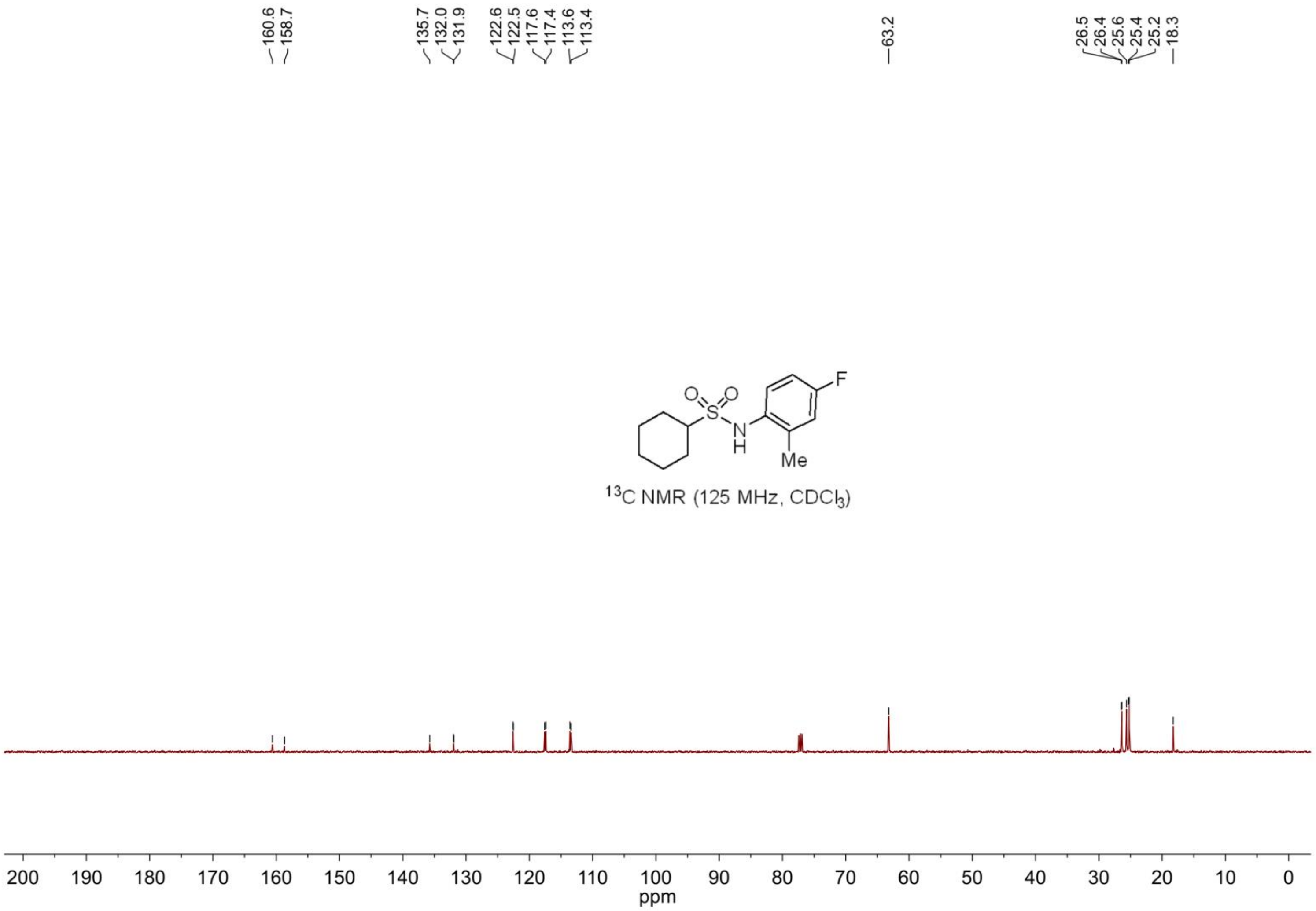

Go back to table of contents

Evaluation Only. Created with Aspose.Pdf. Copyright 2002-2014 Aspose Pty Ltd.

1-(Cyclohexylsulfonyl)indoline (8k)

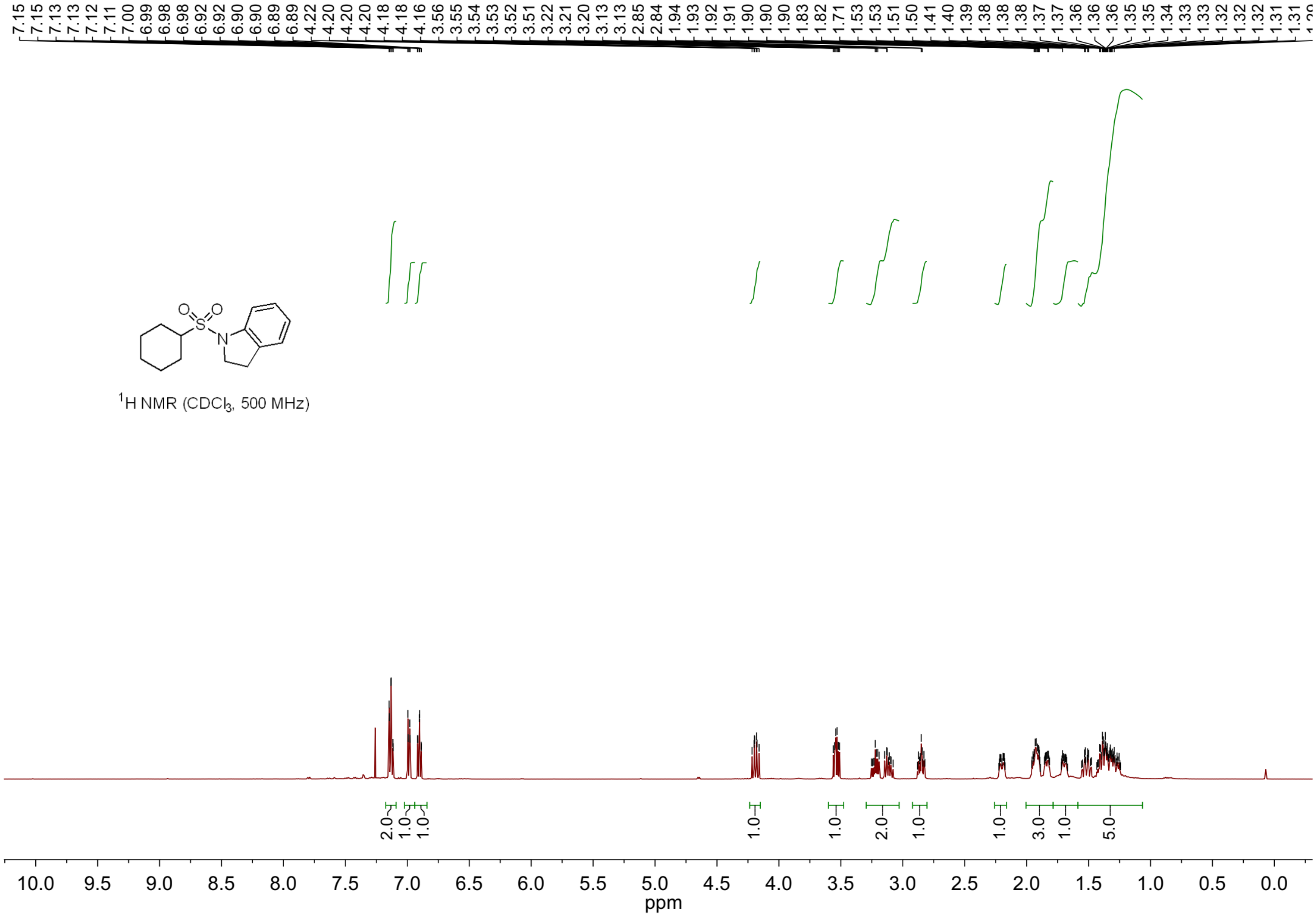

Go back to table of contents

Evaluation Only. Created with Aspose.Pdf. Copyright 2002-2014 Aspose Pty Ltd.

1-(Cyclohexylsulfonyl)indoline (8k)

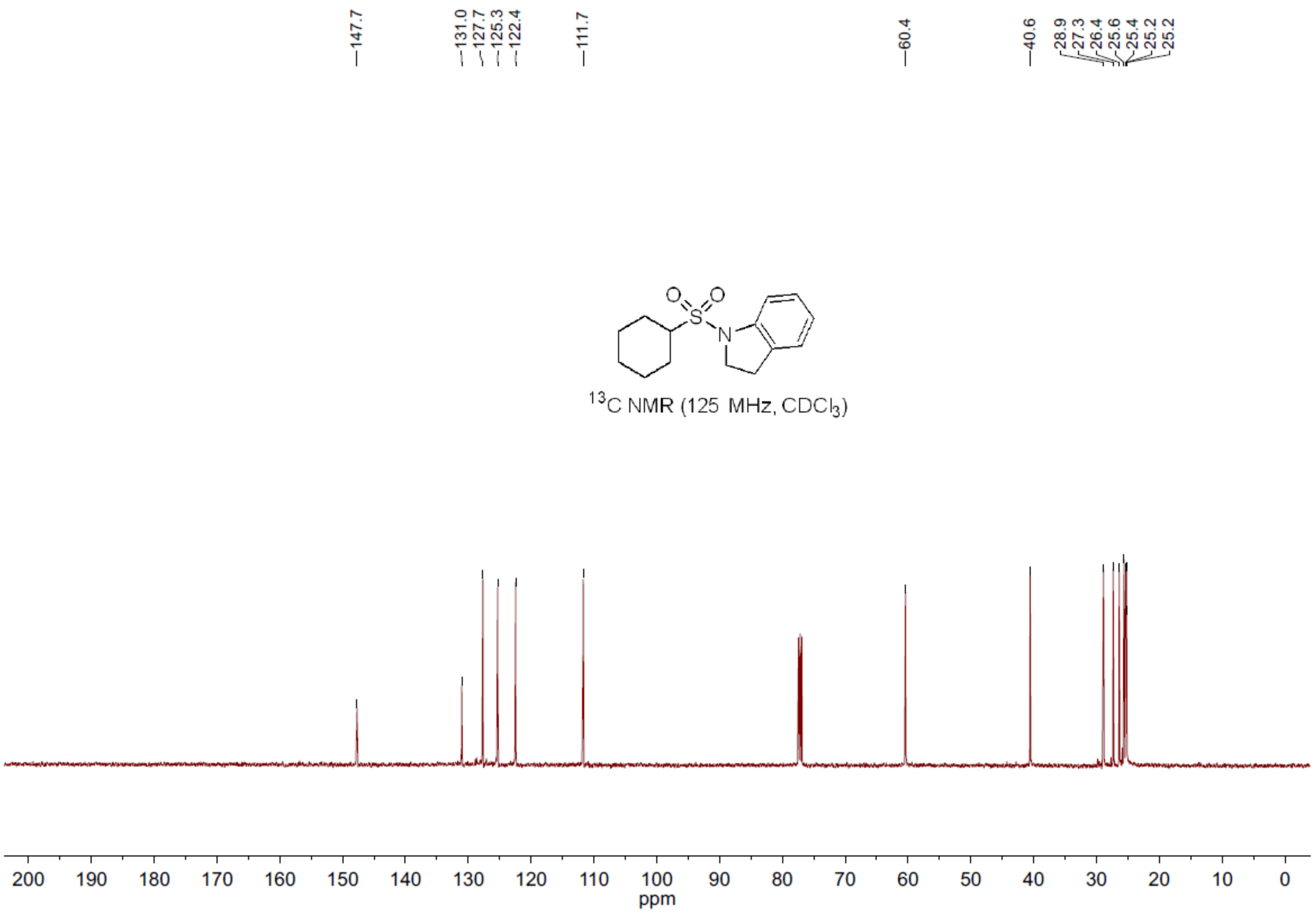

Go back to table of contents

Evaluation Only. Created with Aspose.Pdf. Copyright 2002-2014 Aspose Pty Ltd.

4-Phenyl-*N*-(3,4,5-trimethylphenyl)butane-1-sulfonamide (8l)

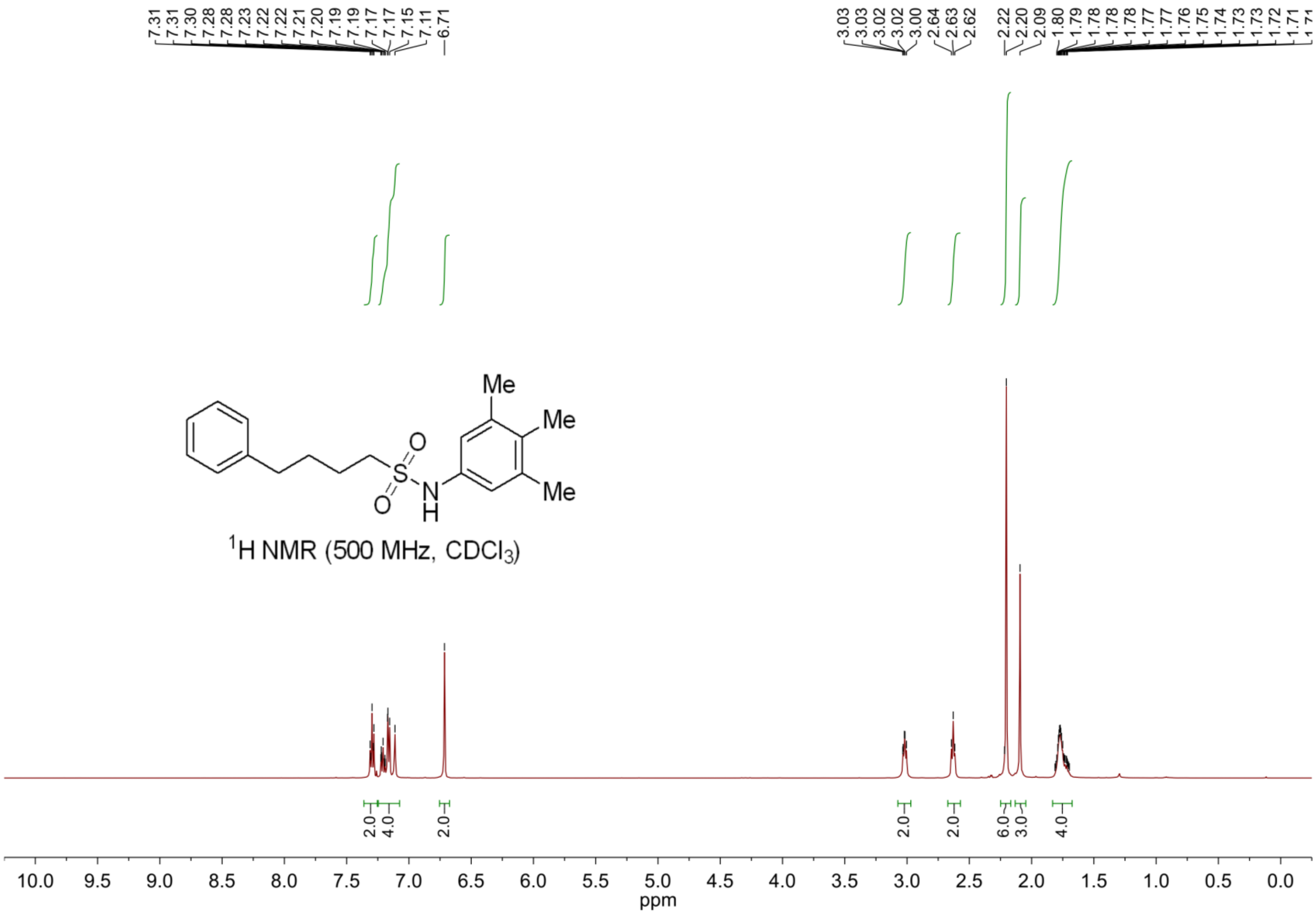

Go back to table of contents

Evaluation Only. Created with Aspose.Pdf. Copyright 2002-2014 Aspose Pty Ltd.

4-Phenyl-*N*-(3,4,5-trimethylphenyl)butane-1-sulfonamide (8l)

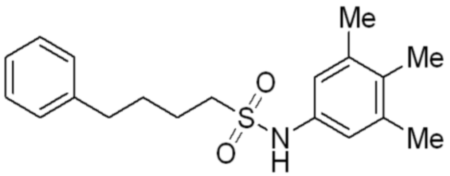

^{13}C NMR (125 MHz, $CDCl_3$)

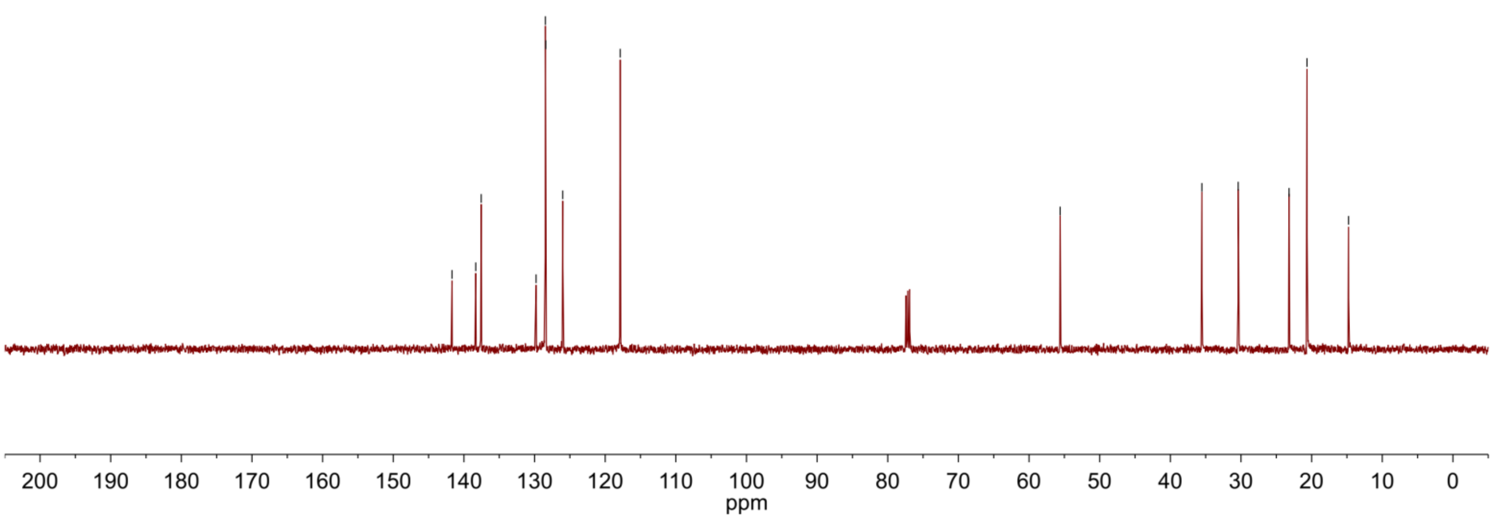

Go back to table of contents

Evaluation Only. Created with Aspose.Pdf. Copyright 2002-2014 Aspose Pty Ltd.

N-(3-isopropylphenyl)butane-1-sulfonamide (8m)

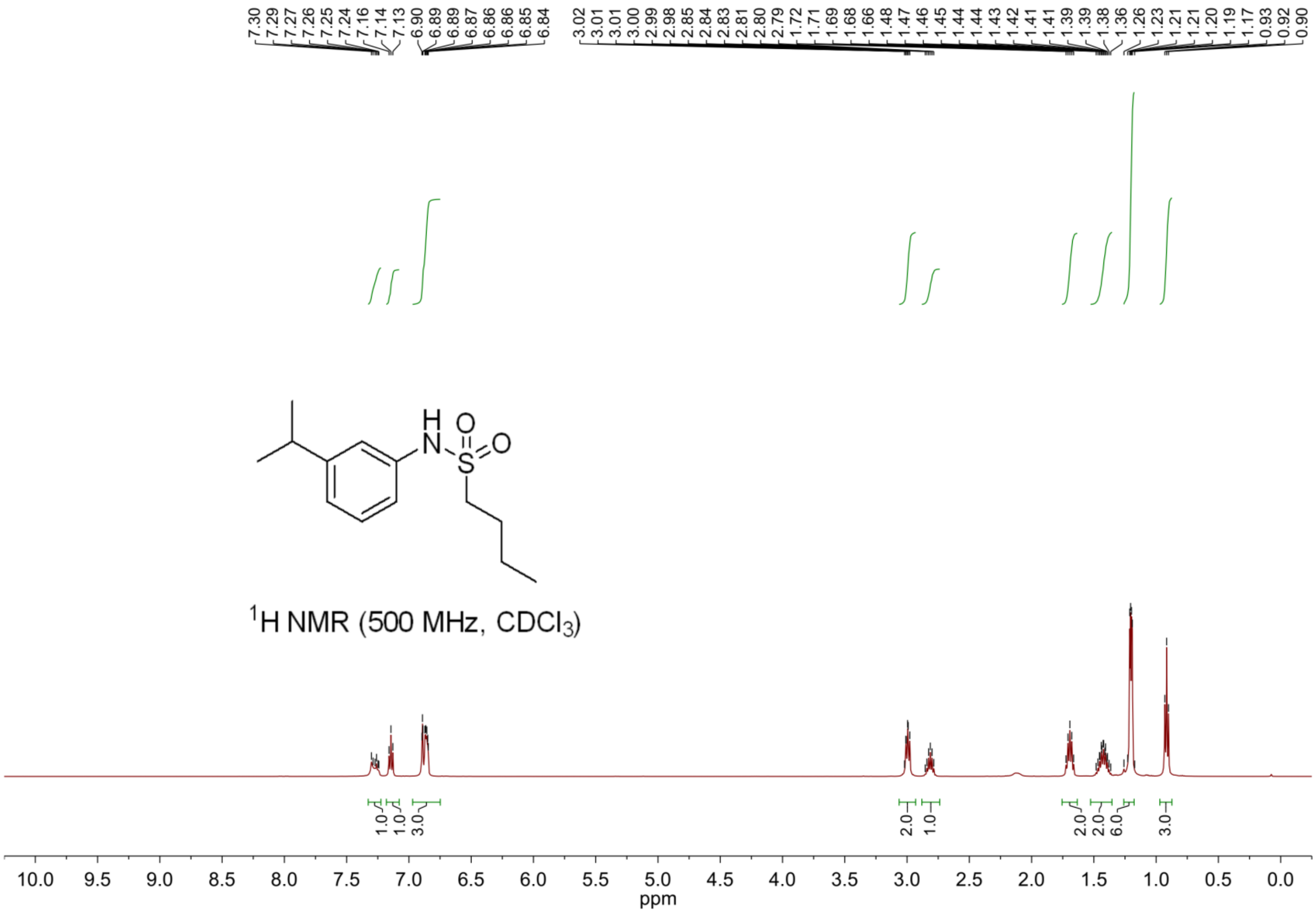

Go back to table of contents

Evaluation Only. Created with Aspose.Pdf. Copyright 2002-2014 Aspose Pty Ltd.

N-(3-isopropylphenyl)butane-1-sulfonamide (8m)

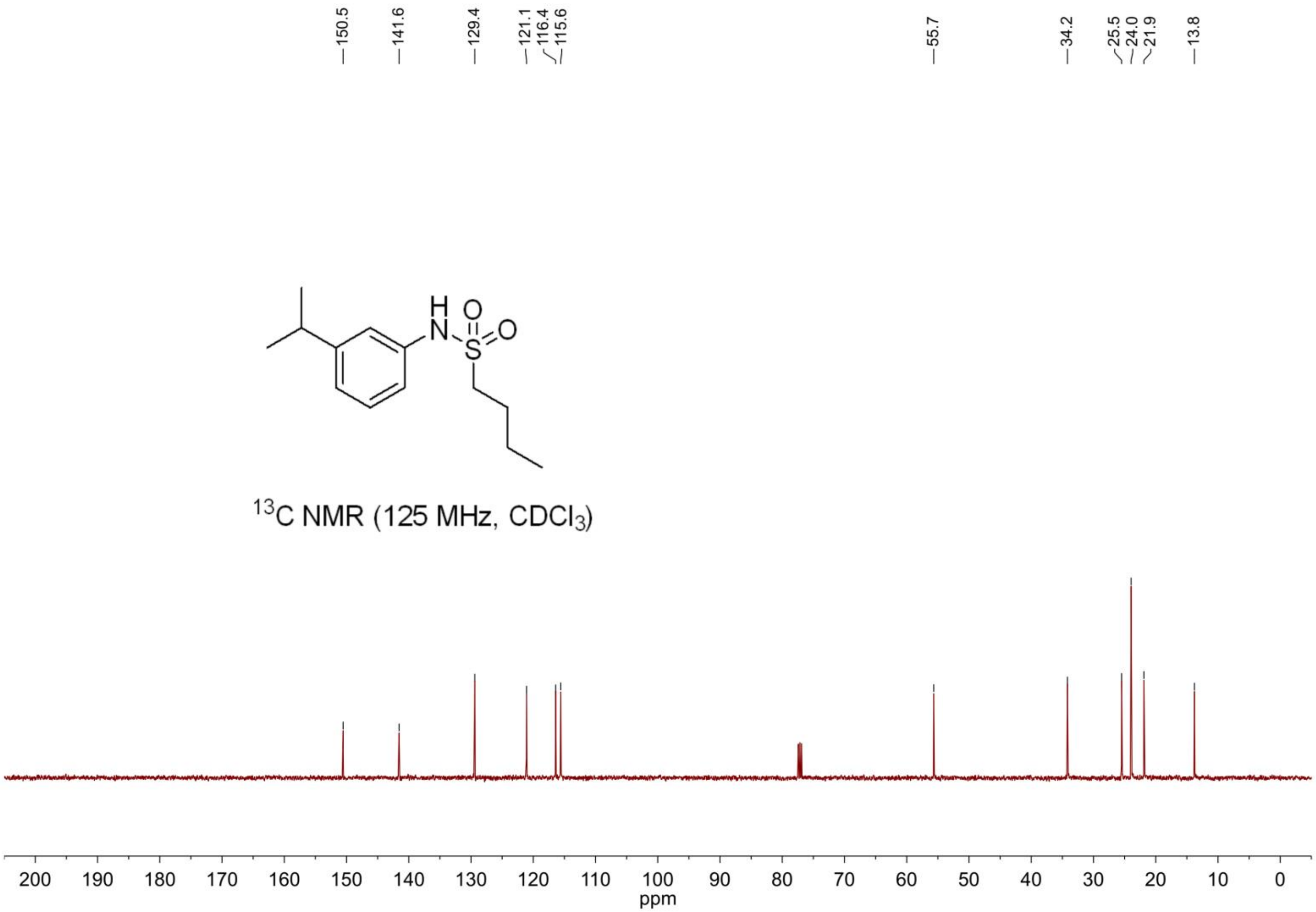

Go back to table of contents

Evaluation Only. Created with Aspose.Pdf. Copyright 2002-2014 Aspose Pty Ltd.

N-(3-acetylphenyl)butane-1-sulfonamide (8n)

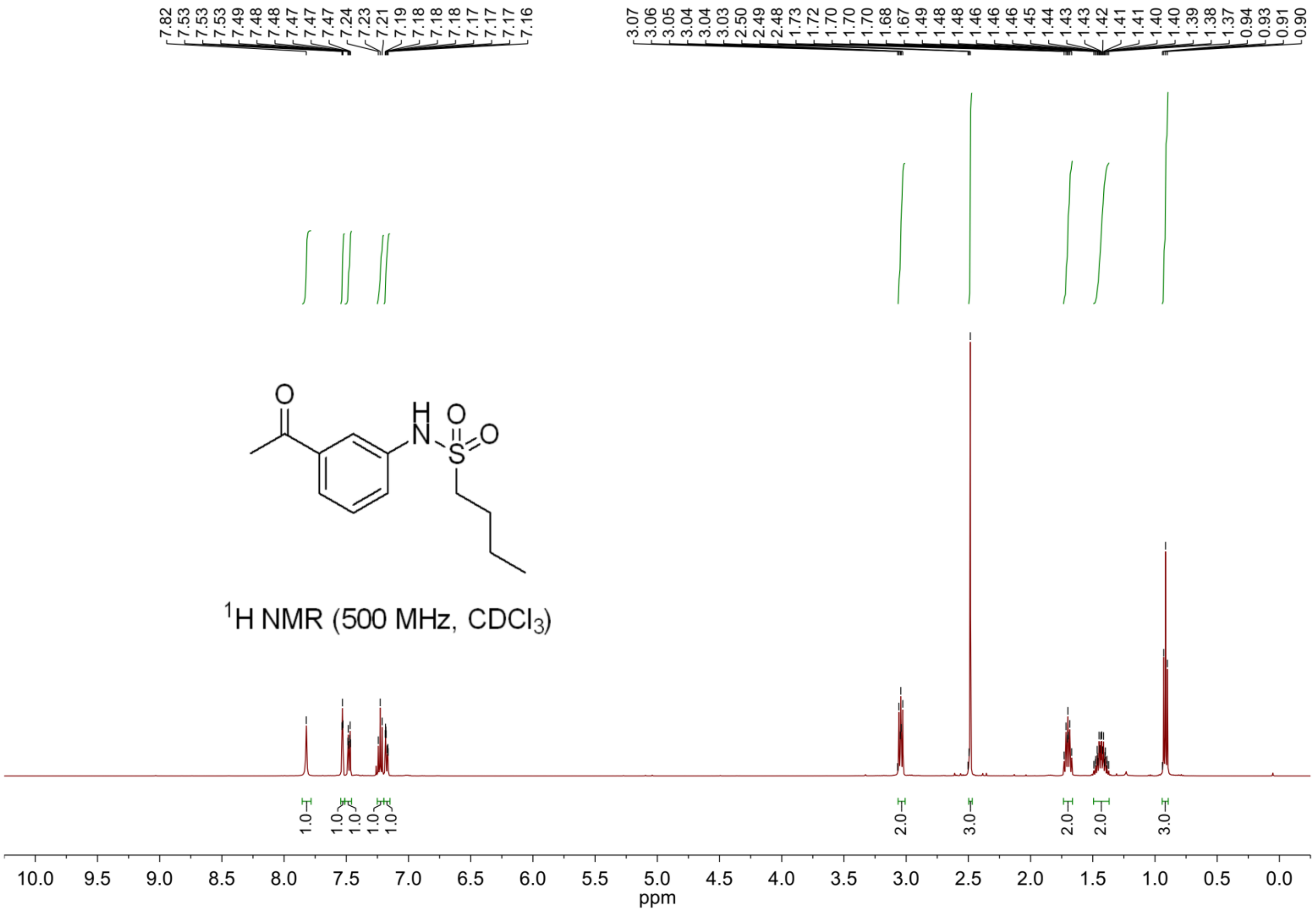

Go back to table of contents

Evaluation Only. Created with Aspose.Pdf. Copyright 2002-2014 Aspose Pty Ltd.

N-(3-acetylphenyl)butane-1-sulfonamide (8n)

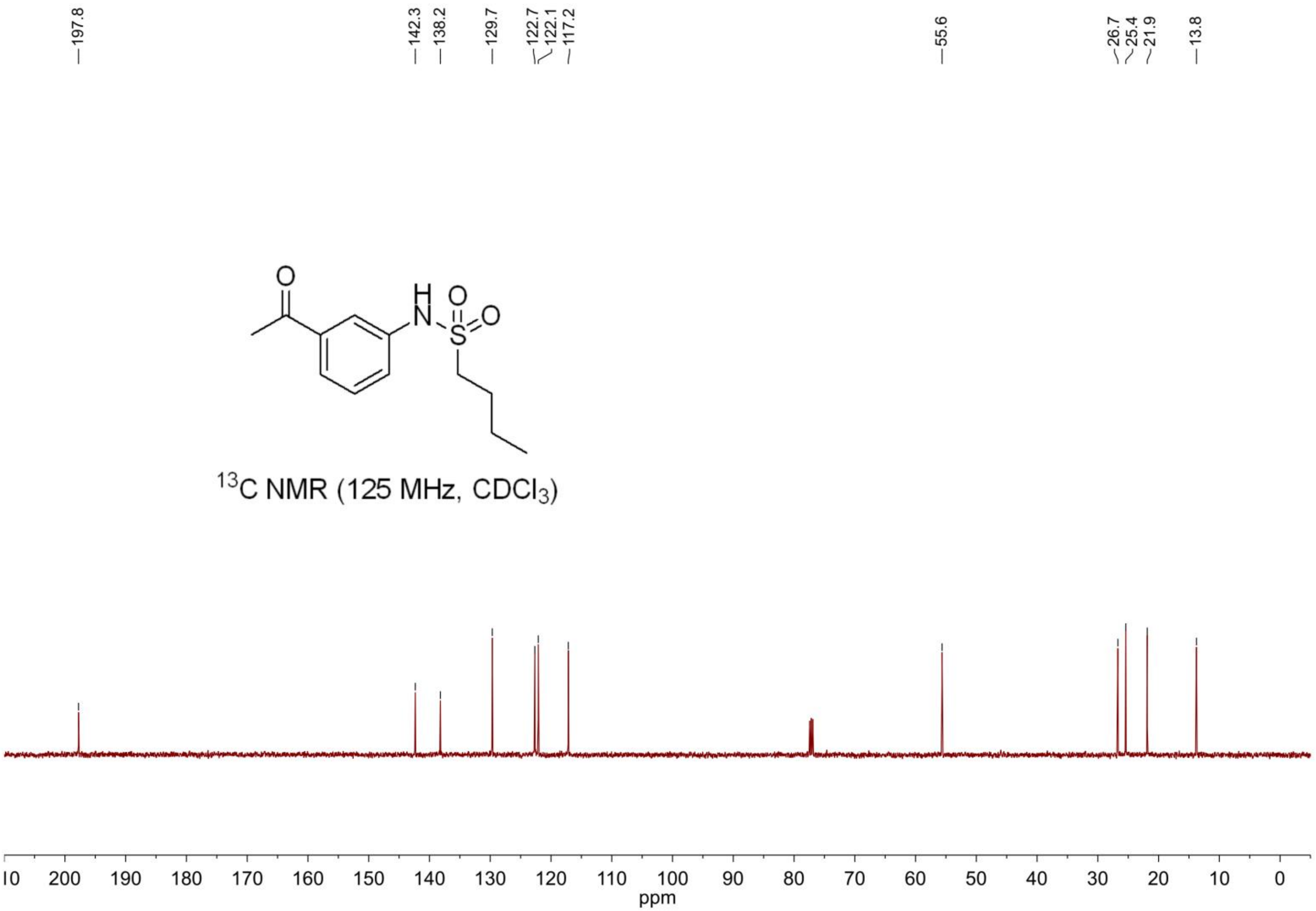

Go back to table of contents

Evaluation Only. Created with Aspose.Pdf. Copyright 2002-2014 Aspose Pty Ltd.

1-Cyclopentyl-*N*-(2,3-dihydro-1*H*-inden-5-yl)methanesulfonamide (8o)

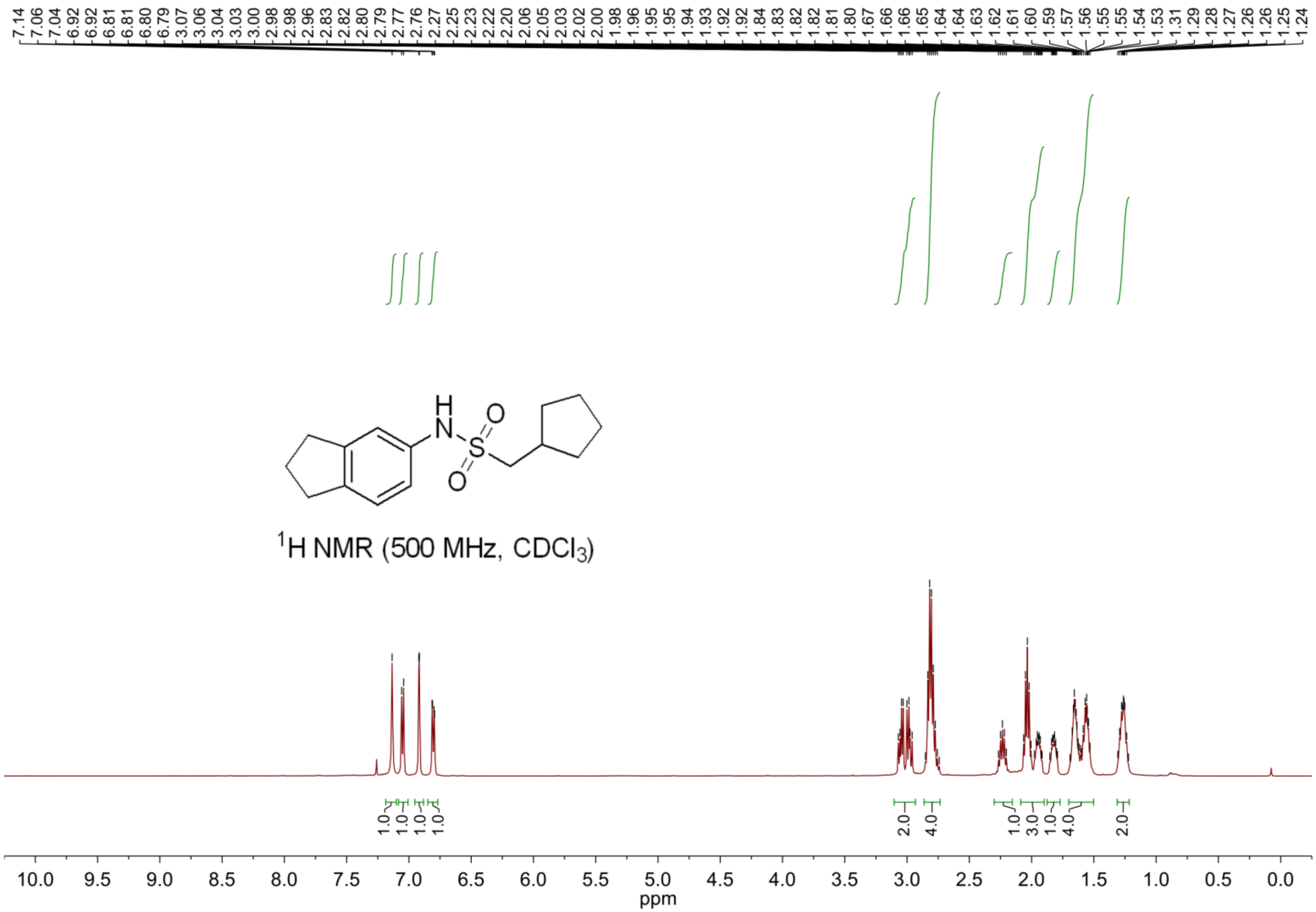

Go back to table of contents

Evaluation Only. Created with Aspose.Pdf. Copyright 2002-2014 Aspose Pty Ltd.

1-Cyclopentyl-*N*-(2,3-dihydro-1*H*-inden-5-yl)methanesulfonamide (8o)

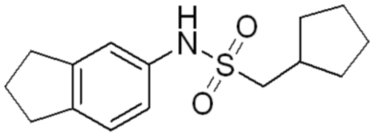

^{13}C NMR (125 MHz, CDCl$_3$)

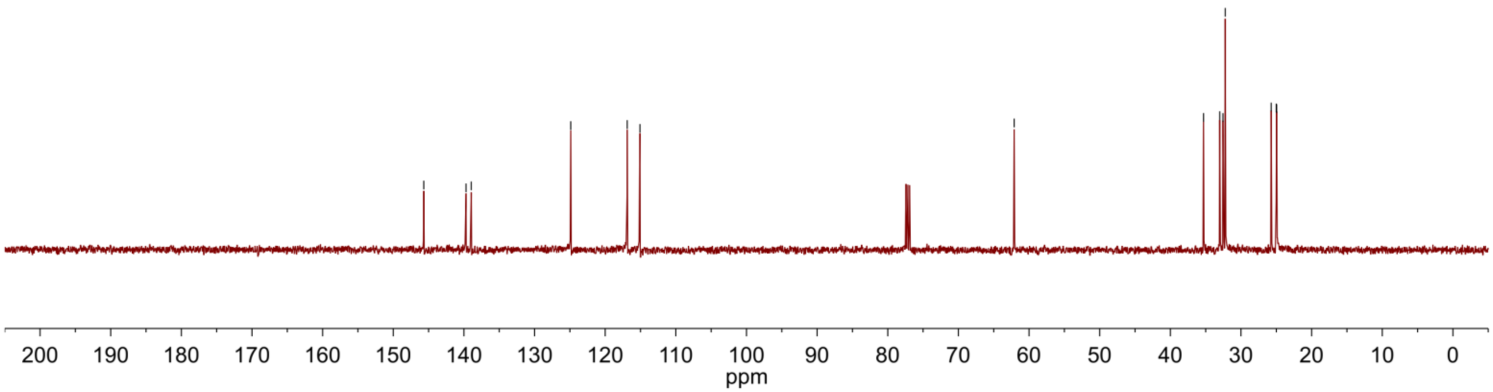

Go back to table of contents

Evaluation Only. Created with Aspose.Pdf. Copyright 2002-2014 Aspose Pty Ltd.

N-(2-(Methylthio)phenyl)tetrahydro-2*H*-pyran-4-sulfonamide (8p)

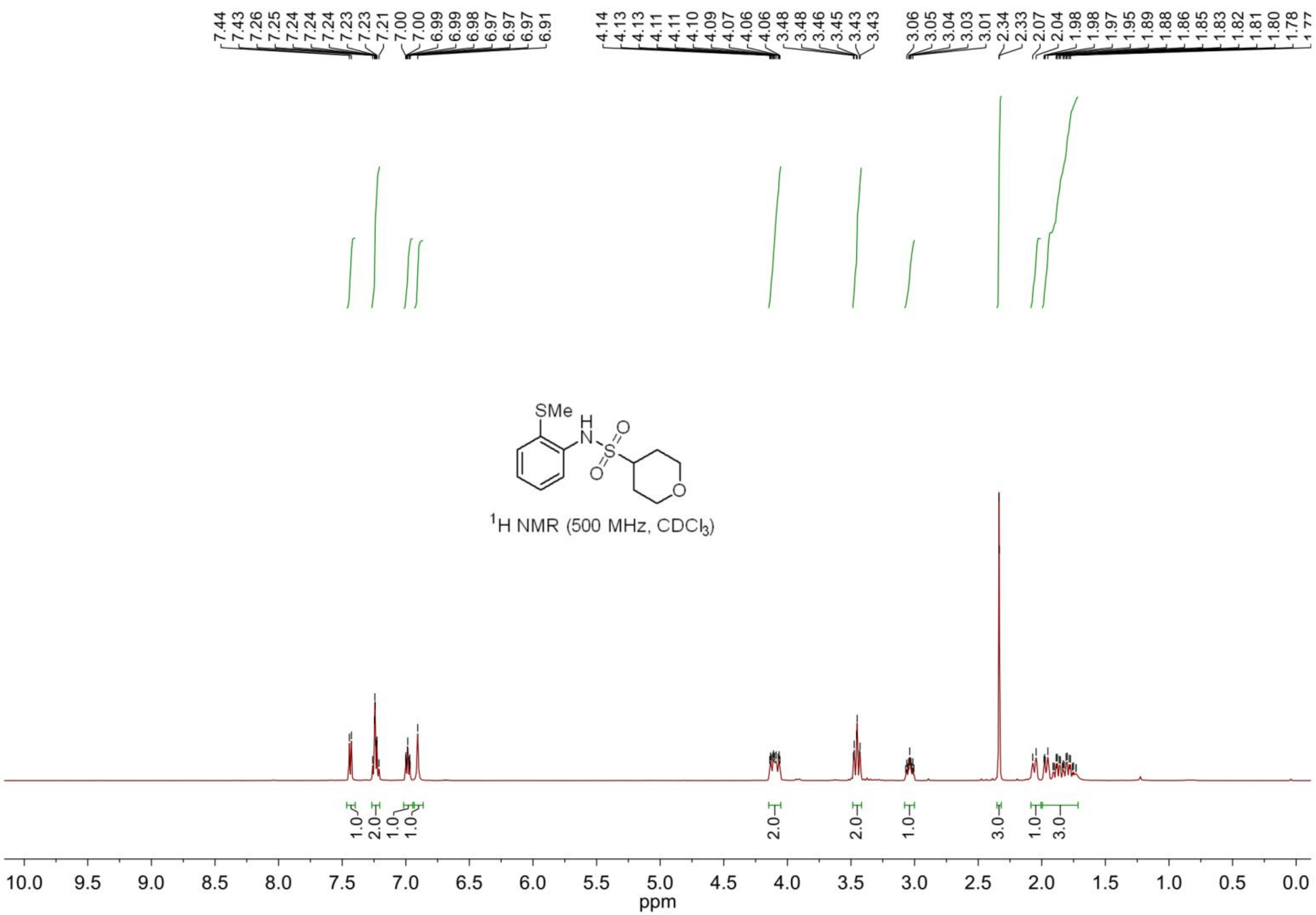

Go back to table of contents

Evaluation Only. Created with Aspose.Pdf. Copyright 2002-2014 Aspose Pty Ltd.

N-(2-(Methylthio)phenyl)tetrahydro-2*H*-pyran-4-sulfonamide (8p)

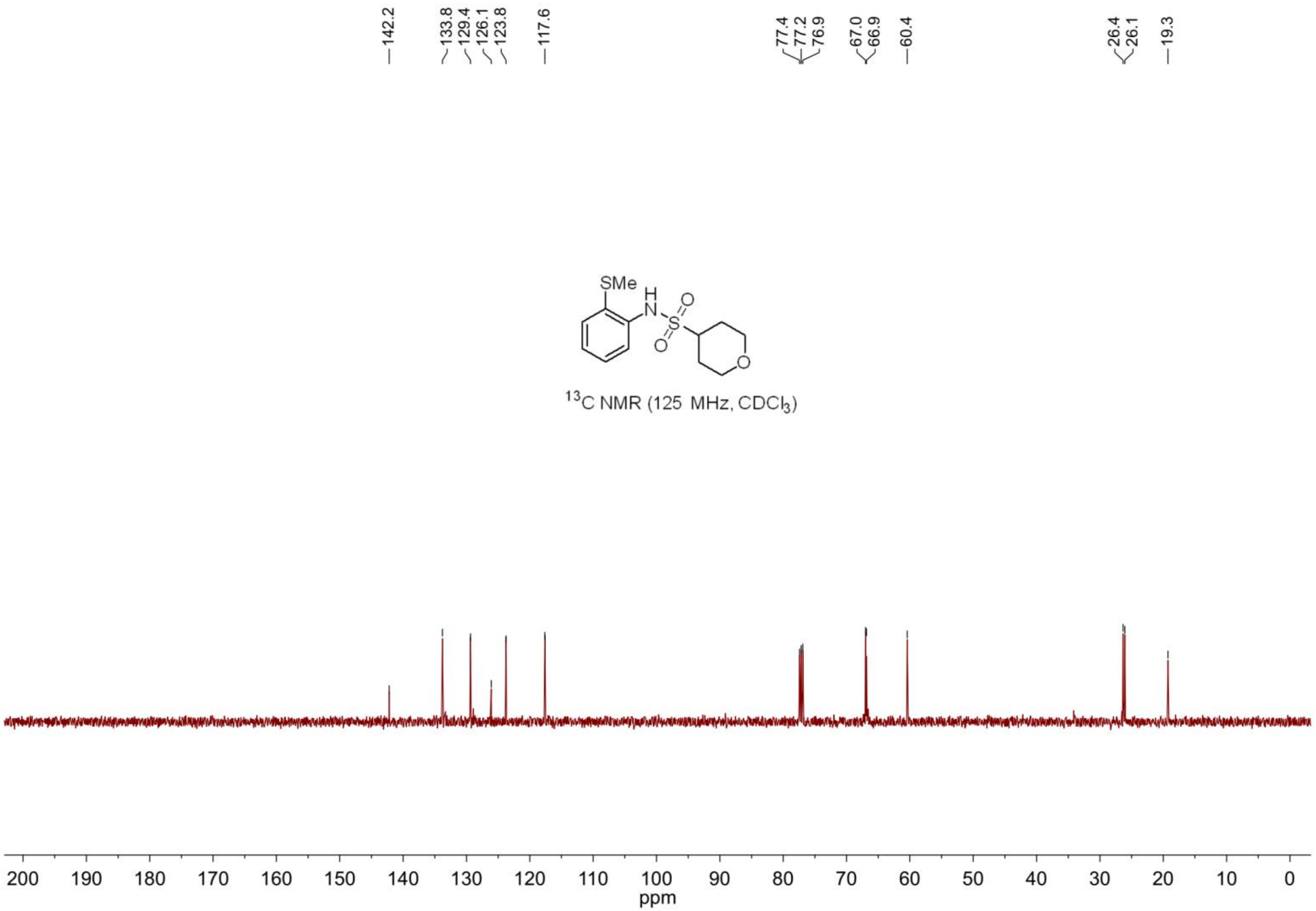

Go back to table of contents

Evaluation Only. Created with Aspose.Pdf. Copyright 2002-2014 Aspose Pty Ltd.

N-(3,5-dimethylphenyl)heptane-4-sulfonamide (8q)

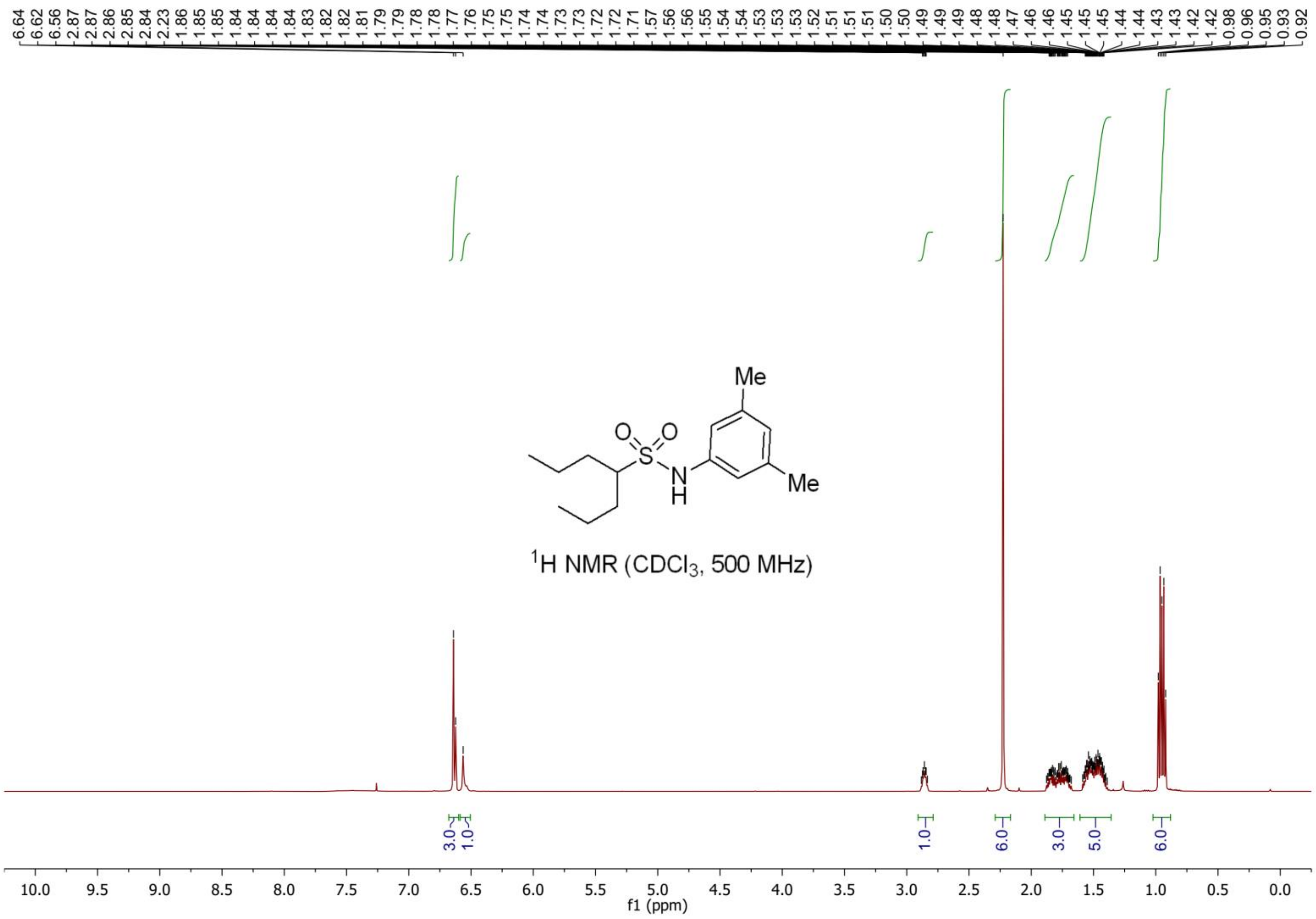

Go back to table of contents

Evaluation Only. Created with Aspose.Pdf. Copyright 2002-2014 Aspose Pty Ltd.

N-(3,5-dimethylphenyl)heptane-4-sulfonamide (8q)

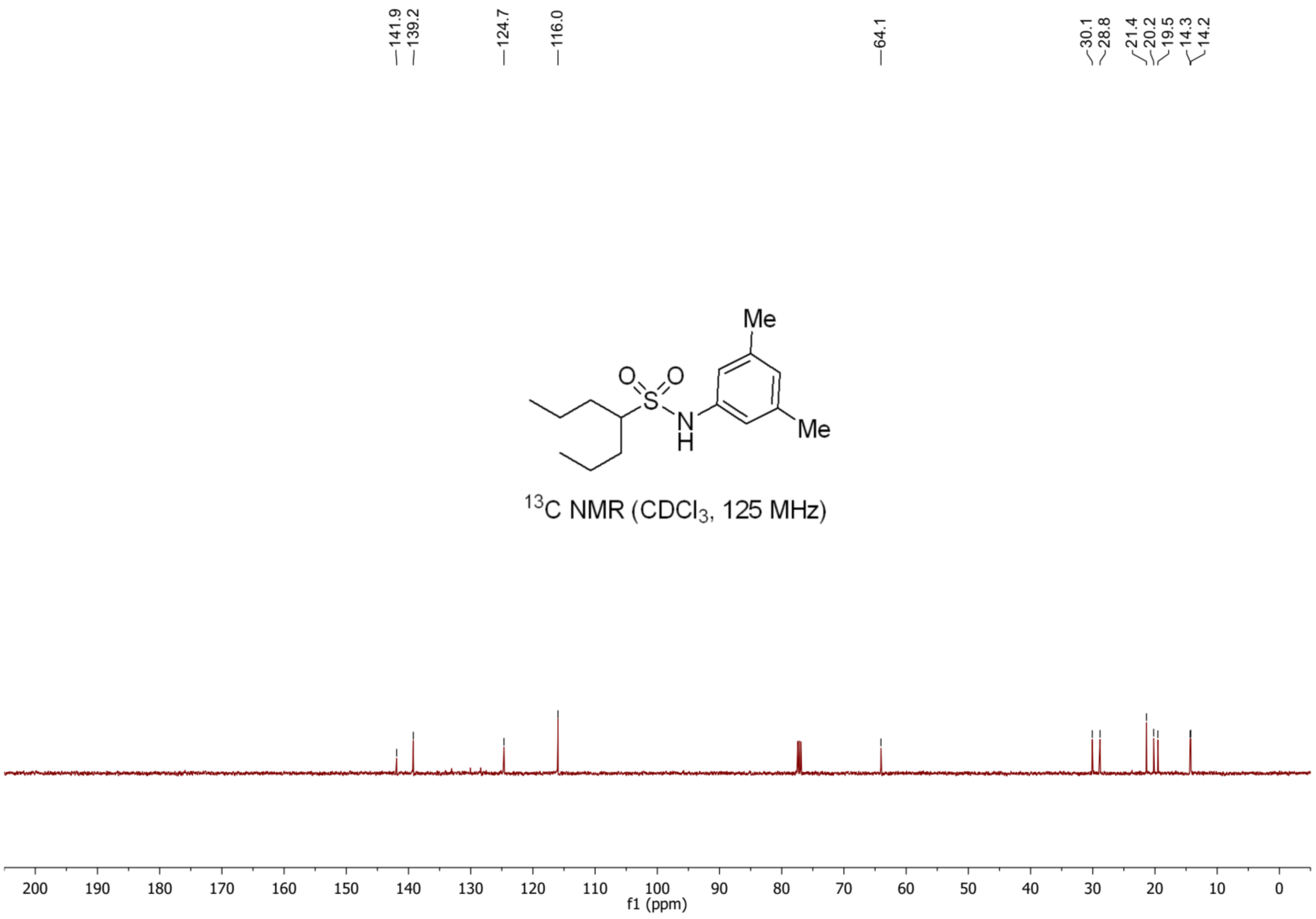

Go back to table of contents

Evaluation Only. Created with Aspose.Pdf. Copyright 2002-2014 Aspose Pty Ltd.

N-(2,3-dihydro-1*H*-inden-5-yl)-4,4-difluorocyclohexane-1-sulfonamide (8r)

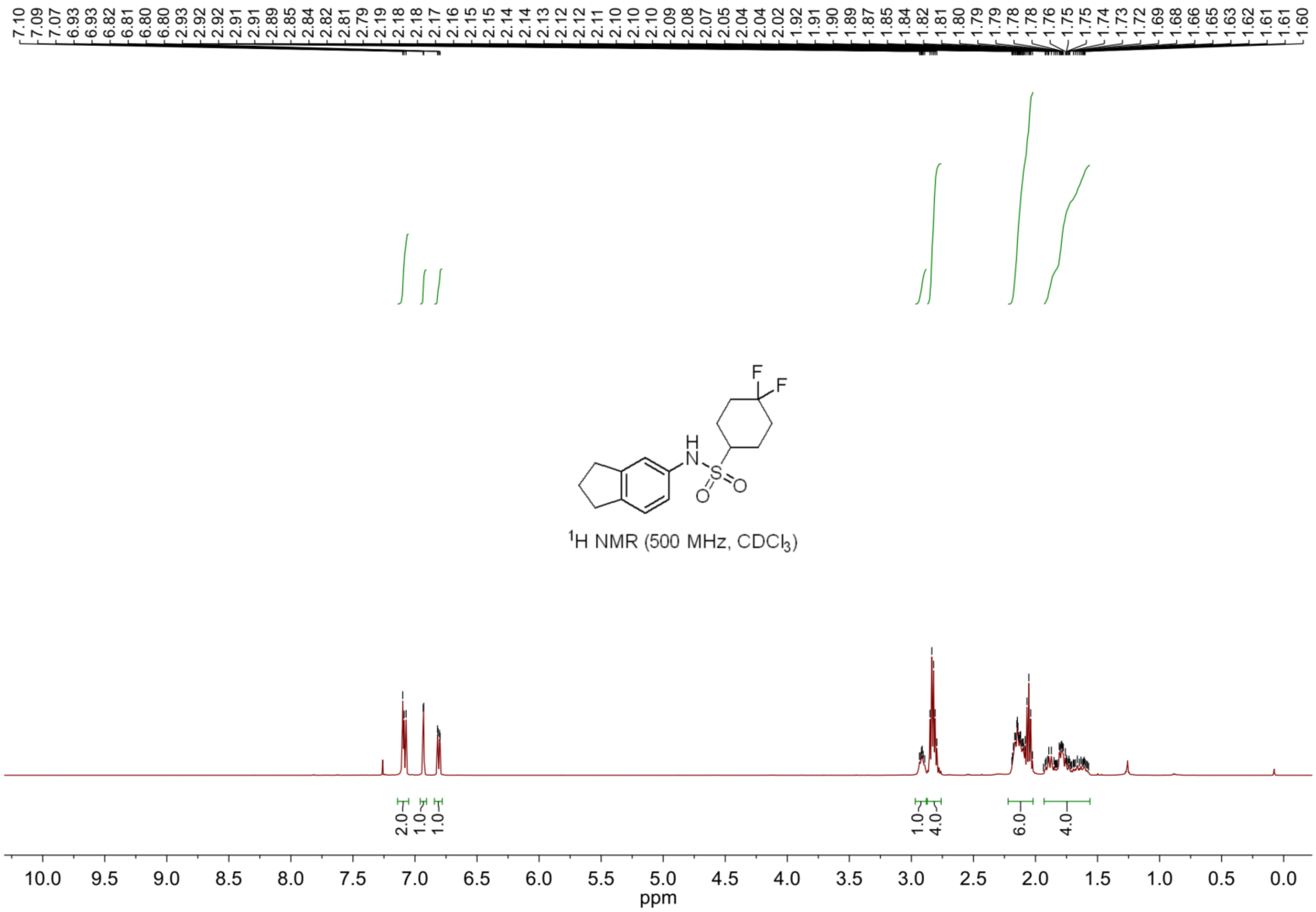

Go back to table of contents

Evaluation Only. Created with Aspose.Pdf. Copyright 2002-2014 Aspose Pty Ltd.

N-(2,3-dihydro-1*H*-inden-5-yl)-4,4-difluorocyclohexane-1-sulfonamide (8r)

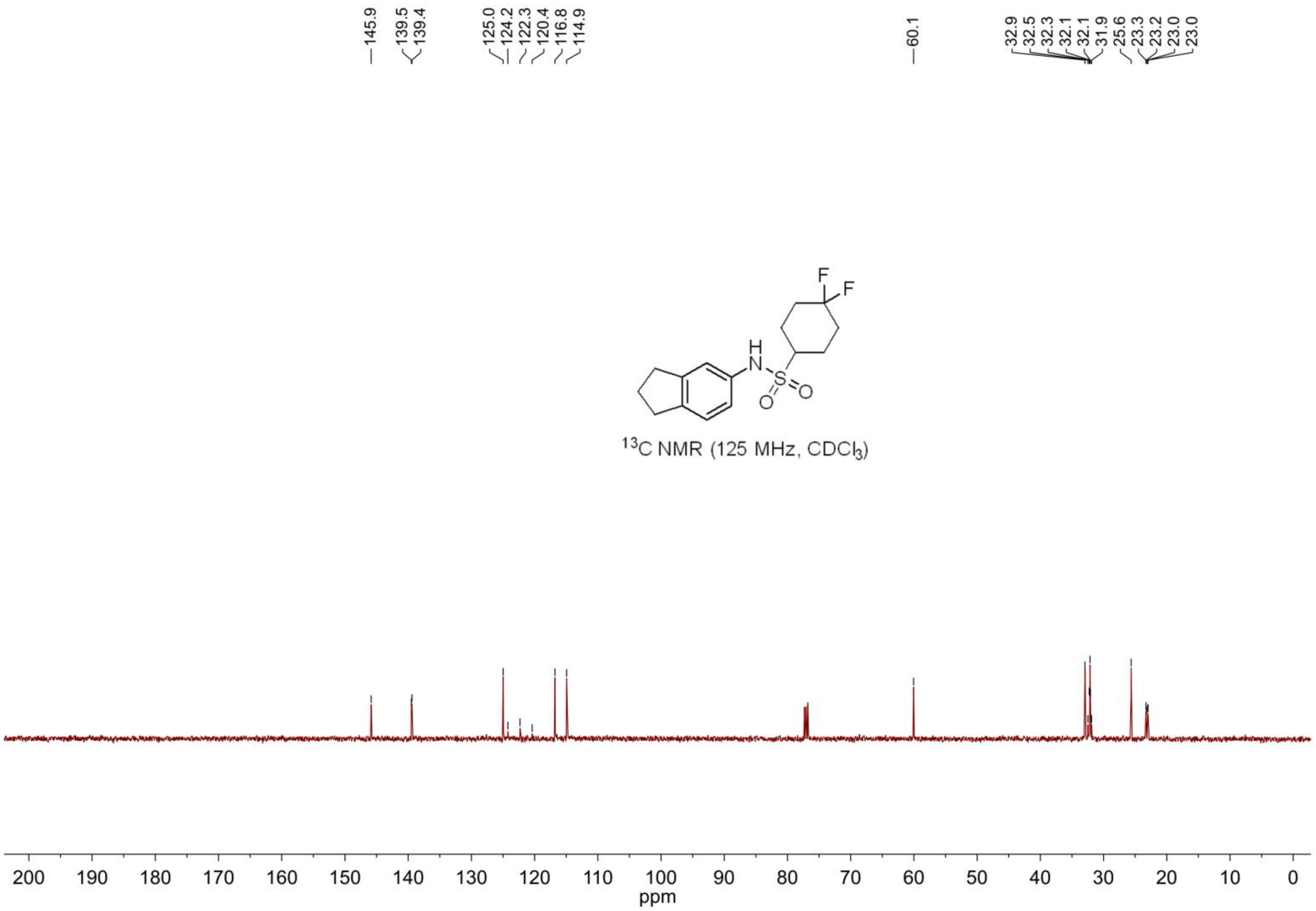

Go back to table of contents

Evaluation Only. Created with Aspose.Pdf. Copyright 2002-2014 Aspose Pty Ltd.

N-(2-chloro-4-methoxyphenyl)tetrahydro-2*H*-pyran-4-sulfonamide (8s)

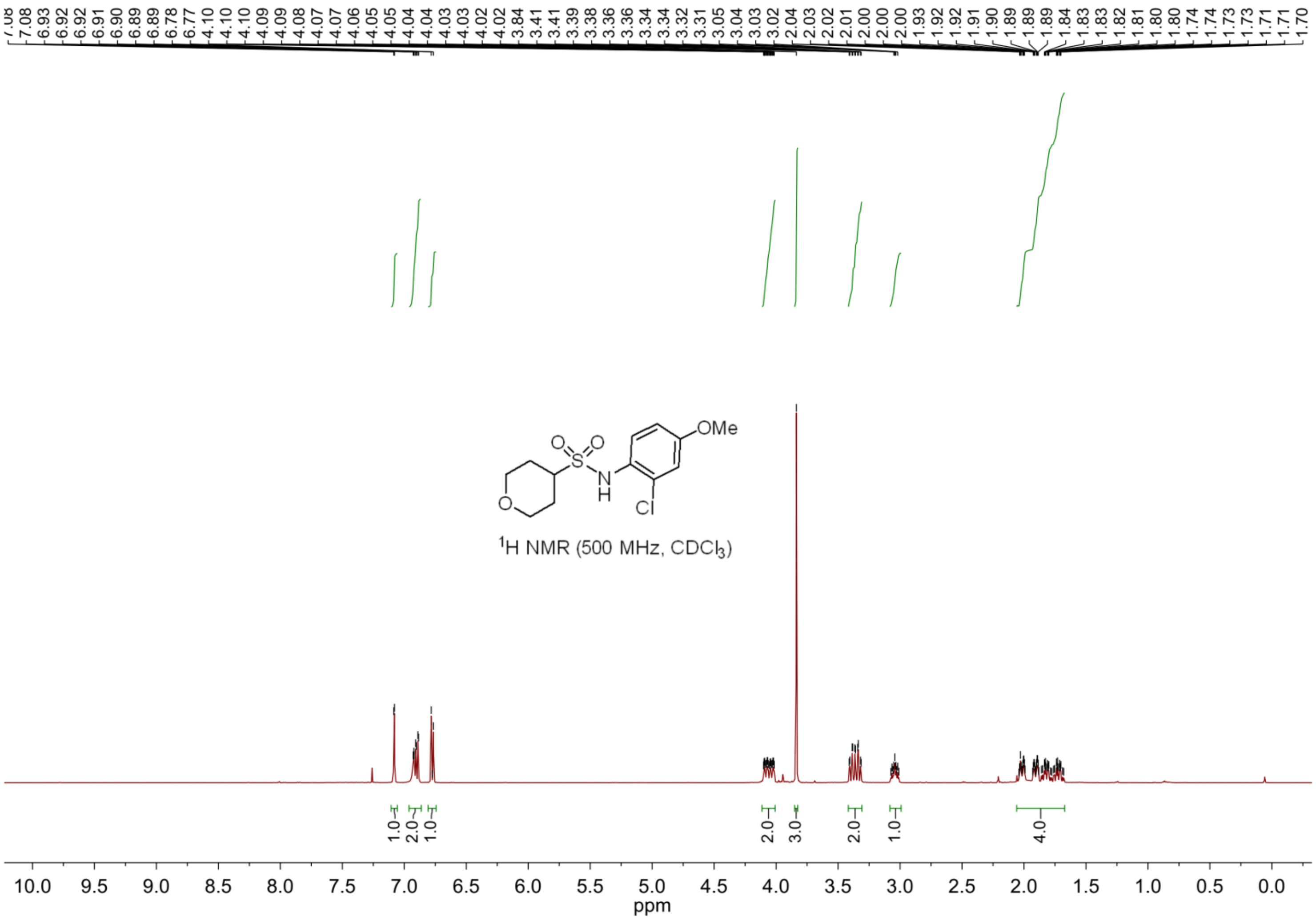

Go back to table of contents

Evaluation Only. Created with Aspose.Pdf. Copyright 2002-2014 Aspose Pty Ltd.

N-(2-chloro-4-methoxyphenyl)tetrahydro-2*H*-pyran-4-sulfonamide (8s)

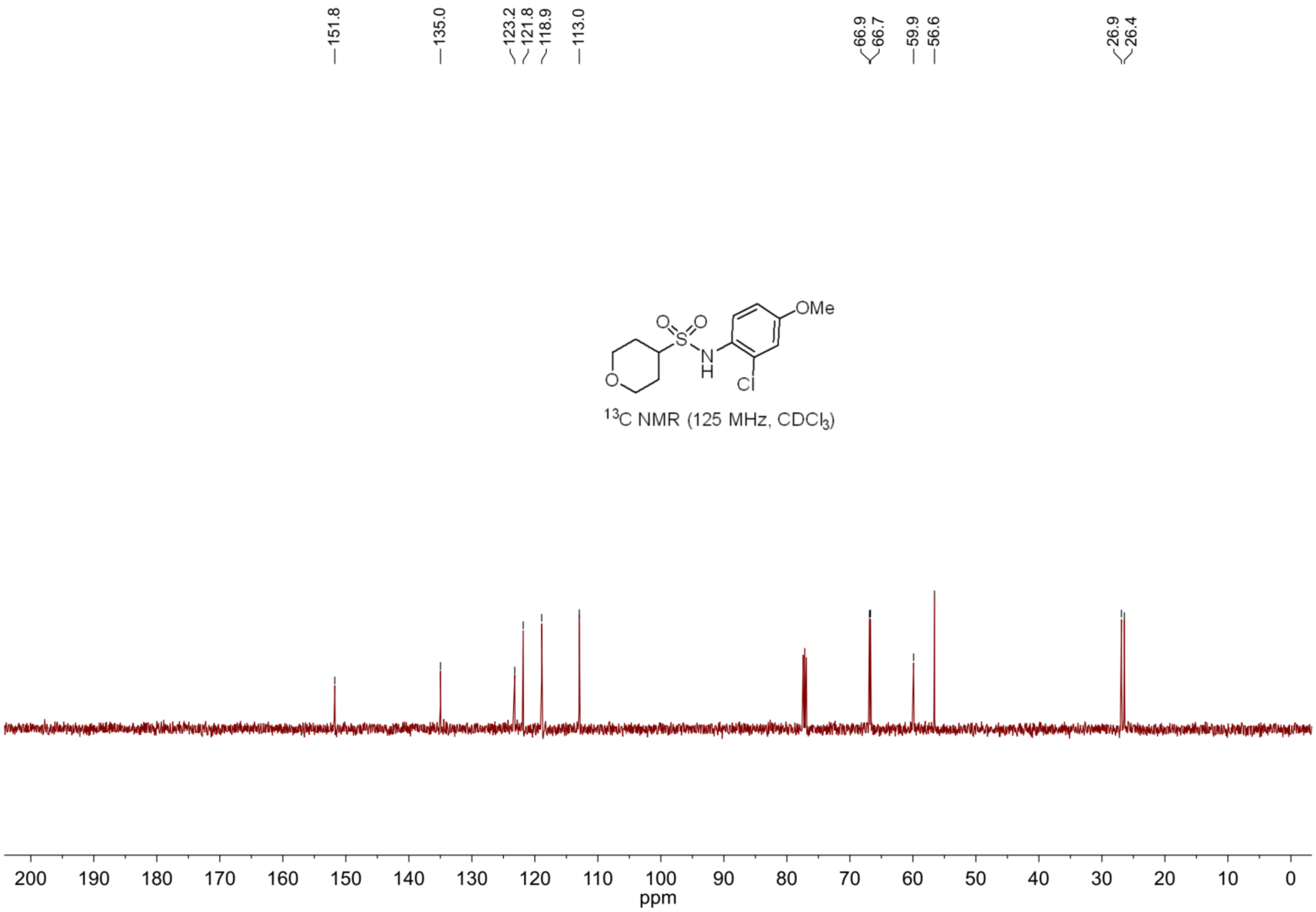

Go back to table of contents

Evaluation Only. Created with Aspose.Pdf. Copyright 2002-2014 Aspose Pty Ltd.

N-(4-Chlorophenyl)-*N*-methylbutane-1-sulfonamide (8t)

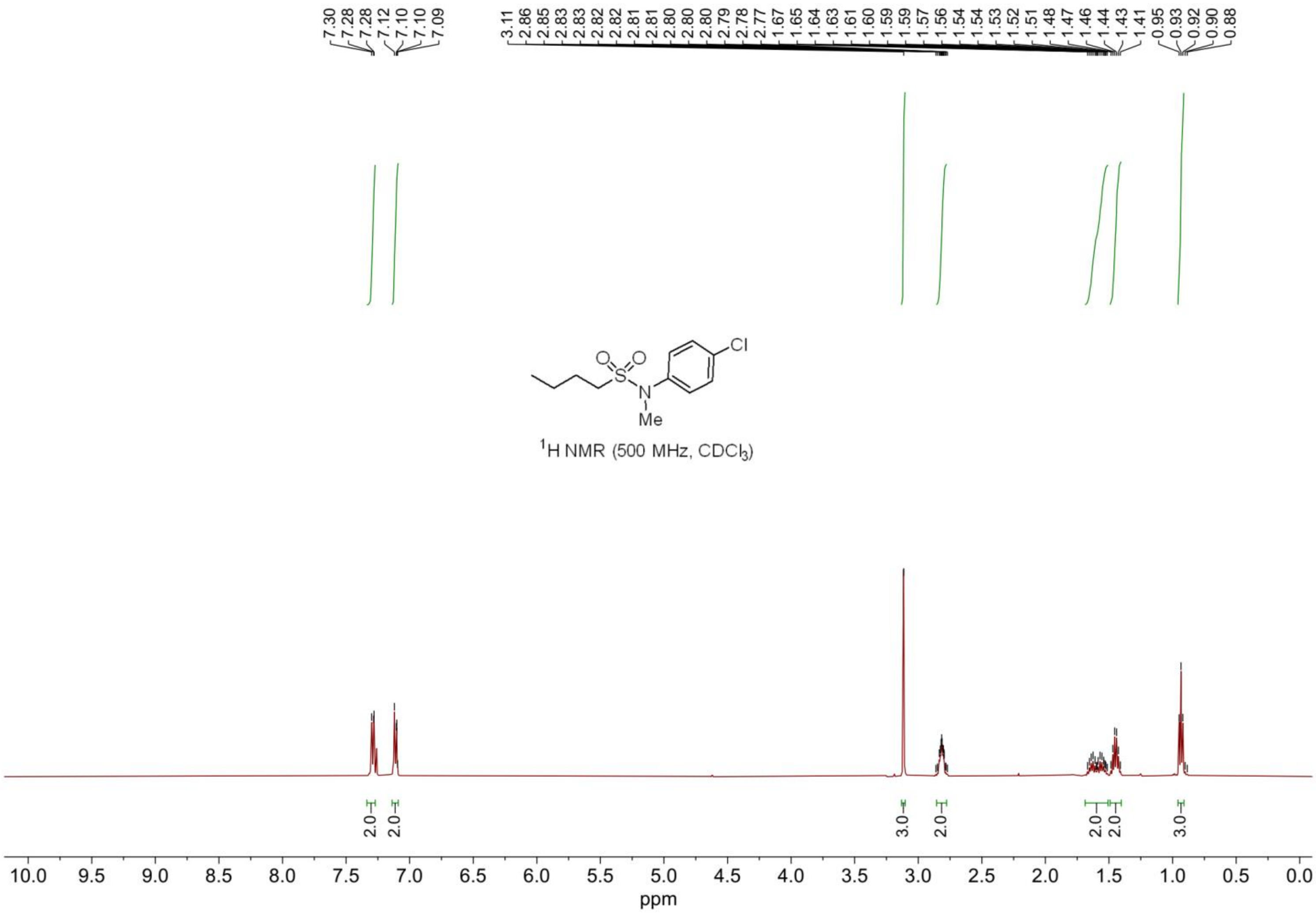

Evaluation Only. Created with Aspose.Pdf. Copyright 2002-2014 Aspose Pty Ltd.

N-(4-Chlorophenyl)-*N*-methylbutane-1-sulfonamide (8t)

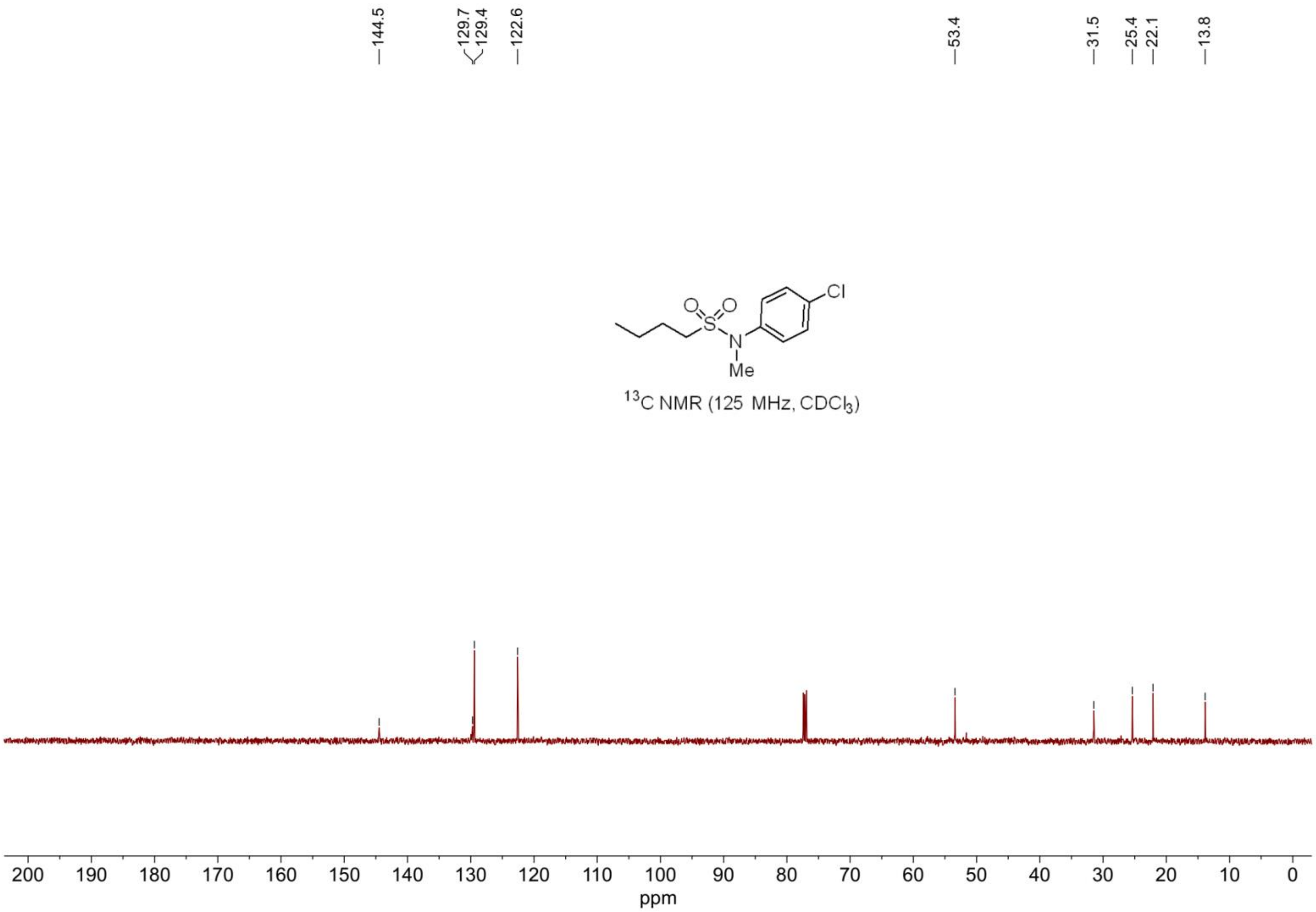

Evaluation Only. Created with Aspose.Pdf. Copyright 2002-2014 Aspose Pty Ltd.

1-((4,4-Difluorocyclohexyl)sulfonyl)indoline (8u)

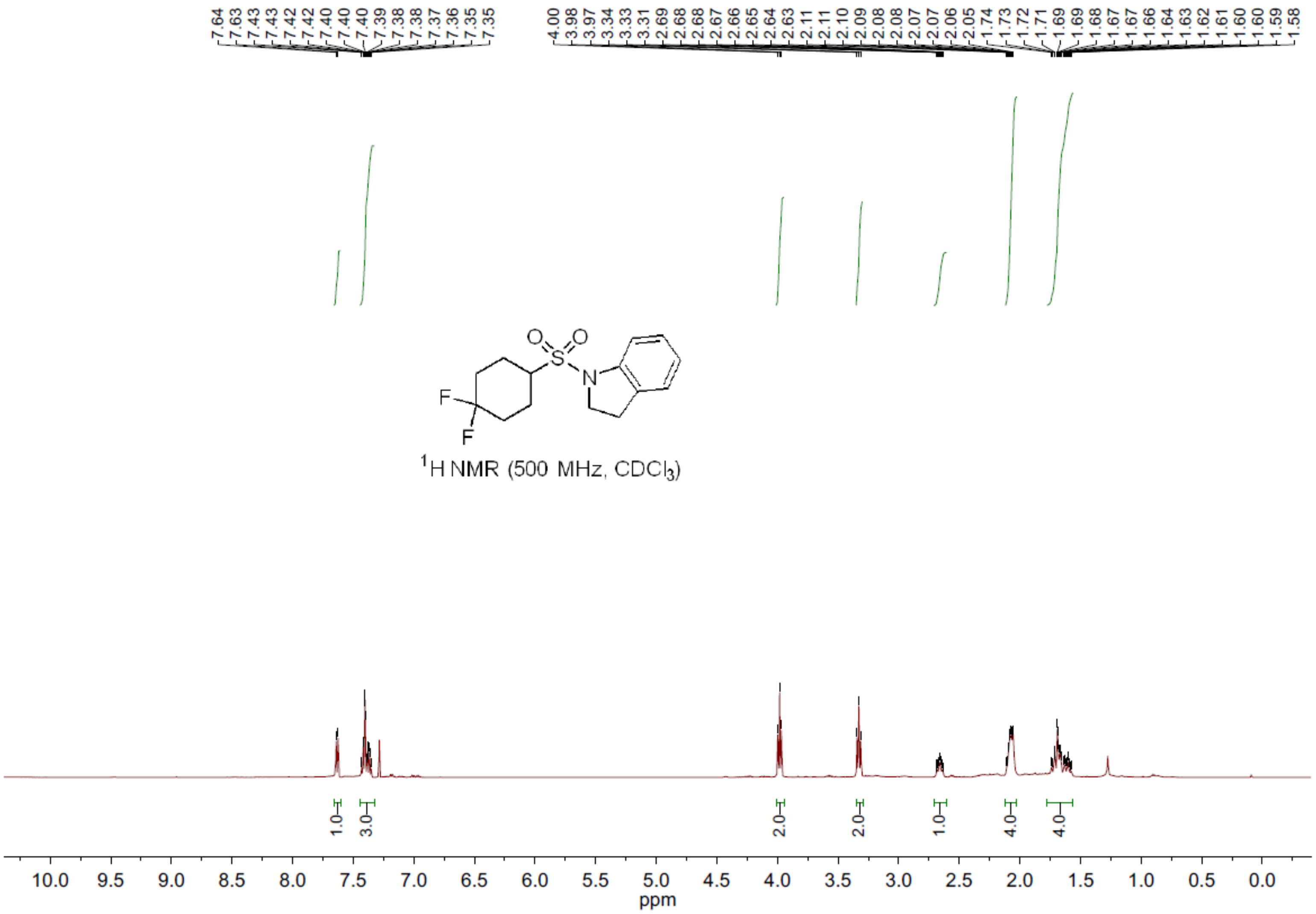

Go back to table of contents

Evaluation Only. Created with Aspose.Pdf. Copyright 2002-2014 Aspose Pty Ltd.

1-((4,4-Difluorocyclohexyl)sulfonyl)indoline (8u)

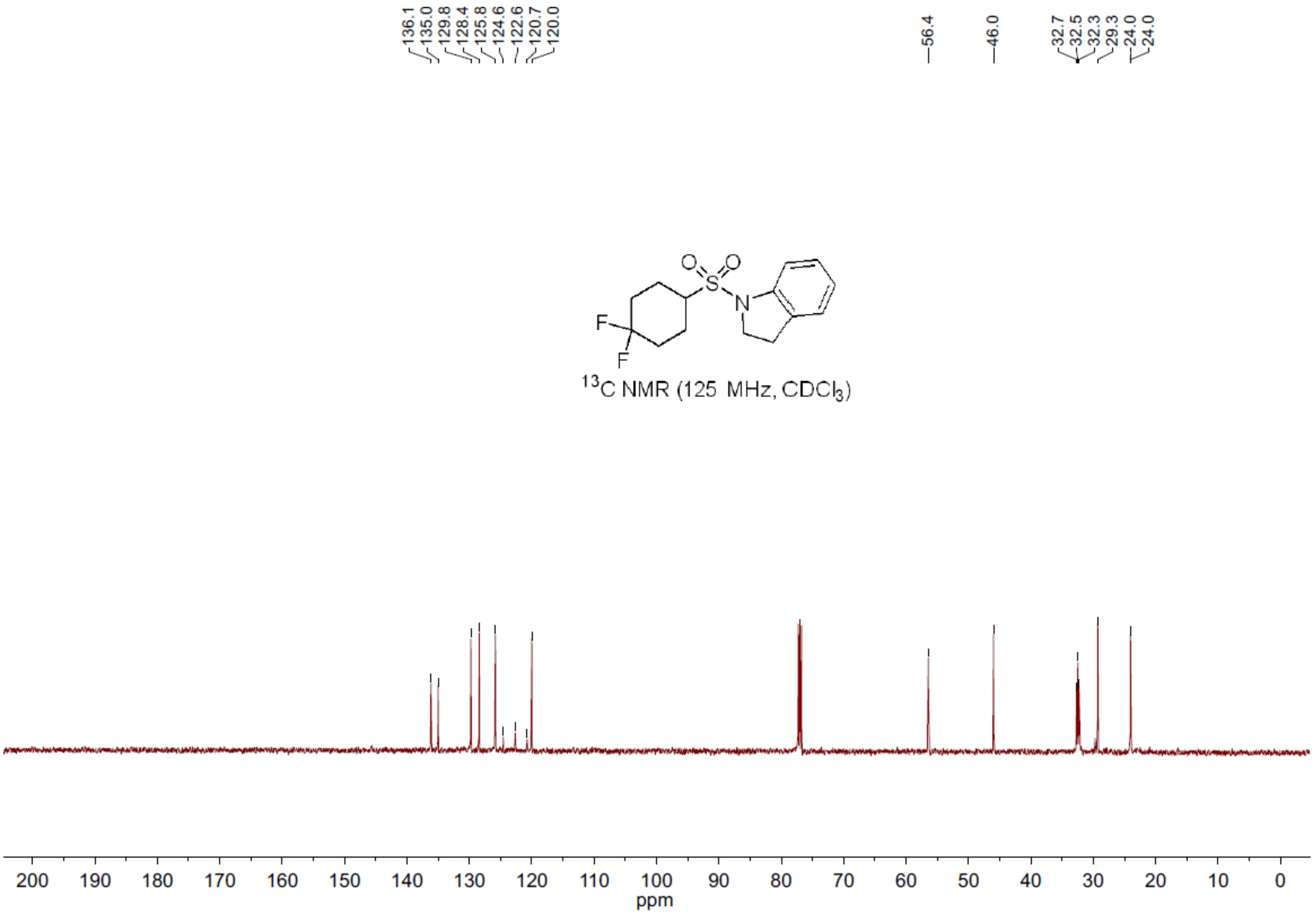

Go back to table of contents

Evaluation Only. Created with Aspose.Pdf. Copyright 2002-2014 Aspose Pty Ltd.

tert-Butyl 4-(*N*-(3,4,5-trimethylphenyl)sulfamoyl)piperidine-1-carboxylate (8v)

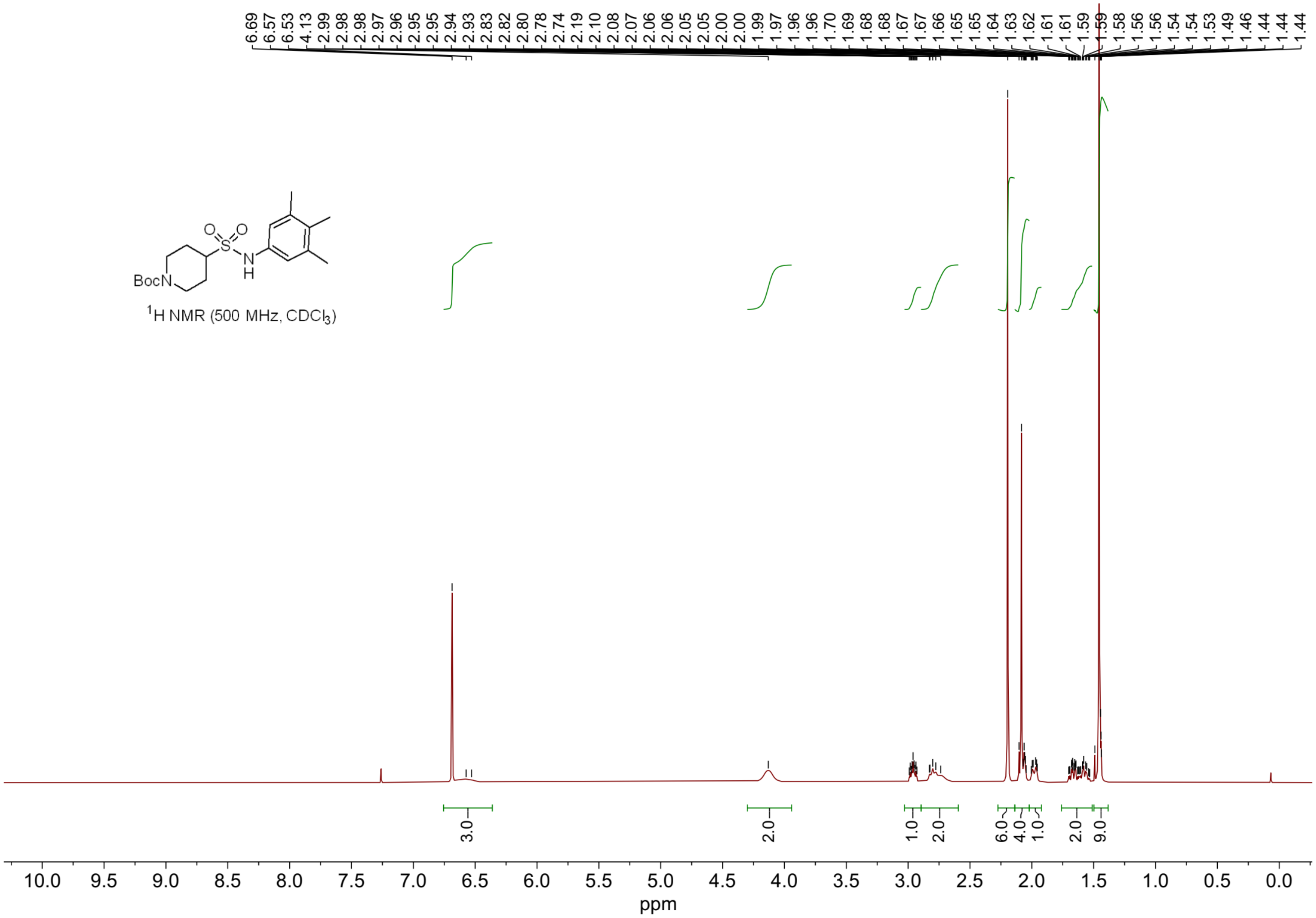

Go back to table of contents

Evaluation Only. Created with Aspose.Pdf. Copyright 2002-2014 Aspose Pty Ltd.

tert-Butyl 4-(*N*-(3,4,5-trimethylphenyl)sulfamoyl)piperidine-1-carboxylate (8v)

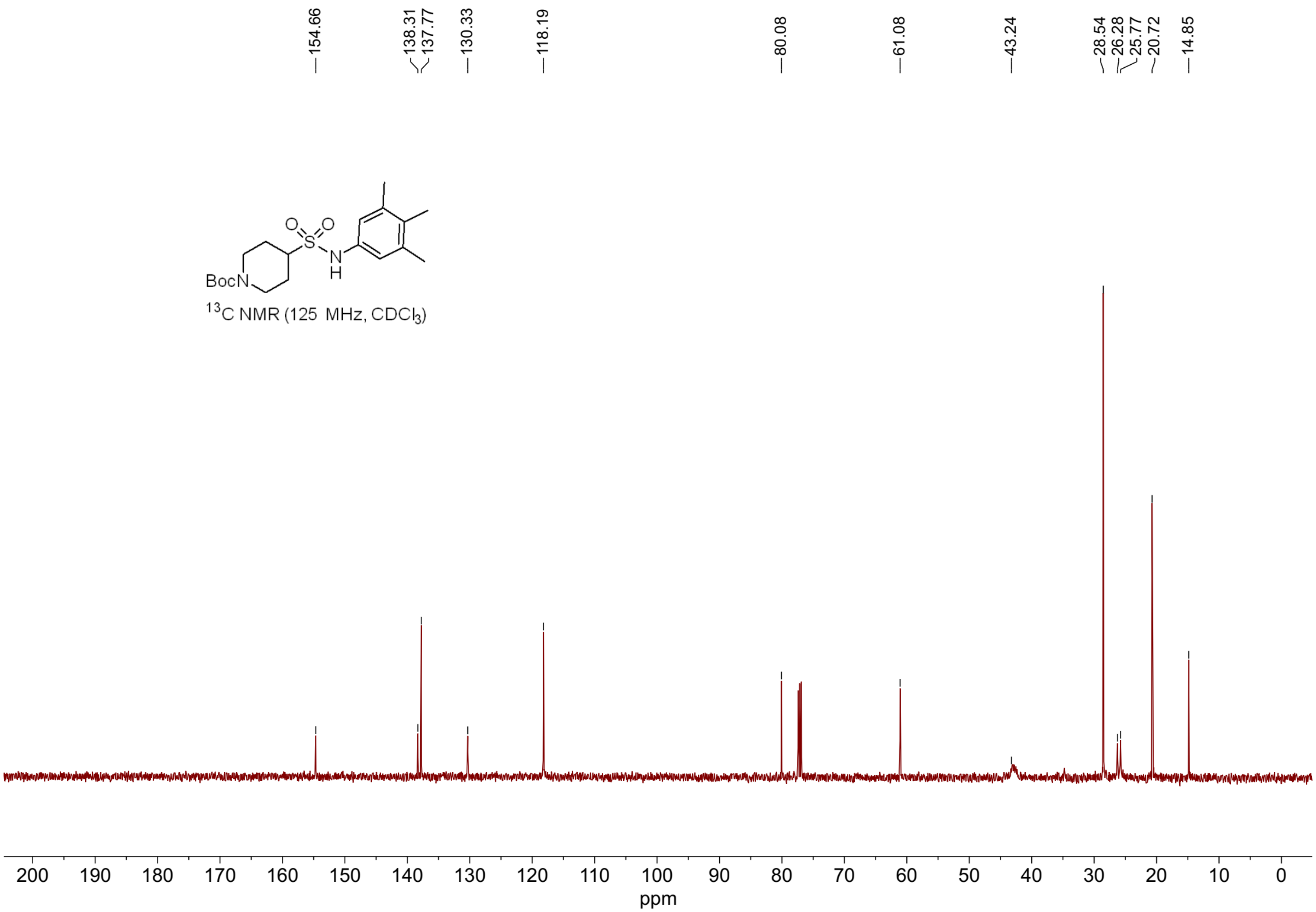

Go back to table of contents

Evaluation Only. Created with Aspose.Pdf. Copyright 2002-2014 Aspose Pty Ltd.

N-(2-methylbenzo[d]thiazol-6-yl)butane-1-sulfonamide (8w)

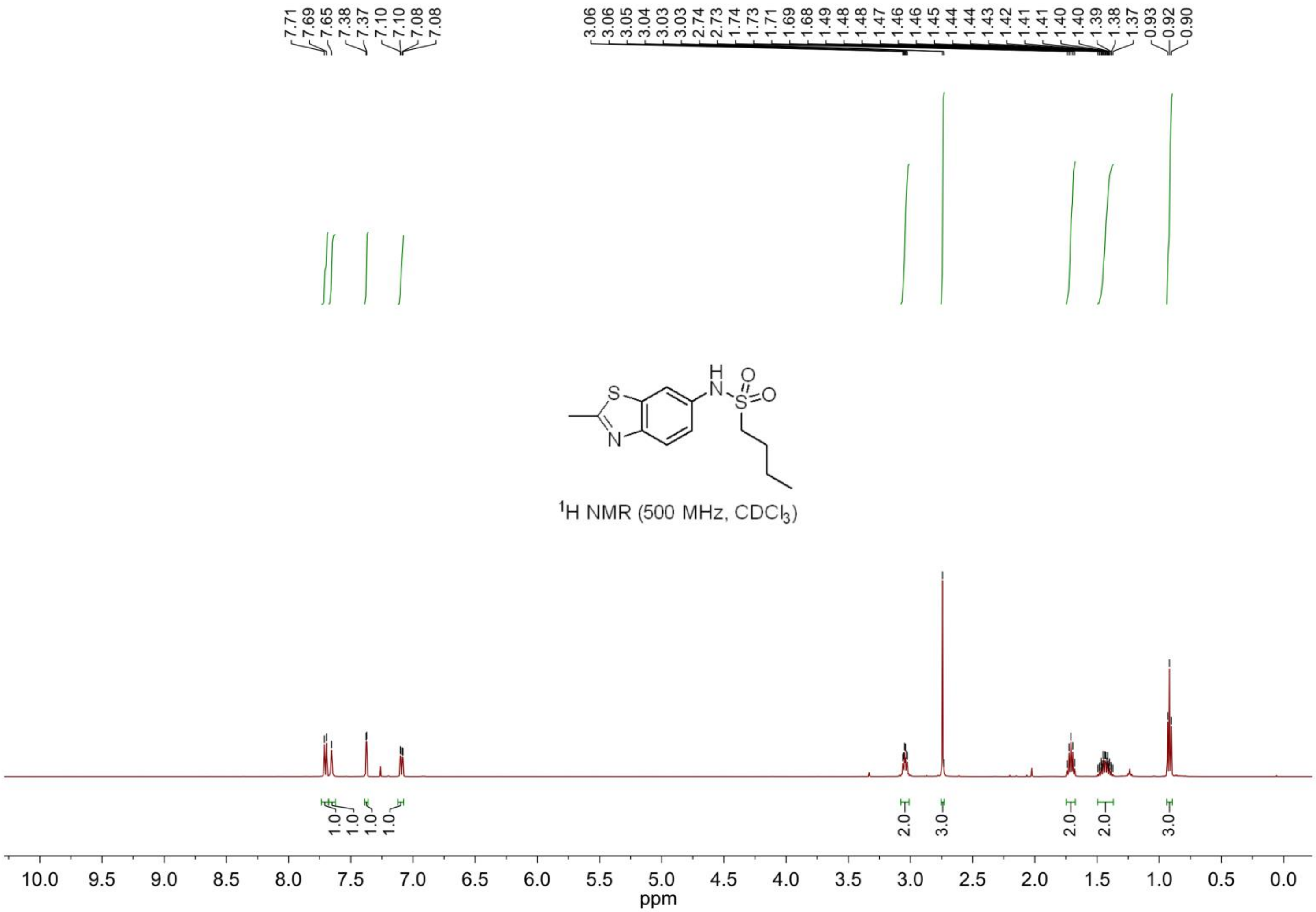

Evaluation Only. Created with Aspose.Pdf. Copyright 2002-2014 Aspose Pty Ltd.

N-(2-methylbenzo[d]thiazol-6-yl)butane-1-sulfonamide (8w)

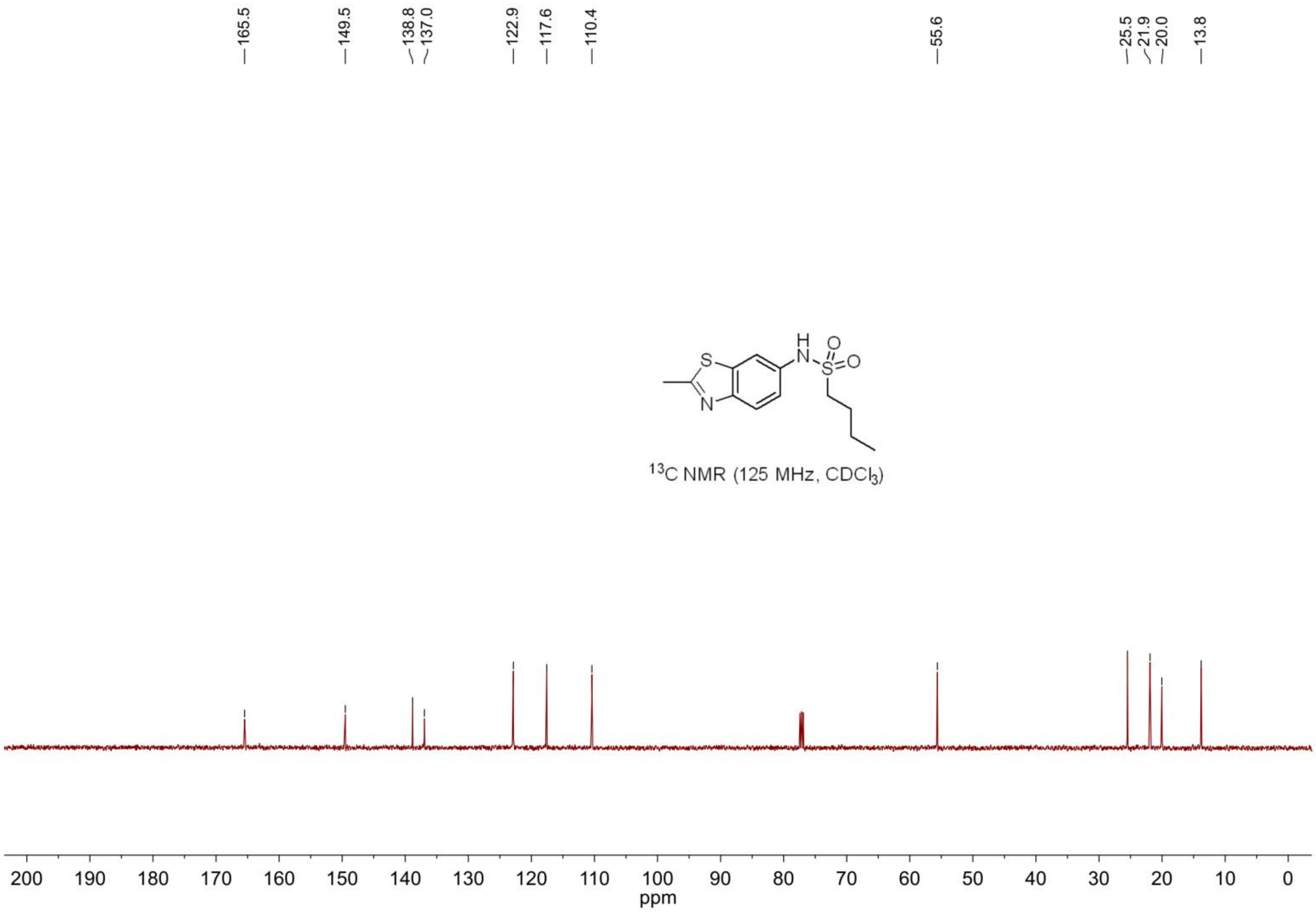

Go back to table of contents

Evaluation Only. Created with Aspose.Pdf. Copyright 2002-2014 Aspose Pty Ltd.

1-((3,3,3-Trifluoropropyl)sulfonyl)indoline (8x)

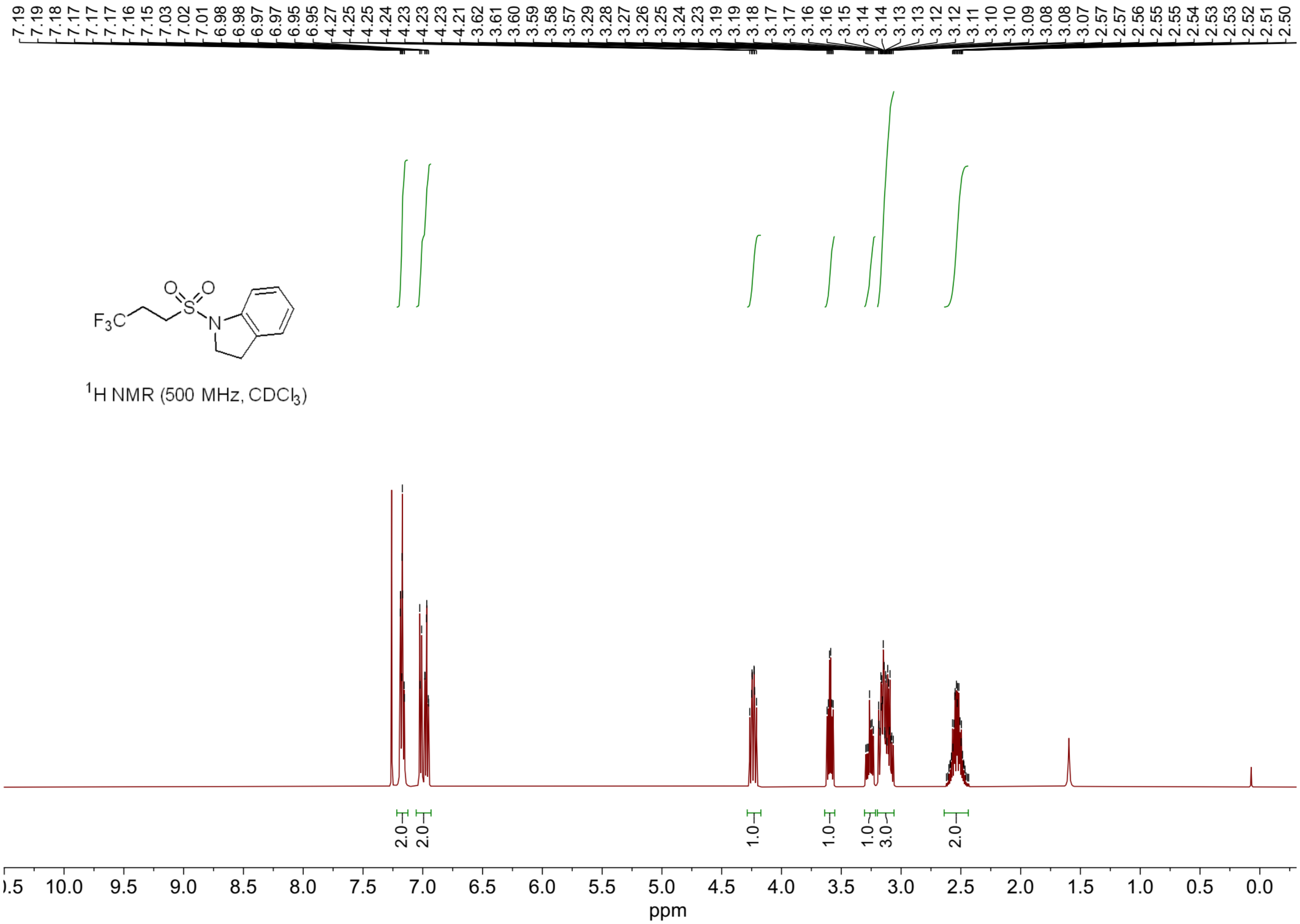

Go back to table of contents

Evaluation Only. Created with Aspose.Pdf. Copyright 2002-2014 Aspose Pty Ltd.

1-((3,3,3-Trifluoropropyl)sulfonyl)indoline (8x)

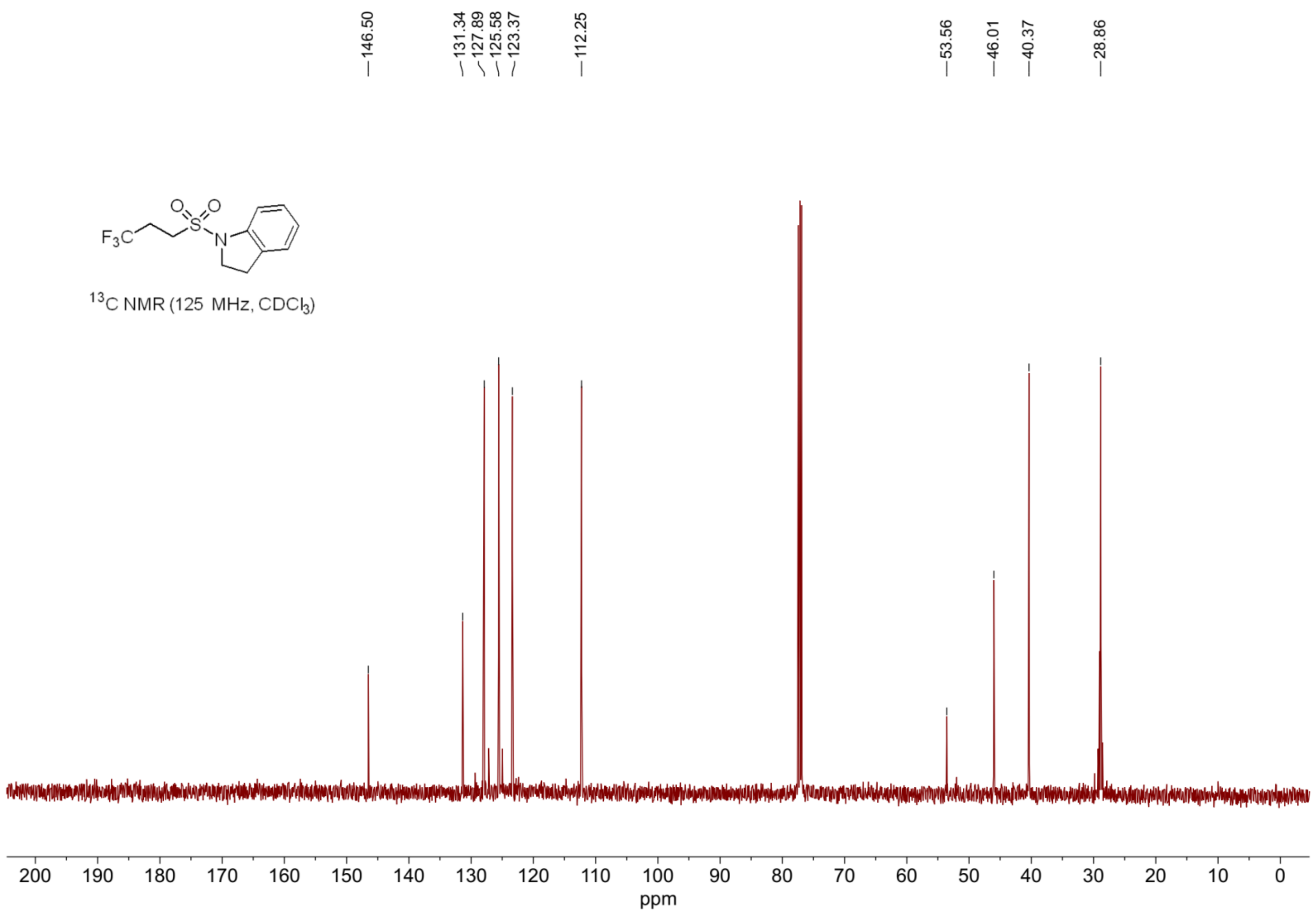

Go back to table of contents

Evaluation Only. Created with Aspose.Pdf. Copyright 2002-2014 Aspose Pty Ltd.

Evaluation Only. Created with Aspose.Pdf. Copyright 2002-2014 Aspose Pty Ltd.

2-(3,4-Dimethoxyphenyl)ethane-1-sulfonyl azide (9a)

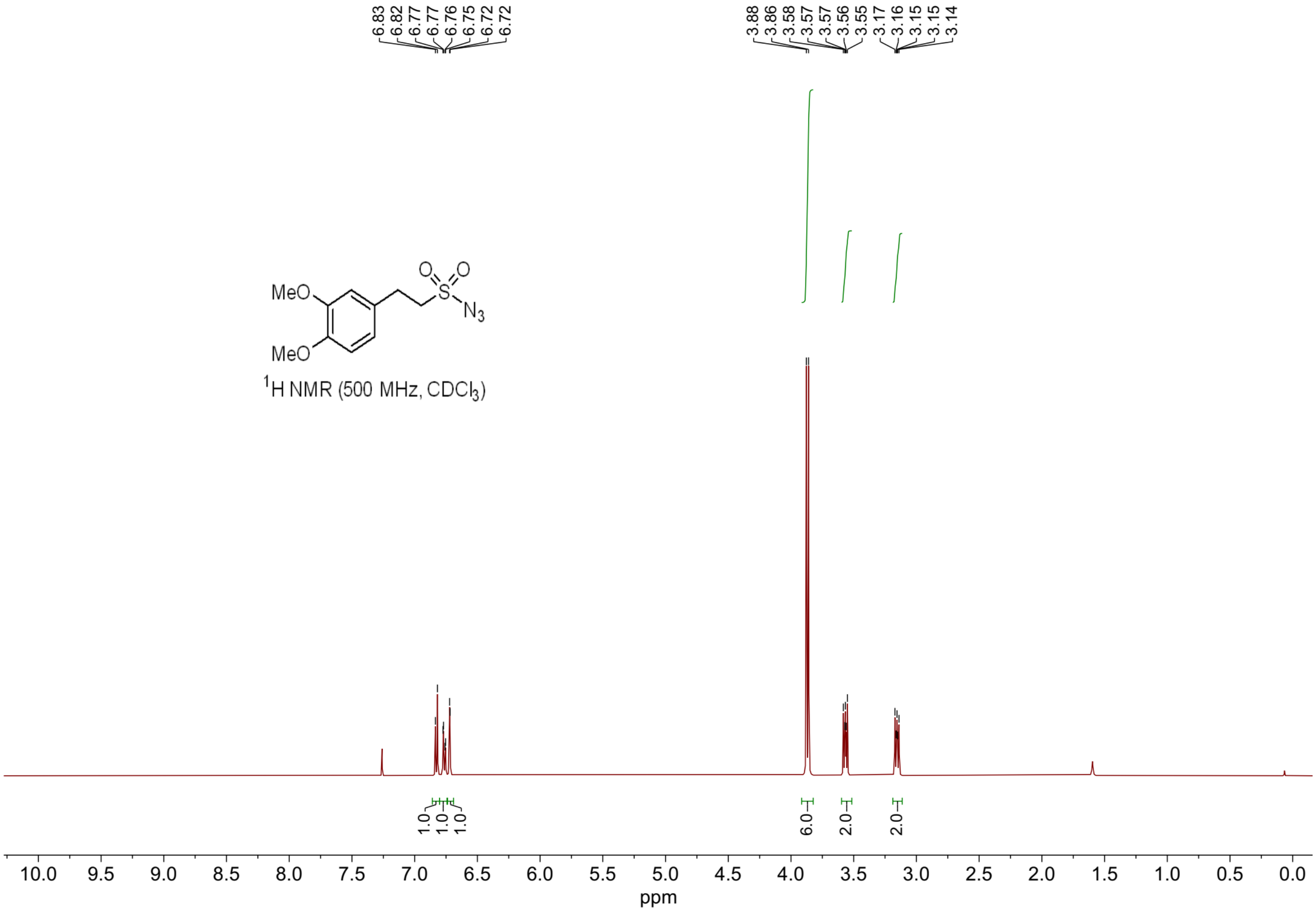

Go back to table of contents

Evaluation Only. Created with Aspose.Pdf. Copyright 2002-2014 Aspose Pty Ltd.

2-(3,4-Dimethoxyphenyl)ethane-1-sulfonyl azide (9a)

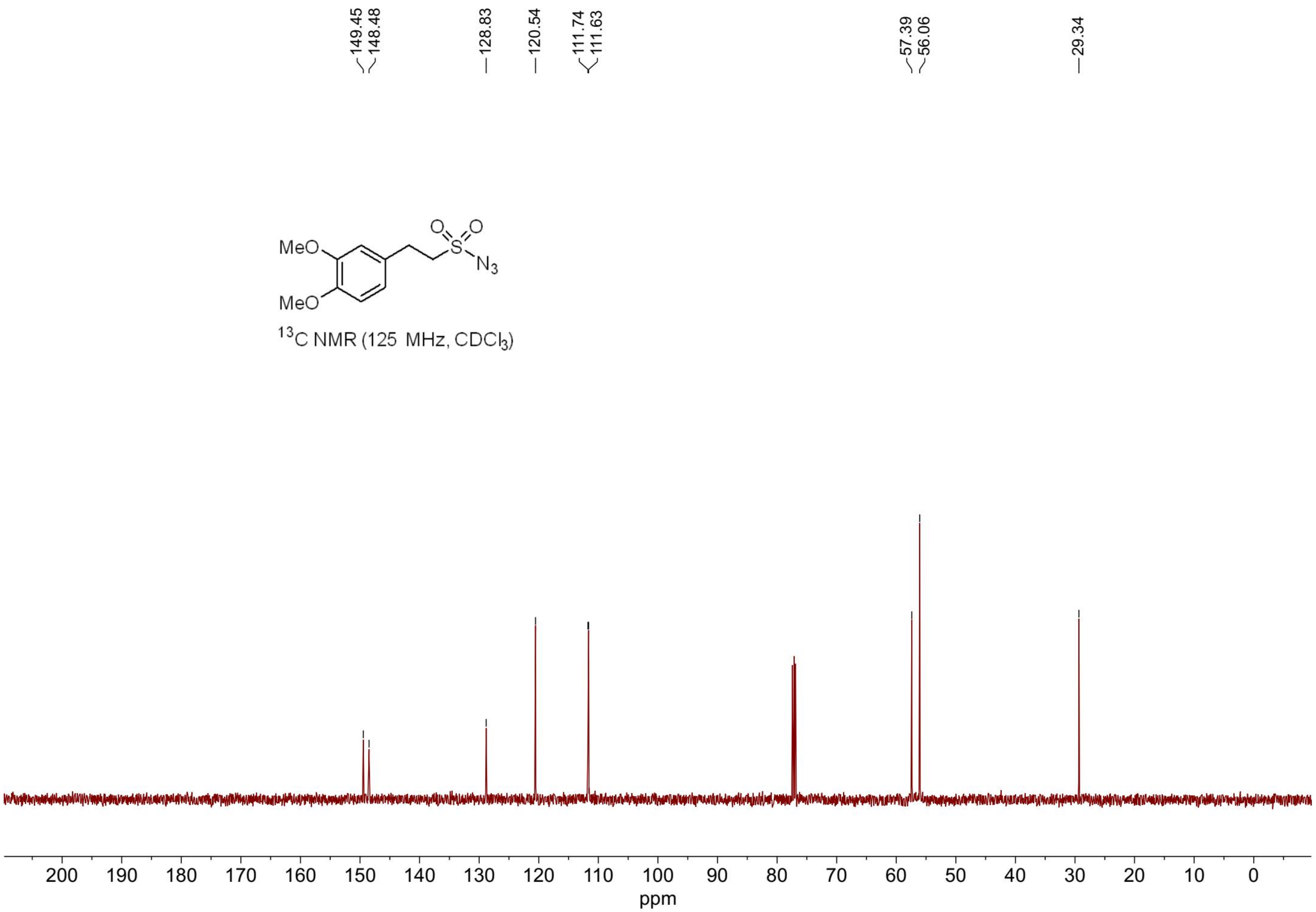

Go back to table of contents

Evaluation Only. Created with Aspose.Pdf. Copyright 2002-2014 Aspose Pty Ltd.

4-Oxo-4-phenylbutane-1-sulfonyl azide (9b)

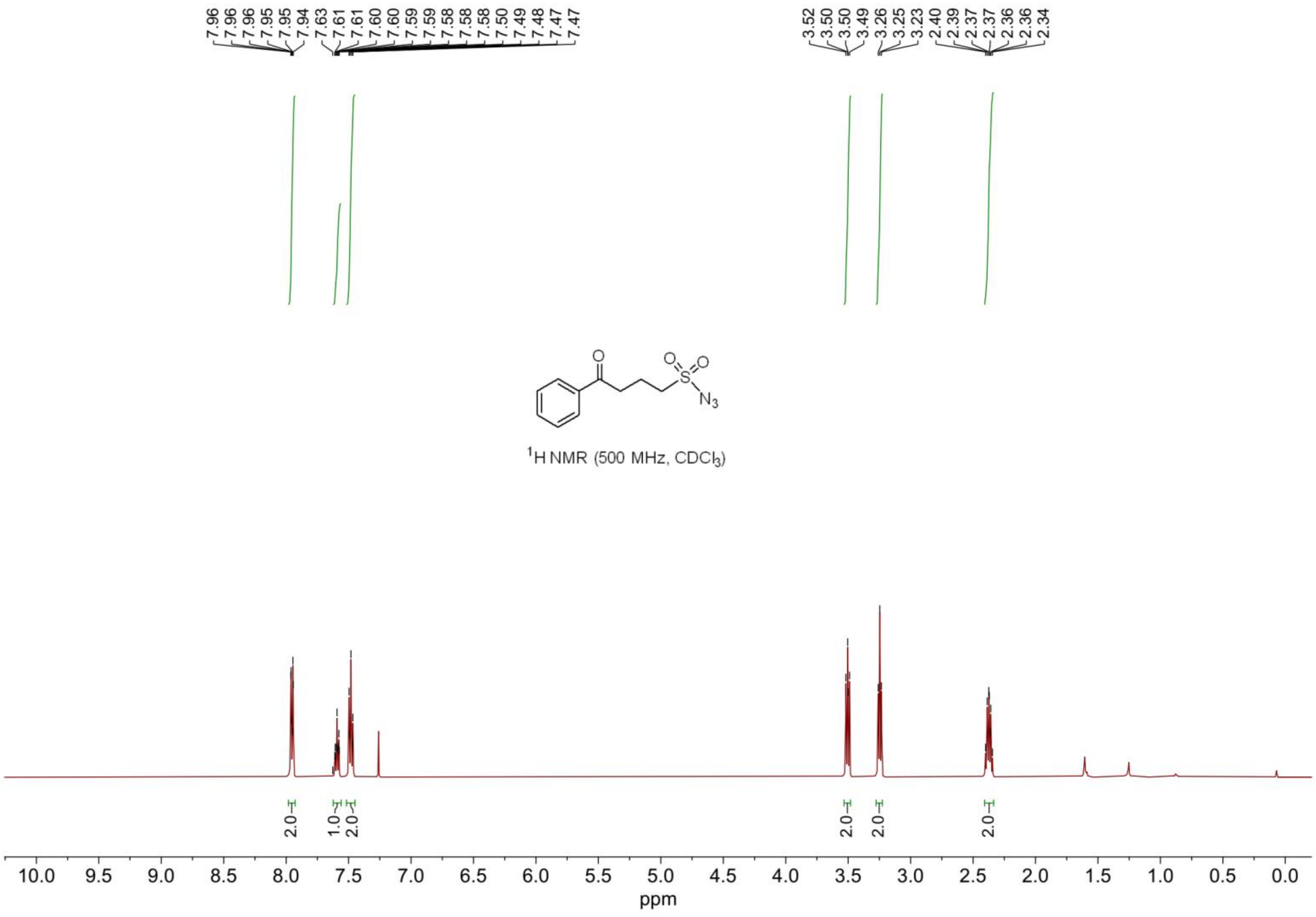

Go back to table of contents

Evaluation Only. Created with Aspose.Pdf. Copyright 2002-2014 Aspose Pty Ltd.

4-Oxo-4-phenylbutane-1-sulfonyl azide (9b)

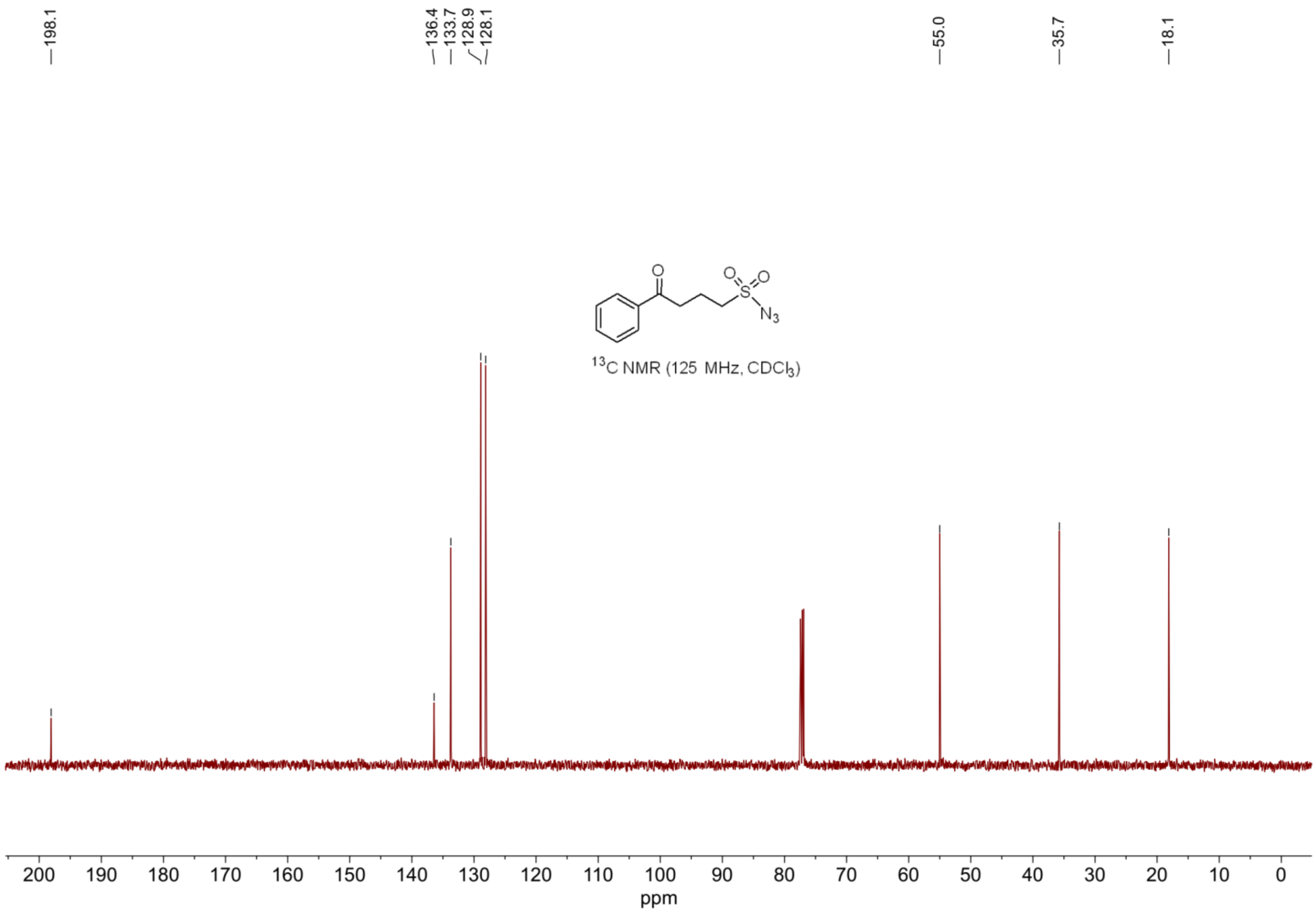

Go back to table of contents

Evaluation Only. Created with Aspose.Pdf. Copyright 2002-2014 Aspose Pty Ltd.

Methyl 5-(azidosulfonyl)pentanoate (9c)

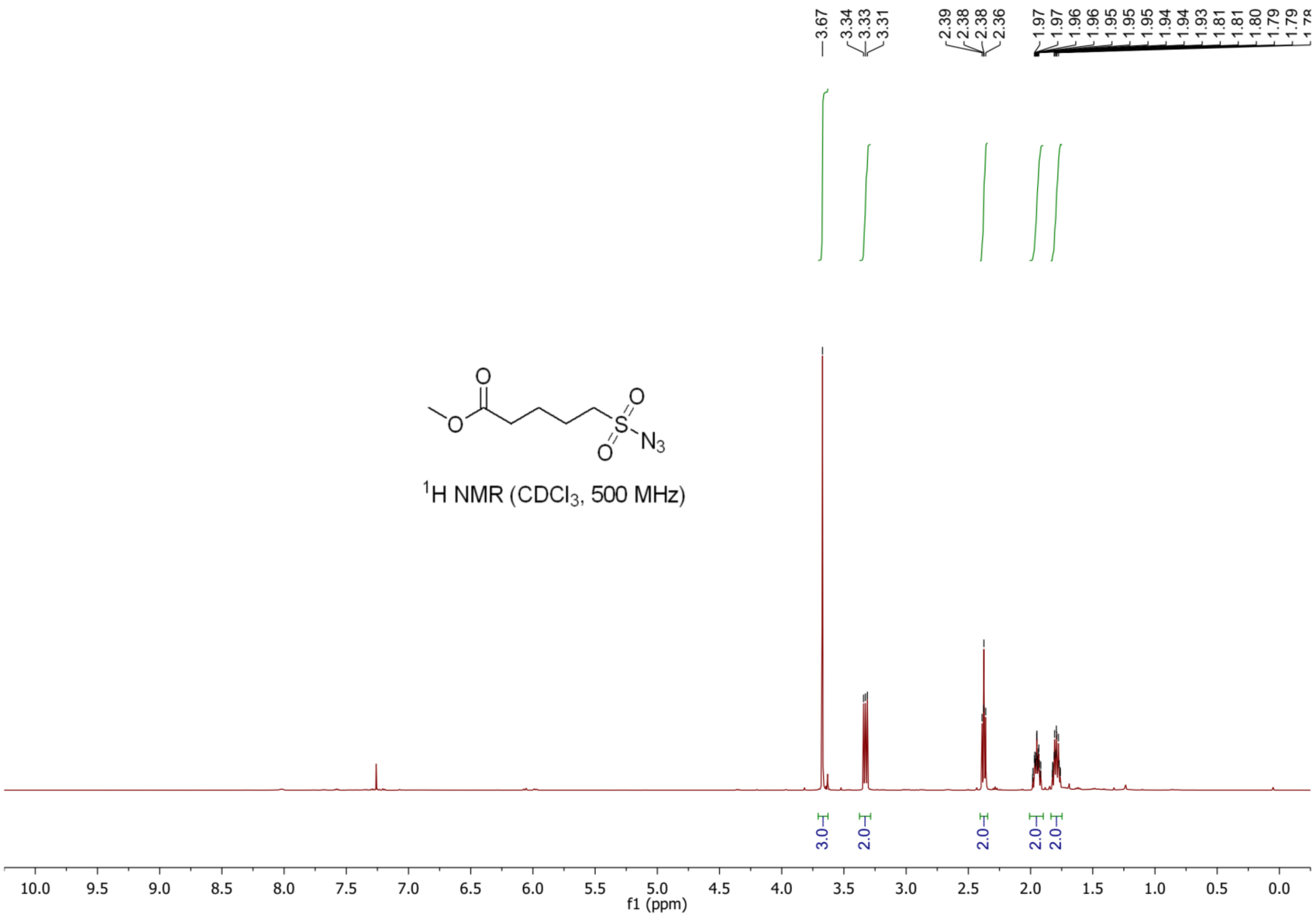

Go back to table of contents

Evaluation Only. Created with Aspose.Pdf. Copyright 2002-2014 Aspose Pty Ltd.

Methyl 5-(azidosulfonyl)pentanoate (9c)

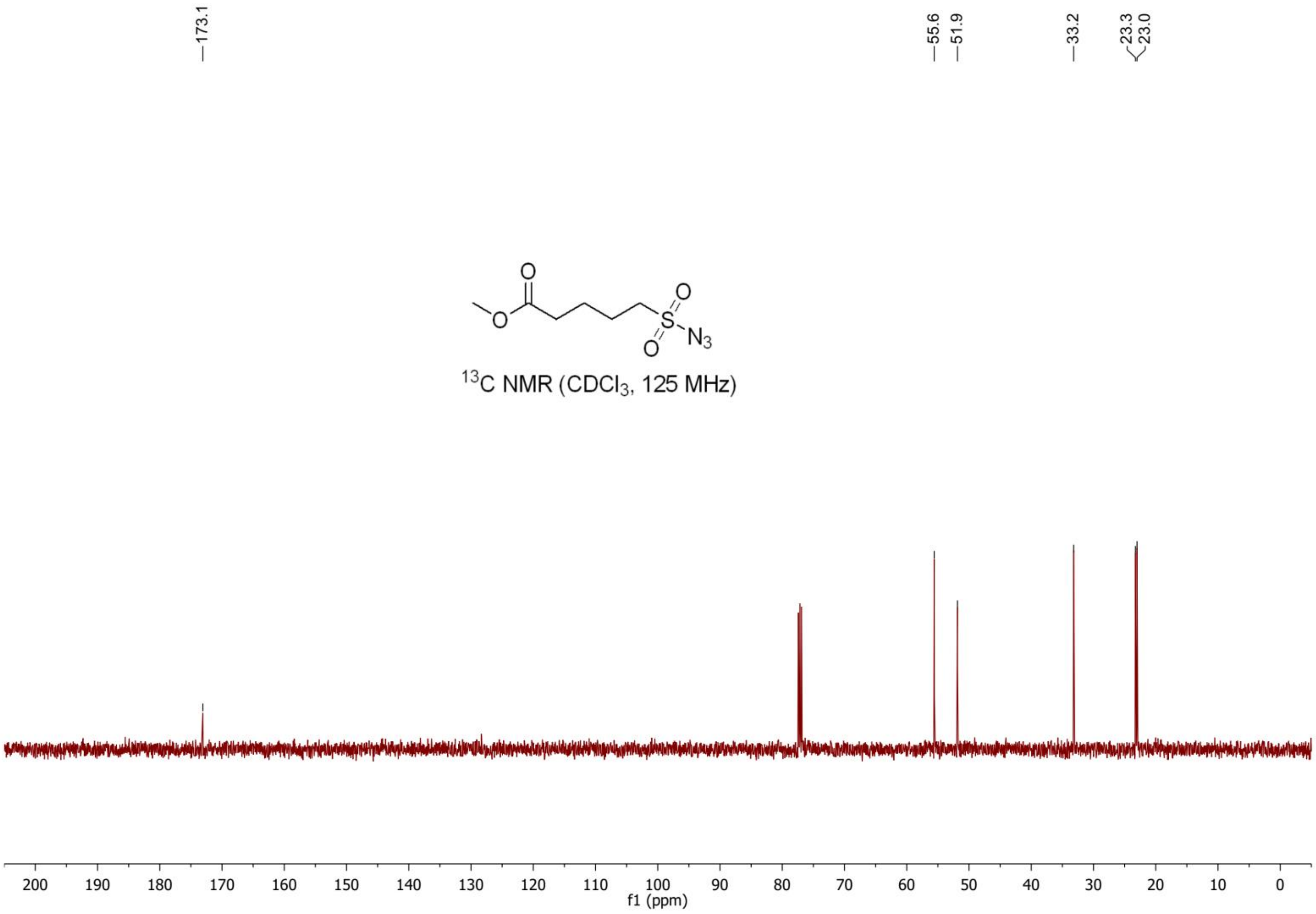

Go back to table of contents

Evaluation Only. Created with Aspose.Pdf. Copyright 2002-2014 Aspose Pty Ltd.

4-(5-Methylthiophen-2-yl)-4-oxobutane-1-sulfonyl azide (9d)

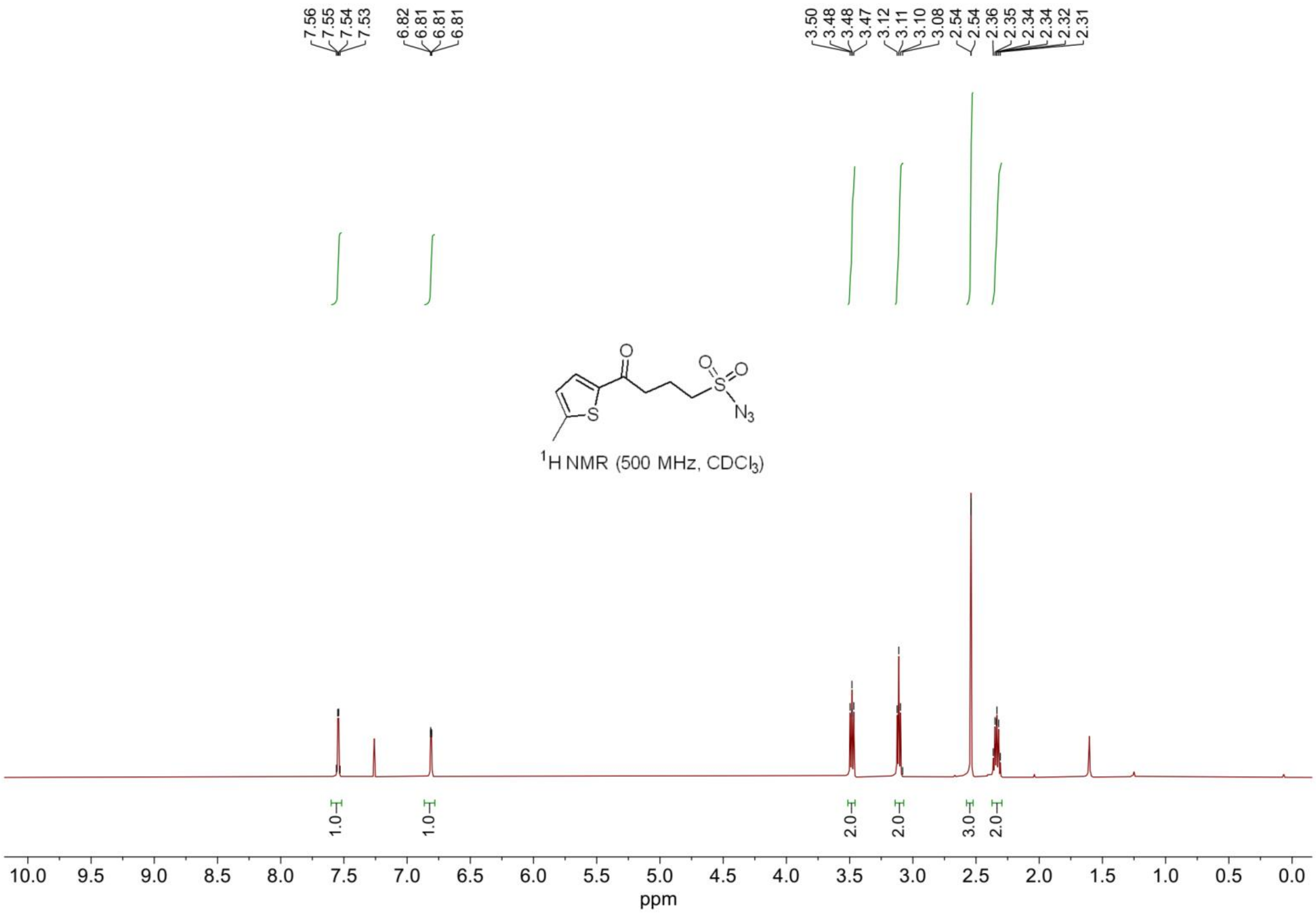

Go back to table of contents

Evaluation Only. Created with Aspose.Pdf. Copyright 2002-2014 Aspose Pty Ltd.

4-(5-Methylthiophen-2-yl)-4-oxobutane-1-sulfonyl azide (9d)

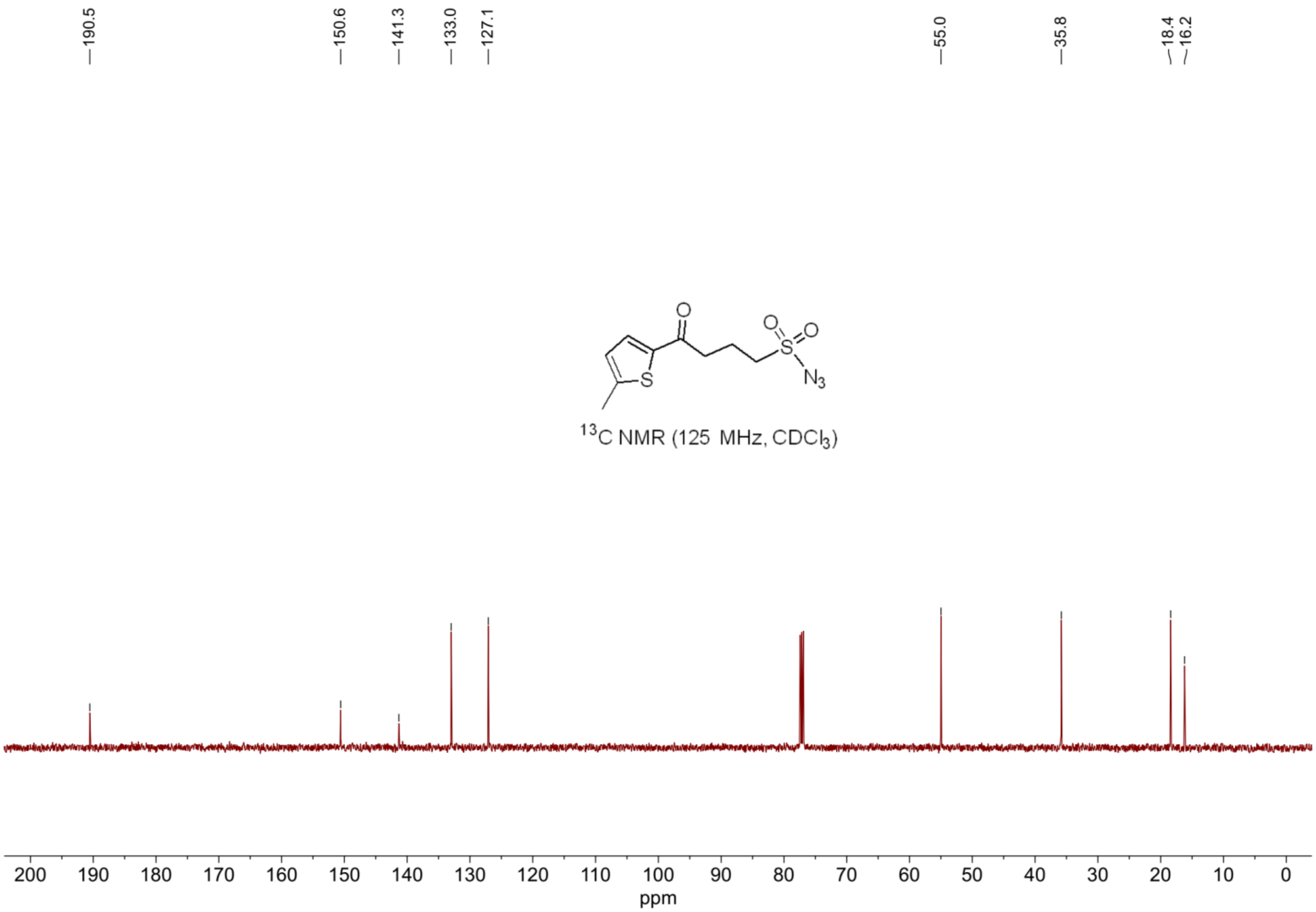

Go back to table of contents

Evaluation Only. Created with Aspose.Pdf. Copyright 2002-2014 Aspose Pty Ltd.

4-Oxo-4-((3-(4,4,5,5-tetramethyl-1,3,2-dioxaborolan-2-yl)phenyl)amino)butane-1-sulfonyl azide (9e)

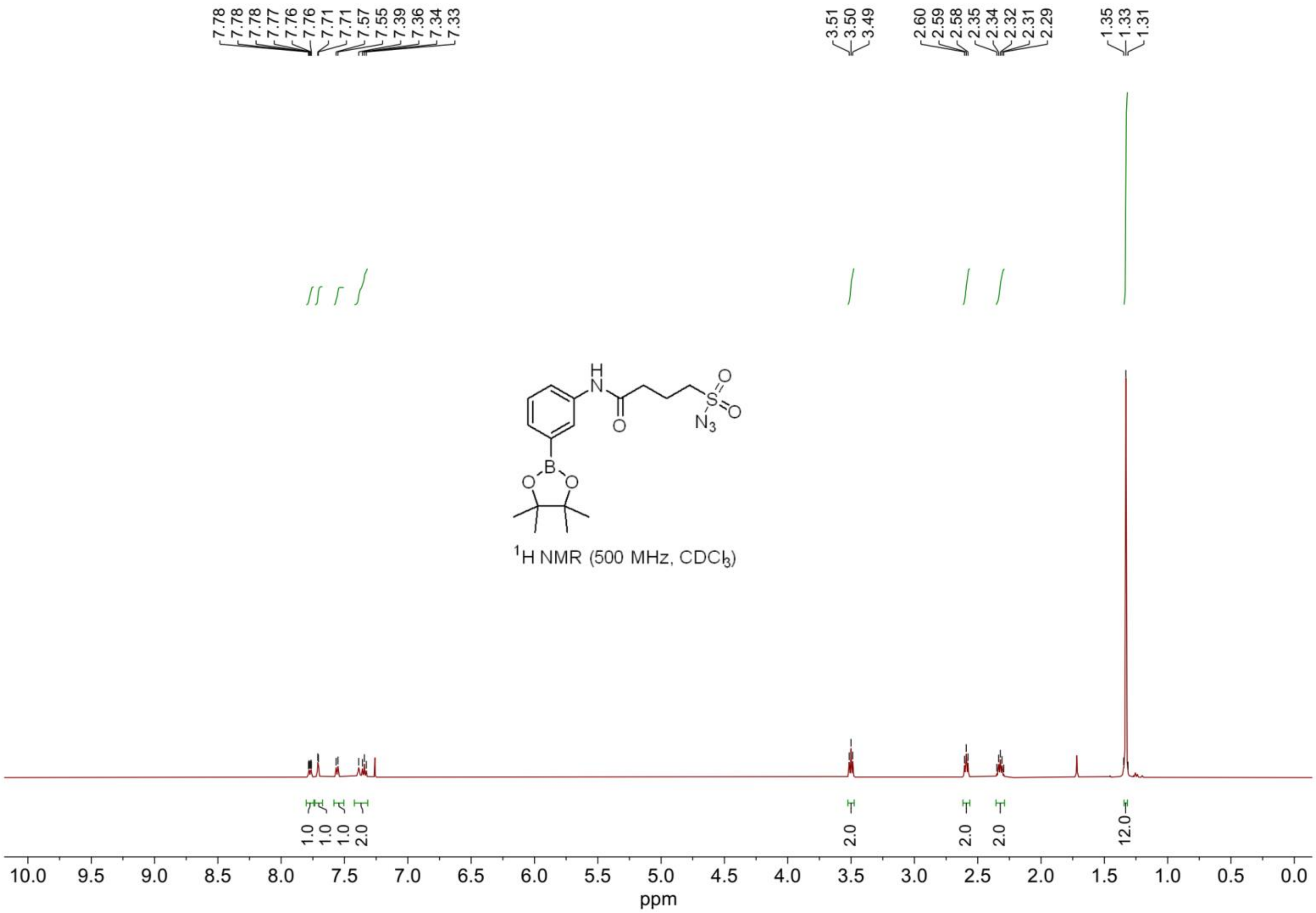

Go back to table of contents

Evaluation Only. Created with Aspose.Pdf. Copyright 2002-2014 Aspose Pty Ltd.

4-Oxo-4-((3-(4,4,5,5-tetramethyl-1,3,2-dioxaborolan-2-yl)phenyl)amino)butane-1-sulfonyl azide (9e)

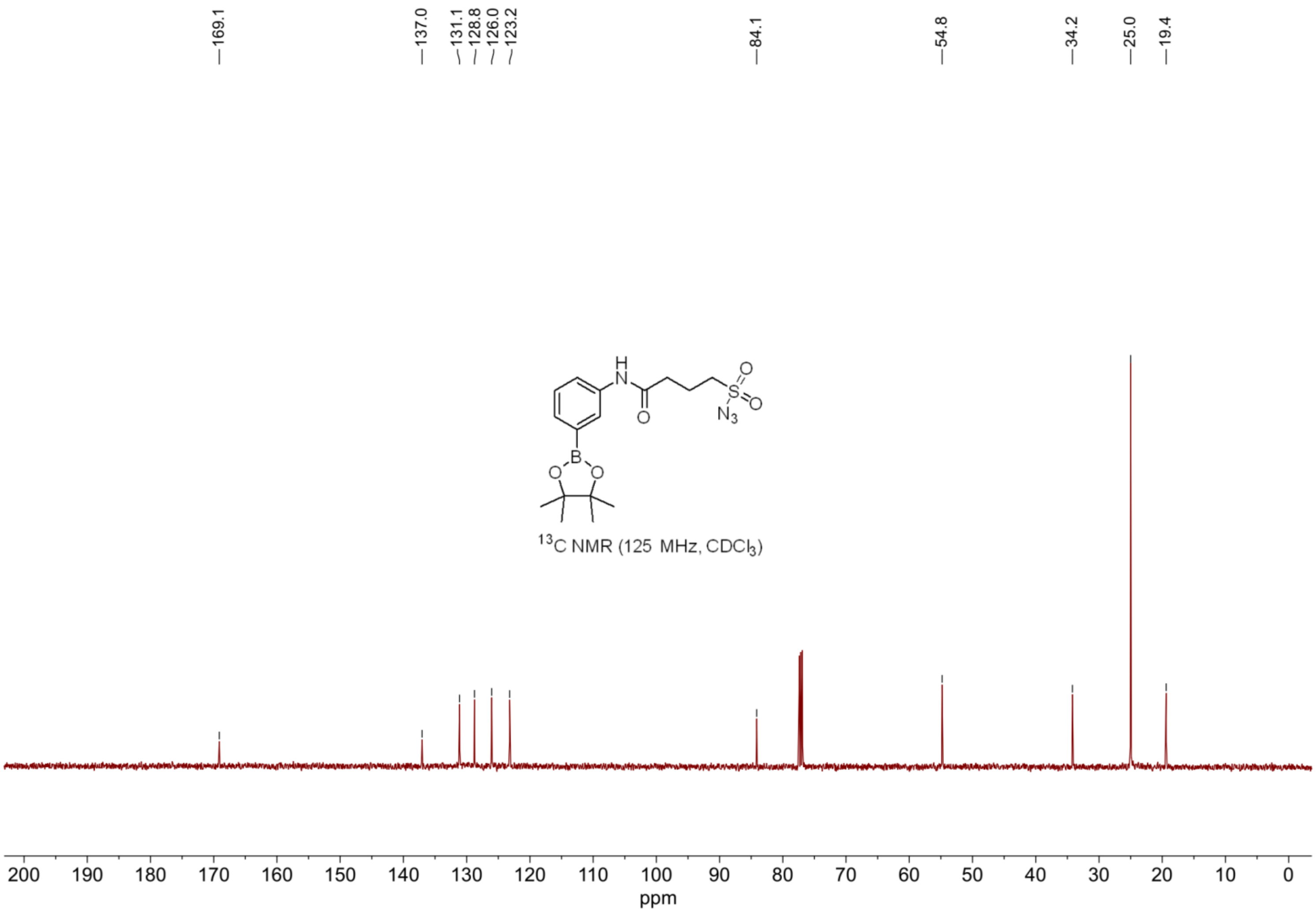

Go back to table of contents

Evaluation Only. Created with Aspose.Pdf. Copyright 2002-2014 Aspose Pty Ltd.

5-(4-Chlorobenzamido)pentane-1-sulfonyl azide (9f)

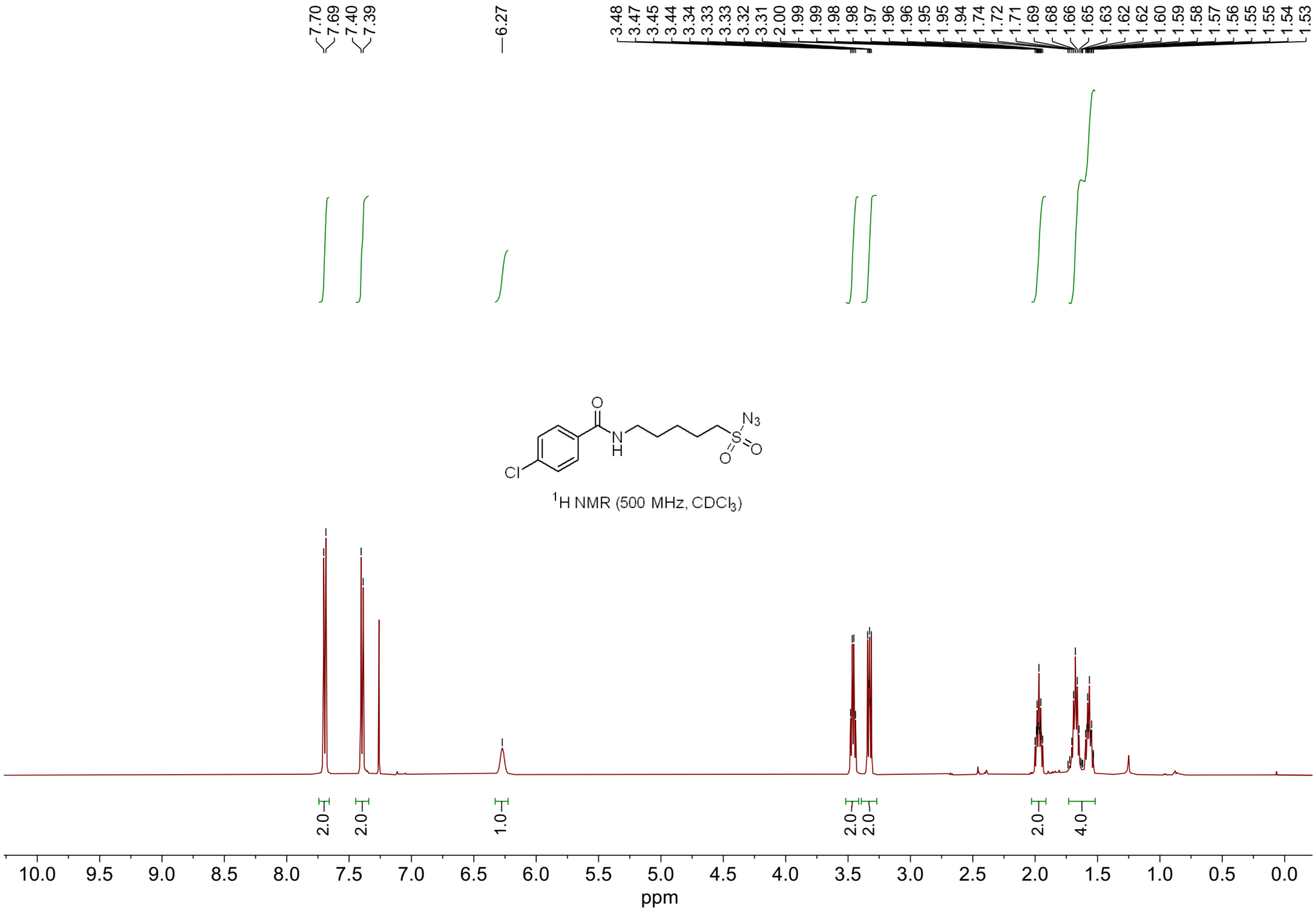

Go back to table of contents

Evaluation Only. Created with Aspose.Pdf. Copyright 2002-2014 Aspose Pty Ltd.

5-(4-Chlorobenzamido)pentane-1-sulfonyl azide (9f)

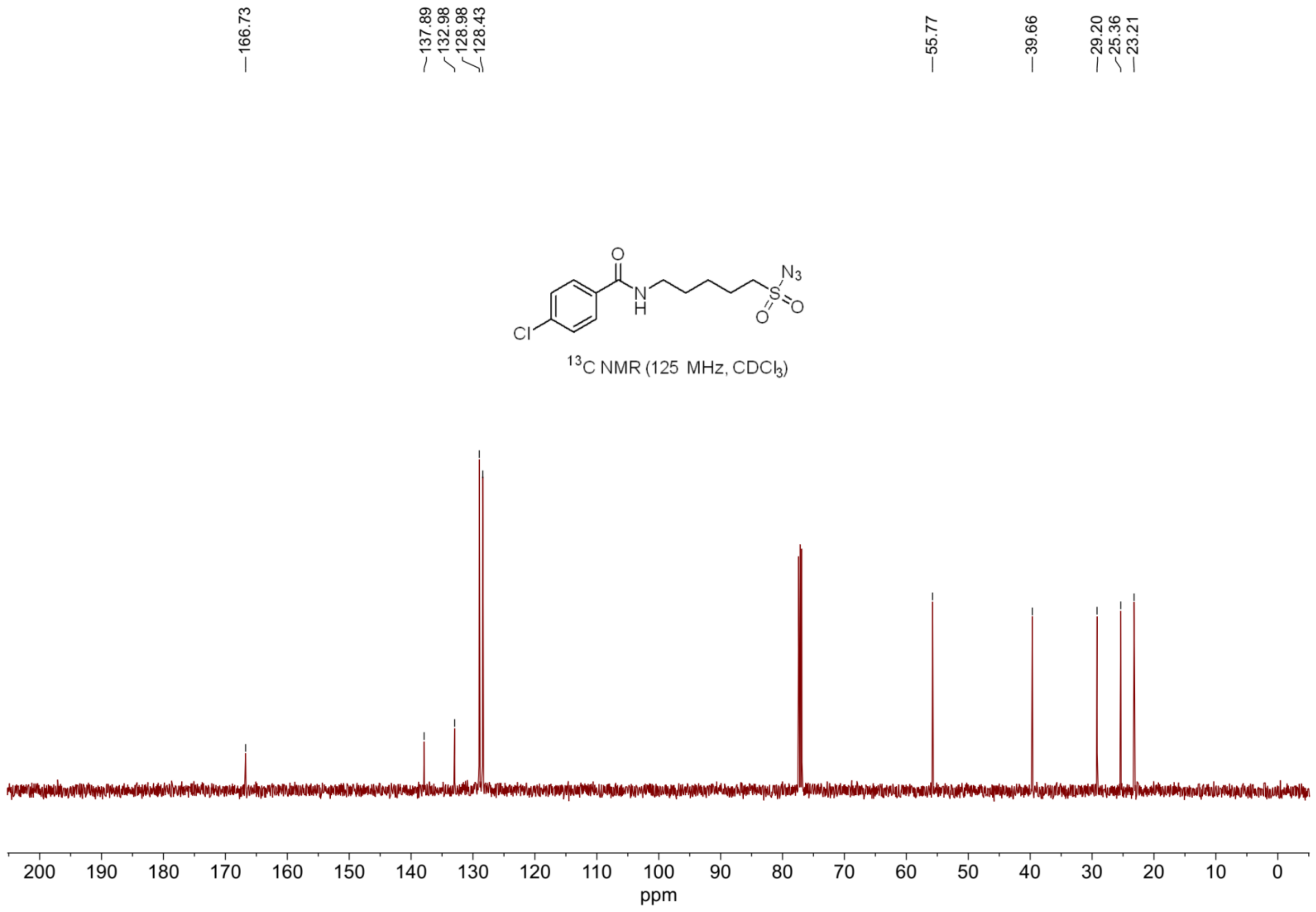

Go back to table of contents

Evaluation Only. Created with Aspose.Pdf. Copyright 2002-2014 Aspose Pty Ltd.

Pentadecane-1-sulfonyl azide (9g)

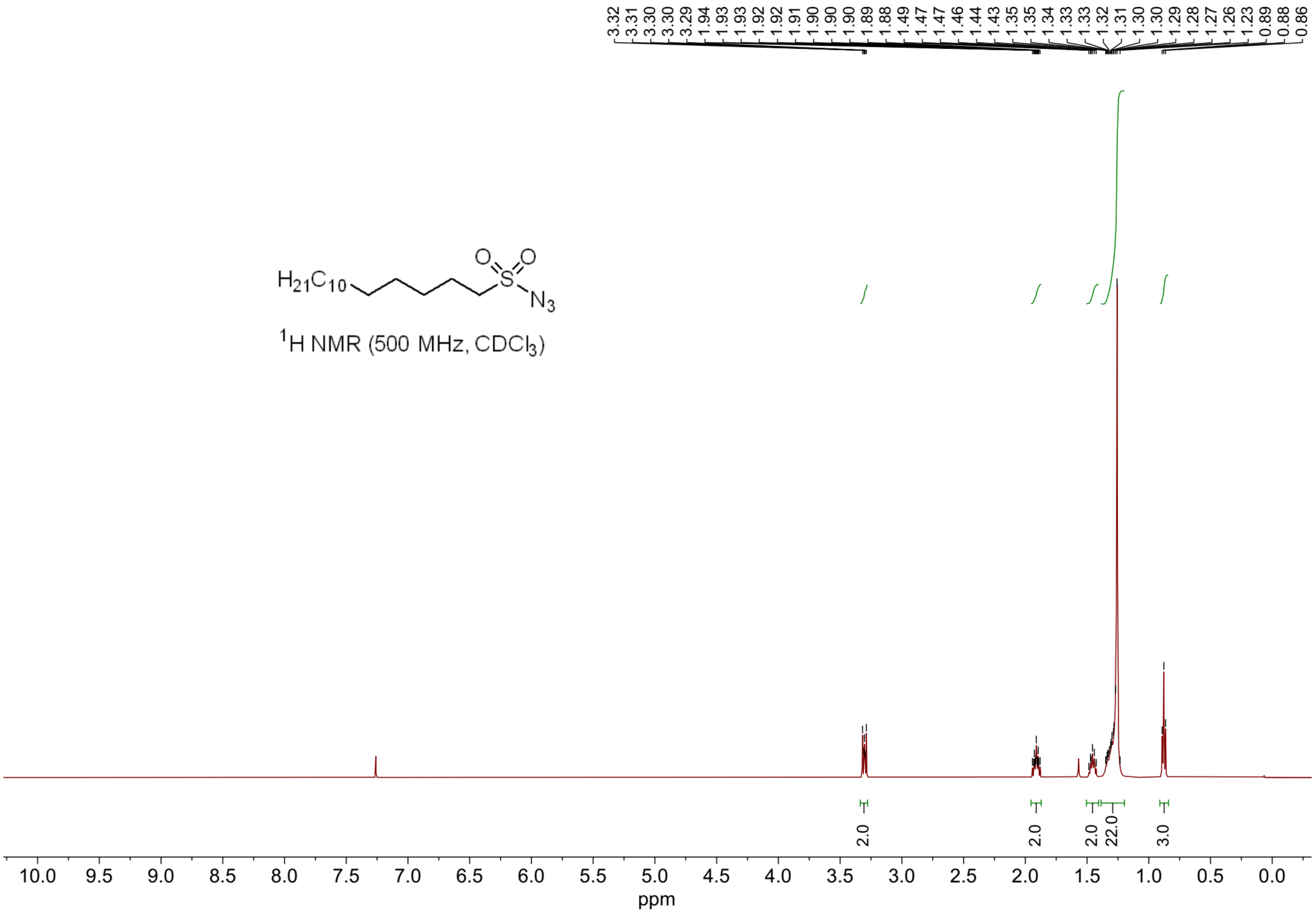

Go back to table of contents

Evaluation Only. Created with Aspose.Pdf. Copyright 2002-2014 Aspose Pty Ltd.

Pentadecane-1-sulfonyl azide (9g)

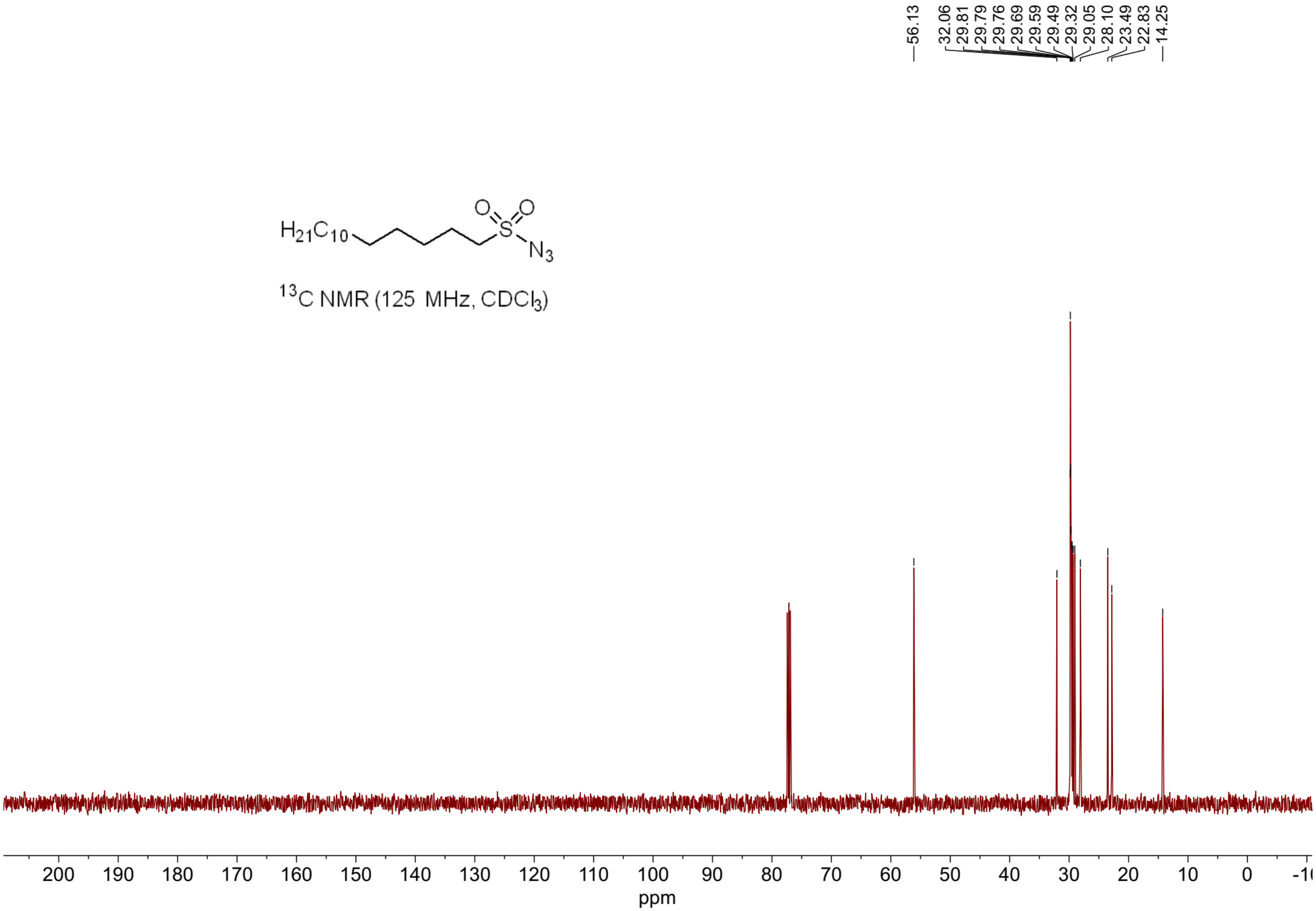

Go back to table of contents

Evaluation Only. Created with Aspose.Pdf. Copyright 2002-2014 Aspose Pty Ltd.

tert-Butyl 4-(azidosulfonyl)piperidine-1-carboxylate (9h)

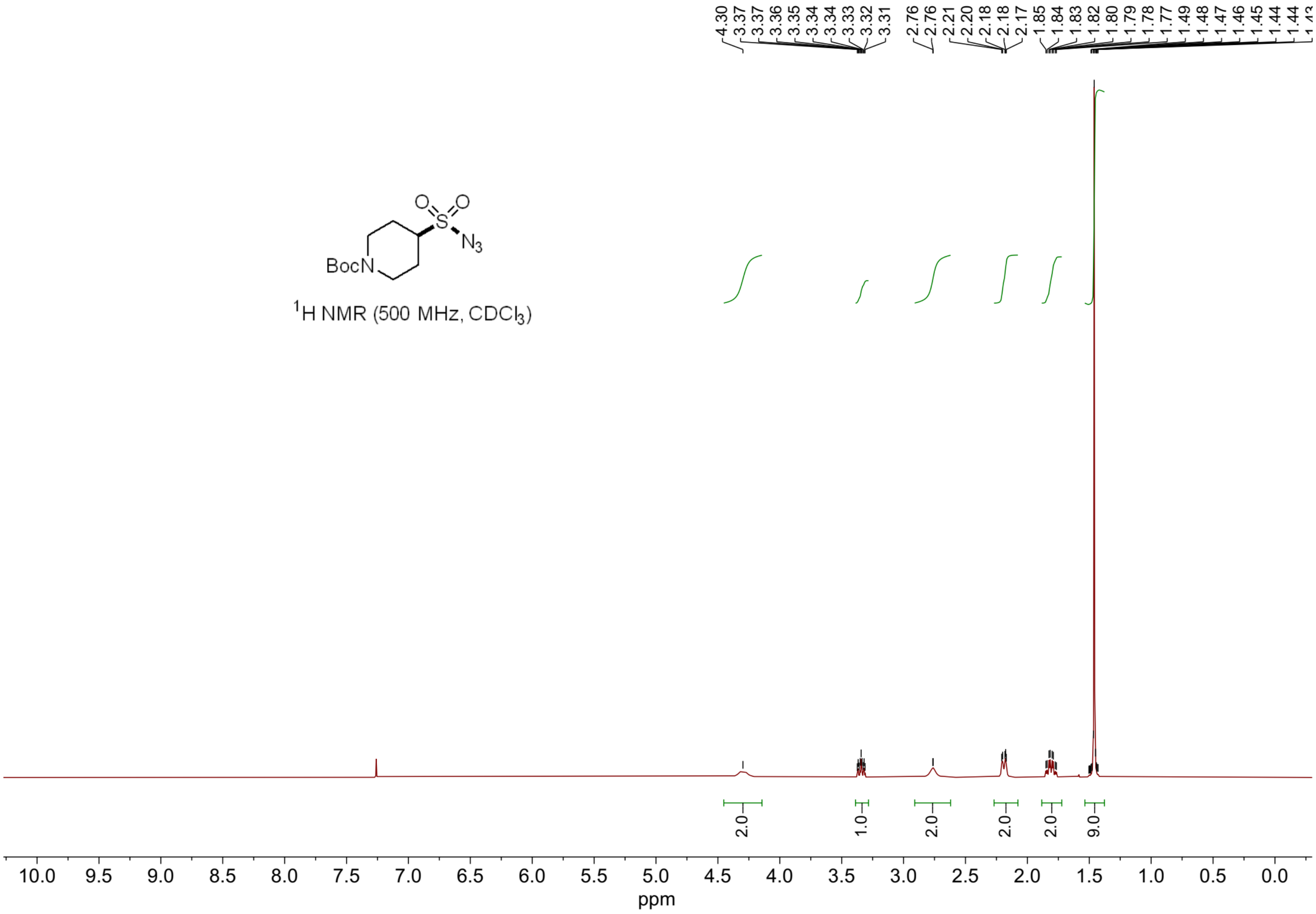

Go back to table of contents

Evaluation Only. Created with Aspose.Pdf. Copyright 2002-2014 Aspose Pty Ltd.

tert-Butyl 4-(azidosulfonyl)piperidine-1-carboxylate (9h)

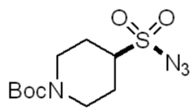

^{13}C NMR (125 MHz, $CDCl_3$)

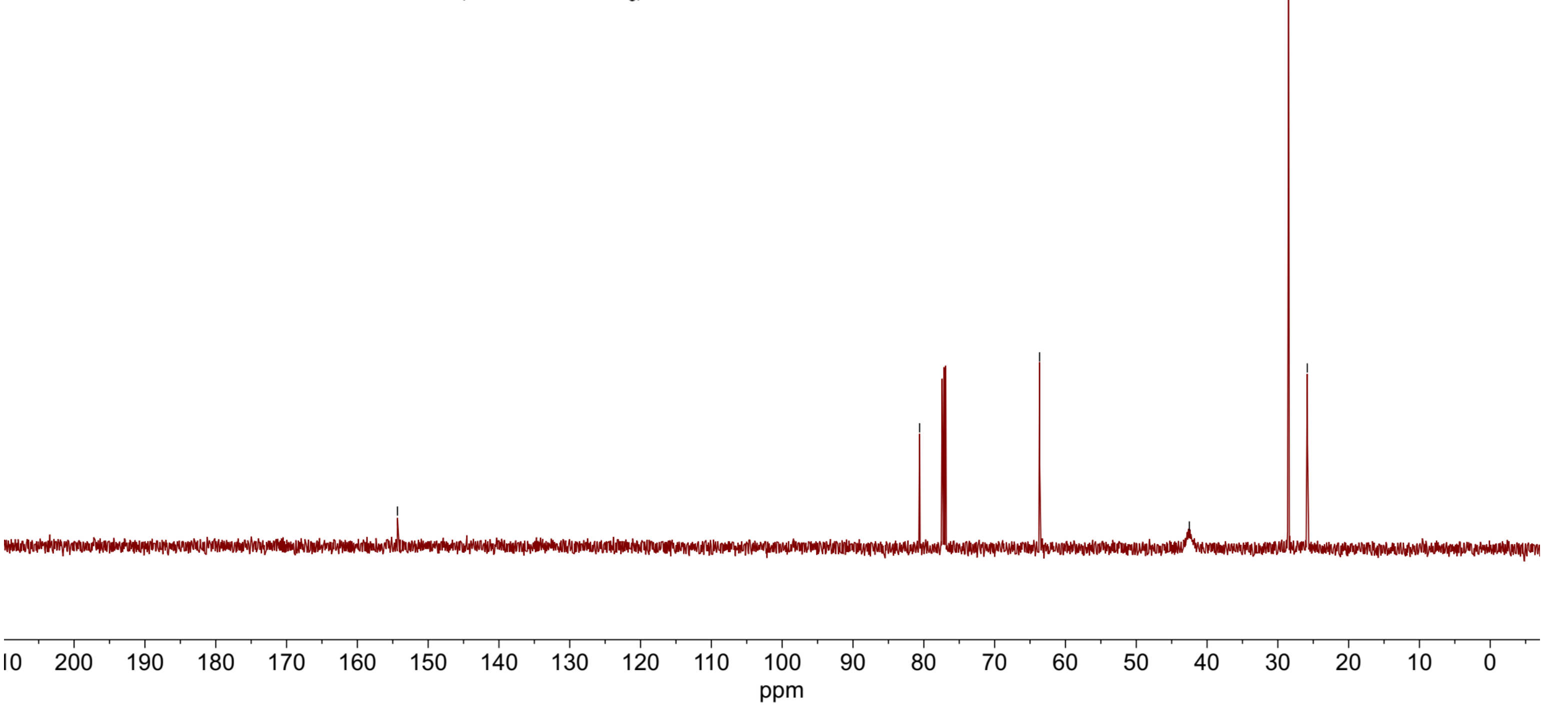

Go back to table of contents

Evaluation Only. Created with Aspose.Pdf. Copyright 2002-2014 Aspose Pty Ltd.

Tetrahydro-2*H*-pyran-4-sulfonyl azide (9i)

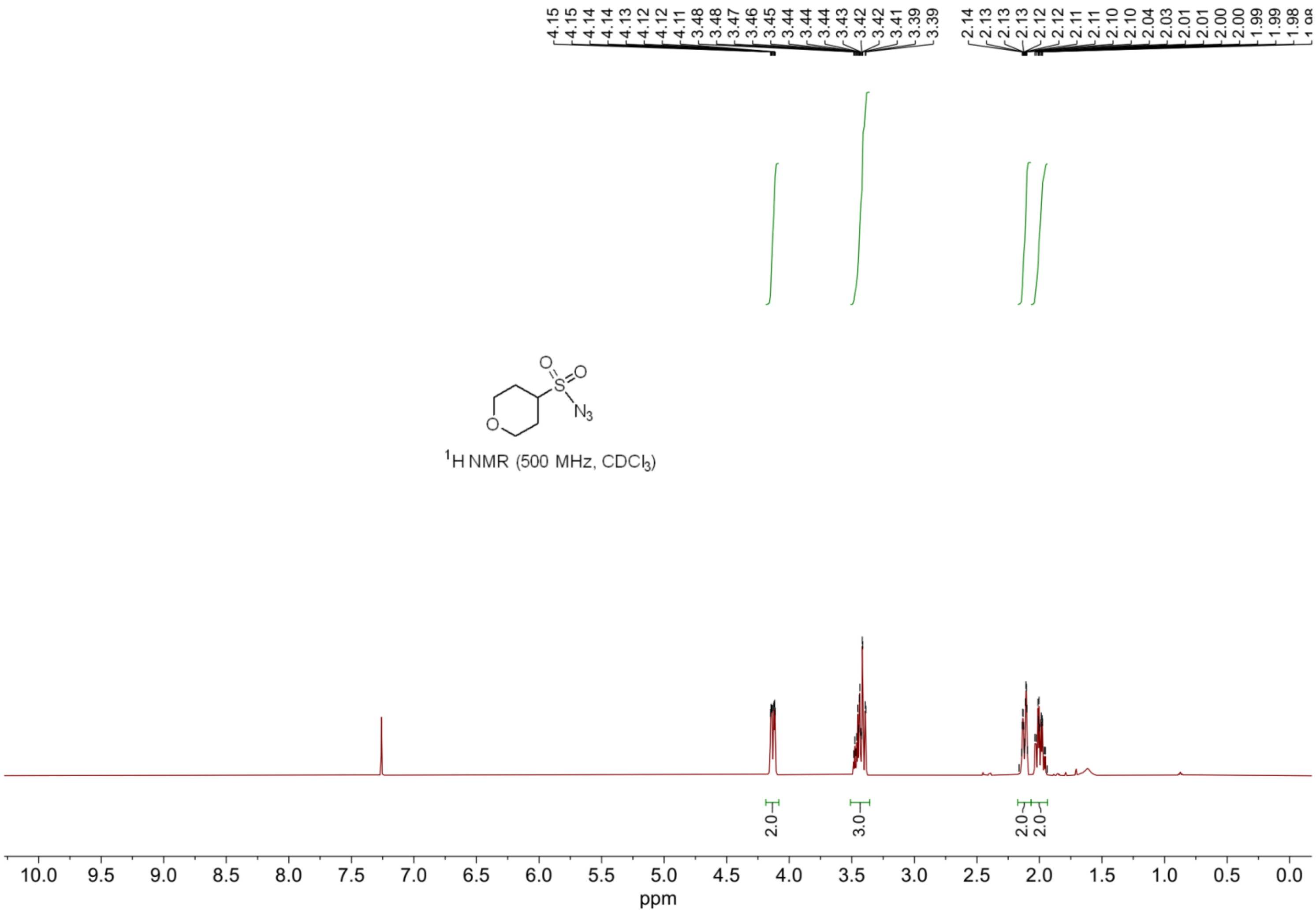

Evaluation Only. Created with Aspose.Pdf. Copyright 2002-2014 Aspose Pty Ltd.

Tetrahydro-2*H*-pyran-4-sulfonyl azide (9i)

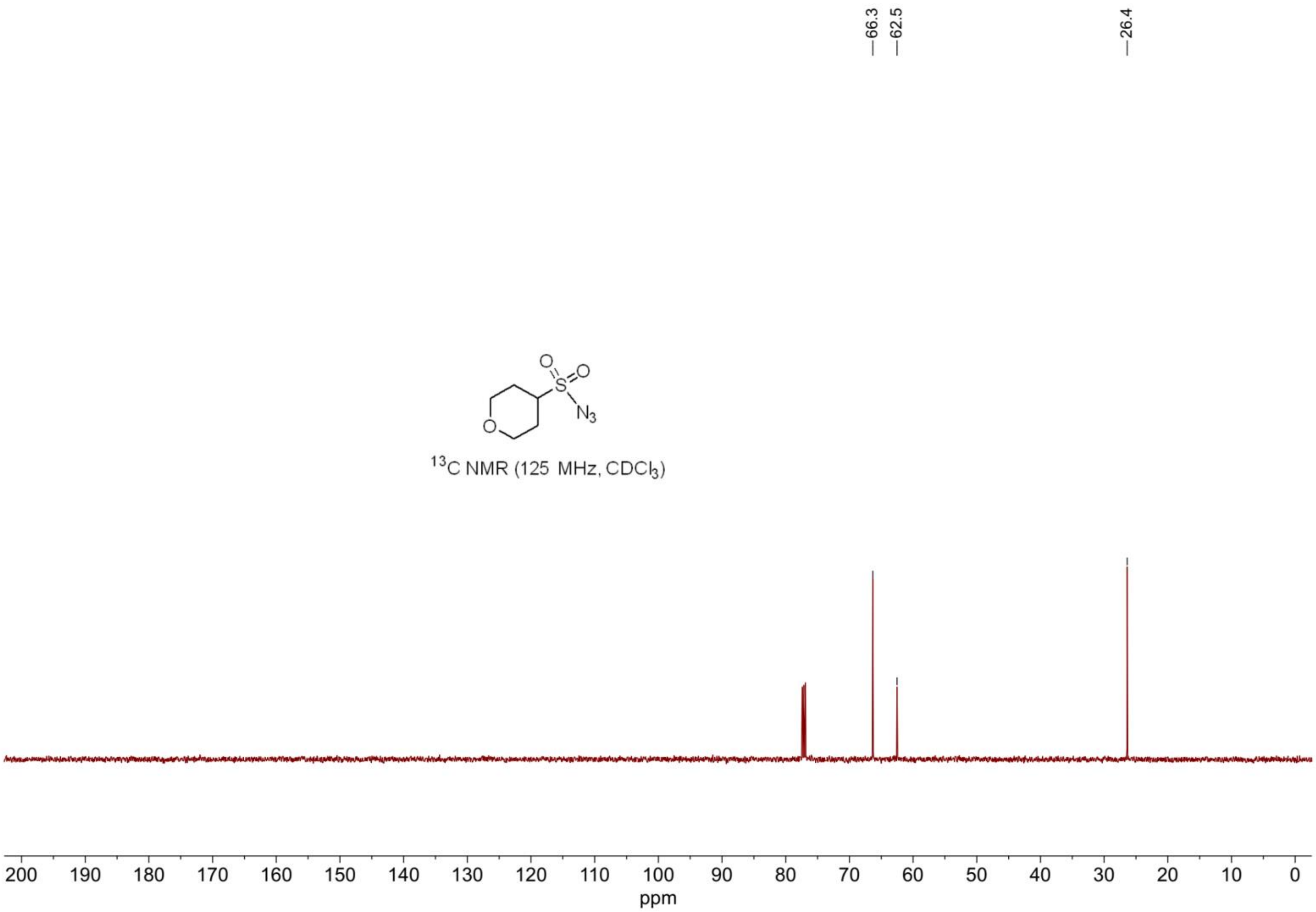

Go back to table of contents

Evaluation Only. Created with Aspose.Pdf. Copyright 2002-2014 Aspose Pty Ltd.

4-Methyltetrahydro-2*H*-pyran-4-sulfonyl azide (9j)

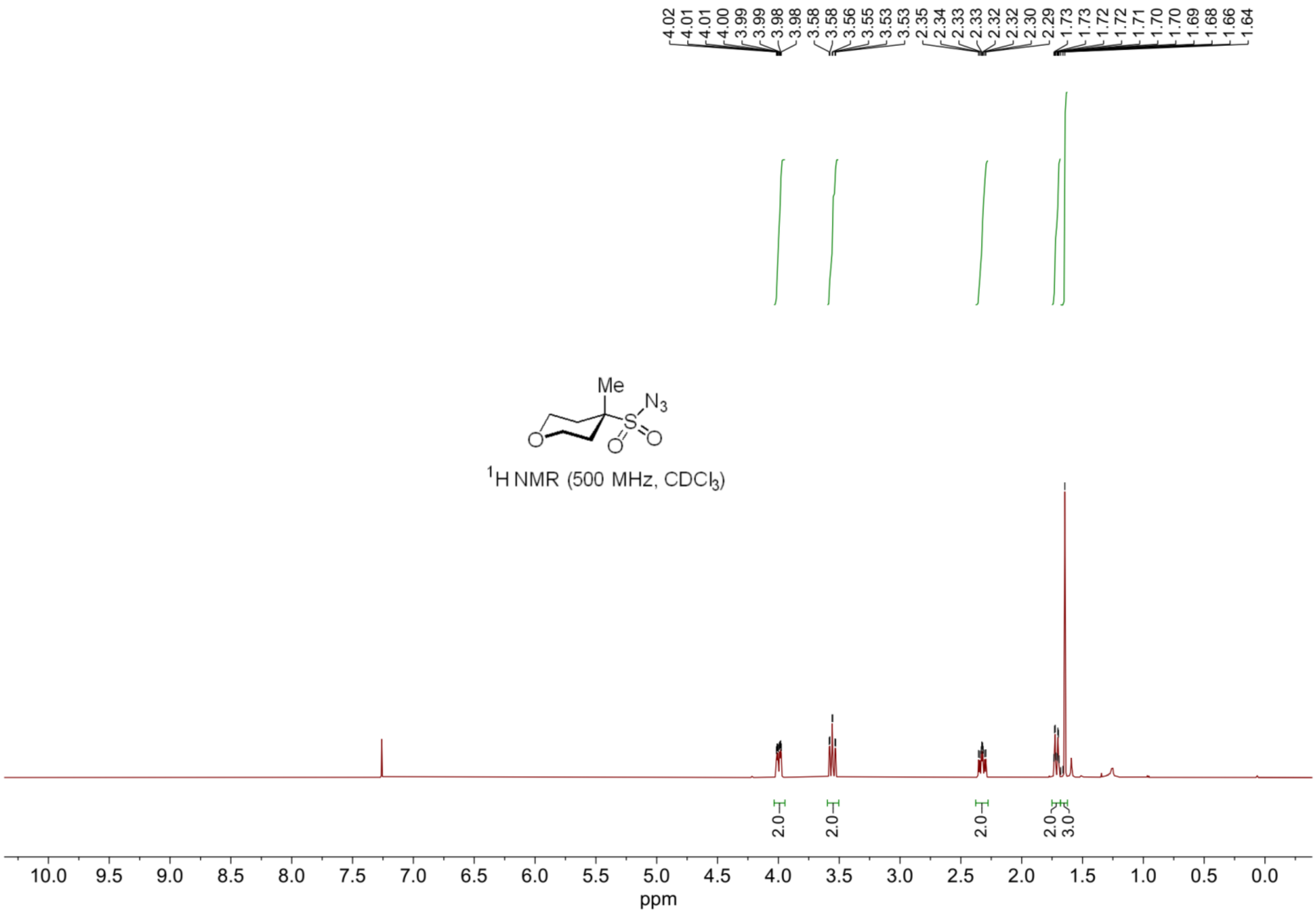

Go back to table of contents

Evaluation Only. Created with Aspose.Pdf. Copyright 2002-2014 Aspose Pty Ltd.

4-Methyltetrahydro-2*H*-pyran-4-sulfonyl azide (9j)

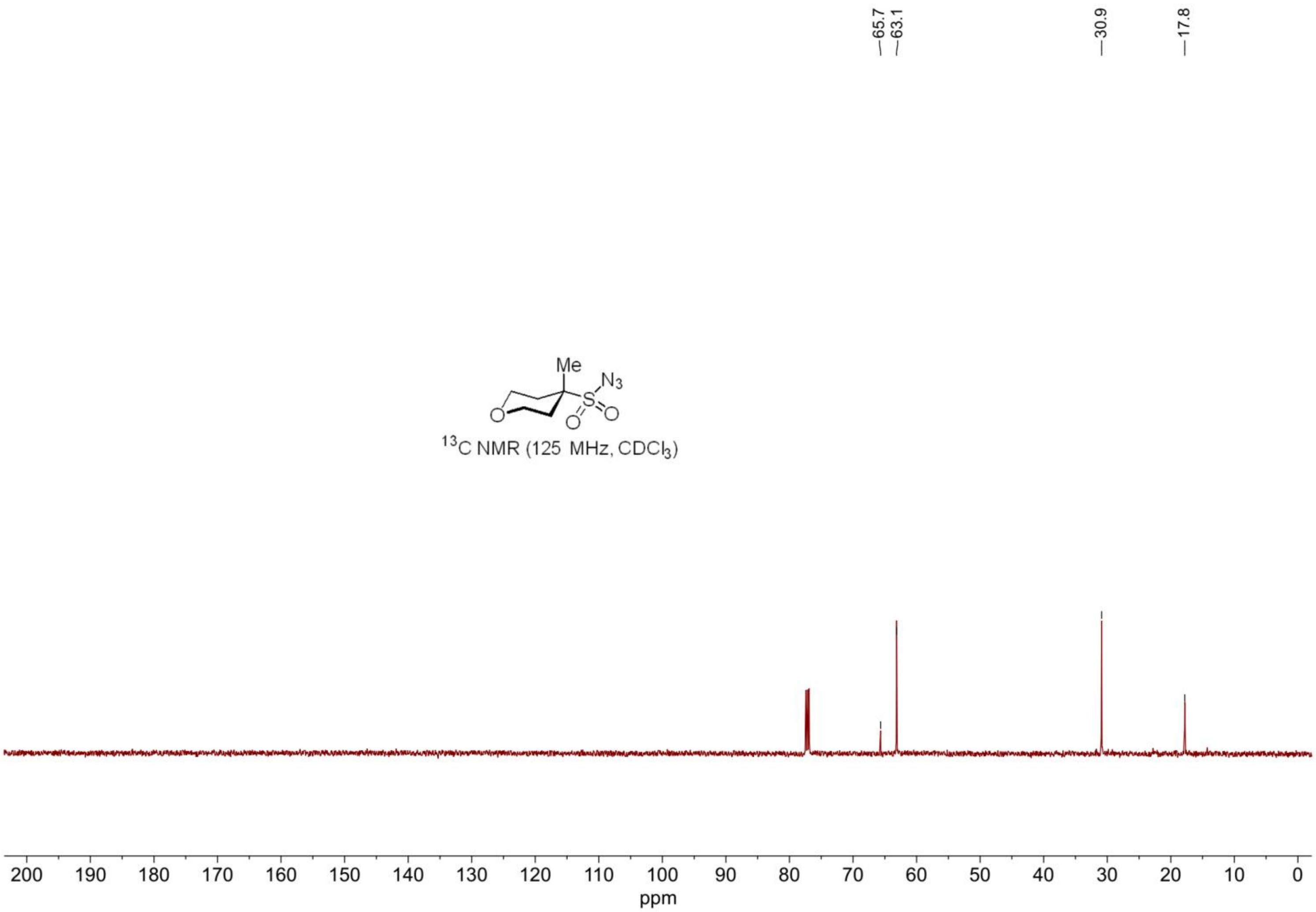

Go back to table of contents

Evaluation Only. Created with Aspose.Pdf. Copyright 2002-2014 Aspose Pty Ltd.

Heptane-4-sulfonyl azide (9k)

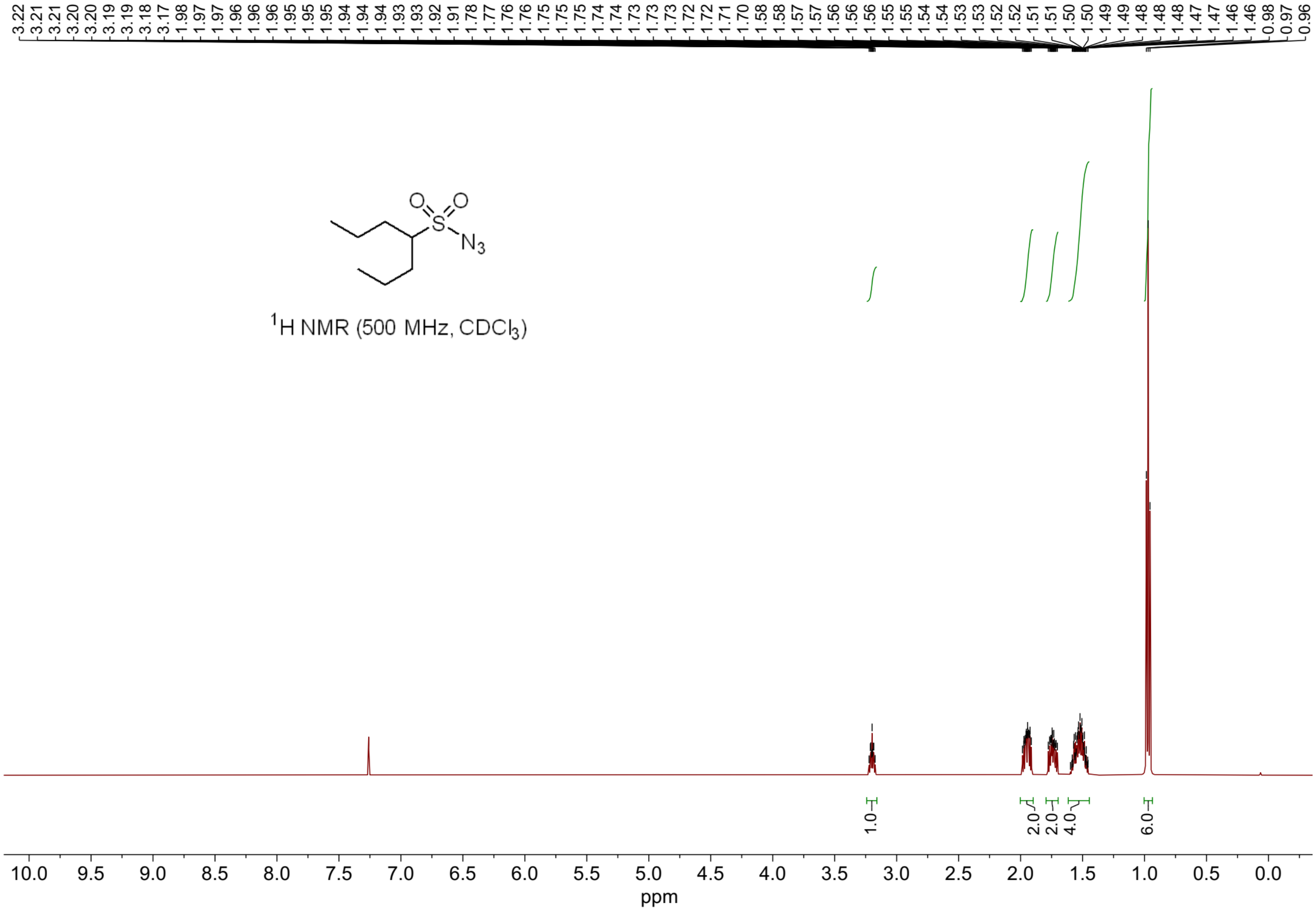

Go back to table of contents

Evaluation Only. Created with Aspose.Pdf. Copyright 2002-2014 Aspose Pty Ltd.

Heptane-4-sulfonyl azide (9k)

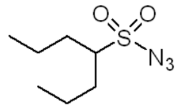

^{13}C NMR (125 MHz, $CDCl_3$)

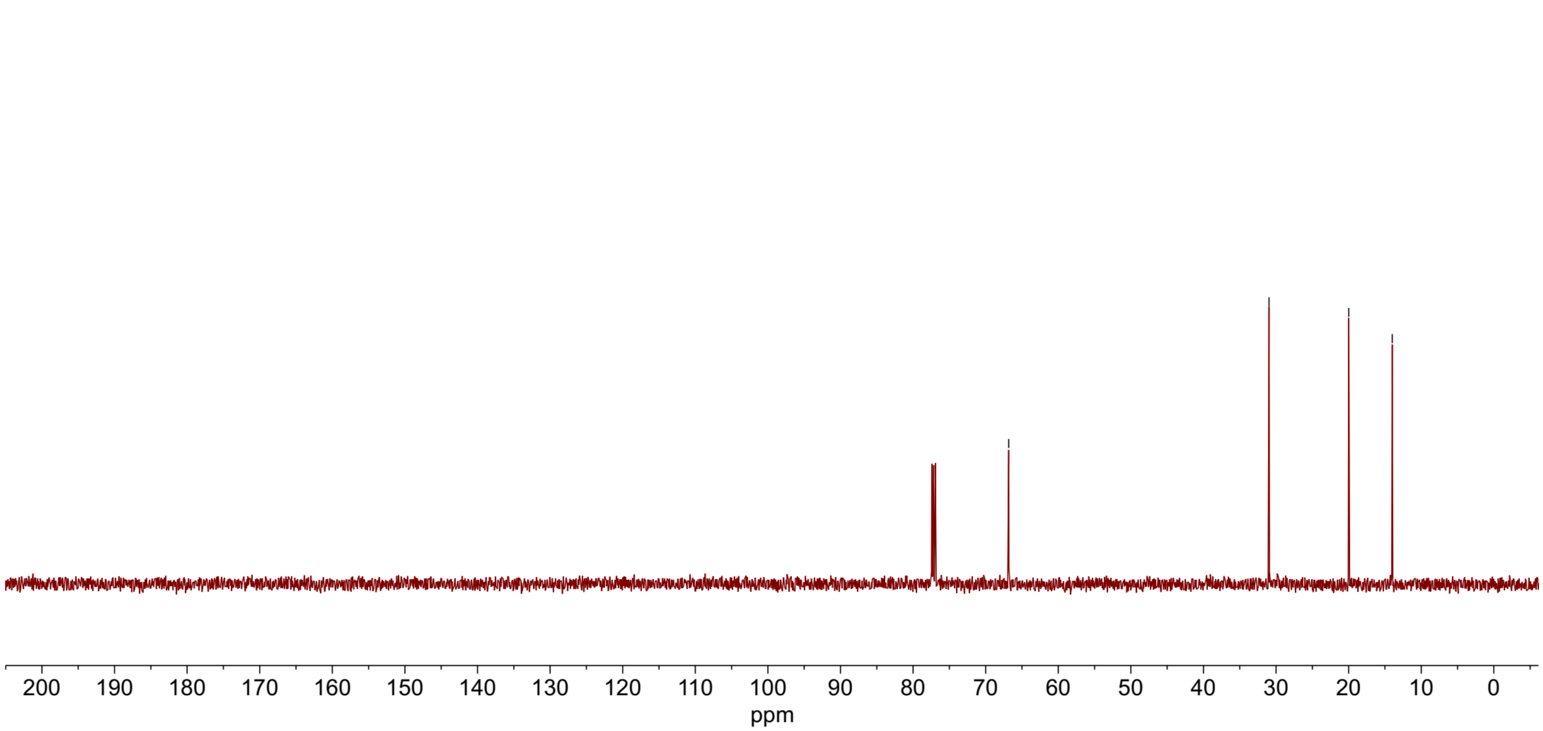

Go back to table of contents

Evaluation Only. Created with Aspose.Pdf. Copyright 2002-2014 Aspose Pty Ltd.

3*R*,7*R*,8*R*,9*S*,10*S*,13*R*,14*S*,17*R*)-10,13-Dimethyl-17-((*R*)-4-(morpholinosulfonyl)butan-2-yl)hexadecahydro-1*H*-cyclopenta[*a*]phenanthrene-3,7-diyl diacetate (10a)

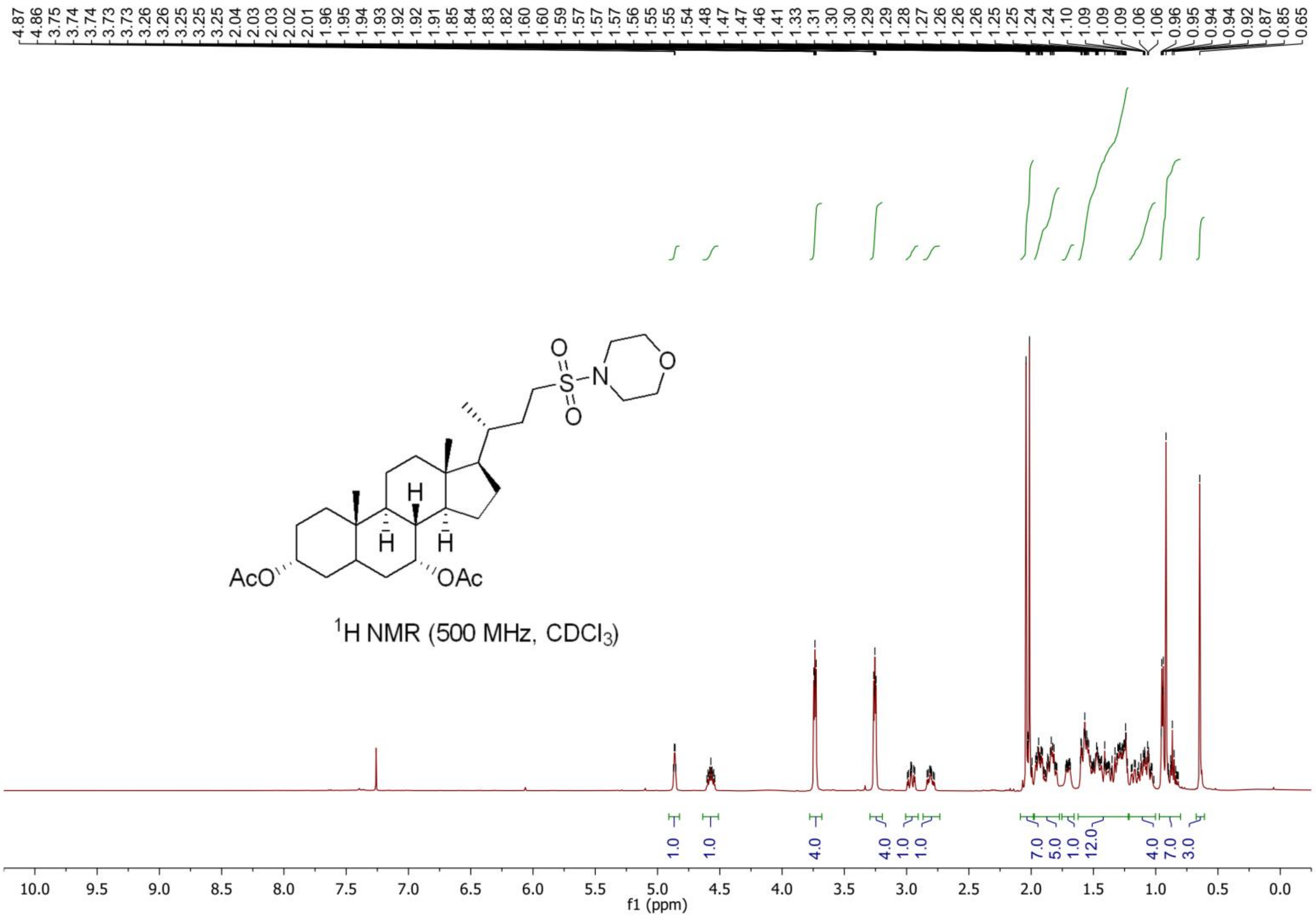

Go back to table of contents

Evaluation Only. Created with Aspose.Pdf. Copyright 2002-2014 Aspose Pty Ltd.

(3*R*,7*R*,8*R*,9*S*,10*S*,13*R*,14*S*,17*R*)-10,13-Dimethyl-17-((*R*)-4-(morpholinosulfonyl)butan-2-yl)hexadecahydro-1*H*-cyclopenta[*a*]phenanthrene-3,7-diyl diacetate (10a)

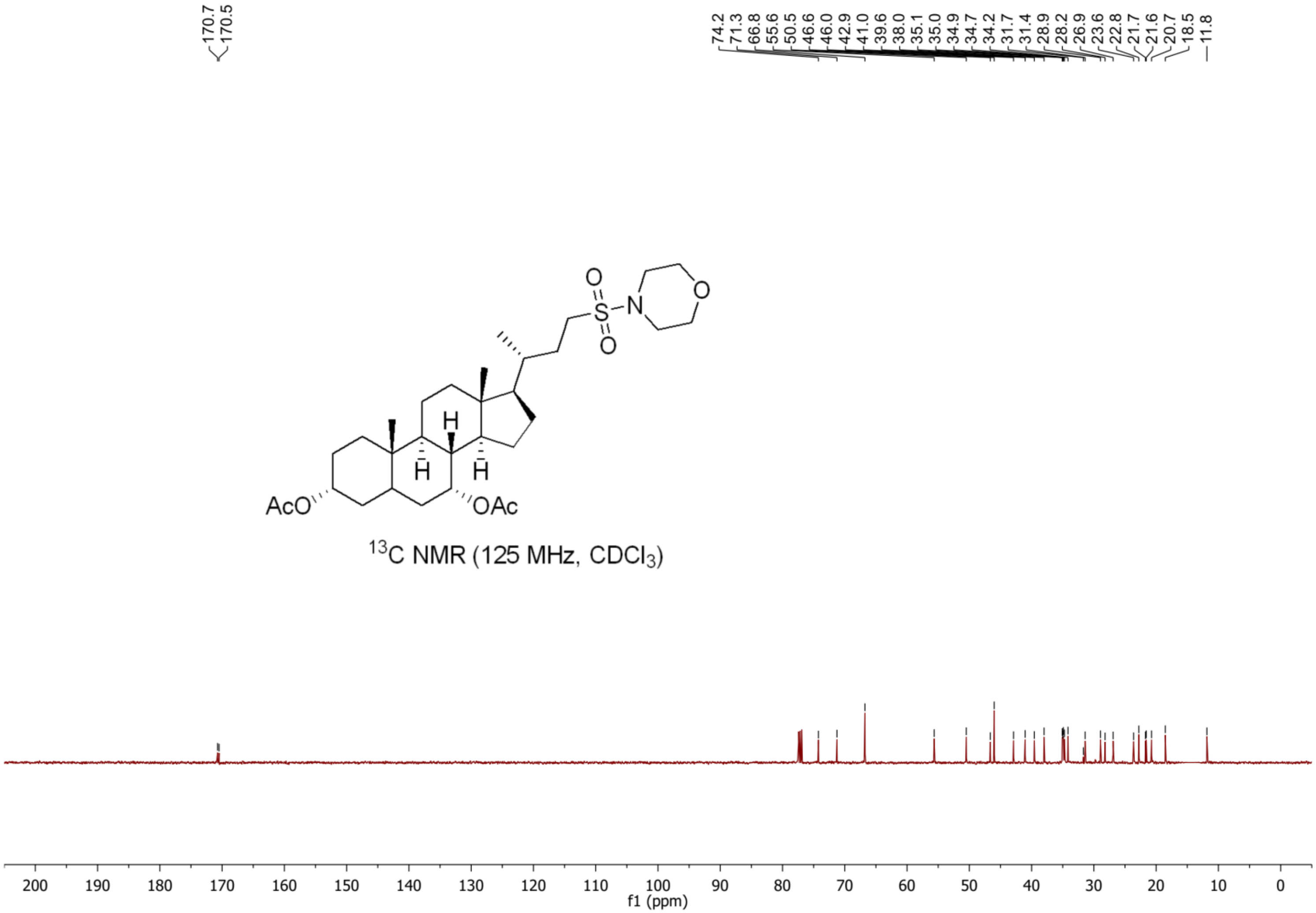

Go back to table of contents

Evaluation Only. Created with Aspose.Pdf. Copyright 2002-2014 Aspose Pty Ltd.

15-(Morpholinosulfonyl)pentadecane-1,7,8-triyl triacetate (10b)

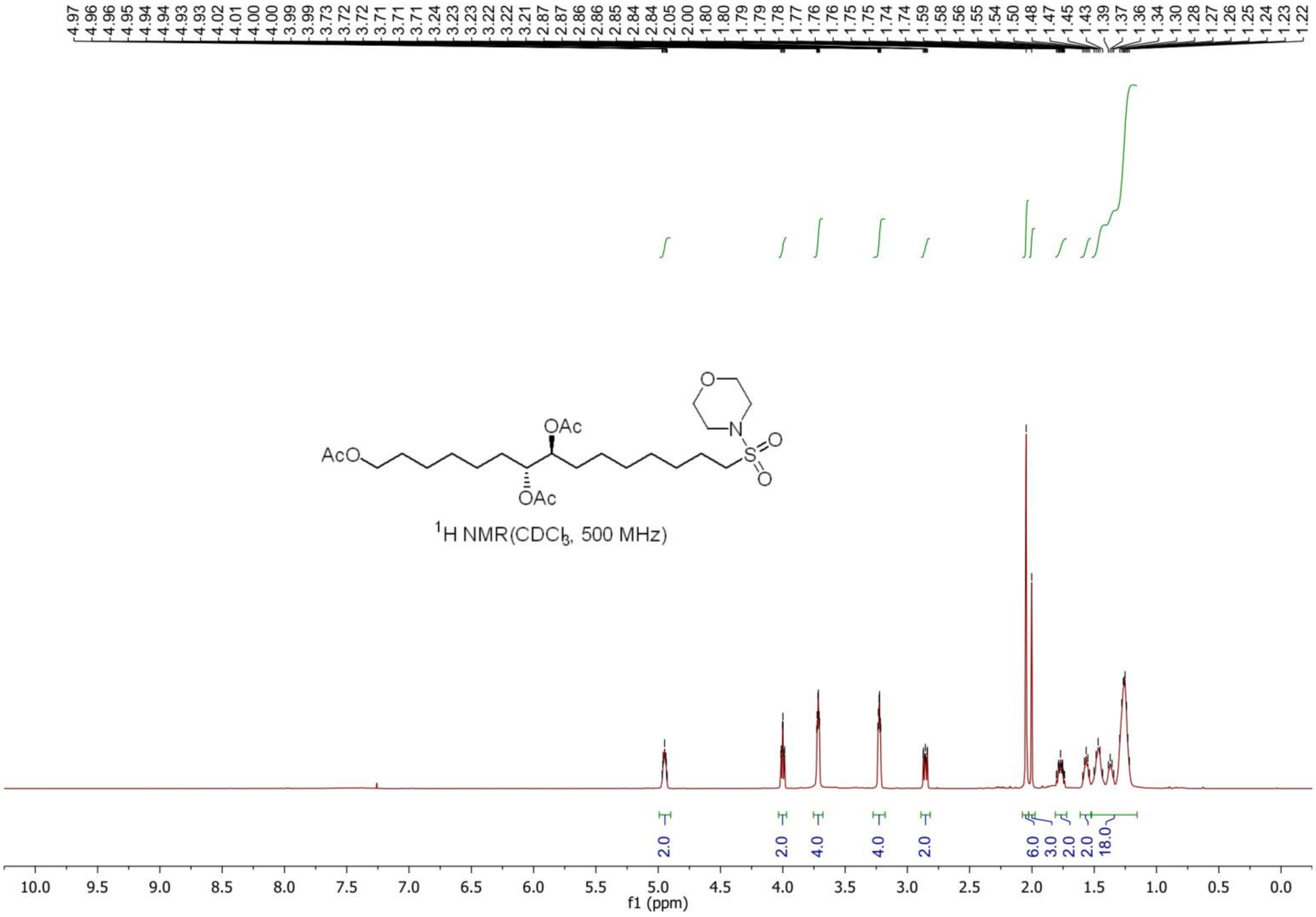

Go back to table of contents

Evaluation Only. Created with Aspose.Pdf. Copyright 2002-2014 Aspose Pty Ltd.

15-(Morpholinosulfonyl)pentadecane-1,7,8-triyl triacetate (10b)

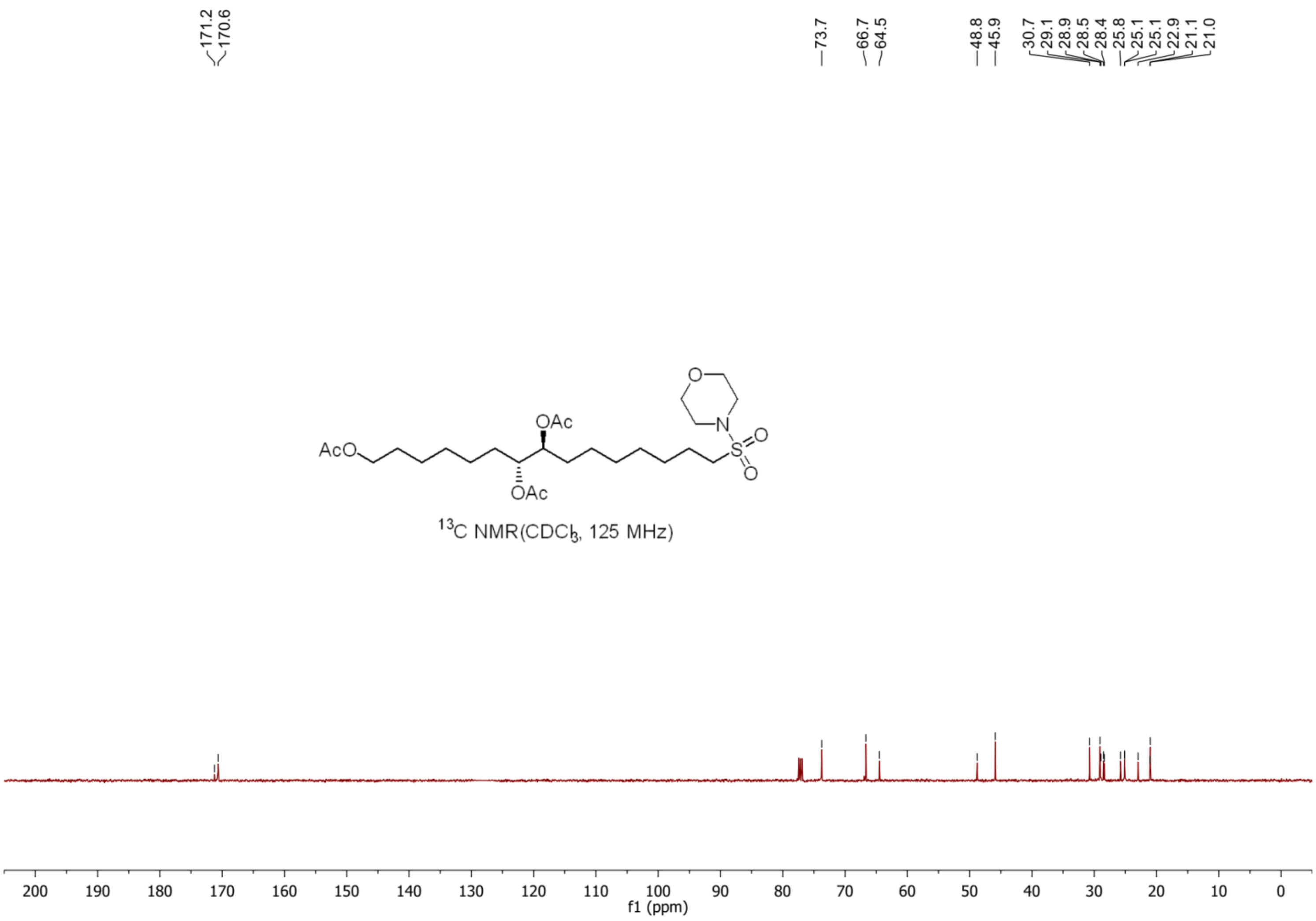

Go back to table of contents

Evaluation Only. Created with Aspose.Pdf. Copyright 2002-2014 Aspose Pty Ltd.

(1*S*,2*S*,4*aR*,4*bR*,7*S*,9*aS*,10*S*,10*aR*)-1-Methyl-8-methylene-10-(morpholinosulfonyl)-13-oxo-1,2,5,6,8,9,10,10a-octahydro-4a,1-(epoxymethano)-7,9a-methanobenzo[*a*]azulene-2,7(4*bH*)-diyl diacetate (10c)

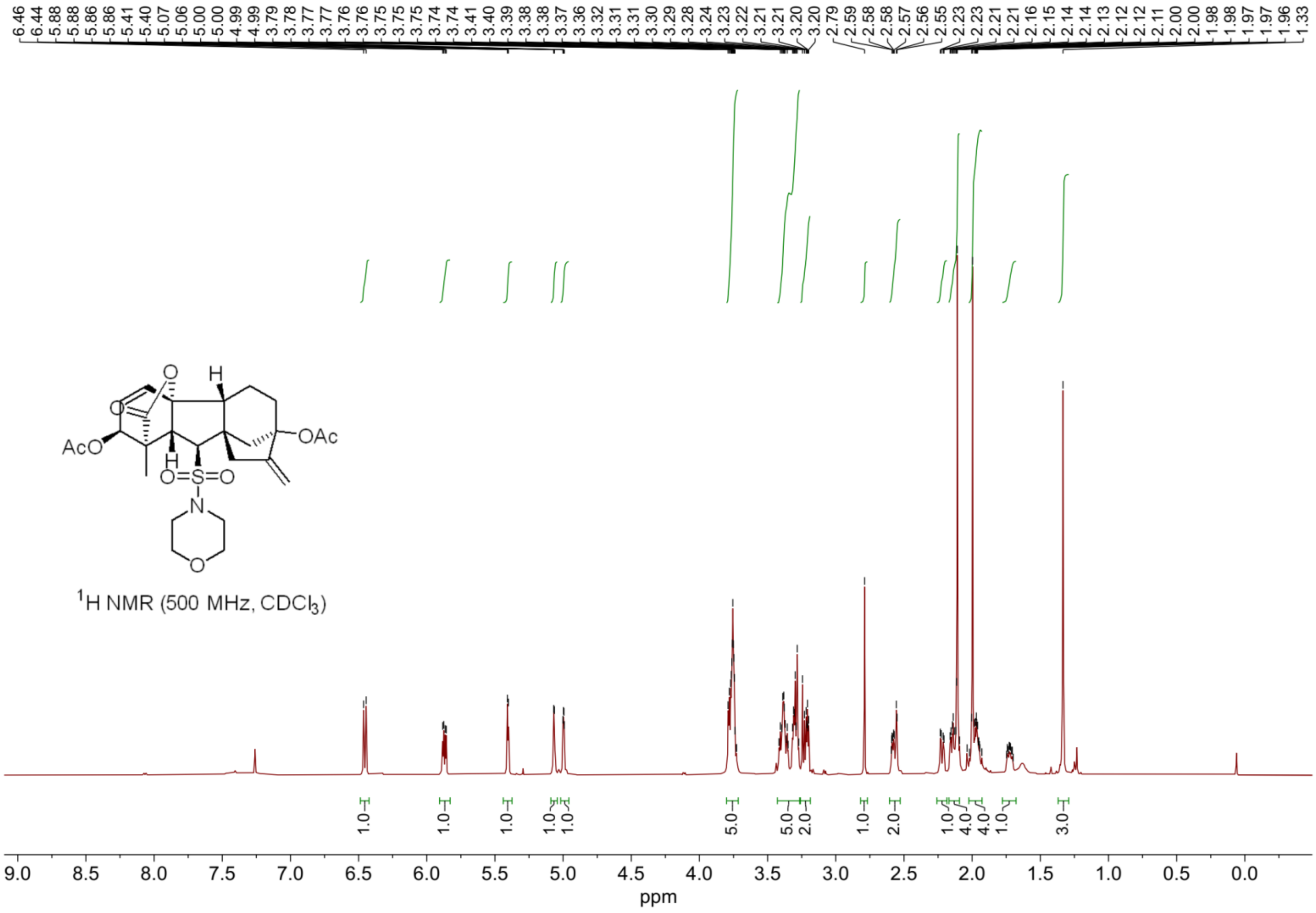

Go back to table of contents

Evaluation Only. Created with Aspose.Pdf. Copyright 2002-2014 Aspose Pty Ltd.

(1*S*,2*S*,4a*R*,4b*R*,7*S*,9a*S*,10*S*,10a*R*)-1-Methyl-8-methylene-10-(morpholinosulfonyl)-13-oxo-1,2,5,6,8,9,10,10a-octahydro-4a,1-(epoxymethano)-7,9a-methanobenzo[*a*]azulene-2,7(4b*H*)-diyl diacetate (10c)

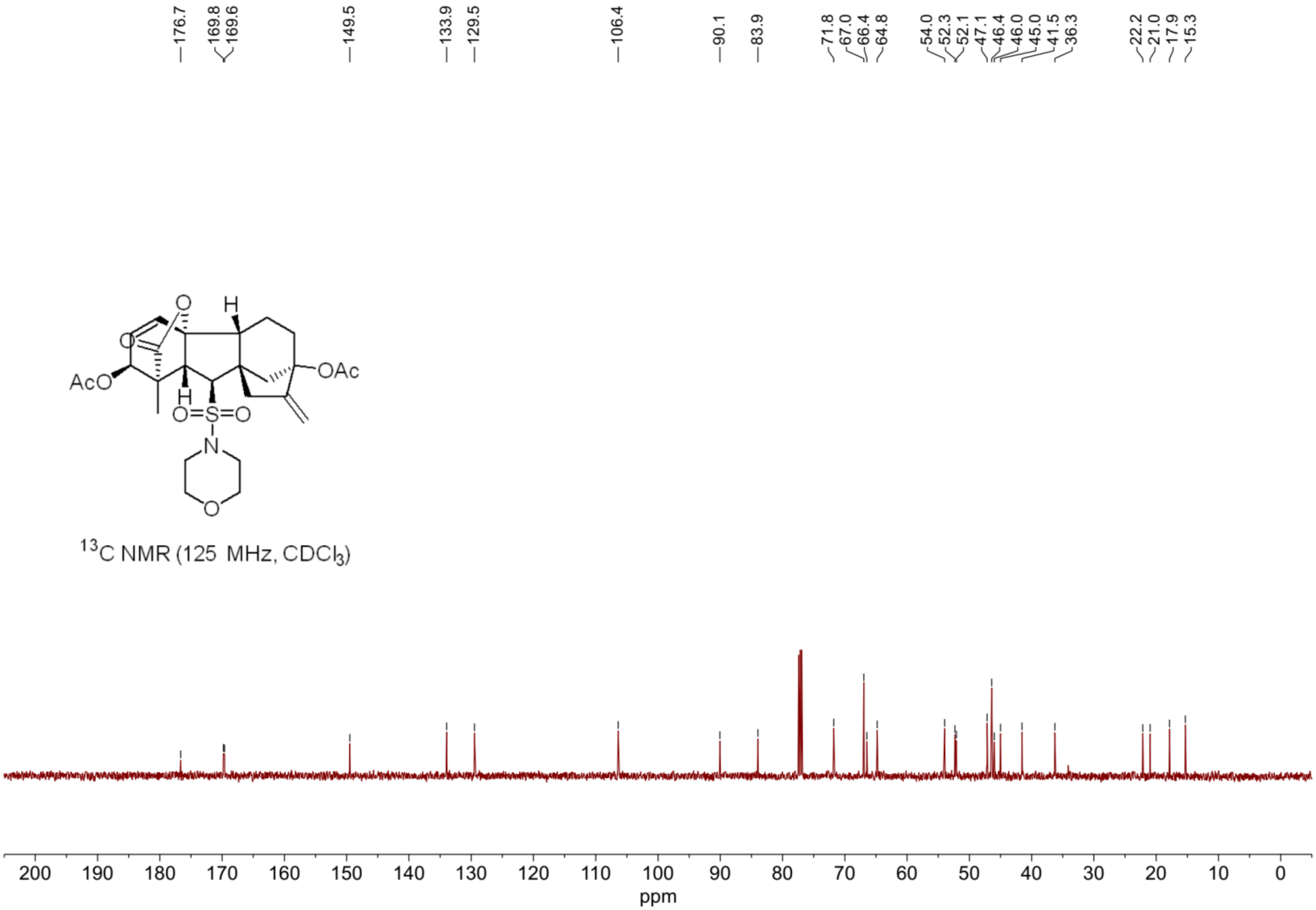

^{13}C NMR (125 MHz, $CDCl_3$)

Go back to table of contents

Evaluation Only. Created with Aspose.Pdf. Copyright 2002-2014 Aspose Pty Ltd.

N-(4-(3-ethyl-2,6-dioxopiperidin-3-yl)phenyl)-4-oxo-4-phenylbutane-1-sulfonamide (10d)

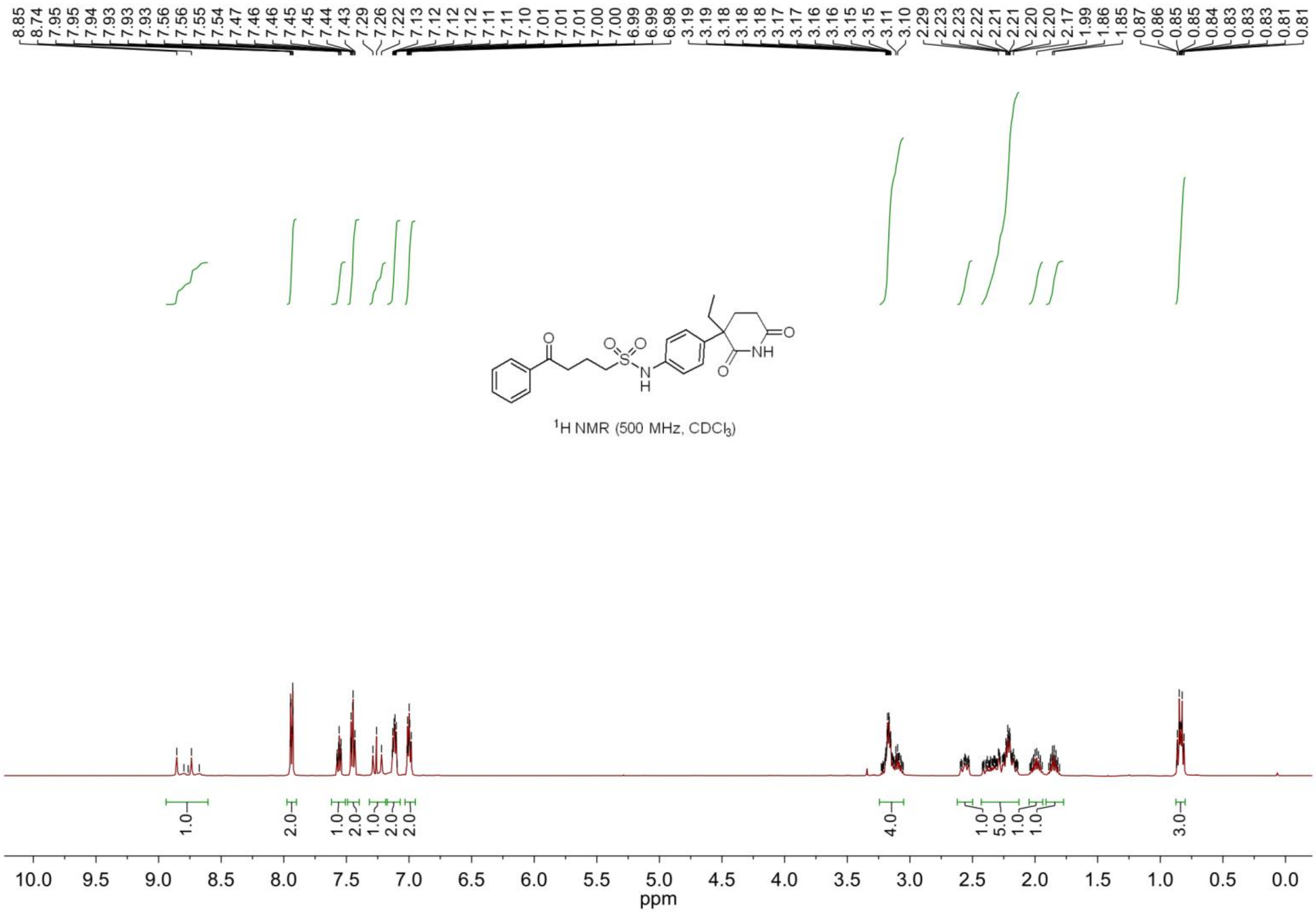

Go back to table of contents

Evaluation Only. Created with Aspose.Pdf. Copyright 2002-2014 Aspose Pty Ltd.

N-(4-(3-ethyl-2,6-dioxopiperidin-3-yl)phenyl)-4-oxo-4-phenylbutane-1-sulfonamide (10d)

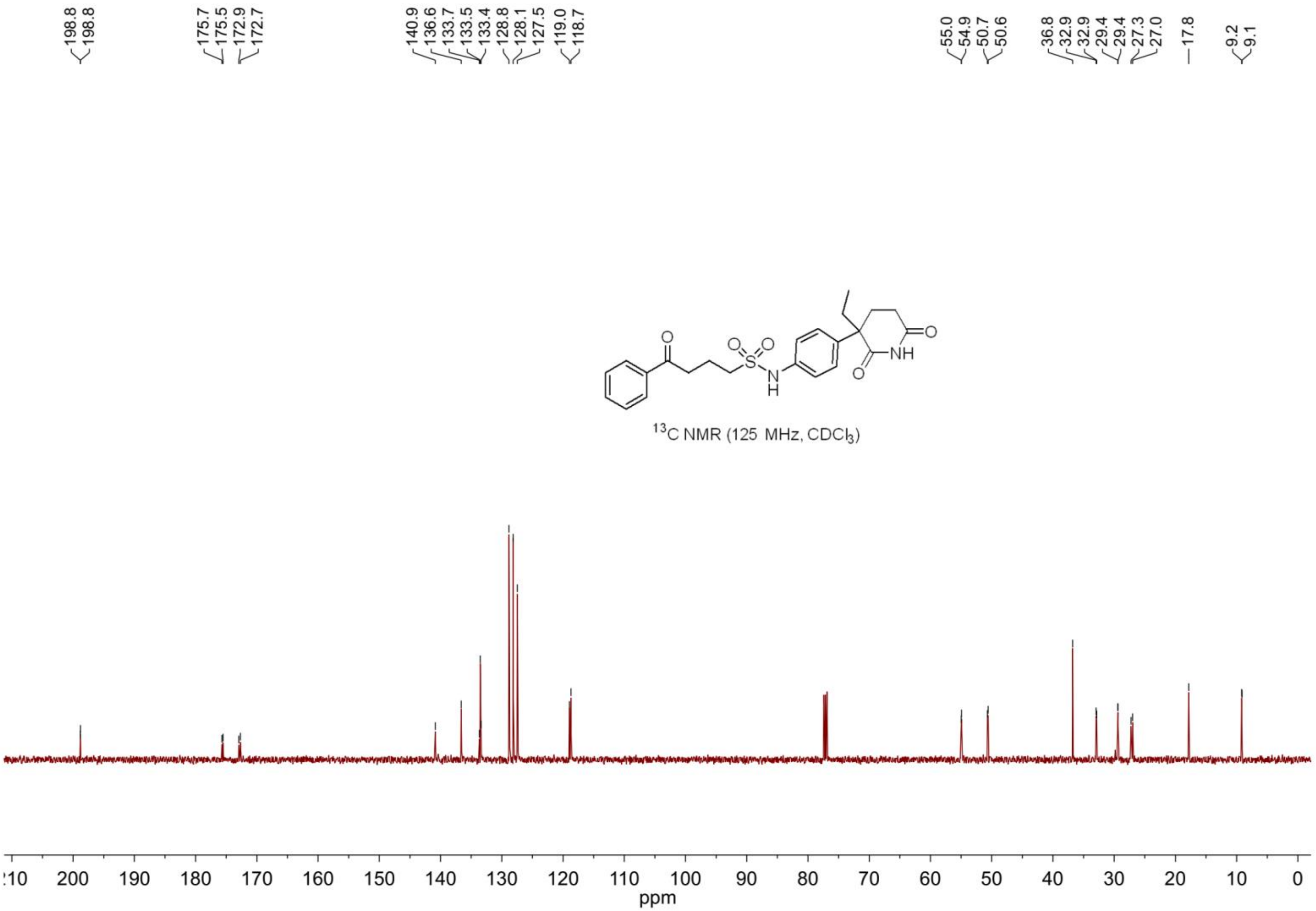

Go back to table of contents

Evaluation Only. Created with Aspose.Pdf. Copyright 2002-2014 Aspose Pty Ltd.

Benzyl (*S*)-4-(azidosulfonyl)-2-(((benzyloxy)carbonyl)amino)butanoate (10e)

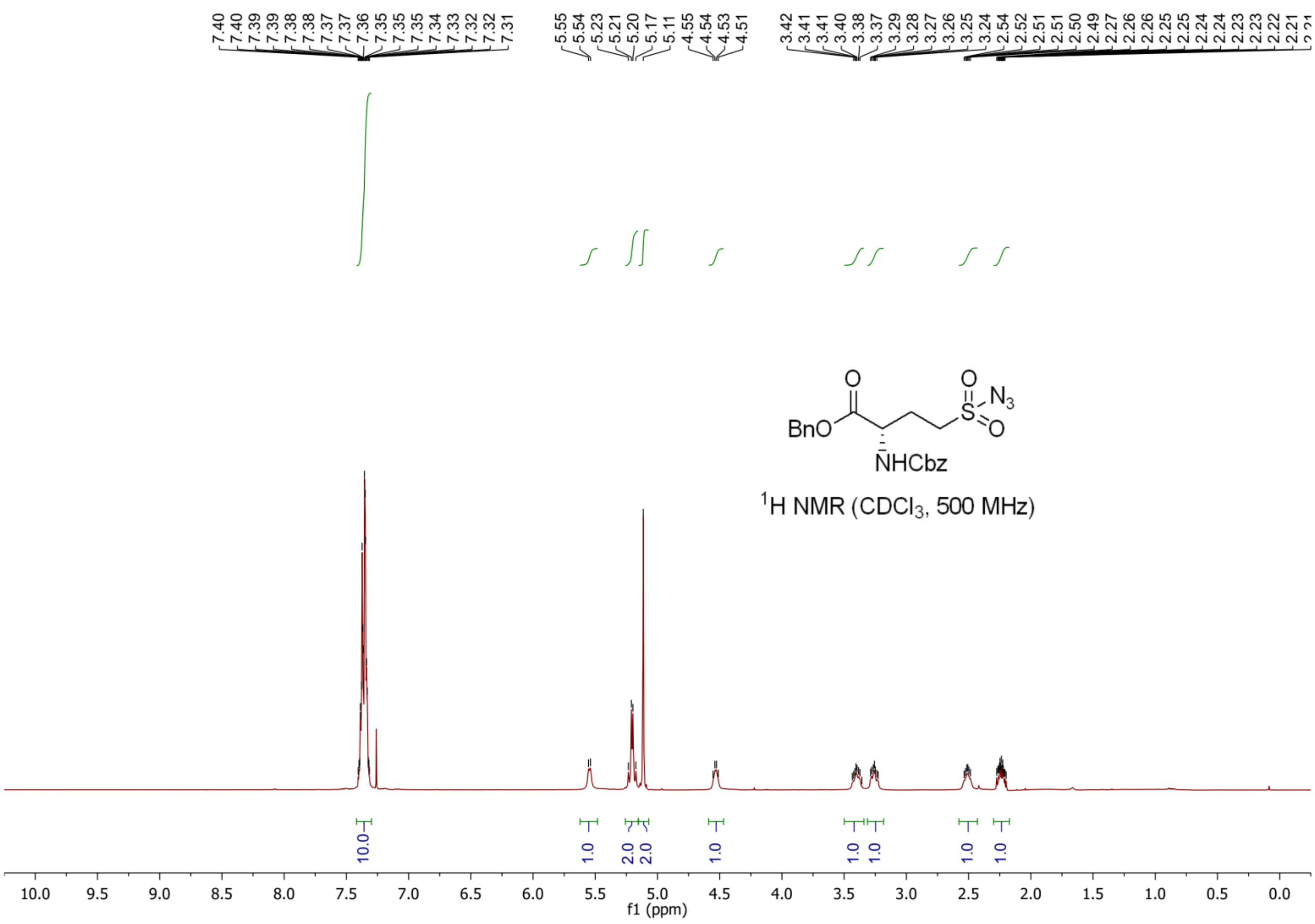

Go back to table of contents

Evaluation Only. Created with Aspose.Pdf. Copyright 2002-2014 Aspose Pty Ltd.

Benzyl (*S*)-4-(azidosulfonyl)-2-(((benzyloxy)carbonyl)amino)butanoate (10e)

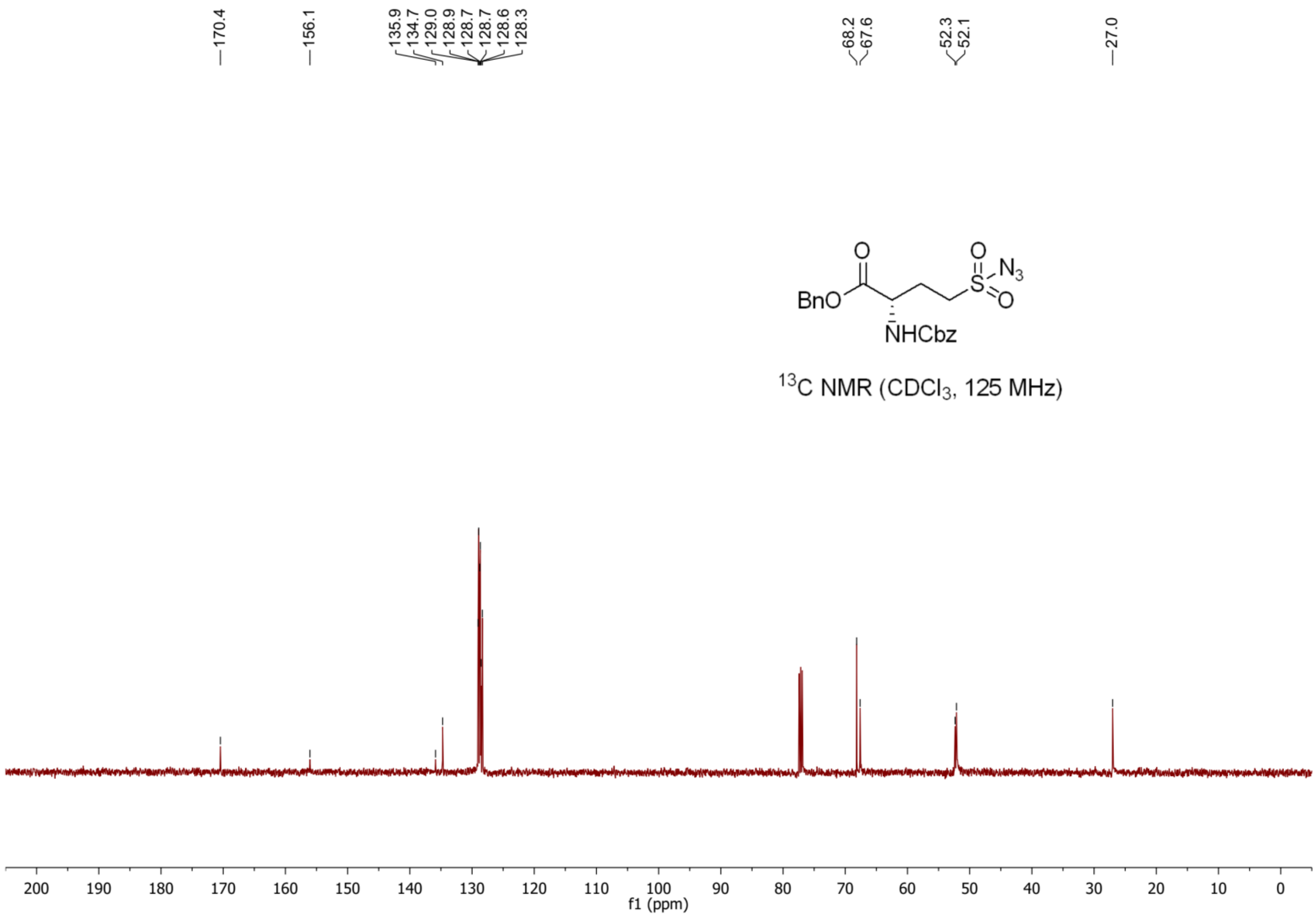

Go back to table of contents

Evaluation Only. Created with Aspose.Pdf. Copyright 2002-2014 Aspose Pty Ltd.

((3a*S*,5a*R*,8a*R*,8b*S*)-2,2,7,7-Tetramethyltetrahydro-3a*H*-bis([1,3]dioxolo)[4,5-*b*:4',5'-*d*]pyran-3a-yl)methyl 4-(azidosulfonyl)butanoate (10f)

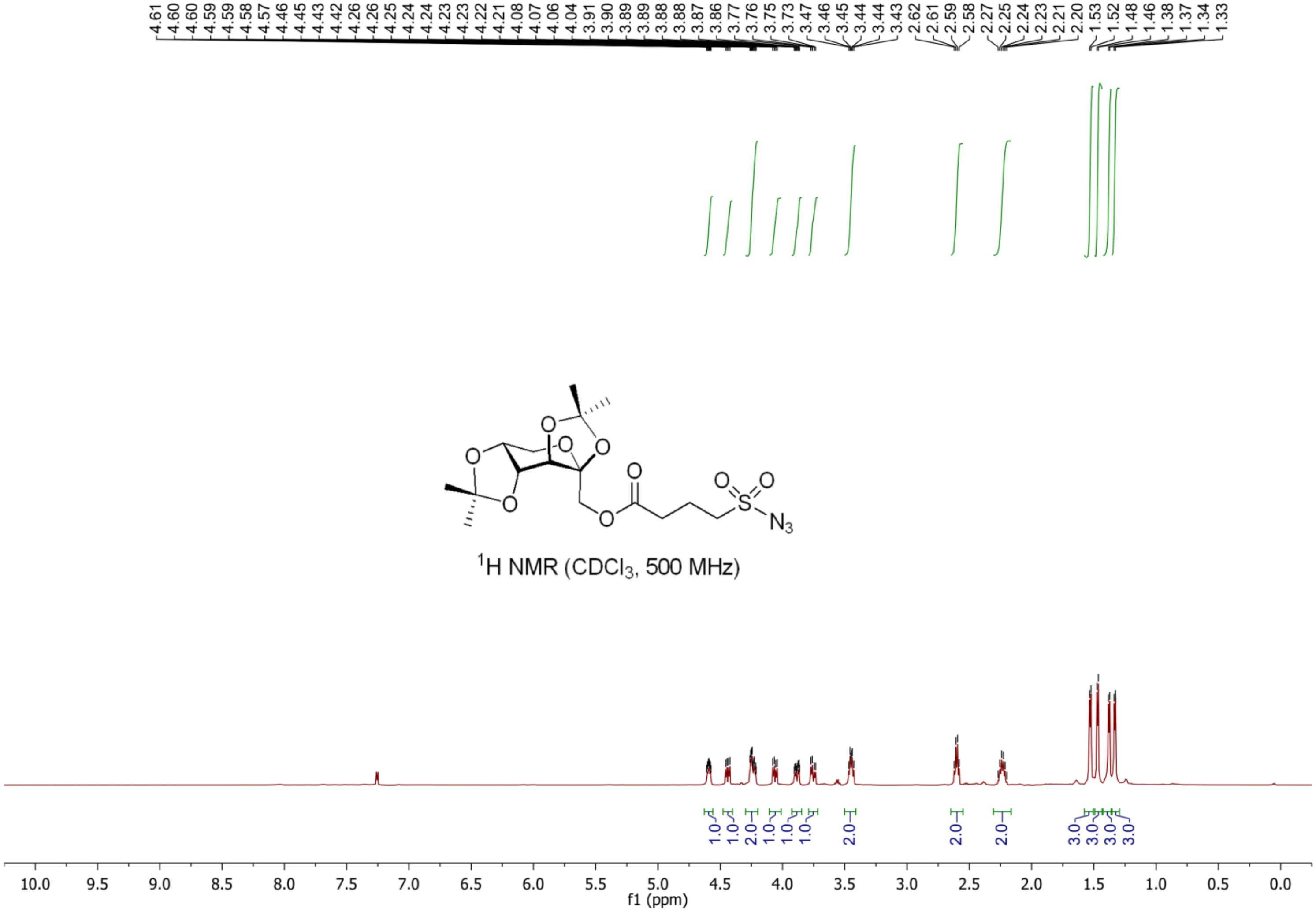

^{1}H NMR ($CDCl_3$, 500 MHz)

Go back to table of contents

Evaluation Only. Created with Aspose.Pdf. Copyright 2002-2014 Aspose Pty Ltd.

((3a*S*,5a*R*,8a*R*,8b*S*)-2,2,7,7-Tetramethyltetrahydro-3a*H*-bis([1,3]dioxolo)[4,5-*b*:4',5'-*d*]pyran-3a-yl)methyl 4-(azidosulfonyl)butanoate (10f)

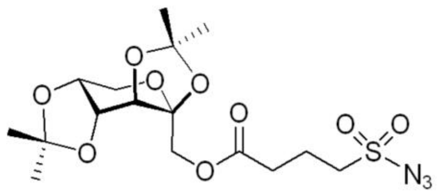

^{13}C NMR ($CDCl_3$, 125 MHz)

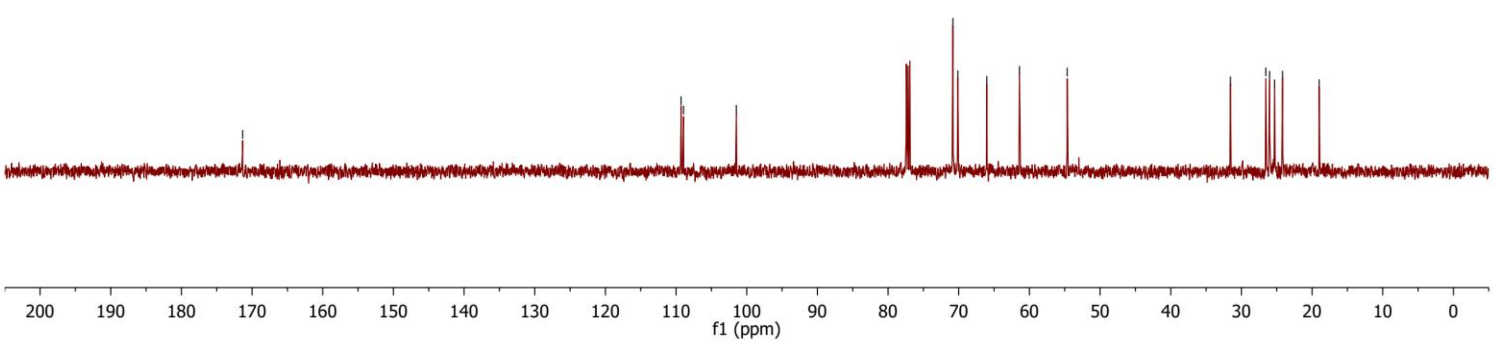

Go back to table of contents

Evaluation Only. Created with Aspose.Pdf. Copyright 2002-2014 Aspose Pty Ltd.

(1*S*,2*S*,4a*R*,4b*R*,7*S*,9a*S*,10*S*,10a*R*)-10-(Azidosulfonyl)-1-methyl-8-methylene-13-oxo-1,2,5,6,8,9,10,10a-octahydro-4a,1-(epoxymethano)-7,9a-methanobenzo[*a*]azulene-2,7(4b*H*)-diyl diacetate (10g)

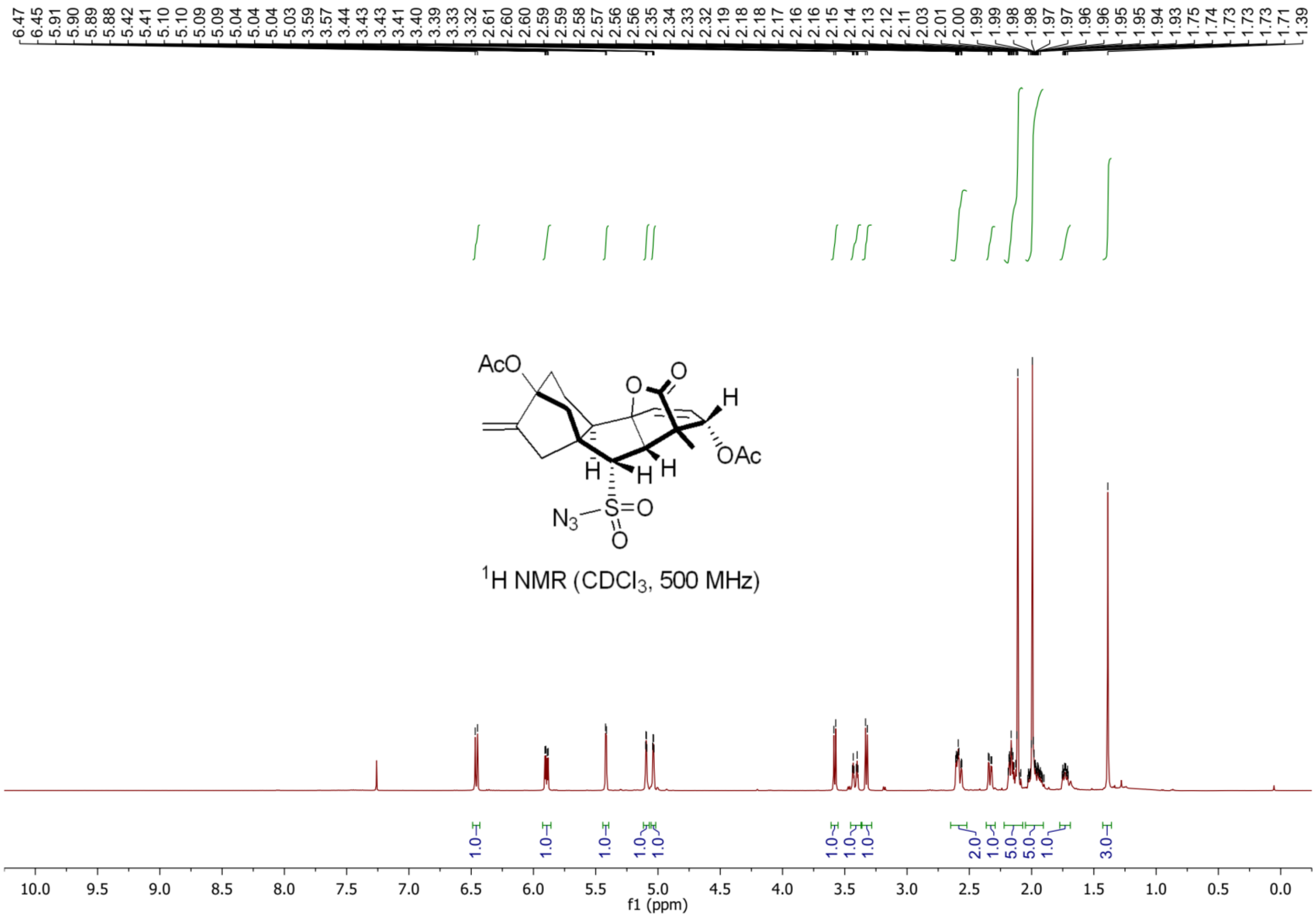

Go back to table of contents

Evaluation Only. Created with Aspose.Pdf. Copyright 2002-2014 Aspose Pty Ltd.

(1*S*,2*S*,4a*R*,4b*R*,7*S*,9a*S*,10*S*,10a*R*)-10-(Azidosulfonyl)-1-methyl-8-methylene-13-oxo-1,2,5,6,8,9,10,10a-octahydro-4a,1-(epoxymethano)-7,9a-methanobenzo[*a*]azulene-2,7(4b*H*)-diyl diacetate (10g)

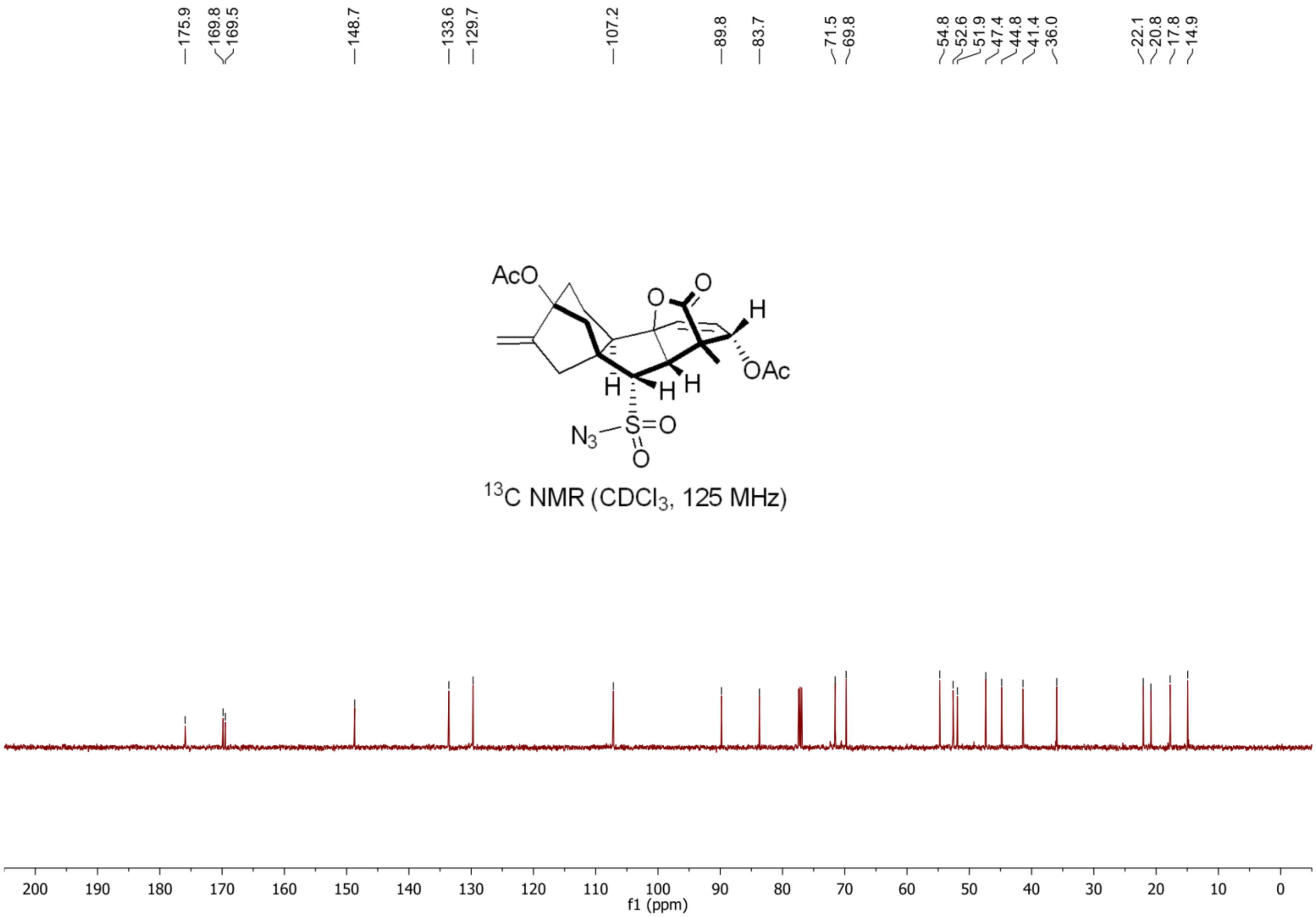

Go back to table of contents

Evaluation Only. Created with Aspose.Pdf. Copyright 2002-2014 Aspose Pty Ltd.

Evaluation Only. Created with Aspose.Pdf. Copyright 2002-2014 Aspose Pty Ltd.

((1*S*,3*R*)-3-Acetyl-2,2-dimethylcyclobutyl)methanesulfonyl azide (10h)

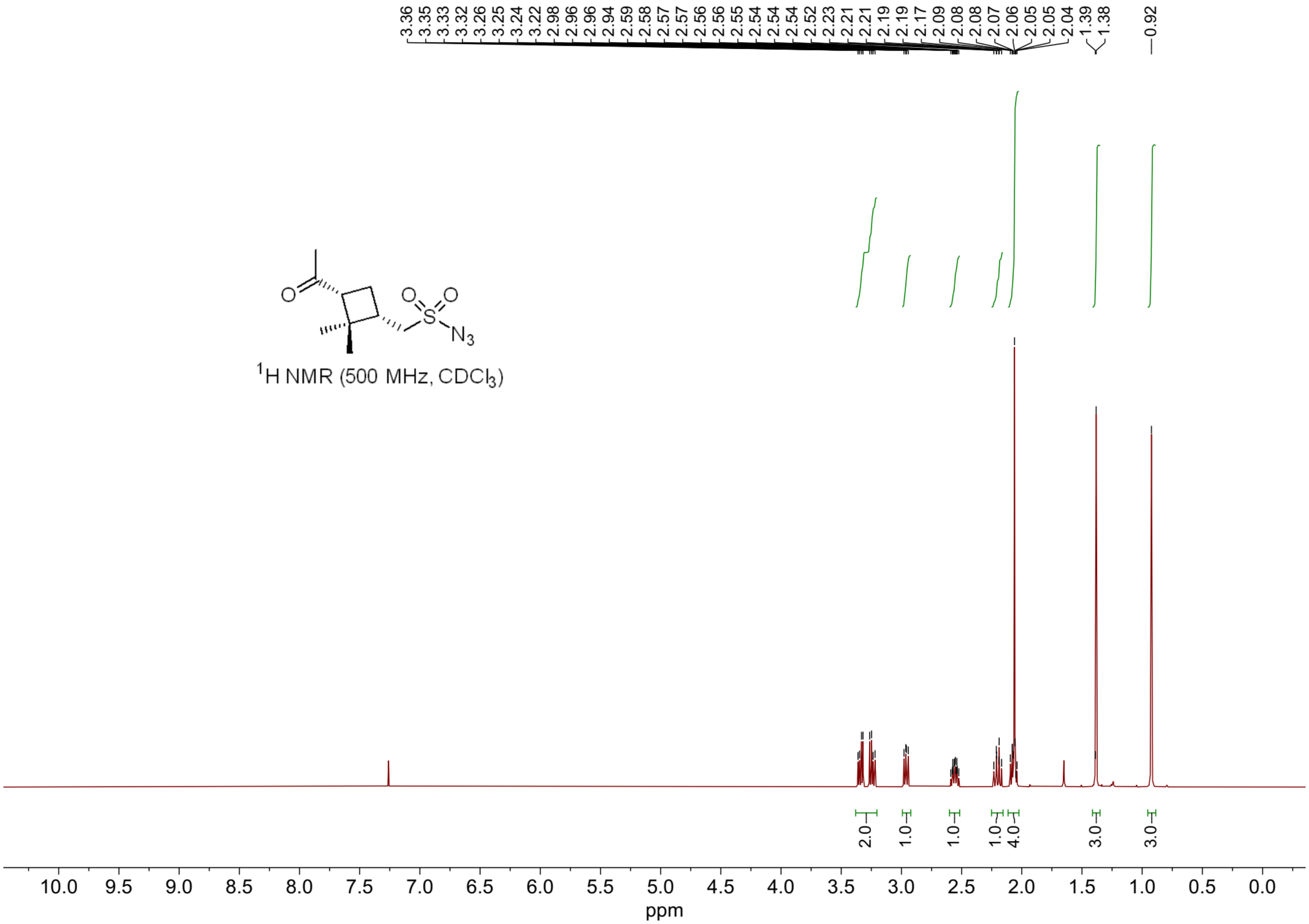

Go back to table of contents

Evaluation Only. Created with Aspose.Pdf. Copyright 2002-2014 Aspose Pty Ltd.

((1*S*,3*R*)-3-Acetyl-2,2-dimethylcyclobutyl)methanesulfonyl azide (10h)

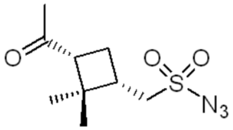

^{13}C NMR (125 MHz, $CDCl_3$)

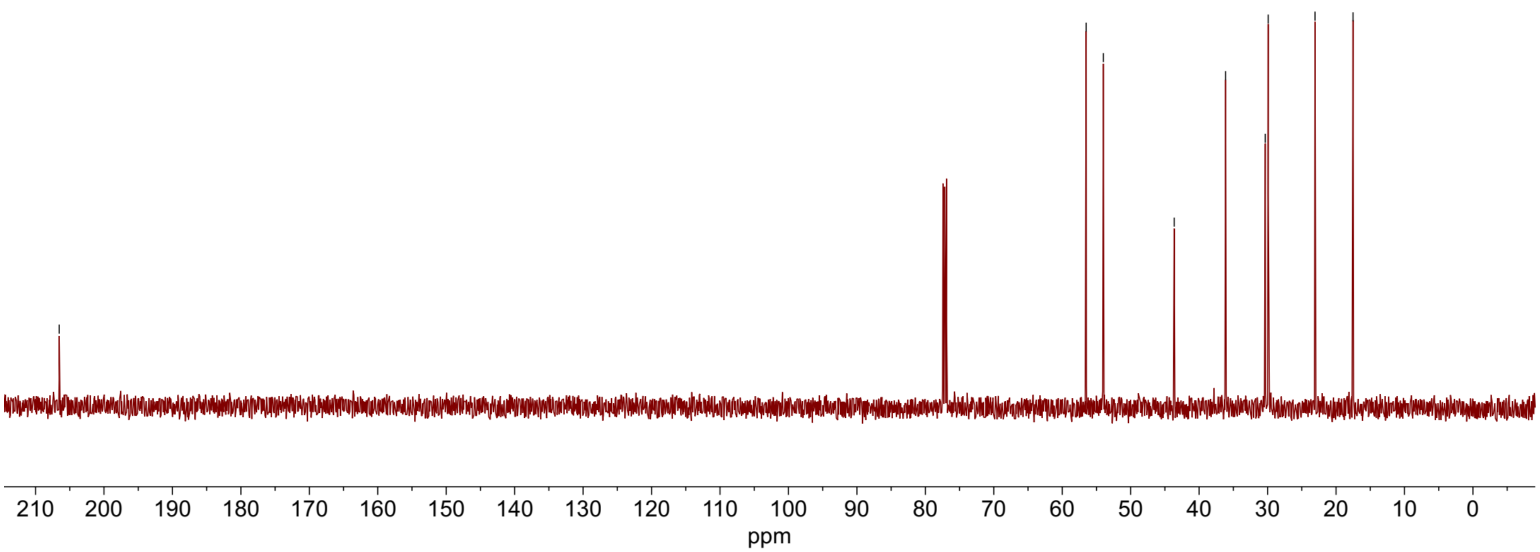

Go back to table of contents

Evaluation Only. Created with Aspose.Pdf. Copyright 2002-2014 Aspose Pty Ltd.

(3*R*,7*R*,8*R*,9*S*,10*S*,12*S*,13*R*,14*S*,17*R*)-17-((*R*)-4-(Azidosulfonyl)butan-2-yl)-10,13-dimethylhexadecahydro-1*H*-cyclopenta[*a*]phenanthrene-3,7,12-triyl triacetate (10i)

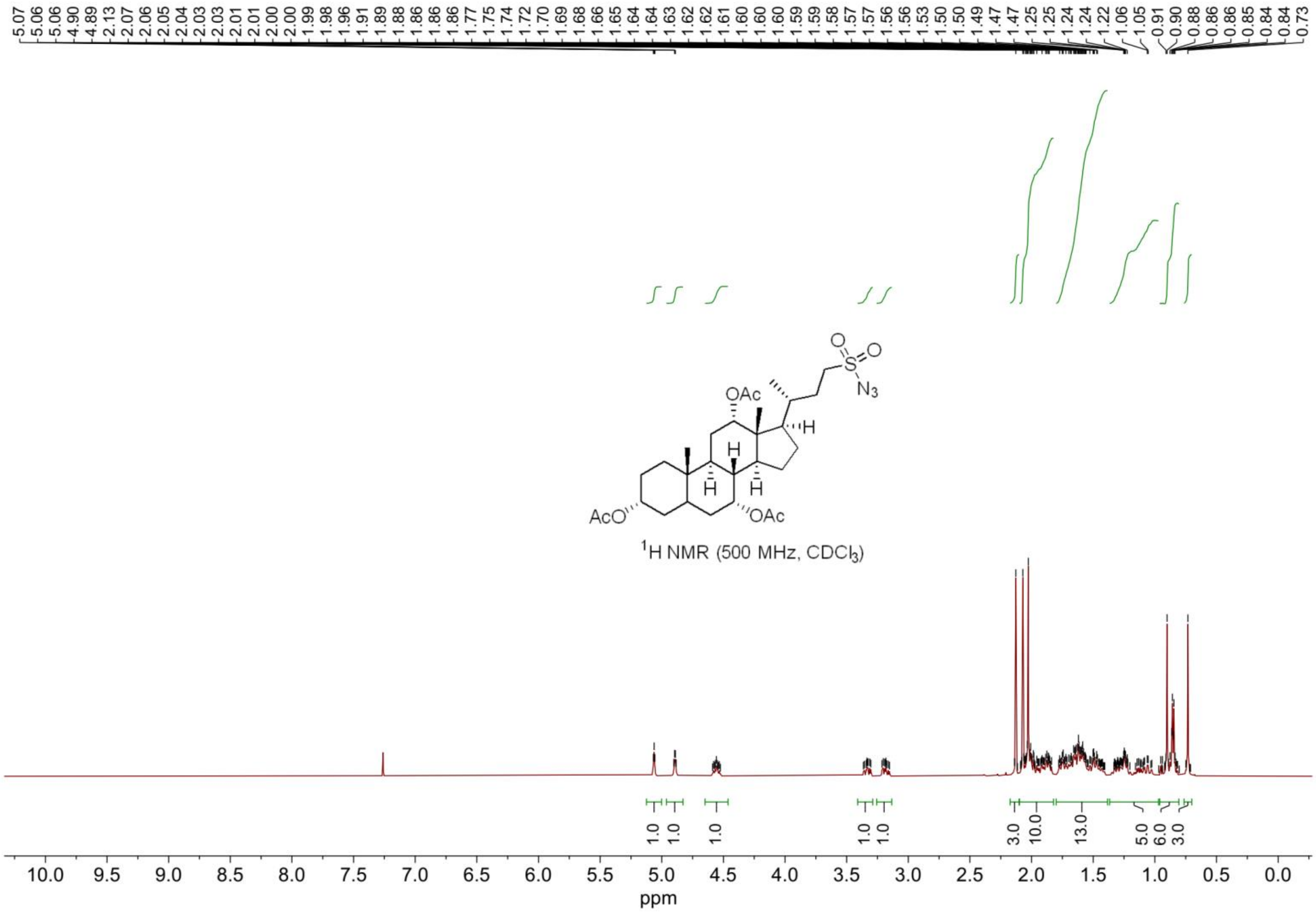

Go back to table of contents

Evaluation Only. Created with Aspose.Pdf. Copyright 2002-2014 Aspose Pty Ltd.

(3*R*,7*R*,8*R*,9*S*,10*S*,12*S*,13*R*,14*S*,17*R*)-17-((*R*)-4-(Azidosulfonyl)butan-2-yl)-10,13-dimethylhexadecahydro-1*H*-cyclopenta[*a*]phenanthrene-3,7,12-triyl triacetate (10i)

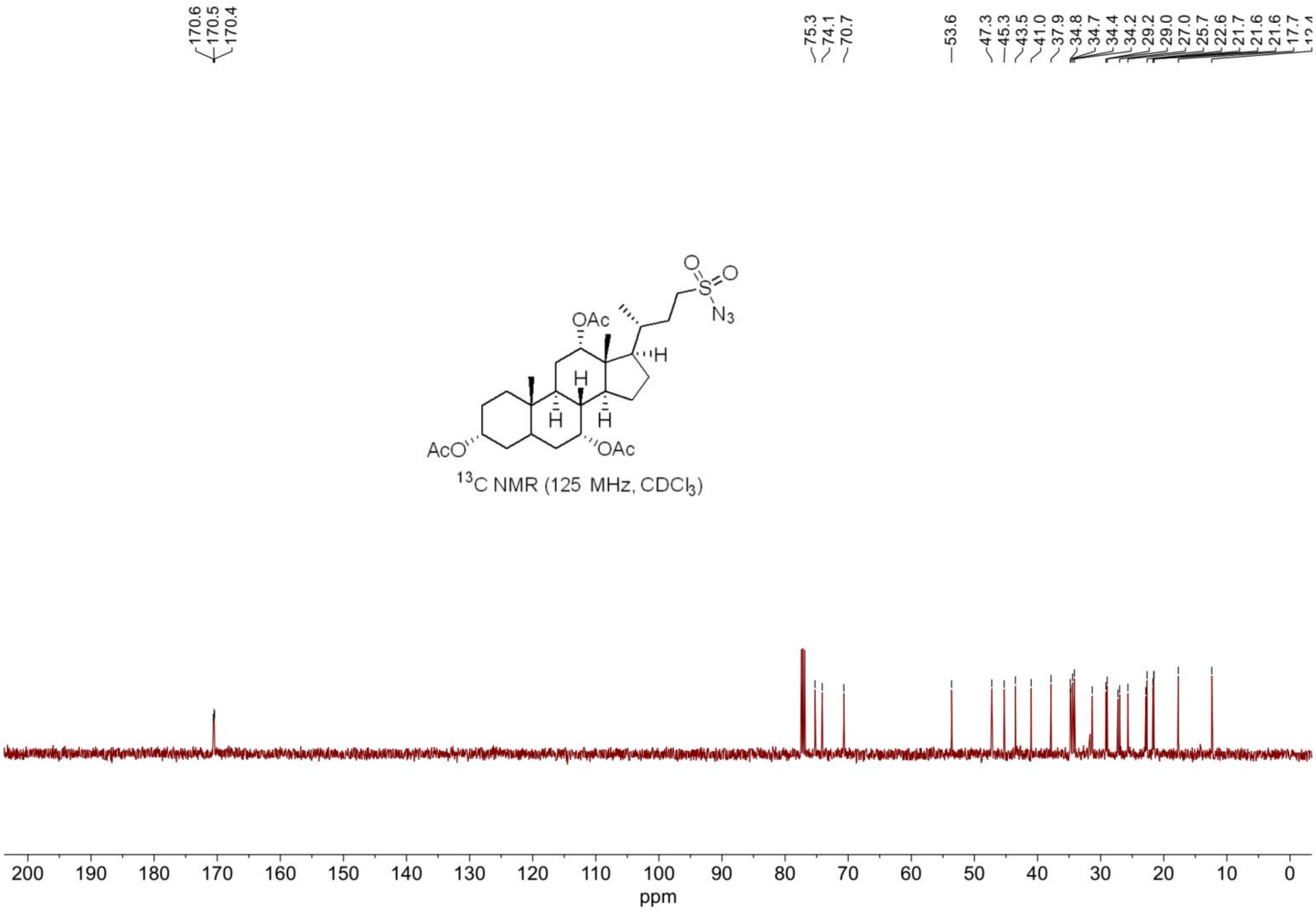

Go back to table of contents

Evaluation Only. Created with Aspose.Pdf. Copyright 2002-2014 Aspose Pty Ltd.

15-(Azidosulfonyl)pentadecane-1,7,8-triyl triacetate (10j)

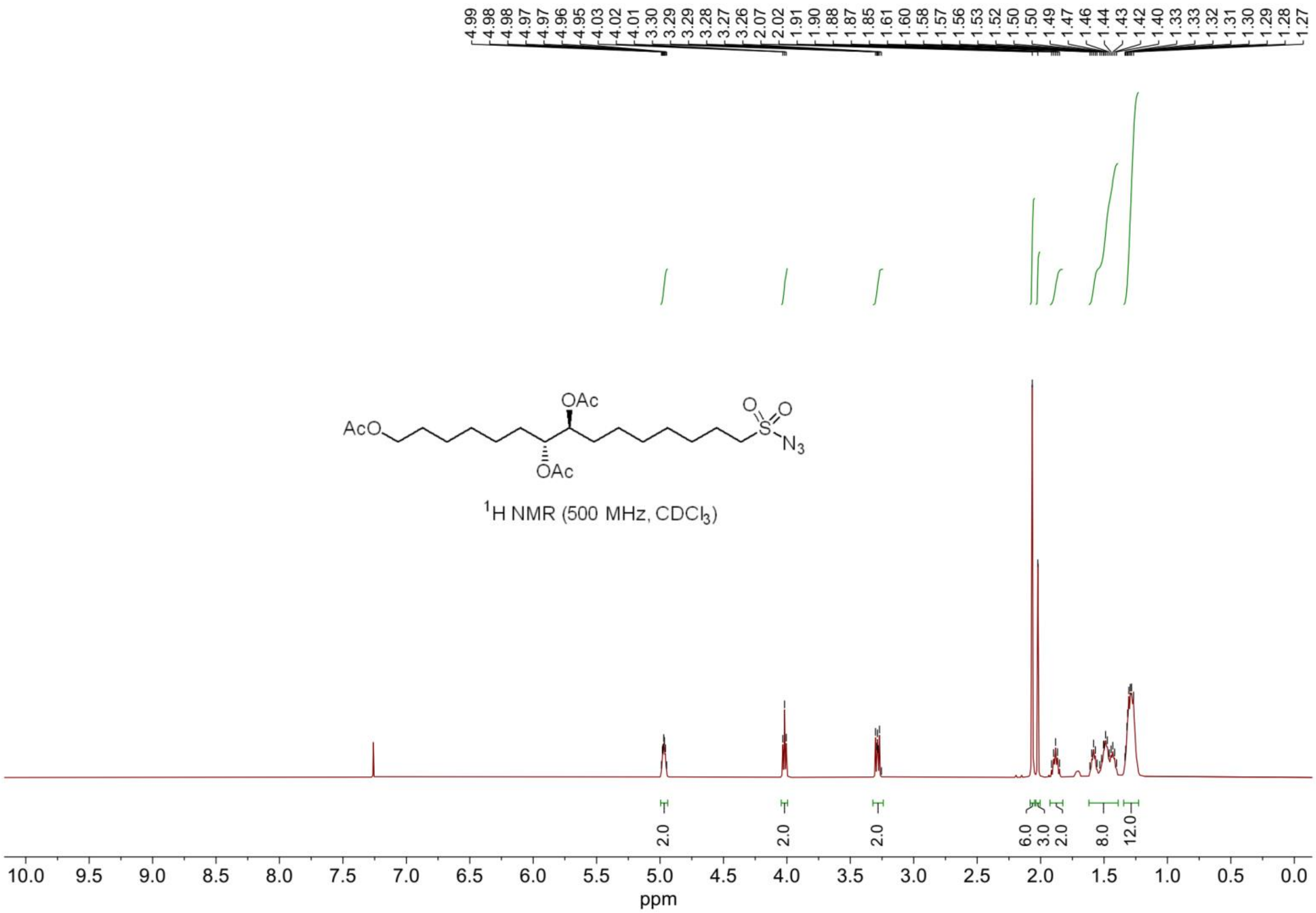

Go back to table of contents

Evaluation Only. Created with Aspose.Pdf. Copyright 2002-2014 Aspose Pty Ltd.

15-(Azidosulfonyl)pentadecane-1,7,8-triyl triacetate (10j)

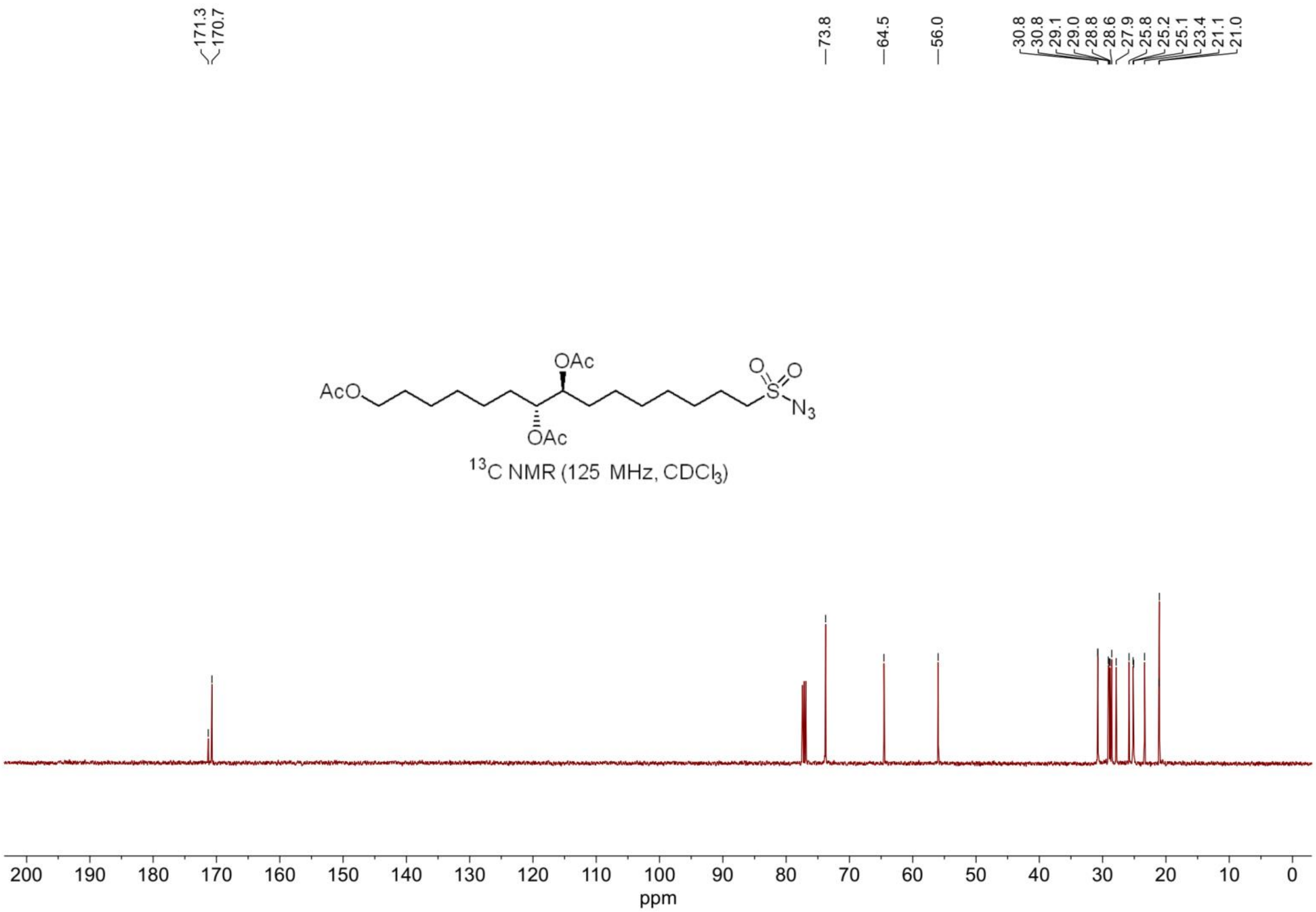

Go back to table of contents

Evaluation Only. Created with Aspose.Pdf. Copyright 2002-2014 Aspose Pty Ltd.

References

1 V. T. Nguyen, V. D. Nguyen, G. C. Haug, H. T. Dang, S. Jin, Z. Li, C. Flores-Hansen, B. S. Benavides, H. D. Arman, O. V. Larionov, *ACS Catal.*, 2019, **9**, 9485–9498.

2 S. Van Mileghem, W. M. De Borggraeve, *Org. Process Res. Dev.*, 2017, **21**, 785–787.

3 E. Brehm, R. Breinbauer, *Org. Biomol. Chem.*, 2013, **11**, 4750–4756.

4 H. Zhao, X. Guo, H. Tian, C. Li, Z. Xie, Y. Geng, F. Wang, *J. Mater. Chem.*, 2010, **20**, 3092–3097.

5 G. B. V. Subramanian, R. Sharma, *Synth. Commun.*, 1989, **19**, 1197–1202.

6 E. C. Liu, J. J. Topczewski, *J. Am. Chem. Soc.*, 2019, **141**, 5135–5138.

7 R. Thakare, H. Gao, R. E. Kosa, Y. A. Bi, M. V. Varma, M. A. Cerny, R. Sharma, M. Kuhn, B. Huang, Y. Liu, A. Yu, *Drug Metab. Dispos.*, 2017, **45**, 721–733.

8 H. T. Dang, G. C. Haug, V. T. Nguyen, N. T. H. Vuong, V. D. Nguyen, H. Arman, O. V. Larionov, *ACS Catalysis*, 2020, **10**, 11448–11457.

9 E. M. Espinoza, J. A. Clark, J. Soliman, J. B. Derr, M. Morales, V. I. Vullev, *J. Electrochem. Soc.*, 2019, **116**, H3175–H3187.

10 Gaussian 16, Revision B.01, M. J. Frisch, G. W. Trucks, H. B. Schlegel, G. E. Scuseria, M. A. Robb, J. R. Cheeseman, G. Scalmani, V. Barone, G. A. Petersson, H. Nakatsuji, X. Li, M. Caricato, A. V. Marenich, J. Bloino, B. G. Janesko, R. Gomperts, B. Mennucci, H. P. Hratchian, J. V. Ortiz, A. F. Izmaylov, J. L. Sonnenberg, D. Williams-Young, F. Ding, F. Lipparini, F. Egidi, J. Goings, B. Peng, A. Petrone, T. Henderson, D. Ranasinghe, V. G. Zakrzewski, J. Gao, N. Rega, G. Zheng, W. Liang, M. Hada, M. Ehara, K. Toyota, R. Fukuda, J. Hasegawa, M. Ishida, T. Nakajima, Y. Honda, O. Kitao, H. Nakai, T. Vreven, K. Throssell, J. A. Montgomery, Jr., J. E. Peralta, F. Ogliaro, M. J. Bearpark, J. J. Heyd, E. N. Brothers, K. N. Kudin, V. N. Staroverov, T. A. Keith, R. Kobayashi, J. Normand, K. Raghavachari, A. P. Rendell, J. C. Burant, S. S. Iyengar, J.

Evaluation Only. Created with Aspose.Pdf. Copyright 2002-2014 Aspose Pty Ltd.

Tomasi, M. Cossi, J. M. Millam, M. Klene, C. Adamo, R. Cammi, J. W. Ochterski, R. L. Martin, K. Morokuma, O. Farkas, J. B. Foresman, D. J. Fox, Gaussian, Inc., Wallingford CT, 2016.

11 Texas Advanced Computing Center (TACC), The University of Texas at Austin

12 P. Pracht, F. Bohle, S. Grimme, *Phys. Chem. Chem. Phys.*, 2020, **22**, 7169-7192.

13 S. Grimme, C. Bannwarth, P. Shushkov, *J Chem. Theory Comput.*, 2017, **13**, 1989-2009.

14 C. Bannwarth, S. Ehlert, S. Grimme, *J. Chem. Theory Comput.*, 2019, **15**, 1652-1671.

15 Chemcraft - graphical software for visualization of quantum chemistry computations. https://www.chemcraftprog.com

16 A. Hansen, C. Bannwarth, S. Grimme, P. Petrović, C. Werlé, J.-P. Djukic, *ChemistryOpen*, 2014, **3**, 177–189.

17 a) G. Luchini, J. V. Alegre-Requena, Y. Guan, I. Funes-Ardoiz, R. S. Paton, GoodVibes: GoodVibes v3.0.1 (2019); b) G. Luchini, J. V. Alegre-Requena, Y. Guan, I. Funes-Ardoiz, R. S. Paton, *F1000Research*, 2020, **9**, 291.

18 CYLview, 1.0b; C. Y. Legault, Université de Sherbrooke, 2009 (http://www.cylview.org).

19 W. Humphrey, A. Dalke, K. Schulten, *J. Molec. Graphics*, 1996, **14**, 33–38.

20 S. Grimme, J. Antony, S. Ehrlich, H. Krieg, *J. Chem. Phs.*, 2010, **132**, 154104.

21 S. Grimme, S. Ehrlich, L. Goerigk, *J. Comput. Chem.*, 2011, **32**, 1456–1465.

22 Y. Zhao, D. G. Truhlar, *J. Phys. Chem. A*, 2005, **109**, 5656–5667.

23 L. Goerigk, A. Hansen, C. Bauer, S. Ehrlich, A. Najibi, S. Grimme, *Phys. Chem. Chem. Phys.*, 2017, **19**, 32184.

24 F. Weigend, R. Ahlrichs, *Phys. Chem. Chem. Phys.*, 2005, **7**, 3297–3305.

25 A. V. Marenich, C. J. Cramer, D. G. Truhlar, *J. Phys. Chem. B*, 2009, **113**, 6378–6396.

Evaluation Only. Created with Aspose.Pdf. Copyright 2002-2014 Aspose Pty Ltd.

26 B. P. Pritchard, D. Altarawy, B. Didier, T. D. Gibson, T. L. Windus, *J. Chem. Inf. Model.*, 2019, **59**, 4814–4820.

27 H. T. Dang, G. C. Haug, V. T. Nguyen, N. T. H. Vuong, V. D. Nguyen, H. D. Arman, O. V. Larionov, *ACS Catal.*, 2020, **10**, 11448–11457.

28 A. Weller, *Electrochem.*, 1952, **56**, 662–668.

29 M. Lõkov, S. Tshepelevitsh, A. Heering, P. G. Plieger, R. Vianello, I. Leito, *Eur. J. Org. Chem.*, 2017, **2017**, 4475–4489.
